# Intensive tropical land use massively shifts soil fungal communities

**DOI:** 10.1038/s41598-019-39829-4

**Published:** 2019-03-04

**Authors:** Nicole Brinkmann, Dominik Schneider, Josephine Sahner, Johannes Ballauff, Nur Edy, Henry Barus, Bambang Irawan, Sri Wilarso Budi, Matin Qaim, Rolf Daniel, Andrea Polle

**Affiliations:** 1 Forest Botany and Tree Physiology, University of Goettingen, Göttingen, Germany; 2 Genomic and Applied Microbiology and Göttingen Genomics Laboratory, University of Goettingen, Göttingen, Germany; 3 Department of Agrotechnology, Faculty of Agriculture, Tadulako University, Palu, Indonesia; 4 Department of Forestry, University of Jambi, Jambi, Indonesia; 5 Department of Silviculture, Faculty of Forestry, Bogor Agriculture University, Bogor, Indonesia; 6 Department of Agricultural Economics and Rural Development, University of Goettingen, Göttingen, Germany

## Abstract

Soil fungi are key players in nutrient cycles as decomposers, mutualists and pathogens, but the impact of tropical rain forest transformation into rubber or oil palm plantations on fungal community structures and their ecological functions are unknown. We hypothesized that increasing land use intensity and habitat loss due to the replacement of the hyperdiverse forest flora by nonendemic cash crops drives a drastic loss of diversity of soil fungal taxa and impairs the ecological soil functions. Unexpectedly, rain forest conversion was not associated with strong diversity loss but with massive shifts in soil fungal community composition. Fungal communities clustered according to land use system and loss of plant species. Network analysis revealed characteristic fungal genera significantly associated with different land use systems. Shifts in soil fungal community structure were particularly distinct among different trophic groups, with substantial decreases in symbiotrophic fungi and increases in saprotrophic and pathotrophic fungi in oil palm and rubber plantations in comparison with rain forests. In conclusion, conversion of rain forests and current land use systems restructure soil fungal communities towards enhanced pathogen pressure and, thus, threaten ecosystem health functions.

## Introduction

Tropical rain forests are the planet’s most species-rich biomes^1^. In the past two decades, tropical rain forests in many parts of the world have been rapidly converted to monospecific plantations^2^. As a result, deforestation and human land use have irretrievably destroyed large areas of unique rain forests and enforced biodiversity loss^3,4^. High plant diversities are associated with active, abundant and diverse fungal communities^5–7^. Plant diversity was, therefore, predicted to be a strong driver of fungal species richness in soils of tropical rain forests^7–9^. However, the consequences of rain forest transformation into agricultural land for soil fungal diversity and the ecological functions of these fungi are not well understood.

Soil fungi are integral components of ecosystems, driving nutrient cycling as decomposers^10–13^, regulating species composition as pathogens^14^ and providing mutualistic benefits as symbiotrophs, thereby playing a key role in biogeochemical processes^15,16^ and in soil health^17,18^. Because of their important functions, the impact of deforestation and land use intensification on soil fungal communities in the tropics is receiving increasing attention. To date, only a few studies have used next-generation sequencing methods to characterize soil fungal communities after the conversion of rain forests into agricultural land^19–22^. Those studies focused mainly on distinct fungal groups such as mycorrhizae and the turnover of their community structure in response to distinct land use systems such as the conversion of rain forest into pasture or cash crop plantations with oil palms or rubber trees^19–22^. However, approaches linking land use systems or aboveground vegetation diversity with soil fungal richness and diversity are rare^23^. To better understand the consequences of forest conversion for the taxonomic and functional composition of soil fungi, studies across different landscapes in relation to land use systems and plant diversity loss are required. Such approaches are urgently needed for recommendations for sustainable land use^24^.

Here, we investigated the impact of land use systems and loss of plant richness on soil fungal diversity in southeast Asia, where natural forests are transformed into cash crop plantations at unprecedented rates^25,26^. Loss of natural forest resources is particularly strong in Indonesia, which is currently the world’s largest palm oil producer^27^ and is second in rubber production^28^. We conducted our study in two regions in Sumatra (Indonesia), selecting unmanaged lowland rain forests, moderately managed jungle rubber agroforests and intensively managed plantations, where oil palm (*Elaeis guineensis* Jacq.) or rubber (*Hevea brasiliensis* Kunth. Muell.) were the only tree species. Oil palm and rubber plantations are fertilized with 300 to 600 kg ha^−1^ yr^−1^ and 100 to 300 kg ha^−1^ yr^−1^ inorganic NPK fertilizer, respectively. Other inputs are cow dung and lime, in addition to herbicide treatments twice a year^29^. We used these inputs and planting intensities to calculate indices for land use intensity. Previous investigations in our study regions showed that plant species richness and above- and belowground plant biomass are reduced in oil palm and rubber plantations compared with rain forests^4,30^. We expected that the replacement of the hyperdiverse forest flora by nonendemic oil palm and rubber trees resulted in drastic diversity losses of soil fungal taxa because plant communities structure soil habitats^31–33^. We used vegetation richness and indicators of land use to test the hypothesis that soil fungal species richness is driven by land use intensity^34^ and by loss of plant species richness. We further tested the hypothesis that intensive land use in monospecific plantations leads to changes in the ecological functions of soil fungi. To investigate this proposition, we categorized soil fungi according to their trophic mode into guilds (symbiotrophic, pathotrophic and saprotrophic), studied shifts in the composition of the ecological fungal groups and employed network analyses to uncover indicator fungi for different land use systems.

## Materials and Methods

### Study sites and sampling design

The study sites were located in two different landscapes (Harapan Rain Forest, National Park Bukit 12) in the Jambi Province of Sumatra, Indonesia. Both landscapes have been previously described based on latitude and longitude, soil type, climate, rainfall, annual precipitation, temperature and vegetation^33,35–37^. Details of our sampling design have been described by Sahner *et al*.^33^. In brief, in each landscape, four land use systems (secondary rain forest, rubber agroforest (jungle rubber), rubber plantations and oil palm plantations) were selected. In each land use system, four plots (50 × 50 m) were installed, resulting in a total of 32 sampling plots. In each plot, three subplots of 5 × 5 m were selected. To account for heterogeneity, in each subplot five soil cores (0.04 m diameter and 0.20 m depth) were extracted (one close to each corner and one in the center of the subplot). Soil cores were stored individually in plastic bags that were then stored in cool bags and transported to the University of Jambi, where they were stored at 4 °C until further processing. Each soil core was weighed and consecutively sieved through two sieves with 10 and 5 mm mesh size, and bulk soil was separated from roots. The five samples from the same subplot were pooled and well mixed, yielding one bulk soil sample per subplot^33^. To freeze dry the bulk soil samples, the reaction tubes (50 ml, Sarstedt, Nümbrecht, Germany) containing bulk soil were opened, and gauze was put into the aperture of each tube to avoid loss of bulk soil during freeze drying. Reaction tubes containing bulk soil were put on a rack and placed in a −80 °C freezer for at least 3 hours before freeze drying to make sure that the bulk soil had a sufficiently low temperature. Freeze drying was performed using a VirTis Benchtop K Freeze Dryer (SP Industries, Warminster, USA) with a dual stage rotary vane vacuum pump (Trivac E2, Leybold Vakuum GmbH, Köln, Germany) for approximately 32 hours. Afterwards, three perforated Eppendorf tubes filled with 5 g of silica gel (desiccant bag of silica gel orange (10 g (40 × 90 mm)), Carl Roth, Karlsruhe, Germany) were put into the reaction tubes to keep the soil samples dry. The freeze-dried bulk soil samples were shipped to the University of Göttingen. A research permit (Kartu Izin Peneliti Asing, permission number: 333/SIP/FRP/SM/IX/2012) was issued by the Ministry of Research and Technology RISTEK (Kementrian Ristek dan Teknologi, Jakarta, Indonesia). The Research Center for Biology of the Indonesian Institute of Science LIPI (Lembaga Ilmu Pengetahuan Indonesia, Jakarta, Indonesia) recommended issuing a sample collection permit (Rekomendasi Ijin Pengambilan dan Angkut (SAT-DN) Sampel Tanah dan Akar, number: 2696/IPH.1/KS:02/XI/2012). The collection permit (number: S.16/KKH-2/2013) and export permit (reference number: 48/KKH-5/TRP/2014) were issued by the Directorate General of Forest Protection and Nature Conservation PHKA (Perlindungan Hutan dan Konservasi Alam, Jakarta, Indonesia) under the Ministry of Forestry of the Republic of Indonesia. The Chamber of Agriculture of Lower Saxony (Plant Protection Office, Hannover, Germany) issued the import permits (Letter of Authority, numbers: DE-NI-12-69 -2008-61-EC, DE-NI-14-08-2008-61-EC).

### Fungal community analysis

The freeze-dried soil samples were stored at −20 °C. They were homogenized in a Type MM400 ball mill (Retsch GmbH, Haan, Germany) in liquid nitrogen. DNA isolations were conducted using 250 mg soil, which was further homogenized with glass beads (MO BIO Laboratories Inc., Carlsbad, USA) and used for DNA extraction with a PowerSoil® DNA Isolation Kit (MO BIO Laboratories Inc.), following the manufacturer’s recommendations. DNA yields were estimated by using a NanoDrop ND-1000 spectrophotometer (PEQLAB Biotechnologie GmbH, Erlangen, Germany). For each DNA extraction, polymerase chain reaction (PCR) was performed in a 50 µl reaction using 0.5 μl of Phusion High-Fidelity DNA Polymerase (2 U/μl, New England Biolabs (NEB), Frankfurt, Germany), 10 μl of 5x Phusion GC buffer (NEB), 0.15 μl of MgCl_2_ (50 mM, NEB), 2.5 µl of DMSO (5%, NEB), 2.5 µl of bovine serum albumin (8 mg/ml, Merck KGaA, Darmstadt, Germany), 1 μl of dNTP mix (10 mM each, Thermo Fisher Scientific, Osterode am Harz, Germany), 1 μl of each primer (10 mmol/l, Microsynth, Wolfurt, Austria) and 5 μl of template DNA. PCR reactions were performed in a Labcycler (SensoQuest, Göttingen, Germany). The cycling parameters were 1 cycle of 98 °C for 3 min; 25 cycles of 98 °C for 10 s, 47 °C for 20 s and 72 °C for 20 s; and a final extension at 72 °C for 5 min. The primers ITS1-F_KYO1^38^ and ITS4^39^ including the Roche 454 pyrosequencing adaptors (Roche, Mannheim, Germany), a key (TCAG), and a variable multiplex identifier (MID) consisting of ten bases were used for amplification of the ITS 1 and 2 regions. PCR products were subjected to electrophoresis in 1.2% agarose gels (Biozym LE Agarose, Biozym Scientific GmbH, Hessisch Oldendorf, Germany) using GelRed (10 000×) to stain (VWR, Darmstadt, Germany) a 1 kb DNA ladder (NEB) for estimation of the product size. PCR products were visualized with an FLA-5100 Fluorescence Laser Scanner (Raytest GmbH, Straubenhardt, Germany) and Aida Image Analyser v. 4.27 (Raytest GmbH). All PCR reactions were performed in triplicate, pooled and purified using an innuPREP PCRpure Kit (Analytik Jena, Jena, Germany). Purified, pooled PCR products were run on an agarose gel and cut in the range of 700–800 base pairs on a UV table (INTAS UV System type N80M, Göttingen, Germany). A QIAquick Gel extraction kit (Qiagen GmbH, Hilden, Germany) was used for DNA extraction following the manufacturer’s recommendations; each sample was eluted in 20 µl of nuclease-free water (AppliChem, Darmstadt, Germany). Quantification of purified PCR products was performed using a Quant-iT dsDNA HS assay kit (Life Technologies GmbH, Darmstadt, Germany) in a Qubit fluorometer (Life Technologies GmbH, Darmstadt, Germany) following the manufacturer’s recommendations. The Göttingen Genomics Laboratory (G2L) determined the sequences of ITS amplicons by using a 454 GS-FLX sequencer (Roche, Mannheim, Germany) and Titanium chemistry following the instructions of the manufacturer for amplicon sequencing.

### Sequence processing

The resulting ITS sequence datasets were quality filtered and primer clipped by employing split_libraries.py from the QIIME 1.9.1 software package^40^. In brief, sequences with lengths below 300 and over 1000 bp, quality scores below 25 and homopolymer stretches of more than 8 bp were removed. An additional primer clipping was performed with cutadapt v1.6^41^. Pyrosequencing noise was removed by employing Acacia v1.53^42^ with default settings. High-quality reads were further processed with USEARCH (version 8.0.1623_i86linux64)^43^, which included steps in the following order: reference-based removal of chimeric sequences against the unite database (v7.0, sh_refs_qiime_ver7_99_s_01.08.2015.fasta)^44^, sequence sorting by length and singleton removal, OTU determination at 97% sequence identity (*pick_open_reference_otus.py*) employing the unite database. Taxonomic classification of OTU sequences was inferred with *parallel_assign_taxonomy_blast.py* against the UNITE database (v7, sh_refs_qiime_ver7_99_s_01.08.2015.fasta)^44,45^. Taxonomic information was added to the OTU table with *make_otu_table.py* (QIIME)^46^. Unclassified OTUs and extrinsic domain OTUs (Protista, Plantae) were removed from the table by employing *filter_otu_table.py* in QIIME. Raw sequences for the resulting fungal OTUs were deposited in the National Center for Biotechnology Information (NCBI) Sequence Read Archive (SRA) under accession number SRP134264 (Bioproject number PRJNA437389). Sampling effort was controlled by rarefaction analysis (Supplementary Data S1) by employing *alpha_rarefaction.py*. Fungal OTUs were assigned to ecological guilds with the annotation tool FUNGuild^47^, located at https://github.com/UMNFuN/FUNGuild. Here, fungi were grouped according to trophic modes: saprotrophic, pathotrophic and symbiotrophic fungi, while fungal sequences without assignment were labeled ‘unknown’ in further analyses^40^.

### Statistical analysis

Sample comparisons were performed for the same surveying effort using rarefied data sets of 1229 sequences representing the lowest number of reads in a sample. Diversity estimates (OTU richness, Michaelis-Menten fit, Chao1, Shannon, Simpson) and rarefaction curves were generated by employing the *alpha_rarefaction.py*. script in QIIME^40^. Differences among land use systems were analyzed by linear mixed effect models with the *lmer* function of the multcomp package^48^ in R (R Core Team, 2015), because the data follow a Gaussian distribution. Generalized mixed effect models with landscape as a random effect were used with the glmer function of the multcomp package to investigate differences in fungal α-diversity among land use systems. For displaying the most abundant fungal species all unidentified fungal OTUs were removed and a heatmap was created with ampvis2^49^, color scale of abundance was square rooted to better visualize low abundant species. Nonmetric multidimensional scaling (NMDS) of fungal communities was conducted in R using the vegan package^50^, based on weighted UniFrac^51^ distance matrices, and used envfit (vegan) to correlate the following explanatory variables: root performance traits (biomass of fine roots, distorted root tips)^33^, plant properties (plant biomass, plant species)^4^, soil properties (soil pH value; soil moisture; and concentrations of magnesium, potassium, carbon, nitrogen, calcium, and available phosphorous^32,34^, litter properties (concentration of carbon and nitrogen)^33^, diversity index (number of OTUs), and land use (land use intensity, LUI)^33^ with the fungal community. The index for tropical land use intensity was developed according to the method of Blüthgen *et al*.^52^. The land use index (LUI) was calculated separately for each landscape, where each component of land treatment was standardized relative to its mean:

$$LUI=\sqrt{\tfrac{F{1}_{i}}{F{1}_{L}}+\tfrac{F{2}_{i}}{F{2}_{L}}+\tfrac{F{3}_{i}}{F{3}_{L}}+\tfrac{F{4}_{i}}{F{4}_{L}}+\tfrac{F{5}_{i}}{F{5}_{L}}+\tfrac{F{6}_{i}}{F{6}_{L}}+\tfrac{F{7}_{i}}{F{7}_{L}}+\tfrac{H{1}_{i}}{H{1}_{L}}+\tfrac{H{2}_{i}}{H{2}_{L}}+\tfrac{H{3}_{i}}{H{3}_{L}}+\tfrac{H{4}_{i}}{H{4}_{L}}+\tfrac{{S}_{i}}{{S}_{L}}+\tfrac{{A}_{i}}{{A}_{L}}+\tfrac{P{1}_{i}}{P{1}_{L}}+\tfrac{P{2}_{i}}{P{2}_{L}}}$$

with F_L_, H_L_, S_L_, A_L_ and P_L_ being the treatment means. The quantities (kg year^−1^, L year^−1^) of fertilizers (F1: urea, F2: potassium chloride, F3: borate, F4: nitrogen-phosphorus-potassium, F5: triple superphosphate, F6: 36% superphosphate (SP 36), F7: kieserite), herbicides (H1: Gramaxon, H2: Noxone, H3: Roundup, H4: Ally), animal manure (A1: cow compost), and liming (S1: CaCO_3_) applied to the study plots were obtained on the basis of interviews of farmers^34,53^. Planting intensity (P1: rubber, P2: oil palm) was quantified as the number of planted trees hectare^−1^. Data for numbers of plant taxa and individuals were obtained from Rembold *et al*.^37^.

The package “indicspecies” in R was used to identify fungal genera, which are significantly associated with different land use systems^54^. These fungal genera are defined as “indicator species” for a given land use system. The *point biserial correlation coefficient* was calculated for all identified genera and all taxa with significant (p ≤ 0.05) associations were visualized in the network. Networks were generated using the land use systems as source nodes and the associated fungal taxa as nodes, with edges corresponding to positive associations of particular taxa with specific land use systems. Networks were generated using the *edge-weighted spring-embedded layout algorithm* in Cytoscape 3.5.1 with edges weighted according to the association strength^55^. The network permits visual inspection of the strength of the connection of a fungal taxon (thickness of edges) with and its abundance (size of nodes) in a given land use type. Fungal taxa, which occur across different land use types, are connected by edges, illustrating the extent of overlap. Detailed information how to visually interpret biological data using networks is given by Merico *et al*.^56^.

### Accession code

The raw ITS rDNA sequences have been deposited in the National Center for Biotechnology Information (NCBI) Sequence Read Archive (SRA) under study accession number SRP134264 (Bioproject number PRJNA437389).

## Results

### Changes in land use shift soil fungal communities

Soil fungi were represented by 293240 high-quality sequences obtained after 454 sequencing and quality filtering. After the removal of singletons and rarefying, 4553 operational taxonomic units (OTUs) at 97% similarity represented soil fungi in this study (Supplementary Data S2a,S2b). Based on the quality-filtered sequences, the observed fungal OTU richness, calculated richness (Michaelis-Menten Fit) and Shannon diversity were highest in the jungle rubber and lowest in the rain forest (Table 1). Fungal OTU richness was driven neither by LUI nor by any other of the tested potential explanatory variables (Supplementary Data S3). Chao1 and the Simpson index showed no differences among the land use systems (Table 1).

**Table 1:** OTU richness and diversity indices of fungal taxa in rain forests and jungle rubber, monospecific rubber and oil palm plantations.

Land use system	Observed OTU richness	Michaelis-Menten Fit	Chao1	Shannon	Simpson
Rain forest	344 ± 90a	581 ± 192a	539 ± 194a	7.20 ± 0.79a	0.980 ± 0.015a
Jungle Rubber	441 ± 18b	804 ± 40b	702 ± 57a	7.93 ± 0.14b	0.992 ± 0.002a
Rubber	394 ± 45ab	698 ± 118ab	621 ± 101a	7.59 ± 0.24a	0.988 ± 0.003a
Oil palm	375 ± 46ab	664 ± 135ab	590 ± 123a	7.44 ± 0.35ab	0.985 ± 0.008a

Rarefied samples (1229 sequences) were used for the analyses. Significant differences between means of groups at p ≤ 0.05 are indicated by different letters (n = 30). OTU richness = calculation for observed species at a sequence depth of 1229 sequence reads. To test for significant differences between land use systems, linear mixed effect models and post hoc (Tukey’s) tests were applied, and differences at p ≤ 0.05 are indicated by different letters in columns. Michaelis-Menten fit and Chao1 were used to estimate the maximum species richness.

**Figure 1:**
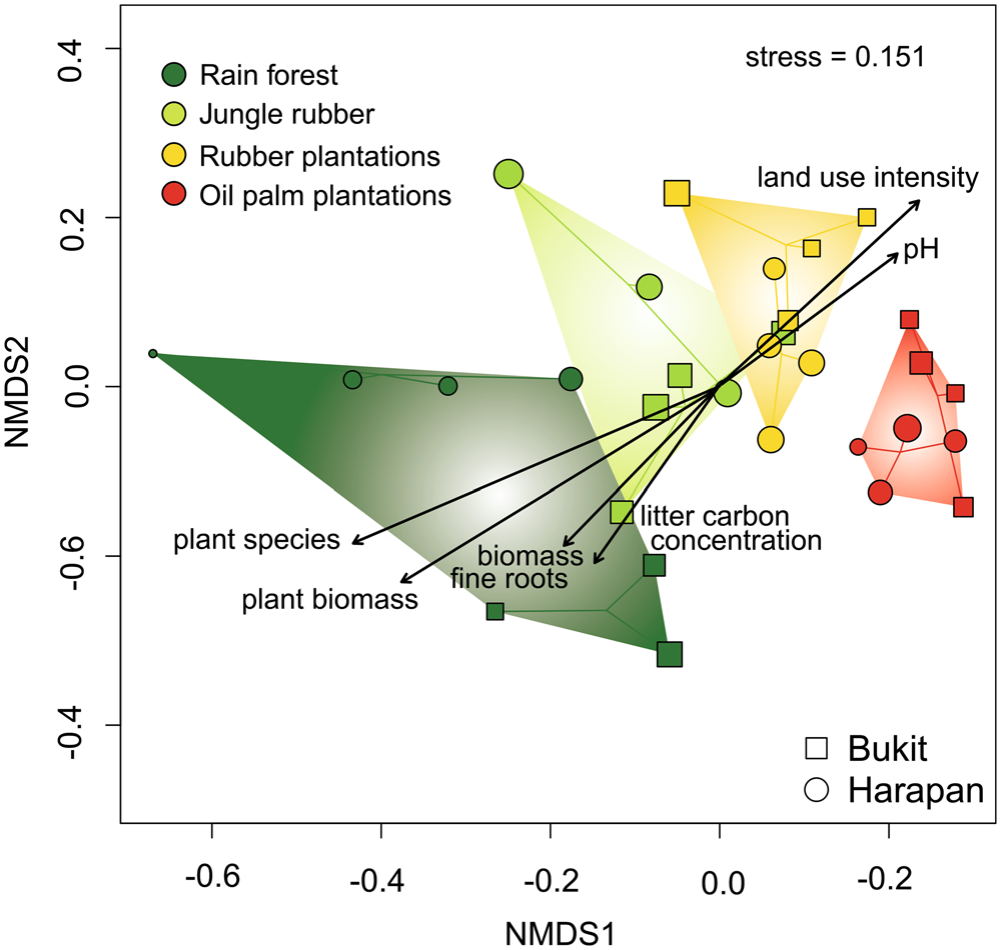
Nonmetric multidimensional scaling (NMDS) of fungal OTU communities. Three samples per plot were rarified to 1229 sequences and pooled. Significant correlations of biotic and abiotic variables with fungal communities are shown by black arrows (p ≤ 0.05). Sizes of symbols (squares and circles) correspond to the number of OTUs found in each plot, with a minimum of 172 OTUs and a maximum of 468 OTUs. Data for plant species and plant biomass were taken from Drescher *et al*., 2015.

Land use (goodness of fit R^2^ = 0.8191, p = 0.001, using landscape as an additional factor; or goodness of fit R^2^ = 0.6503, p = 0.001, ignoring landscape) had a significant influence on soil fungal community composition, while landscape alone had no significant influence (goodness of fit R^2^ = 0.0555, p = 0.236). Nonmetric multidimensional scaling (NMDS, Fig. 1) clearly separated fungal communities according to taxonomic dissimilarities. We detected four fungal clusters distributed along a gradient from unmanaged rain forest and less managed jungle rubber soils to highly managed soils of oil palm and rubber plantations (nonmetric fit, R^2^ = 0.977; linear fit, R^2^ = 0.909). To elucidate the drivers of the gradient in fungal dissimilarities, we fitted sixteen environmental variables (Supplementary Data S3), of which six were significantly related to fungal dissimilarities: land use intensity (R^2^ = 0.4707, p = 0.001), plant biomass (R^2^ = 0.8181, p = 0.001), plant species richness (R^2^ = 0.8218, p = 0.001), biomass of fine roots (R^2^ = 0.4014, p = 0.003), litter carbon concentration (R^2^ = 0.3978, p = 0.006), and soil pH (R^2^ = 0.2968, p = 0.012) (Fig. 1).

### Fungal trophic modes significantly differ among land use systems

**Figure 2:**
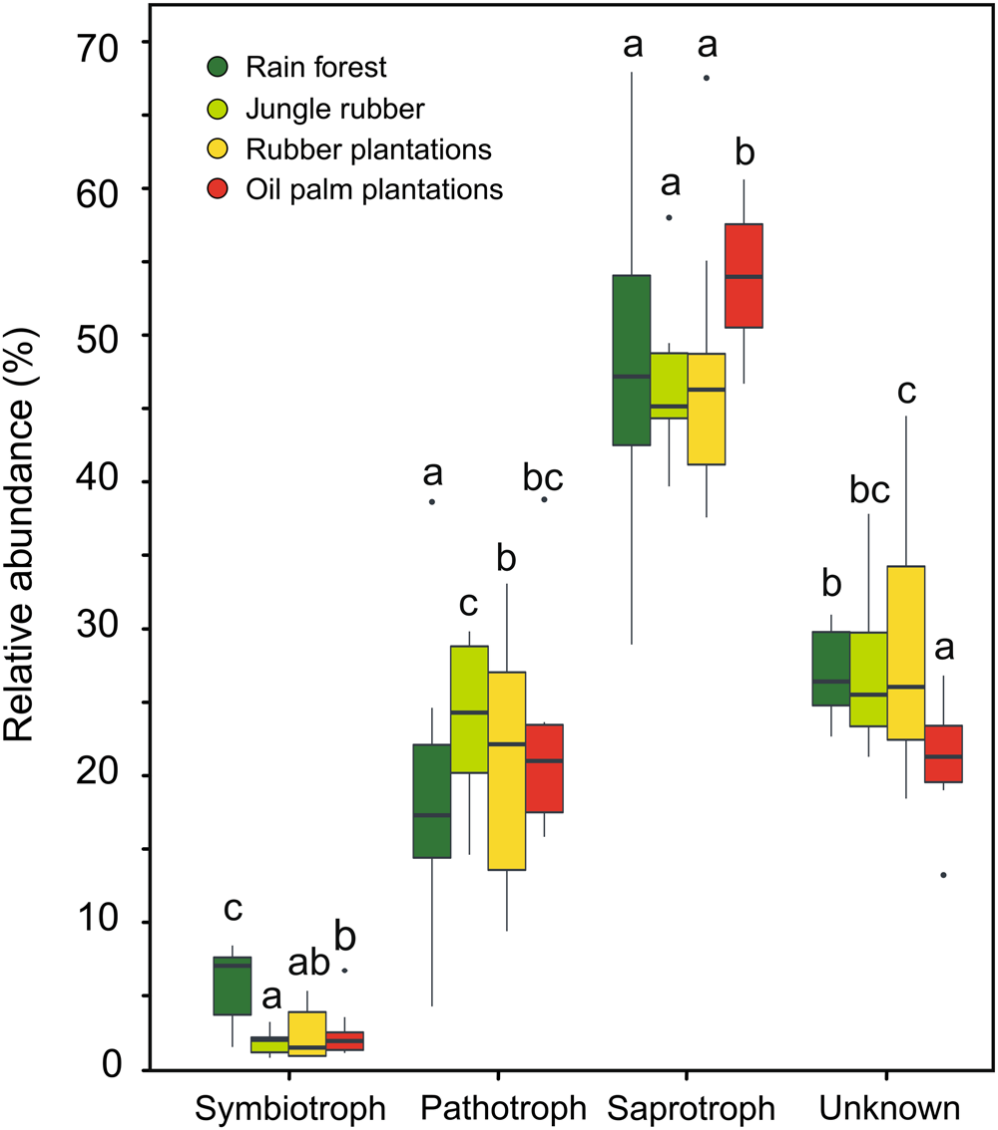
Relative abundance of symbiotrophic, pathotrophic, saprotrophic and unknown fungi in four land use systems. Box-and-whisker plots indicate the range of the data; the horizontal lines, the median; and the dots, outliers. Generalized linear mixed effect models were fit, and post hoc (Tukey’s) tests revealed significant differences at p ≤ 0.05. Significant differences are indicated by different letters (p ≤ 0.05, n = 30).

FUNGuild classified approximately 60% of the fungal OTUs according to their trophic mode. The majority of classified fungi were assigned to the group of saprotrophs (70%); 24%, to pathotrophs; and 6%, to symbiotrophs. Trophic modes differed among the land use systems: symbiotrophic fungi (p ≤ 2.2e-16) showed a maximum relative abundance in rain forests and a minimum in rubber plantations, pathotrophic fungi (p = 8.214e-14) showed the highest abundance in jungle rubber and lowest in the rain forest, and saprotrophs (p ≤ 2.2e-16) exhibited the highest abundance in oil palm plantations and lowest in the jungle rubber (Fig. 2).

### Different land use systems are characterized by shifts in fungal taxonomy

**Figure 3:**
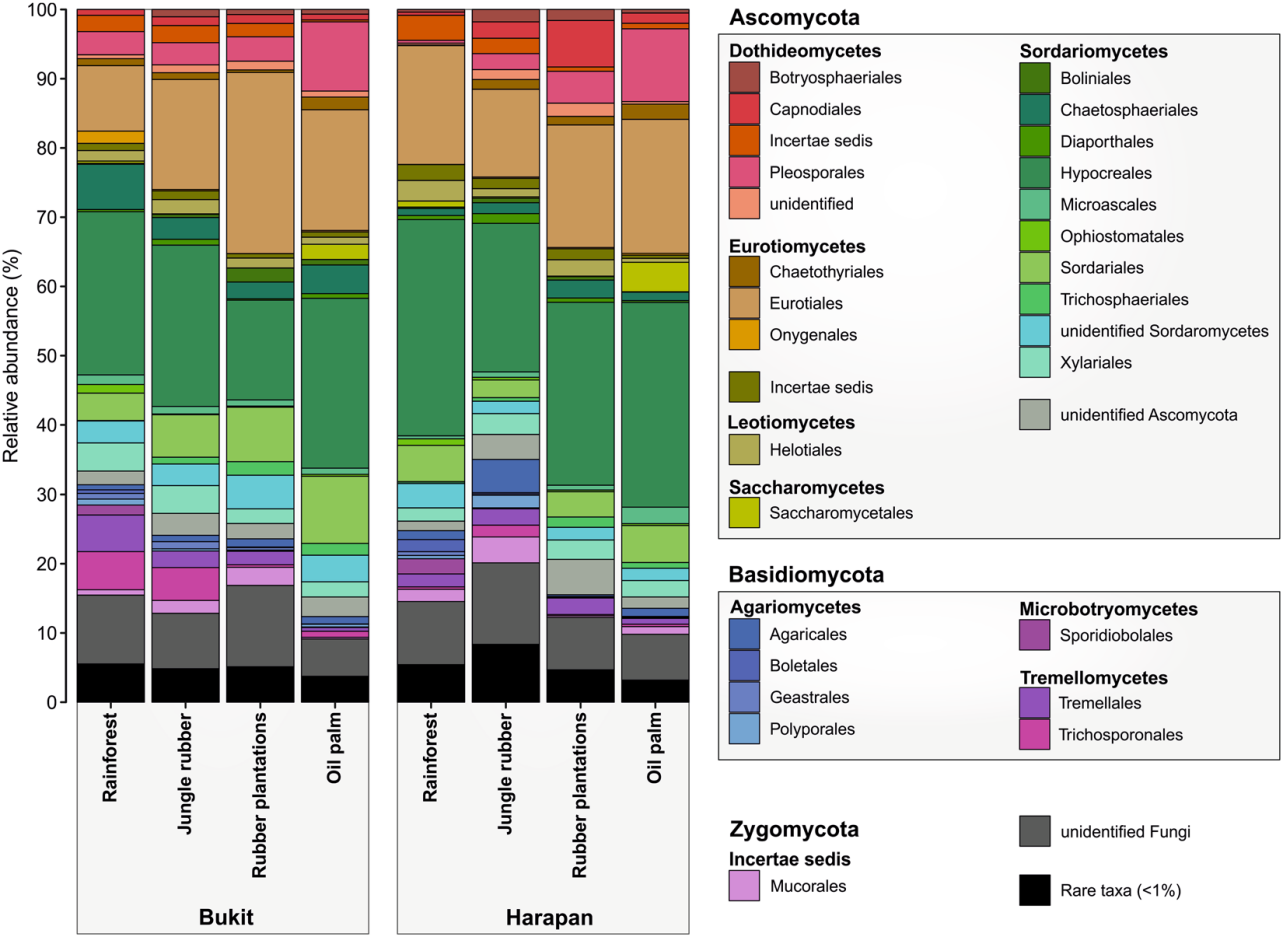
Fungal community composition on order level based on relative abundances separated by landscape and land use system. Orders with lower abundance than 1% in any land use system were summarized as artificial group “Rare taxa”.

Five fungal phyla were detected across all samples. The phylum Ascomycota showed the highest relative abundance across all land use systems, with a maximum in oil palm plantations and a minimum in jungle rubber plantations (p < 2.2e-16) (Table 2). This phylum harbored most of the abundant fungal taxa and showed increases in abundance in the order of Pleosporales and Sordariales and decreases in the order of Eurotiales (Supplementary Data S4, Fig. 3). The phylum Basidiomycota had the second highest relative abundance across all land use systems, and their abundances differed among all land use systems (p = 3.96e-4), with a minimum in oil palm plantations and a maximum in rain forests (Table 2). Decreases were prevalent in the orders of Tremellales and Trichosporonales in oil palm plantations compared with rain forest (Fig. 3). Furthermore, the abundance of Agaricales, which harbor many ectomycorrhizal fungi, also declined between rain forest and oil palm plantations (Fig. 3). The Glomeromycota (p = 0.0072) were significantly enriched in jungle rubber systems compared to other land use systems (Table 2). Chytridiomycota and Zygomycota were scarce (Table 2).

**Table 2:** Comparison of relative abundances (%) of fungal phyla.

Phylum	Rain forest	Jungle rubber	Rubber plantation	Oil palm plantation
Ascomycota	74.5 ± 5.2a	73.9 ± 12.9a	83.3 ± 8.7b	88.8 ± 1.9c
Basidiomycota	14.0 ± 5.2d	12.5 ± 7.2c	5.0 ± 2.4b	4.2 ± 1.2a
Glomeromycota	0.4 ± 0.5a	0.7 ± 0.8b	0.3 ± 0.2a	0.2 ± 0.2a
Chytridiomycota	0.2 ± 0.2a	0.2 ± 0.2a	0.3 ± 0.2a	0.2 ± 0.2a
Zygomycota	1.5 ± 0.8b	3.0 ± 3.4c	1.4 ± 2.0b	0.7 ± 0.6a
Unidentified fungi	9.5 ± 1.7b	9.6 ± 3.9b	9.7 ± 5.6b	6.0 ± 1.7a

The number of sequence reads of a taxonomic group was expressed as a proportion of the total number of sequence reads (1229) of each plot. For statistical analyses, generalized linear mixed effect models with landscape as a random effect were performed. Significant differences at p ≤ 0.05 between means of groups are indicated by letters (n = 30).

### Different land use systems harbor distinct indicator fungi

**Figure 4:**
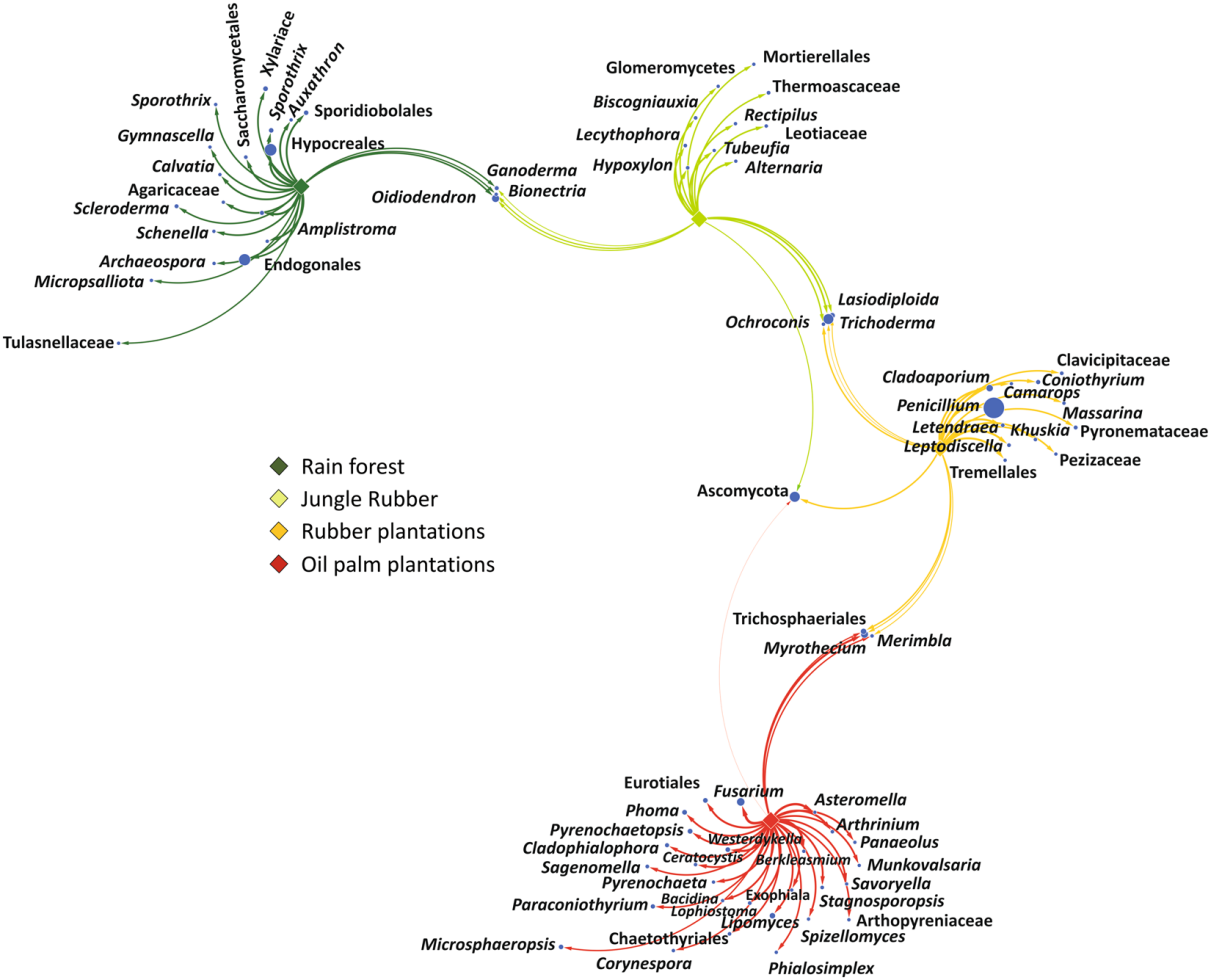
Association network of significantly abundant soil fungi in different land use systems (multipatt function in indicspecies package in R, de Cáceres *et al*., 2010). Node sizes represent the average relative abundance of OTUs in the data sets. Edges represent the association patterns of individual OTUs with the land use systems, and their lengths show the weight of the associations (edge-weighted, spring-embedded layout). The association strength of significant genera is indicated by different edge lengths varying between 0.09 and 0.79.

An association network analysis demonstrated that, of the 590 fungal genera detected in this study, 74 exhibited characteristic associations (p < 0.05) with distinct land use systems (Fig. 4, Supplementary Data S5). In accordance with other studies using this approach, the identified fungal genera were defined as indicator species^57,58^. Among those genera, only 13% were associated with two or three land use systems, and not one was shared between rain forests and the managed monospecies plantations (Supplementary Data S5). Rain forests were characterized by members of the order Hypocreales (p = 0.002) and the family Cordycipitaceae (p = 0.003). Jungle rubber and rubber plantations were characterized by an enhanced abundance of members of the genus *Trichoderma* (p = 0.02). Rubber plantations were distinguished by an increased abundance of members of the genus *Penicillium* (p = 0.003), and oil palm plantations, by an enrichment of members of the genus *Fusarium* (p = 0.001).

## Discussion

In contrast to our initial hypothesis, rain forest transformation into intensely managed oil palm and rubber monocultures did not result in a drastic loss of soil fungal species. Only marginal differences between land use systems were detected. Here, jungle rubber agroforests, representing moderately disturbed secondary rain forests due to the introduction of rubber trees, exhibited the highest OTU richness. This finding is in agreement with the intermediate disturbance hypothesis, which proposes that the highest diversities will occur in ecological systems with moderate disturbances^59^. Furthermore, earlier studies^60^ found that the same jungle rubber plots studied here also contained the highest amounts of fungal-derived fatty acids thereby supporting enhanced fungal abundance in those systems. Despite drastic decreases in plant species richness and biomass on our study sites^4,30^, bacterial diversity was not significantly decreased in oil palm and rubber plantations compared to rain forests^60,61^. However, the composition of bacterial communities was changed in response to land use changes^60^. For example, nitrogen-fixing Burkholderia (Betaproteobacteria) species decreased while ammonia-oxidizing bacteria increased in plantations, most likely due to the use of fertilizers^60^. Furthermore, members of the Bacteroidetes, i.e., uncultured Chitinophagaceae, known as chitin degraders, were also slightly more abundant in managed systems than in rain forests^60^. Here, we found that the taxonomic structures of fungal communities were also massively affected by land use systems. Compositional shifts in fungal communities with a decrease of Basidiomycota and an increase of Ascomycota in agricultural transformation systems in comparison to rain forests were also detected in other studies^19–21^. In our study, the decline in Basidiomycota was underpinned by decreased abundances of Tremellales and Agaricales on the one hand and increases of Pleosporales and Sordariales in the phylum of Ascomycota. Apparently, human interference created new habitats^62^ that were colonized by fungal communities divergent from those in rain forest soil. The main drivers of these changes were the loss of plant diversity, plant biomass, increasing soil pH and increasing land use intensity. Notably, other factors tested here such as soil nutrient availability and the enrichment of potentially toxic elements in roots such as aluminum^63^ or iron^33^ had no effect on soil fungal community composition. At variance with our findings, fungal richness declined in tropical forests in Panama, probably as the result of long-term N fertilization^64^. Herbicide application also caused significant decreases in root colonization and spore biomass of arbuscular mycorrhizal fungi in tropical agriculture^65^. Here, the managerial practices in oil palm and rubber plantations did not result in decreased mycorrhizal spore densities but resulted in lower mycorrhizal colonization and enhanced root mortality in oil palm plantations compared to other systems^33^. High spore abundances may indicate stress responses because of unsuitable environmental conditions for mycorrhizal host colonization^33^.

A striking result, which is in contrast to the occurrence of mycorrhizal species in temperate and boreal forest soils^66–68^, was an apparently low richness and abundance of mycorrhizal fungi. In temperate forests approximately 30 to 40% of the OTUs belong to mycorrhizal fungi^66,69^, whereas here only 6% were assigned to mycorrhizas. Land transformation further resulted in decreased species richness of symbiotrophic fungi in soil and, thus, obviously depleted the reservoir from which the vegetation is being colonized^70^. Because carbon, nitrogen, sulfur, manganese, and base cation concentrations showed a decline in the roots of rubber and oil palm trees compared to those from rain forests^33^, our results suggest that the impoverishment of mutualistic fungi has acute consequences for ecological functions such as plant nutrient provisioning. These findings further pinpoint the trade-off between multiple ecosystem functions and services (climate regulation, carbon storage, habitat loss) and the production of marketable goods by land transformation^71^.

Different tropical land use systems were clearly characterized by the presence of distinct fungal assemblages, in which significantly enriched taxa were denoted as indicator species. The concept of indicator species is useful to predict biodiversity-based ecosystem services, which is essential for sustainable agriculture^72^. An increase in pathogens, as observed here in managed systems, is a well-known phenomenon in monocultures composed of genetically uniform host species planted at high densities^73^. In oil palm plantations, we identified *Fusarium oxysporum* as the most abundant pathogen, supporting the idea that oil palm monoculture management fosters the proliferation of these species^74^. Fungi of the genus *Fusarium* have been described as the most destructive cause of oil palm diseases such as common spear rot in oil palms in Sumatra^75^, crown diseases^76^ and vascular wilt^77^, but in our study, no obvious disease symptoms were observed on the trees (Edy Nur, personal observation). An explanation could be the presence of *Paraconiothyrium variabile*, an antagonist of *Fusarium oxysporum*^78–80^. An additional common pest in oil palm plantations is *Ganoderma* sp., which is able to cause devastating diseases leading to great economic losses^81,82^. *Ganoderma* sp. are abundant in tropical rain forests^83,84^. Here, they were enriched in rain forests and jungle rubber systems but not in the intensively managed plantations.

Although monocropping systems such as oil palm plantations show decreased leaf litter input^33,85^, saprotrophic fungi increased. Nutrient input by fertilization, especially manure application^33^, may have created new ecological habitats, possibly fostering increased abundance of saprotrophic fungi^86^. Furthermore, the introduction of new species such as rubber can also foster new fungal associates. For example, in rubber plantations, saprotrophic species of *Leptodiscella* were enriched, for which a function in degradation of rubber litter has been described^87^. Saprotrophic *Trichoderma* species were also abundant. They are known to prevent rubber trees from fungal pathogen attack and have been successfully used as pest control organisms against *Rigidoporus microporus* and *Ganoderma pseudoferreum*, two of the most important pests of rubber trees, causing white and red root rot^88^. Furthermore, *Penicillium* sp. were identified as indicator taxa in the soil of rubber plantations. *Penicillium* species are antagonists of plant pathogens, inducing resistance^89^, for example, by the production of antibiotic compounds^90^ or establishment of mycoparasitic interactions^91^. Antagonistic relationships between beneficial *Penicillium* species and pathogenic *Fusarium* species have been demonstrated in numerous studies, including in oil palms^92–94^. *Penicillium* species are also present as endophytes in the foliage and sapwood of rubber trees^95^ and, thereby, can contribute to limiting pathogen damage in tropical trees. Our fungal indicator network for rubber and oil palm plantations was linked to saprotrophic *Myrothecium*. Endophytic *Myrothecium* species, isolated from rubber, exhibit inhibitory activity against South American Leaf Blight, a disease responsible for the poor development of rubber plantations in Latin America^96^. Overall, these findings demonstrate that land transformation triggered shifts in fungal communities towards pathogens and antagonists.

In contrast to saprotrophic and pathogenic fungi, the presence of symbiotrophic fungi in soil was strongly decreased in all types of managed systems, most likely as the result of loss of host trees and establishment of nonendemic monocultures^19,20,37^. This decrease was particularly evident for ectomycorrhizal species, which were absent on roots in rubber and oil plantations^33^ and did not appear here as indicator species in plantations. In rain forests, *Scleroderma*, which forms ectomycorrhizae^97^, was identified as an indicator taxon. Members of the genus *Archaeospora*, known to form arbuscular mycorrhizae^98^, were also identified here as indicator fungi in rain forests. Notably, fungi of the genus *Oidiodendron*, which form typical ericoid mycorrhizae^99,100^, were enriched in the rain forests and jungle rubber systems. Ericoid fungi can access organic nitrogen and thereby improve plant nitrogen nutrition under nitrogen limitation^101^. We suspect that this function may be important for the higher nitrogen retention observed in rain forests compared with oil palm plantations^102^.

## Conclusions

Overall, this study demonstrates that even moderate disturbance imposed by extensive rubber cultivation in secondary rain forest resulted in changes in soil fungal community structure compared to unmanaged forests in protected areas. The most drastic changes occurred in oil palm plantations. It is clear that our analysis is limited to some extent because the functional interactions of most soil fungi *in situ* are still unknown and present classifications of fungal guilds are far from complete. Nevertheless, our network analysis and the identified indicator taxa reveal that land transformation causes functional shifts in fungal assemblages, which put the health of these systems at risk by promoting pathogenic fungi. We found corresponding changes in the abundance of antagonistic fungi, which may point towards a control of pathogenic fungi across all land use types. Here we focused on soil inhabiting fungi. It is likely that biodiversity of plant species will also result in changes of rhizosphere and root biota, which represent primary interaction sites for microbiome members^103–105^. In future studies, it will important to analyze the extent of microbial turnover in different niches. For sustainable land use, future studies should elucidate the factors that drive the system across the tipping point and should develop countermeasures.

Because high rates of ecosystem disturbance are known to lead to extinction of all but the most disturbance-adapted species^59^, we had expected that land transformation would result in a drastic loss of fungal species richness. However, a general loss of fungal taxa was not observed, despite the massive biodiversity loss in plantations^4,106^. Instead, a massive reduction in symbiotrophic fungal species occurred, implying a loss of ecosystem provisioning functions. For example, in our oil palm plantations nitrogen losses in the run-off were higher than in the rain forests^35^. As the consequence, nutrient losses have to be compensated by fertilization^107^. Our study thus demonstrates critical links between biodiversity and ecosystem services. Increased knowledge of the impact of land use systems on fungal biodiversity is needed to use the existing agricultural land more efficiently and to balance ecological and economic goals.

## Supplementary information

Supplementary data S1

Supplementary data S2a, S2b

Supplementary data S3

Supplementary data S4

Supplementary data S5
