# Supplementary material for: Intensive tropical land use massively shifts soil fungal communities
Source: Sci Rep. 2019 Mar 4;9:3403. doi: 10.1038/s41598-019-39829-4 (PMC6399230; doi:10.1038/s41598-019-39829-4)
Supplement: Supplementary file 1 — Supplementary data S1 [file 41598_2019_39829_MOESM1_ESM.pdf]

Intensive tropical land use massively shifts soil fungal communities

Nicole Brinkmann[1,#,*], Dominik Schneider[2,#], Josephine Sahner[1], Johannes Ballauff[1], Nur Edy[1,3], Henry Barus[3], Bambang Irawan[4], Sri Wilarso Budi[5], Matin Qaim[6], Rolf Daniel[2], Andrea Polle[1]

[1]Forest Botany and Tree Physiology, University of Goettingen, Germany, [2]Genomic and Applied Microbiology and Göttingen Genomics Laboratory, University of Goettingen, Germany, [3]Department of Agrotechnology, Faculty of Agriculture, Tadulako University, Indonesia, [4]Department of Forestry, University of Jambi, Indonesia, [5]Department of Silviculture, Faculty of Forestry, Bogor Agriculture University, Bogor, Indonesia, [6]Department of Agricultural Economics and Rural Development, University of Goettingen, Germany

[#]These authors contributed equally to this work

*Correspondence: Nicole Brinkmann, Forest Botany and Tree Physiology, University of Goettingen, Büsgenweg 2, 37077 Goettingen, Germany, nbrinkm3@gwdg.de, Tel.: +49 551 39 9745, Fax: +49 551 39 22705

The authors declare no conflict of interest

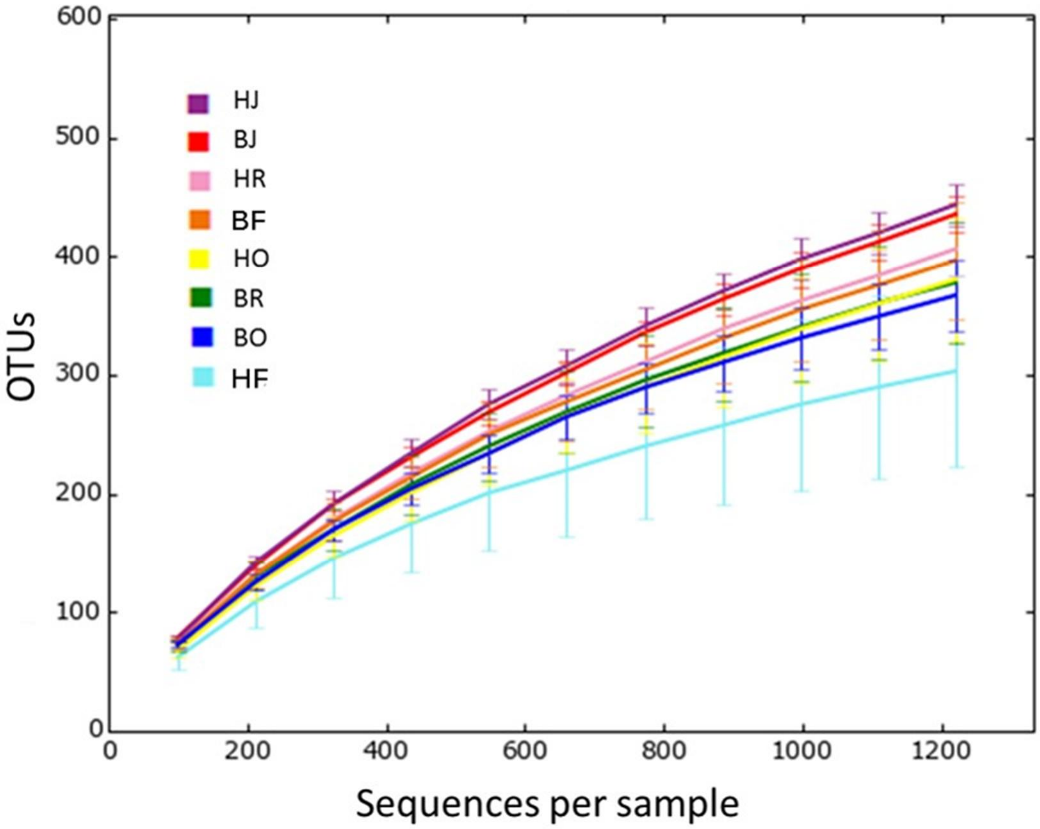

Supplementary data S1. Rarefaction curves in four land use types in two landscapes. Rarefaction curves show the average number of sequence reads per land use system and landscape up to 1229 sequences. This was the lowest number of sequences found in a sample. B = Bukit 12 landscape, H = Harapan landscape, F = rain forest, J = jungle rubber, R = rubber plantations, and O = oil palm plantations. N = 30 (±SD).
