# Supplementary material for: Intensive tropical land use massively shifts soil fungal communities
Source: Sci Rep. 2019 Mar 4;9:3403. doi: 10.1038/s41598-019-39829-4 (PMC6399230; doi:10.1038/s41598-019-39829-4)
Supplement: Supplementary file 2 — Supplementary data S2a, S2b [file 41598_2019_39829_MOESM2_ESM.pdf]

Intensive tropical land use massively shifts soil fungal communities

Nicole Brinkmann[1,#,*], Dominik Schneider[2,#], Josephine Sahner[1], Johannes Ballauff[1], Nur Edy[1,3], Henry Barus[3], Bambang Irawan[4], Sri Wilarso Budi[5], Matin Qaim[6], Rolf Daniel[2], Andrea Polle[1]

[1]Forest Botany and Tree Physiology, University of Goettingen, Germany, [2]Genomic and Applied Microbiology and Göttingen Genomics Laboratory, University of Goettingen, Germany, [3]Department of Agrotechnology, Faculty of Agriculture, Tadulako University, Indonesia, [4]Department of Forestry, University of Jambi, Indonesia, [5]Department of Silviculture, Faculty of Forestry, Bogor Agriculture University, Bogor, Indonesia, [6]Department of Agricultural Economics and Rural Development, University of Goettingen, Germany

[#]These authors contributed equally to this work

*Correspondence: Nicole Brinkmann, Forest Botany and Tree Physiology, University of Goettingen, Büsgenweg 2, 37077 Goettingen, Germany, nbrinkm3@gwdg.de, Tel.: +49 551 39 9745, Fax: +49 551 39 22705

The authors declare no conflict of interest

Supplementary data S2a. Observed numbers of fungal OTUs on sample level and sequence reads

B = Bukit 12 landscape, H = Harapan landscape, F = rain forest, J = jungle rubber, R = rubber plantations, O = oil palm plantations, 1 - 4 = number of core plot, a - c = subplot names, n.d. = not detected.

Landscapes			Land use systems			Core plots			Subplots		
Landscapes	Fungal OTU number	Fungal OTU number after rarifying	Land use systems	Fungal OTU number	Fungal OTU number after rarifying	Core plot	Fungal OTU number	Fungal OTU number after rarifying	Subplots	Sequence number after taxonomic filtering	Fungal OTU number
Bukit	5701	3005	Forest	2204	958	BF1	1207	407	BF1a	2739	390
									BF1b	5354	550
									BF1c	6906	648
						BF2	1335	454	BF2a	3731	443
									BF2b	640	196
									BF2c	8661	1025
						BF3	362	333	BF3a	983	260
									BF3b	487	178
									BF3c	51	18
						BF4	159	nd	BF4a	283	109
									BF4b	161	73
									BF4c	2	2
			Jungle Rubber	2782	1249	BJ1	1330	430	BJ1a	3871	624
									BJ2b	4606	620
									BJ3c	5556	550
						BJ2	1236	462	BJ2a	3618	528
									BJ2b	4043	607
									BJ2c	4291	510
						BJ3	1091	436	BJ3a	2844	410
									BJ3b	3197	429
									BJ3c	2737	617
						BJ4	784	420	BJ4a	2118	444
									BJ4b	1310	297
									BJ4c	2152	369
			Rubber	2702	1094	BR1	1175	340	BR1a	4397	594
									BR1b	3893	393
									BR1c	9233	559
						BR2	1129	384	BR2a	4110	468
									BR2b	6501	772
									BR2c	3052	362
						BR3	829	332	BR3a	2782	296
									BR3b	4541	452
									BR3c	4273	483
						BR4	1358	461	BR4a	2441	342
									BR4b	13680	1030
									BR4c	2309	348
			Oil palm	2041	1008	BO1	960	373	BO1a	3968	479
									BO1b	2731	394
									BO1c	3131	485
						BO2	929	416	BO2a	3558	430
									BO2b	2678	484
									BO2c	2958	432
						BO3	585	338	BO3a	1725	301
									BO3b	1672	300
									BO3c	1140	234
						BO4	863	348	BO4a	641	170
									BO4b	4874	508
									BO4c	3960	487
Harapan	4928	2794	Forest	1227	857	HF1	600	393	HF1a	2017	422
									HF1b	731	228
									HF1c	261	86
						HF2	476	327	HF2a	728	179
									HF2b	n.d.	n.d.
									HF2c	2224	365
						HF3	172	172	HF3a	435	106
									HF3b	794	89
									HF3c	n.d.	n.d.
						HF4	593	324	HF4a	1422	253
									HF4b	3443	366
									HF4c	1412	220
			Jungle Rubber	2427	1085	HJ1	1313	444	HJ1a	5590	735
									HJ1b	4454	617
									HJ1c	4036	632
						HJ2	163	nd	HJ2a	44	3
									HJ2b	548	155
									HJ2c	11	6
						HJ3	1056	425	HJ3a	4692	532
									HJ3b	1931	342
									HJ3c	2719	558
						HJ4	924	468	HJ4a	3130	598
									HJ4b	3002	504
									HJ4c	n.d.	n.d.
			Rubber	2200	1103	HR1	968	401	HR1a	3473	529
									HR1b	3172	515
									HR1c	2606	377
						HR2	1228	425	HR2a	4609	520
									HR2b	2441	374
									HR2c	3979	793
						HR3	1008	432	HR3a	3956	697
									HR3b	627	221
									HR3c	2246	484
						HR4	525	374	HR4a	709	277
									HR4b	854	238
									HR4c	683	204
			Oil palm	2742	1059	HO1	1656	445	HO1a	7645	840
									HO1b	7400	858
									HO1c	8148	896
						HO2	816	300	HO2a	6490	610
									HO2b	2215	294
									HO2c	1853	220
						HO3	801	380	HO3a	1	1
									HO3b	4959	721
									HO3c	935	189
						HO4	1314	403	HO4a	4480	651
									HO4b	7132	555
									HO4c	4414	592

Supplementary data S2b. OTU count table

OTU ID	HJ4	HF2	HJ1	HJ3	BR4	BR3	BR2	BR1	BJ4	BJ3	BJ2	BJ1	HR4	HR1	HO4	HR3	HR2	BF3	BF2	BF1	HO2	HO3	HO1	HF1	BO1	HF3	BO3	BO4	HF4	BO2	taxonomy (Unité database)
New.ReferenceOTU3740	0	0	0	0	0	0	0	0	0	0	0	0	1	0	0	0	0	0	0	0	0	0	0	0	0	0	0	0	0	0	Fungi; Ascomycota; Dothideomycetes; Pleosporales; Incertae_sedis; Leptosphaerulina; Leptosphaerulina_chartarum
SH130685.06FU_KF290956_reps	0	0	0	0	0	1	0	1	0	0	0	0	0	1	0	0	1	0	0	0	0	0	0	0	0	0	0	0	0	0	Fungi; Ascomycota; Dothideomycetes; Pleosporales; Incertae_sedis; Leptosphaerulina; Leptosphaerulina_chartarum
New.ReferenceOTU7220	0	0	0	0	0	0	0	1	0	0	0	0	0	0	0	0	0	0	0	0	0	0	0	0	1	0	0	0	0	0	Fungi; Ascomycota; Dothideomycetes; Pleosporales; Incertae_sedis; Leptosphaerulina; Leptosphaerulina_sp
SH130687.06FU_JQ717314_reps	0	0	0	0	1	0	0	0	0	0	1	0	0	2	0	0	0	0	0	0	0	0	0	0	1	0	0	0	0	0	Fungi; Ascomycota; Dothideomycetes; Pleosporales; Incertae_sedis; Leptosphaerulina; Leptosphaerulina_sp
New.ReferenceOTU4170	0	0	0	0	0	0	0	0	2	0	0	0	0	0	0	0	0	0	0	0	0	0	0	0	0	0	0	0	0	0	Fungi; Ascomycota; Eurotiomycetes; Chaetothyriales; Herpotrichiellaceae; Phialophora; Phialophora_clavispora
New.ReferenceOTU1134	0	0	0	0	0	0	0	0	1	0	0	0	0	0	0	0	0	0	1	1	0	0	0	0	1	0	0	0	0	0	Fungi; Ascomycota; Eurotiomycetes; Chaetothyriales; Herpotrichiellaceae; Phialophora; Phialophora_geniculata
New.ReferenceOTU1797	0	0	0	0	0	0	0	0	0	0	0	0	0	0	0	0	0	0	1	0	0	0	0	0	0	0	0	0	0	0	Fungi; Ascomycota; Eurotiomycetes; Chaetothyriales; Herpotrichiellaceae; Phialophora; Phialophora_geniculata
New.ReferenceOTU5079	0	0	0	0	0	0	0	0	0	0	0	0	0	0	0	0	0	0	5	3	0	0	0	0	0	0	0	0	0	0	Fungi; Ascomycota; Eurotiomycetes; Chaetothyriales; Herpotrichiellaceae; Phialophora; Phialophora_geniculata
New.ReferenceOTU5932	0	0	0	0	0	0	0	0	1	0	0	0	0	0	0	0	0	1	3	2	0	0	0	0	0	0	0	0	0	0	Fungi; Ascomycota; Eurotiomycetes; Chaetothyriales; Herpotrichiellaceae; Phialophora; Phialophora_geniculata
SH030510.06FU_AB190395_reps_singleton	0	0	0	0	0	0	0	0	0	0	0	3	0	0	3	0	0	1	4	6	0	1	1	0	2	0	3	3	0	0	Fungi; Ascomycota; Eurotiomycetes; Chaetothyriales; Herpotrichiellaceae; Phialophora; Phialophora_geniculata
New.ReferenceOTU166	0	0	0	0	0	0	0	0	0	0	0	0	0	0	0	0	0	0	0	1	0	0	0	0	0	0	0	0	0	0	Fungi; Ascomycota; Eurotiomycetes; Chaetothyriales; Herpotrichiellaceae; Phialophora; Phialophora_livistonae
New.ReferenceOTU2452	0	0	0	0	0	0	0	0	0	0	0	0	0	0	0	0	1	0	0	0	0	0	1	0	0	0	0	0	0	4	Fungi; Ascomycota; Eurotiomycetes; Chaetothyriales; Herpotrichiellaceae; Phialophora; Phialophora_livistonae
New.ReferenceOTU5102	0	0	0	0	0	0	0	0	0	0	0	0	0	0	1	0	0	0	0	0	0	1	0	0	0	0	0	0	0	0	Fungi; Ascomycota; Eurotiomycetes; Chaetothyriales; Herpotrichiellaceae; Phialophora; Phialophora_livistonae
New.ReferenceOTU231	0	0	0	0	1	0	0	0	0	0	0	0	0	0	0	0	0	0	0	0	0	0	0	0	0	0	0	0	0	0	Fungi; Ascomycota; Eurotiomycetes; Chaetothyriales; Herpotrichiellaceae; Phialophora; Phialophora_sp_TR076
New.ReferenceOTU3596	0	0	4	2	0	0	0	0	0	0	0	0	0	0	0	0	1	0	0	0	6	0	0	4	0	6	3	0	5	0	Fungi; Ascomycota; Eurotiomycetes; Eurotiales; Elaphomycetaceae; Elaphomyces; Elaphomyces_complexmurus
New.ReferenceOTU2473	0	0	0	0	0	0	0	0	0	0	0	0	0	0	0	0	0	0	0	1	0	0	0	0	0	0	0	0	0	0	Fungi; Ascomycota; Eurotiomycetes; Eurotiales; Elaphomycetaceae; Elaphomyces; Elaphomyces_sp
New.ReferenceOTU2400	0	0	0	0	0	0	0	0	0	0	0	0	0	0	0	0	0	0	2	0	0	0	0	0	0	0	0	0	0	0	Fungi; Ascomycota; Eurotiomycetes; Verrucariales; Verrucariaceae; Catapyrenium; Catapyrenium_lachneum
New.ReferenceOTU221	0	0	0	0	0	0	0	0	0	1	0	0	0	0	0	0	0	0	0	0	0	0	0	0	0	0	0	0	0	0	Fungi; Ascomycota; Eurotiomycetes; Verrucariales; Verrucariaceae; Dermatocarpon; Dermatocarpon_moulinsii
New.ReferenceOTU5898	0	0	0	0	0	0	0	0	0	0	0	0	0	0	0	0	0	0	0	0	0	0	0	0	0	0	0	0	3	0	Fungi; Ascomycota; Eurotiomycetes; Verrucariales; Verrucariaceae; Dermatocarpon; Dermatocarpon_moulinsii
New.ReferenceOTU7216	0	0	0	0	0	0	0	0	0	0	0	0	0	0	0	0	0	0	0	0	1	0	0	0	0	0	0	0	0	0	Fungi; Ascomycota; Lecanoromycetes; Agyriales; Agyriaceae; Trapeliopsis; Trapeliopsis_glaucolepidea
New.ReferenceOTU1270	0	0	0	0	0	1	0	0	0	0	0	0	0	0	0	0	0	0	0	0	0	0	0	0	0	0	0	0	3	0	Fungi; Ascomycota; Lecanoromycetes; Agyriales; Agyriaceae; Trapeliopsis; Trapeliopsis_steppicus
New.ReferenceOTU2506	0	0	0	0	0	1	0	0	0	0	0	0	0	0	0	0	0	0	0	1	0	0	0	0	2	0	0	0	0	0	Fungi; Ascomycota; Lecanoromycetes; Agyriales; Agyriaceae; Trapeliopsis; Trapeliopsis_steppicus
New.ReferenceOTU2770	0	0	0	0	0	0	0	0	0	0	0	0	0	0	0	0	0	0	0	0	0	0	0	0	0	5	0	0	0	0	Fungi; Ascomycota; Lecanoromycetes; Agyriales; Agyriaceae; Trapeliopsis; Trapeliopsis_steppicus
New.ReferenceOTU131	0	0	0	0	0	0	0	0	0	0	0	0	0	0	0	0	0	0	0	0	0	0	0	0	0	0	0	1	0	0	Fungi; Ascomycota; Lecanoromycetes; Lecanorales; Ectolechiaceae; Lasioloma; Lasioloma_arachnoideum
New.ReferenceOTU7811	0	0	0	0	0	0	0	0	0	0	0	0	0	0	0	0	0	0	0	0	0	0	0	1	0	0	0	0	1	0	Fungi; Ascomycota; Lecanoromycetes; Lecanorales; Incertae_sedis; Leprocaulon; Leprocaulon_americanum
New.ReferenceOTU7736	0	0	0	0	0	0	0	0	0	0	0	0	0	0	0	0	0	27	0	0	0	0	0	0	0	0	0	0	0	0	Fungi; Ascomycota; Lecanoromycetes; Lecanorales; Mycoblastaceae; Mycoblastus; Mycoblastus_sanguinarius
New.ReferenceOTU7221	0	0	0	0	0	0	0	0	0	0	0	0	0	0	0	0	2	0	0	0	0	0	0	0	0	0	0	0	0	0	Fungi; Ascomycota; Lecanoromycetes; Lecanorales; Parmeliaceae; Usnea; Usnea_diffracta
New.ReferenceOTU6401	0	0	0	0	0	0	0	0	0	0	0	0	0	0	1	0	0	0	0	0	5	0	0	0	0	0	0	0	0	0	Fungi; Ascomycota; Lecanoromycetes; Lecanorales; Ramalinaceae; Bacidina; Bacidina_neosquamulosa
New.ReferenceOTU2190	0	0	0	0	0	0	0	4	0	0	0	0	0	0	0	0	0	0	0	0	0	0	0	0	0	0	0	0	0	0	Fungi; Ascomycota; Lecanoromycetes; Ostropales; Stictidaceae; Absconditella; Absconditella_lignicola
New.ReferenceOTU5413	0	0	0	0	0	0	0	0	0	0	0	0	0	0	0	0	0	0	1	0	0	0	0	0	0	0	0	0	0	0	Fungi; Ascomycota; Lecanoromycetes; Ostropales; Stictidaceae; Absconditella; Absconditella_lignicola
New.ReferenceOTU7793	0	0	0	0	1	0	0	0	0	0	0	0	0	0	0	0	0	0	0	0	0	0	0	0	0	0	0	0	0	0	Fungi; Ascomycota; Lecanoromycetes; Ostropales; Stictidaceae; Absconditella; Absconditella_lignicola
New.ReferenceOTU1709	0	0	0	0	0	0	0	0	0	0	0	0	0	0	0	0	0	0	3	0	0	0	0	0	0	0	0	0	0	0	Fungi; Ascomycota; Lecanoromycetes; Peltigerales; Collemataceae; Leptogium; Leptogium_sp_BRY_C56025
New.ReferenceOTU2207	0	3	0	0	0	0	0	0	0	0	0	0	0	0	0	0	0	0	0	0	0	0	0	1	0	0	0	0	1	0	Fungi; Ascomycota; Lecanoromycetes; Pertusariales; Pertusariaceae; Pertusaria; Pertusaria_amara
New.ReferenceOTU2606	0	1	0	0	0	1	0	0	0	0	0	0	0	0	0	0	0	0	0	0	0	0	0	0	0	0	0	0	0	0	Fungi; Ascomycota; Lecanoromycetes; Pertusariales; Pertusariaceae; Pertusaria; Pertusaria_pertusa
New.ReferenceOTU7428	0	0	0	0	0	0	0	0	0	0	0	0	0	0	0	0	0	0	0	1	0	0	0	0	0	3	0	0	0	0	Fungi; Ascomycota; Lecanoromycetes; Teloschistales; Physciaceae; Heterodermia; Heterodermia_japonica
New.ReferenceOTU2616	0	0	0	1	0	0	0	0	0	0	0	0	0	0	0	0	0	0	0	0	0	0	0	0	0	0	0	0	0	0	Fungi; Ascomycota; Lecanoromycetes; Teloschistales; Teloschistaceae; Polycauliona; Polycauliona_rosei
New.ReferenceOTU3366	0	0	0	0	0	0	0	0	1	0	0	0	0	0	0	0	0	0	0	0	0	0	0	0	0	0	0	0	0	0	Fungi; Ascomycota; Lecanoromycetes; Teloschistales; Teloschistaceae; Polycauliona; Polycauliona_sp_29_UA_2013
New.ReferenceOTU6363	0	0	0	0	0	1	1	0	0	0	0	0	5	0	0	0	2	0	0	0	0	0	0	0	0	0	0	0	0	0	Fungi; Ascomycota; Lecanoromycetes; Teloschistales; Teloschistaceae; Wetmoreana; Wetmoreana_decipioides
New.ReferenceOTU3785	0	0	0	0	0	0	0	0	0	0	0	0	0	0	0	9	3	0	0	0	0	0	0	0	0	0	0	0	0	0	Fungi; Ascomycota; Lecanoromycetes; Teloschistales; Teloschistaceae; Xanthopeltis; Xanthopeltis_rupicola
New.ReferenceOTU1802	1	0	0	0	0	0	0	0	0	0	0	0	0	0	0	0	0	0	0	0	0	0	0	0	0	0	0	0	0	0	Fungi; Ascomycota; Lecanoromycetes; Umbilicariales; Umbilicariaceae; Lasallia; Lasallia_asiae_orientalis
New.ReferenceOTU7999	0	0	0	0	0	0	0	0	0	0	0	0	0	0	0	0	0	6	0	0	0	0	0	0	0	0	0	0	0	0	Fungi; Ascomycota; Lecanoromycetes; Umbilicariales; Umbilicariaceae; Umbilicaria; Umbilicaria_spodochroa
New.ReferenceOTU3961	0	0	0	1	0	0	0	0	0	0	0	0	0	0	0	0	0	0	0	0	0	0	0	0	0	0	0	0	0	0	Fungi; Ascomycota; Pezizomycetes; Pezizales; Incertae_sedis; Pulvinula; Pulvinula_sp
New.ReferenceOTU1060	0	0	1	0	0	0	0	10	0	1	3	0	9	1	1	2	1	10	17	5	0	1	0	0	3	0	17	0	0	5	Fungi; Ascomycota; Sordariomycetes; Chaetosphaeriales; Chaetosphaeriaceae; Chloridium; Chloridium_sp
New.ReferenceOTU3035	0	4	0	0	0	0	0	0	0	0	0	0	0	0	1	0	0	0	5	32	0	0	0	0	0	0	0	0	0	0	Fungi; Ascomycota; Sordariomycetes; Chaetosphaeriales; Chaetosphaeriaceae; Chloridium; Chloridium_sp
New.ReferenceOTU3959	0	0	0	0	0	0	0	0	2	0	0	0	0	0	0	0	0	3	5	1	0	0	3	0	1	0	8	0	0	0	Fungi; Ascomycota; Sordariomycetes; Chaetosphaeriales; Chaetosphaeriaceae; Chloridium; Chloridium_sp
New.ReferenceOTU3994	0	0	0	0	0	0	0	0	0	0	0	0	0	0	0	0	0	0	0	1	0	0	0	0	0	0	0	0	0	0	Fungi; Ascomycota; Sordariomycetes; Chaetosphaeriales; Chaetosphaeriaceae; Chloridium; Chloridium_sp
New.ReferenceOTU4840	0	0	0	0	0	0	0	0	0	0	0	0	0	0	0	0	0	0	0	1	0	0	0	0	0	0	0	0	0	0	Fungi; Ascomycota; Sordariomycetes; Chaetosphaeriales; Chaetosphaeriaceae; Chloridium; Chloridium_sp
New.ReferenceOTU5214	0	0	0	0	0	0	0	0	0	0	1	0	0	0	0	0	0	0	0	0	0	0	0	0	0	0	0	0	0	0	Fungi; Ascomycota; Sordariomycetes; Chaetosphaeriales; Chaetosphaeriaceae; Chloridium; Chloridium_sp
New.ReferenceOTU6796	0	1	0	2	2	0	0	0	0	0	0	4	0	0	0	0	0	0	0	0	0	0	0	0	0	0	0	0	0	0	Fungi; Ascomycota; Sordariomycetes; Chaetosphaeriales; Chaetosphaeriaceae; Chloridium; Chloridium_sp
New.ReferenceOTU7064	0	0	0	0	0	0	0	0	0	0	0	1	0	0	0	0	0	0	0	0	0	0	0	0	0	0	0	0	0	0	Fungi; Ascomycota; Sordariomycetes; Chaetosphaeriales; Chaetosphaeriaceae; Chloridium; Chloridium_sp
New.ReferenceOTU8009	0	0	0	0	0	0	0	0	0	0	0	0	0	0	0	0	0	0	0	0	0	0	1	0	0	0	0	0	0	0	Fungi; Ascomycota; Sordariomycetes; Chaetosphaeriales; Chaetosphaeriaceae; Chloridium; Chloridium_sp
SH032910.06FU_FJ528714_reps_singleton	0	0	0	0	0	0	0	0	0	0	0	0	0	0	0	0	0	0	6	8	0	0	0	0	0	0	0	0	0	0	Fungi; Ascomycota; Sordariomycetes; Chaetosphaeriales; Chaetosphaeriaceae; Chloridium; Chloridium_sp
SH138847.06FU_GQ331985_reps	0	2	3	3	1	0	0	45	3	5	7	0	11	3	4	6	36	31	20	24	0	18	11	2	15	0	50	16	0	25	Fungi; Ascomycota; Sordariomycetes; Chaetosphaeriales; Chaetosphaeriaceae; Chloridium; Chloridium_sp
SH138851.06FU_JX317423_reps	0	0	0	0	0	0	0	0	0	0	0	0	0	0	0	0	0	1	0	0	0	0	0	0	0	0	1	0	0	0	Fungi; Ascomycota; Sordariomycetes; Chaetosphaeriales; Chaetosphaeriaceae; Chloridium; Chloridium_sp
SH138853.06FU_UDB004172_reps	0	0	0	0	0	0	0	0	0	0	0	0	0	0	0	0	0	1	0	0	0	0	0	0	0	0	0	0	0	0	Fungi; Ascomycota; Sordariomycetes; Chaetosphaeriales; Chaetosphaeriaceae; Chloridium; Chloridium_sp
New.ReferenceOTU5433	0	0	0	0	0	2	0	0	0	0	0	0	5	0	0	0	0	0	0	0	0	0	0	0	0	0	0	0	0	0	Fungi; Ascomycota; Sordariomycetes; Chaetosphaeriales; Chaetosphaeriaceae; Chloridium; Chloridium_sp_52_KF_2011
New.ReferenceOTU415	0	0	0	0	0	0	0	0	1	0	1	0	0	1	1	0	0	0	3	2	0	1	0	0	1	0	0	0	0	1	Fungi; Ascomycota; Sordariomycetes; Chaetosphaeriales; Chaetosphaeriaceae; Chloridium; Chloridium_sp_GHJ_3
New.ReferenceOTU1132	0	0	1	0	0	0	0	0	0	0	0	0	0	0	0	0	0	0	0	0	0	0	0	0	0	0	0	0	0	0	Fungi; Ascomycota; Sordariomycetes; Chaetosphaeriales; Chaetosphaeriaceae; Chloridium; Chloridium_virescens_var_chlamydosporum
New.ReferenceOTU2905	2	0	0	0	0	0	0	1	0	0	0	0	0	0	0	0	0	0	0	0	0	0	0	0	0	0	0	0	0	0	Fungi; Basidiomycota; Agaricomycetes; Agaricales; Amanitaceae; Amanita; Amanita_esculenta
New.ReferenceOTU3625	1	0	0	0	0	0	0	0	0	0	0	0	3	0	0	0	0	0	0	0	0	0	0	0	0	0	0	0	0	0	Fungi; Basidiomycota; Agaricomycetes; Agaricales; Amanitaceae; Amanita; Amanita_esculenta
New.ReferenceOTU6612	1	0	0	0	0	0	0	0	0	0	0	0	2	0	0	0	0	0	0	0	0	0	0	0	0	0	0	0	0	0	Fungi; Basidiomycota; Agaricomycetes; Agaricales; Amanitaceae; Amanita; Amanita_esculenta
New.ReferenceOTU2387	0	0	0	0	0	0	0	0	0	3	0	4	0	0	0	0	0	0	0	0	0	0	0	0	0	0	0	0	0	0	Fungi; Basidiomycota; Agaricomycetes; Agaricales; Amanitaceae; Amanita; Amanita_volvata
New.ReferenceOTU241	0	0	0	0	0	0	0	1	0	0	0	0	0	0	0	0	0	0	0	0	0	0	0	0	0	0	0	0	0	0	Fungi; Basidiomycota; Agaricomycetes; Agaricales; Amanitaceae; Amanita; Amanita_yuaniana
SH136925.06FU_FN429013_refs	0	0	0	0	0	0	1	0	0	0	0	0	0	0	0	0	0	0	0	0	0	0	0	0	0	0	0	0	0	0	Fungi; Basidiomycota; Agaricomycetes; Agaricales; Cortinariaceae; Cortinarius; Cortinarius_casimiri
New.ReferenceOTU1098	0	2	0	0	0	0	0	0	0	0	0	0	0	0	0	0	0	0	0	0	0	0	0	0	0	0	0	0	0	0	Fungi; Basidiomycota; Agaricomycetes; Agaricales; Cortinariaceae; Phaeocollybia; Phaeocollybia_graveolens
New.ReferenceOTU4070	2	0	0	0	0	0	0	0	0	0	0	0	0	0	0	0	0	0	0	0	0	0	0	0	0	0	0	0	0	0	Fungi; Basidiomycota; Agaricomycetes; Agaricales; Inocybaceae; Inocybe; Inocybe_umbrinella
New.ReferenceOTU31	0	0	1	0	0	0	0	0	0	0	0	0	0	0	0	0	0	0	0	0	0	0	0	0	0	0	0	0	0	0	Fungi; Basidiomycota; Agaricomycetes; Agaricales; Inocybaceae; unidentified; Inocybaceae_sp
New.ReferenceOTU3905	0	0	0	0	0	0	0	0	0	0	0	0	0	0	0	0	0	0	0	0	0	0	0	0	0	0	0	0	0	2	Fungi; Basidiomycota; Agaricomycetes; Agaricales; Inocybaceae; unidentified; Inocybaceae_sp
New.ReferenceOTU5257	0	0	0	0	0	0	0	0	0	0	0	0	0	0	0	0	0	0	0	0	0	0	0	23	0	0	0	0	0	0	Fungi; Basidiomycota; Agaricomycetes; Agaricales; Inocybaceae; unidentified; Inocybaceae_sp
New.ReferenceOTU6372	0	0	0	0	0	0	0	0	0	0	0	1	0	0	0	0	0	0	0	0	0	0	0	0	0	0	0	0	0	0	Fungi; Basidiomycota; Agaricomycetes; Atheliales; Atheliaceae; Amphinema; Amphinema_sp
New.ReferenceOTU1204	0	0	0	0	0	0	0	0	0	0	0	0	0	0	0	0	0	0	0	1	0	0	0	0	0	0	0	0	0	0	Fungi; Basidiomycota; Agaricomycetes; Boletales; Sclerodermataceae; Scleroderma; Scleroderma_areolatum
New.ReferenceOTU1274	0	0	0	0	0	0	0	0	0	0	0	0	0	0	0	0	0	0	17	0	0	0	0	0	0	0	0	0	0	0	Fungi; Basidiomycota; Agaricomycetes; Boletales; Sclerodermataceae; Scleroderma; Scleroderma_areolatum
New.ReferenceOTU328	0	0	0	0	0	0	0	0	0	0	0	0	0	0	0	0	0	0	0	1	0	0	0	0	0	0	0	0	0	0	Fungi; Basidiomycota; Agaricomycetes; Boletales; Sclerodermataceae; Scleroderma; Scleroderma_areolatum
New.ReferenceOTU3440	0	0	0	0	0	0	0	0	0	0	0	0	0	0	0	0	0	0	0	0	0	0	0	0	0	0	0	0	80	0	Fungi; Basidiomycota; Agaricomycetes; Boletales; Sclerodermataceae; Scleroderma; Scleroderma_areolatum
New.ReferenceOTU5634	0	0	0	0	0	0	0	1	0	0	0	0	0	0	0	0	0	0	0	0	0	0	0	0	0	0	0	0	0	0	Fungi; Basidiomycota; Agaricomycetes; Boletales; Sclerodermataceae; Scleroderma; Scleroderma_areolatum
New.ReferenceOTU7152	0	0	0	0	0	0	0	0	0	0	0	0	0	1	0	0	0	0	0	0	0	0	0	4	0	0	0	0	0	0	Fungi; Basidiomycota; Agaricomycetes; Boletales; Sclerodermataceae; Scleroderma; Scleroderma_areolatum
New.ReferenceOTU7347	0	0	0	0	1	0	0	0	0	0	0	0	0	0	0	0	0	0	0	0	0	0	0	0	0	0	0	0	0	0	Fungi; Basidiomycota; Agaricomycetes; Boletales; Sclerodermataceae; Scleroderma; Scleroderma_areolatum
New.ReferenceOTU4330	0	0	0	4	0	0	0	0	0	0	0	0	0	0	0	0	0	0	0	0	0	0	0	0	0	0	0	0	0	0	Fungi; Basidiomycota; Agaricomycetes; Boletales; Sclerodermataceae; Scleroderma; Scleroderma_sp
New.ReferenceOTU4774	0	0	0	1	0	0	0	0	0	0	0	0	0	0	0	0	0	0	0	0	0	0	0	0	0	0	0	0	0	0	Fungi; Basidiomycota; Agaricomycetes; Boletales; Sclerodermataceae; Scleroderma; Scleroderma_xanthochroum
New.ReferenceOTU7335	0	0	0	0	0	0	0	0	0	0	0	0	0	0	0	0	0	0	0	0	0	0	0	0	0	0	0	0	0	1	Fungi; Basidiomycota; Agaricomycetes; Cantharellales; Ceratobasidiaceae; Ceratobasidium; Ceratobasidium_sp_JTO017
New.ReferenceOTU3645	0	0	0	0	0	0	0	0	0	0	0	0	0	1	0	0	0	0	0	0	0	0	0	1	0	0	0	0	0	0	Fungi; Basidiomycota; Agaricomycetes; Cantharellales; Clavulinaceae; Clavulina; Clavulina_coralloides
New.CleanUp.ReferenceOTU842	0	0	0	0	0	0	0	0	1	0	0	0	0	0	0	0	0	0	0	0	0	0	0	0	0	0	0	0	0	0	Fungi; Basidiomycota; Agaricomycetes; Gomphales; Gomphaceae; Ramaria; Ramaria_formosa
New.ReferenceOTU2266	0	0	0	3	0	0	0	0	0	0	0	0	0	0	0	0	0	0	0	0	0	0	0	0	0	0	0	0	0	0	Fungi; Basidiomycota; Agaricomycetes; Gomphales; Gomphaceae; Ramaria; Ramaria_formosa
New.ReferenceOTU1142	0	0	0	0	0	0	0	0	0	0	0	0	0	0	0	0	0	0	0	0	0	0	0	0	0	0	0	0	2	0	Fungi; Basidiomycota; Agaricomycetes; Gomphales; Gomphaceae; Ramaria; Ramaria_sp
New.ReferenceOTU967	0	0	0	0	0	0	0	0	0	0	0	0	0	0	0	0	0	0	3	0	0	0	0	0	0	0	0	0	0	0	Fungi; Basidiomycota; Agaricomycetes; Gomphales; Gomphaceae; Ramaria; Ramaria_sp
New.ReferenceOTU95	0	0	0	1	0	0	0	0	0	2	0	0	7	0	0	0	0	0	0	0	0	0	0	1	0	2	0	0	0	0	Fungi; Basidiomycota; Agaricomycetes; Gomphales; Gomphaceae; Ramaria; Ramaria_sp_BZ1897
New.ReferenceOTU3075	0	0	0	0	0	0	0	0	0	0	0	0	0	0	0	0	2	0	0	0	0	0	0	0	0	0	0	0	0	0	Fungi; Basidiomycota; Agaricomycetes; Hysterangiales; Hysterangiaceae; Hysterangium; Hysterangium_sp_MCA972
New.ReferenceOTU6785	0	0	0	0	0	0	0	0	2	0	0	0	0	0	0	0	0	0	0	0	0	0	0	0	0	0	0	0	0	0	Fungi; Basidiomycota; Agaricomycetes; Hysterangiales; Hysterangiaceae; Hysterangium; Hysterangium_sp_MCA972
New.ReferenceOTU6572	0	0	0	0	0	0	0	0	0	0	0	0	0	0	0	0	0	0	0	0	0	0	0	0	0	20	0	0	0	0	Fungi; Basidiomycota; Agaricomycetes; Russulales; Albatrellaceae; Polyporoletus; Polyporoletus_sublividus
New.ReferenceOTU4685	4	0	0	0	0	0	0	0	0	0	0	0	0	0	0	0	0	0	0	0	0	0	0	0	0	0	0	0	0	0	Fungi; Basidiomycota; Agaricomycetes; Russulales; Russulaceae; unidentified; uncultured_Russulaceae
New.ReferenceOTU6817	0	0	0	0	0	0	0	0	0	0	0	0	0	0	0	0	0	0	0	0	0	0	0	0	0	0	0	0	2	0	Fungi; Basidiomycota; Agaricomycetes; Thelephorales; Thelephoraceae; Tomentella; Tomentella_sp
New.ReferenceOTU1486	0	0	0	0	0	0	0	0	0	0	0	0	1	0	0	0	0	1	0	0	0	0	0	0	0	0	0	0	0	0	Fungi; Chytridiomycota; Neocallimastigomycetes; Neocallimastigales; Neocallimastigaceae; Anaeromyces; Anaeromyces_sp_5_BRL_10
New.ReferenceOTU3876	0	0	0	0	1	0	0	0	0	0	0	0	0	0	0	0	0	0	0	0	0	0	0	0	0	0	0	0	0	0	Fungi; Glomeromycota; Glomeromycetes; Archaeosporales; Archaeosporaceae; Archaeospora; Archaeospora_trappei
New.ReferenceOTU5118	0	0	0	0	0	0	0	0	0	0	0	0	0	0	0	0	0	0	0	0	0	0	0	0	0	0	0	0	5	0	Fungi; Glomeromycota; Glomeromycetes; Archaeosporales; Archaeosporaceae; Archaeospora; Archaeospora_trappei
New.ReferenceOTU7892	0	4	0	0	0	0	0	0	0	0	0	0	0	0	0	0	0	0	0	0	0	0	0	6	0	0	0	0	0	0	Fungi; Glomeromycota; Glomeromycetes; Archaeosporales; Archaeosporaceae; Archaeospora; Archaeospora_trappei
New.ReferenceOTU4916	0	0	0	0	0	0	0	1	0	0	0	0	0	0	0	0	0	0	0	0	0	0	0	0	0	0	0	0	0	0	Fungi; Glomeromycota; Glomeromycetes; Archaeosporales; Archaeosporaceae; unidentified; Archaeosporaceae_sp

New.ReferenceOTU4776	0	0	0	0	0	0	0	0	0	0	0	1	0	0	0	0	0	0	0	0	0	0	0	0	0	0	0	0	0	0	Fungi; Glomeromycota; Glomeromycetes; Archaeosporales; Archaeosporaceae; unidentified; uncultured_Archaeospora
New.ReferenceOTU5196	1	0	0	0	0	0	0	0	0	0	0	0	0	0	0	0	0	0	0	0	0	0	0	0	0	0	0	0	0	0	Fungi; Glomeromycota; Glomeromycetes; Archaeosporales; Archaeosporaceae; unidentified; uncultured_Archaeospora
New.ReferenceOTU5670	0	0	0	0	0	0	0	0	0	0	0	0	3	0	0	0	0	0	0	0	0	0	0	0	0	0	0	1	0	0	Fungi; Glomeromycota; Glomeromycetes; Archaeosporales; Archaeosporaceae; unidentified; uncultured_Archaeospora
New.CleanUp.ReferenceOTU1900	0	0	0	0	0	0	0	0	0	0	0	0	0	0	0	1	0	0	0	0	0	0	0	0	0	0	0	0	0	0	Fungi; Glomeromycota; Glomeromycetes; Diversisporales; Acaulosporaceae; Acaulospora; Acaulospora_lacunosa
New.ReferenceOTU6460	0	0	0	0	0	0	0	0	0	0	0	5	0	0	0	0	0	0	0	0	0	0	0	0	0	0	0	0	0	0	Fungi; Glomeromycota; Glomeromycetes; Diversisporales; Acaulosporaceae; unidentified; uncultured_Acaulosporaceae
New.ReferenceOTU2287	0	0	0	0	0	0	0	0	0	0	0	0	0	0	0	0	0	0	0	0	0	0	0	11	0	0	0	0	0	0	Fungi; Glomeromycota; Glomeromycetes; Diversisporales; Gigasporaceae; Cetraspora; Cetraspora_sp
SH005320.06FU_AF004686_reps_singleton	0	0	0	0	0	0	0	0	0	0	0	0	0	0	2	0	0	0	0	0	1	0	0	0	0	0	0	0	0	0	Fungi; Glomeromycota; Glomeromycetes; Diversisporales; Gigasporaceae; Scutellospora; Scutellospora_coralloidea
New.ReferenceOTU4507	0	0	0	0	0	0	0	0	0	1	0	0	0	0	0	4	0	0	0	0	0	0	0	1	0	0	0	0	0	0	Fungi; Glomeromycota; Glomeromycetes; Glomerales; Claroideoglomeraceae; Claroideoglomus; Claroideoglomus_sp
New.ReferenceOTU4508	0	0	0	0	0	0	0	0	0	0	0	0	0	0	0	2	0	0	0	0	0	0	0	0	0	0	0	0	0	0	Fungi; Glomeromycota; Glomeromycetes; Glomerales; Claroideoglomeraceae; Claroideoglomus; Claroideoglomus_sp
New.ReferenceOTU7334	0	0	0	0	0	0	0	0	0	0	0	0	0	0	0	0	0	0	0	0	1	0	0	0	0	0	0	0	0	0	Fungi; Glomeromycota; Glomeromycetes; Glomerales; Claroideoglomeraceae; Claroideoglomus; Claroideoglomus_sp
New.ReferenceOTU7515	0	0	0	0	0	0	0	0	0	0	0	0	0	1	0	0	0	1	0	0	0	0	0	0	0	0	0	0	0	0	Fungi; Glomeromycota; Glomeromycetes; Glomerales; Glomeraceae; Glomus; Glomus_sp_9_SUN_2011
New.ReferenceOTU911	0	0	0	0	0	0	0	0	0	0	0	0	0	0	0	0	0	4	0	0	0	0	0	0	0	0	0	0	0	0	Fungi; Glomeromycota; Glomeromycetes; Glomerales; Glomeraceae; Glomus; Glomus_sp_9_SUN_2011
New.ReferenceOTU2316	1	0	0	0	0	0	0	0	0	0	0	0	0	0	0	0	0	0	0	0	0	0	0	0	0	0	0	0	0	0	Fungi; Glomeromycota; Glomeromycetes; Glomerales; Glomeraceae; unidentified; Glomeraceae_sp
New.ReferenceOTU2659	0	0	0	0	0	0	0	0	0	0	1	0	0	0	0	0	0	0	0	0	0	0	0	0	0	0	0	0	0	1	Fungi; Glomeromycota; Glomeromycetes; Glomerales; Glomeraceae; unidentified; Glomeraceae_sp
New.ReferenceOTU3113	0	0	0	0	0	0	0	0	0	0	0	0	0	0	0	0	0	0	0	0	0	0	0	0	0	0	0	0	0	1	Fungi; Glomeromycota; Glomeromycetes; Glomerales; Glomeraceae; unidentified; Glomeraceae_sp
New.ReferenceOTU3941	0	0	0	0	0	0	0	0	0	0	0	0	0	0	0	0	0	0	0	0	0	0	0	0	0	0	0	0	0	1	Fungi; Glomeromycota; Glomeromycetes; Glomerales; Glomeraceae; unidentified; Glomeraceae_sp
New.ReferenceOTU4368	2	0	0	0	0	0	0	0	0	0	0	0	0	0	0	0	0	0	0	0	0	0	0	0	0	0	0	0	0	0	Fungi; Glomeromycota; Glomeromycetes; Glomerales; Glomeraceae; unidentified; Glomeraceae_sp
New.ReferenceOTU5185	1	0	0	0	0	0	0	0	0	0	0	0	0	0	0	0	0	0	0	0	0	0	0	0	0	0	0	0	0	0	Fungi; Glomeromycota; Glomeromycetes; Glomerales; Glomeraceae; unidentified; Glomeraceae_sp
New.ReferenceOTU5560	0	0	0	0	0	0	0	0	0	0	0	0	0	0	0	0	0	0	0	0	0	0	0	0	0	0	0	0	0	1	Fungi; Glomeromycota; Glomeromycetes; Glomerales; Glomeraceae; unidentified; Glomeraceae_sp
New.ReferenceOTU5759	2	0	0	0	0	0	0	0	0	0	0	0	0	0	0	0	0	0	0	0	0	0	0	0	0	0	0	0	0	0	Fungi; Glomeromycota; Glomeromycetes; Glomerales; Glomeraceae; unidentified; Glomeraceae_sp
New.ReferenceOTU5906	0	0	0	0	0	0	0	0	0	0	0	0	0	0	0	0	0	0	0	0	0	0	0	0	0	0	0	0	0	1	Fungi; Glomeromycota; Glomeromycetes; Glomerales; Glomeraceae; unidentified; Glomeraceae_sp
New.ReferenceOTU6351	3	0	0	0	0	0	0	0	0	0	0	0	0	0	0	0	0	0	0	0	0	0	0	0	0	0	0	0	0	0	Fungi; Glomeromycota; Glomeromycetes; Glomerales; Glomeraceae; unidentified; Glomeraceae_sp
New.ReferenceOTU64	2	0	0	0	0	0	0	0	0	0	0	0	0	0	0	0	0	0	0	0	0	0	0	0	0	0	0	0	0	0	Fungi; Glomeromycota; Glomeromycetes; Glomerales; Glomeraceae; unidentified; Glomeraceae_sp
New.ReferenceOTU6683	0	0	0	1	0	0	0	0	0	0	0	0	0	0	0	0	0	0	0	0	0	0	0	0	0	0	0	0	0	0	Fungi; Glomeromycota; Glomeromycetes; Glomerales; Glomeraceae; unidentified; Glomus_arbuscular_mycorrhizal_symbiont_138_6
New.ReferenceOTU266	1	0	0	0	0	0	0	0	0	0	0	0	0	0	0	0	0	0	0	0	0	0	0	0	0	0	0	0	0	0	Fungi; Glomeromycota; Glomeromycetes; Glomerales; Glomeraceae; unidentified; uncultured_Glomus
New.ReferenceOTU3416	1	0	0	0	0	0	0	0	0	0	0	0	0	0	0	0	0	0	0	0	0	0	0	0	0	0	0	0	0	0	Fungi; Glomeromycota; Glomeromycetes; Glomerales; Glomeraceae; unidentified; uncultured_Glomus
New.ReferenceOTU4052	0	0	0	1	0	0	0	0	0	0	0	0	0	0	0	0	0	0	0	0	0	0	0	0	0	0	0	0	0	0	Fungi; Glomeromycota; Glomeromycetes; Glomerales; Glomeraceae; unidentified; uncultured_Glomus
New.ReferenceOTU478	0	0	0	0	1	3	3	1	0	1	0	8	1	1	0	0	0	0	0	0	0	0	0	0	0	0	0	1	0	0	Fungi; Glomeromycota; Glomeromycetes; Glomerales; Glomeraceae; unidentified; uncultured_Glomus
New.ReferenceOTU4848	0	0	0	0	0	0	0	0	0	0	0	0	0	1	0	0	0	0	0	0	0	0	0	0	0	0	0	0	0	0	Fungi; Glomeromycota; Glomeromycetes; Glomerales; Glomeraceae; unidentified; uncultured_Glomus
New.ReferenceOTU7072	0	0	0	3	0	0	0	0	0	0	0	0	0	0	0	0	0	0	0	0	0	0	0	0	0	0	0	0	0	0	Fungi; Glomeromycota; Glomeromycetes; Glomerales; Glomeraceae; unidentified; uncultured_Glomus
New.ReferenceOTU6502	1	0	0	0	0	0	0	0	0	0	0	0	0	0	0	0	0	0	0	0	0	0	0	0	0	0	0	0	0	0	Fungi; Glomeromycota; Glomeromycetes; Glomerales; Glomeraceae; unidentified; uncultured_Rhizophagus
New.ReferenceOTU6922	2	0	0	1	0	0	0	0	0	0	0	0	0	0	0	0	0	0	0	0	0	0	0	0	0	0	0	0	0	0	Fungi; Glomeromycota; Glomeromycetes; Glomerales; Glomeraceae; unidentified; uncultured_Rhizophagus
New.ReferenceOTU2865	0	0	0	0	0	0	0	0	0	0	0	0	0	0	0	0	0	0	0	0	0	0	0	0	0	0	0	2	0	0	Fungi; Glomeromycota; Glomeromycetes; Glomerales; unidentified; unidentified; Glomerales_sp
New.ReferenceOTU3001	0	0	0	1	0	0	0	0	0	0	0	0	0	0	0	0	0	0	0	0	0	0	0	0	0	0	0	0	0	0	Fungi; Glomeromycota; Glomeromycetes; Glomerales; unidentified; unidentified; Glomerales_sp
New.ReferenceOTU220	2	0	0	0	0	0	0	0	0	0	0	0	0	0	0	0	0	0	0	0	0	0	0	0	0	0	0	0	0	0	Fungi; Glomeromycota; Glomeromycetes; unidentified; unidentified; unidentified; Glomeromycetes_sp
New.ReferenceOTU3049	1	0	0	0	0	0	0	0	0	0	0	0	0	0	0	0	0	0	0	0	0	0	0	0	0	0	0	0	0	0	Fungi; Glomeromycota; unidentified; unidentified; unidentified; unidentified; Glomeromycota_sp
New.ReferenceOTU4019	0	0	0	0	1	0	0	0	0	0	0	0	0	0	0	0	0	0	0	0	0	0	0	0	0	0	0	0	0	0	Fungi; Glomeromycota; unidentified; unidentified; unidentified; unidentified; Glomeromycota_sp
New.ReferenceOTU4418	0	0	0	0	0	0	0	0	0	0	0	0	0	0	0	0	0	0	0	0	0	0	0	0	0	0	1	0	0	1	Fungi; Glomeromycota; unidentified; unidentified; unidentified; unidentified; Glomeromycota_sp
New.ReferenceOTU5101	0	0	0	0	0	0	0	0	2	0	0	0	0	0	0	0	0	0	0	0	0	0	0	0	0	0	0	0	0	0	Fungi; Glomeromycota; unidentified; unidentified; unidentified; unidentified; Glomeromycota_sp
New.ReferenceOTU5472	0	0	0	0	1	0	0	0	0	0	0	0	0	0	0	0	0	0	0	0	0	0	0	0	0	0	0	0	0	0	Fungi; Glomeromycota; unidentified; unidentified; unidentified; unidentified; Glomeromycota_sp
New.ReferenceOTU6852	1	0	0	0	0	0	0	0	0	0	0	0	0	0	0	0	0	0	0	0	0	0	0	0	0	0	0	0	0	0	Fungi; Glomeromycota; unidentified; unidentified; unidentified; unidentified; Glomeromycota_sp
New.ReferenceOTU1975	0	0	0	0	0	0	0	0	6	0	0	0	0	0	0	0	0	0	0	0	0	0	0	0	0	0	0	0	0	0	Fungi; Glomeromycota; unidentified; unidentified; unidentified; unidentified; uncultured_Glomeromycota
New.ReferenceOTU4295	0	0	0	0	0	0	0	0	0	0	0	0	0	0	0	0	0	0	0	0	0	2	0	0	0	0	0	0	0	0	Fungi; Glomeromycota; unidentified; unidentified; unidentified; unidentified; uncultured_Glomeromycota
New.ReferenceOTU5912	0	0	0	0	0	0	0	0	1	0	0	0	0	0	0	0	0	0	0	0	0	0	0	0	0	0	0	0	0	0	Fungi; Glomeromycota; unidentified; unidentified; unidentified; unidentified; uncultured_Glomeromycota
New.ReferenceOTU6338	3	0	0	0	0	0	0	0	0	0	0	0	0	0	0	0	0	0	0	0	0	0	0	0	0	0	0	0	0	0	Fungi; Glomeromycota; unidentified; unidentified; unidentified; unidentified; uncultured_Glomeromycota
New.ReferenceOTU6926	3	0	0	0	0	0	0	0	0	0	0	0	0	0	0	0	0	0	0	0	0	0	0	0	0	0	0	0	0	0	Fungi; Glomeromycota; unidentified; unidentified; unidentified; unidentified; uncultured_Glomeromycota
New.ReferenceOTU7748	0	0	0	0	0	0	0	0	0	0	0	1	0	0	0	0	0	0	0	0	0	0	0	0	0	0	0	0	0	0	Fungi; Glomeromycota; unidentified; unidentified; unidentified; unidentified; uncultured_Glomeromycota
New.ReferenceOTU5834	0	0	0	0	0	0	0	0	0	0	0	0	0	0	0	0	0	0	0	0	1	0	0	0	0	0	0	0	0	0	Fungi; Zygomycota; Incertae_sedis; Endogonales; Endogonaceae; Endogone; Endogone_lactiflua
New.ReferenceOTU7112	1	0	0	0	0	0	0	0	0	0	0	0	0	0	0	0	0	0	0	0	0	0	0	0	0	0	0	0	0	0	Fungi; Zygomycota; Incertae_sedis; Endogonales; Endogonaceae; Endogone; Endogone_lactiflua
New.ReferenceOTU3183	0	0	0	1	0	0	0	0	0	0	0	0	0	0	0	0	0	0	0	0	0	0	0	0	0	0	0	0	0	0	Fungi; Zygomycota; Incertae_sedis; Endogonales; Endogonaceae; Endogone; Endogone_pisiformis
New.ReferenceOTU307	0	0	0	0	0	0	0	0	0	0	0	0	0	0	0	0	0	0	0	0	0	0	0	0	0	0	0	0	2	0	Fungi; Zygomycota; Incertae_sedis; Mucorales; Lentamycetaceae; Lentamyces; Lentamyces_parricida
New.ReferenceOTU3813	0	0	0	0	0	0	0	0	0	0	0	0	0	0	0	0	0	0	0	0	0	0	0	0	0	1	0	0	0	0	Fungi; Zygomycota; Incertae_sedis; Mucorales; Lentamycetaceae; Lentamyces; Lentamyces_parricida
New.ReferenceOTU7773	0	0	0	0	0	0	0	0	0	0	0	0	0	0	0	0	0	0	0	1	0	0	0	0	0	0	0	0	0	0	Fungi; Ascomycota; Dothideomycetes; Incertae_sedis; Myxotrichaceae; Myxotrichum; Myxotrichum_sp_OUCMBI101149
New.ReferenceOTU2256	0	0	0	7	0	0	0	0	0	0	0	0	0	0	0	0	0	0	0	0	0	0	0	0	0	0	0	0	0	0	Fungi; Basidiomycota; Agaricomycetes; Agaricales; Entolomataceae; Entoloma; Entoloma_abortivum
New.ReferenceOTU3407	0	0	0	0	0	0	0	0	0	0	0	0	0	0	0	0	0	0	0	0	0	2	0	0	0	0	0	0	0	0	Fungi; Basidiomycota; Agaricomycetes; Agaricales; Entolomataceae; Entoloma; Entoloma_aprile
New.ReferenceOTU7409	0	0	1	0	0	0	0	0	0	0	0	0	0	0	0	0	0	0	0	0	0	0	0	0	0	0	0	0	0	0	Fungi; Basidiomycota; Agaricomycetes; Agaricales; Entolomataceae; Entoloma; Entoloma_bloxamii
New.ReferenceOTU3156	1	0	0	0	0	0	0	0	0	0	0	0	0	0	0	0	0	0	0	0	0	0	0	0	0	0	0	0	0	0	Fungi; Basidiomycota; Agaricomycetes; Agaricales; Entolomataceae; Entoloma; Entoloma_callidermum
SH451895.06FU_KC710115_reps_singleton	4	0	0	0	0	0	0	0	0	0	0	0	0	0	0	0	0	0	0	0	0	0	0	0	0	0	0	0	0	0	Fungi; Basidiomycota; Agaricomycetes; Agaricales; Entolomataceae; Entoloma; Entoloma_callidermum
New.ReferenceOTU3414	1	0	0	0	0	0	0	0	0	0	0	0	0	0	0	0	0	0	0	0	0	0	0	0	0	0	0	0	0	0	Fungi; Basidiomycota; Agaricomycetes; Agaricales; Entolomataceae; Entoloma; Entoloma_gelatinosum
New.ReferenceOTU7828	0	0	0	0	0	0	0	0	0	0	0	0	0	0	0	0	2	0	0	0	0	0	0	0	0	0	0	0	0	0	Fungi; Basidiomycota; Agaricomycetes; Agaricales; Entolomataceae; Entoloma; Entoloma_korhonenii
New.ReferenceOTU6868	0	0	0	0	0	0	0	0	0	0	0	0	0	0	0	0	0	0	0	0	0	0	0	0	0	0	0	0	1	0	Fungi; Basidiomycota; Agaricomycetes; Agaricales; Entolomataceae; Entoloma; Entoloma_omiense
New.ReferenceOTU6590	2	0	0	0	0	0	0	0	0	0	0	0	0	0	0	0	0	0	0	0	0	0	0	0	0	0	0	0	0	0	Fungi; Basidiomycota; Agaricomycetes; Agaricales; Entolomataceae; Entoloma; Entoloma_perumbilicatum
New.ReferenceOTU1546	1	0	0	0	0	0	0	0	0	0	0	0	0	0	1	0	0	0	0	0	0	0	0	0	0	0	0	0	0	0	Fungi; Basidiomycota; Agaricomycetes; Agaricales; Entolomataceae; Entoloma; Entoloma_platyphylloides
New.ReferenceOTU5936	0	0	0	0	0	0	0	0	0	0	0	0	0	0	0	0	2	0	0	0	0	0	0	0	0	0	0	0	0	0	Fungi; Basidiomycota; Agaricomycetes; Agaricales; Entolomataceae; Entoloma; Entoloma_platyphylloides
New.ReferenceOTU3147	0	0	0	1	0	0	0	0	0	1	0	0	0	0	0	0	0	0	0	0	1	0	1	0	0	0	0	2	2	0	Fungi; Basidiomycota; Agaricomycetes; Agaricales; Entolomataceae; Entoloma; Entoloma_rhodopolium
New.ReferenceOTU4210	0	0	0	0	0	0	0	0	0	0	0	0	0	0	0	0	0	0	0	0	0	0	0	0	0	4	0	0	0	0	Fungi; Basidiomycota; Agaricomycetes; Agaricales; Entolomataceae; Entoloma; Entoloma_sericeoides
New.ReferenceOTU43	4	0	0	0	0	0	0	0	0	0	0	0	0	0	0	0	0	0	0	0	0	0	0	0	0	0	0	0	0	0	Fungi; Basidiomycota; Agaricomycetes; Agaricales; Entolomataceae; Entoloma; Entoloma_sp
New.ReferenceOTU4377	0	0	0	0	0	0	0	0	0	0	0	0	0	0	0	0	0	0	0	2	0	0	0	0	0	0	0	0	0	0	Fungi; Basidiomycota; Agaricomycetes; Agaricales; Entolomataceae; Entoloma; Entoloma_sp
New.ReferenceOTU4881	0	0	0	0	0	0	0	0	0	0	0	0	0	0	0	0	0	0	1	0	0	0	0	0	0	0	0	0	0	0	Fungi; Basidiomycota; Agaricomycetes; Agaricales; Entolomataceae; Entoloma; Entoloma_sp
New.ReferenceOTU5526	0	0	0	0	0	0	0	0	0	0	0	0	0	0	0	0	0	0	0	1	0	0	0	0	0	0	0	0	0	0	Fungi; Basidiomycota; Agaricomycetes; Agaricales; Entolomataceae; Entoloma; Entoloma_sp
New.ReferenceOTU6032	1	0	0	0	0	0	0	0	0	0	0	0	0	0	0	0	0	0	0	0	0	0	0	0	0	0	0	0	0	0	Fungi; Basidiomycota; Agaricomycetes; Agaricales; Entolomataceae; Entoloma; Entoloma_sp
New.ReferenceOTU7603	1	0	0	0	0	0	0	0	0	0	0	0	0	0	0	0	0	0	0	0	0	0	0	0	0	0	0	0	0	0	Fungi; Basidiomycota; Agaricomycetes; Agaricales; Entolomataceae; Entoloma; Entoloma_sp
New.ReferenceOTU839	0	0	1	0	0	0	0	0	0	0	0	0	0	0	0	0	0	0	0	0	1	0	0	0	0	0	0	0	0	0	Fungi; Basidiomycota; Agaricomycetes; Agaricales; Entolomataceae; Entoloma; Entoloma_sp
New.ReferenceOTU126	0	0	0	0	0	0	0	0	0	0	0	0	0	0	1	0	0	0	0	0	0	0	0	0	0	0	0	0	0	0	Fungi; Basidiomycota; Agaricomycetes; Agaricales; Entolomataceae; Entoloma; Entoloma_sp_HMLD1538
New.ReferenceOTU1057	0	0	0	0	0	0	0	0	0	0	0	0	0	0	0	0	0	0	1	0	0	0	0	0	0	0	0	0	0	0	Fungi; Basidiomycota; Agaricomycetes; Agaricales; Entolomataceae; Entoloma; Entoloma_sp_JRW_2013
New.ReferenceOTU3508	0	0	0	0	0	0	0	0	0	0	0	0	0	0	0	0	0	0	0	1	0	0	0	0	0	0	0	0	0	0	Fungi; Basidiomycota; Agaricomycetes; Agaricales; Entolomataceae; Entoloma; Entoloma_sp_JRW_2013
New.ReferenceOTU5913	0	0	0	0	0	0	0	0	0	0	0	0	0	0	0	0	0	0	0	1	0	0	0	0	0	0	0	0	0	0	Fungi; Basidiomycota; Agaricomycetes; Agaricales; Hygrophoraceae; Hygrocybe; Hygrocybe_fornicata
New.ReferenceOTU5164	0	1	0	0	0	0	1	0	1	4	4	0	0	0	1	0	0	0	0	0	0	0	0	0	0	1	0	0	0	0	Fungi; Basidiomycota; Agaricomycetes; Agaricales; Hygrophoraceae; Hygrocybe; Hygrocybe_insipida
New.ReferenceOTU5528	0	0	0	0	0	0	0	0	3	0	0	0	0	0	0	0	0	0	0	0	0	0	0	0	0	0	0	3	0	0	Fungi; Basidiomycota; Agaricomycetes; Agaricales; Hygrophoraceae; Hygrocybe; Hygrocybe_insipida
SH025556.06FU_JF796076_reps_singleton	0	0	0	0	1	0	0	0	0	0	0	0	0	0	0	0	0	0	0	0	0	0	0	0	0	0	0	0	0	0	Fungi; Ascomycota; Dothideomycetes; Pleosporales; Pleosporaceae; Alternaria; Alternaria_alternata
New.ReferenceOTU6195	0	0	0	0	2	0	0	0	2	0	1	3	0	0	0	0	0	0	0	0	0	0	0	1	0	0	0	1	0	0	Fungi; Ascomycota; Dothideomycetes; Pleosporales; Pleosporaceae; Alternaria; Alternaria_juxtiseptata
SH092956.06FU_HE584834_reps	0	0	0	0	0	0	0	0	0	0	0	0	0	0	0	0	0	0	0	0	0	1	0	0	0	0	0	0	0	0	Fungi; Ascomycota; Dothideomycetes; Capnodiales; Capnodiaceae; Capnodium; Capnodium_sp
SH092957.06FU_AY843148_reps	0	0	0	0	0	0	0	0	0	0	0	0	0	0	0	0	0	0	0	1	0	0	0	0	0	0	0	0	0	0	Fungi; Ascomycota; Dothideomycetes; Capnodiales; Capnodiaceae; Capnodium; Capnodium_sp
New.ReferenceOTU1995	0	0	0	0	0	0	0	0	0	0	0	0	0	0	0	0	0	0	0	0	0	0	0	0	1	0	0	0	0	0	Fungi; Ascomycota; Dothideomycetes; Capnodiales; Incertae_sedis; Capnobotryella; Capnobotryella_sp
New.ReferenceOTU2288	0	0	0	0	0	0	0	0	0	0	0	0	0	0	1	0	0	0	0	0	0	0	0	0	0	0	0	1	0	2	Fungi; Ascomycota; Dothideomycetes; Capnodiales; Incertae_sedis; Capnobotryella; Capnobotryella_sp
New.ReferenceOTU5693	0	0	0	0	0	0	0	0	0	0	0	1	0	0	0	0	0	0	0	0	0	0	0	0	0	0	0	0	0	0	Fungi; Ascomycota; Dothideomycetes; Capnodiales; Incertae_sedis; Capnobotryella; Capnobotryella_sp
SH095112.06FU_EU514225_refs	0	0	0	0	0	0	0	0	0	0	0	0	0	1	0	0	0	0	0	0	0	0	0	0	0	0	0	0	0	0	Fungi; Ascomycota; Dothideomycetes; Capnodiales; Mycosphaerellaceae; Uwebraunia; Uwebraunia_musae
SH021456.06FU_KC677912_reps_singleton	0	1	0	0	0	0	0	0	0	0	0	0	0	0	0	1	0	0	0	0	0	0	0	0	0	0	0	0	0	0	Fungi; Ascomycota; Dothideomycetes; Capnodiales; Mycosphaerellaceae; Zasmidium; Zasmidium_aporosae
New.ReferenceOTU7946	0	0	0	0	0	4	0	0	0	0	0	0	0	0	0	0	0	0	0	0	0	0	0	0	0	0	0	0	0	0	Fungi; Ascomycota; Dothideomycetes; Dothideales; Dothioraceae; Aureobasidium; Aureobasidium_sp_HB110
SH132778.06FU_AB568371_reps	0	0	0	0	0	3	0	0	0	0	0	0	0	0	0	0	0	0	0	0	0	0	0	0	0	0	0	0	0	0	Fungi; Ascomycota; Dothideomycetes; Dothideales; Dothioraceae; Aureobasidium; Aureobasidium_sp_HB110
New.ReferenceOTU7108	0	0	0	0	0	0	0	0	0	0	0	0	0	0	0	6	0	0	0	0	0	0	0	0	0	0	0	0	0	0	Fungi; Ascomycota; Dothideomycetes; Incertae_sedis; Eremomycetaceae; Arthrographis; Arthrographis_sp
SH128366.06FU_KF385285_reps	0	0	0	0	0	0	0	0	0	0	0	0	0	0	0	1	0	0	0	0	0	0	0	0	0	0	0	0	0	0	Fungi; Ascomycota; Dothideomycetes; Incertae_sedis; Eremomycetaceae; Arthrographis; Arthrographis_sp
New.ReferenceOTU2699	0	0	1	2	0	0	0	5	2	0	2	0	0	0	1	0	0	1	0	2	7	3	0	0	0	0	2	2	0	4	Fungi; Ascomycota; Dothideomycetes; Incertae_sedis; Eremomycetaceae; Arthrographis; Arthrographis_sp_Sl_2007a
SH023518.06FU_GQ219942_reps_singleton	0	0	0	0	0	0	0	0	0	0	0	0	0	0	0	0	0	0	0	1	0	0	0	0	0	0	0	0	0	0	Fungi; Ascomycota; Dothideomycetes; Incertae_sedis; Pseudeurotiaceae; Leuconeurospora; Leuconeurospora_sp
New.ReferenceOTU5351	0	0	0	0	0	0	0	0	0	0	0	0	0	0	0	0	0	0	0	0	1	0	0	0	0	0	0	0	0	0	Fungi; Ascomycota; Dothideomycetes; Pleosporales; Corynesporascaceae; Corynespora; Corynespora_cassiicola
New.ReferenceOTU914	0	0	0	0	0	0	1	0	0	0	0	0	0	0	0	0	0	0	0	0	1	0	1	0	0	0	0	0	0	1	Fungi; Ascomycota; Dothideomycetes; Pleosporales; Corynesporascaceae; Corynespora; Corynespora_olivacea
SH024196.06FU_JQ814357_reps_singleton	0	0	0	0	0	0	0	0	0	0	0	0	0	0	0	0	0	0	0	0	0	0	0	0	0	0	1	0	0	0	Fungi; Ascomycota; Dothideomycetes; Pleosporales; Corynesporascaceae; Corynespora; Corynespora_sp_FAEII7a
SH125550.06FU_KC870889_reps	0	0	0	0	0	0	0	0	0	0	0	0	0	0	2	0	0	0	0	0	0	0	0	0	0	0	3	0	0	1	Fungi; Ascomycota; Dothideomycetes; Pleosporales; Corynesporascaceae; Corynespora; Corynespora_sp_FAEII7a
New.ReferenceOTU6316	0	0	0	0	0	0	0	0	0	0	0	0	0	0	0	0	0	0	2	0	0	0	0	0	0	0	0	0	0	0	Fungi; Ascomycota; Dothideomycetes; Pleosporales; Cucurbitariaceae; Curreya; Curreya_austroafricana
New.ReferenceOTU1074	0	0	0	0	0	0	0	2	0	0	0	0	0	0	0	1	0	0	0	0	0	0	0	0	0	0	0	0	0	0	Fungi; Ascomycota; Dothideomycetes; Pleosporales; Cucurbitariaceae; Curreya; Curreya_sp

New.ReferenceOTU1842	0	0	0	0	0	2	1	0	0	1	0	1	3	0	0	1	0	0	0	5	1	0	1	0	0	0	0	0	0	0	Fungi; Ascomycota; Dothideomycetes; Pleosporales; Cucurbitariaceae; Curreya; Curreya_sp
New.ReferenceOTU201	0	0	0	0	0	0	0	0	0	0	0	0	0	1	0	0	0	0	0	0	0	0	0	0	0	0	0	0	0	0	Fungi; Ascomycota; Dothideomycetes; Pleosporales; Cucurbitariaceae; Curreya; Curreya_sp
New.ReferenceOTU2186	0	0	0	0	0	0	0	0	0	0	0	0	0	0	1	0	0	0	0	0	0	0	0	0	0	0	0	0	0	0	Fungi; Ascomycota; Dothideomycetes; Pleosporales; Cucurbitariaceae; Curreya; Curreya_sp
New.ReferenceOTU6721	0	0	0	0	0	0	0	0	0	0	0	0	6	0	0	0	0	0	0	0	2	0	0	0	0	0	0	0	0	0	Fungi; Ascomycota; Dothideomycetes; Pleosporales; Cucurbitariaceae; Curreya; Curreya_sp
SH004765.06FU_AF502852_reps_singleton	0	0	0	2	0	0	0	0	0	0	0	1	0	0	0	1	0	0	0	0	0	1	0	0	0	0	0	0	0	0	Fungi; Ascomycota; Dothideomycetes; Pleosporales; Cucurbitariaceae; Pyrenochaetopsis; Pyrenochaetopsis_leptospora
SH092993.06FU_JF740262_refs	0	0	5	18	1	0	6	3	5	0	2	15	6	6	9	7	15	3	14	3	3	18	19	1	11	0	9	4	0	23	Fungi; Ascomycota; Dothideomycetes; Pleosporales; Cucurbitariaceae; Pyrenochaetopsis; Pyrenochaetopsis_leptospora
SH092995.06FU_KC506221_reps	0	0	0	0	0	0	0	0	0	0	0	0	0	0	0	0	0	0	0	0	0	1	0	0	0	0	0	0	0	0	Fungi; Ascomycota; Dothideomycetes; Pleosporales; Cucurbitariaceae; Pyrenochaetopsis; Pyrenochaetopsis_leptospora
New.ReferenceOTU3037	0	0	1	0	0	0	0	0	0	0	0	0	0	0	0	6	4	0	0	0	0	4	0	0	0	0	1	1	0	1	Fungi; Ascomycota; Dothideomycetes; Pleosporales; Cucurbitariaceae; Pyrenochaetopsis; Pyrenochaetopsis_pratorum
New.ReferenceOTU2063	0	0	0	0	0	0	0	0	0	0	0	0	0	0	2	0	0	0	0	0	0	0	0	0	0	0	0	7	0	0	Fungi; Ascomycota; Dothideomycetes; Pleosporales; Dacampiaceae; Munkovalsaria; Munkovalsaria_appendiculata
New.ReferenceOTU6634	0	0	0	0	0	0	0	0	0	0	0	0	0	0	0	0	0	0	0	0	0	0	0	0	1	0	0	0	0	0	Fungi; Ascomycota; Dothideomycetes; Pleosporales; Dacampiaceae; Munkovalsaria; Munkovalsaria_appendiculata
New.ReferenceOTU5690	0	0	0	0	0	0	0	0	0	0	0	0	0	0	0	0	0	1	1	0	0	0	0	0	0	0	0	0	0	0	Fungi; Ascomycota; Dothideomycetes; Pleosporales; Didymosphaeriaceae; Didymosphaeria; Didymosphaeria_futilis
New.ReferenceOTU5917	0	0	0	0	0	0	0	0	0	0	0	0	0	0	0	0	0	0	1	0	0	0	0	0	0	0	0	0	0	0	Fungi; Ascomycota; Dothideomycetes; Pleosporales; Didymosphaeriaceae; Didymosphaeria; Didymosphaeria_futilis
New.ReferenceOTU6048	2	0	0	0	0	0	0	0	0	0	0	0	0	0	0	0	0	0	0	0	0	0	0	0	0	0	0	0	0	0	Fungi; Ascomycota; Dothideomycetes; Pleosporales; Didymosphaeriaceae; Didymosphaeria; Didymosphaeria_futilis
New.ReferenceOTU724	0	0	0	0	0	0	0	0	0	0	0	0	0	0	0	0	0	0	0	0	0	0	1	0	0	0	0	0	0	0	Fungi; Ascomycota; Dothideomycetes; Pleosporales; Incertae_sedis; Dictyosporium; Dictyosporium_toruloides
New.ReferenceOTU2134	0	0	0	0	0	0	0	0	0	0	0	0	0	0	0	0	0	0	0	0	0	0	2	0	0	0	0	0	0	0	Fungi; Ascomycota; Dothideomycetes; Pleosporales; Incertae_sedis; Pyrenochaeta; Pyrenochaeta_sp
New.ReferenceOTU5926	0	0	0	0	0	0	0	0	0	0	0	0	0	0	1	0	0	0	0	0	0	0	0	0	0	0	0	0	0	0	Fungi; Ascomycota; Dothideomycetes; Pleosporales; Incertae_sedis; Pyrenochaeta; Pyrenochaeta_sp
New.ReferenceOTU3725	0	0	0	0	0	0	0	0	0	0	0	0	0	0	0	0	0	0	1	0	0	0	0	0	0	0	0	0	0	0	Fungi; Ascomycota; Dothideomycetes; Pleosporales; Incertae_sedis; Pyrenochaeta; Pyrenochaeta_sp_5_12c
New.CleanUp.ReferenceOTU542	0	0	0	0	0	0	0	0	0	0	0	0	0	1	0	0	0	0	0	0	0	0	0	0	0	0	0	0	0	0	Fungi; Ascomycota; Dothideomycetes; Pleosporales; Lophiostomataceae; Lophiostoma; Lophiostoma_arundinis
New.ReferenceOTU6125	0	0	0	0	0	0	0	0	0	0	0	0	0	0	4	0	0	0	0	0	2	0	0	0	0	0	0	0	0	0	Fungi; Ascomycota; Dothideomycetes; Pleosporales; Lophiostomataceae; Lophiostoma; Lophiostoma_cf_cynaroidis_A33
New.ReferenceOTU7277	0	0	0	0	0	0	0	0	0	0	0	0	0	0	0	0	0	0	0	0	0	0	1	0	1	0	0	0	0	0	Fungi; Ascomycota; Dothideomycetes; Pleosporales; Lophiostomataceae; Lophiostoma; Lophiostoma_cf_cynaroidis_A33
New.ReferenceOTU7490	0	0	0	0	0	0	0	0	0	0	0	0	0	0	0	0	0	0	0	0	0	0	0	0	1	0	1	0	0	0	Fungi; Ascomycota; Dothideomycetes; Pleosporales; Lophiostomataceae; Lophiostoma; Lophiostoma_cf_cynaroidis_A33
New.ReferenceOTU7536	0	0	0	0	0	0	0	1	0	0	0	0	0	0	1	0	0	0	0	0	7	0	3	0	3	0	1	2	0	2	Fungi; Ascomycota; Dothideomycetes; Pleosporales; Lophiostomataceae; Lophiostoma; Lophiostoma_cf_cynaroidis_A33
New.ReferenceOTU2769	0	0	0	0	0	0	0	0	0	0	0	0	0	0	1	0	0	0	0	0	0	0	2	0	1	0	0	0	0	3	Fungi; Ascomycota; Dothideomycetes; Pleosporales; Lophiostomataceae; Lophiostoma; Lophiostoma_cynaroidis
New.ReferenceOTU2093	0	0	1	0	0	1	0	0	0	0	0	0	0	0	6	1	0	0	0	0	0	0	0	1	0	0	0	1	0	0	Fungi; Ascomycota; Dothideomycetes; Pleosporales; Lophiostomataceae; Lophiostoma; Lophiostoma_sp
New.ReferenceOTU2336	0	0	0	0	0	0	0	0	0	0	0	0	0	0	0	0	0	0	4	0	0	0	0	0	0	0	0	0	0	0	Fungi; Ascomycota; Dothideomycetes; Pleosporales; Lophiostomataceae; Lophiostoma; Lophiostoma_sp
New.ReferenceOTU3810	0	0	0	0	0	0	0	0	0	0	0	0	0	0	0	0	0	0	1	0	0	0	0	0	0	0	0	0	0	0	Fungi; Ascomycota; Dothideomycetes; Pleosporales; Lophiostomataceae; Lophiostoma; Lophiostoma_sp
New.ReferenceOTU4737	0	2	0	0	0	0	0	0	0	0	0	0	0	0	0	0	0	0	0	0	0	0	0	0	0	0	0	0	0	0	Fungi; Ascomycota; Dothideomycetes; Pleosporales; Lophiostomataceae; Lophiostoma; Lophiostoma_sp
SH018322.06FU_FJ528715_reps_singleton	0	0	0	0	0	0	0	0	0	0	0	0	0	0	1	0	0	0	0	0	0	0	0	0	0	0	0	0	0	0	Fungi; Ascomycota; Dothideomycetes; Pleosporales; Lophiostomataceae; Lophiostoma; Lophiostoma_sp
SH092095.06FU_AJ877200_reps	0	0	0	0	0	0	0	0	1	0	0	0	0	0	0	0	0	0	0	0	0	0	0	0	0	0	0	0	0	0	Fungi; Ascomycota; Dothideomycetes; Pleosporales; Lophiostomataceae; Lophiostoma; Lophiostoma_sp
SH092098.06FU_EU977287_reps	0	0	0	0	0	0	0	0	0	0	1	0	0	0	0	1	0	0	2	1	0	0	0	0	0	0	0	0	0	0	Fungi; Ascomycota; Dothideomycetes; Pleosporales; Lophiostomataceae; Lophiostoma; Lophiostoma_sp
New.ReferenceOTU5231	0	2	0	0	0	0	0	0	0	0	0	0	0	0	0	0	0	0	0	0	0	0	0	0	0	0	0	0	0	0	Fungi; Ascomycota; Dothideomycetes; Pleosporales; Lophiostomataceae; Lophiostoma; Lophiostoma_sp_OUCMBI101098
New.ReferenceOTU7492	0	0	0	0	0	0	0	0	0	0	1	0	0	0	2	0	0	0	0	0	0	0	0	0	0	0	0	0	0	0	Fungi; Ascomycota; Dothideomycetes; Pleosporales; Massarinaceae; Massarina; Massarina_armatispora
New.CleanUp.ReferenceOTU1716	0	0	0	0	0	0	0	0	0	0	1	0	0	0	0	0	0	0	0	0	0	0	0	0	0	0	0	0	0	0	Fungi; Ascomycota; Dothideomycetes; Pleosporales; Massarinaceae; Massarina; Massarina_sp
New.ReferenceOTU7976	0	0	0	0	0	0	5	0	0	0	0	1	0	0	0	0	0	0	0	0	0	0	0	0	0	0	0	0	0	0	Fungi; Ascomycota; Dothideomycetes; Pleosporales; Massarinaceae; Massarina; Massarina_sp
SH030001.06FU_JX974735_reps_singleton	0	0	0	0	0	3	11	0	0	0	0	1	0	0	0	0	0	0	0	0	0	0	0	0	0	0	0	0	0	1	Fungi; Ascomycota; Dothideomycetes; Pleosporales; Massarinaceae; Massarina; Massarina_sp
New.ReferenceOTU6038	0	0	0	0	0	0	0	2	0	0	0	0	0	0	0	0	0	0	0	0	0	0	0	0	0	0	0	0	0	0	Fungi; Ascomycota; Dothideomycetes; Pleosporales; Massarinaceae; Massarina; Massarina_sp_JP_2013
New.ReferenceOTU4728	0	0	0	0	0	0	0	0	0	0	0	0	0	0	0	0	0	0	0	0	0	1	0	0	0	0	3	0	0	0	Fungi; Ascomycota; Dothideomycetes; Pleosporales; Melanommataceae; Astrosphaeriella; Astrosphaeriella_bakeriana
New.ReferenceOTU4981	0	0	0	0	0	0	0	0	0	0	0	0	0	0	0	3	0	0	0	0	0	0	1	0	0	0	0	0	0	0	Fungi; Ascomycota; Dothideomycetes; Pleosporales; Montagnulaceae; Microsphaeropsis; Microsphaeropsis_arundinis
New.ReferenceOTU598	0	0	0	0	0	0	0	2	0	0	0	0	2	0	0	2	0	0	0	0	0	27	0	0	0	0	0	1	0	3	Fungi; Ascomycota; Dothideomycetes; Pleosporales; Montagnulaceae; Microsphaeropsis; Microsphaeropsis_arundinis
New.ReferenceOTU6985	0	0	0	0	0	0	0	0	0	0	0	0	0	0	0	0	0	0	0	0	0	0	0	0	0	0	0	1	0	2	Fungi; Ascomycota; Dothideomycetes; Pleosporales; Montagnulaceae; Microsphaeropsis; Microsphaeropsis_arundinis
New.ReferenceOTU7049	0	0	0	0	0	0	0	0	0	0	0	0	0	0	0	0	0	0	0	0	0	0	0	0	0	0	0	0	0	1	Fungi; Ascomycota; Dothideomycetes; Pleosporales; Montagnulaceae; Microsphaeropsis; Microsphaeropsis_arundinis
SH005690.06FU_HQ007244_reps_singleton	0	0	0	0	0	0	0	0	0	0	0	0	0	0	0	3	0	0	0	0	0	5	0	0	0	0	0	0	0	1	Fungi; Ascomycota; Dothideomycetes; Pleosporales; Montagnulaceae; Microsphaeropsis; Microsphaeropsis_arundinis
SH094608.06FU_AY642530_reps	0	0	0	0	0	0	0	0	0	0	0	0	1	0	0	3	1	0	0	0	0	5	1	0	1	0	2	1	0	4	Fungi; Ascomycota; Dothideomycetes; Pleosporales; Montagnulaceae; Microsphaeropsis; Microsphaeropsis_arundinis
SH126621.06FU_EF094552_reps	0	0	0	0	0	0	0	0	0	0	0	0	0	0	0	2	0	0	0	0	0	0	2	0	2	0	1	2	0	4	Fungi; Ascomycota; Dothideomycetes; Pleosporales; Montagnulaceae; Microsphaeropsis; Microsphaeropsis_arundinis
New.ReferenceOTU2656	0	0	0	0	0	0	1	0	0	0	0	0	0	0	0	0	0	0	0	0	0	0	0	0	0	0	0	0	0	0	Fungi; Ascomycota; Dothideomycetes; Pleosporales; Montagnulaceae; Montagnula; Montagnula_alo?÷??√µs
New.ReferenceOTU5387	0	0	0	0	0	0	0	0	0	0	0	0	0	0	0	0	0	0	0	0	0	0	2	0	0	0	2	0	0	0	Fungi; Ascomycota; Dothideomycetes; Pleosporales; Montagnulaceae; Montagnula; Montagnula_alo?÷??√µs
New.ReferenceOTU6236	0	0	0	0	0	0	0	0	0	0	4	0	0	0	0	0	0	0	0	0	0	0	0	0	0	0	0	0	0	0	Fungi; Ascomycota; Dothideomycetes; Pleosporales; Montagnulaceae; Montagnula; Montagnula_alo?÷??√µs
New.ReferenceOTU1118	0	0	0	0	0	0	0	0	0	2	0	0	0	0	0	0	0	0	0	0	0	0	0	0	0	0	0	0	0	0	Fungi; Ascomycota; Dothideomycetes; Pleosporales; Montagnulaceae; Paraconiothyrium; Paraconiothyrium_sp
New.ReferenceOTU296	0	0	0	0	0	0	0	1	0	0	0	0	0	0	137	0	0	0	0	0	9	0	6	0	2	0	3	9	0	21	Fungi; Ascomycota; Dothideomycetes; Pleosporales; Montagnulaceae; Paraconiothyrium; Paraconiothyrium_sp
SH126632.06FU_JQ388919_reps	0	0	0	0	0	0	0	0	0	0	0	0	0	0	0	0	0	0	0	0	0	1	0	0	0	0	0	0	0	1	Fungi; Ascomycota; Dothideomycetes; Pleosporales; Montagnulaceae; Paraphaeosphaeria; Paraphaeosphaeria_sp_HKA26
New.ReferenceOTU4024	0	0	0	0	0	0	1	0	0	0	0	0	0	0	0	0	0	0	0	0	0	0	0	0	0	0	0	0	0	0	Fungi; Ascomycota; Dothideomycetes; Pleosporales; Montagnulaceae; Winterella; Winterella_aurantiaca
SH092999.06FU_DQ092510_reps	0	0	0	0	0	0	0	0	0	0	0	0	0	0	0	0	0	0	0	0	0	0	1	0	0	0	0	0	0	0	Fungi; Ascomycota; Dothideomycetes; Pleosporales; Phaeosphaeriaceae; Phaeosphaeria; Phaeosphaeria_sp_HKA27
New.CleanUp.ReferenceOTU461	0	0	0	0	0	0	0	0	0	0	0	0	1	0	0	0	0	0	0	0	0	0	0	0	0	0	0	0	0	0	Fungi; Ascomycota; Dothideomycetes; Pleosporales; Sporormiaceae; Preussia; Preussia_minima
New.ReferenceOTU2286	0	0	0	0	0	0	0	0	0	0	0	0	0	0	0	0	0	0	0	0	0	0	1	0	0	0	0	1	0	0	Fungi; Ascomycota; Dothideomycetes; Pleosporales; Sporormiaceae; Preussia; Preussia_minima
New.ReferenceOTU486	0	0	0	0	0	0	0	0	0	1	0	0	0	0	0	0	0	0	0	0	0	0	0	0	0	0	0	0	0	0	Fungi; Ascomycota; Dothideomycetes; Pleosporales; Sporormiaceae; Preussia; Preussia_polymorpha
SH086017.06FU_DQ468027_reps	0	0	0	0	0	0	0	0	0	0	0	0	0	0	0	0	0	0	0	0	0	2	0	0	12	0	0	0	0	1	Fungi; Ascomycota; Dothideomycetes; Pleosporales; Sporormiaceae; Preussia; Preussia_pseudominima
SH086032.06FU_DQ468034_reps	0	0	0	0	0	0	0	1	0	0	1	0	0	0	0	0	0	0	0	0	0	0	0	0	2	0	0	4	0	0	Fungi; Ascomycota; Dothideomycetes; Pleosporales; Sporormiaceae; Preussia; Preussia_pseudominima
New.ReferenceOTU4738	0	0	0	0	0	0	0	0	0	0	0	0	0	0	0	0	0	0	0	0	0	0	0	0	2	0	0	0	0	0	Fungi; Ascomycota; Dothideomycetes; Pleosporales; Sporormiaceae; Preussia; Preussia_sp
New.ReferenceOTU614	0	0	0	0	0	0	0	1	0	0	0	0	0	1	0	0	0	0	0	0	0	0	0	0	0	0	0	0	0	0	Fungi; Ascomycota; Dothideomycetes; Pleosporales; Sporormiaceae; Preussia; Preussia_sp
New.ReferenceOTU7830	0	0	0	0	0	0	0	0	0	1	0	0	0	0	0	0	0	0	0	0	0	0	0	0	0	0	0	0	0	0	Fungi; Ascomycota; Dothideomycetes; Pleosporales; Sporormiaceae; Preussia; Preussia_sp
SH004864.06FU_FJ528717_reps_singleton	0	0	0	0	0	0	0	2	0	0	0	0	0	0	0	0	0	0	0	0	0	0	0	0	0	0	0	0	0	0	Fungi; Ascomycota; Dothideomycetes; Pleosporales; Sporormiaceae; Preussia; Preussia_sp
SH086016.06FU_GQ203786_reps	0	0	0	0	0	0	0	0	0	0	0	0	0	0	0	0	0	0	0	0	0	0	0	0	1	0	0	0	0	0	Fungi; Ascomycota; Dothideomycetes; Pleosporales; Sporormiaceae; Preussia; Preussia_sp
SH086057.06FU_KC354524_reps	0	0	0	0	0	0	0	0	0	0	0	0	0	0	0	0	0	0	0	0	0	0	0	0	2	0	0	0	0	1	Fungi; Ascomycota; Dothideomycetes; Pleosporales; Sporormiaceae; Preussia; Preussia_sp
SH086065.06FU_FR668022_reps	0	0	0	0	0	1	0	0	0	0	0	0	0	0	0	0	0	0	0	0	0	0	0	0	0	0	0	2	0	0	Fungi; Ascomycota; Dothideomycetes; Pleosporales; Sporormiaceae; Preussia; Preussia_sp_4985
SH140270.06FU_GQ203765_reps	0	0	0	0	0	0	0	0	0	0	0	0	0	1	0	0	0	0	0	0	0	0	0	0	0	0	0	0	0	0	Fungi; Ascomycota; Dothideomycetes; Pleosporales; Sporormiaceae; Preussia; Preussia_terricola
New.ReferenceOTU5623	0	0	0	0	0	0	0	0	0	0	0	0	0	0	0	1	0	0	0	0	0	0	2	0	0	0	2	0	0	1	Fungi; Ascomycota; Dothideomycetes; Pleosporales; Sporormiaceae; Westerdykella; Westerdykella_ornata
New.ReferenceOTU5806	0	0	0	0	0	0	0	0	0	0	0	0	0	0	11	11	0	0	0	0	1	9	27	0	35	0	67	0	0	10	Fungi; Ascomycota; Dothideomycetes; Pleosporales; Sporormiaceae; Westerdykella; Westerdykella_ornata
New.ReferenceOTU4731	0	0	0	0	0	0	0	0	0	0	0	0	0	0	0	0	0	0	0	0	0	1	0	0	0	0	0	0	0	0	Fungi; Ascomycota; Dothideomycetes; Pleosporales; Tetraplosphaeriaceae; Tetraplosphaeria; Tetraplosphaeria_sasicola
New.ReferenceOTU6519	0	0	1	0	0	0	0	0	0	0	0	0	0	0	0	0	0	0	0	0	0	0	0	0	0	0	0	0	0	1	Fungi; Ascomycota; Dothideomycetes; Pleosporales; Tetraplosphaeriaceae; Tetraplosphaeria; Tetraplosphaeria_sasicola
New.ReferenceOTU82	0	0	0	0	0	0	0	0	0	0	0	0	0	0	0	0	0	0	0	0	0	0	0	0	0	0	4	0	0	0	Fungi; Ascomycota; Dothideomycetes; Pleosporales; Tetraplosphaeriaceae; Triplosphaeria; Triplosphaeria_sp_HC_4665
New.CleanUp.ReferenceOTU1334	0	0	0	0	0	0	0	1	0	0	0	0	0	0	0	0	0	0	0	0	0	0	0	0	0	0	0	0	0	0	Fungi; Ascomycota; Dothideomycetes; Pleosporales; Tubeufiaceae; Helicoma; Helicoma_sp_BCC_3512
New.ReferenceOTU2333	0	0	0	0	0	0	0	0	0	0	0	0	0	0	0	0	0	0	0	4	0	0	0	0	0	0	0	0	0	0	Fungi; Ascomycota; Dothideomycetes; Pleosporales; Tubeufiaceae; Helicomyces; Helicomyces_roseus
New.ReferenceOTU6934	0	0	0	0	0	0	0	0	0	0	0	0	0	0	0	0	0	0	1	0	0	0	0	0	0	0	0	0	0	0	Fungi; Ascomycota; Dothideomycetes; Pleosporales; Tubeufiaceae; Helicosporium; Helicosporium_gracile
New.ReferenceOTU2697	0	0	0	0	0	0	0	0	0	0	0	0	0	0	0	1	0	0	0	0	0	0	0	0	0	0	0	0	0	0	Fungi; Ascomycota; Dothideomycetes; Pleosporales; Tubeufiaceae; Tubeufia; Tubeufia_cerea
New.ReferenceOTU341	0	0	0	0	0	0	0	0	0	0	1	0	0	0	0	0	0	0	0	0	0	0	0	0	0	0	0	0	0	0	Fungi; Ascomycota; Eurotiomycetes; Chaetothyriales; Chaetothyriaceae; Cyphellophora; Cyphellophora_cf_pluriseptata_CBS_28585
New.ReferenceOTU3575	0	0	0	0	0	0	0	0	3	0	0	0	0	0	0	0	0	0	0	0	0	0	0	0	0	0	0	0	0	0	Fungi; Ascomycota; Eurotiomycetes; Chaetothyriales; Chaetothyriaceae; Cyphellophora; Cyphellophora_eugeniae
New.CleanUp.ReferenceOTU2175	0	0	0	0	0	0	0	0	0	0	0	0	0	0	0	0	1	0	0	0	0	0	0	0	0	0	0	0	0	0	Fungi; Ascomycota; Eurotiomycetes; Chaetothyriales; Chaetothyriaceae; Cyphellophora; Cyphellophora_guyanensis
New.ReferenceOTU176	0	0	0	0	0	0	0	0	0	0	0	0	0	1	0	2	0	0	0	0	0	0	0	0	0	0	0	0	0	0	Fungi; Ascomycota; Eurotiomycetes; Chaetothyriales; Chaetothyriaceae; Cyphellophora; Cyphellophora_guyanensis
New.ReferenceOTU3788	0	0	0	0	0	0	0	0	0	0	0	0	0	0	0	1	3	0	0	0	0	0	0	0	0	0	0	0	0	1	Fungi; Ascomycota; Eurotiomycetes; Chaetothyriales; Chaetothyriaceae; Cyphellophora; Cyphellophora_guyanensis
New.ReferenceOTU568	0	0	0	0	0	0	0	0	0	0	0	0	0	0	0	0	4	0	0	0	0	2	0	0	0	0	0	0	0	0	Fungi; Ascomycota; Eurotiomycetes; Chaetothyriales; Chaetothyriaceae; Cyphellophora; Cyphellophora_guyanensis
New.ReferenceOTU6734	0	0	1	5	0	1	0	1	0	0	0	0	2	0	0	0	7	0	0	0	0	0	0	0	0	0	0	0	0	8	Fungi; Ascomycota; Eurotiomycetes; Chaetothyriales; Chaetothyriaceae; Cyphellophora; Cyphellophora_guyanensis
SH115207.06FU_JQ766433_reps	0	0	0	3	0	0	0	0	0	0	2	0	0	1	1	0	5	0	0	0	0	0	1	0	2	0	0	0	0	4	Fungi; Ascomycota; Eurotiomycetes; Chaetothyriales; Chaetothyriaceae; Cyphellophora; Cyphellophora_guyanensis
New.CleanUp.ReferenceOTU262	0	0	0	0	0	0	0	0	0	0	0	0	0	0	1	0	0	0	0	0	0	0	0	0	0	0	0	0	0	0	Fungi; Ascomycota; Eurotiomycetes; Chaetothyriales; Chaetothyriaceae; Cyphellophora; Cyphellophora_reptans
New.ReferenceOTU6824	0	0	1	1	0	0	0	0	0	0	0	0	0	0	0	0	0	0	0	0	0	0	0	0	0	0	0	0	0	0	Fungi; Ascomycota; Eurotiomycetes; Chaetothyriales; Chaetothyriaceae; Cyphellophora; Cyphellophora_reptans
New.ReferenceOTU7749	0	0	0	0	0	0	0	0	0	0	0	0	0	0	0	0	0	0	0	0	0	1	0	0	0	0	0	0	0	0	Fungi; Ascomycota; Eurotiomycetes; Chaetothyriales; Chaetothyriaceae; Cyphellophora; Cyphellophora_reptans
New.ReferenceOTU8062	0	0	0	0	0	0	0	0	0	0	0	0	0	0	0	0	0	0	0	1	0	0	0	0	0	0	0	0	0	0	Fungi; Ascomycota; Eurotiomycetes; Chaetothyriales; Chaetothyriaceae; Cyphellophora; Cyphellophora_reptans
New.ReferenceOTU404	0	0	0	0	0	0	0	0	0	0	0	0	0	0	0	0	1	0	0	0	0	0	0	0	0	0	0	0	0	0	Fungi; Ascomycota; Eurotiomycetes; Chaetothyriales; Chaetothyriaceae; Cyphellophora; Cyphellophora_sp
New.ReferenceOTU7554	0	0	0	0	0	0	0	0	0	0	0	1	2	0	0	0	0	0	0	0	0	0	0	0	0	0	0	0	0	0	Fungi; Ascomycota; Eurotiomycetes; Chaetothyriales; Herpotrichiellaceae; Brycekendrickomyces; Brycekendrickomyces_acaciae
SH434976.06FU_FJ839606_refs_singleton	0	0	1	0	0	0	0	0	0	0	0	0	0	0	0	0	0	0	0	0	0	0	0	0	0	0	0	0	0	0	Fungi; Ascomycota; Eurotiomycetes; Chaetothyriales; Herpotrichiellaceae; Brycekendrickomyces; Brycekendrickomyces_acaciae
New.CleanUp.ReferenceOTU679	0	0	0	0	0	0	1	0	0	0	0	0	0	0	0	0	0	0	0	0	0	0	0	0	0	0	0	0	0	0	Fungi; Ascomycota; Eurotiomycetes; Chaetothyriales; Herpotrichiellaceae; Cladophialophora; Cladophialophora_arxii
New.ReferenceOTU3697	0	0	0	0	0	0	0	0	0	2	0	0	0	0	0	0	0	0	0	0	0	0	0	0	0	0	0	0	0	0	Fungi; Ascomycota; Eurotiomycetes; Chaetothyriales; Herpotrichiellaceae; Cladophialophora; Cladophialophora_chaetospira
New.ReferenceOTU5689	1	0	0	0	0	0	0	0	0	1	0	0	0	0	0	0	0	0	0	0	0	0	1	0	1	0	0	0	0	0	Fungi; Ascomycota; Eurotiomycetes; Chaetothyriales; Herpotrichiellaceae; Cladophialophora; Cladophialophora_immunda
SH111324.06FU_EU137318_refs	0	0	0	0	0	0	0	0	0	1	0	0	0	0	0	0	0	0	0	0	0	0	0	0	0	0	0	0	0	0	Fungi; Ascomycota; Eurotiomycetes; Chaetothyriales; Herpotrichiellaceae; Cladophialophora; Cladophialophora_immunda
New.ReferenceOTU1440	0	0	1	0	0	0	0	0	0	0	0	0	0	0	0	0	0	0	0	0	0	0	0	0	0	0	0	0	0	0	Fungi; Ascomycota; Eurotiomycetes; Chaetothyriales; Herpotrichiellaceae; Cladophialophora; Cladophialophora_sp
New.ReferenceOTU1684	0	0	0	0	0	0	0	0	0	0	0	0	0	0	0	0	0	0	0	0	0	0	0	0	0	0	1	0	0	1	Fungi; Ascomycota; Eurotiomycetes; Chaetothyriales; Herpotrichiellaceae; Cladophialophora; Cladophialophora_sp
New.ReferenceOTU1877	0	0	0	0	0	0	0	0	0	0	0	0	0	0	0	0	0	0	0	0	0	0	0	0	0	0	0	5	0	1	Fungi; Ascomycota; Eurotiomycetes; Chaetothyriales; Herpotrichiellaceae; Cladophialophora; Cladophialophora_sp
New.ReferenceOTU191	1	0	0	1	0	0	0	0	2	0	0	0	0	0	0	0	0	0	0	0	0	0	0	1	0	0	0	0	0	0	Fungi; Ascomycota; Eurotiomycetes; Chaetothyriales; Herpotrichiellaceae; Cladophialophora; Cladophialophora_sp
New.ReferenceOTU1912	0	0	0	0	0	0	0	0	0	0	0	0	0	0	0	0	0	0	0	0	0	0	2	0	2	0	0	0	0	0	Fungi; Ascomycota; Eurotiomycetes; Chaetothyriales; Herpotrichiellaceae; Cladophialophora; Cladophialophora_sp
New.ReferenceOTU2206	0	0	0	0	0	0	1	1	0	0	0	0	0	0	0	0	0	0	0	0	0	0	0	0	0	0	0	2	0	0	Fungi; Ascomycota; Eurotiomycetes; Chaetothyriales; Herpotrichiellaceae; Cladophialophora; Cladophialophora_sp
New.ReferenceOTU2567	0	0	0	0	0	0	1	0	0	0	0	0	0	0	0	0	0	0	0	0	1	0	0	0	0	0	0	1	0	1	Fungi; Ascomycota; Eurotiomycetes; Chaetothyriales; Herpotrichiellaceae; Cladophialophora; Cladophialophora_sp

New.ReferenceOTU3274	1	0	0	0	0	0	0	0	0	0	0	0	0	0	0	0	0	0	0	0	0	0	0	0	0	0	0	1	0	0	Fungi; Ascomycota; Eurotiomycetes; Chaetothyriales; Herpotrichiellaceae; Cladophialophora; Cladophialophora_sp
New.ReferenceOTU376	0	0	0	1	0	0	0	0	0	0	0	0	0	0	0	0	0	0	0	0	0	0	0	0	0	0	0	0	0	0	Fungi; Ascomycota; Eurotiomycetes; Chaetothyriales; Herpotrichiellaceae; Cladophialophora; Cladophialophora_sp
New.ReferenceOTU4768	0	0	0	0	0	0	0	0	0	0	1	0	0	0	0	0	0	0	0	0	0	0	0	0	0	0	0	0	0	0	Fungi; Ascomycota; Eurotiomycetes; Chaetothyriales; Herpotrichiellaceae; Cladophialophora; Cladophialophora_sp
New.ReferenceOTU5366	0	0	0	0	0	0	0	0	0	0	0	0	0	0	0	0	0	0	0	0	0	0	0	0	0	0	0	1	0	0	Fungi; Ascomycota; Eurotiomycetes; Chaetothyriales; Herpotrichiellaceae; Cladophialophora; Cladophialophora_sp
New.ReferenceOTU5667	0	0	0	0	0	0	0	0	0	0	0	0	0	1	0	0	0	0	0	0	0	0	0	0	0	0	0	0	0	0	Fungi; Ascomycota; Eurotiomycetes; Chaetothyriales; Herpotrichiellaceae; Cladophialophora; Cladophialophora_sp
New.ReferenceOTU7542	0	0	0	0	0	0	0	0	0	0	0	0	0	0	0	0	0	0	0	0	0	0	0	0	0	0	3	0	0	0	Fungi; Ascomycota; Eurotiomycetes; Chaetothyriales; Herpotrichiellaceae; Cladophialophora; Cladophialophora_sp
New.ReferenceOTU7675	0	0	1	0	0	0	0	0	0	3	0	0	0	0	0	0	0	0	0	0	0	0	0	0	0	0	0	0	0	0	Fungi; Ascomycota; Eurotiomycetes; Chaetothyriales; Herpotrichiellaceae; Cladophialophora; Cladophialophora_sp
New.ReferenceOTU2577	1	0	0	1	2	0	0	0	1	0	0	0	5	0	0	0	0	0	0	0	0	0	0	0	0	0	0	3	0	0	Fungi; Ascomycota; Eurotiomycetes; Chaetothyriales; Herpotrichiellaceae; Cladophialophora; Cladophialophora_sp_99003e
New.ReferenceOTU2642	0	0	0	0	0	0	0	0	2	0	6	0	0	0	0	0	0	0	0	0	0	0	1	0	0	0	6	0	0	0	Fungi; Ascomycota; Eurotiomycetes; Chaetothyriales; Herpotrichiellaceae; Cladophialophora; Cladophialophora_sp_99003e
New.ReferenceOTU2940	0	0	0	0	0	0	0	0	0	0	0	0	0	0	0	0	0	0	0	0	0	0	1	0	0	0	0	1	0	1	Fungi; Ascomycota; Eurotiomycetes; Chaetothyriales; Herpotrichiellaceae; Cladophialophora; Cladophialophora_sp_LS_2013
New.ReferenceOTU3419	0	0	0	0	0	0	0	0	0	0	0	0	0	0	13	0	0	0	0	0	0	0	0	0	0	0	0	0	0	0	Fungi; Ascomycota; Eurotiomycetes; Chaetothyriales; Herpotrichiellaceae; Cladophialophora; Cladophialophora_sp_MJP_2011
New.ReferenceOTU6414	0	0	0	0	0	0	0	0	0	0	0	0	0	0	0	0	0	0	0	0	0	0	0	0	0	0	0	0	0	2	Fungi; Ascomycota; Eurotiomycetes; Chaetothyriales; Herpotrichiellaceae; Cladophialophora; Cladophialophora_sp_R43C3
New.ReferenceOTU7400	0	0	0	0	0	0	0	0	0	0	0	0	0	0	0	0	0	0	0	0	0	0	0	0	0	0	0	0	0	9	Fungi; Ascomycota; Eurotiomycetes; Chaetothyriales; Herpotrichiellaceae; Cladophialophora; Cladophialophora_sp_R43C3
New.ReferenceOTU7364	0	0	0	0	0	0	0	0	0	0	0	0	0	0	0	1	0	0	0	0	0	0	0	0	0	0	0	0	0	0	Fungi; Ascomycota; Eurotiomycetes; Chaetothyriales; Herpotrichiellaceae; Exophiala; Exophiala_dermatitidis
New.ReferenceOTU6009	0	0	1	0	0	0	0	0	0	0	0	0	0	0	0	0	0	0	0	0	0	0	0	0	0	0	0	0	0	0	Fungi; Ascomycota; Eurotiomycetes; Chaetothyriales; Herpotrichiellaceae; Exophiala; Exophiala_phaeomuriformis
New.ReferenceOTU3318	0	0	0	0	0	0	0	0	0	0	1	0	0	0	0	0	0	0	0	0	0	0	0	0	0	0	0	0	0	0	Fungi; Ascomycota; Eurotiomycetes; Chaetothyriales; Herpotrichiellaceae; Exophiala; Exophiala_sp
New.ReferenceOTU7436	0	0	0	0	0	0	0	0	0	0	0	0	0	0	0	0	0	0	0	0	0	0	0	0	1	0	0	0	0	1	Fungi; Ascomycota; Eurotiomycetes; Chaetothyriales; Herpotrichiellaceae; Exophiala; Exophiala_spinifera
SH110035.06FU_AY156976_refs	0	0	0	0	0	0	0	0	0	0	0	0	0	0	0	0	0	0	0	0	0	0	0	0	1	0	0	0	0	0	Fungi; Ascomycota; Eurotiomycetes; Chaetothyriales; Herpotrichiellaceae; Exophiala; Exophiala_spinifera
New.ReferenceOTU3693	0	0	0	0	0	0	0	0	0	0	0	0	0	0	0	0	0	0	0	0	0	0	0	0	0	0	0	0	0	1	Fungi; Ascomycota; Eurotiomycetes; Chaetothyriales; Herpotrichiellaceae; Phaeomoniella; Phaeomoniella_effusa
New.ReferenceOTU3377	0	0	0	6	0	0	0	0	0	0	0	0	0	0	0	0	0	0	0	0	0	0	0	0	0	0	0	0	0	0	Fungi; Ascomycota; Eurotiomycetes; Chaetothyriales; Herpotrichiellaceae; Phaeomoniella; Phaeomoniella_sp_1_ICMP_18935
New.ReferenceOTU512	0	0	0	0	0	0	0	0	0	0	0	0	0	0	16	0	0	0	0	0	0	0	0	0	0	0	0	0	0	0	Fungi; Ascomycota; Eurotiomycetes; Chaetothyriales; Herpotrichiellaceae; Phaeomoniella; Phaeomoniella_sp_1_ICMP_18935
New.ReferenceOTU6761	0	0	0	0	0	0	0	0	0	0	1	0	0	0	0	0	0	0	0	0	0	0	0	0	0	0	0	0	0	0	Fungi; Ascomycota; Eurotiomycetes; Chaetothyriales; Herpotrichiellaceae; Rhinocladiella; Rhinocladiella_sp_MA_4653
New.ReferenceOTU3759	4	0	0	0	0	0	0	0	0	0	0	0	0	0	0	0	0	0	0	0	0	0	0	0	0	0	0	0	0	0	Fungi; Ascomycota; Eurotiomycetes; Eurotiales; Thermoascaceae; unidentified; Thermoascaceae_sp
New.ReferenceOTU4571	5	1	1	0	1	0	0	0	0	0	0	1	1	0	0	0	0	0	0	0	0	2	0	1	0	0	0	0	0	0	Fungi; Ascomycota; Eurotiomycetes; Eurotiales; Thermoascaceae; unidentified; Thermoascaceae_sp
New.ReferenceOTU543	6	0	0	1	1	0	0	0	0	0	1	3	0	0	0	0	0	1	1	0	0	0	0	0	0	0	0	0	0	0	Fungi; Ascomycota; Eurotiomycetes; Eurotiales; Thermoascaceae; unidentified; Thermoascaceae_sp
SH087354.06FU_KF225837_reps	4	0	0	2	0	0	0	0	0	0	1	1	0	0	0	0	0	1	0	1	0	0	0	0	1	0	0	1	0	0	Fungi; Ascomycota; Eurotiomycetes; Eurotiales; Thermoascaceae; unidentified; Thermoascaceae_sp
New.ReferenceOTU4404	0	1	0	0	0	1	0	0	0	0	0	0	3	0	0	0	1	0	1	0	0	0	0	2	0	0	1	0	0	0	Fungi; Ascomycota; Eurotiomycetes; Eurotiales; Trichocomaceae; Aspergillus; Aspergillus_aculeatus
SH032246.06FU_HE805124_reps_singleton	0	0	0	0	0	0	0	0	0	0	0	1	0	0	0	0	0	0	0	0	0	0	0	1	0	0	0	0	0	0	Fungi; Ascomycota; Eurotiomycetes; Eurotiales; Trichocomaceae; Aspergillus; Aspergillus_aculeatus
SH137894.06FU_HM055488_reps	0	0	0	0	0	2	0	1	0	0	0	0	6	3	0	4	2	0	0	3	0	0	1	2	3	0	1	0	0	0	Fungi; Ascomycota; Eurotiomycetes; Eurotiales; Trichocomaceae; Aspergillus; Aspergillus_aculeatus
New.ReferenceOTU3362	0	0	0	0	0	0	0	0	0	3	3	0	0	0	0	0	0	1	0	0	0	0	0	10	0	0	0	0	0	0	Fungi; Ascomycota; Eurotiomycetes; Eurotiales; Trichocomaceae; Aspergillus; Aspergillus_biplanus
New.ReferenceOTU7505	0	138	0	0	5	0	0	0	0	0	0	5	0	0	0	0	0	0	0	0	0	0	0	0	0	2	0	0	6	0	Fungi; Ascomycota; Eurotiomycetes; Eurotiales; Trichocomaceae; Aspergillus; Aspergillus_brevipes
New.ReferenceOTU2383	0	0	0	0	0	0	0	0	0	0	0	0	0	0	0	0	0	0	0	0	0	0	0	0	0	0	1	0	0	0	Fungi; Ascomycota; Eurotiomycetes; Eurotiales; Trichocomaceae; Aspergillus; Aspergillus_candidus
SH113523.06FU_AY373842_refs	0	0	0	0	0	0	0	0	0	0	0	0	0	0	0	0	0	0	0	0	0	0	0	0	1	0	0	0	0	0	Fungi; Ascomycota; Eurotiomycetes; Eurotiales; Trichocomaceae; Aspergillus; Aspergillus_candidus
SH103948.06FU_EU440766_reps	0	0	0	0	0	0	0	0	0	0	0	0	0	0	0	0	0	0	0	0	0	0	0	0	0	0	0	0	1	0	Fungi; Ascomycota; Eurotiomycetes; Eurotiales; Trichocomaceae; Aspergillus; Aspergillus_carbonarius
New.ReferenceOTU4787	0	0	0	0	0	0	0	0	0	0	0	0	0	0	0	0	0	1	4	0	0	0	0	0	0	0	0	0	0	0	Fungi; Ascomycota; Eurotiomycetes; Eurotiales; Trichocomaceae; Aspergillus; Aspergillus_cavernicola
SH114760.06FU_HQ026749_refs	0	0	0	0	0	0	0	1	0	0	0	0	0	0	0	0	0	0	0	0	0	0	0	0	0	0	0	0	0	0	Fungi; Ascomycota; Eurotiomycetes; Eurotiales; Trichocomaceae; Aspergillus; Aspergillus_clavatonanicus
New.CleanUp.ReferenceOTU2044	0	0	0	0	0	0	0	0	0	0	0	0	0	0	0	0	0	1	0	0	0	0	0	0	0	0	0	0	0	0	Fungi; Ascomycota; Eurotiomycetes; Eurotiales; Trichocomaceae; Aspergillus; Aspergillus_clavatus
New.ReferenceOTU5297	0	0	0	0	0	0	0	0	0	0	0	0	0	0	0	0	0	0	0	0	0	0	0	0	0	11	0	0	0	0	Fungi; Ascomycota; Eurotiomycetes; Eurotiales; Trichocomaceae; Aspergillus; Aspergillus_clavatus
New.ReferenceOTU710	0	0	0	0	0	0	0	0	0	0	0	0	0	0	0	0	0	0	1	0	0	0	0	0	0	0	0	0	0	0	Fungi; Ascomycota; Eurotiomycetes; Eurotiales; Trichocomaceae; Aspergillus; Aspergillus_clavatus
New.CleanUp.ReferenceOTU1253	0	0	0	0	0	0	0	0	0	0	0	0	0	0	0	0	0	0	0	0	1	0	0	0	0	0	0	0	0	0	Fungi; Ascomycota; Eurotiomycetes; Eurotiales; Trichocomaceae; Aspergillus; Aspergillus_crystallinus
New.CleanUp.ReferenceOTU1853	0	0	0	0	0	0	0	0	0	0	0	0	0	0	0	0	0	0	0	0	1	0	0	0	0	0	0	0	0	0	Fungi; Ascomycota; Eurotiomycetes; Eurotiales; Trichocomaceae; Aspergillus; Aspergillus_crystallinus
New.ReferenceOTU1124	1	2	8	12	6	12	3	6	5	11	9	5	10	3	3	4	4	0	0	0	15	0	4	4	1	0	0	3	1	2	Fungi; Ascomycota; Eurotiomycetes; Eurotiales; Trichocomaceae; Aspergillus; Aspergillus_crystallinus
New.ReferenceOTU1663	0	4	7	14	5	25	5	7	8	14	12	10	3	3	3	10	3	0	0	2	16	9	4	4	0	0	3	2	2	10	Fungi; Ascomycota; Eurotiomycetes; Eurotiales; Trichocomaceae; Aspergillus; Aspergillus_crystallinus
New.ReferenceOTU422	0	0	0	1	0	0	0	0	0	0	0	0	0	0	0	1	0	0	0	0	1	0	0	0	0	0	0	0	0	0	Fungi; Ascomycota; Eurotiomycetes; Eurotiales; Trichocomaceae; Aspergillus; Aspergillus_crystallinus
New.ReferenceOTU5109	0	0	0	0	0	1	0	0	0	0	0	0	0	0	0	0	0	0	0	0	0	0	0	0	0	0	0	0	0	0	Fungi; Ascomycota; Eurotiomycetes; Eurotiales; Trichocomaceae; Aspergillus; Aspergillus_crystallinus
New.ReferenceOTU5112	0	0	0	0	0	0	1	1	0	0	0	0	0	0	0	0	0	0	0	0	0	0	0	0	0	0	0	0	0	0	Fungi; Ascomycota; Eurotiomycetes; Eurotiales; Trichocomaceae; Aspergillus; Aspergillus_crystallinus
New.ReferenceOTU7030	0	0	1	1	0	0	0	0	0	0	0	0	0	0	0	0	0	0	0	0	0	0	1	0	0	0	0	0	0	0	Fungi; Ascomycota; Eurotiomycetes; Eurotiales; Trichocomaceae; Aspergillus; Aspergillus_crystallinus
New.ReferenceOTU7153	0	0	0	0	0	0	0	0	0	0	1	0	0	0	0	0	0	0	0	0	0	0	0	0	0	0	0	0	0	0	Fungi; Ascomycota; Eurotiomycetes; Eurotiales; Trichocomaceae; Aspergillus; Aspergillus_crystallinus
New.ReferenceOTU7860	2	0	0	0	0	0	0	0	0	0	0	0	0	2	0	0	0	0	0	9	0	0	4	0	0	0	0	0	0	3	Fungi; Ascomycota; Eurotiomycetes; Eurotiales; Trichocomaceae; Aspergillus; Aspergillus_crystallinus
SH098832.06FU_AF033482_refs	0	16	14	45	8	27	7	6	5	45	24	26	29	24	9	7	20	0	0	9	56	22	7	7	2	0	4	12	1	2	Fungi; Ascomycota; Eurotiomycetes; Eurotiales; Trichocomaceae; Aspergillus; Aspergillus_crystallinus
SH098866.06FU_AY360404_reps	0	0	0	1	0	0	0	0	0	0	1	0	0	0	0	0	0	0	0	0	0	0	0	0	0	0	0	0	0	0	Fungi; Ascomycota; Eurotiomycetes; Eurotiales; Trichocomaceae; Aspergillus; Aspergillus_crystallinus
SH114757.06FU_AY213669_refs	0	0	0	0	0	0	0	0	1	1	0	0	0	0	1	0	0	0	0	0	0	0	0	0	0	0	0	0	0	0	Fungi; Ascomycota; Eurotiomycetes; Eurotiales; Trichocomaceae; Aspergillus; Aspergillus_crystallinus
SH114800.06FU_DQ339555_refs	0	0	0	0	0	0	0	0	0	0	0	1	0	0	0	0	0	0	0	0	0	0	0	0	0	0	0	0	0	0	Fungi; Ascomycota; Eurotiomycetes; Eurotiales; Trichocomaceae; Aspergillus; Aspergillus_crystallinus
New.ReferenceOTU3914	0	4	0	0	0	0	0	0	3	0	0	0	0	0	0	2	0	1	0	0	0	0	0	2	0	1	0	0	0	0	Fungi; Ascomycota; Eurotiomycetes; Eurotiales; Trichocomaceae; Aspergillus; Aspergillus_kanagawaensis
New.ReferenceOTU6956	0	0	0	0	0	0	0	0	0	0	0	0	0	0	0	0	0	1	0	0	0	0	0	0	0	0	0	0	0	0	Fungi; Ascomycota; Eurotiomycetes; Eurotiales; Trichocomaceae; Aspergillus; Aspergillus_kanagawaensis
SH114761.06FU_EF661275_reps	0	14	2	0	2	0	0	0	2	9	11	3	0	6	0	4	0	3	0	5	0	0	0	13	0	15	0	1	14	0	Fungi; Ascomycota; Eurotiomycetes; Eurotiales; Trichocomaceae; Aspergillus; Aspergillus_kanagawaensis
SH103947.06FU_FJ629352_refs	0	0	1	0	0	0	0	0	0	0	0	0	0	0	0	0	0	0	0	0	0	0	0	0	2	0	0	0	0	0	Fungi; Ascomycota; Eurotiomycetes; Eurotiales; Trichocomaceae; Aspergillus; Aspergillus_niger
New.ReferenceOTU223	0	0	0	0	0	0	1	0	0	0	0	0	0	0	0	0	0	0	0	0	0	0	0	0	0	0	0	0	0	0	Fungi; Ascomycota; Eurotiomycetes; Eurotiales; Trichocomaceae; Aspergillus; Aspergillus_nomius
SH128406.06FU_AF338643_refs	0	0	0	0	0	0	5	0	0	0	0	0	0	1	0	0	0	0	0	0	0	0	0	0	0	0	0	1	0	0	Fungi; Ascomycota; Eurotiomycetes; Eurotiales; Trichocomaceae; Aspergillus; Aspergillus_nomius
New.ReferenceOTU2411	0	0	0	0	0	0	0	0	0	0	0	0	0	0	0	0	0	0	3	0	0	0	0	0	0	0	0	0	0	0	Fungi; Ascomycota; Eurotiomycetes; Eurotiales; Trichocomaceae; Aspergillus; Aspergillus_ochraceoroseus
New.ReferenceOTU2532	0	0	0	0	0	0	0	0	0	0	0	0	0	0	0	0	0	8	0	0	0	0	0	0	0	0	0	0	0	0	Fungi; Ascomycota; Eurotiomycetes; Eurotiales; Trichocomaceae; Aspergillus; Aspergillus_ochraceoroseus
New.ReferenceOTU2909	0	0	0	0	0	0	0	0	0	0	0	0	0	0	0	0	0	0	4	0	0	0	0	0	0	0	0	0	0	0	Fungi; Ascomycota; Eurotiomycetes; Eurotiales; Trichocomaceae; Aspergillus; Aspergillus_ochraceoroseus
New.ReferenceOTU3151	0	0	0	0	0	0	0	0	0	0	0	0	0	0	0	0	0	1	14	0	0	0	0	0	0	0	0	0	0	0	Fungi; Ascomycota; Eurotiomycetes; Eurotiales; Trichocomaceae; Aspergillus; Aspergillus_ochraceoroseus
New.CleanUp.ReferenceOTU31	0	0	0	0	0	0	0	0	0	0	0	0	0	1	0	0	0	0	0	0	0	0	0	0	0	0	0	0	0	0	Fungi; Ascomycota; Eurotiomycetes; Eurotiales; Trichocomaceae; Aspergillus; Aspergillus_pseudodeflectus
New.ReferenceOTU7361	0	0	0	0	0	0	1	0	0	0	0	0	0	2	0	0	1	0	0	0	0	0	0	0	0	0	0	0	0	1	Fungi; Ascomycota; Eurotiomycetes; Eurotiales; Trichocomaceae; Aspergillus; Aspergillus_pseudonomius
New.ReferenceOTU4701	0	0	0	0	0	0	0	0	0	0	0	0	0	0	0	0	0	0	0	0	0	0	0	0	3	0	0	0	0	0	Fungi; Ascomycota; Eurotiomycetes; Eurotiales; Trichocomaceae; Aspergillus; Aspergillus_sp
SH027164.06FU_HQ116395_reps_singleton	0	0	0	0	0	0	0	0	0	0	0	0	0	0	0	0	0	0	0	0	0	0	0	0	1	0	0	0	0	0	Fungi; Ascomycota; Eurotiomycetes; Eurotiales; Trichocomaceae; Aspergillus; Aspergillus_sp
New.CleanUp.ReferenceOTU1901	0	0	0	0	0	0	0	0	0	0	0	0	0	0	0	0	0	0	0	0	0	0	0	0	0	0	1	0	0	0	Fungi; Ascomycota; Eurotiomycetes; Eurotiales; Trichocomaceae; Aspergillus; Aspergillus_sp_ASR_279
New.ReferenceOTU4427	0	0	0	0	0	0	0	0	0	0	0	0	0	0	0	0	0	0	0	0	0	0	0	0	0	0	2	0	0	1	Fungi; Ascomycota; Eurotiomycetes; Eurotiales; Trichocomaceae; Aspergillus; Aspergillus_sp_ASR_279
New.ReferenceOTU6042	0	0	0	0	0	0	0	0	0	0	0	0	0	0	0	0	0	0	0	0	0	0	0	0	0	0	3	0	0	0	Fungi; Ascomycota; Eurotiomycetes; Eurotiales; Trichocomaceae; Aspergillus; Aspergillus_sp_ASR_279
New.ReferenceOTU1623	0	0	0	0	0	0	0	0	0	0	0	0	0	0	0	0	0	0	2	1	0	0	0	0	0	0	0	0	0	0	Fungi; Ascomycota; Eurotiomycetes; Eurotiales; Trichocomaceae; Aspergillus; Aspergillus_sp_E19D
SH110777.06FU_EF608614_reps	0	0	0	0	0	0	0	0	0	0	0	0	0	0	0	0	0	0	3	1	0	0	0	0	0	0	0	0	0	0	Fungi; Ascomycota; Eurotiomycetes; Eurotiales; Trichocomaceae; Aspergillus; Aspergillus_sp_E19D
New.ReferenceOTU5518	0	0	0	0	0	3	0	0	0	0	0	0	0	0	0	1	0	0	0	0	0	0	1	0	0	0	0	0	0	0	Fungi; Ascomycota; Eurotiomycetes; Eurotiales; Trichocomaceae; Aspergillus; Aspergillus_sp_F1
New.ReferenceOTU2499	0	0	0	0	3	0	0	0	0	0	0	0	0	0	0	0	0	0	0	0	0	0	0	0	0	0	0	0	0	0	Fungi; Ascomycota; Eurotiomycetes; Eurotiales; Trichocomaceae; Aspergillus; Aspergillus_sp_MS351
New.ReferenceOTU3925	0	2	0	0	0	0	0	0	0	0	0	0	0	0	0	0	0	0	0	0	0	0	0	0	0	0	0	0	0	0	Fungi; Ascomycota; Eurotiomycetes; Eurotiales; Trichocomaceae; Aspergillus; Aspergillus_sp_MS351
New.ReferenceOTU6323	0	0	0	0	0	0	0	0	0	0	0	0	0	0	0	0	0	0	0	0	0	0	0	0	1	0	0	0	0	0	Fungi; Ascomycota; Eurotiomycetes; Eurotiales; Trichocomaceae; Aspergillus; Aspergillus_sp_MS351
New.ReferenceOTU6423	0	0	0	0	0	0	0	0	0	0	0	0	0	0	0	0	0	0	0	0	0	0	0	0	0	0	0	0	1	0	Fungi; Ascomycota; Eurotiomycetes; Eurotiales; Trichocomaceae; Aspergillus; Aspergillus_sp_MS351
New.ReferenceOTU6570	0	0	0	0	0	0	0	0	0	0	0	0	0	0	0	0	0	0	0	0	0	0	0	0	1	0	0	0	0	0	Fungi; Ascomycota; Eurotiomycetes; Eurotiales; Trichocomaceae; Aspergillus; Aspergillus_sp_MS351
SH016750.06FU_JQ905779_reps_singleton	0	0	1	0	0	0	0	0	0	0	0	0	0	0	0	0	0	0	0	0	0	0	1	0	0	0	0	0	0	0	Fungi; Ascomycota; Eurotiomycetes; Eurotiales; Trichocomaceae; Aspergillus; Aspergillus_sp_MS351
New.ReferenceOTU4691	0	0	0	0	0	3	0	0	0	0	0	0	0	0	0	0	1	0	0	0	1	0	0	0	0	0	0	0	0	0	Fungi; Ascomycota; Eurotiomycetes; Eurotiales; Trichocomaceae; Aspergillus; Aspergillus_sp_NRRL_32683
SH110788.06FU_EF669595_reps	0	0	0	1	0	0	0	0	0	0	0	0	1	0	0	0	0	0	0	0	6	0	0	0	0	0	0	0	0	0	Fungi; Ascomycota; Eurotiomycetes; Eurotiales; Trichocomaceae; Aspergillus; Aspergillus_sp_NRRL_32683
New.ReferenceOTU4464	0	0	0	0	0	0	0	0	0	0	0	0	0	0	0	0	0	0	1	0	0	0	0	0	0	0	0	0	0	0	Fungi; Ascomycota; Eurotiomycetes; Eurotiales; Trichocomaceae; Aspergillus; Aspergillus_sparsus
New.ReferenceOTU5943	0	0	0	0	0	0	0	0	0	0	2	0	0	0	0	0	0	0	0	0	0	0	0	0	0	0	0	0	0	0	Fungi; Ascomycota; Eurotiomycetes; Eurotiales; Trichocomaceae; Aspergillus; Aspergillus_sparsus
New.ReferenceOTU6359	0	3	1	0	0	0	0	0	0	0	0	0	1	0	0	0	0	0	0	0	0	0	0	3	0	0	0	0	2	0	Fungi; Ascomycota; Eurotiomycetes; Eurotiales; Trichocomaceae; Aspergillus; Aspergillus_sparsus
New.ReferenceOTU7130	0	0	1	0	0	0	0	0	0	0	0	0	0	0	0	0	1	0	0	0	0	0	0	0	0	0	0	0	0	0	Fungi; Ascomycota; Eurotiomycetes; Eurotiales; Trichocomaceae; Aspergillus; Aspergillus_sparsus
New.ReferenceOTU815	0	0	0	0	1	0	0	0	0	0	0	0	0	0	0	0	1	0	0	1	0	0	0	0	0	0	0	0	0	0	Fungi; Ascomycota; Eurotiomycetes; Eurotiales; Trichocomaceae; Aspergillus; Aspergillus_sparsus
New.ReferenceOTU334	0	0	0	0	0	0	0	0	0	1	0	0	0	0	0	0	0	2	0	0	0	0	0	0	0	0	0	0	0	0	Fungi; Ascomycota; Eurotiomycetes; Eurotiales; Trichocomaceae; Aspergillus; Aspergillus_tanneri
New.ReferenceOTU3553	0	0	1	0	0	0	0	0	0	0	0	0	0	0	0	0	0	0	0	0	0	0	0	0	0	0	0	0	0	0	Fungi; Ascomycota; Eurotiomycetes; Eurotiales; Trichocomaceae; Aspergillus; Aspergillus_tanneri
New.ReferenceOTU5606	0	0	0	0	0	0	0	0	0	0	0	0	0	0	0	0	0	5	4	0	0	0	0	0	0	0	0	0	0	0	Fungi; Ascomycota; Eurotiomycetes; Eurotiales; Trichocomaceae; Aspergillus; Aspergillus_tanneri
New.ReferenceOTU8	0	3	14	0	0	0	0	0	0	24	8	0	0	0	0	0	1	16	1	1	1	0	0	0	0	1	0	0	4	0	Fungi; Ascomycota; Eurotiomycetes; Eurotiales; Trichocomaceae; Aspergillus; Aspergillus_tanneri
New.ReferenceOTU4810	0	0	0	0	0	0	0	0	0	0	0	0	0	0	0	1	1	0	0	2	0	0	1	0	0	0	0	0	0	0	Fungi; Ascomycota; Eurotiomycetes; Eurotiales; Trichocomaceae; Aspergillus; Aspergillus_tardus
New.ReferenceOTU6594	0	0	0	0	0	0	0	0	0	0	0	0	0	0	0	0	0	0	0	3	0	0	0	0	0	0	0	0	0	0	Fungi; Ascomycota; Eurotiomycetes; Eurotiales; Trichocomaceae; Aspergillus; Aspergillus_tardus
SH113543.06FU_FJ531044_reps	0	0	1	0	0	0	0	0	0	0	0	0	0	0	0	0	0	0	0	0	0	0	0	0	0	0	0	0	0	0	Fungi; Ascomycota; Eurotiomycetes; Eurotiales; Trichocomaceae; Aspergillus; Aspergillus_tardus
New.ReferenceOTU791	0	0	0	0	0	0	1	1	0	0	0	1	0	1	8	1	0	0	0	0	1	2	0	0	0	0	0	0	0	0	Fungi; Ascomycota; Eurotiomycetes; Eurotiales; Trichocomaceae; Aspergillus; Aspergillus_wentii
New.ReferenceOTU1201	0	0	1	0	0	0	0	0	0	0	0	0	0	0	0	8	0	0	0	0	1	3	0	0	0	0	0	1	0	0	Fungi; Ascomycota; Eurotiomycetes; Eurotiales; Trichocomaceae; Chaetosartorya; Chaetosartorya_cremea
New.ReferenceOTU7835	0	0	0	0	0	0	0	0	0	0	0	0	0	0	0	0	0	0	0	0	0	0	0	1	1	0	0	0	0	0	Fungi; Ascomycota; Eurotiomycetes; Eurotiales; Trichocomaceae; Chromocleista; Chromocleista_sp_CCF3816
New.CleanUp.ReferenceOTU1044	0	0	0	0	0	0	0	0	0	0	0	1	0	0	0	0	0	0	0	0	0	0	0	0	0	0	0	0	0	0	Fungi; Ascomycota; Eurotiomycetes; Eurotiales; Trichocomaceae; Emericella; Emericella_nidulans
New.ReferenceOTU1968	0	0	0	0	0	0	4	0	0	0	0	0	0	0	0	0	0	0	0	0	0	0	0	0	0	0	0	0	0	0	Fungi; Ascomycota; Eurotiomycetes; Eurotiales; Trichocomaceae; Emericella; Emericella_nidulans
New.ReferenceOTU3711	0	0	0	0	0	0	1	0	0	0	0	0	0	0	0	0	0	0	0	0	0	0	0	0	0	0	0	0	0	0	Fungi; Ascomycota; Eurotiomycetes; Eurotiales; Trichocomaceae; Emericella; Emericella_nidulans
New.ReferenceOTU6077	0	0	0	0	0	0	0	0	0	0	0	0	0	0	0	0	0	0	0	0	0	0	0	0	0	0	0	0	0	1	Fungi; Ascomycota; Eurotiomycetes; Eurotiales; Trichocomaceae; Emericella; Emericella_nidulans

New.ReferenceOTU6559	0	0	0	2	0	0	0	0	0	0	0	0	0	1	1	0	0	0	0	0	1	0	1	0	0	0	0	0	0	0	Fungi; Ascomycota; Eurotiomycetes; Eurotiales; Trichocomaceae; Emericella; Emericella_nidulans
New.ReferenceOTU5799	0	0	1	0	0	0	0	0	0	0	0	0	0	0	0	0	0	0	0	0	0	0	0	0	0	0	0	0	0	0	Fungi; Ascomycota; Eurotiomycetes; Eurotiales; Trichocomaceae; Eupenicillium; Eupenicillium_sp
New.ReferenceOTU6428	0	0	1	0	0	0	0	0	0	0	0	0	0	0	0	0	0	0	0	0	0	0	0	0	0	0	0	0	0	0	Fungi; Ascomycota; Eurotiomycetes; Eurotiales; Trichocomaceae; Eupenicillium; Eupenicillium_sp
SH098853.06FU_GU981573_reps	0	0	0	0	1	0	0	0	0	0	0	0	0	0	0	0	0	0	0	0	0	0	0	0	0	0	0	0	0	0	Fungi; Ascomycota; Eurotiomycetes; Eurotiales; Trichocomaceae; Eupenicillium; Eupenicillium_sp_4_JH_2010
New.ReferenceOTU2988	0	0	0	0	0	0	0	0	0	0	0	0	0	0	0	1	0	0	0	0	0	0	0	0	0	0	0	0	0	0	Fungi; Ascomycota; Eurotiomycetes; Eurotiales; Trichocomaceae; Hamigera; Hamigera_fusca
New.ReferenceOTU3485	0	36	0	0	0	0	0	0	0	0	0	0	0	0	1	0	0	0	0	0	0	0	0	0	0	0	0	0	0	0	Fungi; Ascomycota; Eurotiomycetes; Eurotiales; Trichocomaceae; Hamigera; Hamigera_fusca
New.ReferenceOTU785	0	0	0	0	0	0	0	0	0	0	0	0	0	0	0	1	0	0	0	0	0	0	0	0	0	0	0	0	0	0	Fungi; Ascomycota; Eurotiomycetes; Eurotiales; Trichocomaceae; Hamigera; Hamigera_fusca
SH135170.06FU_GU092938_reps	0	0	0	0	0	0	0	0	0	0	0	0	0	0	1	0	0	0	0	0	0	0	0	0	0	0	0	0	0	0	Fungi; Ascomycota; Eurotiomycetes; Eurotiales; Trichocomaceae; Hamigera; Hamigera_fusca
New.ReferenceOTU163	0	0	1	0	0	5	0	1	0	0	0	0	1	0	0	2	1	0	0	0	6	4	1	0	0	0	0	0	0	2	Fungi; Ascomycota; Eurotiomycetes; Eurotiales; Trichocomaceae; Merimbla; Merimbla_ingelheimensis
New.ReferenceOTU776	0	0	0	0	0	0	0	0	0	0	0	0	0	0	0	0	0	0	0	1	0	0	0	0	0	0	0	0	0	0	Fungi; Ascomycota; Eurotiomycetes; Eurotiales; Trichocomaceae; Merimbla; Merimbla_ingelheimensis
New.ReferenceOTU279	0	0	0	0	0	0	1	0	0	0	0	4	0	0	0	0	0	0	0	0	0	0	0	0	0	0	0	0	0	0	Fungi; Ascomycota; Eurotiomycetes; Eurotiales; Trichocomaceae; Neosartorya; Neosartorya_aurata
New.ReferenceOTU7574	0	0	0	0	0	0	0	0	0	0	0	4	0	0	0	0	0	0	0	0	0	0	0	0	0	0	0	0	0	0	Fungi; Ascomycota; Eurotiomycetes; Eurotiales; Trichocomaceae; Neosartorya; Neosartorya_aurata
SH114790.06FU_EF669979_reps	0	0	0	0	0	1	0	0	0	0	0	4	0	0	0	0	0	0	0	0	0	0	0	0	0	0	0	0	0	0	Fungi; Ascomycota; Eurotiomycetes; Eurotiales; Trichocomaceae; Neosartorya; Neosartorya_aurata
New.ReferenceOTU3867	0	0	0	0	0	0	2	0	0	0	0	0	0	0	0	0	0	0	0	0	0	0	0	0	0	0	2	1	0	0	Fungi; Ascomycota; Eurotiomycetes; Eurotiales; Trichocomaceae; Neosartorya; Neosartorya_fennelliae
New.ReferenceOTU5918	0	0	0	0	0	0	0	0	0	0	0	1	0	0	0	0	0	0	0	0	0	0	0	0	0	0	2	0	0	1	Fungi; Ascomycota; Eurotiomycetes; Eurotiales; Trichocomaceae; Neosartorya; Neosartorya_fennelliae
New.ReferenceOTU622	0	0	0	0	0	1	0	0	0	0	0	0	0	0	0	0	0	0	0	0	0	0	0	0	0	0	0	0	0	0	Fungi; Ascomycota; Eurotiomycetes; Eurotiales; Trichocomaceae; Neosartorya; Neosartorya_fennelliae
SH114771.06FU_JN943573_reps	0	0	0	0	0	0	0	0	0	0	0	0	0	0	0	0	0	0	0	0	0	0	0	0	0	0	1	0	0	1	Fungi; Ascomycota; Eurotiomycetes; Eurotiales; Trichocomaceae; Neosartorya; Neosartorya_fennelliae
New.ReferenceOTU1558	0	0	0	1	0	1	1	0	0	0	0	0	5	0	0	0	0	0	0	0	0	0	0	0	0	0	1	0	0	0	Fungi; Ascomycota; Eurotiomycetes; Eurotiales; Trichocomaceae; Neosartorya; Neosartorya_sp
New.ReferenceOTU2106	0	1	0	0	0	0	0	0	0	2	2	0	0	0	0	0	0	18	0	4	0	0	0	1	0	20	0	0	1	0	Fungi; Ascomycota; Eurotiomycetes; Eurotiales; Trichocomaceae; Neosartorya; Neosartorya_sp
New.ReferenceOTU2757	0	0	0	0	0	0	0	0	0	0	0	0	0	0	0	0	0	0	0	0	0	0	0	0	0	0	1	0	0	1	Fungi; Ascomycota; Eurotiomycetes; Eurotiales; Trichocomaceae; Neosartorya; Neosartorya_sp
New.ReferenceOTU4146	0	0	0	0	0	0	0	0	0	0	0	0	0	0	0	0	0	0	0	0	0	0	0	0	0	0	3	0	0	0	Fungi; Ascomycota; Eurotiomycetes; Eurotiales; Trichocomaceae; Neosartorya; Neosartorya_sp
New.ReferenceOTU4177	0	0	1	0	3	0	0	0	0	0	0	0	1	0	0	0	0	0	0	0	1	0	0	0	0	0	10	0	0	0	Fungi; Ascomycota; Eurotiomycetes; Eurotiales; Trichocomaceae; Neosartorya; Neosartorya_sp
New.ReferenceOTU7590	0	0	0	0	0	0	0	0	0	0	0	0	0	0	0	0	0	6	0	0	0	0	0	0	0	0	0	0	0	0	Fungi; Ascomycota; Eurotiomycetes; Eurotiales; Trichocomaceae; Neosartorya; Neosartorya_sp
SH096862.06FU_HQ026746_refs	0	0	0	0	0	0	0	0	0	0	0	1	0	0	0	0	0	0	0	0	0	0	0	0	0	0	4	0	0	1	Fungi; Ascomycota; Eurotiomycetes; Eurotiales; Trichocomaceae; Neosartorya; Neosartorya_sp
New.ReferenceOTU1902	0	0	0	1	0	0	0	0	0	1	0	0	0	0	0	0	0	0	0	0	0	0	0	0	0	0	1	0	0	0	Fungi; Ascomycota; Eurotiomycetes; Eurotiales; Trichocomaceae; Neosartorya; Neosartorya_spathulata
New.ReferenceOTU3654	0	0	0	0	0	0	0	0	0	0	0	0	0	0	0	0	0	0	0	0	0	0	0	0	0	0	1	0	0	0	Fungi; Ascomycota; Eurotiomycetes; Eurotiales; Trichocomaceae; Neosartorya; Neosartorya_spathulata
SH133863.06FU_DQ836183_reps	0	0	0	0	0	0	0	0	0	0	0	0	0	0	0	0	0	0	0	0	0	0	0	0	2	0	0	0	0	0	Fungi; Ascomycota; Eurotiomycetes; Eurotiales; Trichocomaceae; Paecilomyces; Paecilomyces_carneus
New.ReferenceOTU1916	0	0	3	0	0	0	0	0	0	0	0	0	0	0	0	0	0	0	0	0	0	0	0	0	0	0	1	0	0	0	Fungi; Ascomycota; Eurotiomycetes; Eurotiales; Trichocomaceae; Paecilomyces; Paecilomyces_cf_penicillatus_1_RH_2012
New.ReferenceOTU4782	0	0	0	0	0	3	0	0	0	1	0	1	0	0	0	0	0	0	0	0	0	0	1	0	0	0	7	0	0	0	Fungi; Ascomycota; Eurotiomycetes; Eurotiales; Trichocomaceae; Paecilomyces; Paecilomyces_fulvus
New.ReferenceOTU7080	0	0	0	0	0	3	0	1	1	0	5	0	0	0	0	0	0	0	0	0	0	0	0	0	0	0	6	1	0	0	Fungi; Ascomycota; Eurotiomycetes; Eurotiales; Trichocomaceae; Paecilomyces; Paecilomyces_fulvus
SH112356.06FU_FJ389940_refs	1	0	0	0	2	6	8	0	0	3	5	11	0	0	0	0	0	0	0	0	0	0	2	0	1	0	11	5	0	1	Fungi; Ascomycota; Eurotiomycetes; Eurotiales; Trichocomaceae; Paecilomyces; Paecilomyces_fulvus
SH132448.06FU_JQ846086_reps	0	0	0	0	0	0	0	0	0	0	0	0	0	0	0	0	1	0	0	0	0	0	0	0	0	0	0	0	0	0	Fungi; Ascomycota; Eurotiomycetes; Eurotiales; Trichocomaceae; Paecilomyces; Paecilomyces_marquandii
New.ReferenceOTU5493	0	0	0	0	0	0	0	0	1	0	0	0	0	0	0	0	0	0	0	0	0	0	0	0	0	0	0	0	0	0	Fungi; Ascomycota; Eurotiomycetes; Eurotiales; Trichocomaceae; Paecilomyces; Paecilomyces_niphetodes
New.ReferenceOTU4088	0	0	0	0	0	0	0	0	0	0	1	0	0	0	0	0	0	0	0	0	0	0	0	0	0	0	0	0	0	0	Fungi; Ascomycota; Eurotiomycetes; Eurotiales; Trichocomaceae; Paecilomyces; Paecilomyces_sp
New.ReferenceOTU709	0	0	0	0	0	0	0	0	0	0	0	0	0	0	0	0	0	0	0	0	0	0	0	1	0	0	0	0	0	0	Fungi; Ascomycota; Eurotiomycetes; Eurotiales; Trichocomaceae; Paecilomyces; Paecilomyces_sp
SH112362.06FU_JX155884_reps	0	0	0	0	0	0	0	0	0	0	0	0	0	0	0	0	0	0	0	0	0	0	0	0	0	0	0	3	0	0	Fungi; Ascomycota; Eurotiomycetes; Eurotiales; Trichocomaceae; Paecilomyces; Paecilomyces_sp
New.ReferenceOTU4426	0	0	0	0	0	0	0	0	0	0	0	0	0	0	0	0	1	0	0	0	0	0	0	0	2	0	0	0	0	0	Fungi; Ascomycota; Eurotiomycetes; Eurotiales; Trichocomaceae; Paecilomyces; Paecilomyces_sp_WSH_L07
New.CleanUp.ReferenceOTU2189	0	0	0	0	0	0	0	0	1	0	0	0	0	0	0	0	0	0	0	0	0	0	0	0	0	0	0	0	0	0	Fungi; Ascomycota; Eurotiomycetes; Eurotiales; Trichocomaceae; Paecilomyces; Paecilomyces_verrucosus
New.ReferenceOTU1100	0	0	2	0	0	0	1	1	0	0	1	0	1	0	5	4	3	0	1	0	1	2	0	0	0	0	0	0	0	0	Fungi; Ascomycota; Eurotiomycetes; Eurotiales; Trichocomaceae; Penicillium; Penicillium_abidjanum
New.ReferenceOTU4864	0	0	0	0	0	0	0	0	0	0	0	0	0	0	0	0	0	0	0	0	2	1	0	1	0	0	0	1	0	0	Fungi; Ascomycota; Eurotiomycetes; Eurotiales; Trichocomaceae; Penicillium; Penicillium_abidjanum
New.ReferenceOTU5945	0	0	1	0	0	0	0	0	0	0	0	0	0	0	4	1	2	0	0	0	1	2	0	0	0	0	0	0	0	0	Fungi; Ascomycota; Eurotiomycetes; Eurotiales; Trichocomaceae; Penicillium; Penicillium_abidjanum
New.ReferenceOTU3433	0	0	1	0	0	0	0	0	0	0	0	0	0	0	0	0	0	0	0	0	0	0	0	0	0	0	0	0	0	0	Fungi; Ascomycota; Eurotiomycetes; Eurotiales; Trichocomaceae; Penicillium; Penicillium_adametzii
SH113528.06FU_AF033401_refs	0	0	1	0	0	0	0	0	0	0	0	0	0	0	0	0	0	0	0	0	0	0	0	0	0	0	0	0	0	0	Fungi; Ascomycota; Eurotiomycetes; Eurotiales; Trichocomaceae; Penicillium; Penicillium_adametzii
New.ReferenceOTU5534	0	0	0	0	0	0	0	0	0	0	0	0	0	0	0	0	0	0	0	0	0	0	0	1	0	0	0	0	0	0	Fungi; Ascomycota; Eurotiomycetes; Eurotiales; Trichocomaceae; Penicillium; Penicillium_amaliae
SH113539.06FU_JX091443_refs	0	0	0	0	0	0	0	0	0	0	0	0	0	0	0	0	0	0	0	0	0	0	0	1	0	0	0	0	0	0	Fungi; Ascomycota; Eurotiomycetes; Eurotiales; Trichocomaceae; Penicillium; Penicillium_amaliae
New.ReferenceOTU6965	0	0	0	0	0	0	0	0	0	0	0	0	0	0	0	0	1	0	0	0	0	0	0	0	0	0	0	0	0	0	Fungi; Ascomycota; Eurotiomycetes; Eurotiales; Trichocomaceae; Penicillium; Penicillium_capsulatum
New.ReferenceOTU2629	0	0	0	0	0	0	0	0	0	0	0	0	0	0	0	0	0	0	0	0	0	1	0	0	0	0	0	0	0	0	Fungi; Ascomycota; Eurotiomycetes; Eurotiales; Trichocomaceae; Penicillium; Penicillium_citrinum
New.ReferenceOTU4437	0	0	0	0	0	0	0	0	0	0	0	0	0	0	0	1	0	0	0	0	0	0	0	0	0	0	0	0	0	0	Fungi; Ascomycota; Eurotiomycetes; Eurotiales; Trichocomaceae; Penicillium; Penicillium_citrinum
New.ReferenceOTU4631	0	0	0	0	0	0	0	0	0	0	0	0	0	0	0	0	1	0	0	0	0	0	0	0	0	0	0	0	0	0	Fungi; Ascomycota; Eurotiomycetes; Eurotiales; Trichocomaceae; Penicillium; Penicillium_citrinum
SH016774.06FU_KC485454_reps_singleton	0	0	0	0	0	2	0	1	0	0	0	0	6	0	0	1	4	0	0	0	0	0	0	1	0	0	0	1	0	0	Fungi; Ascomycota; Eurotiomycetes; Eurotiales; Trichocomaceae; Penicillium; Penicillium_citrinum
SH113520.06FU_AF033422_refs	2	0	3	0	2	12	1	2	0	3	1	2	4	20	4	7	33	0	0	0	4	5	4	0	6	1	0	2	0	0	Fungi; Ascomycota; Eurotiomycetes; Eurotiales; Trichocomaceae; Penicillium; Penicillium_citrinum
New.ReferenceOTU7942	0	0	0	0	0	0	0	0	0	0	0	0	0	0	0	0	0	0	0	0	0	0	2	0	0	0	0	0	0	1	Fungi; Ascomycota; Eurotiomycetes; Eurotiales; Trichocomaceae; Penicillium; Penicillium_commune
SH135164.06FU_AF033475_refs	0	0	0	0	0	0	0	0	0	0	0	1	0	0	0	0	0	0	0	0	0	0	0	0	0	0	0	0	0	0	Fungi; Ascomycota; Eurotiomycetes; Eurotiales; Trichocomaceae; Penicillium; Penicillium_concentricum
SH098803.06FU_JN617685_refs	0	0	0	0	0	0	0	0	0	0	0	0	0	0	0	0	2	0	0	0	0	1	0	0	0	0	0	0	0	0	Fungi; Ascomycota; Eurotiomycetes; Eurotiales; Trichocomaceae; Penicillium; Penicillium_copticola
New.ReferenceOTU366	0	0	0	0	0	1	0	0	0	0	0	0	0	0	0	0	0	0	0	0	0	0	0	0	0	0	0	0	0	0	Fungi; Ascomycota; Eurotiomycetes; Eurotiales; Trichocomaceae; Penicillium; Penicillium_daleae
SH098815.06FU_DQ187954_reps	0	0	0	0	0	0	0	0	0	0	0	0	0	0	0	0	0	0	0	0	0	1	0	0	0	0	0	0	0	0	Fungi; Ascomycota; Eurotiomycetes; Eurotiales; Trichocomaceae; Penicillium; Penicillium_daleae
New.ReferenceOTU5991	0	0	0	0	1	6	3	0	0	0	0	0	0	0	0	0	0	0	0	0	0	0	0	0	1	0	0	1	0	0	Fungi; Ascomycota; Eurotiomycetes; Eurotiales; Trichocomaceae; Penicillium; Penicillium_dierckxii
New.CleanUp.ReferenceOTU2119	0	0	0	0	0	0	0	1	0	0	0	0	0	0	0	0	0	0	0	0	0	0	0	0	0	0	0	1	0	0	Fungi; Ascomycota; Eurotiomycetes; Eurotiales; Trichocomaceae; Penicillium; Penicillium_erubescens
New.ReferenceOTU1003	1	0	7	0	0	12	2	70	3	1	1	2	0	0	0	1	9	0	1	0	3	0	9	1	2	0	10	0	0	0	Fungi; Ascomycota; Eurotiomycetes; Eurotiales; Trichocomaceae; Penicillium; Penicillium_erubescens
New.ReferenceOTU1044	0	0	0	2	0	3	2	23	0	0	0	3	2	1	0	0	10	0	1	0	1	0	4	0	0	0	3	4	0	0	Fungi; Ascomycota; Eurotiomycetes; Eurotiales; Trichocomaceae; Penicillium; Penicillium_erubescens
New.ReferenceOTU1260	0	0	1	0	0	0	0	0	0	0	0	0	0	0	0	0	0	0	0	0	0	0	0	0	0	0	0	0	0	0	Fungi; Ascomycota; Eurotiomycetes; Eurotiales; Trichocomaceae; Penicillium; Penicillium_erubescens
New.ReferenceOTU1469	0	0	0	0	0	0	0	2	0	0	0	0	0	0	0	0	0	0	0	0	0	0	0	0	0	0	0	0	0	0	Fungi; Ascomycota; Eurotiomycetes; Eurotiales; Trichocomaceae; Penicillium; Penicillium_erubescens
New.ReferenceOTU1939	0	0	0	0	0	1	0	4	0	0	0	0	1	0	0	0	1	0	0	0	0	0	0	0	0	0	0	1	0	0	Fungi; Ascomycota; Eurotiomycetes; Eurotiales; Trichocomaceae; Penicillium; Penicillium_erubescens
New.ReferenceOTU2019	0	0	0	0	0	0	0	4	0	0	0	0	0	0	0	0	0	0	0	0	0	0	0	0	0	0	0	0	0	0	Fungi; Ascomycota; Eurotiomycetes; Eurotiales; Trichocomaceae; Penicillium; Penicillium_erubescens
New.ReferenceOTU219	0	0	0	0	0	0	0	2	0	0	0	0	0	0	0	0	0	0	0	0	0	0	0	0	0	0	0	0	0	0	Fungi; Ascomycota; Eurotiomycetes; Eurotiales; Trichocomaceae; Penicillium; Penicillium_erubescens
New.ReferenceOTU2649	0	0	0	0	0	0	0	3	0	0	0	0	0	0	0	0	0	0	0	0	0	0	0	0	0	0	0	0	0	0	Fungi; Ascomycota; Eurotiomycetes; Eurotiales; Trichocomaceae; Penicillium; Penicillium_erubescens
New.ReferenceOTU2783	0	0	0	0	0	0	0	2	0	0	0	0	0	0	0	0	1	0	0	0	0	0	0	1	0	0	0	0	0	0	Fungi; Ascomycota; Eurotiomycetes; Eurotiales; Trichocomaceae; Penicillium; Penicillium_erubescens
New.ReferenceOTU3219	0	0	0	0	0	0	0	18	0	0	0	0	0	0	0	0	0	0	0	0	0	0	0	0	0	0	0	0	0	0	Fungi; Ascomycota; Eurotiomycetes; Eurotiales; Trichocomaceae; Penicillium; Penicillium_erubescens
New.ReferenceOTU3357	0	0	1	0	0	0	0	1	0	0	0	1	0	0	0	0	0	0	0	0	0	0	2	0	0	0	0	0	0	0	Fungi; Ascomycota; Eurotiomycetes; Eurotiales; Trichocomaceae; Penicillium; Penicillium_erubescens
New.ReferenceOTU3526	0	0	2	1	0	0	1	2	0	1	0	1	2	0	0	0	5	0	0	0	0	0	4	0	0	0	1	0	0	0	Fungi; Ascomycota; Eurotiomycetes; Eurotiales; Trichocomaceae; Penicillium; Penicillium_erubescens
New.ReferenceOTU3624	0	0	0	0	0	0	0	0	0	0	0	0	0	0	0	0	0	0	0	0	0	0	0	0	0	0	0	1	0	0	Fungi; Ascomycota; Eurotiomycetes; Eurotiales; Trichocomaceae; Penicillium; Penicillium_erubescens
New.ReferenceOTU3720	0	0	0	0	0	0	0	1	0	0	0	0	0	0	0	0	0	0	0	0	0	0	0	0	0	0	0	0	0	0	Fungi; Ascomycota; Eurotiomycetes; Eurotiales; Trichocomaceae; Penicillium; Penicillium_erubescens
New.ReferenceOTU3803	0	0	1	0	0	0	0	1	0	0	0	0	0	0	0	0	0	0	0	0	0	0	0	0	0	0	1	0	0	0	Fungi; Ascomycota; Eurotiomycetes; Eurotiales; Trichocomaceae; Penicillium; Penicillium_erubescens
New.ReferenceOTU4190	0	0	0	0	0	0	0	2	0	0	0	0	0	0	0	0	0	0	0	0	0	0	0	0	0	0	0	0	0	0	Fungi; Ascomycota; Eurotiomycetes; Eurotiales; Trichocomaceae; Penicillium; Penicillium_erubescens
New.ReferenceOTU4192	0	0	0	0	1	0	0	2	0	0	0	0	0	0	0	0	0	0	0	0	0	0	0	0	0	0	0	0	0	0	Fungi; Ascomycota; Eurotiomycetes; Eurotiales; Trichocomaceae; Penicillium; Penicillium_erubescens
New.ReferenceOTU4290	0	0	0	0	0	0	0	1	0	0	0	0	0	0	0	0	0	0	0	0	0	0	0	0	0	0	0	0	0	0	Fungi; Ascomycota; Eurotiomycetes; Eurotiales; Trichocomaceae; Penicillium; Penicillium_erubescens
New.ReferenceOTU436	0	0	0	1	0	1	0	1	0	0	0	0	0	0	0	0	0	0	0	0	0	0	0	0	0	0	0	0	0	0	Fungi; Ascomycota; Eurotiomycetes; Eurotiales; Trichocomaceae; Penicillium; Penicillium_erubescens
New.ReferenceOTU5032	0	0	0	0	0	0	0	2	0	0	0	0	0	0	0	0	0	0	0	0	0	0	0	0	0	0	0	0	0	0	Fungi; Ascomycota; Eurotiomycetes; Eurotiales; Trichocomaceae; Penicillium; Penicillium_erubescens
New.ReferenceOTU5240	0	0	0	0	0	0	0	0	0	0	0	0	0	0	0	0	0	0	0	0	0	0	2	0	0	0	1	0	0	0	Fungi; Ascomycota; Eurotiomycetes; Eurotiales; Trichocomaceae; Penicillium; Penicillium_erubescens
New.ReferenceOTU5320	0	0	0	0	0	0	0	2	0	0	0	0	0	0	0	0	0	0	0	0	0	0	0	0	0	0	0	0	0	0	Fungi; Ascomycota; Eurotiomycetes; Eurotiales; Trichocomaceae; Penicillium; Penicillium_erubescens
New.ReferenceOTU5514	0	0	0	0	0	0	0	1	0	0	0	0	0	0	0	0	0	0	0	0	0	0	0	0	0	0	0	0	0	0	Fungi; Ascomycota; Eurotiomycetes; Eurotiales; Trichocomaceae; Penicillium; Penicillium_erubescens
New.ReferenceOTU6478	0	0	0	0	0	0	0	1	0	0	0	0	0	0	0	0	0	0	0	0	0	0	0	0	0	0	0	0	0	0	Fungi; Ascomycota; Eurotiomycetes; Eurotiales; Trichocomaceae; Penicillium; Penicillium_erubescens
New.ReferenceOTU7351	0	0	0	0	0	0	0	2	0	0	0	0	0	0	0	0	0	0	0	0	0	0	0	0	0	0	0	0	0	0	Fungi; Ascomycota; Eurotiomycetes; Eurotiales; Trichocomaceae; Penicillium; Penicillium_erubescens
SH002845.06FU_JQ898688_reps_singleton	0	0	0	0	0	0	0	2	1	0	0	0	0	0	0	0	1	0	0	0	1	0	1	0	2	0	2	0	0	0	Fungi; Ascomycota; Eurotiomycetes; Eurotiales; Trichocomaceae; Penicillium; Penicillium_erubescens
SH113524.06FU_AF037431_refs	1	0	1	3	3	27	9	52	3	3	5	23	1	60	4	0	52	0	0	1	3	0	13	4	1	0	16	45	0	2	Fungi; Ascomycota; Eurotiomycetes; Eurotiales; Trichocomaceae; Penicillium; Penicillium_erubescens
SH113586.06FU_AF033464_refs	0	0	6	1	0	1	5	21	1	0	0	10	3	2	0	1	30	0	0	0	4	0	54	0	1	0	8	12	0	1	Fungi; Ascomycota; Eurotiomycetes; Eurotiales; Trichocomaceae; Penicillium; Penicillium_erubescens
SH008171.06FU_FJ861426_reps_singleton	0	0	1	1	0	0	0	0	0	0	0	0	0	0	0	0	0	0	0	0	0	0	0	0	0	0	0	0	0	0	Fungi; Ascomycota; Eurotiomycetes; Eurotiales; Trichocomaceae; Penicillium; Penicillium_herquei
SH098801.06FU_JN626101_refs	0	0	0	0	1	0	0	0	0	0	1	0	0	0	0	0	0	0	0	0	0	0	0	0	0	0	0	1	0	0	Fungi; Ascomycota; Eurotiomycetes; Eurotiales; Trichocomaceae; Penicillium; Penicillium_herquei
New.ReferenceOTU7757	0	0	0	0	0	0	0	0	0	0	0	0	0	0	1	0	0	0	0	0	0	0	0	0	0	0	0	0	0	0	Fungi; Ascomycota; Eurotiomycetes; Eurotiales; Trichocomaceae; Penicillium; Penicillium_implicatum
New.ReferenceOTU3321	0	0	0	0	0	0	0	0	0	0	0	0	0	1	0	0	0	0	0	0	0	0	0	0	0	0	0	0	0	0	Fungi; Ascomycota; Eurotiomycetes; Eurotiales; Trichocomaceae; Penicillium; Penicillium_inflatum
New.ReferenceOTU5561	0	0	0	0	0	0	0	0	0	0	0	0	0	1	0	0	0	0	2	2	0	0	0	0	0	0	0	0	0	0	Fungi; Ascomycota; Eurotiomycetes; Eurotiales; Trichocomaceae; Penicillium; Penicillium_inflatum
New.CleanUp.ReferenceOTU1553	0	0	0	0	0	1	3	1	0	0	0	0	0	0	0	0	0	0	0	0	0	0	0	0	0	0	0	0	0	0	Fungi; Ascomycota; Eurotiomycetes; Eurotiales; Trichocomaceae; Penicillium; Penicillium_inusitatum
New.ReferenceOTU1	0	0	0	0	0	1	0	0	0	1	0	0	0	0	0	0	0	0	0	0	0	0	0	0	0	0	0	0	0	0	Fungi; Ascomycota; Eurotiomycetes; Eurotiales; Trichocomaceae; Penicillium; Penicillium_inusitatum
New.ReferenceOTU3290	0	0	0	0	0	0	2	0	0	0	0	0	0	0	0	0	0	0	0	0	0	0	0	0	0	0	0	0	0	0	Fungi; Ascomycota; Eurotiomycetes; Eurotiales; Trichocomaceae; Penicillium; Penicillium_inusitatum
New.ReferenceOTU3350	0	0	0	0	1	1	3	0	1	0	0	0	1	0	0	1	0	0	0	0	0	0	0	0	0	0	0	0	0	0	Fungi; Ascomycota; Eurotiomycetes; Eurotiales; Trichocomaceae; Penicillium; Penicillium_inusitatum
New.ReferenceOTU5744	0	0	0	0	0	0	1	0	0	0	0	1	0	0	0	0	0	0	0	0	0	0	0	0	0	0	0	0	0	0	Fungi; Ascomycota; Eurotiomycetes; Eurotiales; Trichocomaceae; Penicillium; Penicillium_inusitatum
New.ReferenceOTU7502	0	0	0	0	0	0	0	0	0	0	0	0	2	0	0	0	0	0	0	0	0	0	0	0	0	0	0	0	0	0	Fungi; Ascomycota; Eurotiomycetes; Eurotiales; Trichocomaceae; Penicillium; Penicillium_levitum
SH098830.06FU_JN626097_refs	0	0	0	0	1	0	0	0	0	0	0	0	0	0	0	0	0	0	0	0	0	1	0	0	0	0	0	0	0	0	Fungi; Ascomycota; Eurotiomycetes; Eurotiales; Trichocomaceae; Penicillium; Penicillium_levitum
New.ReferenceOTU2600	0	0	0	0	0	1	0	0	0	0	0	0	0	0	0	0	0	0	0	0	0	0	0	0	0	0	0	0	0	0	Fungi; Ascomycota; Eurotiomycetes; Eurotiales; Trichocomaceae; Penicillium; Penicillium_madriti
New.ReferenceOTU2735	0	0	0	0	0	0	2	2	1	3	0	2	0	0	0	0	0	0	0	0	0	0	0	0	0	0	3	0	0	0	Fungi; Ascomycota; Eurotiomycetes; Eurotiales; Trichocomaceae; Penicillium; Penicillium_madriti

New.ReferenceOTU299	0	0	0	0	0	0	0	0	0	0	0	0	0	0	0	0	0	0	0	0	0	0	0	0	0	0	0	0	0	3	Fungi; Ascomycota; Eurotiomycetes; Eurotiales; Trichocomaceae; Penicillium; Penicillium_madriti
New.ReferenceOTU3552	0	0	1	0	0	1	0	0	0	1	1	0	0	0	0	0	1	0	0	0	9	2	0	0	0	0	0	0	0	0	Fungi; Ascomycota; Eurotiomycetes; Eurotiales; Trichocomaceae; Penicillium; Penicillium_madriti
New.ReferenceOTU4080	0	0	0	0	0	0	0	0	0	0	1	0	0	1	0	0	0	1	0	0	0	0	1	0	0	0	0	0	0	2	Fungi; Ascomycota; Eurotiomycetes; Eurotiales; Trichocomaceae; Penicillium; Penicillium_madriti
New.ReferenceOTU4391	0	0	0	0	0	0	0	0	0	0	0	0	0	0	0	0	0	0	0	0	0	0	0	0	0	2	0	0	0	0	Fungi; Ascomycota; Eurotiomycetes; Eurotiales; Trichocomaceae; Penicillium; Penicillium_malacaense
New.ReferenceOTU5148	0	0	0	0	0	0	0	0	0	0	0	0	0	0	0	0	0	0	0	0	0	0	0	0	0	0	3	0	0	0	Fungi; Ascomycota; Eurotiomycetes; Eurotiales; Trichocomaceae; Penicillium; Penicillium_malacaense
SH098793.06FU_JN626104_refs	0	0	6	2	4	1	0	0	0	0	0	0	0	0	1	0	2	0	0	0	0	4	0	1	0	0	0	0	0	0	Fungi; Ascomycota; Eurotiomycetes; Eurotiales; Trichocomaceae; Penicillium; Penicillium_mallochii
SH098843.06FU_KF302657_reps	0	0	1	0	0	0	0	0	0	0	0	0	0	0	0	0	0	0	0	0	0	0	0	0	0	0	0	0	0	0	Fungi; Ascomycota; Eurotiomycetes; Eurotiales; Trichocomaceae; Penicillium; Penicillium_mallochii
New.ReferenceOTU3344	0	0	0	0	0	0	0	0	0	0	0	0	0	1	0	0	0	0	0	0	0	0	0	0	0	0	0	0	0	0	Fungi; Ascomycota; Eurotiomycetes; Eurotiales; Trichocomaceae; Penicillium; Penicillium_maximae
New.ReferenceOTU4148	0	0	2	2	1	0	2	1	0	3	1	0	0	0	0	0	2	1	0	1	0	0	0	1	1	0	0	0	3	1	Fungi; Ascomycota; Eurotiomycetes; Eurotiales; Trichocomaceae; Penicillium; Penicillium_maximae
SH098798.06FU_EU427298_refs	0	0	1	10	0	0	0	2	0	4	2	0	1	4	3	1	8	1	0	1	0	2	0	0	0	0	0	0	0	1	Fungi; Ascomycota; Eurotiomycetes; Eurotiales; Trichocomaceae; Penicillium; Penicillium_maximae
SH089798.06FU_AF033451_refs	0	0	0	0	0	0	0	0	0	0	0	0	0	0	0	0	0	0	0	0	0	0	1	0	0	0	0	0	0	0	Fungi; Ascomycota; Eurotiomycetes; Eurotiales; Trichocomaceae; Penicillium; Penicillium_meridianum
New.ReferenceOTU5203	0	0	0	0	0	0	0	0	0	0	0	0	0	0	0	0	0	0	0	0	0	0	4	0	0	0	0	0	0	0	Fungi; Ascomycota; Eurotiomycetes; Eurotiales; Trichocomaceae; Penicillium; Penicillium_minioluteum
New.ReferenceOTU7730	0	0	0	0	0	0	0	0	0	0	3	0	0	0	0	0	0	0	0	0	0	0	1	0	0	0	0	0	0	0	Fungi; Ascomycota; Eurotiomycetes; Eurotiales; Trichocomaceae; Penicillium; Penicillium_minioluteum
SH128757.06FU_FR670332_reps	0	0	0	0	0	0	0	0	0	0	0	0	0	0	0	0	0	0	0	0	0	0	1	0	0	0	0	0	0	0	Fungi; Ascomycota; Eurotiomycetes; Eurotiales; Trichocomaceae; Penicillium; Penicillium_minioluteum
New.ReferenceOTU1295	0	0	0	0	1	2	1	2	0	1	3	0	0	0	2	0	0	0	0	1	0	0	0	0	0	0	1	0	1	1	Fungi; Ascomycota; Eurotiomycetes; Eurotiales; Trichocomaceae; Penicillium; Penicillium_novae-zeelandiae
New.ReferenceOTU5247	0	0	0	0	0	0	0	0	0	0	0	0	0	0	0	4	0	0	0	0	0	9	0	0	0	0	0	0	0	0	Fungi; Ascomycota; Eurotiomycetes; Eurotiales; Trichocomaceae; Penicillium; Penicillium_onobense
New.ReferenceOTU3273	0	0	0	0	0	0	0	0	0	0	0	1	0	0	0	0	0	0	0	0	0	0	1	0	0	0	0	0	0	0	Fungi; Ascomycota; Eurotiomycetes; Eurotiales; Trichocomaceae; Penicillium; Penicillium_ornatum
New.ReferenceOTU442	0	0	0	0	0	0	0	0	0	0	0	0	0	0	0	1	0	0	0	0	0	0	3	0	0	0	0	0	0	0	Fungi; Ascomycota; Eurotiomycetes; Eurotiales; Trichocomaceae; Penicillium; Penicillium_ornatum
New.ReferenceOTU6928	0	0	0	0	0	0	0	0	0	0	0	0	0	0	0	0	0	0	0	0	0	0	1	0	0	0	0	0	0	0	Fungi; Ascomycota; Eurotiomycetes; Eurotiales; Trichocomaceae; Penicillium; Penicillium_ornatum
SH030560.06FU_JX841244_reps_singleton	0	0	1	0	0	0	0	0	1	1	1	0	0	16	0	0	1	0	0	1	0	0	40	0	0	0	1	0	0	0	Fungi; Ascomycota; Eurotiomycetes; Eurotiales; Trichocomaceae; Penicillium; Penicillium_ornatum
New.CleanUp.ReferenceOTU847	0	0	0	0	0	0	0	0	0	0	0	0	0	0	0	0	0	0	0	0	0	0	0	0	0	0	0	0	0	1	Fungi; Ascomycota; Eurotiomycetes; Eurotiales; Trichocomaceae; Penicillium; Penicillium_oxalicum
SH098791.06FU_AF033438_refs	0	0	0	0	0	0	0	0	0	0	0	0	0	0	0	0	0	0	0	0	0	0	0	0	0	0	0	0	0	3	Fungi; Ascomycota; Eurotiomycetes; Eurotiales; Trichocomaceae; Penicillium; Penicillium_oxalicum
New.ReferenceOTU1450	0	0	3	2	0	1	3	0	0	1	0	1	0	0	0	0	1	0	0	0	1	0	0	0	0	0	0	0	0	0	Fungi; Ascomycota; Eurotiomycetes; Eurotiales; Trichocomaceae; Penicillium; Penicillium_paxilli
New.ReferenceOTU2732	0	0	1	1	0	1	0	1	0	0	0	0	0	0	0	0	4	1	0	0	0	0	0	0	0	0	0	0	0	0	Fungi; Ascomycota; Eurotiomycetes; Eurotiales; Trichocomaceae; Penicillium; Penicillium_paxilli
New.ReferenceOTU5274	0	0	0	0	0	0	0	0	0	0	0	0	0	0	0	1	0	0	0	0	0	0	0	0	0	0	0	0	0	0	Fungi; Ascomycota; Eurotiomycetes; Eurotiales; Trichocomaceae; Penicillium; Penicillium_paxilli
New.ReferenceOTU6168	0	0	0	0	0	0	0	0	0	1	0	0	0	0	0	0	0	0	0	0	0	0	0	0	0	0	0	0	0	0	Fungi; Ascomycota; Eurotiomycetes; Eurotiales; Trichocomaceae; Penicillium; Penicillium_paxilli
SH098805.06FU_GU944577_refs	0	0	8	4	0	0	0	0	0	0	1	0	0	4	0	4	4	0	2	0	0	0	1	1	0	0	0	0	0	0	Fungi; Ascomycota; Eurotiomycetes; Eurotiales; Trichocomaceae; Penicillium; Penicillium_paxilli
New.ReferenceOTU250	0	0	0	0	0	0	0	1	0	0	0	0	0	0	0	0	0	0	0	0	0	0	0	0	0	0	0	0	0	0	Fungi; Ascomycota; Eurotiomycetes; Eurotiales; Trichocomaceae; Penicillium; Penicillium_pimiteouiense
New.ReferenceOTU3303	0	0	0	0	0	0	0	0	0	0	0	0	0	0	0	0	0	0	1	0	0	0	0	0	0	0	0	0	0	0	Fungi; Ascomycota; Eurotiomycetes; Eurotiales; Trichocomaceae; Penicillium; Penicillium_pimiteouiense
New.ReferenceOTU4090	0	0	0	0	0	0	0	1	0	0	0	0	0	0	0	0	0	0	0	0	0	0	0	0	0	0	0	0	0	0	Fungi; Ascomycota; Eurotiomycetes; Eurotiales; Trichocomaceae; Penicillium; Penicillium_pimiteouiense
SH128748.06FU_JN899333_refs	0	0	5	0	0	3	0	0	0	1	0	1	0	0	0	0	2	0	0	0	1	0	0	2	0	2	1	0	0	0	Fungi; Ascomycota; Eurotiomycetes; Eurotiales; Trichocomaceae; Penicillium; Penicillium_pinophilum
SH128785.06FU_JN899341_refs	0	0	0	0	0	0	0	0	0	0	0	0	0	0	0	0	0	1	0	0	0	0	0	0	0	0	0	0	0	0	Fungi; Ascomycota; Eurotiomycetes; Eurotiales; Trichocomaceae; Penicillium; Penicillium_pinophilum
New.ReferenceOTU4995	0	0	1	0	0	0	0	0	0	0	0	0	0	0	3	0	0	0	0	0	0	0	1	0	0	0	0	1	0	0	Fungi; Ascomycota; Eurotiomycetes; Eurotiales; Trichocomaceae; Penicillium; Penicillium_rolfsii
New.ReferenceOTU3040	0	2	0	1	0	1	0	0	2	0	0	0	1	0	0	0	0	0	0	0	0	1	0	0	0	0	0	0	0	0	Fungi; Ascomycota; Eurotiomycetes; Eurotiales; Trichocomaceae; Penicillium; Penicillium_sclerotigenum
New.ReferenceOTU7036	0	0	0	0	0	0	0	0	0	0	0	0	0	0	0	0	1	0	0	0	0	0	0	0	0	0	0	0	0	0	Fungi; Ascomycota; Eurotiomycetes; Eurotiales; Trichocomaceae; Penicillium; Penicillium_sclerotiorum
SH098802.06FU_AY373930_refs	0	0	0	0	2	0	0	0	0	0	0	1	0	1	2	0	0	0	0	0	0	0	0	0	0	0	0	0	0	0	Fungi; Ascomycota; Eurotiomycetes; Eurotiales; Trichocomaceae; Penicillium; Penicillium_sclerotiorum
New.CleanUp.ReferenceOTU1003	0	0	0	0	0	0	0	0	0	0	0	0	0	0	0	0	0	0	0	0	0	0	1	0	0	0	0	0	0	0	Fungi; Ascomycota; Eurotiomycetes; Eurotiales; Trichocomaceae; Penicillium; Penicillium_simplicissimum
New.CleanUp.ReferenceOTU548	0	0	0	0	0	0	0	0	0	0	1	0	0	0	0	0	0	0	0	0	0	0	0	0	0	0	0	0	0	0	Fungi; Ascomycota; Eurotiomycetes; Eurotiales; Trichocomaceae; Penicillium; Penicillium_simplicissimum
New.ReferenceOTU121	0	0	0	0	1	1	0	0	0	0	0	0	0	0	0	0	0	0	0	0	0	0	0	0	0	0	0	0	0	0	Fungi; Ascomycota; Eurotiomycetes; Eurotiales; Trichocomaceae; Penicillium; Penicillium_simplicissimum
New.ReferenceOTU1584	0	0	1	0	0	0	0	0	0	0	0	0	0	0	0	0	0	0	0	0	0	0	0	0	0	0	0	1	0	0	Fungi; Ascomycota; Eurotiomycetes; Eurotiales; Trichocomaceae; Penicillium; Penicillium_simplicissimum
New.ReferenceOTU1813	0	0	0	0	0	0	0	0	0	0	0	0	1	0	0	0	0	0	0	0	0	0	0	0	0	0	0	0	0	0	Fungi; Ascomycota; Eurotiomycetes; Eurotiales; Trichocomaceae; Penicillium; Penicillium_simplicissimum
New.ReferenceOTU2308	0	0	0	0	0	0	0	0	0	0	0	0	0	0	0	0	0	0	0	0	0	1	0	0	0	0	1	0	0	0	Fungi; Ascomycota; Eurotiomycetes; Eurotiales; Trichocomaceae; Penicillium; Penicillium_simplicissimum
New.ReferenceOTU2908	0	0	0	1	0	1	0	0	0	0	0	0	0	0	0	0	0	0	0	0	0	0	0	0	0	0	0	0	0	0	Fungi; Ascomycota; Eurotiomycetes; Eurotiales; Trichocomaceae; Penicillium; Penicillium_simplicissimum
New.ReferenceOTU3090	0	0	0	0	0	0	1	0	0	0	0	1	0	0	1	0	0	0	0	0	0	0	0	0	0	0	1	0	0	0	Fungi; Ascomycota; Eurotiomycetes; Eurotiales; Trichocomaceae; Penicillium; Penicillium_simplicissimum
New.ReferenceOTU336	0	0	0	0	1	0	0	0	0	0	0	0	0	0	0	0	0	0	0	0	0	0	0	0	0	0	0	0	0	0	Fungi; Ascomycota; Eurotiomycetes; Eurotiales; Trichocomaceae; Penicillium; Penicillium_simplicissimum
New.ReferenceOTU3842	0	0	0	0	0	0	0	1	0	0	0	0	0	0	0	0	0	0	0	0	0	0	0	0	0	0	0	0	0	0	Fungi; Ascomycota; Eurotiomycetes; Eurotiales; Trichocomaceae; Penicillium; Penicillium_simplicissimum
New.ReferenceOTU3929	0	0	0	0	0	0	0	0	0	0	0	0	0	0	0	1	0	1	0	0	0	0	0	0	0	0	0	0	0	0	Fungi; Ascomycota; Eurotiomycetes; Eurotiales; Trichocomaceae; Penicillium; Penicillium_simplicissimum
New.ReferenceOTU4218	0	0	0	0	0	0	0	0	0	0	0	1	0	0	0	0	0	0	0	0	0	0	0	0	0	0	0	0	0	0	Fungi; Ascomycota; Eurotiomycetes; Eurotiales; Trichocomaceae; Penicillium; Penicillium_simplicissimum
New.ReferenceOTU4537	0	0	0	0	2	0	0	0	0	0	0	0	0	0	0	0	0	0	0	0	0	0	0	0	0	0	0	0	0	0	Fungi; Ascomycota; Eurotiomycetes; Eurotiales; Trichocomaceae; Penicillium; Penicillium_simplicissimum
New.ReferenceOTU4880	0	0	1	0	0	0	0	0	0	0	0	0	0	0	0	0	0	0	0	0	0	0	1	0	0	0	0	0	0	0	Fungi; Ascomycota; Eurotiomycetes; Eurotiales; Trichocomaceae; Penicillium; Penicillium_simplicissimum
SH098797.06FU_HQ850334_reps	0	0	0	0	0	0	0	0	0	0	0	0	0	0	0	0	0	0	0	0	1	0	0	0	0	0	0	0	0	0	Fungi; Ascomycota; Eurotiomycetes; Eurotiales; Trichocomaceae; Penicillium; Penicillium_simplicissimum
SH098829.06FU_JN861764_reps	0	0	1	0	1	0	0	0	0	0	0	0	0	0	0	0	0	0	0	0	0	0	0	0	0	0	0	0	0	0	Fungi; Ascomycota; Eurotiomycetes; Eurotiales; Trichocomaceae; Penicillium; Penicillium_simplicissimum
SH098833.06FU_GU212865_reps	0	0	0	0	0	0	0	0	0	0	0	1	0	3	0	0	0	0	0	0	0	0	0	0	0	0	0	0	0	0	Fungi; Ascomycota; Eurotiomycetes; Eurotiales; Trichocomaceae; Penicillium; Penicillium_simplicissimum
New.CleanUp.ReferenceOTU1413	0	0	0	0	0	0	0	0	0	0	0	0	0	0	0	0	0	0	0	1	0	0	0	0	0	0	0	0	0	0	Fungi; Ascomycota; Eurotiomycetes; Eurotiales; Trichocomaceae; Penicillium; Penicillium_sp
New.CleanUp.ReferenceOTU1794	0	0	2	0	0	0	0	0	0	0	0	0	0	0	0	0	3	0	0	0	0	0	0	0	0	0	0	0	0	0	Fungi; Ascomycota; Eurotiomycetes; Eurotiales; Trichocomaceae; Penicillium; Penicillium_sp
New.CleanUp.ReferenceOTU1906	0	0	0	0	0	2	0	0	1	0	0	1	0	0	0	1	0	2	0	0	0	1	0	1	0	0	0	0	0	0	Fungi; Ascomycota; Eurotiomycetes; Eurotiales; Trichocomaceae; Penicillium; Penicillium_sp
New.CleanUp.ReferenceOTU1938	0	0	0	0	0	0	0	0	0	0	0	0	0	0	0	1	0	0	0	0	0	0	0	0	0	0	0	0	0	0	Fungi; Ascomycota; Eurotiomycetes; Eurotiales; Trichocomaceae; Penicillium; Penicillium_sp
New.ReferenceOTU115	0	0	0	0	0	0	0	0	0	0	0	0	0	1	1	1	0	8	0	4	0	0	0	1	0	0	0	0	0	0	Fungi; Ascomycota; Eurotiomycetes; Eurotiales; Trichocomaceae; Penicillium; Penicillium_sp
New.ReferenceOTU1547	0	0	0	0	0	1	0	0	0	0	0	0	1	0	0	0	0	0	0	0	0	0	1	0	0	0	0	0	0	1	Fungi; Ascomycota; Eurotiomycetes; Eurotiales; Trichocomaceae; Penicillium; Penicillium_sp
New.ReferenceOTU1920	0	0	0	0	0	0	0	0	0	0	2	1	0	0	0	0	0	0	0	0	0	0	0	0	0	0	0	0	0	0	Fungi; Ascomycota; Eurotiomycetes; Eurotiales; Trichocomaceae; Penicillium; Penicillium_sp
New.ReferenceOTU2776	0	5	0	0	0	1	0	0	0	0	0	0	0	0	0	0	0	0	0	0	0	0	0	4	0	59	0	0	24	0	Fungi; Ascomycota; Eurotiomycetes; Eurotiales; Trichocomaceae; Penicillium; Penicillium_sp
New.ReferenceOTU3990	0	0	1	0	0	0	0	0	0	0	0	0	0	0	0	0	0	0	0	0	0	0	0	0	0	0	0	0	0	0	Fungi; Ascomycota; Eurotiomycetes; Eurotiales; Trichocomaceae; Penicillium; Penicillium_sp
New.ReferenceOTU4203	0	1	1	0	0	0	0	0	0	0	0	0	0	1	0	1	3	0	0	0	1	1	0	0	0	0	0	0	0	0	Fungi; Ascomycota; Eurotiomycetes; Eurotiales; Trichocomaceae; Penicillium; Penicillium_sp
New.ReferenceOTU4611	0	0	0	0	0	0	0	0	0	0	1	0	0	0	0	0	0	0	0	0	0	0	0	0	0	0	0	0	0	0	Fungi; Ascomycota; Eurotiomycetes; Eurotiales; Trichocomaceae; Penicillium; Penicillium_sp
New.ReferenceOTU4797	0	8	0	0	0	0	0	0	0	0	0	0	0	0	0	0	0	0	0	0	0	0	0	0	0	0	0	0	9	0	Fungi; Ascomycota; Eurotiomycetes; Eurotiales; Trichocomaceae; Penicillium; Penicillium_sp
New.ReferenceOTU5308	0	0	0	0	0	1	0	0	0	0	0	0	0	0	0	0	0	0	0	0	0	0	0	0	0	0	0	0	0	0	Fungi; Ascomycota; Eurotiomycetes; Eurotiales; Trichocomaceae; Penicillium; Penicillium_sp
New.ReferenceOTU5652	0	0	0	0	0	0	0	0	0	0	0	0	0	0	0	0	0	1	0	0	0	0	0	0	0	0	0	0	0	0	Fungi; Ascomycota; Eurotiomycetes; Eurotiales; Trichocomaceae; Penicillium; Penicillium_sp
New.ReferenceOTU6795	1	0	0	0	0	0	0	0	0	0	0	0	0	0	0	0	0	0	0	0	0	0	0	0	0	0	0	0	0	0	Fungi; Ascomycota; Eurotiomycetes; Eurotiales; Trichocomaceae; Penicillium; Penicillium_sp
New.ReferenceOTU7812	0	0	0	0	0	1	0	0	0	0	0	0	3	0	0	0	0	0	0	0	0	0	1	0	1	0	0	0	0	0	Fungi; Ascomycota; Eurotiomycetes; Eurotiales; Trichocomaceae; Penicillium; Penicillium_sp
New.ReferenceOTU8011	0	0	2	0	0	3	1	0	0	0	0	0	0	2	5	1	17	0	0	2	16	2	0	0	0	0	0	1	0	0	Fungi; Ascomycota; Eurotiomycetes; Eurotiales; Trichocomaceae; Penicillium; Penicillium_sp
New.ReferenceOTU845	0	1	0	0	0	0	1	0	0	0	0	0	0	0	0	1	0	0	0	0	0	1	1	0	0	0	0	0	0	0	Fungi; Ascomycota; Eurotiomycetes; Eurotiales; Trichocomaceae; Penicillium; Penicillium_sp
SH008160.06FU_AF178515_reps_singleton	0	0	0	0	0	0	0	0	0	0	0	0	0	0	0	0	0	0	0	0	0	1	0	0	0	0	0	0	0	0	Fungi; Ascomycota; Eurotiomycetes; Eurotiales; Trichocomaceae; Penicillium; Penicillium_sp
SH008174.06FU_JX156049_reps_singleton	0	0	0	0	0	1	0	0	0	0	0	0	0	0	0	0	0	0	0	0	0	0	0	0	0	0	0	0	1	0	Fungi; Ascomycota; Eurotiomycetes; Eurotiales; Trichocomaceae; Penicillium; Penicillium_sp
SH098792.06FU_JN617705_refs	0	0	1	0	7	1	0	0	4	1	0	1	0	0	2	0	0	1	0	0	2	2	0	0	1	0	0	2	0	1	Fungi; Ascomycota; Eurotiomycetes; Eurotiales; Trichocomaceae; Penicillium; Penicillium_sp
SH098819.06FU_JX155992_reps	2	3	4	1	4	22	0	3	1	0	0	10	12	3	3	1	4	1	0	1	1	7	3	1	7	0	0	6	0	5	Fungi; Ascomycota; Eurotiomycetes; Eurotiales; Trichocomaceae; Penicillium; Penicillium_sp
SH113536.06FU_HM239866_reps	0	0	2	0	0	0	0	0	0	0	0	0	0	1	0	0	0	2	0	3	0	0	0	0	0	0	0	0	0	0	Fungi; Ascomycota; Eurotiomycetes; Eurotiales; Trichocomaceae; Penicillium; Penicillium_sp
New.CleanUp.ReferenceOTU1311	0	0	0	0	0	0	0	0	0	0	0	1	0	0	0	0	0	0	0	0	0	0	0	0	0	0	0	0	0	0	Fungi; Ascomycota; Eurotiomycetes; Eurotiales; Trichocomaceae; Penicillium; Penicillium_sp_0410ARD6F_2
New.ReferenceOTU2835	0	0	0	0	0	0	0	0	0	0	0	0	0	0	0	0	0	0	0	0	0	0	0	0	0	0	1	0	0	0	Fungi; Ascomycota; Eurotiomycetes; Eurotiales; Trichocomaceae; Penicillium; Penicillium_sp_0410ARD6F_2
New.ReferenceOTU6949	0	0	0	0	0	0	0	0	0	0	0	0	1	0	0	0	0	0	0	0	0	0	0	0	0	0	0	0	0	0	Fungi; Ascomycota; Eurotiomycetes; Eurotiales; Trichocomaceae; Penicillium; Penicillium_sp_0410ARD6F_2
New.ReferenceOTU8014	0	0	0	0	0	0	0	0	0	0	0	0	0	0	0	0	0	0	1	0	0	0	0	0	0	0	0	0	0	0	Fungi; Ascomycota; Eurotiomycetes; Eurotiales; Trichocomaceae; Penicillium; Penicillium_sp_0410ARD6F_2
SH128752.06FU_KC411917_reps	0	0	0	0	0	0	0	0	0	0	0	0	0	0	0	0	0	0	0	0	0	0	1	0	0	0	0	0	0	0	Fungi; Ascomycota; Eurotiomycetes; Eurotiales; Trichocomaceae; Penicillium; Penicillium_sp_0410ARD6F_2
New.ReferenceOTU6877	2	1	0	0	0	1	0	0	2	0	2	0	0	0	0	0	0	8	1	0	0	0	0	15	0	0	0	0	5	0	Fungi; Ascomycota; Eurotiomycetes; Eurotiales; Trichocomaceae; Penicillium; Penicillium_sp_13_M_2
SH030666.06FU_EU076921_reps_singleton	0	0	0	0	0	0	0	0	1	0	0	0	0	0	0	0	0	0	0	0	0	0	0	0	0	0	0	0	0	0	Fungi; Ascomycota; Eurotiomycetes; Eurotiales; Trichocomaceae; Penicillium; Penicillium_sp_13_M_2
New.ReferenceOTU4752	3	1	2	0	0	2	4	0	1	0	0	1	2	1	0	0	0	0	0	0	1	0	1	0	1	0	1	5	1	0	Fungi; Ascomycota; Eurotiomycetes; Eurotiales; Trichocomaceae; Penicillium; Penicillium_sp_2_MM_2011
New.ReferenceOTU7977	0	0	0	0	0	0	0	0	0	0	0	0	1	0	1	0	0	0	0	0	2	0	0	0	0	0	0	0	0	0	Fungi; Ascomycota; Eurotiomycetes; Eurotiales; Trichocomaceae; Penicillium; Penicillium_sp_2_MM_2011
New.ReferenceOTU1504	0	0	0	0	0	0	0	0	0	0	0	0	0	0	0	0	0	1	0	0	0	0	0	0	0	0	0	0	0	0	Fungi; Ascomycota; Eurotiomycetes; Eurotiales; Trichocomaceae; Penicillium; Penicillium_sp_3_JH_2010
New.ReferenceOTU3561	0	0	0	0	0	0	0	0	0	0	0	0	0	0	0	0	0	3	0	0	0	0	0	0	0	0	0	0	0	0	Fungi; Ascomycota; Eurotiomycetes; Eurotiales; Trichocomaceae; Penicillium; Penicillium_sp_3_JH_2010
SH098824.06FU_GU981569_reps	0	0	1	0	0	0	0	0	0	0	0	0	0	1	0	0	0	1	0	0	0	0	0	0	0	0	0	0	0	0	Fungi; Ascomycota; Eurotiomycetes; Eurotiales; Trichocomaceae; Penicillium; Penicillium_sp_3_JH_2010
New.ReferenceOTU3025	0	0	0	0	0	0	0	0	0	0	0	0	0	0	0	0	0	0	0	0	0	0	2	0	0	0	2	0	0	0	Fungi; Ascomycota; Eurotiomycetes; Eurotiales; Trichocomaceae; Penicillium; Penicillium_sp_AHBR14
New.ReferenceOTU5523	0	1	0	0	0	1	0	0	1	0	0	0	1	0	0	0	1	1	1	0	0	0	0	0	0	0	0	0	0	0	Fungi; Ascomycota; Eurotiomycetes; Eurotiales; Trichocomaceae; Penicillium; Penicillium_sp_AHBR14
New.CleanUp.ReferenceOTU439	0	1	1	0	0	0	0	0	0	0	1	0	0	0	0	0	0	0	0	0	0	0	1	0	0	0	0	0	0	0	Fungi; Ascomycota; Eurotiomycetes; Eurotiales; Trichocomaceae; Penicillium; Penicillium_sp_ASR_146
New.ReferenceOTU1227	2	0	0	0	0	0	0	0	0	0	0	2	0	0	1	0	0	0	0	0	0	0	2	0	0	0	1	2	0	0	Fungi; Ascomycota; Eurotiomycetes; Eurotiales; Trichocomaceae; Penicillium; Penicillium_sp_ASR_146
New.ReferenceOTU6251	0	0	0	0	0	0	0	0	0	0	0	0	0	0	0	0	0	0	0	0	0	0	4	0	0	0	0	0	0	0	Fungi; Ascomycota; Eurotiomycetes; Eurotiales; Trichocomaceae; Penicillium; Penicillium_sp_ASR_146
New.ReferenceOTU1891	0	0	0	0	0	0	0	0	0	0	0	0	0	0	0	0	0	0	0	0	0	0	0	0	0	0	0	0	2	0	Fungi; Ascomycota; Eurotiomycetes; Eurotiales; Trichocomaceae; Penicillium; Penicillium_sp_ATT162
New.ReferenceOTU6217	0	0	0	0	0	0	0	0	0	0	0	0	0	0	0	0	1	0	0	0	0	0	0	0	0	0	0	0	0	0	Fungi; Ascomycota; Eurotiomycetes; Eurotiales; Trichocomaceae; Penicillium; Penicillium_sp_ATT162
New.ReferenceOTU2471	0	0	0	0	0	0	0	0	0	0	0	0	3	0	0	0	0	0	0	0	0	0	0	0	0	0	0	0	0	0	Fungi; Ascomycota; Eurotiomycetes; Eurotiales; Trichocomaceae; Penicillium; Penicillium_sp_CBMAI1335
New.ReferenceOTU2581	0	0	0	1	0	0	0	0	0	0	0	0	0	0	0	0	0	0	0	0	0	0	0	0	0	0	0	0	0	0	Fungi; Ascomycota; Eurotiomycetes; Eurotiales; Trichocomaceae; Penicillium; Penicillium_sp_CBMAI1335
New.ReferenceOTU2977	0	0	0	0	2	2	1	0	0	0	0	3	14	0	1	0	0	0	0	0	0	0	0	0	0	0	15	0	0	5	Fungi; Ascomycota; Eurotiomycetes; Eurotiales; Trichocomaceae; Penicillium; Penicillium_sp_CBMAI1335
SH128761.06FU_FR799508_reps	0	0	8	0	0	1	0	0	0	0	1	0	4	0	0	0	0	0	0	0	0	0	0	3	0	0	0	0	0	0	Fungi; Ascomycota; Eurotiomycetes; Eurotiales; Trichocomaceae; Penicillium; Penicillium_sp_CMV_2008d
New.ReferenceOTU4624	0	0	0	0	0	0	0	0	0	0	0	0	0	0	0	0	0	0	0	0	0	0	0	0	0	0	0	0	0	1	Fungi; Ascomycota; Eurotiomycetes; Eurotiales; Trichocomaceae; Penicillium; Penicillium_sp_FG48

New.ReferenceOTU4354	0	0	0	0	0	0	0	0	0	0	0	0	0	0	0	0	0	0	0	0	0	0	0	3	0	0	0	0	0	0	Fungi; Ascomycota; Eurotiomycetes; Eurotiales; Trichocomaceae; Penicillium; Penicillium_sp_FKI_4942
New.ReferenceOTU5557	0	0	0	0	0	0	0	0	0	0	0	3	0	0	0	0	0	0	0	0	0	0	0	0	0	0	0	0	0	0	Fungi; Ascomycota; Eurotiomycetes; Eurotiales; Trichocomaceae; Penicillium; Penicillium_sp_KCR_2010
New.ReferenceOTU7346	0	0	0	0	0	0	0	0	0	0	0	0	0	0	0	0	0	0	0	0	0	1	0	0	0	0	0	0	0	0	Fungi; Ascomycota; Eurotiomycetes; Eurotiales; Trichocomaceae; Penicillium; Penicillium_sp_KJ_2012
New.ReferenceOTU1345	1	0	0	0	0	0	0	0	0	0	0	1	0	0	0	0	0	0	0	0	0	0	0	0	0	0	0	0	0	0	Fungi; Ascomycota; Eurotiomycetes; Eurotiales; Trichocomaceae; Penicillium; Penicillium_sp_MS431
New.ReferenceOTU4154	0	0	0	0	0	0	0	0	0	0	0	0	0	0	0	0	0	0	0	0	0	2	1	0	0	0	0	0	0	0	Fungi; Ascomycota; Eurotiomycetes; Eurotiales; Trichocomaceae; Penicillium; Penicillium_sp_MS431
SH016773.06FU_JQ905765_reps_singleton	0	0	0	0	0	0	0	0	0	0	2	0	0	0	0	0	0	0	0	0	0	0	0	0	0	0	0	0	0	0	Fungi; Ascomycota; Eurotiomycetes; Eurotiales; Trichocomaceae; Penicillium; Penicillium_sp_MS431
SH113522.06FU_GU944578_refs	1	0	0	0	0	0	0	0	0	0	1	0	0	1	0	0	0	0	0	0	0	0	0	0	0	0	0	0	0	0	Fungi; Ascomycota; Eurotiomycetes; Eurotiales; Trichocomaceae; Penicillium; Penicillium_sp_MS431
New.ReferenceOTU2101	0	0	0	0	0	4	1	0	0	0	0	0	0	0	0	0	0	0	0	0	0	0	0	0	0	0	1	0	0	0	Fungi; Ascomycota; Eurotiomycetes; Eurotiales; Trichocomaceae; Penicillium; Penicillium_sp_NRRL_35186
New.ReferenceOTU6586	0	0	0	0	0	1	0	0	0	0	0	0	0	0	0	0	0	0	0	0	0	0	0	0	0	0	0	0	0	0	Fungi; Ascomycota; Eurotiomycetes; Eurotiales; Trichocomaceae; Penicillium; Penicillium_sp_NRRL_35186
New.ReferenceOTU7290	0	0	0	0	0	0	0	0	0	0	0	1	0	0	0	0	0	0	0	0	0	0	0	0	0	0	0	0	0	0	Fungi; Ascomycota; Eurotiomycetes; Eurotiales; Trichocomaceae; Penicillium; Penicillium_sp_NRRL_35186
New.ReferenceOTU7381	0	0	0	0	0	0	0	0	0	0	0	0	0	0	0	0	0	0	0	0	0	0	0	0	0	16	0	0	0	0	Fungi; Ascomycota; Eurotiomycetes; Eurotiales; Trichocomaceae; Penicillium; Penicillium_sp_NRRL_35186
SH128760.06FU_DQ123635_reps	0	0	0	0	0	0	0	0	0	0	0	1	1	0	0	0	0	0	0	0	0	0	0	0	0	0	0	0	0	0	Fungi; Ascomycota; Eurotiomycetes; Eurotiales; Trichocomaceae; Penicillium; Penicillium_sp_NRRL_35186
New.ReferenceOTU1998	0	0	0	0	0	0	0	0	0	0	0	0	0	0	0	0	0	0	0	0	0	0	0	0	0	0	0	0	1	0	Fungi; Ascomycota; Eurotiomycetes; Eurotiales; Trichocomaceae; Penicillium; Penicillium_sp_NRRL_35203
New.ReferenceOTU3452	0	0	0	0	0	0	0	1	0	0	0	0	0	0	0	0	0	0	0	0	0	0	0	0	0	0	0	0	0	0	Fungi; Ascomycota; Eurotiomycetes; Eurotiales; Trichocomaceae; Penicillium; Penicillium_sp_NRRL_35203
New.ReferenceOTU3709	0	0	1	0	0	0	0	0	0	0	0	0	0	0	0	0	0	0	0	0	0	0	0	0	0	0	0	0	0	0	Fungi; Ascomycota; Eurotiomycetes; Eurotiales; Trichocomaceae; Penicillium; Penicillium_sp_NRRL_35203
New.ReferenceOTU437	0	0	1	0	0	0	0	0	1	0	3	0	1	5	0	0	0	0	0	0	0	0	0	0	0	2	0	0	0	0	Fungi; Ascomycota; Eurotiomycetes; Eurotiales; Trichocomaceae; Penicillium; Penicillium_sp_NRRL_35203
New.ReferenceOTU4822	0	0	0	0	0	0	0	0	0	0	0	0	0	0	0	0	0	0	3	0	0	0	0	0	0	0	0	0	0	0	Fungi; Ascomycota; Eurotiomycetes; Eurotiales; Trichocomaceae; Penicillium; Penicillium_sp_NRRL_35203
New.ReferenceOTU795	0	0	0	0	0	0	0	0	0	0	0	0	0	2	0	0	0	0	19	0	0	0	0	0	0	0	0	0	0	0	Fungi; Ascomycota; Eurotiomycetes; Eurotiales; Trichocomaceae; Penicillium; Penicillium_sp_NRRL_35203
New.ReferenceOTU5066	0	0	0	0	0	0	0	0	0	0	0	0	1	0	0	0	0	0	0	0	0	0	0	0	0	0	0	0	0	0	Fungi; Ascomycota; Eurotiomycetes; Eurotiales; Trichocomaceae; Penicillium; Penicillium_sp_PSF34
SH098790.06FU_GU981582_refs	0	0	2	0	1	0	0	0	0	0	0	1	0	0	0	0	0	0	0	0	0	2	1	0	0	3	1	0	1	0	Fungi; Ascomycota; Eurotiomycetes; Eurotiales; Trichocomaceae; Penicillium; Penicillium_sp_PSF34
New.ReferenceOTU6944	1	0	5	0	1	0	3	2	2	1	1	1	0	1	2	4	2	0	2	1	5	5	0	1	0	0	0	0	0	0	Fungi; Ascomycota; Eurotiomycetes; Eurotiales; Trichocomaceae; Penicillium; Penicillium_sp_PSF36
New.ReferenceOTU7452	0	0	0	0	0	1	0	0	0	0	0	0	0	0	0	0	0	0	0	0	0	0	0	0	0	0	0	0	0	0	Fungi; Ascomycota; Eurotiomycetes; Eurotiales; Trichocomaceae; Penicillium; Penicillium_sp_RMF_4011B
New.ReferenceOTU2113	0	2	0	0	0	0	0	0	0	0	0	0	0	0	0	0	0	0	0	0	0	0	0	0	0	0	0	0	0	0	Fungi; Ascomycota; Eurotiomycetes; Eurotiales; Trichocomaceae; Penicillium; Penicillium_sp_RPS29
New.ReferenceOTU2591	0	0	4	0	0	4	1	0	2	0	1	1	4	1	0	2	0	3	0	0	0	2	2	0	0	0	1	1	0	0	Fungi; Ascomycota; Eurotiomycetes; Eurotiales; Trichocomaceae; Penicillium; Penicillium_sp_RPS29
New.ReferenceOTU4266	0	0	0	0	0	0	0	17	0	0	0	0	0	0	0	0	0	0	0	0	0	0	0	0	0	0	0	0	0	0	Fungi; Ascomycota; Eurotiomycetes; Eurotiales; Trichocomaceae; Penicillium; Penicillium_sp_RPS29
New.ReferenceOTU6024	0	0	1	0	0	1	0	0	0	0	1	0	0	0	0	0	0	0	0	0	0	0	1	0	1	0	0	0	0	0	Fungi; Ascomycota; Eurotiomycetes; Eurotiales; Trichocomaceae; Penicillium; Penicillium_sp_RPS29
SH098818.06FU_GU981608_reps	0	0	0	0	0	0	0	0	0	0	0	1	0	0	0	0	0	0	0	0	0	2	0	3	0	0	0	0	0	0	Fungi; Ascomycota; Eurotiomycetes; Eurotiales; Trichocomaceae; Penicillium; Penicillium_sp_RPS29
New.CleanUp.ReferenceOTU1041	0	0	0	1	0	0	0	0	0	0	0	0	0	0	0	0	0	0	0	0	0	0	0	0	0	0	0	0	0	0	Fungi; Ascomycota; Eurotiomycetes; Eurotiales; Trichocomaceae; Penicillium; Penicillium_sp_TSR60_5
New.ReferenceOTU1071	1	0	0	0	0	0	0	0	0	0	0	0	0	0	0	0	0	0	0	0	0	0	0	0	0	0	0	0	0	0	Fungi; Ascomycota; Eurotiomycetes; Eurotiales; Trichocomaceae; Penicillium; Penicillium_sp_TSR60_5
New.ReferenceOTU290	0	0	0	0	0	4	0	0	0	0	0	0	0	0	0	0	0	0	0	1	0	0	0	0	0	0	0	0	0	0	Fungi; Ascomycota; Eurotiomycetes; Eurotiales; Trichocomaceae; Penicillium; Penicillium_sp_TSR60_5
New.ReferenceOTU4559	0	0	0	0	0	0	0	1	0	0	0	0	0	0	0	0	0	0	0	0	0	0	0	0	0	0	0	0	0	0	Fungi; Ascomycota; Eurotiomycetes; Eurotiales; Trichocomaceae; Penicillium; Penicillium_sp_TSR60_5
New.ReferenceOTU7145	0	0	0	0	0	0	0	0	0	3	0	0	0	1	1	0	0	0	0	0	0	0	0	0	0	0	0	0	1	0	Fungi; Ascomycota; Eurotiomycetes; Eurotiales; Trichocomaceae; Penicillium; Penicillium_sp_TSR60_5
New.ReferenceOTU1189	0	0	1	0	0	0	0	0	0	0	2	0	0	0	0	0	1	0	0	0	0	0	0	0	0	0	0	0	0	0	Fungi; Ascomycota; Eurotiomycetes; Eurotiales; Trichocomaceae; Penicillium; Penicillium_sp_TSS19
New.ReferenceOTU1312	0	1	1	0	0	2	0	0	0	0	0	0	1	0	1	0	0	0	0	0	0	1	0	0	0	0	0	0	0	0	Fungi; Ascomycota; Eurotiomycetes; Eurotiales; Trichocomaceae; Penicillium; Penicillium_sp_TSS19
New.ReferenceOTU1395	0	0	0	0	0	0	0	0	0	0	0	0	0	0	0	0	0	0	0	0	0	0	2	0	0	0	0	0	0	0	Fungi; Ascomycota; Eurotiomycetes; Eurotiales; Trichocomaceae; Penicillium; Penicillium_sp_TSS19
New.ReferenceOTU1505	0	1	0	0	0	0	0	0	0	0	1	0	0	0	0	0	0	0	0	0	0	0	0	0	0	0	1	0	0	0	Fungi; Ascomycota; Eurotiomycetes; Eurotiales; Trichocomaceae; Penicillium; Penicillium_sp_TSS19
New.ReferenceOTU234	0	0	0	0	0	0	0	0	0	0	0	0	0	0	0	0	0	1	0	0	0	0	0	0	0	0	0	0	0	0	Fungi; Ascomycota; Eurotiomycetes; Eurotiales; Trichocomaceae; Penicillium; Penicillium_sp_TSS19
New.ReferenceOTU3126	0	4	0	0	0	0	0	1	0	0	0	0	1	0	0	0	0	0	0	0	0	0	0	0	0	0	0	0	0	0	Fungi; Ascomycota; Eurotiomycetes; Eurotiales; Trichocomaceae; Penicillium; Penicillium_sp_TSS19
New.ReferenceOTU358	0	0	0	0	0	0	0	0	0	0	0	0	0	0	0	0	0	1	0	0	0	0	0	0	0	0	0	0	0	0	Fungi; Ascomycota; Eurotiomycetes; Eurotiales; Trichocomaceae; Penicillium; Penicillium_sp_TSS19
New.ReferenceOTU4870	2	0	0	2	1	10	17	4	0	0	1	6	4	3	1	0	0	0	0	1	7	0	2	0	3	0	3	8	3	3	Fungi; Ascomycota; Eurotiomycetes; Eurotiales; Trichocomaceae; Penicillium; Penicillium_sp_TSS19
SH098822.06FU_GU981581_refs	0	2	0	0	0	0	0	0	1	0	0	0	0	0	0	0	0	0	0	0	0	0	0	0	0	0	0	0	0	0	Fungi; Ascomycota; Eurotiomycetes; Eurotiales; Trichocomaceae; Penicillium; Penicillium_sp_TSS19
SH135183.06FU_AF033413_reps	0	0	0	0	0	0	0	0	0	0	0	0	0	1	0	0	0	0	0	0	0	0	0	0	0	0	0	0	0	0	Fungi; Ascomycota; Eurotiomycetes; Eurotiales; Trichocomaceae; Penicillium; Penicillium_sp_Vega852
New.ReferenceOTU935	0	1	0	0	0	0	1	0	0	0	0	0	0	0	0	0	0	0	0	1	0	0	0	0	0	0	0	0	2	0	Fungi; Ascomycota; Eurotiomycetes; Eurotiales; Trichocomaceae; Penicillium; Penicillium_sp_VN5_3_2
New.ReferenceOTU6054	1	0	0	0	0	9	21	0	0	2	0	8	1	0	0	0	0	0	0	0	0	0	1	0	0	0	1	11	0	2	Fungi; Ascomycota; Eurotiomycetes; Eurotiales; Trichocomaceae; Penicillium; Penicillium_sp_WD2714
New.ReferenceOTU6167	2	0	0	0	4	64	24	2	2	5	0	28	6	0	4	1	0	0	0	0	1	0	1	0	6	0	8	35	0	3	Fungi; Ascomycota; Eurotiomycetes; Eurotiales; Trichocomaceae; Penicillium; Penicillium_sp_WD2714
SH098841.06FU_KF313081_reps	0	0	0	0	0	0	0	0	0	0	2	0	0	0	0	0	0	0	0	0	0	0	0	0	0	0	0	0	0	0	Fungi; Ascomycota; Eurotiomycetes; Eurotiales; Trichocomaceae; Penicillium; Penicillium_sp_YJM_2013
New.ReferenceOTU2739	0	0	0	0	0	0	0	0	0	0	0	0	0	0	0	1	0	0	0	0	0	0	0	0	0	0	0	0	0	0	Fungi; Ascomycota; Eurotiomycetes; Eurotiales; Trichocomaceae; Penicillium; Penicillium_sp_YM3II
SH020647.06FU_AB297800_reps_singleton	0	0	0	0	0	1	1	0	0	0	0	0	0	0	0	0	0	0	0	0	0	0	0	0	0	0	0	0	0	3	Fungi; Ascomycota; Eurotiomycetes; Eurotiales; Trichocomaceae; Penicillium; Penicillium_sp_YM3II
SH008168.06FU_KC485450_reps_singleton	0	0	0	0	0	0	0	0	0	0	0	0	0	0	0	0	0	0	0	0	0	1	0	0	0	0	0	0	0	0	Fungi; Ascomycota; Eurotiomycetes; Eurotiales; Trichocomaceae; Penicillium; Penicillium_steckii
SH098795.06FU_GU944597_refs	0	0	0	0	0	0	0	0	0	0	0	0	0	0	0	0	0	0	0	1	0	1	1	0	0	0	0	0	0	0	Fungi; Ascomycota; Eurotiomycetes; Eurotiales; Trichocomaceae; Penicillium; Penicillium_steckii
New.ReferenceOTU3288	0	0	0	0	0	0	0	0	0	0	0	0	0	0	0	0	0	1	0	0	0	0	0	0	0	0	0	0	0	0	Fungi; Ascomycota; Eurotiomycetes; Eurotiales; Trichocomaceae; Penicillium; Penicillium_toxicarium
New.ReferenceOTU7366	0	0	0	0	0	0	0	0	0	0	0	0	0	0	0	0	0	0	0	0	0	0	0	0	0	0	0	0	0	1	Fungi; Ascomycota; Eurotiomycetes; Eurotiales; Trichocomaceae; Penicillium; Penicillium_toxicarium
SH089796.06FU_FJ231015_reps	0	0	0	0	0	0	0	0	0	0	0	0	2	0	0	0	0	0	0	0	0	1	0	0	0	2	0	0	0	0	Fungi; Ascomycota; Eurotiomycetes; Eurotiales; Trichocomaceae; Penicillium; Penicillium_toxicarium
SH089801.06FU_FR774153_reps	0	0	0	0	1	0	0	0	0	0	0	0	0	0	0	0	0	0	0	0	0	0	0	0	0	1	0	0	0	0	Fungi; Ascomycota; Eurotiomycetes; Eurotiales; Trichocomaceae; Penicillium; Penicillium_toxicarium
New.ReferenceOTU3770	0	0	0	0	0	0	0	0	0	0	0	0	4	0	0	0	0	0	0	0	0	0	0	0	0	0	0	0	0	0	Fungi; Ascomycota; Eurotiomycetes; Eurotiales; Trichocomaceae; Penicillium; Penicillium_tularense
New.ReferenceOTU1173	0	0	3	1	1	1	0	0	0	0	1	0	0	0	0	0	0	0	0	0	0	4	1	2	0	0	0	0	0	0	Fungi; Ascomycota; Eurotiomycetes; Eurotiales; Trichocomaceae; Penicillium; Penicillium_vanoranjei
SH098796.06FU_GU981591_refs	0	0	0	0	0	20	0	1	0	0	0	1	0	1	0	0	0	0	0	1	0	1	1	0	1	0	0	0	0	1	Fungi; Ascomycota; Eurotiomycetes; Eurotiales; Trichocomaceae; Penicillium; Penicillium_wotroi
New.CleanUp.ReferenceOTU106	0	0	0	0	0	0	0	0	0	0	0	0	0	0	0	0	0	0	0	0	0	0	0	0	0	0	0	0	1	0	Fungi; Ascomycota; Eurotiomycetes; Eurotiales; Trichocomaceae; Penicillium; Penicillium_zonatum
New.CleanUp.ReferenceOTU1499	0	0	0	0	0	0	0	0	0	0	0	0	0	0	0	0	0	0	0	0	0	0	0	0	0	0	0	0	1	0	Fungi; Ascomycota; Eurotiomycetes; Eurotiales; Trichocomaceae; Penicillium; Penicillium_zonatum
New.ReferenceOTU4450	0	0	0	0	0	0	0	0	0	0	0	0	0	0	0	1	0	3	0	0	0	0	0	0	0	0	0	0	0	0	Fungi; Ascomycota; Eurotiomycetes; Eurotiales; Trichocomaceae; Penicillium; Penicillium_zonatum
New.ReferenceOTU4775	0	0	0	0	5	0	0	0	0	0	0	0	0	0	0	0	0	0	0	0	0	0	0	0	0	0	0	0	0	0	Fungi; Ascomycota; Eurotiomycetes; Eurotiales; Trichocomaceae; Penicillium; Penicillium_zonatum
New.ReferenceOTU5550	0	0	0	0	0	0	1	0	0	0	0	0	0	0	0	0	0	0	0	0	0	0	0	0	0	0	0	1	0	0	Fungi; Ascomycota; Eurotiomycetes; Eurotiales; Trichocomaceae; Penicillium; Penicillium_zonatum
New.ReferenceOTU6457	0	0	0	0	0	0	0	2	0	0	0	0	0	0	0	0	0	0	0	0	0	0	0	0	0	0	0	0	0	0	Fungi; Ascomycota; Eurotiomycetes; Eurotiales; Trichocomaceae; Penicillium; Penicillium_zonatum
New.ReferenceOTU7126	0	0	0	0	0	1	0	0	0	0	0	0	0	0	0	0	0	0	0	0	0	0	0	0	0	0	0	0	0	0	Fungi; Ascomycota; Eurotiomycetes; Eurotiales; Trichocomaceae; Penicillium; Penicillium_zonatum
New.ReferenceOTU1447	0	0	0	0	0	0	0	0	0	0	0	0	0	0	0	0	0	0	0	0	0	1	0	0	0	0	0	0	0	0	Fungi; Ascomycota; Eurotiomycetes; Eurotiales; Trichocomaceae; Phialosimplex; Phialosimplex_caninus
New.ReferenceOTU5134	0	0	0	0	0	0	0	0	0	0	0	0	0	0	0	0	0	0	0	0	0	3	0	0	0	0	0	0	0	1	Fungi; Ascomycota; Eurotiomycetes; Eurotiales; Trichocomaceae; Phialosimplex; Phialosimplex_caninus
SH033977.06FU_GQ169314_reps_singleton	0	0	0	0	0	0	0	0	0	0	0	0	0	0	1	0	0	0	0	0	0	8	0	0	0	0	0	0	0	0	Fungi; Ascomycota; Eurotiomycetes; Eurotiales; Trichocomaceae; Phialosimplex; Phialosimplex_caninus
New.ReferenceOTU104	0	0	0	3	0	9	0	0	0	0	0	0	0	0	0	0	0	0	0	0	0	0	0	0	0	0	0	0	0	0	Fungi; Ascomycota; Eurotiomycetes; Eurotiales; Trichocomaceae; Rasamsonia; Rasamsonia_brevistipitata
New.ReferenceOTU4094	0	0	0	0	0	1	0	0	0	0	0	0	0	0	0	0	0	0	0	0	0	0	0	0	0	0	0	0	0	0	Fungi; Ascomycota; Eurotiomycetes; Eurotiales; Trichocomaceae; Rasamsonia; Rasamsonia_brevistipitata
New.ReferenceOTU4674	0	0	0	3	0	0	0	0	0	0	0	0	0	0	0	0	0	0	0	0	0	0	0	0	0	0	0	0	0	0	Fungi; Ascomycota; Eurotiomycetes; Eurotiales; Trichocomaceae; Rasamsonia; Rasamsonia_brevistipitata
New.ReferenceOTU5179	0	0	0	1	5	0	0	0	0	0	0	0	0	0	0	0	0	0	0	0	0	0	0	0	0	0	0	0	0	0	Fungi; Ascomycota; Eurotiomycetes; Eurotiales; Trichocomaceae; Rasamsonia; Rasamsonia_brevistipitata
New.ReferenceOTU6087	0	0	0	0	0	1	0	0	0	0	0	0	0	0	0	0	0	0	0	0	0	0	0	0	0	0	0	0	0	0	Fungi; Ascomycota; Eurotiomycetes; Eurotiales; Trichocomaceae; Rasamsonia; Rasamsonia_brevistipitata
New.ReferenceOTU6617	0	0	0	0	0	2	0	0	0	0	0	0	0	0	0	0	0	0	0	0	0	0	0	0	0	0	0	0	0	0	Fungi; Ascomycota; Eurotiomycetes; Eurotiales; Trichocomaceae; Rasamsonia; Rasamsonia_brevistipitata
New.ReferenceOTU7676	0	0	0	0	0	1	0	0	0	0	0	0	0	0	0	0	0	0	0	0	0	0	0	0	0	0	0	0	0	0	Fungi; Ascomycota; Eurotiomycetes; Eurotiales; Trichocomaceae; Rasamsonia; Rasamsonia_brevistipitata
SH092257.06FU_JF417488_refs	0	0	0	1	0	5	0	1	0	0	0	0	0	0	0	0	0	0	0	0	0	0	0	0	0	0	0	0	0	0	Fungi; Ascomycota; Eurotiomycetes; Eurotiales; Trichocomaceae; Rasamsonia; Rasamsonia_brevistipitata
New.ReferenceOTU1819	0	0	0	0	0	0	0	0	0	0	0	0	0	0	0	1	0	0	0	0	0	1	0	0	0	0	0	0	0	0	Fungi; Ascomycota; Eurotiomycetes; Eurotiales; Trichocomaceae; Rasamsonia; Rasamsonia_byssochlamydoides
SH113557.06FU_JF417476_refs	0	0	1	0	0	0	0	1	0	0	0	0	0	2	0	2	1	0	0	0	0	0	4	0	1	0	0	0	0	0	Fungi; Ascomycota; Eurotiomycetes; Eurotiales; Trichocomaceae; Rasamsonia; Rasamsonia_byssochlamydoides
New.ReferenceOTU7877	0	0	1	0	0	0	0	0	0	0	0	0	0	0	0	0	0	0	0	0	0	0	0	0	0	0	0	0	0	0	Fungi; Ascomycota; Eurotiomycetes; Eurotiales; Trichocomaceae; Rasamsonia; Rasamsonia_cylindrospora
SH113568.06FU_JF417470_refs	0	0	0	0	0	0	0	0	0	0	0	0	0	0	0	0	0	0	0	0	0	0	0	0	0	0	0	1	0	0	Fungi; Ascomycota; Eurotiomycetes; Eurotiales; Trichocomaceae; Rasamsonia; Rasamsonia_cylindrospora
New.ReferenceOTU6026	0	0	0	0	0	0	0	0	0	0	1	0	0	0	0	0	0	0	0	0	0	0	0	0	0	0	0	0	0	0	Fungi; Ascomycota; Eurotiomycetes; Eurotiales; Trichocomaceae; Sagenomella; Sagenomella_sp
New.ReferenceOTU1374	0	0	0	0	0	0	1	0	0	0	0	0	0	0	0	4	0	0	0	0	1	1	2	0	0	0	8	0	0	0	Fungi; Ascomycota; Eurotiomycetes; Eurotiales; Trichocomaceae; Sagenomella; Sagenomella_sp_ASR_61
New.ReferenceOTU2617	0	0	0	0	0	0	0	0	0	0	0	0	0	0	0	0	0	0	0	0	0	3	0	0	0	0	1	0	0	0	Fungi; Ascomycota; Eurotiomycetes; Eurotiales; Trichocomaceae; Sagenomella; Sagenomella_sp_ASR_61
New.ReferenceOTU3286	0	0	0	0	0	0	0	0	0	0	0	0	0	0	0	0	0	0	0	0	0	0	0	0	0	0	0	0	0	1	Fungi; Ascomycota; Eurotiomycetes; Eurotiales; Trichocomaceae; Sagenomella; Sagenomella_sp_ASR_61
New.ReferenceOTU4482	0	0	0	0	0	0	0	0	0	0	0	0	0	0	2	0	0	0	0	0	0	0	0	0	0	0	0	0	0	0	Fungi; Ascomycota; Eurotiomycetes; Eurotiales; Trichocomaceae; Sagenomella; Sagenomella_sp_ASR_61
New.ReferenceOTU6728	0	0	0	0	0	0	0	0	0	0	0	0	0	0	0	0	0	0	0	0	0	3	0	0	0	0	0	0	0	0	Fungi; Ascomycota; Eurotiomycetes; Eurotiales; Trichocomaceae; Sagenomella; Sagenomella_sp_ASR_61
New.ReferenceOTU7371	0	0	0	0	0	0	0	0	0	0	0	1	0	0	2	2	0	1	0	0	5	24	0	0	2	0	6	1	0	0	Fungi; Ascomycota; Eurotiomycetes; Eurotiales; Trichocomaceae; Sagenomella; Sagenomella_sp_ASR_61
New.ReferenceOTU1800	0	0	0	0	1	3	0	0	0	0	0	0	0	1	0	0	1	0	0	0	0	0	0	0	0	0	5	0	0	7	Fungi; Ascomycota; Eurotiomycetes; Eurotiales; Trichocomaceae; Talaromyces; Talaromyces_bacillisporus
New.ReferenceOTU1840	0	0	1	0	1	2	4	0	0	0	0	0	0	0	0	0	0	0	0	0	2	0	0	0	0	0	0	0	1	0	Fungi; Ascomycota; Eurotiomycetes; Eurotiales; Trichocomaceae; Talaromyces; Talaromyces_bacillisporus
New.ReferenceOTU1905	0	0	0	0	0	1	2	0	0	0	0	0	0	0	0	0	0	0	0	0	0	0	0	0	0	0	0	0	0	0	Fungi; Ascomycota; Eurotiomycetes; Eurotiales; Trichocomaceae; Talaromyces; Talaromyces_bacillisporus
New.ReferenceOTU2522	0	0	0	0	0	0	1	0	0	0	0	0	0	0	1	0	0	0	0	0	0	0	0	0	0	0	0	0	1	0	Fungi; Ascomycota; Eurotiomycetes; Eurotiales; Trichocomaceae; Talaromyces; Talaromyces_bacillisporus
New.ReferenceOTU355	0	0	0	0	1	0	0	0	0	0	0	0	0	0	0	0	0	0	0	0	0	0	0	0	0	0	0	0	0	0	Fungi; Ascomycota; Eurotiomycetes; Eurotiales; Trichocomaceae; Talaromyces; Talaromyces_bacillisporus
New.ReferenceOTU5707	0	3	0	0	1	5	9	0	0	0	0	2	1	5	2	0	1	0	0	0	2	1	0	0	0	0	0	0	4	1	Fungi; Ascomycota; Eurotiomycetes; Eurotiales; Trichocomaceae; Talaromyces; Talaromyces_bacillisporus
SH109964.06FU_AB176608_reps	0	0	0	18	3	16	2	0	0	3	0	2	0	13	10	0	2	0	0	0	0	0	1	0	3	0	22	0	0	7	Fungi; Ascomycota; Eurotiomycetes; Eurotiales; Trichocomaceae; Talaromyces; Talaromyces_bacillisporus
SH109968.06FU_GU396599_reps	0	0	0	1	1	1	0	0	0	1	1	0	0	0	0	0	0	0	0	0	0	0	1	0	1	0	1	0	0	1	Fungi; Ascomycota; Eurotiomycetes; Eurotiales; Trichocomaceae; Talaromyces; Talaromyces_bacillisporus
New.ReferenceOTU1743	0	0	0	0	0	0	0	0	0	0	0	0	0	0	0	0	0	0	0	0	0	0	0	0	0	0	0	0	1	0	Fungi; Ascomycota; Eurotiomycetes; Eurotiales; Trichocomaceae; Talaromyces; Talaromyces_flavus
New.ReferenceOTU1767	0	0	0	0	0	1	0	0	0	0	0	0	0	0	0	0	1	0	0	0	0	0	0	1	0	0	0	0	6	0	Fungi; Ascomycota; Eurotiomycetes; Eurotiales; Trichocomaceae; Talaromyces; Talaromyces_flavus
New.ReferenceOTU2229	0	0	0	0	0	0	0	0	0	0	0	0	1	0	0	0	0	0	0	0	0	0	0	0	0	0	0	0	0	0	Fungi; Ascomycota; Eurotiomycetes; Eurotiales; Trichocomaceae; Talaromyces; Talaromyces_flavus
New.ReferenceOTU2896	0	1	0	0	3	0	0	0	0	0	1	1	0	0	0	0	0	0	0	0	0	0	0	4	0	1	0	0	1	0	Fungi; Ascomycota; Eurotiomycetes; Eurotiales; Trichocomaceae; Talaromyces; Talaromyces_flavus
New.ReferenceOTU338	0	0	2	0	1	0	0	0	0	0	2	0	0	0	0	0	0	0	0	0	0	2	2	0	0	0	1	1	0	0	Fungi; Ascomycota; Eurotiomycetes; Eurotiales; Trichocomaceae; Talaromyces; Talaromyces_flavus
New.ReferenceOTU3423	0	0	0	0	0	0	0	0	0	0	0	0	0	0	1	2	0	0	1	0	3	0	5	0	1	0	2	0	1	0	Fungi; Ascomycota; Eurotiomycetes; Eurotiales; Trichocomaceae; Talaromyces; Talaromyces_flavus

New.ReferenceOTU5243	0	0	1	0	0	0	0	0	0	0	0	0	0	0	0	0	0	0	0	0	1	0	0	0	1	0	0	0	12	0	Fungi; Ascomycota; Eurotiomycetes; Eurotiales; Trichocomaceae; Talaromyces; Talaromyces_flavus
New.ReferenceOTU68	0	0	0	0	1	0	0	0	0	0	0	0	0	0	0	0	0	0	0	0	0	0	0	0	0	0	0	0	0	0	Fungi; Ascomycota; Eurotiomycetes; Eurotiales; Trichocomaceae; Talaromyces; Talaromyces_flavus
New.ReferenceOTU7127	0	0	0	0	2	0	0	0	0	0	0	0	0	0	0	0	0	0	0	0	0	0	1	0	0	0	0	0	0	0	Fungi; Ascomycota; Eurotiomycetes; Eurotiales; Trichocomaceae; Talaromyces; Talaromyces_flavus
New.ReferenceOTU7957	0	0	0	0	0	0	0	0	0	0	0	0	0	0	1	0	0	0	0	0	0	0	0	0	0	0	0	0	0	0	Fungi; Ascomycota; Eurotiomycetes; Eurotiales; Trichocomaceae; Talaromyces; Talaromyces_flavus
New.ReferenceOTU8020	0	0	0	0	0	0	0	0	0	0	0	0	0	0	0	0	0	0	0	0	1	0	0	0	0	0	0	1	0	0	Fungi; Ascomycota; Eurotiomycetes; Eurotiales; Trichocomaceae; Talaromyces; Talaromyces_flavus
SH026206.06FU_JN098082_reps_singleton	0	2	0	0	0	0	0	0	0	1	0	1	0	0	1	0	0	0	0	1	0	0	4	1	0	1	0	2	0	1	Fungi; Ascomycota; Eurotiomycetes; Eurotiales; Trichocomaceae; Talaromyces; Talaromyces_flavus
New.ReferenceOTU2488	0	0	0	0	0	0	0	0	0	0	0	0	0	0	0	2	0	0	0	0	0	0	0	0	0	0	0	0	0	0	Fungi; Ascomycota; Eurotiomycetes; Eurotiales; Trichocomaceae; Talaromyces; Talaromyces_leycettanus
New.ReferenceOTU39	0	3	0	0	0	0	0	0	0	0	0	0	0	0	0	0	0	0	0	0	0	0	0	0	0	0	0	0	0	0	Fungi; Ascomycota; Eurotiomycetes; Eurotiales; Trichocomaceae; Talaromyces; Talaromyces_leycettanus
New.ReferenceOTU4964	0	0	0	0	0	0	0	0	0	0	0	0	0	0	0	0	0	0	0	0	0	0	3	0	0	0	0	0	0	0	Fungi; Ascomycota; Eurotiomycetes; Eurotiales; Trichocomaceae; Talaromyces; Talaromyces_marneffei
New.ReferenceOTU6513	0	0	0	0	0	0	0	0	0	0	0	0	0	0	0	0	0	13	0	0	0	0	0	0	0	0	0	0	0	0	Fungi; Ascomycota; Eurotiomycetes; Eurotiales; Trichocomaceae; Talaromyces; Talaromyces_mimosinus
New.ReferenceOTU1497	0	0	0	0	0	0	0	0	0	0	0	0	0	0	0	0	0	0	0	0	0	0	0	0	0	0	0	0	1	0	Fungi; Ascomycota; Eurotiomycetes; Eurotiales; Trichocomaceae; Talaromyces; Talaromyces_palmae
New.ReferenceOTU960	0	0	0	0	0	0	0	0	0	0	0	0	0	0	0	0	0	0	0	0	0	0	1	0	1	0	0	0	0	0	Fungi; Ascomycota; Eurotiomycetes; Eurotiales; Trichocomaceae; Talaromyces; Talaromyces_palmae
SH109971.06FU_JN899396_refs	0	0	0	0	0	0	0	0	0	0	0	0	0	0	0	0	0	0	0	0	0	0	0	0	3	0	0	0	0	0	Fungi; Ascomycota; Eurotiomycetes; Eurotiales; Trichocomaceae; Talaromyces; Talaromyces_palmae
New.ReferenceOTU2372	0	0	0	0	0	0	0	0	0	0	0	0	0	0	0	0	0	0	0	0	0	0	0	0	4	0	0	0	0	0	Fungi; Ascomycota; Eurotiomycetes; Eurotiales; Trichocomaceae; Talaromyces; Talaromyces_proteolyticus
New.ReferenceOTU2974	0	0	0	0	0	0	0	0	0	0	0	0	0	0	0	0	0	0	0	0	0	0	0	0	2	0	0	0	0	0	Fungi; Ascomycota; Eurotiomycetes; Eurotiales; Trichocomaceae; Talaromyces; Talaromyces_proteolyticus
New.ReferenceOTU3964	0	0	0	0	0	0	0	0	0	0	0	0	0	0	16	0	0	0	0	0	0	0	4	0	19	0	0	3	0	0	Fungi; Ascomycota; Eurotiomycetes; Eurotiales; Trichocomaceae; Talaromyces; Talaromyces_proteolyticus
New.ReferenceOTU5174	0	0	0	0	0	0	0	0	0	0	0	0	0	0	0	0	0	0	0	2	0	0	0	0	0	0	0	0	0	0	Fungi; Ascomycota; Eurotiomycetes; Eurotiales; Trichocomaceae; Talaromyces; Talaromyces_proteolyticus
New.ReferenceOTU6089	0	0	0	0	0	0	0	0	0	0	0	0	0	0	0	0	0	0	0	0	0	0	0	0	3	0	0	0	0	0	Fungi; Ascomycota; Eurotiomycetes; Eurotiales; Trichocomaceae; Talaromyces; Talaromyces_proteolyticus
New.ReferenceOTU6825	0	0	0	0	0	0	0	0	0	0	0	0	0	0	0	0	0	0	0	1	0	0	0	0	0	0	0	0	0	0	Fungi; Ascomycota; Eurotiomycetes; Eurotiales; Trichocomaceae; Talaromyces; Talaromyces_proteolyticus
SH014690.06FU_JN899336_refs_singleton	0	1	0	0	0	0	0	0	0	0	0	0	0	0	0	0	0	0	0	0	0	0	1	0	0	0	0	0	0	0	Fungi; Ascomycota; Eurotiomycetes; Eurotiales; Trichocomaceae; Talaromyces; Talaromyces_proteolyticus
New.ReferenceOTU1666	2	0	1	1	0	1	0	0	1	3	1	1	2	0	0	0	2	10	0	1	0	0	0	25	0	2	0	0	1	0	Fungi; Ascomycota; Eurotiomycetes; Eurotiales; Trichocomaceae; Talaromyces; Talaromyces_purpurogenus
New.ReferenceOTU3401	0	0	0	0	0	0	0	0	0	0	0	0	0	0	0	0	0	0	0	0	0	0	0	1	0	0	0	0	2	0	Fungi; Ascomycota; Eurotiomycetes; Eurotiales; Trichocomaceae; Talaromyces; Talaromyces_purpurogenus
New.ReferenceOTU4097	0	0	0	0	0	0	0	0	0	3	0	0	0	0	0	0	0	0	0	0	0	0	0	0	0	0	0	0	0	0	Fungi; Ascomycota; Eurotiomycetes; Eurotiales; Trichocomaceae; Talaromyces; Talaromyces_purpurogenus
New.ReferenceOTU4211	0	0	0	0	0	0	0	0	0	0	0	0	0	0	0	0	0	0	0	0	0	0	0	2	0	0	0	0	0	0	Fungi; Ascomycota; Eurotiomycetes; Eurotiales; Trichocomaceae; Talaromyces; Talaromyces_purpurogenus
New.ReferenceOTU5796	0	0	0	1	0	0	0	0	0	8	7	1	0	0	1	0	0	0	0	5	1	0	0	16	0	1	0	0	4	0	Fungi; Ascomycota; Eurotiomycetes; Eurotiales; Trichocomaceae; Talaromyces; Talaromyces_purpurogenus
New.ReferenceOTU6481	0	0	0	0	1	0	0	0	0	0	0	0	0	0	0	0	0	0	0	0	0	0	0	0	0	0	0	0	0	0	Fungi; Ascomycota; Eurotiomycetes; Eurotiales; Trichocomaceae; Talaromyces; Talaromyces_purpurogenus
New.ReferenceOTU6686	0	0	0	0	0	0	0	0	0	0	0	0	0	0	0	0	0	0	0	0	0	0	0	0	0	13	0	0	6	0	Fungi; Ascomycota; Eurotiomycetes; Eurotiales; Trichocomaceae; Talaromyces; Talaromyces_purpurogenus
New.ReferenceOTU6885	0	0	1	0	0	0	0	0	0	4	4	0	0	0	2	0	0	3	0	0	0	0	0	21	0	3	0	0	16	0	Fungi; Ascomycota; Eurotiomycetes; Eurotiales; Trichocomaceae; Talaromyces; Talaromyces_purpurogenus
New.ReferenceOTU8085	0	0	0	0	0	0	0	0	0	0	0	0	0	0	0	0	0	0	0	0	0	0	0	0	0	4	0	0	0	0	Fungi; Ascomycota; Eurotiomycetes; Eurotiales; Trichocomaceae; Talaromyces; Talaromyces_purpurogenus
New.ReferenceOTU6170	0	0	0	0	0	0	0	1	2	0	0	0	0	0	0	0	0	0	0	0	0	0	0	0	0	0	1	0	0	0	Fungi; Ascomycota; Eurotiomycetes; Eurotiales; Trichocomaceae; Talaromyces; Talaromyces_rotundus
New.ReferenceOTU7293	0	0	0	0	1	0	0	0	0	0	0	0	0	0	0	0	0	0	0	0	0	0	0	0	0	0	0	0	0	0	Fungi; Ascomycota; Eurotiomycetes; Eurotiales; Trichocomaceae; Talaromyces; Talaromyces_rotundus
SH109977.06FU_KF196906_reps	0	0	0	0	0	0	0	0	0	0	0	0	0	0	0	0	0	0	0	0	0	0	1	0	0	0	0	0	0	0	Fungi; Ascomycota; Eurotiomycetes; Eurotiales; Trichocomaceae; Talaromyces; Talaromyces_rotundus
New.ReferenceOTU4514	0	0	0	0	0	0	0	0	0	1	0	0	0	0	0	0	0	0	0	0	0	0	0	0	0	0	0	0	0	0	Fungi; Ascomycota; Eurotiomycetes; Eurotiales; Trichocomaceae; Talaromyces; Talaromyces_sp
New.ReferenceOTU5453	3	0	0	4	0	0	0	0	0	0	0	0	0	0	0	0	0	0	0	0	0	0	0	0	0	0	0	0	1	0	Fungi; Ascomycota; Eurotiomycetes; Eurotiales; Trichocomaceae; Talaromyces; Talaromyces_sp
New.ReferenceOTU7333	0	0	0	0	0	0	0	0	0	0	0	0	1	0	0	0	0	0	0	0	0	0	0	0	0	0	0	0	0	0	Fungi; Ascomycota; Eurotiomycetes; Eurotiales; Trichocomaceae; Talaromyces; Talaromyces_sp
New.ReferenceOTU957	0	0	0	0	0	0	0	0	0	0	0	0	0	0	0	0	0	0	0	0	2	0	0	0	0	0	0	0	0	0	Fungi; Ascomycota; Eurotiomycetes; Eurotiales; Trichocomaceae; Talaromyces; Talaromyces_sp
New.ReferenceOTU7441	0	0	0	0	0	0	0	0	0	0	0	0	0	0	0	0	0	0	2	0	0	0	0	0	0	0	0	0	0	0	Fungi; Ascomycota; Eurotiomycetes; Eurotiales; Trichocomaceae; Talaromyces; Talaromyces_sp_ASR_194
New.ReferenceOTU3334	0	1	0	0	0	0	0	0	0	0	0	0	0	0	0	1	1	0	0	0	0	0	6	0	0	0	0	0	0	0	Fungi; Ascomycota; Eurotiomycetes; Eurotiales; Trichocomaceae; Talaromyces; Talaromyces_sp_NRRL_62223
SH014685.06FU_KF196917_reps_singleton	0	0	0	0	0	0	0	0	0	0	0	0	0	0	0	0	1	0	0	0	0	0	7	0	0	0	0	1	0	0	Fungi; Ascomycota; Eurotiomycetes; Eurotiales; Trichocomaceae; Talaromyces; Talaromyces_sp_NRRL_62223
New.ReferenceOTU4545	0	0	0	0	0	0	0	0	0	0	0	0	0	0	0	0	0	1	0	0	0	0	0	0	0	0	0	0	0	0	Fungi; Ascomycota; Eurotiomycetes; Eurotiales; Trichocomaceae; Talaromyces; Talaromyces_subinflatus
New.ReferenceOTU6160	0	0	0	0	0	0	0	0	0	0	0	0	0	0	0	0	0	0	1	0	0	0	0	0	0	0	0	0	0	0	Fungi; Ascomycota; Eurotiomycetes; Eurotiales; Trichocomaceae; Talaromyces; Talaromyces_subinflatus
SH109973.06FU_JN899397_refs	0	0	0	0	0	0	0	0	0	0	0	0	0	0	0	0	0	0	0	0	0	0	0	4	0	0	0	0	0	1	Fungi; Ascomycota; Eurotiomycetes; Eurotiales; Trichocomaceae; Talaromyces; Talaromyces_subinflatus
New.ReferenceOTU1195	0	0	0	0	0	2	1	1	0	0	1	2	4	0	0	0	0	0	0	1	0	0	0	4	0	1	0	0	3	0	Fungi; Ascomycota; Eurotiomycetes; Eurotiales; Trichocomaceae; Talaromyces; Talaromyces_viridulus
New.ReferenceOTU3874	0	0	8	0	1	1	0	1	2	8	1	3	1	3	0	8	0	0	0	0	0	0	0	2	0	0	0	0	0	0	Fungi; Ascomycota; Eurotiomycetes; Eurotiales; Trichocomaceae; Talaromyces; Talaromyces_viridulus
New.ReferenceOTU5469	0	2	0	0	0	0	0	0	0	0	0	0	0	0	0	0	0	1	0	0	0	0	0	0	0	1	0	0	0	0	Fungi; Ascomycota; Eurotiomycetes; Eurotiales; Trichocomaceae; Talaromyces; Talaromyces_viridulus
SH128745.06FU_L37406_refs	0	1	1	0	1	0	0	0	0	1	2	2	1	1	1	0	0	0	1	1	0	2	3	4	5	0	4	2	3	3	Fungi; Ascomycota; Eurotiomycetes; Eurotiales; Trichocomaceae; Talaromyces; Talaromyces_viridulus
SH128746.06FU_JX315664_refs	0	0	0	0	0	0	0	0	0	0	0	0	0	0	1	0	0	0	0	0	0	0	0	0	0	0	0	0	0	0	Fungi; Ascomycota; Eurotiomycetes; Eurotiales; Trichocomaceae; Talaromyces; Talaromyces_viridulus
SH128769.06FU_JN899314_refs	0	4	1	0	0	0	1	0	1	0	1	0	0	0	0	0	0	0	0	0	0	0	0	1	0	1	0	1	0	0	Fungi; Ascomycota; Eurotiomycetes; Eurotiales; Trichocomaceae; Talaromyces; Talaromyces_viridulus
SH128775.06FU_KC768091_reps	0	0	0	0	0	0	0	0	0	1	0	1	0	0	0	0	0	0	0	0	0	0	0	0	0	0	0	0	0	0	Fungi; Ascomycota; Eurotiomycetes; Eurotiales; Trichocomaceae; Talaromyces; Talaromyces_viridulus
New.ReferenceOTU643	0	0	0	0	0	0	0	0	0	0	0	0	0	0	0	0	0	0	0	4	0	0	0	0	0	0	0	0	0	0	Fungi; Ascomycota; Eurotiomycetes; Eurotiales; Trichocomaceae; Trichocoma; Trichocoma_paradoxa
New.CleanUp.ReferenceOTU1629	0	0	0	0	0	0	0	0	1	0	0	0	0	0	0	0	0	0	0	0	0	0	0	0	0	0	0	0	0	0	Fungi; Ascomycota; Eurotiomycetes; Eurotiales; Trichocomaceae; unidentified; Trichocomaceae_sp
New.CleanUp.ReferenceOTU885	0	0	0	0	0	0	0	0	0	0	0	0	0	0	0	0	0	1	0	0	0	0	0	0	0	0	0	0	0	0	Fungi; Ascomycota; Eurotiomycetes; Eurotiales; Trichocomaceae; unidentified; Trichocomaceae_sp
New.ReferenceOTU1024	0	7	0	0	0	0	0	0	0	0	0	0	0	0	0	0	0	0	0	0	0	0	0	0	0	0	0	0	3	0	Fungi; Ascomycota; Eurotiomycetes; Eurotiales; Trichocomaceae; unidentified; Trichocomaceae_sp
New.ReferenceOTU1155	0	0	1	0	0	0	0	0	0	0	0	1	0	0	0	0	0	0	0	0	0	0	0	0	0	0	0	0	0	0	Fungi; Ascomycota; Eurotiomycetes; Eurotiales; Trichocomaceae; unidentified; Trichocomaceae_sp
New.ReferenceOTU1200	0	0	0	0	0	1	6	0	0	0	0	0	1	0	0	0	0	1	0	0	0	0	0	0	0	0	0	0	0	0	Fungi; Ascomycota; Eurotiomycetes; Eurotiales; Trichocomaceae; unidentified; Trichocomaceae_sp
New.ReferenceOTU136	0	0	1	0	0	0	0	0	0	0	0	0	0	0	0	0	0	0	0	0	0	0	0	0	0	0	0	0	0	0	Fungi; Ascomycota; Eurotiomycetes; Eurotiales; Trichocomaceae; unidentified; Trichocomaceae_sp
New.ReferenceOTU1458	0	0	0	0	1	4	1	0	0	2	0	1	1	0	0	0	0	0	0	0	0	0	1	0	0	0	4	0	0	1	Fungi; Ascomycota; Eurotiomycetes; Eurotiales; Trichocomaceae; unidentified; Trichocomaceae_sp
New.ReferenceOTU161	0	1	0	0	0	0	0	0	0	0	0	0	0	0	0	0	0	0	0	0	0	0	0	0	0	2	0	0	1	0	Fungi; Ascomycota; Eurotiomycetes; Eurotiales; Trichocomaceae; unidentified; Trichocomaceae_sp
New.ReferenceOTU1652	0	0	0	0	3	0	0	0	0	0	0	4	0	0	0	0	0	0	0	0	0	0	0	0	0	0	0	0	0	0	Fungi; Ascomycota; Eurotiomycetes; Eurotiales; Trichocomaceae; unidentified; Trichocomaceae_sp
New.ReferenceOTU1780	0	0	0	0	0	0	0	0	0	0	0	0	0	0	0	0	0	0	0	0	0	0	0	0	2	0	0	0	0	0	Fungi; Ascomycota; Eurotiomycetes; Eurotiales; Trichocomaceae; unidentified; Trichocomaceae_sp
New.ReferenceOTU182	0	3	2	0	7	15	2	4	3	1	3	15	1	0	2	3	2	4	1	3	8	24	22	2	2	0	7	7	3	7	Fungi; Ascomycota; Eurotiomycetes; Eurotiales; Trichocomaceae; unidentified; Trichocomaceae_sp
New.ReferenceOTU1940	1	0	1	1	0	0	0	3	0	1	0	0	1	0	0	0	1	0	0	0	0	1	2	0	4	0	0	0	0	1	Fungi; Ascomycota; Eurotiomycetes; Eurotiales; Trichocomaceae; unidentified; Trichocomaceae_sp
New.ReferenceOTU2145	0	0	0	0	0	0	0	0	0	0	0	0	0	0	0	0	0	0	0	0	0	0	2	1	0	0	0	0	0	1	Fungi; Ascomycota; Eurotiomycetes; Eurotiales; Trichocomaceae; unidentified; Trichocomaceae_sp
New.ReferenceOTU2208	2	0	0	1	0	11	21	0	0	0	0	1	1	0	0	0	0	0	0	0	0	0	0	0	0	0	0	0	0	2	Fungi; Ascomycota; Eurotiomycetes; Eurotiales; Trichocomaceae; unidentified; Trichocomaceae_sp
New.ReferenceOTU228	0	0	1	0	0	1	0	0	0	0	0	0	0	0	0	0	0	21	0	0	0	0	0	0	0	0	0	0	0	0	Fungi; Ascomycota; Eurotiomycetes; Eurotiales; Trichocomaceae; unidentified; Trichocomaceae_sp
New.ReferenceOTU2318	0	1	1	0	0	0	1	1	0	0	0	0	1	0	0	1	0	0	0	0	0	1	0	0	0	0	2	0	0	0	Fungi; Ascomycota; Eurotiomycetes; Eurotiales; Trichocomaceae; unidentified; Trichocomaceae_sp
New.ReferenceOTU2607	0	0	0	0	0	0	0	2	0	3	0	0	0	0	0	0	0	0	0	1	1	0	0	0	0	0	0	0	0	1	Fungi; Ascomycota; Eurotiomycetes; Eurotiales; Trichocomaceae; unidentified; Trichocomaceae_sp
New.ReferenceOTU2844	0	0	1	0	0	0	0	0	0	0	0	1	0	1	0	0	0	0	0	0	0	0	0	0	0	0	1	0	0	0	Fungi; Ascomycota; Eurotiomycetes; Eurotiales; Trichocomaceae; unidentified; Trichocomaceae_sp
New.ReferenceOTU3056	0	0	0	0	0	0	0	0	0	0	0	0	6	0	0	0	0	0	0	0	0	0	0	0	0	0	0	0	0	0	Fungi; Ascomycota; Eurotiomycetes; Eurotiales; Trichocomaceae; unidentified; Trichocomaceae_sp
New.ReferenceOTU3153	1	4	0	2	0	0	0	0	0	0	0	0	0	0	0	0	0	0	0	0	0	1	0	0	0	0	0	0	0	0	Fungi; Ascomycota; Eurotiomycetes; Eurotiales; Trichocomaceae; unidentified; Trichocomaceae_sp
New.ReferenceOTU327	0	0	0	0	0	0	0	0	0	0	0	0	0	0	0	0	0	0	0	0	0	0	0	0	2	0	0	1	0	0	Fungi; Ascomycota; Eurotiomycetes; Eurotiales; Trichocomaceae; unidentified; Trichocomaceae_sp
New.ReferenceOTU3295	0	0	0	0	0	0	0	0	0	0	0	0	0	1	0	0	0	0	0	0	0	0	0	0	0	0	0	0	0	0	Fungi; Ascomycota; Eurotiomycetes; Eurotiales; Trichocomaceae; unidentified; Trichocomaceae_sp
New.ReferenceOTU3337	0	0	0	0	0	0	0	0	0	0	0	0	0	0	0	0	0	0	0	0	1	0	0	0	0	0	0	0	0	0	Fungi; Ascomycota; Eurotiomycetes; Eurotiales; Trichocomaceae; unidentified; Trichocomaceae_sp
New.ReferenceOTU3661	0	1	0	0	0	7	0	0	0	0	0	0	0	0	0	0	0	0	0	0	1	0	0	0	4	1	0	4	0	0	Fungi; Ascomycota; Eurotiomycetes; Eurotiales; Trichocomaceae; unidentified; Trichocomaceae_sp
New.ReferenceOTU3678	0	0	0	0	0	0	0	0	0	0	0	0	0	0	0	0	0	0	0	0	0	1	1	0	0	0	0	0	0	0	Fungi; Ascomycota; Eurotiomycetes; Eurotiales; Trichocomaceae; unidentified; Trichocomaceae_sp
New.ReferenceOTU4200	0	0	0	0	0	0	0	0	0	0	0	0	0	0	0	0	0	0	0	0	0	0	0	0	0	0	1	0	0	0	Fungi; Ascomycota; Eurotiomycetes; Eurotiales; Trichocomaceae; unidentified; Trichocomaceae_sp
New.ReferenceOTU4364	0	0	0	0	0	0	2	0	0	0	0	1	0	0	0	0	0	0	0	0	0	0	1	0	0	0	1	0	0	2	Fungi; Ascomycota; Eurotiomycetes; Eurotiales; Trichocomaceae; unidentified; Trichocomaceae_sp
New.ReferenceOTU4595	0	1	0	0	0	0	0	0	0	0	0	0	0	0	0	0	0	0	0	0	0	0	0	1	0	0	0	0	0	0	Fungi; Ascomycota; Eurotiomycetes; Eurotiales; Trichocomaceae; unidentified; Trichocomaceae_sp
New.ReferenceOTU4675	0	0	0	0	0	0	0	0	0	0	0	0	0	0	0	0	0	0	0	1	0	0	0	0	0	0	0	0	0	0	Fungi; Ascomycota; Eurotiomycetes; Eurotiales; Trichocomaceae; unidentified; Trichocomaceae_sp
New.ReferenceOTU4700	0	0	0	0	0	0	0	0	0	0	0	0	0	0	0	0	0	0	0	0	0	0	0	0	1	0	0	0	0	0	Fungi; Ascomycota; Eurotiomycetes; Eurotiales; Trichocomaceae; unidentified; Trichocomaceae_sp
New.ReferenceOTU4791	0	0	0	0	0	0	0	0	0	0	0	0	0	0	0	0	0	0	0	0	1	0	1	0	0	0	0	0	0	0	Fungi; Ascomycota; Eurotiomycetes; Eurotiales; Trichocomaceae; unidentified; Trichocomaceae_sp
New.ReferenceOTU5094	0	0	0	0	0	0	0	0	0	0	0	0	0	0	0	0	0	0	0	0	0	0	0	0	0	0	0	0	1	0	Fungi; Ascomycota; Eurotiomycetes; Eurotiales; Trichocomaceae; unidentified; Trichocomaceae_sp
New.ReferenceOTU5326	1	0	0	0	0	0	0	0	0	0	0	0	0	0	0	0	0	0	0	0	0	0	0	0	0	0	0	0	0	0	Fungi; Ascomycota; Eurotiomycetes; Eurotiales; Trichocomaceae; unidentified; Trichocomaceae_sp
New.ReferenceOTU5716	0	0	2	1	0	10	1	1	0	1	0	0	6	0	7	5	7	0	0	0	12	10	1	0	3	0	0	5	0	1	Fungi; Ascomycota; Eurotiomycetes; Eurotiales; Trichocomaceae; unidentified; Trichocomaceae_sp
New.ReferenceOTU5745	0	0	0	0	0	0	0	1	0	0	0	0	0	0	0	0	0	0	0	0	0	0	0	0	0	0	0	0	0	0	Fungi; Ascomycota; Eurotiomycetes; Eurotiales; Trichocomaceae; unidentified; Trichocomaceae_sp
New.ReferenceOTU5939	0	0	0	0	0	3	6	1	0	0	0	0	0	0	0	0	0	0	0	0	0	0	0	0	0	0	1	0	0	0	Fungi; Ascomycota; Eurotiomycetes; Eurotiales; Trichocomaceae; unidentified; Trichocomaceae_sp
New.ReferenceOTU6129	0	0	1	0	0	0	0	1	0	0	0	0	0	0	0	0	0	0	0	0	0	0	2	0	0	0	0	0	0	0	Fungi; Ascomycota; Eurotiomycetes; Eurotiales; Trichocomaceae; unidentified; Trichocomaceae_sp
New.ReferenceOTU625	0	0	0	0	0	0	0	0	0	0	0	0	0	4	0	0	0	0	0	0	8	0	1	0	0	0	0	7	0	0	Fungi; Ascomycota; Eurotiomycetes; Eurotiales; Trichocomaceae; unidentified; Trichocomaceae_sp
New.ReferenceOTU6675	0	0	0	0	0	0	0	0	0	0	0	0	0	0	0	0	0	0	0	0	0	0	0	0	0	0	1	0	0	0	Fungi; Ascomycota; Eurotiomycetes; Eurotiales; Trichocomaceae; unidentified; Trichocomaceae_sp
New.ReferenceOTU6861	0	0	0	0	0	1	0	0	0	1	0	0	0	0	0	0	0	0	0	0	0	0	0	0	0	0	0	0	0	0	Fungi; Ascomycota; Eurotiomycetes; Eurotiales; Trichocomaceae; unidentified; Trichocomaceae_sp
New.ReferenceOTU7115	0	0	0	0	1	0	2	0	0	0	0	1	0	0	0	0	0	0	0	0	0	0	0	0	0	0	0	0	0	0	Fungi; Ascomycota; Eurotiomycetes; Eurotiales; Trichocomaceae; unidentified; Trichocomaceae_sp
New.ReferenceOTU7177	0	0	2	0	1	4	0	4	0	0	0	6	6	0	1	0	0	0	0	0	0	0	2	0	0	0	10	0	0	0	Fungi; Ascomycota; Eurotiomycetes; Eurotiales; Trichocomaceae; unidentified; Trichocomaceae_sp
New.ReferenceOTU7218	0	0	0	0	0	0	1	0	0	0	0	0	0	0	0	0	0	0	0	0	0	0	0	0	0	0	0	1	0	0	Fungi; Ascomycota; Eurotiomycetes; Eurotiales; Trichocomaceae; unidentified; Trichocomaceae_sp
New.ReferenceOTU7326	0	0	0	0	0	1	0	0	0	0	0	0	0	0	0	0	0	0	0	0	0	0	0	0	0	0	0	0	0	0	Fungi; Ascomycota; Eurotiomycetes; Eurotiales; Trichocomaceae; unidentified; Trichocomaceae_sp
New.ReferenceOTU7645	0	0	0	0	0	0	0	0	0	0	0	0	0	0	0	0	0	0	0	0	0	0	0	2	0	0	0	0	0	0	Fungi; Ascomycota; Eurotiomycetes; Eurotiales; Trichocomaceae; unidentified; Trichocomaceae_sp
New.ReferenceOTU7693	0	0	0	0	0	0	0	0	0	0	3	0	0	0	0	0	0	0	0	0	0	0	0	0	0	0	0	0	0	0	Fungi; Ascomycota; Eurotiomycetes; Eurotiales; Trichocomaceae; unidentified; Trichocomaceae_sp
New.ReferenceOTU7760	0	0	1	0	0	0	0	0	0	0	0	1	0	0	0	0	0	0	0	0	0	0	0	0	0	0	0	0	0	0	Fungi; Ascomycota; Eurotiomycetes; Eurotiales; Trichocomaceae; unidentified; Trichocomaceae_sp
New.ReferenceOTU7841	0	0	0	0	1	0	0	0	0	0	0	0	0	0	0	0	0	0	0	0	0	0	0	0	0	0	0	0	0	0	Fungi; Ascomycota; Eurotiomycetes; Eurotiales; Trichocomaceae; unidentified; Trichocomaceae_sp
New.ReferenceOTU7854	0	0	0	0	0	0	0	0	0	0	0	1	0	0	0	0	0	0	0	0	0	0	0	0	0	0	0	0	0	0	Fungi; Ascomycota; Eurotiomycetes; Eurotiales; Trichocomaceae; unidentified; Trichocomaceae_sp
SH014683.06FU_DQ778916_reps_singleton	0	3	0	0	0	0	0	0	0	0	0	0	0	0	0	0	0	0	0	0	0	0	0	0	0	0	0	0	0	0	Fungi; Ascomycota; Eurotiomycetes; Eurotiales; Trichocomaceae; unidentified; Trichocomaceae_sp

SH016733.06FU_JN713910_reps_singleton	0	0	1	0	0	0	0	0	0	0	0	0	0	0	0	0	0	0	0	0	0	0	0	0	0	0	0	1	0	0	Fungi; Ascomycota; Eurotiomycetes; Eurotiales; Trichocomaceae; unidentified; Trichocomaceae_sp
SH026208.06FU_AY533699_reps_singleton	0	0	0	0	0	0	0	0	0	0	0	0	0	0	0	0	0	0	0	0	0	0	0	0	0	0	0	0	0	1	Fungi; Ascomycota; Eurotiomycetes; Eurotiales; Trichocomaceae; unidentified; Trichocomaceae_sp
SH096863.06FU_AB250779_refs	1	0	1	0	0	2	1	1	0	1	0	5	0	1	0	0	0	0	0	0	0	1	1	0	1	0	2	0	0	3	Fungi; Ascomycota; Eurotiomycetes; Eurotiales; Trichocomaceae; unidentified; Trichocomaceae_sp
SH096864.06FU_JX021685_refs	0	0	0	0	0	0	2	0	0	0	0	0	0	0	1	0	0	0	0	0	0	0	0	0	0	0	2	1	0	1	Fungi; Ascomycota; Eurotiomycetes; Eurotiales; Trichocomaceae; unidentified; Trichocomaceae_sp
SH096865.06FU_EF669936_reps	0	0	0	0	0	0	0	0	0	0	0	0	0	0	0	0	0	0	0	0	0	0	0	0	0	0	2	0	0	0	Fungi; Ascomycota; Eurotiomycetes; Eurotiales; Trichocomaceae; unidentified; Trichocomaceae_sp
SH096869.06FU_JN246063_reps	0	0	0	0	0	0	0	0	0	0	0	1	0	0	0	0	0	0	0	0	0	0	0	0	0	0	0	0	0	0	Fungi; Ascomycota; Eurotiomycetes; Eurotiales; Trichocomaceae; unidentified; Trichocomaceae_sp
SH109957.06FU_FJ904849_reps	0	1	0	0	1	0	1	0	0	0	0	1	0	0	0	0	0	0	0	0	0	0	0	2	0	0	0	0	0	0	Fungi; Ascomycota; Eurotiomycetes; Eurotiales; Trichocomaceae; unidentified; Trichocomaceae_sp
SH109961.06FU_JN709041_reps	0	0	0	0	0	0	0	0	0	0	0	0	0	0	0	0	0	0	0	0	0	0	0	0	0	0	0	1	0	0	Fungi; Ascomycota; Eurotiomycetes; Eurotiales; Trichocomaceae; unidentified; Trichocomaceae_sp
SH109972.06FU_GU396536_reps	0	3	0	0	1	0	0	0	0	0	1	0	0	0	0	0	0	0	0	0	0	0	0	0	0	0	0	0	0	0	Fungi; Ascomycota; Eurotiomycetes; Eurotiales; Trichocomaceae; unidentified; Trichocomaceae_sp
SH109981.06FU_JN899343_refs	0	3	2	0	0	0	0	0	0	0	4	0	0	0	0	0	0	1	0	0	0	0	3	2	0	0	0	0	0	0	Fungi; Ascomycota; Eurotiomycetes; Eurotiales; Trichocomaceae; unidentified; Trichocomaceae_sp
SH110778.06FU_KC113290_reps	0	0	0	0	0	0	0	0	0	0	0	0	0	0	0	0	0	0	0	0	0	0	2	0	0	0	0	0	0	0	Fungi; Ascomycota; Eurotiomycetes; Eurotiales; Trichocomaceae; unidentified; Trichocomaceae_sp
SH112358.06FU_AY373941_reps	0	0	0	0	0	0	4	1	0	0	0	1	0	0	0	0	0	0	0	0	0	0	0	0	0	0	0	0	0	3	Fungi; Ascomycota; Eurotiomycetes; Eurotiales; Trichocomaceae; unidentified; Trichocomaceae_sp
SH112359.06FU_EU037056_reps	0	0	0	0	0	0	0	0	0	0	0	0	0	0	0	0	0	0	0	0	0	0	0	0	0	0	2	0	0	0	Fungi; Ascomycota; Eurotiomycetes; Eurotiales; Trichocomaceae; unidentified; Trichocomaceae_sp
SH112360.06FU_KC157702_reps	0	0	0	0	0	0	2	0	0	0	0	0	0	0	0	0	0	0	0	0	0	0	0	0	0	0	0	0	0	1	Fungi; Ascomycota; Eurotiomycetes; Eurotiales; Trichocomaceae; unidentified; Trichocomaceae_sp
SH113582.06FU_JX174116_reps	0	0	1	0	0	0	0	0	0	0	0	0	0	0	0	0	0	0	0	0	0	0	0	0	0	0	0	0	0	0	Fungi; Ascomycota; Eurotiomycetes; Eurotiales; Trichocomaceae; unidentified; Trichocomaceae_sp
SH114764.06FU_AB813154_reps	0	0	0	0	0	2	3	0	0	6	0	6	1	1	0	0	0	0	0	0	0	0	0	0	2	0	3	2	0	2	Fungi; Ascomycota; Eurotiomycetes; Eurotiales; Trichocomaceae; unidentified; Trichocomaceae_sp
SH114772.06FU_EF669947_reps	0	0	0	0	0	1	0	0	0	2	0	0	0	1	0	0	0	0	0	0	0	0	0	0	2	0	0	0	0	0	Fungi; Ascomycota; Eurotiomycetes; Eurotiales; Trichocomaceae; unidentified; Trichocomaceae_sp
SH114776.06FU_EU982016_reps	0	0	0	0	0	0	0	1	0	0	0	0	0	1	0	0	0	0	0	0	0	0	0	0	0	0	0	0	0	0	Fungi; Ascomycota; Eurotiomycetes; Eurotiales; Trichocomaceae; unidentified; Trichocomaceae_sp
SH128758.06FU_HQ392492_reps	0	0	0	0	1	0	0	0	0	0	0	2	0	0	1	0	0	1	0	0	0	1	1	0	0	0	0	1	0	0	Fungi; Ascomycota; Eurotiomycetes; Eurotiales; Trichocomaceae; unidentified; Trichocomaceae_sp
SH128766.06FU_KF305753_reps	0	0	1	0	0	0	0	0	0	0	0	0	0	0	1	0	0	0	1	0	0	0	1	0	0	1	0	2	0	0	Fungi; Ascomycota; Eurotiomycetes; Eurotiales; Trichocomaceae; unidentified; Trichocomaceae_sp
SH128777.06FU_JN899334_reps	0	0	0	0	0	0	0	0	0	0	0	0	0	0	0	0	0	0	0	0	0	0	0	0	0	0	1	0	0	0	Fungi; Ascomycota; Eurotiomycetes; Eurotiales; Trichocomaceae; unidentified; Trichocomaceae_sp
SH128778.06FU_EU021596_reps	0	0	0	0	0	2	0	0	1	0	0	5	0	0	0	1	0	0	0	0	1	2	1	0	1	0	0	7	0	1	Fungi; Ascomycota; Eurotiomycetes; Eurotiales; Trichocomaceae; unidentified; Trichocomaceae_sp
SH128786.06FU_KF366489_reps	0	0	5	0	3	4	0	0	0	0	0	8	2	1	2	5	4	1	0	0	7	7	9	0	2	1	3	13	0	5	Fungi; Ascomycota; Eurotiomycetes; Eurotiales; Trichocomaceae; unidentified; Trichocomaceae_sp
SH130728.06FU_EF591743_refs	0	0	0	0	0	0	0	0	0	0	0	0	0	1	1	0	0	0	0	0	0	0	0	0	0	0	0	0	0	0	Fungi; Ascomycota; Eurotiomycetes; Eurotiales; Trichocomaceae; unidentified; Trichocomaceae_sp
SH130729.06FU_EF591736_reps	0	0	0	0	0	0	0	0	0	0	0	0	0	0	0	0	0	0	0	0	0	0	0	0	1	0	0	0	0	0	Fungi; Ascomycota; Eurotiomycetes; Eurotiales; Trichocomaceae; unidentified; Trichocomaceae_sp
SH141139.06FU_AY373849_reps	0	0	0	0	0	1	0	0	0	0	0	0	0	0	0	0	0	0	0	0	0	0	2	0	0	0	0	0	0	0	Fungi; Ascomycota; Eurotiomycetes; Eurotiales; Trichocomaceae; unidentified; Trichocomaceae_sp
New.CleanUp.ReferenceOTU1191	0	0	0	0	1	0	0	0	0	0	0	0	0	0	0	0	0	0	0	0	0	0	0	0	0	0	0	0	0	0	Fungi; Ascomycota; Eurotiomycetes; Eurotiales; Trichocomaceae; Warcupiella; Warcupiella_spinulosa
New.CleanUp.ReferenceOTU715	0	0	0	0	0	1	0	0	0	0	0	0	0	0	0	0	0	0	0	0	0	0	0	0	0	0	0	0	0	0	Fungi; Ascomycota; Eurotiomycetes; Eurotiales; Trichocomaceae; Warcupiella; Warcupiella_spinulosa
New.ReferenceOTU1338	0	0	0	0	0	1	0	0	0	0	0	0	0	0	0	0	0	0	0	0	0	0	0	0	0	0	0	0	0	0	Fungi; Ascomycota; Eurotiomycetes; Eurotiales; Trichocomaceae; Warcupiella; Warcupiella_spinulosa
New.ReferenceOTU4082	0	0	0	0	0	0	0	0	0	0	0	0	0	0	0	0	0	0	0	1	0	0	0	0	0	0	0	0	0	0	Fungi; Ascomycota; Eurotiomycetes; Eurotiales; Trichocomaceae; Warcupiella; Warcupiella_spinulosa
New.ReferenceOTU4761	0	0	0	0	1	0	0	0	0	0	0	0	0	0	0	0	0	0	0	0	0	0	0	0	0	0	0	0	0	0	Fungi; Ascomycota; Eurotiomycetes; Eurotiales; Trichocomaceae; Warcupiella; Warcupiella_spinulosa
New.ReferenceOTU6211	0	0	0	0	3	10	1	0	0	0	0	1	0	0	0	0	0	0	0	0	0	0	0	0	0	0	2	0	0	0	Fungi; Ascomycota; Eurotiomycetes; Eurotiales; Trichocomaceae; Warcupiella; Warcupiella_spinulosa
New.ReferenceOTU6381	0	0	0	0	1	1	0	0	0	0	0	0	0	0	0	0	1	0	0	0	0	0	0	0	0	0	0	0	0	0	Fungi; Ascomycota; Eurotiomycetes; Eurotiales; Trichocomaceae; Warcupiella; Warcupiella_spinulosa
New.ReferenceOTU7678	0	0	0	0	0	3	0	0	0	0	0	0	0	0	0	0	0	0	0	0	0	0	0	0	0	0	0	0	0	4	Fungi; Ascomycota; Eurotiomycetes; Eurotiales; Trichocomaceae; Warcupiella; Warcupiella_spinulosa
SH008155.06FU_EF669706_reps_singleton	0	0	6	7	24	49	4	0	0	0	0	39	26	1	4	0	12	0	0	0	0	1	3	0	3	0	12	12	0	28	Fungi; Ascomycota; Eurotiomycetes; Eurotiales; Trichocomaceae; Warcupiella; Warcupiella_spinulosa
SH008169.06FU_L76807_reps_singleton	0	0	0	0	1	4	0	0	0	0	0	0	0	0	0	0	0	0	0	0	0	0	0	0	0	0	0	0	0	3	Fungi; Ascomycota; Eurotiomycetes; Eurotiales; Trichocomaceae; Warcupiella; Warcupiella_spinulosa
New.ReferenceOTU1181	0	0	0	5	0	5	1	1	0	0	2	0	0	1	2	0	0	0	0	0	1	4	7	0	0	0	3	5	0	1	Fungi; Ascomycota; Eurotiomycetes; Eurotiales; unidentified; unidentified; Eurotiales_sp
New.ReferenceOTU1643	0	0	3	0	1	0	2	0	1	6	0	1	0	0	2	1	1	0	0	0	1	0	0	0	0	0	0	0	0	2	Fungi; Ascomycota; Eurotiomycetes; Eurotiales; unidentified; unidentified; Eurotiales_sp
New.ReferenceOTU1752	0	0	0	0	0	0	0	0	0	0	0	0	1	0	0	0	0	1	0	0	0	0	0	0	0	0	0	0	0	0	Fungi; Ascomycota; Eurotiomycetes; Eurotiales; unidentified; unidentified; Eurotiales_sp
New.ReferenceOTU1943	0	0	0	0	0	1	0	0	0	0	0	0	0	0	0	0	0	0	0	0	0	0	1	0	0	0	0	0	0	0	Fungi; Ascomycota; Eurotiomycetes; Eurotiales; unidentified; unidentified; Eurotiales_sp
New.ReferenceOTU3889	0	1	0	0	0	0	0	0	0	0	0	0	0	0	0	0	0	0	0	0	0	0	1	0	1	0	0	0	1	0	Fungi; Ascomycota; Eurotiomycetes; Eurotiales; unidentified; unidentified; Eurotiales_sp
New.ReferenceOTU5034	1	0	0	0	0	0	0	0	0	0	0	0	0	0	0	0	0	0	0	0	0	0	0	0	0	0	0	0	0	0	Fungi; Ascomycota; Eurotiomycetes; Eurotiales; unidentified; unidentified; Eurotiales_sp
New.ReferenceOTU5607	0	0	0	0	0	0	0	0	0	0	0	0	0	0	0	0	0	0	0	0	0	0	0	0	0	0	0	14	0	0	Fungi; Ascomycota; Eurotiomycetes; Eurotiales; unidentified; unidentified; Eurotiales_sp
New.ReferenceOTU6164	0	0	0	0	0	0	0	0	0	0	0	0	0	0	0	0	0	0	0	1	0	0	0	0	0	0	0	0	0	0	Fungi; Ascomycota; Eurotiomycetes; Eurotiales; unidentified; unidentified; Eurotiales_sp
New.ReferenceOTU6702	0	0	0	0	0	0	0	0	0	0	0	0	0	0	0	0	0	0	0	0	0	4	0	0	0	0	0	0	0	0	Fungi; Ascomycota; Eurotiomycetes; Eurotiales; unidentified; unidentified; Eurotiales_sp
New.ReferenceOTU6747	0	0	0	0	0	0	0	0	0	0	0	0	0	0	0	0	0	0	0	0	0	1	0	0	0	0	0	1	0	0	Fungi; Ascomycota; Eurotiomycetes; Eurotiales; unidentified; unidentified; Eurotiales_sp
New.ReferenceOTU7077	0	0	1	0	0	0	3	0	0	0	0	0	0	0	0	0	0	0	0	0	0	0	0	0	0	0	0	0	0	0	Fungi; Ascomycota; Eurotiomycetes; Eurotiales; unidentified; unidentified; Eurotiales_sp
New.ReferenceOTU878	0	0	0	0	0	0	1	0	0	0	0	0	0	0	0	0	0	0	0	0	0	0	1	0	0	0	0	0	0	0	Fungi; Ascomycota; Eurotiomycetes; Eurotiales; unidentified; unidentified; Eurotiales_sp
New.ReferenceOTU995	0	0	0	0	0	0	0	0	0	3	0	0	0	0	0	0	0	0	0	0	0	0	0	0	0	0	0	0	0	0	Fungi; Ascomycota; Eurotiomycetes; Eurotiales; unidentified; unidentified; Eurotiales_sp
SH008170.06FU_HM036606_reps_singleton	0	0	2	0	0	0	1	0	0	0	1	0	0	0	0	0	1	0	0	0	0	5	0	0	0	0	1	0	0	0	Fungi; Ascomycota; Eurotiomycetes; Eurotiales; unidentified; unidentified; Eurotiales_sp
SH098834.06FU_FJ231021_reps	0	0	4	2	1	4	0	0	1	0	0	0	0	0	0	0	0	0	0	0	1	6	1	0	0	0	3	1	0	1	Fungi; Ascomycota; Eurotiomycetes; Eurotiales; unidentified; unidentified; Eurotiales_sp
SH098861.06FU_JQ965028_reps	0	0	0	6	1	3	0	0	0	0	0	0	0	0	2	0	1	0	0	0	0	0	1	0	0	0	0	1	0	0	Fungi; Ascomycota; Eurotiomycetes; Eurotiales; unidentified; unidentified; Eurotiales_sp
New.CleanUp.ReferenceOTU1793	0	0	0	0	0	0	0	0	0	0	0	0	0	0	0	0	0	0	0	1	0	0	0	0	0	0	0	0	0	0	Fungi; Ascomycota; Eurotiomycetes; Eurotiales; unidentified; unidentified; uncultured_Aspergillus
New.ReferenceOTU2973	0	0	0	0	0	0	0	0	0	0	0	0	0	0	0	0	0	0	3	0	0	0	0	0	0	0	0	0	0	0	Fungi; Ascomycota; Eurotiomycetes; Eurotiales; unidentified; unidentified; uncultured_Eurotiales
New.ReferenceOTU591	0	0	0	0	0	0	0	0	0	0	0	0	0	0	0	0	0	0	1	2	0	0	0	0	0	0	0	0	0	0	Fungi; Ascomycota; Eurotiomycetes; Eurotiales; unidentified; unidentified; uncultured_Eurotiales
New.ReferenceOTU7939	0	0	0	0	0	0	0	0	0	0	0	0	0	0	0	0	0	0	0	0	0	0	0	0	0	0	0	0	1	0	Fungi; Ascomycota; Eurotiomycetes; Eurotiales; unidentified; unidentified; uncultured_Eurotiales
New.ReferenceOTU7936	0	0	0	0	0	0	0	0	0	0	0	1	0	0	1	0	0	0	0	0	0	0	1	0	0	0	0	0	0	0	Fungi; Ascomycota; Eurotiomycetes; Onygenales; Ajellomycetaceae; Ajellomyces; Ajellomyces_capsulatus
New.ReferenceOTU566	0	0	0	0	0	0	0	0	0	0	0	0	1	0	0	0	0	0	0	0	1	0	0	0	0	0	0	0	0	0	Fungi; Ascomycota; Eurotiomycetes; Onygenales; Arthrodermataceae; Trichophyton; Trichophyton_raubitschekii
New.ReferenceOTU1543	0	0	0	1	0	0	0	0	0	0	0	0	0	0	0	0	0	0	0	0	0	0	0	0	0	0	0	0	0	0	Fungi; Ascomycota; Eurotiomycetes; Onygenales; Gymnoascaceae; Gymnascella; Gymnascella_sp_MEXU25095
New.ReferenceOTU5622	0	0	0	0	0	0	0	0	0	0	0	0	0	0	0	0	1	1	0	0	0	0	0	0	0	0	0	0	0	0	Fungi; Ascomycota; Eurotiomycetes; Onygenales; Gymnoascaceae; Gymnascella; Gymnascella_sp_MEXU25095
New.ReferenceOTU2356	0	0	0	0	0	0	0	0	0	0	0	0	0	0	0	0	0	0	9	0	0	0	0	0	0	0	0	0	0	0	Fungi; Ascomycota; Eurotiomycetes; Onygenales; Onygenaceae; Auxarthron; Auxarthron_alboluteum
New.ReferenceOTU5051	0	0	0	0	0	0	0	0	0	0	0	0	0	0	0	0	0	2	0	2	0	0	0	0	0	0	0	0	0	0	Fungi; Ascomycota; Eurotiomycetes; Onygenales; Onygenaceae; Auxarthron; Auxarthron_alboluteum
New.ReferenceOTU4043	0	0	0	0	0	0	0	0	0	0	0	0	2	0	0	0	0	0	0	0	0	0	0	0	0	0	0	0	0	0	Fungi; Ascomycota; Eurotiomycetes; Onygenales; Onygenaceae; Auxarthron; Auxarthron_sp_RV24809
New.ReferenceOTU3141	0	0	0	0	0	0	0	0	0	0	0	0	0	0	0	0	0	0	0	0	1	0	0	0	0	0	0	0	0	0	Fungi; Ascomycota; Eurotiomycetes; Onygenales; Onygenaceae; Chrysosporium; Chrysosporium_lucknowense
SH110882.06FU_AJ131682_reps	0	0	0	0	0	0	0	0	0	0	0	0	0	0	0	0	0	0	0	0	1	0	0	0	0	0	0	0	0	0	Fungi; Ascomycota; Eurotiomycetes; Onygenales; Onygenaceae; Chrysosporium; Chrysosporium_lucknowense
New.ReferenceOTU3305	0	0	0	0	0	0	0	0	0	0	0	0	0	0	0	0	0	0	0	1	0	0	0	0	0	0	0	0	0	0	Fungi; Ascomycota; Eurotiomycetes; Onygenales; Onygenaceae; Onygena; Onygena_equina
New.ReferenceOTU6098	0	0	0	0	0	0	0	0	0	1	0	0	0	0	0	0	0	0	0	0	0	0	0	0	0	0	0	0	0	0	Fungi; Ascomycota; Incertae_sedis; Incertae_sedis; Incertae_sedis; Campylospora; Campylospora_sp_ML_2012
New.ReferenceOTU4576	0	0	0	0	0	0	0	0	2	0	0	0	0	0	0	0	0	0	0	0	0	0	0	0	0	0	0	0	0	0	Fungi; Ascomycota; Incertae_sedis; Incertae_sedis; Incertae_sedis; Dendrosporium; Dendrosporium_sp_ICMP14921
New.ReferenceOTU2245	0	0	0	0	0	0	0	0	0	0	0	0	0	0	0	0	0	0	0	0	1	0	0	0	0	0	0	0	0	0	Fungi; Ascomycota; Incertae_sedis; Incertae_sedis; Incertae_sedis; Hansfordia; Hansfordia_pulvinata
New.ReferenceOTU84	0	0	1	2	0	0	0	0	0	0	0	0	1	0	0	1	1	0	0	0	0	0	0	0	0	0	0	0	0	0	Fungi; Ascomycota; Incertae_sedis; Incertae_sedis; Incertae_sedis; Hansfordia; Hansfordia_pulvinata
SH122649.06FU_AY908995_reps	0	0	2	0	0	0	0	0	0	0	0	0	0	1	0	1	1	0	0	0	0	0	0	0	0	0	0	0	0	1	Fungi; Ascomycota; Incertae_sedis; Incertae_sedis; Incertae_sedis; Hansfordia; Hansfordia_pulvinata
New.ReferenceOTU2163	0	0	0	0	0	0	0	0	0	0	0	0	0	0	0	0	0	0	0	0	0	2	0	0	0	0	0	0	0	0	Fungi; Ascomycota; Incertae_sedis; Incertae_sedis; Incertae_sedis; Leptodiscella; Leptodiscella_africana
New.ReferenceOTU1928	0	0	0	0	0	0	0	0	0	0	1	0	0	0	0	0	0	0	0	0	0	0	0	0	0	0	3	0	0	0	Fungi; Ascomycota; Incertae_sedis; Incertae_sedis; Incertae_sedis; Leptodiscella; Leptodiscella_chlamydospora
New.ReferenceOTU2657	0	0	0	0	0	0	0	0	0	0	0	0	0	0	0	1	0	0	0	0	0	0	0	0	0	0	0	0	0	0	Fungi; Ascomycota; Incertae_sedis; Incertae_sedis; Incertae_sedis; Leptodiscella; Leptodiscella_chlamydospora
New.ReferenceOTU4705	0	0	1	0	0	2	1	0	0	0	0	2	8	13	0	1	8	0	0	0	0	0	0	0	0	0	0	0	0	0	Fungi; Ascomycota; Incertae_sedis; Incertae_sedis; Incertae_sedis; Leptodiscella; Leptodiscella_chlamydospora
SH032997.06FU_FR745398_reps_singleton	0	0	0	0	0	0	1	0	0	0	3	0	0	0	0	0	0	0	0	0	0	0	0	0	0	0	0	0	0	0	Fungi; Ascomycota; Incertae_sedis; Incertae_sedis; Incertae_sedis; Leptodiscella; Leptodiscella_chlamydospora
New.ReferenceOTU7341	0	0	0	0	0	0	0	0	0	0	0	0	0	0	0	0	0	0	0	0	0	1	0	0	1	0	0	0	0	0	Fungi; Ascomycota; Incertae_sedis; Incertae_sedis; Incertae_sedis; Leptodiscella; Leptodiscella_sp_F277786
New.ReferenceOTU7908	0	0	0	1	0	2	1	0	1	0	0	0	0	0	1	0	1	0	0	0	0	0	0	0	0	0	0	0	0	0	Fungi; Ascomycota; Incertae_sedis; Incertae_sedis; Incertae_sedis; Lunulospora; Lunulospora_curvula
New.ReferenceOTU3066	0	0	0	0	0	0	0	0	0	0	0	0	0	0	0	0	0	0	1	0	0	0	0	0	0	0	0	0	0	0	Fungi; Ascomycota; Incertae_sedis; Incertae_sedis; Incertae_sedis; Lunulospora; Lunulospora_sp
New.ReferenceOTU7373	0	0	0	0	0	0	0	0	0	0	0	0	0	0	0	0	0	0	0	1	0	0	0	0	0	0	0	0	0	1	Fungi; Ascomycota; Incertae_sedis; Incertae_sedis; Incertae_sedis; Ochroconis; Ochroconis_constricta
New.ReferenceOTU3197	3	0	0	0	1	0	2	0	0	1	1	0	0	0	0	0	0	0	0	0	0	0	0	0	0	0	0	0	0	0	Fungi; Ascomycota; Incertae_sedis; Incertae_sedis; Incertae_sedis; Ochroconis; Ochroconis_sp
New.ReferenceOTU3307	0	0	0	0	0	1	0	0	0	0	0	0	0	0	0	0	0	0	0	0	0	0	0	0	0	0	0	0	0	0	Fungi; Ascomycota; Incertae_sedis; Incertae_sedis; Incertae_sedis; Ochroconis; Ochroconis_sp
New.ReferenceOTU3676	0	0	2	0	0	0	0	0	0	0	2	3	5	2	0	4	1	0	0	0	0	0	0	0	0	0	0	0	0	0	Fungi; Ascomycota; Incertae_sedis; Incertae_sedis; Incertae_sedis; Ochroconis; Ochroconis_sp
New.ReferenceOTU5099	0	0	0	0	0	0	0	0	1	0	0	0	0	0	0	0	0	0	0	0	0	0	0	0	0	0	0	0	0	0	Fungi; Ascomycota; Incertae_sedis; Incertae_sedis; Incertae_sedis; Ochroconis; Ochroconis_sp
New.ReferenceOTU6267	0	0	0	0	0	0	0	0	0	0	0	0	1	0	0	0	0	0	0	0	0	0	0	0	0	0	0	0	0	0	Fungi; Ascomycota; Incertae_sedis; Incertae_sedis; Incertae_sedis; Ochroconis; Ochroconis_sp
New.ReferenceOTU763	0	0	0	0	3	0	0	0	0	0	0	0	0	0	0	0	0	0	0	0	0	0	0	0	0	0	0	0	0	0	Fungi; Ascomycota; Incertae_sedis; Incertae_sedis; Incertae_sedis; Ochroconis; Ochroconis_sp
New.ReferenceOTU7962	0	0	0	1	0	0	0	0	1	0	0	0	0	0	0	0	1	0	0	0	0	0	0	0	0	0	0	0	0	0	Fungi; Ascomycota; Incertae_sedis; Incertae_sedis; Incertae_sedis; Ochroconis; Ochroconis_sp
New.ReferenceOTU6858	0	0	0	6	0	0	0	0	0	0	0	0	0	0	0	0	0	0	0	0	0	0	0	0	0	0	0	0	0	0	Fungi; Ascomycota; Incertae_sedis; Incertae_sedis; Incertae_sedis; Ochroconis; Ochroconis_sp_L1265
SH024942.06FU_AB725393_reps_singleton	0	0	0	0	0	0	0	0	0	0	0	1	0	0	0	0	0	0	0	0	0	0	0	0	0	0	0	0	0	0	Fungi; Ascomycota; Incertae_sedis; Incertae_sedis; Incertae_sedis; Phaeoisaria; Phaeoisaria_sp
New.ReferenceOTU4291	0	0	0	0	0	0	0	0	0	0	0	0	0	0	1	0	0	0	0	0	0	0	0	0	0	0	0	0	0	0	Fungi; Ascomycota; Incertae_sedis; Incertae_sedis; Incertae_sedis; Satchmopsis; Satchmopsis_brasiliensis
New.ReferenceOTU5255	0	0	0	0	0	0	0	0	0	0	0	0	0	1	0	0	0	0	0	0	0	0	0	0	0	0	0	0	0	0	Fungi; Ascomycota; Incertae_sedis; Incertae_sedis; Incertae_sedis; Scolecobasidium; Scolecobasidium_sp_F77
New.ReferenceOTU5665	0	0	0	0	0	0	0	0	0	1	1	0	0	0	0	0	0	0	1	0	0	0	0	0	0	0	0	0	0	1	Fungi; Ascomycota; Incertae_sedis; Incertae_sedis; Incertae_sedis; Staphylotrichum; Staphylotrichum_sp
SH139431.06FU_EU563611_reps	0	0	0	0	0	0	1	0	3	2	4	2	0	0	1	0	0	0	0	1	0	1	1	0	5	0	2	4	0	0	Fungi; Ascomycota; Incertae_sedis; Incertae_sedis; Incertae_sedis; Staphylotrichum; Staphylotrichum_sp
New.ReferenceOTU15	0	0	0	0	0	0	0	0	0	0	0	0	0	0	0	0	0	0	0	2	0	0	0	0	0	0	0	0	0	0	Fungi; Ascomycota; Incertae_sedis; Incertae_sedis; Incertae_sedis; Staphylotrichum; Staphylotrichum_sp_Amy_4
SH139423.06FU_AB728536_reps	0	0	0	0	0	0	0	0	0	0	0	0	0	0	0	0	0	0	0	5	0	2	0	0	3	0	0	1	0	0	Fungi; Ascomycota; Incertae_sedis; Incertae_sedis; Incertae_sedis; Staphylotrichum; Staphylotrichum_sp_Amy_4
New.ReferenceOTU6349	0	0	0	0	0	0	0	0	0	0	0	0	3	0	0	0	0	0	0	0	0	0	0	0	0	0	0	0	0	0	Fungi; Ascomycota; Lecanoromycetes; Ostropales; Odontotremataceae; Claviradulomyces; Claviradulomyces_dabeicola
New.ReferenceOTU1234	1	0	0	0	0	0	0	0	0	0	0	0	0	0	0	0	0	0	0	0	0	0	0	0	0	0	0	0	0	0	Fungi; Ascomycota; Lecanoromycetes; Ostropales; Stictidaceae; Cryptodiscus; Cryptodiscus_sp
SH124376.06FU_AJ877197_reps	0	0	1	0	0	0	0	0	0	0	0	0	0	0	0	0	0	0	0	0	0	0	0	0	0	0	0	0	0	0	Fungi; Ascomycota; Lecanoromycetes; Ostropales; Stictidaceae; Cryptodiscus; Cryptodiscus_sp
New.ReferenceOTU6321	0	0	0	0	0	0	0	0	0	0	0	0	0	0	0	0	0	0	0	1	0	0	0	0	0	0	0	0	0	0	Fungi; Ascomycota; Leotiomycetes; Geoglossales; Geoglossaceae; Geoglossum; Geoglossum_sp
New.ReferenceOTU3494	0	0	0	0	0	0	0	0	0	0	1	0	0	0	0	0	0	0	2	0	0	0	0	0	0	0	0	0	0	0	Fungi; Ascomycota; Leotiomycetes; Helotiales; Helotiaceae; Idriella; Idriella_sp_EXP001F

New.ReferenceOTU5620	0	0	0	0	0	0	0	0	8	0	0	0	0	0	0	0	0	0	1	1	1	2	0	0	0	0	0	0	0	0	Fungi; Ascomycota; Leotiomycetes; Helotiales; Incertae_sedis; Scytalidium; Scytalidium_cuboideum
SH132294.06FU_GQ370375_reps	0	0	0	1	0	1	3	0	1	1	0	2	0	0	0	0	0	7	0	3	0	0	0	0	0	0	0	2	0	0	Fungi; Ascomycota; Leotiomycetes; Helotiales; Incertae_sedis; Scytalidium; Scytalidium_sp
New.ReferenceOTU6131	0	0	0	0	0	0	0	0	0	0	0	0	1	2	0	0	0	0	0	0	0	0	0	0	0	0	0	0	0	0	Fungi; Ascomycota; Leotiomycetes; Helotiales; Incertae_sedis; Spirosphaera; Spirosphaera_beverwijkiana
New.ReferenceOTU6229	0	0	0	0	0	0	0	0	0	0	1	0	0	0	0	0	0	0	0	0	0	0	0	0	0	0	0	0	0	0	Fungi; Ascomycota; Leotiomycetes; Helotiales; Incertae_sedis; Spirosphaera; Spirosphaera_cupreorufescens
New.ReferenceOTU5637	1	0	0	0	0	0	0	0	0	0	0	0	0	0	0	0	0	0	0	0	0	0	0	0	0	0	0	0	0	0	Fungi; Ascomycota; Leotiomycetes; Helotiales; Incertae_sedis; Spirosphaera; Spirosphaera_minuta
New.ReferenceOTU1009	0	0	0	0	2	0	0	0	0	0	0	0	0	0	0	0	0	0	0	0	0	0	0	0	0	0	0	0	0	0	Fungi; Ascomycota; Leotiomycetes; Leotiales; Leotiaceae; Leotia; Leotia_lubrica
New.ReferenceOTU7666	0	0	0	0	0	0	0	0	0	0	1	0	0	0	0	0	0	0	0	0	0	0	0	0	0	0	0	0	0	0	Fungi; Ascomycota; Neolectomycetes; Neolectales; Neolectaceae; Neolecta; Neolecta_vitellina
New.ReferenceOTU363	0	0	2	0	0	0	0	0	0	0	2	0	0	0	0	0	0	0	0	2	0	0	0	0	0	0	0	0	0	0	Fungi; Ascomycota; Orbiliomycetes; Orbiliales; Orbiliaceae; Arthrobotrys; Arthrobotrys_javanica
New.ReferenceOTU3474	0	0	0	0	0	0	0	0	0	0	0	0	0	0	0	0	0	0	0	5	0	0	0	0	0	0	0	0	0	0	Fungi; Ascomycota; Orbiliomycetes; Orbiliales; Orbiliaceae; Arthrobotrys; Arthrobotrys_musiformis
New.ReferenceOTU7379	1	0	0	0	0	0	0	0	0	0	0	0	0	0	0	0	0	0	0	0	0	0	0	0	0	0	0	0	0	0	Fungi; Ascomycota; Orbiliomycetes; Orbiliales; Orbiliaceae; Arthrobotrys; Arthrobotrys_sp_ATCC_MYA_4125
New.ReferenceOTU1302	0	0	0	0	0	0	0	0	0	0	0	0	0	0	0	0	0	0	0	3	0	0	0	0	0	0	0	0	0	0	Fungi; Ascomycota; Orbiliomycetes; Orbiliales; Orbiliaceae; Dactylellina; Dactylellina_drechsleri
New.ReferenceOTU8015	0	0	0	0	0	0	0	0	1	0	0	0	0	0	0	0	0	0	0	0	0	0	0	0	0	0	0	0	0	0	Fungi; Ascomycota; Orbiliomycetes; Orbiliales; Orbiliaceae; Orbilia; Orbilia_sp_XJ_2009
New.ReferenceOTU5827	0	0	0	0	0	0	0	0	0	0	0	0	0	0	0	1	0	0	0	0	0	0	0	0	0	0	0	0	0	0	Fungi; Ascomycota; Orbiliomycetes; Orbiliales; Orbiliaceae; unidentified; Helicoon_gigantisporum
New.ReferenceOTU169	0	0	0	0	1	0	0	0	0	0	0	0	0	0	0	0	0	0	0	0	0	0	0	0	0	0	0	1	1	0	Fungi; Ascomycota; Pezizomycetes; Pezizales; Ascobolaceae; Ascobolus; Ascobolus_sp
New.ReferenceOTU3724	0	0	0	1	0	0	0	0	0	0	0	0	0	0	0	0	0	0	0	0	0	0	0	0	0	0	0	0	0	0	Fungi; Ascomycota; Pezizomycetes; Pezizales; Ascobolaceae; Ascobolus; Ascobolus_sp
New.ReferenceOTU3775	0	0	0	0	0	0	0	3	0	0	0	0	0	0	0	0	1	0	0	0	0	0	0	0	0	0	0	1	0	0	Fungi; Ascomycota; Pezizomycetes; Pezizales; Pyronemataceae; Monascella; Monascella_botryosa
New.ReferenceOTU4049	0	0	0	0	3	0	0	0	0	0	0	0	1	0	0	0	1	0	0	0	0	0	1	0	0	0	0	0	0	0	Fungi; Ascomycota; Pezizomycetes; Pezizales; Pyronemataceae; Scutellinia; Scutellinia_sp_YZ_2012
SH140559.06FU_AF394019_reps	0	0	0	0	0	0	0	0	0	0	0	0	1	0	0	0	0	0	0	0	0	0	0	0	0	0	0	0	0	0	Fungi; Ascomycota; Pezizomycetes; Pezizales; Sarcoscyphaceae; Cookeina; Cookeina_tricholoma
New.ReferenceOTU4726	0	0	0	0	0	0	0	0	0	0	0	0	0	0	0	0	0	0	0	0	0	0	0	0	0	0	0	0	0	2	Fungi; Ascomycota; Saccharomycetes; Saccharomycetales; Debaryomycetaceae; Meyerozyma; Meyerozyma_guilliermondii
SH117961.06FU_EF568003_refs	0	0	0	0	0	0	0	0	0	0	0	1	0	0	0	0	0	0	0	0	0	0	0	0	0	0	0	5	0	15	Fungi; Ascomycota; Saccharomycetes; Saccharomycetales; Debaryomycetaceae; Meyerozyma; Meyerozyma_guilliermondii
SH117972.06FU_JN255494_reps	0	0	0	0	0	0	0	0	0	0	0	0	0	0	0	0	0	0	0	0	0	0	0	0	0	0	0	1	0	1	Fungi; Ascomycota; Saccharomycetes; Saccharomycetales; Debaryomycetaceae; Meyerozyma; Meyerozyma_guilliermondii
New.ReferenceOTU856	0	0	0	0	0	0	0	0	1	0	0	0	0	0	0	0	0	0	0	0	0	0	0	0	0	0	0	0	0	0	Fungi; Ascomycota; Saccharomycetes; Saccharomycetales; Incertae_sedis; Candida; Candida_castellii
New.ReferenceOTU7772	0	0	0	0	0	0	0	0	0	0	0	0	0	0	0	0	0	0	1	0	0	0	0	0	0	0	0	0	0	0	Fungi; Ascomycota; Saccharomycetes; Saccharomycetales; Incertae_sedis; Candida; Candida_dendrica
New.ReferenceOTU979	0	0	0	0	0	0	0	0	0	0	0	0	0	0	1	0	0	0	0	0	0	0	0	0	0	0	0	0	0	0	Fungi; Ascomycota; Saccharomycetes; Saccharomycetales; Incertae_sedis; Candida; Candida_polysorbophila
New.ReferenceOTU2081	0	0	0	0	0	0	0	0	0	0	0	0	0	0	0	0	0	0	4	0	0	0	0	0	0	0	0	0	0	0	Fungi; Ascomycota; Saccharomycetes; Saccharomycetales; Incertae_sedis; Candida; Candida_sp_EN28S10
SH459112.06FU_FJ873408_reps_singleton	0	0	0	0	0	0	0	0	0	0	0	0	0	0	0	0	0	0	1	0	0	0	0	0	0	0	0	0	0	0	Fungi; Ascomycota; Saccharomycetes; Saccharomycetales; Incertae_sedis; Candida; Candida_sp_EN28S10
New.ReferenceOTU2685	0	0	0	0	0	0	0	0	0	0	0	0	0	0	0	0	0	1	0	0	0	0	0	0	0	0	0	0	0	0	Fungi; Ascomycota; Saccharomycetes; Saccharomycetales; Incertae_sedis; Candida; Candida_sp_Gy44SO2
New.ReferenceOTU49	0	0	0	0	0	0	0	0	0	1	0	0	0	0	0	0	0	0	0	0	0	0	0	0	0	0	0	1	0	0	Fungi; Ascomycota; Saccharomycetes; Saccharomycetales; Incertae_sedis; Candida; Candida_sp_HNX35_3
New.ReferenceOTU1910	0	0	0	0	0	0	0	0	0	0	0	0	0	0	0	0	1	0	0	0	0	0	0	0	0	0	0	0	0	0	Fungi; Ascomycota; Saccharomycetes; Saccharomycetales; Incertae_sedis; Candida; Candida_sp_LF_W28
New.ReferenceOTU3466	0	0	0	0	0	0	0	0	0	0	0	0	0	0	0	0	0	0	0	0	0	0	1	0	0	0	0	0	0	0	Fungi; Ascomycota; Saccharomycetes; Saccharomycetales; Incertae_sedis; Candida; Candida_sp_VVT_2012
New.ReferenceOTU7650	0	0	0	0	0	0	0	0	0	0	0	0	0	0	0	0	1	0	0	0	0	0	0	0	0	0	0	0	0	0	Fungi; Ascomycota; Saccharomycetes; Saccharomycetales; Incertae_sedis; Spathaspora; Spathaspora_sp_UFMG_IO_271
New.ReferenceOTU6884	0	0	0	0	0	0	0	0	0	0	1	0	0	0	0	0	0	0	0	0	0	0	0	0	0	0	0	0	0	0	Fungi; Ascomycota; Saccharomycetes; Saccharomycetales; Lipomycetaceae; Lipomyces; Lipomyces_kononenkoae
SH095463.06FU_AB747653_reps	0	0	0	0	0	0	0	0	0	0	0	0	0	2	0	0	0	0	0	0	0	0	1	0	0	0	0	1	0	0	Fungi; Ascomycota; Saccharomycetes; Saccharomycetales; Lipomycetaceae; Lipomyces; Lipomyces_kononenkoae
New.ReferenceOTU1436	0	0	0	0	0	0	0	0	0	0	0	0	0	0	0	0	0	0	0	0	0	0	0	0	0	0	0	0	1	0	Fungi; Ascomycota; Saccharomycetes; Saccharomycetales; Lipomycetaceae; Lipomyces; Lipomyces_starkeyi
New.ReferenceOTU2199	0	0	0	0	0	0	0	0	0	0	0	0	0	0	3	0	0	0	0	0	0	0	1	0	14	0	0	0	0	0	Fungi; Ascomycota; Saccharomycetes; Saccharomycetales; Lipomycetaceae; Lipomyces; Lipomyces_yarrowii
New.ReferenceOTU3365	0	0	0	0	0	0	0	0	0	0	0	0	0	0	148	0	0	0	0	0	2	5	46	0	53	0	8	0	0	4	Fungi; Ascomycota; Saccharomycetes; Saccharomycetales; Lipomycetaceae; Lipomyces; Lipomyces_yarrowii
SH095460.06FU_JN943154_reps	0	0	0	0	0	0	0	0	0	0	0	0	0	0	0	0	0	2	0	0	1	0	0	0	0	0	0	0	0	0	Fungi; Ascomycota; Saccharomycetes; Saccharomycetales; Lipomycetaceae; Lipomyces; Lipomyces_yarrowii
SH095466.06FU_NR_103689_reps	0	0	0	0	0	0	0	0	0	0	0	0	0	0	0	0	0	0	0	0	0	0	0	0	2	0	0	0	0	1	Fungi; Ascomycota; Saccharomycetes; Saccharomycetales; Lipomycetaceae; Lipomyces; Lipomyces_yarrowii
New.ReferenceOTU1086	0	0	0	0	0	0	0	0	0	0	0	0	0	0	0	0	0	1	0	0	0	0	0	0	0	0	0	0	0	0	Fungi; Ascomycota; Saccharomycetes; Saccharomycetales; Trichomonascaceae; Blastobotrys; Blastobotrys_mokoenaii
New.ReferenceOTU1984	0	0	1	0	0	0	0	0	0	0	0	0	0	0	0	0	0	0	0	0	0	0	0	0	0	0	0	0	0	0	Fungi; Ascomycota; Saccharomycetes; Saccharomycetales; Trichomonascaceae; Blastobotrys; Blastobotrys_sp_IMBZ21
New.ReferenceOTU3767	0	1	0	1	0	0	0	0	0	0	0	0	0	0	0	0	0	0	0	0	0	0	0	0	0	0	0	0	0	0	Fungi; Ascomycota; Saccharomycetes; Saccharomycetales; Trichomonascaceae; Sugiyamaella; Sugiyamaella_marionensis
New.ReferenceOTU3374	0	0	0	0	0	0	0	0	0	0	0	0	0	0	0	0	0	0	0	0	0	0	0	0	0	0	0	0	2	0	Fungi; Ascomycota; Saccharomycetes; Saccharomycetales; Trichomonascaceae; Sugiyamaella; Sugiyamaella_paludigena
New.ReferenceOTU1117	0	0	0	0	0	0	0	0	0	0	0	0	0	0	0	0	0	0	0	0	0	0	0	0	0	1	0	0	4	0	Fungi; Ascomycota; Saccharomycetes; Saccharomycetales; unidentified; unidentified; Saccharomycetales_sp
New.ReferenceOTU2243	0	4	0	0	0	0	0	0	0	0	0	0	0	0	0	0	0	0	0	0	0	0	0	0	0	1	0	0	5	0	Fungi; Ascomycota; Saccharomycetes; Saccharomycetales; unidentified; unidentified; Saccharomycetales_sp
New.ReferenceOTU2396	0	0	0	0	0	0	0	0	0	0	0	0	0	0	0	0	0	0	0	0	0	0	0	0	0	6	0	0	10	0	Fungi; Ascomycota; Saccharomycetes; Saccharomycetales; unidentified; unidentified; Saccharomycetales_sp
New.ReferenceOTU2593	0	3	0	0	0	0	0	0	0	0	0	0	0	0	0	0	0	0	0	0	0	0	0	0	0	0	0	0	0	0	Fungi; Ascomycota; Saccharomycetes; Saccharomycetales; unidentified; unidentified; Saccharomycetales_sp
New.ReferenceOTU3313	0	0	0	0	1	0	0	0	0	0	0	0	0	0	0	0	0	0	0	0	0	0	0	0	0	0	0	0	0	0	Fungi; Ascomycota; Saccharomycetes; Saccharomycetales; unidentified; unidentified; Saccharomycetales_sp
New.ReferenceOTU3928	0	1	0	0	0	0	0	0	0	0	0	0	0	0	0	0	0	1	0	0	0	0	0	0	0	0	0	0	1	0	Fungi; Ascomycota; Saccharomycetes; Saccharomycetales; unidentified; unidentified; Saccharomycetales_sp
New.ReferenceOTU5431	3	0	0	0	0	0	0	0	0	0	0	0	0	0	0	0	0	0	0	0	0	0	0	0	0	0	0	0	0	0	Fungi; Ascomycota; Saccharomycetes; Saccharomycetales; unidentified; unidentified; Saccharomycetales_sp
SH023473.06FU_AY969855_reps_singleton	0	1	2	0	0	0	0	0	0	0	0	0	0	0	0	0	0	1	0	0	0	0	0	0	0	0	0	0	2	0	Fungi; Ascomycota; Saccharomycetes; Saccharomycetales; unidentified; unidentified; Saccharomycetales_sp
New.ReferenceOTU1520	0	0	0	0	0	0	0	0	0	0	0	0	0	1	0	0	0	0	0	0	0	0	0	0	0	0	0	0	0	0	Fungi; Ascomycota; Sordariomycetes; Boliniales; Boliniaceae; Camarops; Camarops_sp_W2_6
New.ReferenceOTU3204	0	0	0	0	0	2	0	0	0	0	0	0	0	0	0	0	2	0	0	0	0	0	0	0	0	0	0	0	0	0	Fungi; Ascomycota; Sordariomycetes; Boliniales; Boliniaceae; Camarops; Camarops_sp_W2_6
New.ReferenceOTU2687	0	0	0	0	0	0	0	0	0	0	1	0	0	0	0	0	0	0	0	0	0	0	0	0	0	0	0	0	0	0	Fungi; Ascomycota; Sordariomycetes; Boliniales; Boliniaceae; Endoxyla; Endoxyla_sp
New.ReferenceOTU3042	0	0	0	0	0	0	0	0	0	0	0	0	0	0	0	0	0	0	0	0	0	0	0	0	0	0	0	1	0	0	Fungi; Ascomycota; Sordariomycetes; Boliniales; Boliniaceae; Endoxyla; Endoxyla_sp
New.ReferenceOTU3491	2	1	0	3	0	6	1	1	0	15	1	0	6	2	1	0	4	3	0	0	1	0	0	4	0	0	11	3	0	7	Fungi; Ascomycota; Sordariomycetes; Boliniales; Boliniaceae; Endoxyla; Endoxyla_sp
New.ReferenceOTU7784	0	0	0	0	0	0	1	1	0	0	0	1	0	0	0	0	0	0	0	0	0	0	0	0	0	0	0	11	0	1	Fungi; Ascomycota; Sordariomycetes; Boliniales; Boliniaceae; Endoxyla; Endoxyla_sp
New.ReferenceOTU4623	0	0	0	0	0	0	0	0	2	0	3	0	0	0	0	0	2	0	0	0	0	0	0	0	0	0	0	0	0	0	Fungi; Ascomycota; Sordariomycetes; Chaetosphaeriales; Chaetosphaeriaceae; Chaetosphaeria; Chaetosphaeria_dilabens
New.ReferenceOTU6569	0	0	0	0	0	0	0	0	0	0	0	0	0	0	0	0	0	2	0	0	0	0	0	0	0	0	0	0	0	0	Fungi; Ascomycota; Sordariomycetes; Chaetosphaeriales; Chaetosphaeriaceae; Chaetosphaeria; Chaetosphaeria_dilabens
New.ReferenceOTU7934	0	0	0	3	0	0	0	4	0	1	0	0	1	3	2	0	1	0	0	0	0	0	0	0	0	0	0	0	0	0	Fungi; Ascomycota; Sordariomycetes; Chaetosphaeriales; Chaetosphaeriaceae; Chaetosphaeria; Chaetosphaeria_dilabens
New.ReferenceOTU6908	0	0	0	0	0	0	0	0	0	1	0	0	0	0	0	0	0	0	0	0	0	0	0	0	0	0	0	0	0	0	Fungi; Ascomycota; Sordariomycetes; Chaetosphaeriales; Chaetosphaeriaceae; Chaetosphaeria; Chaetosphaeria_fennica
New.ReferenceOTU6620	1	0	0	0	0	0	0	0	0	0	0	0	0	0	0	0	0	0	0	0	0	0	0	0	0	0	0	0	0	0	Fungi; Ascomycota; Sordariomycetes; Chaetosphaeriales; Chaetosphaeriaceae; Chaetosphaeria; Chaetosphaeria_fusiformis
New.ReferenceOTU1381	0	0	0	0	0	0	0	0	0	0	0	0	0	0	0	0	0	0	2	0	0	0	0	0	0	0	0	0	0	0	Fungi; Ascomycota; Sordariomycetes; Chaetosphaeriales; Chaetosphaeriaceae; Chaetosphaeria; Chaetosphaeria_hebetiseta
New.ReferenceOTU6977	0	0	0	0	0	0	0	0	0	0	0	0	0	0	0	0	0	0	0	0	0	0	0	0	0	0	0	1	0	0	Fungi; Ascomycota; Sordariomycetes; Chaetosphaeriales; Chaetosphaeriaceae; Chaetosphaeria; Chaetosphaeria_hebetiseta
New.ReferenceOTU2994	0	0	0	0	0	0	0	0	2	0	0	0	0	0	0	0	0	0	0	0	0	0	0	0	0	0	0	0	0	0	Fungi; Ascomycota; Sordariomycetes; Chaetosphaeriales; Chaetosphaeriaceae; Chaetosphaeria; Chaetosphaeria_innumera
New.ReferenceOTU3694	0	0	0	0	0	0	0	0	0	0	1	0	0	0	0	0	0	0	0	0	0	0	0	0	0	0	0	0	0	0	Fungi; Ascomycota; Sordariomycetes; Chaetosphaeriales; Chaetosphaeriaceae; Chaetosphaeria; Chaetosphaeria_innumera
New.ReferenceOTU3851	0	0	0	0	0	0	0	0	0	0	0	0	0	0	0	0	0	0	0	0	0	0	0	1	0	0	0	0	0	1	Fungi; Ascomycota; Sordariomycetes; Chaetosphaeriales; Chaetosphaeriaceae; Chaetosphaeria; Chaetosphaeria_innumera
New.ReferenceOTU3910	0	0	0	0	0	0	0	0	0	0	0	0	0	0	0	0	0	0	0	0	0	0	0	0	0	0	0	0	0	2	Fungi; Ascomycota; Sordariomycetes; Chaetosphaeriales; Chaetosphaeriaceae; Chaetosphaeria; Chaetosphaeria_innumera
New.ReferenceOTU4484	0	0	0	0	0	0	0	1	0	0	0	0	0	0	0	0	0	0	0	0	0	0	0	0	0	0	0	0	1	0	Fungi; Ascomycota; Sordariomycetes; Chaetosphaeriales; Chaetosphaeriaceae; Chaetosphaeria; Chaetosphaeria_innumera
New.ReferenceOTU4847	0	0	1	0	0	0	0	0	0	0	0	2	0	0	0	0	0	0	0	0	0	0	0	0	0	0	0	0	0	0	Fungi; Ascomycota; Sordariomycetes; Chaetosphaeriales; Chaetosphaeriaceae; Chaetosphaeria; Chaetosphaeria_innumera
New.ReferenceOTU6387	0	0	0	0	0	0	0	0	0	0	0	0	0	0	0	0	0	0	0	0	0	0	0	0	0	0	0	0	0	31	Fungi; Ascomycota; Sordariomycetes; Chaetosphaeriales; Chaetosphaeriaceae; Chaetosphaeria; Chaetosphaeria_innumera
New.ReferenceOTU7687	0	0	0	0	0	0	0	0	0	0	0	0	0	0	0	0	0	0	0	0	0	1	0	0	1	0	0	0	0	0	Fungi; Ascomycota; Sordariomycetes; Chaetosphaeriales; Chaetosphaeriaceae; Chaetosphaeria; Chaetosphaeria_innumera
New.ReferenceOTU1783	1	0	0	0	0	0	0	0	0	0	0	1	0	0	0	0	0	0	0	0	0	0	0	0	0	0	0	0	0	0	Fungi; Ascomycota; Sordariomycetes; Chaetosphaeriales; Chaetosphaeriaceae; Chaetosphaeria; Chaetosphaeria_lignomollis
New.ReferenceOTU1012	0	0	0	0	0	0	0	0	0	0	0	0	0	0	0	0	0	0	0	0	4	0	0	0	0	0	0	0	0	0	Fungi; Ascomycota; Sordariomycetes; Chaetosphaeriales; Chaetosphaeriaceae; Chaetosphaeria; Chaetosphaeria_sp
New.ReferenceOTU1087	0	0	0	0	1	0	0	0	0	0	0	0	0	0	0	0	0	0	0	0	0	0	0	0	0	0	0	0	0	0	Fungi; Ascomycota; Sordariomycetes; Chaetosphaeriales; Chaetosphaeriaceae; Chaetosphaeria; Chaetosphaeria_sp
New.ReferenceOTU1828	0	0	1	0	0	0	0	0	0	0	0	0	0	0	0	0	0	0	0	0	0	0	0	0	0	0	0	0	0	0	Fungi; Ascomycota; Sordariomycetes; Chaetosphaeriales; Chaetosphaeriaceae; Chaetosphaeria; Chaetosphaeria_sp
New.ReferenceOTU2841	0	0	0	0	0	0	0	0	0	4	0	0	0	0	0	0	0	0	1	0	0	0	0	0	0	0	0	0	0	0	Fungi; Ascomycota; Sordariomycetes; Chaetosphaeriales; Chaetosphaeriaceae; Chaetosphaeria; Chaetosphaeria_sp
New.ReferenceOTU3584	0	0	0	0	6	0	0	0	2	3	0	0	1	0	0	0	0	0	0	0	0	0	0	0	0	0	0	0	0	0	Fungi; Ascomycota; Sordariomycetes; Chaetosphaeriales; Chaetosphaeriaceae; Chaetosphaeria; Chaetosphaeria_sp
New.ReferenceOTU4187	0	0	0	0	0	0	0	0	0	0	0	0	0	0	0	0	0	0	0	1	0	0	0	0	1	0	0	0	0	0	Fungi; Ascomycota; Sordariomycetes; Chaetosphaeriales; Chaetosphaeriaceae; Chaetosphaeria; Chaetosphaeria_sp
New.ReferenceOTU4336	9	1	4	1	0	0	0	0	4	1	4	1	0	0	0	0	0	5	1	1	0	0	0	2	1	0	3	0	1	0	Fungi; Ascomycota; Sordariomycetes; Chaetosphaeriales; Chaetosphaeriaceae; Chaetosphaeria; Chaetosphaeria_sp
New.ReferenceOTU4855	0	0	0	0	0	0	0	0	0	0	0	0	0	0	0	0	0	4	0	1	0	0	0	0	0	0	0	0	0	0	Fungi; Ascomycota; Sordariomycetes; Chaetosphaeriales; Chaetosphaeriaceae; Chaetosphaeria; Chaetosphaeria_sp
New.ReferenceOTU5256	0	0	0	0	0	0	0	0	1	0	0	0	0	0	0	0	0	0	0	0	0	0	0	0	0	0	0	0	0	0	Fungi; Ascomycota; Sordariomycetes; Chaetosphaeriales; Chaetosphaeriaceae; Chaetosphaeria; Chaetosphaeria_sp
New.ReferenceOTU5638	0	0	2	0	1	0	0	0	0	0	0	0	0	0	0	0	0	0	0	0	0	0	0	0	0	0	0	0	0	0	Fungi; Ascomycota; Sordariomycetes; Chaetosphaeriales; Chaetosphaeriaceae; Chaetosphaeria; Chaetosphaeria_sp
New.ReferenceOTU5836	0	0	1	0	0	0	0	0	7	7	0	0	2	0	0	8	3	1	0	2	0	0	3	0	5	0	2	0	0	0	Fungi; Ascomycota; Sordariomycetes; Chaetosphaeriales; Chaetosphaeriaceae; Chaetosphaeria; Chaetosphaeria_sp
New.ReferenceOTU6060	0	0	0	0	0	0	0	0	0	0	0	0	0	0	0	0	0	0	1	0	0	0	0	0	0	0	0	0	0	0	Fungi; Ascomycota; Sordariomycetes; Chaetosphaeriales; Chaetosphaeriaceae; Chaetosphaeria; Chaetosphaeria_sp
New.ReferenceOTU6315	0	0	0	0	0	0	0	1	14	2	2	0	0	0	0	0	0	0	0	8	0	5	0	0	0	3	0	1	0	2	Fungi; Ascomycota; Sordariomycetes; Chaetosphaeriales; Chaetosphaeriaceae; Chaetosphaeria; Chaetosphaeria_sp
New.ReferenceOTU7113	0	0	1	0	0	0	0	0	3	0	0	0	0	0	0	0	0	0	0	0	0	0	0	0	0	0	0	0	0	0	Fungi; Ascomycota; Sordariomycetes; Chaetosphaeriales; Chaetosphaeriaceae; Chaetosphaeria; Chaetosphaeria_sp
New.ReferenceOTU7300	0	0	0	0	0	0	0	0	0	0	3	0	0	0	0	0	0	0	0	0	0	0	0	0	0	0	0	0	0	0	Fungi; Ascomycota; Sordariomycetes; Chaetosphaeriales; Chaetosphaeriaceae; Chaetosphaeria; Chaetosphaeria_sp
New.ReferenceOTU7609	0	0	0	0	0	0	0	0	0	0	0	0	0	0	0	0	0	17	0	0	0	0	0	0	0	0	0	0	0	0	Fungi; Ascomycota; Sordariomycetes; Chaetosphaeriales; Chaetosphaeriaceae; Chaetosphaeria; Chaetosphaeria_sp
SH013091.06FU_GQ219819_reps_singleton	0	0	0	1	21	0	0	0	13	11	0	0	12	1	0	3	1	0	1	0	0	0	0	0	0	0	0	0	0	0	Fungi; Ascomycota; Sordariomycetes; Chaetosphaeriales; Chaetosphaeriaceae; Chaetosphaeria; Chaetosphaeria_sp
SH013094.06FU_JQ666431_reps_singleton	0	0	0	0	0	0	0	0	0	1	0	0	0	0	0	0	0	0	0	3	0	0	0	0	0	0	0	0	0	0	Fungi; Ascomycota; Sordariomycetes; Chaetosphaeriales; Chaetosphaeriaceae; Chaetosphaeria; Chaetosphaeria_sp
SH138843.06FU_EU076954_reps	6	3	10	0	0	0	0	0	9	3	0	1	0	0	0	2	1	6	3	2	0	0	0	1	2	0	0	0	0	0	Fungi; Ascomycota; Sordariomycetes; Chaetosphaeriales; Chaetosphaeriaceae; Chaetosphaeria; Chaetosphaeria_sp
SH138849.06FU_AY699663_reps	0	0	0	0	0	0	0	0	1	0	1	0	0	0	1	0	0	0	0	0	0	0	0	0	0	0	0	0	0	0	Fungi; Ascomycota; Sordariomycetes; Chaetosphaeriales; Chaetosphaeriaceae; Chaetosphaeria; Chaetosphaeria_sp
New.ReferenceOTU7039	0	0	0	0	0	0	0	0	2	0	0	0	0	0	0	0	0	0	0	0	0	0	0	0	0	0	0	0	0	0	Fungi; Ascomycota; Sordariomycetes; Chaetosphaeriales; Chaetosphaeriaceae; Chaetosphaeria; Chaetosphaeria_sp_KRCF710
New.ReferenceOTU412	0	0	0	0	0	0	0	0	0	0	0	0	0	0	0	0	0	0	0	0	0	0	0	0	0	0	0	0	2	0	Fungi; Ascomycota; Sordariomycetes; Chaetosphaeriales; Chaetosphaeriaceae; Chaetosphaeria; Chaetosphaeria_sp_olrim767
New.ReferenceOTU2638	0	0	0	0	0	0	0	0	0	0	0	0	0	0	0	9	0	0	0	0	0	0	0	0	0	0	0	0	0	0	Fungi; Ascomycota; Sordariomycetes; Chaetosphaeriales; Chaetosphaeriaceae; Codinaeopsis; Codinaeopsis_gonytrichoides
New.ReferenceOTU2663	0	0	0	0	0	0	0	0	0	0	0	0	0	0	0	0	0	0	0	0	0	1	0	0	0	0	0	0	0	0	Fungi; Ascomycota; Sordariomycetes; Chaetosphaeriales; Chaetosphaeriaceae; Codinaeopsis; Codinaeopsis_gonytrichoides
New.ReferenceOTU681	0	0	0	0	0	0	0	0	0	0	0	0	0	0	0	0	0	0	0	1	0	0	0	0	0	0	0	0	0	0	Fungi; Ascomycota; Sordariomycetes; Chaetosphaeriales; Chaetosphaeriaceae; Codinaeopsis; Codinaeopsis_gonytrichoides
New.ReferenceOTU7138	0	0	1	0	0	0	0	0	0	0	0	0	0	0	0	0	0	0	0	1	0	0	0	0	0	0	2	0	0	0	Fungi; Ascomycota; Sordariomycetes; Chaetosphaeriales; Chaetosphaeriaceae; Codinaeopsis; Codinaeopsis_gonytrichoides
New.ReferenceOTU6783	0	0	0	0	0	0	0	0	0	0	0	0	0	0	0	0	0	0	0	0	0	0	0	0	0	0	0	0	0	3	Fungi; Ascomycota; Sordariomycetes; Chaetosphaeriales; Chaetosphaeriaceae; Kylindria; Kylindria_ellisii
New.ReferenceOTU2637	0	0	0	0	0	0	0	0	0	0	0	0	0	0	0	0	0	0	0	0	0	0	0	0	1	0	0	0	0	0	Fungi; Ascomycota; Sordariomycetes; Chaetosphaeriales; Chaetosphaeriaceae; Menispora; Menispora_tortuosa

New.ReferenceOTU3531	0	0	0	0	0	0	0	0	0	1	0	0	0	0	0	0	0	0	0	0	0	0	0	0	0	0	0	0	0	0	Fungi; Ascomycota; Sordariomycetes; Chaetosphaeriales; Chaetosphaeriaceae; Striatosphaeria; Striatosphaeria_codinaeophora
New.ReferenceOTU1369	0	0	0	0	0	0	0	0	0	0	1	0	0	0	0	0	0	0	0	0	0	1	0	0	0	0	0	0	0	0	Fungi; Ascomycota; Sordariomycetes; Chaetosphaeriales; Chaetosphaeriaceae; Thozetella; Thozetella_sp_ASR_187
SH017929.06FU_GU973741_reps_singleton	0	0	0	0	0	0	0	0	0	0	0	0	1	0	0	0	0	0	0	0	0	0	0	0	0	0	0	0	0	0	Fungi; Ascomycota; Sordariomycetes; Chaetosphaeriales; Chaetosphaeriaceae; Thozetella; Thozetella_sp_ASR_257
New.ReferenceOTU1933	0	0	1	0	0	0	0	0	0	0	0	0	0	1	0	0	0	0	0	0	0	0	0	0	0	0	0	0	0	0	Fungi; Ascomycota; Sordariomycetes; Coniochaetales; Coniochaetaceae; Coniochaeta; Coniochaeta_sp_SS_1760
New.ReferenceOTU6850	0	3	0	0	0	0	0	1	0	0	0	0	0	0	0	0	0	0	0	0	0	0	0	0	0	0	0	0	0	0	Fungi; Ascomycota; Sordariomycetes; Diaporthales; Incertae_sedis; Harknessia; Harknessia_eucalyptorum
New.ReferenceOTU7805	0	0	0	0	0	0	0	0	0	4	0	0	0	0	0	0	0	0	0	0	0	0	0	0	0	0	0	0	0	0	Fungi; Ascomycota; Sordariomycetes; Diaporthales; Incertae_sedis; Prosthecium; Prosthecium_sp
New.ReferenceOTU5881	0	0	0	0	0	0	0	0	1	0	0	0	0	0	0	0	0	0	0	1	0	0	0	0	0	0	0	0	0	0	Fungi; Ascomycota; Sordariomycetes; Diaporthales; Melanconidaceae; Melanconiella; Melanconiella_spodiaea
New.ReferenceOTU4716	0	0	0	0	0	0	0	0	0	0	0	0	0	0	1	0	0	0	0	0	0	0	0	0	1	0	0	0	0	1	Fungi; Ascomycota; Sordariomycetes; Diaporthales; Sydowiellaceae; Hapalocystis; Hapalocystis_berkeleyi
New.ReferenceOTU4959	0	0	0	0	0	0	0	0	0	0	0	0	0	0	0	0	0	0	0	0	0	1	0	0	0	0	0	0	0	0	Fungi; Ascomycota; Sordariomycetes; Diaporthales; Sydowiellaceae; Hapalocystis; Hapalocystis_berkeleyi
New.ReferenceOTU6495	0	0	0	0	0	0	0	0	0	0	0	0	0	0	0	0	0	0	0	0	0	0	0	0	0	0	0	0	0	1	Fungi; Ascomycota; Sordariomycetes; Diaporthales; Sydowiellaceae; Hapalocystis; Hapalocystis_berkeleyi
New.ReferenceOTU7703	0	0	0	0	0	0	0	0	0	1	1	0	0	3	0	0	0	0	0	0	0	0	0	0	0	0	0	0	0	0	Fungi; Ascomycota; Sordariomycetes; Diaporthales; Sydowiellaceae; Hapalocystis; Hapalocystis_berkeleyi
New.ReferenceOTU6958	0	0	0	0	0	0	0	0	0	0	0	0	0	0	0	0	0	0	0	0	0	0	0	0	0	29	0	0	0	0	Fungi; Ascomycota; Sordariomycetes; Hypocreales; Bionectriaceae; Stephanonectria; Stephanonectria_sp_HN_02
New.ReferenceOTU6298	0	0	0	0	0	0	0	0	0	0	0	0	0	0	0	0	0	0	1	0	0	0	0	0	0	0	0	0	0	0	Fungi; Ascomycota; Sordariomycetes; Hypocreales; Bionectriaceae; Stromatonectria; Stromatonectria_caraganae
New.ReferenceOTU642	0	0	0	0	0	0	0	0	1	1	0	0	0	0	0	0	1	0	0	0	0	0	0	0	0	0	0	0	0	0	Fungi; Ascomycota; Sordariomycetes; Hypocreales; Bionectriaceae; Stromatonectria; Stromatonectria_sp
New.ReferenceOTU7186	0	0	0	0	0	0	0	0	0	1	0	0	0	0	0	0	0	0	0	0	0	0	0	0	0	0	0	0	0	0	Fungi; Ascomycota; Sordariomycetes; Hypocreales; Bionectriaceae; Stromatonectria; Stromatonectria_sp
New.ReferenceOTU2961	0	0	0	0	0	0	0	0	0	0	0	0	0	0	0	0	0	0	1	0	0	0	0	0	0	0	0	0	0	0	Fungi; Ascomycota; Sordariomycetes; Hypocreales; Bionectriaceae; unidentified; Bionectriaceae_sp
SH127995.06FU_AF358227_reps	0	0	0	0	0	0	0	0	0	0	0	0	0	0	0	1	0	0	3	0	0	0	0	0	0	0	0	0	0	0	Fungi; Ascomycota; Sordariomycetes; Hypocreales; Bionectriaceae; unidentified; Bionectriaceae_sp
SH141470.06FU_AY254158_reps	0	0	3	0	0	0	0	0	2	2	0	0	0	1	1	0	2	1	1	0	0	1	0	1	0	0	0	0	0	0	Fungi; Ascomycota; Sordariomycetes; Hypocreales; Bionectriaceae; unidentified; Bionectriaceae_sp
New.ReferenceOTU1886	0	0	0	0	0	0	0	0	0	0	0	0	0	0	0	0	0	0	0	0	0	0	7	0	0	0	0	0	0	0	Fungi; Ascomycota; Sordariomycetes; Hypocreales; Bionectriaceae; Verrucostoma; Verrucostoma_freycinetiae
New.ReferenceOTU5287	0	0	0	0	0	0	0	0	0	0	0	0	0	0	0	0	0	0	0	0	0	0	1	0	0	0	0	0	0	0	Fungi; Ascomycota; Sordariomycetes; Hypocreales; Bionectriaceae; Verrucostoma; Verrucostoma_freycinetiae
New.ReferenceOTU5293	0	0	1	0	0	0	0	0	0	0	0	0	0	0	0	0	0	0	0	0	0	0	0	0	0	0	0	0	0	0	Fungi; Ascomycota; Sordariomycetes; Hypocreales; Clavicipitaceae; Drechmeria; Drechmeria_coniospora
New.ReferenceOTU705	0	0	0	0	4	0	0	0	0	0	0	0	0	0	0	0	0	0	0	0	0	0	0	0	0	0	0	0	0	0	Fungi; Ascomycota; Sordariomycetes; Hypocreales; Clavicipitaceae; Drechmeria; Drechmeria_coniospora
New.ReferenceOTU1035	0	0	1	0	0	0	0	0	0	0	0	0	0	0	0	0	0	0	0	0	0	0	0	0	0	0	0	0	0	0	Fungi; Ascomycota; Sordariomycetes; Hypocreales; Clavicipitaceae; unidentified; Clavicipitaceae_sp
New.ReferenceOTU1707	1	0	1	0	0	0	1	2	0	0	0	0	0	0	0	0	3	0	0	0	0	0	0	0	1	0	0	0	0	0	Fungi; Ascomycota; Sordariomycetes; Hypocreales; Clavicipitaceae; unidentified; Clavicipitaceae_sp
New.ReferenceOTU8019	0	0	2	0	0	0	0	0	0	0	0	0	0	0	0	0	0	1	0	0	0	0	0	1	0	0	0	0	2	0	Fungi; Ascomycota; Sordariomycetes; Hypocreales; Clavicipitaceae; unidentified; Clavicipitaceae_sp
SH015256.06FU_FJ765032_reps_singleton	0	0	1	2	0	1	0	7	0	0	0	1	3	1	0	0	5	0	1	0	0	2	0	0	4	0	0	0	0	0	Fungi; Ascomycota; Sordariomycetes; Hypocreales; Clavicipitaceae; unidentified; Clavicipitaceae_sp
SH119064.06FU_HM030586_reps	0	0	0	0	0	0	0	0	0	0	0	0	0	0	0	0	0	0	1	0	0	0	0	1	0	0	0	0	0	0	Fungi; Ascomycota; Sordariomycetes; Hypocreales; Clavicipitaceae; unidentified; Clavicipitaceae_sp
New.ReferenceOTU746	0	0	0	0	0	0	0	0	0	0	0	0	0	0	0	0	0	0	0	0	0	0	0	0	1	0	0	0	0	0	Fungi; Ascomycota; Sordariomycetes; Hypocreales; Clavicipitaceae; unidentified; Clavicipitaceae_sp_PrubF01
New.CleanUp.ReferenceOTU1415	0	0	0	0	0	0	0	0	0	0	0	0	0	0	0	0	0	0	0	0	0	0	0	0	0	3	0	0	1	0	Fungi; Ascomycota; Sordariomycetes; Hypocreales; Cordycipitaceae; unidentified; Cordycipitaceae_sp
New.CleanUp.ReferenceOTU361	0	1	0	0	0	0	0	0	0	0	0	0	0	0	0	0	0	0	0	0	0	0	0	0	0	1	0	0	0	0	Fungi; Ascomycota; Sordariomycetes; Hypocreales; Cordycipitaceae; unidentified; Cordycipitaceae_sp
New.ReferenceOTU1601	0	0	0	0	0	0	0	0	0	0	0	0	0	0	0	0	0	0	0	0	0	0	0	0	0	0	0	0	2	0	Fungi; Ascomycota; Sordariomycetes; Hypocreales; Cordycipitaceae; unidentified; Cordycipitaceae_sp
New.ReferenceOTU2374	0	6	0	0	0	0	0	0	0	0	0	0	0	0	0	0	0	0	0	0	0	0	0	0	0	0	0	0	0	0	Fungi; Ascomycota; Sordariomycetes; Hypocreales; Cordycipitaceae; unidentified; Cordycipitaceae_sp
New.ReferenceOTU3758	0	109	0	0	0	0	0	0	0	0	0	0	0	0	0	0	0	0	0	0	0	0	0	10	1	184	0	0	127	0	Fungi; Ascomycota; Sordariomycetes; Hypocreales; Cordycipitaceae; unidentified; Cordycipitaceae_sp
New.ReferenceOTU5142	0	3	0	0	0	0	0	0	0	1	0	0	0	0	0	0	0	0	0	0	0	0	0	0	4	140	0	0	20	0	Fungi; Ascomycota; Sordariomycetes; Hypocreales; Cordycipitaceae; unidentified; Cordycipitaceae_sp
New.ReferenceOTU527	0	0	0	0	0	0	0	0	0	0	0	0	0	0	0	0	0	0	0	0	0	0	0	0	0	0	0	0	0	1	Fungi; Ascomycota; Sordariomycetes; Hypocreales; Cordycipitaceae; unidentified; Cordycipitaceae_sp
New.ReferenceOTU6560	0	0	0	0	0	0	1	0	0	0	0	0	0	0	0	0	0	0	0	0	0	0	0	0	1	0	0	0	0	0	Fungi; Ascomycota; Sordariomycetes; Hypocreales; Cordycipitaceae; unidentified; Cordycipitaceae_sp
New.ReferenceOTU6756	0	0	0	0	0	0	0	0	2	0	2	0	0	0	0	0	0	0	6	0	0	0	0	0	0	0	0	0	0	0	Fungi; Ascomycota; Sordariomycetes; Hypocreales; Cordycipitaceae; unidentified; Cordycipitaceae_sp
New.ReferenceOTU7034	0	3	0	0	0	0	0	0	0	0	0	0	0	0	0	0	0	0	0	0	0	0	0	0	0	0	0	0	2	0	Fungi; Ascomycota; Sordariomycetes; Hypocreales; Cordycipitaceae; unidentified; Cordycipitaceae_sp
New.ReferenceOTU7144	0	0	0	0	0	0	0	0	0	0	2	0	0	0	0	0	0	0	0	0	0	0	0	0	0	0	0	0	0	0	Fungi; Ascomycota; Sordariomycetes; Hypocreales; Cordycipitaceae; unidentified; Cordycipitaceae_sp
New.ReferenceOTU793	0	10	0	0	0	0	0	0	0	0	0	0	0	0	0	0	0	0	0	0	0	0	0	0	0	92	0	0	11	0	Fungi; Ascomycota; Sordariomycetes; Hypocreales; Cordycipitaceae; unidentified; Cordycipitaceae_sp
SH110560.06FU_HQ021813_reps	0	0	0	1	0	0	0	0	1	0	4	0	0	3	0	0	0	0	6	0	0	0	0	0	0	2	0	0	0	0	Fungi; Ascomycota; Sordariomycetes; Hypocreales; Cordycipitaceae; unidentified; Cordycipitaceae_sp
New.ReferenceOTU4560	0	0	0	0	0	0	0	0	0	0	0	0	1	0	0	0	0	0	0	0	0	0	0	0	0	0	0	0	0	0	Fungi; Ascomycota; Sordariomycetes; Hypocreales; Hypocreaceae; Acrostalagmus; Acrostalagmus_luteoalbus
New.ReferenceOTU641	0	0	0	0	3	0	0	0	0	0	0	0	0	0	0	0	0	0	0	0	0	0	0	0	0	0	0	0	0	0	Fungi; Ascomycota; Sordariomycetes; Hypocreales; Hypocreaceae; Acrostalagmus; Acrostalagmus_luteoalbus
SH121357.06FU_GU180632_reps	0	0	0	0	0	0	0	0	0	0	0	0	0	0	0	0	0	0	2	0	0	0	1	0	3	0	0	0	0	0	Fungi; Ascomycota; Sordariomycetes; Hypocreales; Hypocreaceae; Acrostalagmus; Acrostalagmus_sp_MR_2010
SH124974.06FU_KC171340_refs	1	0	0	0	0	0	0	0	0	0	0	0	0	1	1	0	0	0	0	0	0	0	0	0	0	0	0	0	0	0	Fungi; Ascomycota; Sordariomycetes; Hypocreales; Hypocreaceae; Trichoderma; Trichoderma_asperellum
New.ReferenceOTU344	0	0	0	0	0	1	0	3	0	0	0	0	0	0	0	0	0	0	0	0	0	0	0	0	0	0	1	0	0	0	Fungi; Ascomycota; Sordariomycetes; Hypocreales; Hypocreaceae; Trichoderma; Trichoderma_ceramicum
New.ReferenceOTU1266	0	0	0	0	0	0	0	0	0	0	2	0	0	0	0	0	0	0	0	0	0	0	0	0	0	0	0	0	0	0	Fungi; Ascomycota; Sordariomycetes; Hypocreales; Hypocreaceae; Trichoderma; Trichoderma_erinaceum
New.ReferenceOTU7578	6	0	1	1	0	0	0	1	0	0	0	1	0	0	0	1	0	0	0	0	3	1	1	0	1	0	2	0	0	0	Fungi; Ascomycota; Sordariomycetes; Hypocreales; Hypocreaceae; Trichoderma; Trichoderma_erinaceum
SH095980.06FU_DQ993578_reps	0	0	0	0	0	0	0	0	0	0	0	1	1	0	0	0	0	0	0	0	0	1	0	0	0	0	0	0	0	0	Fungi; Ascomycota; Sordariomycetes; Hypocreales; Hypocreaceae; Trichoderma; Trichoderma_erinaceum
New.ReferenceOTU3894	0	0	0	1	0	0	0	0	0	0	0	1	0	0	0	0	0	0	2	0	0	0	0	0	0	0	0	0	0	0	Fungi; Ascomycota; Sordariomycetes; Hypocreales; Hypocreaceae; Trichoderma; Trichoderma_harzianum
New.ReferenceOTU480	0	1	0	0	0	0	0	0	0	0	0	0	0	0	0	0	0	0	0	0	0	0	0	0	0	0	0	0	0	0	Fungi; Ascomycota; Sordariomycetes; Hypocreales; Hypocreaceae; Trichoderma; Trichoderma_harzianum
New.ReferenceOTU1090	0	0	0	0	0	0	0	0	0	0	0	1	0	1	0	0	0	1	1	0	0	0	1	0	0	0	0	0	3	0	Fungi; Ascomycota; Sordariomycetes; Hypocreales; Hypocreaceae; Trichoderma; Trichoderma_helicum
New.ReferenceOTU2373	0	0	0	0	0	0	0	0	3	1	0	0	0	1	0	1	0	1	0	0	1	0	0	0	0	1	0	0	0	0	Fungi; Ascomycota; Sordariomycetes; Hypocreales; Hypocreaceae; Trichoderma; Trichoderma_helicum
SH099939.06FU_EU680561_reps	0	0	0	0	0	0	0	0	0	0	0	0	0	0	0	0	0	0	0	0	1	0	0	0	0	0	0	0	0	0	Fungi; Ascomycota; Sordariomycetes; Hypocreales; Hypocreaceae; Trichoderma; Trichoderma_helicum
SH099952.06FU_KC171339_reps	0	0	0	0	0	0	0	0	0	0	0	0	0	0	0	0	0	0	0	0	1	0	0	0	0	0	0	0	0	0	Fungi; Ascomycota; Sordariomycetes; Hypocreales; Hypocreaceae; Trichoderma; Trichoderma_helicum
New.ReferenceOTU2277	0	0	0	0	0	0	0	0	1	0	1	0	0	0	0	0	0	0	0	0	0	0	0	0	0	0	0	0	0	0	Fungi; Ascomycota; Sordariomycetes; Hypocreales; Hypocreaceae; Trichoderma; Trichoderma_rossicum
New.ReferenceOTU4442	0	0	0	0	0	0	0	0	0	0	2	0	0	0	0	0	0	0	0	0	0	0	0	0	0	0	0	0	0	0	Fungi; Ascomycota; Sordariomycetes; Hypocreales; Hypocreaceae; Trichoderma; Trichoderma_rossicum
New.ReferenceOTU5405	0	0	0	0	0	0	0	0	0	0	1	0	0	0	0	0	0	0	0	0	0	0	0	0	0	0	0	0	0	0	Fungi; Ascomycota; Sordariomycetes; Hypocreales; Hypocreaceae; Trichoderma; Trichoderma_sp
New.ReferenceOTU2376	0	0	0	0	0	0	0	0	0	0	0	0	0	0	0	0	0	0	1	0	0	0	0	0	0	0	0	0	0	0	Fungi; Ascomycota; Sordariomycetes; Hypocreales; Hypocreaceae; Trichoderma; Trichoderma_sp_1212_03
New.ReferenceOTU3655	0	0	0	0	0	0	0	0	0	0	0	0	0	0	0	0	0	0	0	0	0	0	1	0	0	0	0	0	0	0	Fungi; Ascomycota; Sordariomycetes; Hypocreales; Hypocreaceae; Trichoderma; Trichoderma_sp_1212_03
New.ReferenceOTU7299	0	0	3	0	0	0	0	0	0	0	0	0	0	0	0	0	0	0	0	0	0	0	0	0	0	0	0	0	0	0	Fungi; Ascomycota; Sordariomycetes; Hypocreales; Hypocreaceae; Trichoderma; Trichoderma_sp_1212_03
New.ReferenceOTU3560	0	0	1	0	2	1	0	0	0	0	0	0	0	0	0	0	0	0	0	0	0	0	0	0	0	1	0	0	0	0	Fungi; Ascomycota; Sordariomycetes; Hypocreales; Hypocreaceae; Trichoderma; Trichoderma_sp_CHX_2009a
New.ReferenceOTU1570	0	2	0	0	0	0	0	0	0	0	0	0	0	0	0	0	0	0	0	0	0	0	0	0	0	0	0	0	0	0	Fungi; Ascomycota; Sordariomycetes; Hypocreales; Hypocreaceae; Trichoderma; Trichoderma_sp_FH6_16
New.ReferenceOTU6490	0	0	0	0	0	0	0	0	0	0	0	0	0	0	0	0	0	0	1	0	0	0	0	0	0	0	0	0	0	0	Fungi; Ascomycota; Sordariomycetes; Hypocreales; Hypocreaceae; Trichoderma; Trichoderma_sp_FH6_16
New.CleanUp.ReferenceOTU1158	2	0	2	0	0	0	0	0	3	0	3	1	1	0	0	0	1	0	0	2	0	0	0	2	0	0	1	2	1	1	Fungi; Ascomycota; Sordariomycetes; Hypocreales; Hypocreaceae; Trichoderma; Trichoderma_spirale
New.ReferenceOTU1559	0	0	1	2	0	0	4	0	7	2	1	2	12	0	1	0	0	0	5	0	0	0	0	0	0	0	0	0	0	0	Fungi; Ascomycota; Sordariomycetes; Hypocreales; Hypocreaceae; Trichoderma; Trichoderma_spirale
New.ReferenceOTU28	7	2	38	4	4	1	7	11	34	14	8	17	30	7	4	4	8	1	7	4	4	7	2	5	4	0	4	7	3	4	Fungi; Ascomycota; Sordariomycetes; Hypocreales; Hypocreaceae; Trichoderma; Trichoderma_spirale
New.ReferenceOTU3225	2	0	1	1	3	0	2	2	4	1	1	2	10	0	0	2	0	0	1	0	0	0	0	1	1	0	0	0	0	0	Fungi; Ascomycota; Sordariomycetes; Hypocreales; Hypocreaceae; Trichoderma; Trichoderma_spirale
New.ReferenceOTU5548	0	0	2	0	0	0	1	0	2	0	0	0	1	0	0	0	0	0	1	0	0	0	0	0	0	0	1	0	0	0	Fungi; Ascomycota; Sordariomycetes; Hypocreales; Hypocreaceae; Trichoderma; Trichoderma_spirale
New.ReferenceOTU6917	0	0	0	0	0	0	0	0	0	0	0	0	0	0	0	0	0	1	0	0	0	0	0	0	0	0	0	0	0	0	Fungi; Ascomycota; Sordariomycetes; Hypocreales; Hypocreaceae; Trichoderma; Trichoderma_spirale
SH099954.06FU_JF303860_reps	0	0	0	0	0	0	0	1	0	0	0	0	0	1	0	0	0	0	0	0	0	0	0	0	0	0	0	0	0	0	Fungi; Ascomycota; Sordariomycetes; Hypocreales; Hypocreaceae; Trichoderma; Trichoderma_spirale
SH144342.06FU_FJ860770_refs	1	0	11	0	0	1	2	6	2	7	4	7	12	11	2	2	4	3	5	6	0	0	1	3	3	0	0	2	0	1	Fungi; Ascomycota; Sordariomycetes; Hypocreales; Hypocreaceae; Trichoderma; Trichoderma_spirale
SH144358.06FU_AB666514_reps	7	1	7	5	3	2	6	5	27	7	5	8	10	2	2	1	6	0	4	3	0	2	0	3	3	1	4	5	0	2	Fungi; Ascomycota; Sordariomycetes; Hypocreales; Hypocreaceae; Trichoderma; Trichoderma_spirale
SH146048.06FU_EU280068_refs	1	0	7	1	0	0	0	8	5	4	8	8	2	2	1	0	0	0	2	1	0	0	1	1	1	0	0	0	0	1	Fungi; Ascomycota; Sordariomycetes; Hypocreales; Hypocreaceae; Trichoderma; Trichoderma_spirale
SH146058.06FU_EF601619_reps	4	0	11	1	4	0	1	5	5	10	9	4	12	21	1	0	9	12	3	9	3	7	1	4	1	1	0	1	2	0	Fungi; Ascomycota; Sordariomycetes; Hypocreales; Hypocreaceae; Trichoderma; Trichoderma_spirale
New.ReferenceOTU1749	0	0	0	0	2	0	0	0	1	2	0	0	1	4	0	1	0	0	2	1	0	0	0	0	0	3	0	1	0	0	Fungi; Ascomycota; Sordariomycetes; Hypocreales; Hypocreaceae; Trichoderma; Trichoderma_tomentosum
New.ReferenceOTU6599	2	6	0	2	0	0	0	1	0	0	3	0	0	0	0	0	0	1	3	0	0	0	0	0	0	1	0	0	0	0	Fungi; Ascomycota; Sordariomycetes; Hypocreales; Hypocreaceae; Trichoderma; Trichoderma_tomentosum
SH111676.06FU_KC576652_reps	0	1	1	0	0	0	0	0	0	2	2	0	0	0	0	0	0	0	0	0	0	0	0	0	0	0	0	0	0	0	Fungi; Ascomycota; Sordariomycetes; Hypocreales; Hypocreaceae; Trichoderma; Trichoderma_tomentosum
SH111677.06FU_FJ412032_reps	1	4	1	5	1	0	0	0	1	2	2	1	0	0	0	0	0	2	0	1	0	0	0	4	0	2	1	0	2	0	Fungi; Ascomycota; Sordariomycetes; Hypocreales; Hypocreaceae; Trichoderma; Trichoderma_tomentosum
SH140770.06FU_AY605715_reps	0	0	0	5	1	0	0	0	0	0	0	0	0	0	0	0	0	0	1	0	0	0	0	1	0	0	0	0	0	0	Fungi; Ascomycota; Sordariomycetes; Hypocreales; Hypocreaceae; Trichoderma; Trichoderma_tomentosum
New.CleanUp.ReferenceOTU1340	0	0	0	0	0	0	0	0	0	0	0	0	0	0	0	0	0	0	0	0	0	0	0	0	0	0	1	0	1	0	Fungi; Ascomycota; Sordariomycetes; Hypocreales; Hypocreaceae; unidentified; Hypocreaceae_sp
New.CleanUp.ReferenceOTU1974	0	0	0	0	0	0	0	0	0	0	0	0	0	0	0	0	1	0	0	0	0	0	0	0	0	0	0	0	0	0	Fungi; Ascomycota; Sordariomycetes; Hypocreales; Hypocreaceae; unidentified; Hypocreaceae_sp
New.CleanUp.ReferenceOTU2057	0	0	0	0	0	0	0	0	0	0	0	1	0	0	0	0	0	0	0	0	0	0	0	0	0	0	0	0	0	0	Fungi; Ascomycota; Sordariomycetes; Hypocreales; Hypocreaceae; unidentified; Hypocreaceae_sp
New.ReferenceOTU1291	0	0	2	0	0	0	1	0	0	0	0	0	1	0	0	0	0	2	6	0	0	0	0	0	0	0	0	0	0	0	Fungi; Ascomycota; Sordariomycetes; Hypocreales; Hypocreaceae; unidentified; Hypocreaceae_sp
New.ReferenceOTU1386	0	1	1	1	0	0	0	3	5	2	4	5	1	0	0	1	0	0	4	2	0	0	0	0	1	0	0	0	0	0	Fungi; Ascomycota; Sordariomycetes; Hypocreales; Hypocreaceae; unidentified; Hypocreaceae_sp
New.ReferenceOTU1414	0	1	0	0	2	0	0	0	1	2	2	6	1	0	1	1	1	0	2	0	0	0	0	1	0	1	0	0	0	0	Fungi; Ascomycota; Sordariomycetes; Hypocreales; Hypocreaceae; unidentified; Hypocreaceae_sp
New.ReferenceOTU1430	0	0	0	0	0	0	0	0	2	0	0	0	0	0	0	0	0	0	0	0	0	0	0	0	0	0	0	0	0	0	Fungi; Ascomycota; Sordariomycetes; Hypocreales; Hypocreaceae; unidentified; Hypocreaceae_sp
New.ReferenceOTU170	1	0	3	1	1	2	1	1	7	9	0	0	11	1	2	4	1	0	5	0	0	0	1	6	3	0	3	0	0	2	Fungi; Ascomycota; Sordariomycetes; Hypocreales; Hypocreaceae; unidentified; Hypocreaceae_sp
New.ReferenceOTU1750	0	0	0	0	0	0	0	0	0	0	0	0	1	0	0	0	0	0	0	0	0	0	0	0	0	0	0	0	0	0	Fungi; Ascomycota; Sordariomycetes; Hypocreales; Hypocreaceae; unidentified; Hypocreaceae_sp
New.ReferenceOTU2050	0	0	0	0	0	0	0	0	0	0	0	0	0	0	0	0	1	0	1	0	0	0	1	0	0	0	0	0	0	0	Fungi; Ascomycota; Sordariomycetes; Hypocreales; Hypocreaceae; unidentified; Hypocreaceae_sp
New.ReferenceOTU2076	0	0	0	1	0	0	0	0	0	0	0	0	0	0	0	0	0	0	0	0	0	0	0	0	0	0	0	0	0	0	Fungi; Ascomycota; Sordariomycetes; Hypocreales; Hypocreaceae; unidentified; Hypocreaceae_sp
New.ReferenceOTU2689	0	0	0	0	0	0	0	0	0	0	0	0	0	0	0	0	0	0	0	0	0	0	0	0	1	0	0	0	0	0	Fungi; Ascomycota; Sordariomycetes; Hypocreales; Hypocreaceae; unidentified; Hypocreaceae_sp
New.ReferenceOTU2742	0	4	0	0	0	0	0	0	0	0	0	0	0	0	0	1	0	1	2	0	0	0	0	1	0	0	1	0	0	0	Fungi; Ascomycota; Sordariomycetes; Hypocreales; Hypocreaceae; unidentified; Hypocreaceae_sp
New.ReferenceOTU2755	0	0	0	0	0	0	0	0	0	0	0	0	0	0	0	1	4	0	0	1	0	0	0	0	0	0	0	0	0	0	Fungi; Ascomycota; Sordariomycetes; Hypocreales; Hypocreaceae; unidentified; Hypocreaceae_sp
New.ReferenceOTU2793	0	0	0	0	1	0	0	0	0	0	0	0	0	0	0	0	1	0	0	0	0	0	0	0	0	0	0	0	0	0	Fungi; Ascomycota; Sordariomycetes; Hypocreales; Hypocreaceae; unidentified; Hypocreaceae_sp
New.ReferenceOTU3054	0	0	0	0	0	0	0	0	0	0	0	0	0	0	0	1	0	0	0	0	0	0	0	0	0	0	0	0	0	1	Fungi; Ascomycota; Sordariomycetes; Hypocreales; Hypocreaceae; unidentified; Hypocreaceae_sp
New.ReferenceOTU3322	0	0	0	1	0	0	0	0	0	0	0	0	0	0	0	0	0	0	0	0	0	0	0	0	0	0	0	0	0	0	Fungi; Ascomycota; Sordariomycetes; Hypocreales; Hypocreaceae; unidentified; Hypocreaceae_sp
New.ReferenceOTU4540	0	0	0	0	1	0	0	0	1	0	0	0	1	5	0	2	1	0	0	0	0	0	0	1	0	0	0	0	0	0	Fungi; Ascomycota; Sordariomycetes; Hypocreales; Hypocreaceae; unidentified; Hypocreaceae_sp
New.ReferenceOTU4877	0	0	0	0	0	0	0	0	0	0	0	0	0	0	0	0	0	0	0	0	0	0	0	0	0	0	0	0	0	2	Fungi; Ascomycota; Sordariomycetes; Hypocreales; Hypocreaceae; unidentified; Hypocreaceae_sp
New.ReferenceOTU5902	2	6	1	0	0	0	1	0	0	0	1	0	1	1	0	1	0	1	0	0	0	3	0	0	1	0	2	0	0	0	Fungi; Ascomycota; Sordariomycetes; Hypocreales; Hypocreaceae; unidentified; Hypocreaceae_sp

New.ReferenceOTU5928	0	0	0	0	0	1	0	0	0	0	0	0	0	0	0	0	0	0	0	0	0	0	0	0	0	0	0	0	0	0	Fungi; Ascomycota; Sordariomycetes; Hypocreales; Hypocreaceae; unidentified; Hypocreaceae_sp
New.ReferenceOTU6240	0	0	1	0	0	0	0	0	0	0	0	0	0	0	0	0	0	0	0	0	0	0	0	0	0	0	0	0	0	0	Fungi; Ascomycota; Sordariomycetes; Hypocreales; Hypocreaceae; unidentified; Hypocreaceae_sp
New.ReferenceOTU6719	1	0	0	0	0	0	3	0	0	0	1	8	2	10	1	0	3	0	0	1	0	1	0	0	0	0	0	0	0	0	Fungi; Ascomycota; Sordariomycetes; Hypocreales; Hypocreaceae; unidentified; Hypocreaceae_sp
SH037764.06FU_AM889224_reps_singleton	0	0	0	0	0	0	0	0	0	0	0	0	0	0	0	0	0	0	0	0	0	0	0	0	1	0	0	0	0	0	Fungi; Ascomycota; Sordariomycetes; Hypocreales; Hypocreaceae; unidentified; Hypocreaceae_sp
SH092794.06FU_Z69588_refs	1	0	0	0	0	0	0	0	0	0	0	0	0	0	0	0	0	0	0	0	0	0	0	0	0	0	0	0	0	0	Fungi; Ascomycota; Sordariomycetes; Hypocreales; Hypocreaceae; unidentified; Hypocreaceae_sp
SH092796.06FU_EF552702_reps	0	0	0	0	0	0	0	0	0	0	1	3	0	0	0	0	0	0	0	0	0	0	0	0	0	0	0	0	0	0	Fungi; Ascomycota; Sordariomycetes; Hypocreales; Hypocreaceae; unidentified; Hypocreaceae_sp
SH099935.06FU_JN798592_reps	0	1	3	3	1	0	1	4	1	2	16	13	1	4	0	1	1	2	8	3	0	0	2	3	7	0	4	0	9	1	Fungi; Ascomycota; Sordariomycetes; Hypocreales; Hypocreaceae; unidentified; Hypocreaceae_sp
SH099941.06FU_DQ345853_reps	0	0	1	0	2	0	0	0	0	0	1	2	0	0	0	0	0	0	0	0	0	0	0	0	0	0	2	0	0	0	Fungi; Ascomycota; Sordariomycetes; Hypocreales; Hypocreaceae; unidentified; Hypocreaceae_sp
SH099942.06FU_JQ797389_reps	0	5	1	1	0	0	0	0	0	1	2	1	0	1	0	1	0	0	3	0	0	0	0	0	0	2	0	0	2	0	Fungi; Ascomycota; Sordariomycetes; Hypocreales; Hypocreaceae; unidentified; Hypocreaceae_sp
SH099955.06FU_FR686989_reps	2	3	0	0	3	0	1	0	2	1	0	4	0	0	0	0	0	0	1	0	0	0	0	0	0	4	0	0	3	1	Fungi; Ascomycota; Sordariomycetes; Hypocreales; Hypocreaceae; unidentified; Hypocreaceae_sp
SH111663.06FU_KC582837_reps	0	0	0	0	0	0	1	1	0	3	1	0	0	0	0	0	1	0	1	0	0	0	0	0	1	0	0	0	0	0	Fungi; Ascomycota; Sordariomycetes; Hypocreales; Hypocreaceae; unidentified; Hypocreaceae_sp
SH111664.06FU_FJ610287_reps	0	0	0	0	0	0	0	0	0	0	0	3	0	1	0	0	2	0	4	0	0	0	0	0	0	0	0	0	0	0	Fungi; Ascomycota; Sordariomycetes; Hypocreales; Hypocreaceae; unidentified; Hypocreaceae_sp
SH111666.06FU_FN396565_reps	0	0	0	3	0	0	0	0	1	0	1	0	1	0	0	0	0	1	1	1	0	0	0	0	0	0	1	0	0	0	Fungi; Ascomycota; Sordariomycetes; Hypocreales; Hypocreaceae; unidentified; Hypocreaceae_sp
SH111667.06FU_HM142358_refs	0	0	0	0	0	0	0	0	1	0	0	0	0	0	0	0	0	0	0	0	0	0	0	1	0	0	1	0	0	0	Fungi; Ascomycota; Sordariomycetes; Hypocreales; Hypocreaceae; unidentified; Hypocreaceae_sp
SH111672.06FU_AF057613_reps	0	0	0	0	0	0	0	0	1	0	0	0	0	0	1	0	1	0	0	0	0	0	0	2	2	0	3	0	0	0	Fungi; Ascomycota; Sordariomycetes; Hypocreales; Hypocreaceae; unidentified; Hypocreaceae_sp
SH111690.06FU_EU917111_reps	0	0	0	0	0	0	0	0	0	0	0	0	0	0	0	0	0	0	2	0	0	0	0	0	0	0	0	0	0	0	Fungi; Ascomycota; Sordariomycetes; Hypocreales; Hypocreaceae; unidentified; Hypocreaceae_sp
SH111699.06FU_AB570244_reps	0	1	0	0	0	0	0	0	0	0	0	0	0	0	0	0	0	0	1	0	0	0	0	0	0	0	0	0	0	0	Fungi; Ascomycota; Sordariomycetes; Hypocreales; Hypocreaceae; unidentified; Hypocreaceae_sp
SH111704.06FU_EF601674_reps	0	0	0	0	0	0	0	0	0	0	0	0	0	0	0	0	0	0	0	0	0	0	1	0	0	0	1	0	0	0	Fungi; Ascomycota; Sordariomycetes; Hypocreales; Hypocreaceae; unidentified; Hypocreaceae_sp
SH111705.06FU_FR686991_reps	0	0	0	0	0	0	0	0	1	0	0	0	0	0	0	0	1	0	0	1	0	0	0	0	0	0	0	0	0	1	Fungi; Ascomycota; Sordariomycetes; Hypocreales; Hypocreaceae; unidentified; Hypocreaceae_sp
SH124979.06FU_AY380910_reps	0	0	1	0	0	0	0	0	0	0	1	0	0	0	1	0	0	0	0	0	0	0	0	2	0	0	0	0	0	0	Fungi; Ascomycota; Sordariomycetes; Hypocreales; Hypocreaceae; unidentified; Hypocreaceae_sp
SH140764.06FU_AF057584_reps	0	1	0	0	2	0	0	0	1	1	0	0	0	0	0	0	0	0	1	0	0	0	0	0	0	0	1	0	0	0	Fungi; Ascomycota; Sordariomycetes; Hypocreales; Hypocreaceae; unidentified; Hypocreaceae_sp
SH140765.06FU_AY328037_reps	0	0	0	0	0	0	0	0	1	0	0	0	0	0	0	0	0	0	0	0	0	0	0	0	0	0	0	0	0	0	Fungi; Ascomycota; Sordariomycetes; Hypocreales; Hypocreaceae; unidentified; Hypocreaceae_sp
SH144340.06FU_JQ040358_reps	0	0	0	0	0	0	0	0	1	0	0	0	0	0	0	0	0	0	1	0	0	0	0	0	0	0	1	0	0	0	Fungi; Ascomycota; Sordariomycetes; Hypocreales; Hypocreaceae; unidentified; Hypocreaceae_sp
SH144349.06FU_FR686967_reps	0	1	0	0	1	0	0	0	1	0	0	0	0	0	0	0	0	0	0	0	0	0	0	0	1	0	1	0	0	0	Fungi; Ascomycota; Sordariomycetes; Hypocreales; Hypocreaceae; unidentified; Hypocreaceae_sp
SH144350.06FU_GQ131394_reps	1	0	0	0	0	0	0	0	0	3	0	0	0	1	0	0	0	0	0	0	0	0	2	0	0	0	0	0	0	0	Fungi; Ascomycota; Sordariomycetes; Hypocreales; Hypocreaceae; unidentified; Hypocreaceae_sp
SH146046.06FU_KF144627_reps	0	0	0	0	0	0	0	0	0	0	1	0	0	0	0	0	0	0	0	2	0	0	0	3	3	0	1	0	0	0	Fungi; Ascomycota; Sordariomycetes; Hypocreales; Hypocreaceae; unidentified; Hypocreaceae_sp
SH146056.06FU_KC576661_reps	0	3	0	0	0	0	0	0	0	0	0	0	0	0	0	0	0	1	0	0	0	0	0	0	0	0	0	0	0	0	Fungi; Ascomycota; Sordariomycetes; Hypocreales; Hypocreaceae; unidentified; Hypocreaceae_sp
SH146063.06FU_GU060027_reps	0	0	0	0	0	0	0	0	0	0	0	0	0	0	0	0	0	0	1	0	0	0	0	0	0	0	0	0	0	0	Fungi; Ascomycota; Sordariomycetes; Hypocreales; Hypocreaceae; unidentified; Hypocreaceae_sp
SH146067.06FU_GU059944_reps	0	0	0	0	0	0	0	0	0	0	0	0	2	1	0	0	1	0	0	0	0	0	0	0	1	0	0	0	0	0	Fungi; Ascomycota; Sordariomycetes; Hypocreales; Hypocreaceae; unidentified; Hypocreaceae_sp
New.ReferenceOTU8007	1	0	0	0	0	1	0	0	0	0	0	0	0	0	2	0	0	0	0	0	0	0	0	0	12	0	0	0	0	0	Fungi; Ascomycota; Sordariomycetes; Hypocreales; Hypocreaceae; unidentified; uncultured_Trichoderma
SH023852.06FU_HQ857112_reps_singleton	0	0	0	0	0	0	0	0	0	0	0	0	0	0	0	0	0	0	1	0	0	0	0	0	0	0	0	0	0	0	Fungi; Ascomycota; Sordariomycetes; Hypocreales; Hypocreaceae; unidentified; uncultured_Trichoderma
SH023854.06FU_EU828352_reps_singleton	0	0	0	0	0	0	0	0	0	0	0	0	0	0	0	0	0	0	0	0	0	0	0	0	1	0	0	0	0	0	Fungi; Ascomycota; Sordariomycetes; Hypocreales; Hypocreaceae; unidentified; uncultured_Trichoderma
SH124973.06FU_EU280121_refs	0	0	0	0	0	0	0	0	0	0	0	0	0	0	0	0	0	0	0	0	0	1	0	0	0	0	0	0	0	1	Fungi; Ascomycota; Sordariomycetes; Hypocreales; Hypocreaceae; unidentified; uncultured_Trichoderma
SH124987.06FU_AB297720_reps	0	0	0	0	0	1	0	0	0	0	0	0	0	0	0	0	0	0	0	0	0	0	1	0	1	0	0	0	0	0	Fungi; Ascomycota; Sordariomycetes; Hypocreales; Hypocreaceae; unidentified; uncultured_Trichoderma
SH124997.06FU_KC576718_reps	0	0	0	0	0	0	0	0	0	0	0	0	0	0	0	0	0	0	0	0	0	0	0	0	0	0	0	1	0	0	Fungi; Ascomycota; Sordariomycetes; Hypocreales; Hypocreaceae; unidentified; uncultured_Trichoderma
SH145776.06FU_AY566992_refs	0	0	0	0	0	0	0	0	0	0	0	0	0	0	0	0	0	0	0	0	0	3	0	0	0	0	0	0	0	0	Fungi; Ascomycota; Sordariomycetes; Hypocreales; Incertae_sedis; Acremonium; Acremonium_alternatum
SH121344.06FU_KC506359_reps	0	0	0	1	0	0	1	0	0	0	0	0	0	4	0	0	0	2	0	2	0	2	0	0	9	0	3	0	0	0	Fungi; Ascomycota; Sordariomycetes; Hypocreales; Incertae_sedis; Acremonium; Acremonium_antarcticum
SH121367.06FU_DQ825970_reps	0	0	2	1	0	0	0	0	0	0	0	0	0	0	0	0	0	0	0	0	0	0	0	0	1	0	0	0	0	0	Fungi; Ascomycota; Sordariomycetes; Hypocreales; Incertae_sedis; Acremonium; Acremonium_antarcticum
New.ReferenceOTU3605	0	0	0	0	0	0	0	0	0	0	0	0	0	0	0	0	0	0	0	0	0	0	0	0	1	0	0	0	0	0	Fungi; Ascomycota; Sordariomycetes; Hypocreales; Incertae_sedis; Acremonium; Acremonium_charticola
New.ReferenceOTU3850	0	0	0	0	0	0	0	0	0	0	0	0	0	0	0	0	2	0	0	0	0	0	0	0	0	0	0	0	0	1	Fungi; Ascomycota; Sordariomycetes; Hypocreales; Incertae_sedis; Acremonium; Acremonium_charticola
New.ReferenceOTU1267	0	0	0	0	0	0	0	0	0	0	0	0	0	0	0	0	0	0	0	0	0	0	0	0	0	0	0	2	0	0	Fungi; Ascomycota; Sordariomycetes; Hypocreales; Incertae_sedis; Acremonium; Acremonium_chrysogenum
New.ReferenceOTU2854	0	0	0	0	0	0	0	0	0	0	1	0	0	0	0	0	0	0	0	0	0	0	0	0	0	0	0	0	0	0	Fungi; Ascomycota; Sordariomycetes; Hypocreales; Incertae_sedis; Acremonium; Acremonium_furcatum
New.ReferenceOTU3448	0	0	0	0	0	0	0	0	0	0	0	0	0	1	0	0	0	0	0	0	0	0	0	0	0	0	0	0	0	0	Fungi; Ascomycota; Sordariomycetes; Hypocreales; Incertae_sedis; Acremonium; Acremonium_furcatum
New.ReferenceOTU5128	0	0	0	0	0	0	0	0	0	0	0	0	0	0	0	0	0	0	0	1	0	0	0	0	0	0	0	0	0	0	Fungi; Ascomycota; Sordariomycetes; Hypocreales; Incertae_sedis; Acremonium; Acremonium_furcatum
New.ReferenceOTU7147	0	0	0	0	0	0	0	0	0	1	0	0	0	0	0	0	0	0	0	0	0	0	0	0	0	0	0	0	0	0	Fungi; Ascomycota; Sordariomycetes; Hypocreales; Incertae_sedis; Acremonium; Acremonium_longisporum
SH121340.06FU_JQ647430_refs	0	0	0	0	0	0	0	0	1	0	0	0	0	0	0	0	0	0	0	0	0	0	0	0	0	0	0	0	0	0	Fungi; Ascomycota; Sordariomycetes; Hypocreales; Incertae_sedis; Acremonium; Acremonium_nepalense
New.ReferenceOTU3033	0	0	0	0	0	0	0	0	0	0	0	0	0	0	0	0	0	0	0	0	0	0	2	0	0	0	0	0	0	0	Fungi; Ascomycota; Sordariomycetes; Hypocreales; Incertae_sedis; Acremonium; Acremonium_polychromum
New.ReferenceOTU6144	0	0	0	0	1	2	0	0	0	0	0	0	3	0	0	2	2	0	1	1	0	4	3	0	1	0	1	0	0	0	Fungi; Ascomycota; Sordariomycetes; Hypocreales; Incertae_sedis; Acremonium; Acremonium_polychromum
SH121232.06FU_AB540567_refs	2	3	0	0	4	2	1	1	1	0	1	1	1	3	3	2	6	0	1	1	1	6	9	0	3	0	10	1	1	1	Fungi; Ascomycota; Sordariomycetes; Hypocreales; Incertae_sedis; Acremonium; Acremonium_polychromum
SH456452.06FU_KC427041_reps_singleton	0	0	0	0	0	0	0	0	1	0	0	0	1	0	1	1	1	0	0	2	0	1	1	0	0	0	1	1	1	0	Fungi; Ascomycota; Sordariomycetes; Hypocreales; Incertae_sedis; Acremonium; Acremonium_polychromum
New.ReferenceOTU1500	0	0	0	0	0	0	0	0	0	0	0	0	0	0	0	1	3	0	0	0	0	0	0	0	0	0	0	0	0	0	Fungi; Ascomycota; Sordariomycetes; Hypocreales; Incertae_sedis; Acremonium; Acremonium_sp
New.ReferenceOTU1553	0	1	0	0	0	0	0	0	0	0	0	0	0	0	0	0	0	0	0	0	0	0	0	0	0	0	0	0	0	0	Fungi; Ascomycota; Sordariomycetes; Hypocreales; Incertae_sedis; Acremonium; Acremonium_sp
New.ReferenceOTU1829	0	0	0	0	0	0	0	0	0	0	1	0	0	0	0	0	0	0	0	0	0	0	0	0	0	0	0	0	0	0	Fungi; Ascomycota; Sordariomycetes; Hypocreales; Incertae_sedis; Acremonium; Acremonium_sp
New.ReferenceOTU1894	0	0	0	0	0	0	0	0	0	0	0	0	0	0	1	0	0	0	0	1	1	0	0	0	2	0	0	1	0	0	Fungi; Ascomycota; Sordariomycetes; Hypocreales; Incertae_sedis; Acremonium; Acremonium_sp
New.ReferenceOTU1953	0	0	0	0	0	0	0	0	1	0	0	0	0	0	0	0	0	0	1	0	0	0	0	0	0	0	0	0	0	0	Fungi; Ascomycota; Sordariomycetes; Hypocreales; Incertae_sedis; Acremonium; Acremonium_sp
New.ReferenceOTU2119	0	0	0	0	0	1	1	0	4	2	0	1	0	2	0	1	3	0	0	0	0	0	0	0	2	0	0	0	0	0	Fungi; Ascomycota; Sordariomycetes; Hypocreales; Incertae_sedis; Acremonium; Acremonium_sp
New.ReferenceOTU2447	0	0	0	0	0	0	0	0	0	0	0	0	0	0	0	0	6	0	0	0	0	0	0	0	0	0	0	0	0	0	Fungi; Ascomycota; Sordariomycetes; Hypocreales; Incertae_sedis; Acremonium; Acremonium_sp
New.ReferenceOTU2679	0	0	0	0	0	0	0	0	0	0	0	0	0	0	0	0	0	0	0	0	0	0	0	0	1	0	0	1	0	0	Fungi; Ascomycota; Sordariomycetes; Hypocreales; Incertae_sedis; Acremonium; Acremonium_sp
New.ReferenceOTU2754	0	0	0	0	0	1	0	0	0	0	0	0	0	0	0	0	0	0	0	0	0	0	0	0	0	0	0	0	0	0	Fungi; Ascomycota; Sordariomycetes; Hypocreales; Incertae_sedis; Acremonium; Acremonium_sp
New.ReferenceOTU339	0	0	0	0	0	0	0	0	0	0	0	0	0	2	0	0	0	0	0	0	0	0	0	0	0	0	0	0	0	0	Fungi; Ascomycota; Sordariomycetes; Hypocreales; Incertae_sedis; Acremonium; Acremonium_sp
New.ReferenceOTU3535	0	0	1	0	0	0	0	0	0	0	0	0	0	1	1	1	0	0	0	0	0	0	0	0	0	0	0	0	0	0	Fungi; Ascomycota; Sordariomycetes; Hypocreales; Incertae_sedis; Acremonium; Acremonium_sp
New.ReferenceOTU381	0	0	1	0	0	0	0	0	0	0	0	0	0	0	0	0	0	0	1	0	0	0	0	0	0	0	0	0	0	0	Fungi; Ascomycota; Sordariomycetes; Hypocreales; Incertae_sedis; Acremonium; Acremonium_sp
New.ReferenceOTU4601	0	0	0	2	0	0	0	0	0	0	0	0	1	1	0	0	0	0	0	0	0	4	0	0	0	0	0	0	0	0	Fungi; Ascomycota; Sordariomycetes; Hypocreales; Incertae_sedis; Acremonium; Acremonium_sp
New.ReferenceOTU4823	0	0	0	0	0	0	0	0	0	0	0	0	0	0	0	0	0	0	0	0	0	0	0	0	0	0	4	0	0	0	Fungi; Ascomycota; Sordariomycetes; Hypocreales; Incertae_sedis; Acremonium; Acremonium_sp
New.ReferenceOTU4926	0	1	0	0	0	0	0	0	0	10	0	0	8	17	3	2	8	0	0	0	0	0	0	12	0	0	0	1	0	0	Fungi; Ascomycota; Sordariomycetes; Hypocreales; Incertae_sedis; Acremonium; Acremonium_sp
New.ReferenceOTU516	0	0	4	0	0	0	0	0	0	0	2	0	0	0	0	0	0	0	0	0	0	0	0	0	0	0	0	0	0	0	Fungi; Ascomycota; Sordariomycetes; Hypocreales; Incertae_sedis; Acremonium; Acremonium_sp
New.ReferenceOTU5242	0	0	0	0	0	0	0	0	0	0	0	0	0	0	0	0	0	0	0	0	0	0	0	0	3	0	0	0	0	0	Fungi; Ascomycota; Sordariomycetes; Hypocreales; Incertae_sedis; Acremonium; Acremonium_sp
New.ReferenceOTU5870	0	0	0	0	0	1	0	0	0	0	0	0	0	0	0	0	0	0	0	0	0	1	0	0	0	0	0	0	0	0	Fungi; Ascomycota; Sordariomycetes; Hypocreales; Incertae_sedis; Acremonium; Acremonium_sp
New.ReferenceOTU588	0	0	0	0	0	0	0	0	0	0	0	0	0	0	0	0	0	0	0	0	0	0	0	1	0	0	0	0	0	0	Fungi; Ascomycota; Sordariomycetes; Hypocreales; Incertae_sedis; Acremonium; Acremonium_sp
New.ReferenceOTU6403	0	0	0	0	0	0	0	0	0	0	0	0	0	0	0	0	0	0	0	0	0	0	1	0	0	0	0	0	0	0	Fungi; Ascomycota; Sordariomycetes; Hypocreales; Incertae_sedis; Acremonium; Acremonium_sp
New.ReferenceOTU6543	0	0	0	0	0	0	0	0	0	2	0	0	0	0	0	0	0	0	0	0	0	0	0	0	0	0	0	0	0	0	Fungi; Ascomycota; Sordariomycetes; Hypocreales; Incertae_sedis; Acremonium; Acremonium_sp
New.ReferenceOTU6805	0	0	0	0	0	0	0	0	0	0	0	0	0	0	0	0	0	0	0	3	0	0	0	0	0	0	0	0	0	0	Fungi; Ascomycota; Sordariomycetes; Hypocreales; Incertae_sedis; Acremonium; Acremonium_sp
New.ReferenceOTU6983	0	0	0	0	0	0	0	0	0	0	0	0	0	0	0	0	0	0	0	0	0	0	1	0	0	0	0	0	0	2	Fungi; Ascomycota; Sordariomycetes; Hypocreales; Incertae_sedis; Acremonium; Acremonium_sp
New.ReferenceOTU7720	0	0	0	0	0	0	0	0	0	0	0	0	0	0	0	0	0	0	0	0	0	0	0	0	1	0	0	0	0	1	Fungi; Ascomycota; Sordariomycetes; Hypocreales; Incertae_sedis; Acremonium; Acremonium_sp
New.ReferenceOTU7788	0	0	0	0	0	0	0	0	0	0	0	0	0	0	0	1	0	0	0	0	0	0	0	0	0	0	0	0	0	0	Fungi; Ascomycota; Sordariomycetes; Hypocreales; Incertae_sedis; Acremonium; Acremonium_sp
New.ReferenceOTU7901	0	0	0	0	0	0	0	0	0	0	0	0	0	0	0	0	0	0	0	0	0	2	1	0	1	0	4	0	0	0	Fungi; Ascomycota; Sordariomycetes; Hypocreales; Incertae_sedis; Acremonium; Acremonium_sp
SH112164.06FU_KC007264_reps	0	0	0	0	0	0	0	0	0	0	0	0	0	0	0	0	0	0	0	0	0	0	2	0	0	0	1	0	0	0	Fungi; Ascomycota; Sordariomycetes; Hypocreales; Incertae_sedis; Acremonium; Acremonium_sp
SH126838.06FU_HQ832975_reps	0	2	0	30	0	0	0	0	3	1	0	0	0	0	0	1	0	1	0	0	0	0	1	0	0	0	0	0	0	0	Fungi; Ascomycota; Sordariomycetes; Hypocreales; Incertae_sedis; Acremonium; Acremonium_sp
SH138387.06FU_DQ778915_reps	0	0	0	1	0	0	0	0	0	0	0	0	2	1	0	0	0	0	0	0	0	0	0	0	0	0	0	0	0	0	Fungi; Ascomycota; Sordariomycetes; Hypocreales; Incertae_sedis; Acremonium; Acremonium_sp
SH140464.06FU_EF619706_reps	0	0	0	0	0	1	8	0	1	0	4	3	1	11	0	1	1	0	0	0	0	0	3	0	0	0	2	3	0	2	Fungi; Ascomycota; Sordariomycetes; Hypocreales; Incertae_sedis; Acremonium; Acremonium_sp
New.ReferenceOTU4206	0	0	0	0	0	0	0	0	0	0	0	0	0	0	13	0	0	0	0	0	2	0	6	0	0	0	6	0	0	0	Fungi; Ascomycota; Sordariomycetes; Hypocreales; Incertae_sedis; Acremonium; Acremonium_sp_BCC_14080
New.ReferenceOTU12	0	0	1	0	0	0	0	0	0	0	0	0	0	0	0	0	1	0	1	3	1	0	0	0	3	0	0	0	0	0	Fungi; Ascomycota; Sordariomycetes; Hypocreales; Incertae_sedis; Acremonium; Acremonium_sp_BRO_2013
New.ReferenceOTU5097	0	0	0	0	0	0	0	0	0	0	0	0	0	0	0	0	0	0	0	0	0	0	0	0	0	0	0	2	0	0	Fungi; Ascomycota; Sordariomycetes; Hypocreales; Incertae_sedis; Acremonium; Acremonium_sp_BRO_2013
New.ReferenceOTU6550	0	0	0	0	0	0	0	0	0	0	0	0	0	1	0	1	0	0	0	0	0	0	0	0	0	0	0	0	0	0	Fungi; Ascomycota; Sordariomycetes; Hypocreales; Incertae_sedis; Acremonium; Acremonium_sp_BRO_2013
New.ReferenceOTU1328	0	0	0	0	1	0	0	0	0	0	0	0	0	0	0	0	0	0	0	0	0	0	0	0	0	0	0	0	0	0	Fungi; Ascomycota; Sordariomycetes; Hypocreales; Incertae_sedis; Acremonium; Acremonium_sp_KRCF709
New.ReferenceOTU2574	0	0	0	0	0	0	0	0	0	0	1	0	0	0	0	0	0	0	0	0	0	0	0	0	0	0	0	0	0	0	Fungi; Ascomycota; Sordariomycetes; Hypocreales; Incertae_sedis; Acremonium; Acremonium_sp_KRCF709
New.ReferenceOTU5444	1	1	0	0	0	0	0	0	0	0	0	0	0	0	0	0	0	0	0	0	0	0	0	0	0	0	0	0	0	0	Fungi; Ascomycota; Sordariomycetes; Hypocreales; Incertae_sedis; Acremonium; Acremonium_sp_KRCF709
New.ReferenceOTU5694	0	0	0	0	0	0	0	0	0	1	0	0	0	0	0	0	0	1	0	0	0	0	0	0	0	0	0	0	0	0	Fungi; Ascomycota; Sordariomycetes; Hypocreales; Incertae_sedis; Acremonium; Acremonium_sp_KRCF709
New.ReferenceOTU7392	0	0	0	0	0	0	0	0	0	1	0	1	0	0	0	1	0	0	0	0	0	0	0	0	0	0	0	0	0	0	Fungi; Ascomycota; Sordariomycetes; Hypocreales; Incertae_sedis; Acremonium; Acremonium_sp_KRCF709
New.ReferenceOTU5056	0	0	0	0	0	0	0	0	0	0	0	0	0	0	0	0	0	1	1	0	0	0	0	0	0	0	0	0	0	0	Fungi; Ascomycota; Sordariomycetes; Hypocreales; Incertae_sedis; Acremonium; Acremonium_sp_LB_2012
SH118647.06FU_KC787021_reps	0	0	0	0	0	0	0	0	0	0	0	0	0	0	0	0	0	0	0	0	0	0	0	0	0	0	1	0	0	1	Fungi; Ascomycota; Sordariomycetes; Hypocreales; Incertae_sedis; Acremonium; Acremonium_sp_MIAE1207
New.ReferenceOTU4447	0	0	0	0	2	0	0	0	0	0	0	0	0	0	0	0	0	0	0	0	0	5	0	0	1	0	16	0	0	2	Fungi; Ascomycota; Sordariomycetes; Hypocreales; Incertae_sedis; Acremonium; Acremonium_sp_OY34007
New.ReferenceOTU3014	0	0	0	0	0	0	0	0	0	0	0	0	0	0	0	0	0	0	0	0	0	0	0	0	1	0	0	0	0	0	Fungi; Ascomycota; Sordariomycetes; Hypocreales; Incertae_sedis; Acremonium; Acremonium_sp_R8_9
New.ReferenceOTU6684	0	0	0	0	0	0	2	0	0	1	0	0	0	4	4	7	8	0	0	0	0	0	2	0	0	0	1	0	0	0	Fungi; Ascomycota; Sordariomycetes; Hypocreales; Incertae_sedis; Acremonium; Acremonium_sp_R8_9
SH140181.06FU_JQ988825_reps	0	0	0	0	0	0	0	0	0	0	0	0	0	0	0	0	0	0	0	0	0	0	0	0	1	0	0	0	0	0	Fungi; Ascomycota; Sordariomycetes; Hypocreales; Incertae_sedis; Acremonium; Acremonium_sp_SHW11
New.CleanUp.ReferenceOTU1523	0	0	0	0	0	0	1	0	0	0	0	0	0	0	0	1	0	0	0	0	0	0	0	0	0	0	0	0	0	0	Fungi; Ascomycota; Sordariomycetes; Hypocreales; Incertae_sedis; Acremonium; Acremonium_sp_TR080
New.ReferenceOTU1581	0	0	1	0	0	0	0	0	0	0	0	0	0	0	0	0	0	0	0	0	0	0	0	0	0	0	0	0	0	0	Fungi; Ascomycota; Sordariomycetes; Hypocreales; Incertae_sedis; Acremonium; Acremonium_sp_TR080
New.ReferenceOTU2230	0	0	2	0	0	0	0	0	0	0	0	0	0	0	0	0	0	0	0	0	0	0	0	0	0	0	0	0	0	0	Fungi; Ascomycota; Sordariomycetes; Hypocreales; Incertae_sedis; Acremonium; Acremonium_sp_TR080
New.ReferenceOTU2280	0	1	0	0	0	0	0	0	0	0	1	0	0	0	0	0	0	7	0	1	0	0	0	0	0	0	0	0	0	0	Fungi; Ascomycota; Sordariomycetes; Hypocreales; Incertae_sedis; Acremonium; Acremonium_sp_TR080
New.ReferenceOTU3668	1	0	1	0	0	0	0	0	0	1	0	0	0	2	0	0	0	0	0	0	0	0	0	5	0	0	0	0	0	0	Fungi; Ascomycota; Sordariomycetes; Hypocreales; Incertae_sedis; Acremonium; Acremonium_sp_TR080

New.ReferenceOTU5841	1	0	1	0	0	4	0	0	0	0	0	0	0	0	0	2	0	0	0	0	0	0	0	2	0	0	1	0	0	0	Fungi; Ascomycota; Sordariomycetes; Hypocreales; Incertae_sedis; Acremonium; Acremonium_sp_TR080
New.ReferenceOTU659	0	0	0	0	0	0	0	0	0	0	0	0	0	0	0	0	0	0	0	1	0	0	0	0	0	0	0	0	0	0	Fungi; Ascomycota; Sordariomycetes; Hypocreales; Incertae_sedis; Acremonium; Acremonium_sp_TR080
New.ReferenceOTU7349	4	0	0	0	0	0	0	0	0	0	0	0	0	0	0	0	0	0	0	0	0	0	0	0	0	0	0	0	0	0	Fungi; Ascomycota; Sordariomycetes; Hypocreales; Incertae_sedis; Acremonium; Acremonium_sp_TR080
SH015869.06FU_HQ608111_reps_singleton	0	0	0	2	2	6	0	3	0	0	1	0	0	0	0	1	0	0	0	0	0	0	2	1	0	0	0	0	0	0	Fungi; Ascomycota; Sordariomycetes; Hypocreales; Incertae_sedis; Acremonium; Acremonium_sp_TR080
New.ReferenceOTU2377	1	0	0	0	0	0	0	0	0	0	0	0	0	0	0	0	0	0	0	0	0	2	0	0	0	0	0	0	0	0	Fungi; Ascomycota; Sordariomycetes; Hypocreales; Incertae_sedis; Acremonium; Acremonium_stromaticum
New.ReferenceOTU5075	0	0	0	0	0	0	0	0	0	0	0	0	0	0	0	0	0	0	2	2	0	0	0	0	0	0	0	0	0	0	Fungi; Ascomycota; Sordariomycetes; Hypocreales; Incertae_sedis; Acremonium; Acremonium_stromaticum
New.ReferenceOTU6356	0	0	0	0	0	0	0	0	1	0	1	2	0	0	0	0	0	0	0	0	2	8	2	0	0	0	0	0	0	0	Fungi; Ascomycota; Sordariomycetes; Hypocreales; Incertae_sedis; Acremonium; Acremonium_stromaticum
SH121334.06FU_HQ649874_reps	1	1	0	0	0	0	0	0	1	0	0	2	0	3	0	1	4	2	20	20	20	73	8	1	15	0	6	1	0	0	Fungi; Ascomycota; Sordariomycetes; Hypocreales; Incertae_sedis; Acremonium; Acremonium_stromaticum
SH121353.06FU_DQ825983_reps	0	0	0	0	0	0	0	0	0	0	1	1	0	0	1	0	0	0	0	1	8	7	0	0	1	0	0	2	0	0	Fungi; Ascomycota; Sordariomycetes; Hypocreales; Incertae_sedis; Acremonium; Acremonium_stromaticum
SH121366.06FU_JQ647433_reps	0	0	0	0	1	0	0	0	3	0	1	2	1	1	1	0	0	0	1	0	9	1	1	0	0	0	2	1	0	0	Fungi; Ascomycota; Sordariomycetes; Hypocreales; Incertae_sedis; Acremonium; Acremonium_stromaticum
New.ReferenceOTU4120	0	0	0	0	0	0	0	0	0	0	0	0	0	0	0	0	0	0	0	0	0	0	0	0	0	0	0	1	0	0	Fungi; Ascomycota; Sordariomycetes; Hypocreales; Incertae_sedis; Geosmithia; Geosmithia_sp
New.ReferenceOTU47	0	0	0	0	0	0	0	0	0	0	0	0	0	0	0	0	0	0	0	0	0	0	0	0	0	0	0	0	1	0	Fungi; Ascomycota; Sordariomycetes; Hypocreales; Incertae_sedis; Geosmithia; Geosmithia_sp
New.ReferenceOTU7604	0	0	0	0	0	0	1	0	0	0	0	0	0	0	0	0	0	0	0	0	0	0	0	0	0	0	0	0	0	0	Fungi; Ascomycota; Sordariomycetes; Hypocreales; Incertae_sedis; Geosmithia; Geosmithia_sp_CCF_4206
New.ReferenceOTU3354	0	0	0	0	0	0	0	0	0	0	0	0	0	1	0	0	0	0	1	0	0	0	0	0	0	0	0	0	0	0	Fungi; Ascomycota; Sordariomycetes; Hypocreales; Incertae_sedis; Gliomastix; Gliomastix_murorum
New.ReferenceOTU6135	0	0	0	1	0	0	0	0	0	0	0	0	0	0	0	0	0	0	0	0	0	0	0	0	0	0	0	0	0	0	Fungi; Ascomycota; Sordariomycetes; Hypocreales; Incertae_sedis; Gliomastix; Gliomastix_murorum
New.ReferenceOTU4113	0	0	2	0	0	0	0	0	0	0	0	0	1	0	0	3	2	0	0	0	0	1	0	0	0	0	0	0	0	0	Fungi; Ascomycota; Sordariomycetes; Hypocreales; Incertae_sedis; Myrothecium; Myrothecium_atrum
SH096972.06FU_AY254160_reps	0	0	0	0	0	0	0	0	0	2	0	1	12	2	1	3	12	0	0	0	0	10	0	0	0	0	0	1	0	0	Fungi; Ascomycota; Sordariomycetes; Hypocreales; Incertae_sedis; Myrothecium; Myrothecium_atrum
SH096967.06FU_AY262038_reps	0	0	0	0	0	0	0	0	0	0	0	0	0	0	1	0	0	0	0	0	0	0	0	0	0	0	0	0	0	0	Fungi; Ascomycota; Sordariomycetes; Hypocreales; Incertae_sedis; Myrothecium; Myrothecium_cinctum
New.ReferenceOTU4315	0	0	0	0	0	0	1	2	0	0	0	0	1	0	0	0	0	0	0	0	0	0	0	0	0	0	0	0	0	0	Fungi; Ascomycota; Sordariomycetes; Hypocreales; Incertae_sedis; Myrothecium; Myrothecium_gramineum
New.ReferenceOTU4744	0	0	0	0	0	0	1	0	0	0	0	0	0	0	0	0	0	0	0	0	0	6	0	0	0	0	0	0	0	1	Fungi; Ascomycota; Sordariomycetes; Hypocreales; Incertae_sedis; Myrothecium; Myrothecium_gramineum
SH027334.06FU_HQ608010_reps_singleton	0	0	1	0	0	0	1	0	0	0	0	1	0	1	0	2	6	0	0	0	0	5	0	0	3	0	0	0	0	0	Fungi; Ascomycota; Sordariomycetes; Hypocreales; Incertae_sedis; Myrothecium; Myrothecium_gramineum
SH096979.06FU_FJ825374_reps	0	0	0	0	0	0	0	0	0	0	0	0	0	0	0	1	0	0	0	0	0	0	0	0	1	0	0	0	0	0	Fungi; Ascomycota; Sordariomycetes; Hypocreales; Incertae_sedis; Myrothecium; Myrothecium_gramineum
SH130648.06FU_DQ092526_reps	0	0	0	0	1	0	2	2	0	0	0	0	0	0	0	1	0	0	0	0	0	1	2	0	0	0	0	0	0	0	Fungi; Ascomycota; Sordariomycetes; Hypocreales; Incertae_sedis; Myrothecium; Myrothecium_gramineum
New.ReferenceOTU2183	0	0	0	0	0	0	0	0	0	0	0	0	0	0	0	0	0	0	0	1	0	0	0	0	0	0	0	0	0	0	Fungi; Ascomycota; Sordariomycetes; Hypocreales; Incertae_sedis; Myrothecium; Myrothecium_inundatum
New.ReferenceOTU1236	0	0	0	0	0	0	0	0	0	1	2	0	0	0	0	1	0	0	0	0	0	0	0	0	0	0	0	0	0	0	Fungi; Ascomycota; Sordariomycetes; Hypocreales; Incertae_sedis; Myrothecium; Myrothecium_prestonii
SH124788.06FU_AY254154_refs	0	0	1	0	1	0	0	0	0	3	9	0	0	0	0	0	0	0	0	0	0	0	0	0	0	0	0	0	0	0	Fungi; Ascomycota; Sordariomycetes; Hypocreales; Incertae_sedis; Myrothecium; Myrothecium_prestonii
New.ReferenceOTU1793	0	0	0	0	0	0	0	1	0	0	0	0	0	0	0	0	1	0	0	0	0	1	0	0	3	0	1	0	0	0	Fungi; Ascomycota; Sordariomycetes; Hypocreales; Incertae_sedis; Myrothecium; Myrothecium_roridum
New.ReferenceOTU3943	1	0	0	0	0	0	0	0	0	0	0	0	0	0	0	0	0	0	0	0	0	0	0	0	0	0	0	0	0	0	Fungi; Ascomycota; Sordariomycetes; Hypocreales; Incertae_sedis; Myrothecium; Myrothecium_roridum
New.ReferenceOTU5401	0	0	0	0	0	0	0	0	0	0	0	0	0	0	0	0	0	0	0	0	0	1	0	0	0	0	0	0	0	0	Fungi; Ascomycota; Sordariomycetes; Hypocreales; Incertae_sedis; Myrothecium; Myrothecium_roridum
New.ReferenceOTU6365	0	0	0	0	0	0	0	0	0	0	0	0	0	0	0	0	0	1	1	0	0	0	0	0	0	0	0	0	0	0	Fungi; Ascomycota; Sordariomycetes; Hypocreales; Incertae_sedis; Myrothecium; Myrothecium_roridum
New.ReferenceOTU6862	0	0	0	11	0	0	0	0	0	0	0	0	0	0	0	0	0	0	0	0	0	0	0	0	0	0	0	0	0	0	Fungi; Ascomycota; Sordariomycetes; Hypocreales; Incertae_sedis; Myrothecium; Myrothecium_roridum
SH096969.06FU_AY254150_reps	0	0	0	0	0	0	0	0	0	0	0	1	0	0	1	0	0	0	0	0	1	15	1	0	13	0	9	2	0	0	Fungi; Ascomycota; Sordariomycetes; Hypocreales; Incertae_sedis; Myrothecium; Myrothecium_roridum
New.ReferenceOTU2114	0	0	0	0	0	0	0	0	0	0	0	2	0	0	0	0	0	0	0	0	0	0	0	0	0	0	0	0	0	0	Fungi; Ascomycota; Sordariomycetes; Hypocreales; Incertae_sedis; Myrothecium; Myrothecium_setiramosum
SH023697.06FU_AY254156_reps_singleton	1	0	1	3	18	0	0	3	0	0	0	3	0	0	0	1	0	0	0	0	0	0	0	1	0	0	0	0	0	0	Fungi; Ascomycota; Sordariomycetes; Hypocreales; Incertae_sedis; Myrothecium; Myrothecium_setiramosum
New.ReferenceOTU1336	0	0	0	0	0	0	1	0	0	0	0	1	1	0	0	2	0	0	0	0	0	0	0	0	0	0	0	0	0	0	Fungi; Ascomycota; Sordariomycetes; Hypocreales; Incertae_sedis; Myrothecium; Myrothecium_sp
New.ReferenceOTU5685	0	0	0	0	0	0	0	0	0	0	0	0	0	1	0	0	0	0	0	0	1	3	1	0	7	0	0	2	0	1	Fungi; Ascomycota; Sordariomycetes; Hypocreales; Incertae_sedis; Myrothecium; Myrothecium_sp
New.ReferenceOTU6873	0	0	0	0	0	0	0	0	0	0	0	0	0	0	0	0	0	0	0	0	0	0	1	0	0	0	0	0	0	0	Fungi; Ascomycota; Sordariomycetes; Hypocreales; Incertae_sedis; Myrothecium; Myrothecium_sp
SH096980.06FU_JX156070_reps	0	0	0	0	0	1	0	0	0	0	0	2	0	2	0	0	0	0	0	0	0	0	0	0	0	0	0	0	0	0	Fungi; Ascomycota; Sordariomycetes; Hypocreales; Incertae_sedis; Myrothecium; Myrothecium_sp
New.ReferenceOTU4942	0	0	0	0	0	0	0	0	0	1	0	0	0	0	0	1	2	0	0	0	0	6	3	0	0	0	2	0	0	0	Fungi; Ascomycota; Sordariomycetes; Hypocreales; Incertae_sedis; Myrothecium; Myrothecium_sp_CCN22
SH096982.06FU_DQ092541_reps	0	0	0	0	0	0	0	0	0	0	0	0	0	2	0	5	1	0	0	0	2	11	7	0	1	0	5	8	0	0	Fungi; Ascomycota; Sordariomycetes; Hypocreales; Incertae_sedis; Myrothecium; Myrothecium_sp_CCN22
New.ReferenceOTU6911	0	0	0	0	0	0	0	0	0	1	0	0	0	0	0	0	0	0	0	0	0	0	0	0	0	0	0	0	0	0	Fungi; Ascomycota; Sordariomycetes; Hypocreales; Incertae_sedis; Myrothecium; Myrothecium_sp_cp0024d
New.ReferenceOTU2469	0	0	0	0	0	0	0	0	0	0	0	0	0	0	0	0	0	0	0	0	0	0	0	0	0	0	1	0	0	0	Fungi; Ascomycota; Sordariomycetes; Hypocreales; Incertae_sedis; Myrothecium; Myrothecium_sp_HZ_6
SH096965.06FU_EU301637_reps	0	0	0	0	0	0	0	0	0	0	0	0	0	0	0	0	0	0	0	0	0	0	1	0	0	0	0	0	0	0	Fungi; Ascomycota; Sordariomycetes; Hypocreales; Incertae_sedis; Myrothecium; Myrothecium_sp_HZ_6
New.ReferenceOTU7287	0	1	0	0	0	0	17	0	0	6	0	0	4	0	9	6	9	0	0	0	3	4	1	2	2	0	8	3	0	4	Fungi; Ascomycota; Sordariomycetes; Hypocreales; Incertae_sedis; Myrothecium; Myrothecium_sp_NF_08
SH096964.06FU_HM347342_reps	0	0	0	0	0	0	8	3	0	2	0	4	7	13	24	5	3	0	0	0	9	15	8	0	10	0	6	30	0	12	Fungi; Ascomycota; Sordariomycetes; Hypocreales; Incertae_sedis; Myrothecium; Myrothecium_sp_NF_08
SH096966.06FU_KC806230_reps	0	0	0	0	0	0	3	0	0	1	0	0	0	0	2	1	3	0	0	0	2	0	1	0	1	0	2	4	0	5	Fungi; Ascomycota; Sordariomycetes; Hypocreales; Incertae_sedis; Myrothecium; Myrothecium_sp_NF_08
New.ReferenceOTU3371	0	0	0	2	0	0	0	0	1	0	0	0	0	0	0	0	0	0	0	0	0	0	0	0	0	0	1	0	0	0	Fungi; Ascomycota; Sordariomycetes; Hypocreales; Incertae_sedis; Stachybotrys; aff_Stachybotrys_sp_LA227
New.ReferenceOTU5587	0	0	0	1	0	0	0	0	0	0	0	0	0	0	1	0	0	0	0	0	0	1	0	0	0	0	0	0	0	0	Fungi; Ascomycota; Sordariomycetes; Hypocreales; Incertae_sedis; Stachybotrys; aff_Stachybotrys_sp_LA227
SH452784.06FU_HQ022508_reps_singleton	0	0	0	0	0	0	0	0	0	0	0	0	0	0	2	0	0	0	0	0	1	2	0	0	0	0	0	0	0	0	Fungi; Ascomycota; Sordariomycetes; Hypocreales; Incertae_sedis; Stachybotrys; aff_Stachybotrys_sp_LA227
New.ReferenceOTU2601	0	0	0	1	0	0	0	0	0	4	0	0	0	0	0	0	0	0	2	0	0	0	0	0	0	0	0	0	0	0	Fungi; Ascomycota; Sordariomycetes; Hypocreales; Incertae_sedis; Stachybotrys; Stachybotrys_cf_elegans_HGUP_0310
New.ReferenceOTU496	0	0	0	0	0	0	0	0	0	0	0	0	0	0	0	0	0	0	5	2	0	0	0	0	0	0	0	0	0	0	Fungi; Ascomycota; Sordariomycetes; Hypocreales; Incertae_sedis; Stachybotrys; Stachybotrys_cf_elegans_HGUP_0310
New.ReferenceOTU1255	0	0	0	0	0	0	0	0	1	0	0	0	0	0	0	0	0	0	0	0	0	0	0	0	0	0	1	0	0	0	Fungi; Ascomycota; Sordariomycetes; Hypocreales; Incertae_sedis; Stachybotrys; Stachybotrys_kampalensis
New.ReferenceOTU1151	0	0	0	0	0	0	0	0	2	0	0	0	0	0	0	0	0	0	0	0	0	0	0	0	0	0	0	0	0	0	Fungi; Ascomycota; Sordariomycetes; Hypocreales; Incertae_sedis; Stachybotrys; Stachybotrys_nephrospora
New.ReferenceOTU1036	0	0	0	0	0	0	0	0	0	0	0	0	0	0	0	0	0	0	0	1	0	0	0	0	0	0	0	0	0	0	Fungi; Ascomycota; Sordariomycetes; Hypocreales; Incertae_sedis; Stachybotrys; Stachybotrys_parvispora
New.ReferenceOTU1658	0	1	0	0	0	1	0	0	0	0	0	0	0	0	0	1	0	0	0	0	0	0	0	2	0	0	0	0	0	0	Fungi; Ascomycota; Sordariomycetes; Hypocreales; Incertae_sedis; Stachybotrys; Stachybotrys_parvispora
New.ReferenceOTU2894	0	0	0	0	0	0	0	0	0	0	0	0	0	0	0	0	0	0	1	0	0	0	0	0	0	0	0	0	0	0	Fungi; Ascomycota; Sordariomycetes; Hypocreales; Incertae_sedis; Stachybotrys; Stachybotrys_parvispora
New.ReferenceOTU322	2	0	0	0	0	0	0	0	0	0	0	0	0	0	0	0	0	0	0	0	0	0	0	0	0	0	0	0	0	0	Fungi; Ascomycota; Sordariomycetes; Hypocreales; Incertae_sedis; Stachybotrys; Stachybotrys_parvispora
New.ReferenceOTU3268	0	0	0	0	0	0	3	0	0	0	0	0	0	0	0	0	0	0	0	0	0	0	0	0	0	0	0	0	0	0	Fungi; Ascomycota; Sordariomycetes; Hypocreales; Incertae_sedis; Stachybotrys; Stachybotrys_parvispora
New.ReferenceOTU3545	0	0	0	0	0	0	2	0	0	0	0	0	0	0	0	0	0	0	0	0	0	0	0	0	0	0	0	0	0	0	Fungi; Ascomycota; Sordariomycetes; Hypocreales; Incertae_sedis; Stachybotrys; Stachybotrys_parvispora
New.ReferenceOTU4347	0	0	0	0	0	1	0	0	0	1	0	0	0	0	0	1	0	0	0	0	0	0	0	0	0	0	0	0	0	0	Fungi; Ascomycota; Sordariomycetes; Hypocreales; Incertae_sedis; Stachybotrys; Stachybotrys_parvispora
New.ReferenceOTU4411	0	0	0	1	0	0	0	0	0	0	0	0	0	0	0	0	0	0	0	0	0	0	0	0	0	0	0	0	0	0	Fungi; Ascomycota; Sordariomycetes; Hypocreales; Incertae_sedis; Stachybotrys; Stachybotrys_parvispora
New.ReferenceOTU6081	4	4	0	0	0	3	0	1	0	1	1	0	0	1	0	1	0	0	0	0	0	0	0	7	0	0	0	0	0	0	Fungi; Ascomycota; Sordariomycetes; Hypocreales; Incertae_sedis; Stachybotrys; Stachybotrys_parvispora
New.ReferenceOTU6095	1	0	0	1	0	1	0	0	0	0	0	0	0	0	0	0	0	0	0	4	0	0	0	0	0	0	0	0	0	0	Fungi; Ascomycota; Sordariomycetes; Hypocreales; Incertae_sedis; Stachybotrys; Stachybotrys_parvispora
SH027336.06FU_AF081483_reps_singleton	0	0	0	0	0	0	0	0	0	0	0	0	0	0	0	1	0	0	0	0	0	0	0	0	0	0	0	0	0	0	Fungi; Ascomycota; Sordariomycetes; Hypocreales; Incertae_sedis; Stachybotrys; Stachybotrys_parvispora
New.ReferenceOTU2403	0	0	0	0	0	0	0	0	0	0	0	0	0	0	2	0	0	0	0	0	0	9	3	0	0	0	2	0	0	3	Fungi; Ascomycota; Sordariomycetes; Hypocreales; Incertae_sedis; Stachybotrys; Stachybotrys_sp_HH1
New.ReferenceOTU2646	0	0	0	0	0	0	0	0	0	0	0	0	0	0	0	0	0	0	0	0	0	0	0	0	1	0	0	0	0	1	Fungi; Ascomycota; Sordariomycetes; Hypocreales; Incertae_sedis; Stachybotrys; Stachybotrys_sp_HH1
SH027329.06FU_AF081471_reps_singleton	0	0	0	0	0	0	0	0	0	0	0	0	0	0	0	0	1	0	0	0	0	0	0	0	5	0	1	0	0	0	Fungi; Ascomycota; Sordariomycetes; Hypocreales; Incertae_sedis; Stachybotrys; Stachybotrys_sp_HH1
New.ReferenceOTU7337	0	0	0	0	0	0	0	0	1	0	0	0	0	0	0	0	0	0	0	0	0	0	0	0	0	0	0	0	0	0	Fungi; Ascomycota; Sordariomycetes; Hypocreales; Incertae_sedis; Stilbella; Stilbella_sp
SH118627.06FU_EF619702_reps	0	1	0	0	0	0	0	0	0	1	10	0	0	7	2	0	13	0	0	2	0	0	0	17	0	1	0	9	0	1	Fungi; Ascomycota; Sordariomycetes; Hypocreales; Incertae_sedis; Stilbella; Stilbella_sp
New.ReferenceOTU2346	0	0	0	0	0	0	0	0	0	0	0	0	0	0	0	0	0	0	0	0	0	1	1	0	7	0	0	0	0	0	Fungi; Ascomycota; Sordariomycetes; Hypocreales; Incertae_sedis; unidentified; Hypocreales_sp
New.ReferenceOTU38	0	0	0	0	0	0	0	0	1	0	1	0	0	0	0	0	0	0	0	0	0	0	0	0	0	0	0	0	0	0	Fungi; Ascomycota; Sordariomycetes; Hypocreales; Incertae_sedis; unidentified; Hypocreales_sp
SH087384.06FU_AY214439_refs	0	0	0	0	0	0	0	0	0	0	0	0	0	0	0	0	1	0	0	0	0	0	1	0	0	0	0	0	0	0	Fungi; Ascomycota; Sordariomycetes; Hypocreales; Incertae_sedis; unidentified; Hypocreales_sp
New.ReferenceOTU5261	0	0	0	0	1	0	0	0	0	0	0	0	0	0	0	0	0	0	0	0	0	0	0	0	0	0	0	0	0	0	Fungi; Ascomycota; Sordariomycetes; Hypocreales; Nectriaceae; Flagellospora; Flagellospora_saccata
New.ReferenceOTU4933	0	0	0	0	1	0	0	0	0	0	0	0	0	0	0	0	0	0	0	0	0	0	0	0	0	0	0	0	0	0	Fungi; Ascomycota; Sordariomycetes; Hypocreales; Nectriaceae; Fusicolla; Fusicolla_violacea
New.ReferenceOTU5822	0	0	0	0	0	0	0	0	0	0	0	0	0	0	0	0	1	0	0	0	0	0	0	0	0	0	0	0	0	0	Fungi; Ascomycota; Sordariomycetes; Hypocreales; Nectriaceae; Fusicolla; Fusicolla_violacea
New.ReferenceOTU2137	0	0	0	0	0	0	0	0	0	0	0	0	0	0	0	0	0	0	0	0	0	0	0	0	3	0	0	0	0	0	Fungi; Ascomycota; Sordariomycetes; Hypocreales; Nectriaceae; Gliocephalotrichum; Gliocephalotrichum_bulbilium
SH019813.06FU_AB666512_reps_singleton	0	0	0	0	0	0	0	0	0	0	0	0	0	0	0	0	0	0	1	0	0	0	0	0	0	0	0	0	0	0	Fungi; Ascomycota; Sordariomycetes; Hypocreales; Nectriaceae; Gliocephalotrichum; Gliocephalotrichum_simplex
SH118591.06FU_DQ278423_reps	0	0	0	0	0	0	0	0	0	0	0	0	0	1	0	0	0	0	4	0	0	0	0	0	0	0	0	0	0	0	Fungi; Ascomycota; Sordariomycetes; Hypocreales; Nectriaceae; Gliocephalotrichum; Gliocephalotrichum_simplex
SH118593.06FU_JN628172_reps	0	1	0	0	0	0	0	0	0	0	0	0	0	0	0	0	0	0	6	0	0	0	0	0	0	0	0	0	0	0	Fungi; Ascomycota; Sordariomycetes; Hypocreales; Nectriaceae; Gliocephalotrichum; Gliocephalotrichum_simplex
SH118594.06FU_JX484850_reps	0	0	0	0	0	0	0	0	0	0	0	0	0	0	0	0	0	0	2	0	0	0	0	0	0	0	0	0	0	0	Fungi; Ascomycota; Sordariomycetes; Hypocreales; Nectriaceae; Gliocephalotrichum; Gliocephalotrichum_simplex
SH118631.06FU_EU563601_reps	0	0	0	1	0	0	0	0	0	0	0	0	0	0	0	0	0	0	0	0	0	0	0	0	0	0	0	1	0	0	Fungi; Ascomycota; Sordariomycetes; Hypocreales; Nectriaceae; Gliocladiopsis; Gliocladiopsis_curvata
New.ReferenceOTU2848	0	0	0	0	0	0	0	0	2	1	0	0	0	0	0	0	0	0	1	2	0	0	0	0	0	0	0	0	0	0	Fungi; Ascomycota; Sordariomycetes; Hypocreales; Nectriaceae; Nalanthamala; Nalanthamala_vermoesenii
New.ReferenceOTU930	0	1	0	0	0	0	0	0	0	4	0	0	0	1	0	1	0	0	0	0	0	0	0	4	0	0	0	0	0	0	Fungi; Ascomycota; Sordariomycetes; Hypocreales; Nectriaceae; Pseudonectria; Pseudonectria_pachysandricola
New.ReferenceOTU395	0	0	0	0	0	0	0	0	0	0	0	0	0	0	0	0	0	0	0	0	0	0	2	0	0	0	0	0	0	0	Fungi; Ascomycota; Sordariomycetes; Hypocreales; Nectriaceae; Pseudonectria; Pseudonectria_sp_AUMC_6707
New.CleanUp.ReferenceOTU101	0	0	0	0	0	0	0	0	0	0	0	0	0	0	0	0	0	0	1	0	0	0	0	0	0	0	0	0	0	0	Fungi; Ascomycota; Sordariomycetes; Hypocreales; Nectriaceae; unidentified; Nectriaceae_sp
New.CleanUp.ReferenceOTU595	0	0	0	0	0	0	0	0	0	0	0	0	0	0	0	0	0	0	0	0	0	0	0	0	0	0	0	0	1	0	Fungi; Ascomycota; Sordariomycetes; Hypocreales; Nectriaceae; unidentified; Nectriaceae_sp
New.ReferenceOTU1013	0	2	0	0	0	0	0	0	0	0	0	0	0	0	0	0	0	0	0	0	0	0	0	0	0	0	0	0	0	0	Fungi; Ascomycota; Sordariomycetes; Hypocreales; Nectriaceae; unidentified; Nectriaceae_sp
New.ReferenceOTU1069	0	0	0	0	0	0	0	0	0	0	0	0	0	0	0	0	0	0	0	0	0	0	1	0	0	0	0	0	0	0	Fungi; Ascomycota; Sordariomycetes; Hypocreales; Nectriaceae; unidentified; Nectriaceae_sp
New.ReferenceOTU1078	0	3	0	2	0	0	1	0	0	1	0	0	0	3	0	1	0	1	0	0	1	0	0	1	0	0	0	0	0	0	Fungi; Ascomycota; Sordariomycetes; Hypocreales; Nectriaceae; unidentified; Nectriaceae_sp
New.ReferenceOTU1170	0	0	0	0	0	0	0	0	0	0	0	0	0	0	0	0	0	1	6	0	0	0	0	0	0	0	0	0	0	0	Fungi; Ascomycota; Sordariomycetes; Hypocreales; Nectriaceae; unidentified; Nectriaceae_sp
New.ReferenceOTU1563	0	0	0	0	0	0	0	0	0	0	1	0	0	0	0	0	0	0	0	0	1	0	1	0	0	0	0	0	0	0	Fungi; Ascomycota; Sordariomycetes; Hypocreales; Nectriaceae; unidentified; Nectriaceae_sp
New.ReferenceOTU1631	0	0	1	0	0	0	7	0	0	1	0	0	0	0	1	1	0	0	0	5	4	0	0	2	0	0	0	0	5	0	Fungi; Ascomycota; Sordariomycetes; Hypocreales; Nectriaceae; unidentified; Nectriaceae_sp
New.ReferenceOTU1766	0	2	0	0	0	0	0	0	0	0	0	0	0	0	0	0	0	0	0	0	0	0	0	0	0	0	0	0	0	0	Fungi; Ascomycota; Sordariomycetes; Hypocreales; Nectriaceae; unidentified; Nectriaceae_sp
New.ReferenceOTU2263	12	36	7	10	0	30	29	4	1	38	11	1	1	5	15	50	4	23	9	1	43	0	9	36	8	0	2	2	6	2	Fungi; Ascomycota; Sordariomycetes; Hypocreales; Nectriaceae; unidentified; Nectriaceae_sp
New.ReferenceOTU2630	0	0	0	0	0	0	0	0	0	0	0	0	0	0	0	1	0	0	0	0	0	0	0	0	0	0	0	0	0	0	Fungi; Ascomycota; Sordariomycetes; Hypocreales; Nectriaceae; unidentified; Nectriaceae_sp
New.ReferenceOTU2644	0	0	0	0	0	0	1	0	0	0	0	0	0	0	0	0	0	0	0	0	0	0	0	0	0	0	0	0	0	0	Fungi; Ascomycota; Sordariomycetes; Hypocreales; Nectriaceae; unidentified; Nectriaceae_sp
New.ReferenceOTU2813	0	0	0	0	0	0	4	0	0	0	0	0	0	0	0	0	0	0	0	0	3	0	0	0	1	0	0	0	0	0	Fungi; Ascomycota; Sordariomycetes; Hypocreales; Nectriaceae; unidentified; Nectriaceae_sp
New.ReferenceOTU3385	0	1	0	0	0	3	3	0	0	0	0	0	0	0	0	0	0	0	0	0	0	0	0	0	0	0	0	0	0	0	Fungi; Ascomycota; Sordariomycetes; Hypocreales; Nectriaceae; unidentified; Nectriaceae_sp
New.ReferenceOTU3642	0	0	0	0	0	0	0	0	0	0	0	0	0	0	0	0	0	0	0	0	1	0	0	0	0	0	0	0	0	0	Fungi; Ascomycota; Sordariomycetes; Hypocreales; Nectriaceae; unidentified; Nectriaceae_sp
New.ReferenceOTU3684	0	0	0	0	0	0	1	0	0	0	0	0	0	0	0	0	0	0	0	0	0	0	0	0	0	0	0	0	0	0	Fungi; Ascomycota; Sordariomycetes; Hypocreales; Nectriaceae; unidentified; Nectriaceae_sp

New.ReferenceOTU3691	0	0	0	0	0	0	0	0	0	0	0	0	0	0	0	1	0	0	0	0	0	0	0	0	0	0	0	0	0	0	Fungi; Ascomycota; Sordariomycetes; Hypocreales; Nectriaceae; unidentified; Nectriaceae_sp
New.ReferenceOTU3931	0	0	0	0	0	0	0	0	0	0	0	0	0	0	1	0	0	0	0	0	1	0	0	0	0	0	0	0	0	0	Fungi; Ascomycota; Sordariomycetes; Hypocreales; Nectriaceae; unidentified; Nectriaceae_sp
New.ReferenceOTU4126	0	0	0	0	0	0	0	0	0	0	0	0	0	0	0	0	0	1	1	0	0	0	0	0	0	0	0	0	0	0	Fungi; Ascomycota; Sordariomycetes; Hypocreales; Nectriaceae; unidentified; Nectriaceae_sp
New.ReferenceOTU4135	1	0	1	0	0	0	0	0	0	0	0	0	0	0	1	0	0	1	0	0	0	0	0	0	0	0	0	0	0	0	Fungi; Ascomycota; Sordariomycetes; Hypocreales; Nectriaceae; unidentified; Nectriaceae_sp
New.ReferenceOTU4509	0	0	0	0	0	0	0	0	0	0	0	0	0	0	0	0	0	0	0	0	0	0	0	0	1	0	0	0	0	0	Fungi; Ascomycota; Sordariomycetes; Hypocreales; Nectriaceae; unidentified; Nectriaceae_sp
New.ReferenceOTU4994	0	0	0	0	0	0	0	0	0	0	1	0	0	0	0	0	0	0	0	0	0	0	0	0	0	0	0	0	0	0	Fungi; Ascomycota; Sordariomycetes; Hypocreales; Nectriaceae; unidentified; Nectriaceae_sp
New.ReferenceOTU5145	0	0	0	0	0	1	6	1	0	2	0	0	0	0	0	1	0	0	0	0	0	0	1	0	0	0	0	0	1	0	Fungi; Ascomycota; Sordariomycetes; Hypocreales; Nectriaceae; unidentified; Nectriaceae_sp
New.ReferenceOTU5376	0	0	0	0	0	0	0	0	0	0	0	0	0	0	0	2	0	0	0	0	0	0	0	0	0	0	0	0	0	0	Fungi; Ascomycota; Sordariomycetes; Hypocreales; Nectriaceae; unidentified; Nectriaceae_sp
New.ReferenceOTU5795	0	0	0	0	0	0	0	0	0	0	0	0	0	0	0	0	0	0	0	0	1	0	0	0	0	0	1	0	0	0	Fungi; Ascomycota; Sordariomycetes; Hypocreales; Nectriaceae; unidentified; Nectriaceae_sp
New.ReferenceOTU6340	0	0	0	0	0	0	1	0	0	1	0	0	0	0	0	0	0	0	0	0	0	0	0	0	0	0	0	0	0	0	Fungi; Ascomycota; Sordariomycetes; Hypocreales; Nectriaceae; unidentified; Nectriaceae_sp
New.ReferenceOTU6461	0	0	0	0	0	0	0	0	0	0	0	0	0	0	0	0	0	0	0	0	0	0	0	0	2	0	0	0	0	0	Fungi; Ascomycota; Sordariomycetes; Hypocreales; Nectriaceae; unidentified; Nectriaceae_sp
New.ReferenceOTU6614	0	0	0	0	0	0	3	0	0	0	0	0	0	0	0	0	0	0	0	0	1	0	0	0	0	0	0	0	0	0	Fungi; Ascomycota; Sordariomycetes; Hypocreales; Nectriaceae; unidentified; Nectriaceae_sp
New.ReferenceOTU6657	0	0	0	0	0	0	0	0	0	0	0	0	0	0	0	1	0	0	0	0	0	0	0	0	0	0	0	0	0	0	Fungi; Ascomycota; Sordariomycetes; Hypocreales; Nectriaceae; unidentified; Nectriaceae_sp
New.ReferenceOTU7380	0	0	0	0	0	0	0	0	0	1	0	0	0	0	0	0	0	0	0	0	0	0	0	0	0	0	0	0	0	0	Fungi; Ascomycota; Sordariomycetes; Hypocreales; Nectriaceae; unidentified; Nectriaceae_sp
New.ReferenceOTU7573	0	0	0	0	0	0	0	0	0	0	0	1	0	0	0	0	0	0	0	2	3	0	0	0	0	0	0	0	0	0	Fungi; Ascomycota; Sordariomycetes; Hypocreales; Nectriaceae; unidentified; Nectriaceae_sp
SH038043.06FU_AF502877_reps_singleton	0	0	0	0	0	0	0	0	0	0	0	0	0	0	0	0	0	0	1	0	0	0	0	0	0	0	0	0	0	0	Fungi; Ascomycota; Sordariomycetes; Hypocreales; Nectriaceae; unidentified; Nectriaceae_sp
SH121121.06FU_KC427033_reps	0	0	0	0	0	0	0	0	0	0	0	0	0	2	0	0	0	0	0	0	0	0	0	0	0	0	0	0	0	0	Fungi; Ascomycota; Sordariomycetes; Hypocreales; Nectriaceae; unidentified; Nectriaceae_sp
SH137920.06FU_DQ094747_reps	0	0	1	0	0	0	0	0	0	0	0	0	0	0	0	0	0	0	0	0	0	0	0	0	0	0	0	0	0	0	Fungi; Ascomycota; Sordariomycetes; Hypocreales; Nectriaceae; unidentified; Nectriaceae_sp
SH146531.06FU_HQ023180_reps	0	0	0	1	0	0	0	0	0	0	0	0	0	0	0	0	0	0	0	0	0	0	0	0	0	0	0	0	0	0	Fungi; Ascomycota; Sordariomycetes; Hypocreales; Nectriaceae; unidentified; Nectriaceae_sp
New.ReferenceOTU171	0	0	0	0	0	0	0	0	0	0	0	0	0	0	0	0	0	0	1	0	0	0	0	0	0	0	0	0	0	0	Fungi; Ascomycota; Sordariomycetes; Hypocreales; Nectriaceae; unidentified; Nectriaceae_sp_GJS_95_143
New.ReferenceOTU3744	0	0	0	0	0	0	0	0	0	0	1	0	0	0	0	0	0	0	0	1	0	0	0	0	0	0	0	0	0	0	Fungi; Ascomycota; Sordariomycetes; Hypocreales; Nectriaceae; unidentified; Nectriaceae_sp_GJS_95_143
New.ReferenceOTU2602	0	2	0	0	3	2	0	0	0	1	0	0	1	1	0	0	1	0	0	0	0	0	0	2	1	0	0	0	0	3	Fungi; Ascomycota; Sordariomycetes; Hypocreales; Nectriaceae; Viridispora; Viridispora_sp
New.ReferenceOTU3312	0	0	0	0	1	0	0	0	0	0	0	0	1	0	0	0	1	0	0	0	1	0	0	0	0	0	1	0	1	0	Fungi; Ascomycota; Sordariomycetes; Hypocreales; Nectriaceae; Viridispora; Viridispora_sp
New.ReferenceOTU6070	0	4	2	0	4	0	0	0	0	0	0	1	2	2	1	0	0	0	0	1	0	0	0	2	3	0	1	0	3	3	Fungi; Ascomycota; Sordariomycetes; Hypocreales; Nectriaceae; Viridispora; Viridispora_sp
New.ReferenceOTU6482	0	0	0	0	0	0	0	0	0	0	0	0	2	0	0	0	0	0	0	0	0	0	0	0	0	0	0	0	0	0	Fungi; Ascomycota; Sordariomycetes; Hypocreales; Nectriaceae; Viridispora; Viridispora_sp
New.ReferenceOTU7808	0	3	0	0	2	1	2	0	0	3	0	1	1	1	1	2	0	0	0	1	1	0	1	1	0	0	0	6	0	2	Fungi; Ascomycota; Sordariomycetes; Hypocreales; Nectriaceae; Viridispora; Viridispora_sp
New.CleanUp.ReferenceOTU1350	2	0	0	0	0	0	0	0	0	0	0	0	0	0	0	0	0	0	0	0	0	0	0	0	0	0	0	0	0	0	Fungi; Ascomycota; Sordariomycetes; Hypocreales; Ophiocordycipitaceae; unidentified; Ophiocordycipitaceae_sp
New.ReferenceOTU1188	9	0	5	4	4	0	0	1	1	0	0	2	0	0	0	5	0	6	0	0	0	0	0	0	0	0	2	0	0	0	Fungi; Ascomycota; Sordariomycetes; Hypocreales; Ophiocordycipitaceae; unidentified; Ophiocordycipitaceae_sp
New.ReferenceOTU1949	0	0	2	0	0	0	0	0	0	0	0	0	1	0	0	0	0	0	0	0	0	0	0	1	0	0	1	0	0	0	Fungi; Ascomycota; Sordariomycetes; Hypocreales; Ophiocordycipitaceae; unidentified; Ophiocordycipitaceae_sp
New.ReferenceOTU2628	0	0	0	0	0	0	0	0	0	0	0	0	0	0	0	0	0	0	2	0	0	0	0	0	0	0	0	0	0	0	Fungi; Ascomycota; Sordariomycetes; Hypocreales; Ophiocordycipitaceae; unidentified; Ophiocordycipitaceae_sp
New.ReferenceOTU3199	0	0	0	0	0	0	0	0	0	0	0	0	0	0	0	3	0	0	0	0	0	0	0	0	0	0	0	0	0	0	Fungi; Ascomycota; Sordariomycetes; Hypocreales; Ophiocordycipitaceae; unidentified; Ophiocordycipitaceae_sp
New.ReferenceOTU357	0	0	2	1	0	0	0	0	0	0	0	0	0	0	0	1	0	0	0	0	0	0	0	0	0	0	0	0	0	0	Fungi; Ascomycota; Sordariomycetes; Hypocreales; Ophiocordycipitaceae; unidentified; Ophiocordycipitaceae_sp
New.ReferenceOTU4868	0	0	0	0	0	0	0	0	0	0	0	0	0	0	0	0	0	0	1	1	0	0	0	0	0	0	0	0	0	0	Fungi; Ascomycota; Sordariomycetes; Hypocreales; Ophiocordycipitaceae; unidentified; Ophiocordycipitaceae_sp
New.ReferenceOTU4978	0	0	0	0	0	0	0	0	0	1	0	0	0	0	0	0	0	0	0	0	0	0	0	0	0	0	0	0	0	0	Fungi; Ascomycota; Sordariomycetes; Hypocreales; Ophiocordycipitaceae; unidentified; Ophiocordycipitaceae_sp
New.ReferenceOTU6491	0	0	0	0	0	0	0	0	0	0	0	0	0	0	0	0	0	1	0	0	0	0	0	0	0	0	0	0	0	0	Fungi; Ascomycota; Sordariomycetes; Hypocreales; Ophiocordycipitaceae; unidentified; Ophiocordycipitaceae_sp
New.ReferenceOTU672	0	0	1	0	0	0	0	0	0	0	0	0	0	0	0	1	0	1	0	0	0	0	0	0	0	0	0	0	0	0	Fungi; Ascomycota; Sordariomycetes; Hypocreales; Ophiocordycipitaceae; unidentified; Ophiocordycipitaceae_sp
New.ReferenceOTU7056	0	0	0	0	0	0	0	0	0	0	0	0	0	0	0	0	0	0	4	0	0	0	0	0	0	0	0	0	1	0	Fungi; Ascomycota; Sordariomycetes; Hypocreales; Ophiocordycipitaceae; unidentified; Ophiocordycipitaceae_sp
New.ReferenceOTU7906	0	0	0	0	0	0	0	0	0	0	0	0	0	0	0	0	0	0	0	1	0	0	0	0	0	0	0	0	0	0	Fungi; Ascomycota; Sordariomycetes; Hypocreales; Ophiocordycipitaceae; unidentified; Ophiocordycipitaceae_sp
SH005982.06FU_JQ905655_reps_singleton	0	0	0	0	0	0	0	0	0	0	0	0	0	0	0	0	0	0	0	0	0	0	0	0	0	0	0	0	1	0	Fungi; Ascomycota; Sordariomycetes; Hypocreales; Ophiocordycipitaceae; unidentified; Ophiocordycipitaceae_sp
SH095127.06FU_AF389191_refs	0	0	2	0	0	0	0	0	0	0	1	0	0	0	0	0	0	0	0	0	0	0	0	0	0	0	0	0	0	0	Fungi; Ascomycota; Sordariomycetes; Hypocreales; Ophiocordycipitaceae; unidentified; Ophiocordycipitaceae_sp
SH095131.06FU_JX155907_reps	4	5	18	25	24	0	0	1	0	20	1	4	0	2	1	9	0	0	0	1	0	0	0	1	0	0	3	0	0	0	Fungi; Ascomycota; Sordariomycetes; Hypocreales; Ophiocordycipitaceae; unidentified; Ophiocordycipitaceae_sp
SH095134.06FU_JQ411376_reps	1	0	6	1	6	0	0	7	0	0	0	1	6	6	0	5	3	0	0	2	0	1	0	0	0	0	0	2	0	1	Fungi; Ascomycota; Sordariomycetes; Hypocreales; Ophiocordycipitaceae; unidentified; Ophiocordycipitaceae_sp
SH095141.06FU_JX155971_reps	0	0	5	2	1	0	0	0	0	2	1	0	0	0	0	2	0	2	0	0	0	0	0	0	0	0	0	0	0	0	Fungi; Ascomycota; Sordariomycetes; Hypocreales; Ophiocordycipitaceae; unidentified; Ophiocordycipitaceae_sp
SH139736.06FU_DQ078757_reps	0	0	0	0	0	0	0	0	0	0	0	0	0	1	0	0	0	0	0	0	0	0	0	0	0	0	0	0	0	0	Fungi; Ascomycota; Sordariomycetes; Hypocreales; Ophiocordycipitaceae; unidentified; Ophiocordycipitaceae_sp
New.CleanUp.ReferenceOTU1079	0	0	1	0	0	0	0	0	0	0	0	0	0	0	0	0	0	0	0	0	0	0	0	0	0	0	0	0	0	0	Fungi; Ascomycota; Sordariomycetes; Hypocreales; unidentified; unidentified; Hypocreales_sp
New.CleanUp.ReferenceOTU1709	0	1	0	0	0	0	0	0	0	0	0	0	0	0	0	0	0	0	0	0	0	0	0	0	0	0	0	0	0	0	Fungi; Ascomycota; Sordariomycetes; Hypocreales; unidentified; unidentified; Hypocreales_sp
New.CleanUp.ReferenceOTU2002	0	0	0	0	0	0	0	0	0	0	0	0	0	0	0	0	0	0	0	0	0	0	0	0	0	0	0	0	1	0	Fungi; Ascomycota; Sordariomycetes; Hypocreales; unidentified; unidentified; Hypocreales_sp
New.CleanUp.ReferenceOTU2105	0	0	0	0	0	0	0	0	1	0	0	0	0	0	0	0	0	0	0	0	0	0	0	0	0	0	0	0	0	0	Fungi; Ascomycota; Sordariomycetes; Hypocreales; unidentified; unidentified; Hypocreales_sp
New.CleanUp.ReferenceOTU2214	0	0	0	0	0	0	0	0	0	0	0	0	0	0	0	0	0	3	0	0	0	0	0	0	0	0	0	0	0	0	Fungi; Ascomycota; Sordariomycetes; Hypocreales; unidentified; unidentified; Hypocreales_sp
New.CleanUp.ReferenceOTU894	0	0	0	0	0	0	0	0	0	0	0	0	0	0	0	0	0	1	0	0	0	0	0	0	0	0	0	0	0	0	Fungi; Ascomycota; Sordariomycetes; Hypocreales; unidentified; unidentified; Hypocreales_sp
New.ReferenceOTU1046	0	0	1	0	0	0	0	0	0	0	5	0	0	0	0	3	0	0	0	0	0	0	0	0	0	0	0	0	0	0	Fungi; Ascomycota; Sordariomycetes; Hypocreales; unidentified; unidentified; Hypocreales_sp
New.ReferenceOTU1047	0	0	0	0	0	0	0	0	0	0	0	0	0	0	0	0	0	0	0	0	0	0	1	0	0	0	0	0	0	1	Fungi; Ascomycota; Sordariomycetes; Hypocreales; unidentified; unidentified; Hypocreales_sp
New.ReferenceOTU1105	0	4	0	0	0	0	0	0	0	0	0	0	0	0	0	0	0	0	0	0	0	0	0	0	0	0	0	0	2	0	Fungi; Ascomycota; Sordariomycetes; Hypocreales; unidentified; unidentified; Hypocreales_sp
New.ReferenceOTU1240	0	0	0	0	0	0	0	0	0	0	1	0	0	0	0	0	0	0	0	0	0	0	0	0	0	0	0	0	0	0	Fungi; Ascomycota; Sordariomycetes; Hypocreales; unidentified; unidentified; Hypocreales_sp
New.ReferenceOTU1276	0	0	0	0	0	0	0	0	0	0	0	0	0	0	0	0	0	0	0	0	0	0	1	0	0	0	0	0	0	0	Fungi; Ascomycota; Sordariomycetes; Hypocreales; unidentified; unidentified; Hypocreales_sp
New.ReferenceOTU1309	0	0	0	0	0	2	0	0	0	0	0	0	0	0	0	0	0	0	0	0	0	0	2	0	0	0	0	0	0	0	Fungi; Ascomycota; Sordariomycetes; Hypocreales; unidentified; unidentified; Hypocreales_sp
New.ReferenceOTU137	0	0	0	0	1	0	0	0	0	0	0	1	0	0	0	0	0	0	0	0	0	0	0	0	22	0	0	1	0	0	Fungi; Ascomycota; Sordariomycetes; Hypocreales; unidentified; unidentified; Hypocreales_sp
New.ReferenceOTU1396	0	0	0	0	0	0	0	0	0	0	0	0	0	0	0	0	0	0	0	0	0	0	0	0	0	1	0	0	1	0	Fungi; Ascomycota; Sordariomycetes; Hypocreales; unidentified; unidentified; Hypocreales_sp
New.ReferenceOTU1657	3	1	0	0	0	0	0	0	0	0	0	0	4	0	0	0	0	0	0	0	0	0	0	0	0	0	0	0	4	0	Fungi; Ascomycota; Sordariomycetes; Hypocreales; unidentified; unidentified; Hypocreales_sp
New.ReferenceOTU1718	1	1	0	0	1	0	0	0	0	0	0	0	0	0	0	0	0	0	0	0	0	0	0	0	0	0	0	0	0	0	Fungi; Ascomycota; Sordariomycetes; Hypocreales; unidentified; unidentified; Hypocreales_sp
New.ReferenceOTU1956	0	0	0	0	0	0	0	0	0	0	1	0	0	0	0	0	0	0	0	0	0	0	0	0	0	0	0	0	0	0	Fungi; Ascomycota; Sordariomycetes; Hypocreales; unidentified; unidentified; Hypocreales_sp
New.ReferenceOTU1963	0	0	0	0	0	0	0	0	0	0	0	0	0	0	0	0	0	0	0	0	0	0	0	0	0	5	0	0	5	0	Fungi; Ascomycota; Sordariomycetes; Hypocreales; unidentified; unidentified; Hypocreales_sp
New.ReferenceOTU1997	1	0	0	0	0	0	0	0	0	0	0	0	0	0	0	0	0	0	0	0	0	0	0	0	0	0	0	0	0	0	Fungi; Ascomycota; Sordariomycetes; Hypocreales; unidentified; unidentified; Hypocreales_sp
New.ReferenceOTU200	0	0	1	0	0	0	0	0	0	0	0	0	0	0	0	0	0	0	0	0	0	0	0	0	0	0	0	0	0	0	Fungi; Ascomycota; Sordariomycetes; Hypocreales; unidentified; unidentified; Hypocreales_sp
New.ReferenceOTU2072	0	0	0	0	0	0	0	0	0	0	0	1	0	0	0	0	0	0	0	0	0	0	0	0	0	0	0	0	0	0	Fungi; Ascomycota; Sordariomycetes; Hypocreales; unidentified; unidentified; Hypocreales_sp
New.ReferenceOTU2103	0	0	12	0	0	0	0	0	0	0	0	0	0	0	0	0	0	0	0	0	0	0	0	0	0	0	0	0	0	0	Fungi; Ascomycota; Sordariomycetes; Hypocreales; unidentified; unidentified; Hypocreales_sp
New.ReferenceOTU2375	5	0	0	0	0	0	0	0	0	0	0	0	0	0	0	0	0	0	0	0	0	0	0	0	0	0	0	0	0	0	Fungi; Ascomycota; Sordariomycetes; Hypocreales; unidentified; unidentified; Hypocreales_sp
New.ReferenceOTU2414	0	0	0	0	0	0	0	0	0	0	9	0	0	0	0	0	0	0	0	0	0	0	0	0	0	1	0	0	5	0	Fungi; Ascomycota; Sordariomycetes; Hypocreales; unidentified; unidentified; Hypocreales_sp
New.ReferenceOTU2609	0	0	0	0	0	0	0	0	0	0	0	0	0	0	0	0	0	1	0	0	0	0	0	0	0	0	0	0	0	0	Fungi; Ascomycota; Sordariomycetes; Hypocreales; unidentified; unidentified; Hypocreales_sp
New.ReferenceOTU2718	0	0	0	0	1	0	0	0	0	0	0	0	0	0	0	0	0	0	0	0	0	0	0	0	0	0	0	0	0	0	Fungi; Ascomycota; Sordariomycetes; Hypocreales; unidentified; unidentified; Hypocreales_sp
New.ReferenceOTU2749	0	0	0	0	0	0	0	0	0	0	0	0	0	0	0	0	0	0	0	1	0	0	0	0	0	0	0	0	0	0	Fungi; Ascomycota; Sordariomycetes; Hypocreales; unidentified; unidentified; Hypocreales_sp
New.ReferenceOTU2850	0	3	1	0	1	0	0	0	2	0	0	0	0	0	0	0	1	0	10	0	0	0	0	0	0	1	0	0	0	0	Fungi; Ascomycota; Sordariomycetes; Hypocreales; unidentified; unidentified; Hypocreales_sp
New.ReferenceOTU3004	0	0	0	0	0	0	1	0	3	0	3	2	0	0	1	0	0	0	6	0	0	1	0	0	0	0	0	0	0	0	Fungi; Ascomycota; Sordariomycetes; Hypocreales; unidentified; unidentified; Hypocreales_sp
New.ReferenceOTU3073	0	0	0	0	0	0	0	0	0	0	0	0	0	0	0	0	0	16	0	0	0	0	0	0	0	0	0	0	0	0	Fungi; Ascomycota; Sordariomycetes; Hypocreales; unidentified; unidentified; Hypocreales_sp
New.ReferenceOTU3085	0	0	0	0	0	0	0	0	0	0	0	1	0	0	0	0	0	0	0	0	0	0	0	0	0	0	0	0	0	0	Fungi; Ascomycota; Sordariomycetes; Hypocreales; unidentified; unidentified; Hypocreales_sp
New.ReferenceOTU3089	0	0	0	0	0	0	0	0	0	0	0	0	0	0	0	0	0	0	0	0	0	0	0	0	0	0	0	0	8	0	Fungi; Ascomycota; Sordariomycetes; Hypocreales; unidentified; unidentified; Hypocreales_sp
New.ReferenceOTU3107	0	0	3	0	0	0	0	0	1	0	0	0	0	0	0	0	0	0	0	0	0	0	0	5	0	0	0	0	0	0	Fungi; Ascomycota; Sordariomycetes; Hypocreales; unidentified; unidentified; Hypocreales_sp
New.ReferenceOTU3142	0	0	0	0	0	0	0	0	0	0	0	0	0	0	0	0	0	0	0	0	0	0	0	0	1	0	0	0	0	0	Fungi; Ascomycota; Sordariomycetes; Hypocreales; unidentified; unidentified; Hypocreales_sp
New.ReferenceOTU3152	0	0	0	0	0	0	0	1	0	0	0	0	0	0	0	1	0	0	0	0	0	1	0	0	0	0	0	0	0	0	Fungi; Ascomycota; Sordariomycetes; Hypocreales; unidentified; unidentified; Hypocreales_sp
New.ReferenceOTU3184	1	0	0	0	0	0	0	0	0	0	0	0	0	0	0	0	0	0	0	0	0	0	0	0	0	0	0	0	0	0	Fungi; Ascomycota; Sordariomycetes; Hypocreales; unidentified; unidentified; Hypocreales_sp
New.ReferenceOTU3255	0	0	0	0	0	0	0	0	0	0	0	0	0	0	0	0	0	0	0	0	0	0	0	0	0	0	0	0	5	0	Fungi; Ascomycota; Sordariomycetes; Hypocreales; unidentified; unidentified; Hypocreales_sp
New.ReferenceOTU3269	0	0	3	0	0	0	0	0	0	0	1	0	0	1	0	0	0	0	0	0	0	0	0	0	0	0	0	0	0	0	Fungi; Ascomycota; Sordariomycetes; Hypocreales; unidentified; unidentified; Hypocreales_sp
New.ReferenceOTU3292	0	0	0	0	2	0	0	0	0	0	0	0	0	0	0	0	0	0	0	0	0	0	0	0	0	0	0	0	0	3	Fungi; Ascomycota; Sordariomycetes; Hypocreales; unidentified; unidentified; Hypocreales_sp
New.ReferenceOTU3481	0	0	0	0	0	0	0	0	1	0	0	0	0	0	0	0	0	0	0	0	0	0	0	0	0	2	0	0	0	0	Fungi; Ascomycota; Sordariomycetes; Hypocreales; unidentified; unidentified; Hypocreales_sp
New.ReferenceOTU3585	1	0	0	0	0	0	0	0	0	0	0	0	0	0	0	0	0	0	0	0	0	0	0	0	0	0	0	0	0	0	Fungi; Ascomycota; Sordariomycetes; Hypocreales; unidentified; unidentified; Hypocreales_sp
New.ReferenceOTU3893	0	0	0	1	0	0	0	0	0	0	0	0	0	0	0	0	0	0	0	0	0	0	0	0	0	0	0	0	0	0	Fungi; Ascomycota; Sordariomycetes; Hypocreales; unidentified; unidentified; Hypocreales_sp
New.ReferenceOTU403	4	0	0	1	0	0	0	0	14	0	0	0	0	0	0	0	0	1	15	0	0	0	0	0	0	0	0	0	1	0	Fungi; Ascomycota; Sordariomycetes; Hypocreales; unidentified; unidentified; Hypocreales_sp
New.ReferenceOTU4034	0	0	0	0	0	0	0	0	0	0	1	0	0	0	0	0	0	0	0	0	0	0	0	0	0	0	0	0	0	0	Fungi; Ascomycota; Sordariomycetes; Hypocreales; unidentified; unidentified; Hypocreales_sp
New.ReferenceOTU4100	0	0	0	0	0	0	0	0	0	0	0	0	0	0	0	0	0	0	1	0	0	0	0	0	0	0	0	0	0	0	Fungi; Ascomycota; Sordariomycetes; Hypocreales; unidentified; unidentified; Hypocreales_sp
New.ReferenceOTU4104	0	0	0	0	5	0	0	0	0	0	0	0	0	0	0	0	0	0	0	0	0	0	0	0	0	0	0	0	0	0	Fungi; Ascomycota; Sordariomycetes; Hypocreales; unidentified; unidentified; Hypocreales_sp
New.ReferenceOTU4131	0	0	0	0	0	0	0	0	0	0	0	0	0	0	0	0	0	0	0	0	0	0	0	0	0	10	0	0	0	0	Fungi; Ascomycota; Sordariomycetes; Hypocreales; unidentified; unidentified; Hypocreales_sp
New.ReferenceOTU4239	0	0	0	0	0	0	0	0	0	0	0	0	0	0	0	0	0	0	1	0	0	0	0	0	0	0	0	0	0	0	Fungi; Ascomycota; Sordariomycetes; Hypocreales; unidentified; unidentified; Hypocreales_sp
New.ReferenceOTU4241	2	29	0	0	1	0	0	0	0	0	0	0	0	2	0	0	0	92	0	0	4	0	0	28	0	0	0	0	15	0	Fungi; Ascomycota; Sordariomycetes; Hypocreales; unidentified; unidentified; Hypocreales_sp
New.ReferenceOTU4602	0	0	0	0	0	0	0	0	0	0	0	0	0	0	0	0	0	0	2	0	0	0	0	0	2	0	0	0	0	0	Fungi; Ascomycota; Sordariomycetes; Hypocreales; unidentified; unidentified; Hypocreales_sp
New.ReferenceOTU4762	0	0	0	0	0	0	0	0	0	0	0	0	0	0	0	0	0	3	0	0	0	0	0	0	0	0	0	0	0	0	Fungi; Ascomycota; Sordariomycetes; Hypocreales; unidentified; unidentified; Hypocreales_sp
New.ReferenceOTU477	0	0	0	0	0	0	0	0	0	0	0	0	0	0	0	0	0	0	0	0	0	0	0	0	1	0	0	0	0	0	Fungi; Ascomycota; Sordariomycetes; Hypocreales; unidentified; unidentified; Hypocreales_sp
New.ReferenceOTU4818	0	0	0	0	0	0	0	0	0	0	0	0	0	0	0	0	0	0	0	0	0	0	0	0	0	0	0	0	2	0	Fungi; Ascomycota; Sordariomycetes; Hypocreales; unidentified; unidentified; Hypocreales_sp
New.ReferenceOTU4911	0	0	0	0	0	0	0	0	0	0	0	0	0	0	0	0	0	0	0	0	0	0	0	1	0	0	3	0	0	0	Fungi; Ascomycota; Sordariomycetes; Hypocreales; unidentified; unidentified; Hypocreales_sp
New.ReferenceOTU5000	0	0	0	0	0	0	0	0	0	0	0	0	0	0	0	0	0	0	0	0	0	0	0	0	0	8	0	0	0	0	Fungi; Ascomycota; Sordariomycetes; Hypocreales; unidentified; unidentified; Hypocreales_sp
New.ReferenceOTU5023	0	0	0	0	0	0	0	0	0	0	0	0	0	0	0	0	0	0	0	0	0	0	0	0	0	0	0	0	1	0	Fungi; Ascomycota; Sordariomycetes; Hypocreales; unidentified; unidentified; Hypocreales_sp

New.ReferenceOTU5042	0	0	0	0	0	0	0	0	0	0	0	1	0	0	0	0	0	0	0	0	0	0	0	0	0	0	0	0	0	0	Fungi; Ascomycota; Sordariomycetes; Hypocreales; unidentified; unidentified; Hypocreales_sp
New.ReferenceOTU5126	0	0	0	0	0	0	0	0	0	0	0	0	1	0	0	0	0	0	0	0	0	0	0	0	0	0	0	0	0	0	Fungi; Ascomycota; Sordariomycetes; Hypocreales; unidentified; unidentified; Hypocreales_sp
New.ReferenceOTU5165	0	0	0	0	0	0	0	0	0	0	0	0	0	0	0	0	0	0	0	0	0	0	0	0	0	7	0	0	0	0	Fungi; Ascomycota; Sordariomycetes; Hypocreales; unidentified; unidentified; Hypocreales_sp
New.ReferenceOTU5192	0	0	0	0	0	0	0	0	0	0	1	0	0	0	0	0	0	0	2	0	0	0	0	0	0	0	0	0	0	0	Fungi; Ascomycota; Sordariomycetes; Hypocreales; unidentified; unidentified; Hypocreales_sp
New.ReferenceOTU5289	1	0	0	0	0	0	0	0	0	0	0	0	0	0	0	2	0	5	0	0	0	0	1	1	0	0	0	0	0	0	Fungi; Ascomycota; Sordariomycetes; Hypocreales; unidentified; unidentified; Hypocreales_sp
New.ReferenceOTU5329	0	0	0	0	0	0	0	0	0	0	0	0	0	0	2	0	0	0	0	0	0	0	0	0	0	0	0	0	0	0	Fungi; Ascomycota; Sordariomycetes; Hypocreales; unidentified; unidentified; Hypocreales_sp
New.ReferenceOTU5332	0	4	0	0	0	0	0	0	0	0	0	1	0	0	0	0	0	0	0	1	0	0	0	0	0	0	0	0	0	0	Fungi; Ascomycota; Sordariomycetes; Hypocreales; unidentified; unidentified; Hypocreales_sp
New.ReferenceOTU5377	1	2	0	0	0	0	0	0	0	0	0	0	0	0	0	0	0	0	6	0	0	0	0	4	0	0	0	0	0	0	Fungi; Ascomycota; Sordariomycetes; Hypocreales; unidentified; unidentified; Hypocreales_sp
New.ReferenceOTU5406	0	0	0	0	0	0	0	0	0	0	0	0	0	0	0	0	0	1	0	0	0	0	0	0	0	0	0	0	0	0	Fungi; Ascomycota; Sordariomycetes; Hypocreales; unidentified; unidentified; Hypocreales_sp
New.ReferenceOTU5547	0	0	0	0	0	0	0	0	0	0	0	0	0	0	0	0	0	0	0	0	0	0	0	0	0	0	0	0	0	19	Fungi; Ascomycota; Sordariomycetes; Hypocreales; unidentified; unidentified; Hypocreales_sp
New.ReferenceOTU5599	0	0	0	0	0	0	0	0	0	0	0	0	0	0	0	0	0	0	0	0	0	0	0	0	0	0	0	0	4	0	Fungi; Ascomycota; Sordariomycetes; Hypocreales; unidentified; unidentified; Hypocreales_sp
New.ReferenceOTU5631	0	0	0	0	0	0	0	0	0	0	1	0	0	0	0	0	0	0	0	0	0	0	1	0	0	0	0	0	0	0	Fungi; Ascomycota; Sordariomycetes; Hypocreales; unidentified; unidentified; Hypocreales_sp
New.ReferenceOTU5651	2	0	0	0	3	1	0	0	0	0	2	0	0	2	0	0	0	0	0	0	1	0	0	0	3	0	1	1	0	0	Fungi; Ascomycota; Sordariomycetes; Hypocreales; unidentified; unidentified; Hypocreales_sp
New.ReferenceOTU5658	0	0	0	0	0	0	0	0	2	0	0	0	0	0	0	0	0	0	0	0	0	0	0	0	0	0	0	0	0	0	Fungi; Ascomycota; Sordariomycetes; Hypocreales; unidentified; unidentified; Hypocreales_sp
New.ReferenceOTU5672	0	0	0	0	0	0	0	0	0	0	0	0	0	0	0	0	0	0	0	0	1	0	0	0	0	0	0	0	0	0	Fungi; Ascomycota; Sordariomycetes; Hypocreales; unidentified; unidentified; Hypocreales_sp
New.ReferenceOTU5787	0	0	11	0	0	0	0	0	0	0	0	0	0	0	0	0	0	0	0	0	0	0	0	0	0	0	0	0	0	0	Fungi; Ascomycota; Sordariomycetes; Hypocreales; unidentified; unidentified; Hypocreales_sp
New.ReferenceOTU6141	0	1	0	0	0	0	0	0	0	0	0	0	0	0	0	0	0	0	0	0	0	0	0	0	0	0	0	0	0	0	Fungi; Ascomycota; Sordariomycetes; Hypocreales; unidentified; unidentified; Hypocreales_sp
New.ReferenceOTU6147	0	0	0	0	0	0	0	0	0	0	0	0	0	0	0	0	0	0	0	0	0	0	0	0	0	0	0	0	1	0	Fungi; Ascomycota; Sordariomycetes; Hypocreales; unidentified; unidentified; Hypocreales_sp
New.ReferenceOTU6226	0	0	0	0	0	0	1	0	0	0	0	0	0	0	0	0	0	0	0	0	0	0	0	0	0	0	0	0	0	0	Fungi; Ascomycota; Sordariomycetes; Hypocreales; unidentified; unidentified; Hypocreales_sp
New.ReferenceOTU6398	0	0	0	0	0	0	0	0	0	0	0	0	0	0	0	0	0	0	0	0	0	0	0	0	0	35	0	0	0	0	Fungi; Ascomycota; Sordariomycetes; Hypocreales; unidentified; unidentified; Hypocreales_sp
New.ReferenceOTU6421	0	0	0	0	1	0	0	0	1	0	0	0	0	0	0	0	0	0	0	0	0	0	2	0	0	0	0	0	0	0	Fungi; Ascomycota; Sordariomycetes; Hypocreales; unidentified; unidentified; Hypocreales_sp
New.ReferenceOTU6485	0	0	0	0	0	0	0	0	0	0	0	0	0	0	0	0	0	0	1	0	0	0	0	0	0	0	0	0	0	0	Fungi; Ascomycota; Sordariomycetes; Hypocreales; unidentified; unidentified; Hypocreales_sp
New.ReferenceOTU6637	0	0	0	0	0	0	0	0	0	0	0	0	0	0	0	0	0	0	0	0	0	0	0	0	0	0	0	0	0	2	Fungi; Ascomycota; Sordariomycetes; Hypocreales; unidentified; unidentified; Hypocreales_sp
New.ReferenceOTU6816	0	3	0	0	0	0	0	0	0	0	0	0	0	0	0	0	0	0	0	0	0	0	0	0	0	0	0	0	2	0	Fungi; Ascomycota; Sordariomycetes; Hypocreales; unidentified; unidentified; Hypocreales_sp
New.ReferenceOTU6937	0	1	0	0	0	0	0	0	0	0	0	0	0	0	0	0	0	0	0	0	0	0	0	0	0	0	0	0	0	0	Fungi; Ascomycota; Sordariomycetes; Hypocreales; unidentified; unidentified; Hypocreales_sp
New.ReferenceOTU7020	0	0	0	1	0	0	0	0	0	0	0	0	0	0	0	0	0	0	0	0	0	0	0	0	0	0	0	0	0	0	Fungi; Ascomycota; Sordariomycetes; Hypocreales; unidentified; unidentified; Hypocreales_sp
New.ReferenceOTU7051	0	0	0	0	0	0	0	0	0	0	0	0	0	0	0	0	0	0	0	0	0	1	0	0	0	0	0	0	0	0	Fungi; Ascomycota; Sordariomycetes; Hypocreales; unidentified; unidentified; Hypocreales_sp
New.ReferenceOTU7066	0	0	0	0	0	0	0	0	0	0	1	0	0	0	0	0	0	0	1	0	0	0	0	0	0	0	0	0	0	0	Fungi; Ascomycota; Sordariomycetes; Hypocreales; unidentified; unidentified; Hypocreales_sp
New.ReferenceOTU7117	3	27	0	0	0	0	0	0	0	0	0	0	3	0	0	0	0	0	0	0	0	0	0	0	0	4	0	0	8	0	Fungi; Ascomycota; Sordariomycetes; Hypocreales; unidentified; unidentified; Hypocreales_sp
New.ReferenceOTU712	0	0	0	0	0	0	0	0	1	1	0	3	0	1	1	0	0	0	0	1	0	0	1	0	1	0	0	1	0	2	Fungi; Ascomycota; Sordariomycetes; Hypocreales; unidentified; unidentified; Hypocreales_sp
New.ReferenceOTU7289	0	0	0	1	0	0	0	0	0	0	0	0	0	0	0	0	0	0	0	0	0	0	0	0	0	0	0	0	0	0	Fungi; Ascomycota; Sordariomycetes; Hypocreales; unidentified; unidentified; Hypocreales_sp
New.ReferenceOTU7357	0	0	0	0	0	0	0	1	0	0	0	0	0	0	0	0	0	1	0	0	0	0	0	0	0	0	0	0	0	0	Fungi; Ascomycota; Sordariomycetes; Hypocreales; unidentified; unidentified; Hypocreales_sp
New.ReferenceOTU743	0	0	0	0	0	0	0	0	4	0	0	0	0	0	0	0	0	0	0	0	0	0	0	0	0	0	0	0	0	0	Fungi; Ascomycota; Sordariomycetes; Hypocreales; unidentified; unidentified; Hypocreales_sp
New.ReferenceOTU7506	0	0	0	0	0	0	0	0	0	0	0	0	0	0	0	0	0	0	0	0	0	0	2	0	0	0	0	0	0	0	Fungi; Ascomycota; Sordariomycetes; Hypocreales; unidentified; unidentified; Hypocreales_sp
New.ReferenceOTU756	0	0	0	0	0	0	0	0	0	0	0	0	0	1	0	0	0	0	0	0	0	0	0	0	0	0	0	0	0	0	Fungi; Ascomycota; Sordariomycetes; Hypocreales; unidentified; unidentified; Hypocreales_sp
New.ReferenceOTU758	0	0	0	0	0	0	0	0	0	0	0	0	0	0	1	0	0	0	0	0	0	0	0	0	0	0	0	0	0	0	Fungi; Ascomycota; Sordariomycetes; Hypocreales; unidentified; unidentified; Hypocreales_sp
New.ReferenceOTU7698	0	0	0	0	0	0	0	0	1	0	0	0	0	0	0	0	0	1	0	0	0	0	0	0	0	0	0	0	0	0	Fungi; Ascomycota; Sordariomycetes; Hypocreales; unidentified; unidentified; Hypocreales_sp
New.ReferenceOTU7735	0	0	0	0	0	0	0	0	0	1	0	0	0	0	0	0	0	0	0	0	0	1	0	0	0	0	0	0	0	0	Fungi; Ascomycota; Sordariomycetes; Hypocreales; unidentified; unidentified; Hypocreales_sp
New.ReferenceOTU7795	0	0	0	0	1	0	0	0	0	0	0	0	0	0	0	0	0	0	0	0	0	0	0	0	0	0	0	0	0	0	Fungi; Ascomycota; Sordariomycetes; Hypocreales; unidentified; unidentified; Hypocreales_sp
New.ReferenceOTU7914	0	0	0	0	0	0	0	0	0	0	0	0	0	0	2	0	0	0	0	0	0	0	0	0	0	0	0	0	0	0	Fungi; Ascomycota; Sordariomycetes; Hypocreales; unidentified; unidentified; Hypocreales_sp
New.ReferenceOTU794	0	0	0	0	0	0	0	0	0	0	0	0	0	0	0	0	0	0	0	0	0	0	0	0	0	0	1	0	0	0	Fungi; Ascomycota; Sordariomycetes; Hypocreales; unidentified; unidentified; Hypocreales_sp
New.ReferenceOTU922	0	0	0	0	0	1	0	0	0	0	0	0	0	0	0	0	0	0	0	0	0	0	0	0	0	0	0	0	0	0	Fungi; Ascomycota; Sordariomycetes; Hypocreales; unidentified; unidentified; Hypocreales_sp
New.ReferenceOTU933	0	0	0	0	0	0	0	0	0	0	0	0	0	0	0	0	0	0	0	0	0	0	0	0	0	0	1	0	0	0	Fungi; Ascomycota; Sordariomycetes; Hypocreales; unidentified; unidentified; Hypocreales_sp
SH021352.06FU_EU687056_reps_singleton	0	0	0	0	0	0	1	0	0	0	0	2	0	0	0	0	1	0	0	0	0	0	0	0	0	0	0	0	0	0	Fungi; Ascomycota; Sordariomycetes; Hypocreales; unidentified; unidentified; Hypocreales_sp
SH021437.06FU_EU002935_reps_singleton	0	0	0	0	0	0	0	0	0	0	0	0	0	1	0	0	0	0	0	0	0	0	0	0	0	0	0	0	0	0	Fungi; Ascomycota; Sordariomycetes; Hypocreales; unidentified; unidentified; Hypocreales_sp
SH032486.06FU_JQ081535_reps_singleton	0	0	1	0	0	0	0	0	0	1	0	0	1	1	0	0	0	0	0	0	3	0	0	0	0	0	0	0	0	0	Fungi; Ascomycota; Sordariomycetes; Hypocreales; unidentified; unidentified; Hypocreales_sp
SH093577.06FU_DQ682567_reps	0	0	0	0	0	0	0	0	0	0	0	0	0	0	0	0	0	0	0	0	1	0	0	0	1	0	0	0	0	0	Fungi; Ascomycota; Sordariomycetes; Hypocreales; unidentified; unidentified; Hypocreales_sp
SH095046.06FU_HQ607821_reps	0	0	0	1	0	0	0	0	0	0	0	0	0	0	0	0	0	0	0	0	0	0	0	0	0	0	0	0	0	0	Fungi; Ascomycota; Sordariomycetes; Hypocreales; unidentified; unidentified; Hypocreales_sp
SH095128.06FU_AY268191_reps	2	0	0	0	4	3	0	1	0	0	0	0	0	0	0	2	0	4	0	0	0	0	1	0	1	0	0	0	1	0	Fungi; Ascomycota; Sordariomycetes; Hypocreales; unidentified; unidentified; Hypocreales_sp
SH095130.06FU_JX033590_reps	1	0	0	0	4	0	0	0	2	2	0	1	0	0	0	0	0	1	0	0	0	0	0	0	0	0	0	2	0	0	Fungi; Ascomycota; Sordariomycetes; Hypocreales; unidentified; unidentified; Hypocreales_sp
SH110561.06FU_HQ021890_reps	3	1	0	0	1	0	0	0	5	0	0	0	0	0	0	1	0	1	3	0	0	0	0	0	0	9	0	0	0	0	Fungi; Ascomycota; Sordariomycetes; Hypocreales; unidentified; unidentified; Hypocreales_sp
SH118600.06FU_AY381139_reps	0	0	0	1	0	0	0	0	0	1	0	0	0	0	0	1	0	0	0	0	0	0	0	0	0	0	0	0	0	0	Fungi; Ascomycota; Sordariomycetes; Hypocreales; unidentified; unidentified; Hypocreales_sp
SH118603.06FU_FJ613000_reps	0	0	0	0	0	0	0	0	0	0	0	0	0	0	0	0	0	0	0	0	1	0	0	0	0	0	0	1	0	0	Fungi; Ascomycota; Sordariomycetes; Hypocreales; unidentified; unidentified; Hypocreales_sp
SH118608.06FU_HQ608142_reps	0	0	0	0	0	0	0	0	0	0	1	0	0	1	10	0	0	0	0	0	0	0	0	0	3	0	1	0	0	0	Fungi; Ascomycota; Sordariomycetes; Hypocreales; unidentified; unidentified; Hypocreales_sp
SH121370.06FU_EU372830_reps	0	0	0	0	0	0	0	0	0	0	0	0	0	0	0	0	0	0	0	0	0	0	0	0	1	0	0	0	0	0	Fungi; Ascomycota; Sordariomycetes; Hypocreales; unidentified; unidentified; Hypocreales_sp
SH124355.06FU_JQ717359_reps	0	0	0	0	0	0	0	0	0	0	0	0	0	0	0	0	0	0	0	0	0	0	1	0	1	0	0	0	0	0	Fungi; Ascomycota; Sordariomycetes; Hypocreales; unidentified; unidentified; Hypocreales_sp
SH125857.06FU_JX244032_reps	0	0	0	0	0	0	0	0	0	0	0	0	0	0	0	0	0	1	0	0	0	0	0	0	1	0	0	0	0	0	Fungi; Ascomycota; Sordariomycetes; Hypocreales; unidentified; unidentified; Hypocreales_sp
SH125861.06FU_HQ248209_reps	0	0	0	0	0	0	0	0	0	0	0	0	0	0	1	0	0	0	0	0	0	0	0	0	0	0	0	0	0	0	Fungi; Ascomycota; Sordariomycetes; Hypocreales; unidentified; unidentified; Hypocreales_sp
SH126835.06FU_FN706554_reps	0	0	0	0	0	0	0	0	0	0	0	0	0	0	0	0	0	0	0	0	0	0	0	0	0	0	2	0	0	5	Fungi; Ascomycota; Sordariomycetes; Hypocreales; unidentified; unidentified; Hypocreales_sp
SH126840.06FU_FR668017_reps	0	0	0	0	0	0	0	0	1	0	0	0	0	0	0	0	0	0	0	0	0	0	1	0	0	0	0	0	0	0	Fungi; Ascomycota; Sordariomycetes; Hypocreales; unidentified; unidentified; Hypocreales_sp
SH127930.06FU_KC007311_reps	0	0	0	0	0	0	0	0	0	0	2	0	0	0	0	0	0	0	0	0	0	0	0	0	0	0	0	0	0	0	Fungi; Ascomycota; Sordariomycetes; Hypocreales; unidentified; unidentified; Hypocreales_sp
SH130581.06FU_EU139248_reps	0	0	0	0	1	0	0	0	1	0	14	1	0	0	0	0	0	0	1	0	0	0	0	0	0	0	0	0	0	0	Fungi; Ascomycota; Sordariomycetes; Hypocreales; unidentified; unidentified; Hypocreales_sp
SH140223.06FU_DQ421127_reps	3	0	1	2	2	6	9	7	15	0	8	34	2	1	2	0	0	1	10	2	9	18	1	0	2	0	9	2	0	3	Fungi; Ascomycota; Sordariomycetes; Hypocreales; unidentified; unidentified; Hypocreales_sp
SH141469.06FU_JQ936269_reps	0	0	0	0	0	0	0	0	0	0	0	0	0	0	5	0	0	0	0	0	0	0	0	0	0	0	0	0	0	0	Fungi; Ascomycota; Sordariomycetes; Hypocreales; unidentified; unidentified; Hypocreales_sp
SH142707.06FU_JQ905674_reps	0	0	3	7	0	0	0	0	0	0	3	1	0	0	0	2	0	1	0	0	0	1	0	4	0	1	0	0	0	0	Fungi; Ascomycota; Sordariomycetes; Hypocreales; unidentified; unidentified; Hypocreales_sp
New.ReferenceOTU4224	0	0	0	0	0	0	0	0	0	0	0	2	0	0	0	2	2	0	0	0	0	0	0	0	1	0	0	0	0	0	Fungi; Ascomycota; Sordariomycetes; Hypocreales; unidentified; unidentified; Hypocreales_sp_2_MJ17
New.ReferenceOTU5169	0	0	0	2	0	0	0	0	0	0	0	0	0	1	0	0	0	0	0	0	0	0	0	0	0	1	0	0	0	0	Fungi; Ascomycota; Sordariomycetes; Hypocreales; unidentified; unidentified; Hypocreales_sp_BBP_2010
New.ReferenceOTU6400	0	0	0	0	0	0	0	0	0	0	0	0	0	0	1	0	0	0	0	0	0	0	0	0	0	0	0	0	0	0	Fungi; Ascomycota; Sordariomycetes; Hypocreales; unidentified; unidentified; Hypocreales_sp_BBP_2010
SH027283.06FU_HQ437657_reps_singleton	0	0	0	0	0	0	0	1	0	0	0	2	0	3	0	0	0	0	0	0	0	0	0	0	0	0	0	0	0	0	Fungi; Ascomycota; Sordariomycetes; Hypocreales; unidentified; unidentified; Hypocreales_sp_BBP_2010
New.ReferenceOTU6019	0	0	0	0	0	0	0	0	0	0	0	0	0	0	0	0	1	0	0	0	0	0	0	0	0	0	0	0	0	0	Fungi; Ascomycota; Sordariomycetes; Hypocreales; unidentified; unidentified; Hypocreales_sp_GMG_C1
New.ReferenceOTU4863	0	0	0	2	0	0	0	0	0	0	0	0	0	0	0	0	1	0	0	0	0	0	0	0	0	0	0	0	0	0	Fungi; Ascomycota; Sordariomycetes; Hypocreales; unidentified; unidentified; Hypocreales_sp_KLAR_B5
SH024811.06FU_EU164804_reps_singleton	0	0	0	0	0	0	0	0	0	0	0	0	0	0	0	0	1	0	0	0	0	0	0	0	0	0	0	0	0	0	Fungi; Ascomycota; Sordariomycetes; Hypocreales; unidentified; unidentified; Hypocreales_sp_KLAR_B5
New.ReferenceOTU2792	4	0	0	0	1	0	0	0	0	0	0	1	0	0	0	0	0	0	0	0	0	0	0	0	0	0	0	0	0	0	Fungi; Ascomycota; Sordariomycetes; Hypocreales; unidentified; unidentified; Hypocreales_sp_MS325
New.ReferenceOTU1522	0	0	0	0	1	0	0	0	0	0	0	0	0	0	0	0	0	0	0	0	0	0	0	0	0	0	0	0	0	0	Fungi; Ascomycota; Sordariomycetes; Hypocreales; unidentified; unidentified; Hypocreales_sp_TR173
New.ReferenceOTU2573	0	0	0	0	0	0	0	0	0	0	0	0	0	0	0	1	1	0	0	0	0	0	0	3	0	0	0	0	0	0	Fungi; Ascomycota; Sordariomycetes; Hypocreales; unidentified; unidentified; uncultured_Hypocreales
New.ReferenceOTU3	0	0	0	0	0	0	0	0	0	1	0	0	0	0	0	0	0	0	0	0	0	0	0	0	0	0	0	0	0	0	Fungi; Ascomycota; Sordariomycetes; Hypocreales; unidentified; unidentified; uncultured_Hypocreales
New.ReferenceOTU3013	0	0	0	0	1	0	0	0	0	0	0	0	0	0	0	2	0	1	0	0	0	0	0	0	0	0	0	0	0	0	Fungi; Ascomycota; Sordariomycetes; Hypocreales; unidentified; unidentified; uncultured_Hypocreales
New.ReferenceOTU4412	0	0	0	0	0	0	0	0	0	0	0	0	0	0	0	0	0	0	0	0	0	0	0	0	1	0	0	0	0	0	Fungi; Ascomycota; Sordariomycetes; Hypocreales; unidentified; unidentified; uncultured_Hypocreales
New.ReferenceOTU5644	0	0	0	0	0	0	0	0	0	0	0	0	0	0	0	0	0	0	0	0	0	2	0	0	0	0	0	0	0	0	Fungi; Ascomycota; Sordariomycetes; Hypocreales; unidentified; unidentified; uncultured_Hypocreales
New.ReferenceOTU7913	0	0	0	0	0	0	0	0	0	0	0	0	0	1	0	0	0	0	0	0	0	0	0	0	0	0	0	0	0	0	Fungi; Ascomycota; Sordariomycetes; Hypocreales; unidentified; unidentified; uncultured_Hypocreales
New.ReferenceOTU8079	0	0	0	0	0	0	0	0	0	0	0	0	0	0	0	0	0	0	0	0	0	0	0	0	1	0	0	0	0	0	Fungi; Ascomycota; Sordariomycetes; Hypocreales; unidentified; unidentified; uncultured_Hypocreales
SH027531.06FU_FJ553653_reps_singleton	0	0	0	0	0	0	0	0	0	1	0	0	0	2	0	0	0	0	0	0	0	0	0	0	0	0	0	0	0	0	Fungi; Ascomycota; Sordariomycetes; Hypocreales; unidentified; unidentified; uncultured_Hypocreales
New.ReferenceOTU5402	0	0	6	0	0	2	0	0	0	0	0	0	0	0	0	0	0	0	0	0	0	0	0	0	1	0	1	0	0	0	Fungi; Ascomycota; Sordariomycetes; Incertae_sedis; Annulatascaceae; Conlarium; Conlarium_duplumascospora
New.ReferenceOTU7653	0	0	0	0	0	0	0	1	0	0	0	0	0	0	0	0	0	0	0	0	0	0	0	0	0	0	0	0	0	0	Fungi; Ascomycota; Sordariomycetes; Incertae_sedis; Annulatascaceae; Conlarium; Conlarium_duplumascospora
SH128317.06FU_JN936995_reps	0	0	4	0	0	0	0	0	0	0	1	0	1	0	0	0	0	0	0	0	0	0	0	0	3	0	0	0	0	0	Fungi; Ascomycota; Sordariomycetes; Incertae_sedis; Annulatascaceae; Conlarium; Conlarium_duplumascospora
New.ReferenceOTU0	0	0	0	0	0	0	0	0	0	0	0	0	0	0	0	0	0	0	0	0	2	0	0	0	0	0	0	0	0	0	Fungi; Ascomycota; Sordariomycetes; Incertae_sedis; Annulatascaceae; Conlarium; Conlarium_sp
New.ReferenceOTU1361	0	0	0	0	0	0	0	0	1	0	2	0	0	0	0	0	0	0	1	0	0	0	0	0	0	0	0	0	0	0	Fungi; Ascomycota; Sordariomycetes; Incertae_sedis; Annulatascaceae; Conlarium; Conlarium_sp
New.ReferenceOTU4498	0	0	0	0	0	0	0	0	0	0	0	0	0	0	1	0	0	0	0	0	0	0	0	0	0	0	0	0	0	0	Fungi; Ascomycota; Sordariomycetes; Incertae_sedis; Annulatascaceae; Conlarium; Conlarium_sp
New.ReferenceOTU4749	0	0	0	0	0	0	0	0	1	0	0	0	0	0	0	0	0	0	1	0	0	0	0	0	1	0	0	0	0	0	Fungi; Ascomycota; Sordariomycetes; Incertae_sedis; Annulatascaceae; Conlarium; Conlarium_sp
New.ReferenceOTU5229	0	0	0	0	0	0	0	0	0	0	0	0	0	0	0	1	0	0	0	0	0	0	0	0	0	0	0	0	0	0	Fungi; Ascomycota; Sordariomycetes; Incertae_sedis; Annulatascaceae; Conlarium; Conlarium_sp
New.ReferenceOTU6231	0	0	0	0	0	0	0	0	0	0	0	0	0	0	0	0	0	0	0	0	0	0	0	0	0	0	0	0	0	1	Fungi; Ascomycota; Sordariomycetes; Incertae_sedis; Annulatascaceae; Conlarium; Conlarium_sp
New.ReferenceOTU737	0	0	0	0	0	0	0	0	0	1	0	0	0	7	0	4	1	0	0	0	0	0	0	1	0	0	0	0	0	0	Fungi; Ascomycota; Sordariomycetes; Incertae_sedis; Annulatascaceae; Conlarium; Conlarium_sp
New.ReferenceOTU7838	0	0	0	0	0	0	0	0	0	0	0	0	0	0	1	0	0	0	0	0	0	0	0	0	0	0	0	0	0	0	Fungi; Ascomycota; Sordariomycetes; Incertae_sedis; Annulatascaceae; Conlarium; Conlarium_sp
SH025873.06FU_JX489782_reps_singleton	0	0	0	0	0	0	0	0	0	0	0	0	0	0	0	1	0	0	0	0	0	4	0	0	0	0	0	2	0	2	Fungi; Ascomycota; Sordariomycetes; Incertae_sedis; Annulatascaceae; Conlarium; Conlarium_sp
New.ReferenceOTU1218	0	0	0	0	0	0	2	0	0	0	0	0	0	0	0	0	0	0	0	0	0	0	0	0	0	0	0	0	0	0	Fungi; Ascomycota; Sordariomycetes; Incertae_sedis; Annulatascaceae; Vertexicola; Vertexicola_confusa
New.ReferenceOTU3583	4	0	0	0	0	0	1	0	0	0	0	0	0	0	0	0	0	0	0	1	0	0	0	0	0	0	0	0	0	0	Fungi; Ascomycota; Sordariomycetes; Incertae_sedis; Annulatascaceae; Vertexicola; Vertexicola_confusa
New.ReferenceOTU1001	0	0	0	0	0	0	0	0	0	0	0	0	0	0	0	1	0	0	0	0	0	0	0	0	0	0	0	0	0	0	Fungi; Ascomycota; Sordariomycetes; Incertae_sedis; Apiosporaceae; Arthrinium; Arthrinium_urticae
New.ReferenceOTU2624	0	0	0	0	0	0	0	0	1	0	0	0	0	0	0	0	0	0	0	1	0	0	0	0	0	0	0	1	0	0	Fungi; Ascomycota; Sordariomycetes; Incertae_sedis; Apiosporaceae; Arthrinium; Arthrinium_urticae
SH110109.06FU_EU977228_reps	0	0	0	0	0	0	0	0	0	0	4	0	0	0	0	0	0	0	0	0	1	10	1	0	3	0	0	7	0	1	Fungi; Ascomycota; Sordariomycetes; Incertae_sedis; Apiosporaceae; Arthrinium; Arthrinium_urticae
SH110115.06FU_AB220245_reps	0	0	0	0	0	0	0	0	5	1	0	0	0	0	0	0	0	0	0	0	0	0	2	0	0	0	0	0	0	0	Fungi; Ascomycota; Sordariomycetes; Incertae_sedis; Apiosporaceae; Arthrinium; Arthrinium_urticae

New.ReferenceOTU4653	0	0	0	0	0	0	0	0	0	0	0	0	0	0	0	0	0	0	0	0	0	0	1	0	0	0	0	0	0	0	Fungi; Ascomycota; Sordariomycetes; Incertae_sedis; Incertae_sedis; Myrmecridium; Myrmecridium_schulzeri
New.ReferenceOTU1052	0	0	1	0	0	0	0	0	0	0	0	0	0	1	0	0	3	0	0	0	0	0	0	0	0	0	0	0	0	0	Fungi; Ascomycota; Sordariomycetes; Incertae_sedis; Incertae_sedis; Pleurotheciella; Pleurotheciella_centenaria
New.ReferenceOTU1264	0	0	0	0	0	0	0	0	0	1	0	0	0	0	0	0	0	0	0	0	0	0	0	0	0	0	0	0	0	0	Fungi; Ascomycota; Sordariomycetes; Incertae_sedis; Incertae_sedis; Pleurotheciella; Pleurotheciella_centenaria
New.ReferenceOTU7158	0	0	0	0	0	0	0	0	0	0	0	1	0	0	0	0	0	0	0	0	0	0	0	0	0	0	0	0	0	0	Fungi; Ascomycota; Sordariomycetes; Incertae_sedis; Incertae_sedis; Pleurotheciella; Pleurotheciella_centenaria
New.ReferenceOTU2155	0	0	0	0	0	0	0	0	0	0	0	0	0	0	0	0	0	0	0	0	0	0	1	0	0	0	0	1	0	0	Fungi; Ascomycota; Sordariomycetes; Incertae_sedis; Incertae_sedis; Savoryella; Savoryella_verrucosa
New.ReferenceOTU5763	0	0	0	0	0	0	0	0	0	0	0	0	0	0	0	0	0	0	0	0	0	0	0	0	1	0	0	0	0	0	Fungi; Ascomycota; Sordariomycetes; Incertae_sedis; Incertae_sedis; Savoryella; Savoryella_verrucosa
SH143374.06FU_FR837913_reps	0	0	0	0	0	0	0	0	0	0	0	0	0	0	0	0	0	0	0	0	0	1	0	0	0	0	0	0	0	0	Fungi; Ascomycota; Sordariomycetes; Incertae_sedis; Incertae_sedis; Xylomelasma; Xylomelasma_sp_ZK29/08
New.ReferenceOTU1545	0	0	0	0	0	0	0	0	0	0	0	0	0	0	0	0	0	0	0	0	1	0	0	0	0	0	0	0	0	0	Fungi; Ascomycota; Sordariomycetes; Incertae_sedis; Magnaporthaceae; Pseudohalonectria; Pseudohalonectria_lignicola
New.ReferenceOTU4842	0	0	0	0	0	0	0	0	0	0	1	0	0	0	0	0	0	0	0	0	0	0	0	0	0	0	0	0	0	0	Fungi; Ascomycota; Sordariomycetes; Incertae_sedis; Magnaporthaceae; Pseudohalonectria; Pseudohalonectria_lignicola
New.ReferenceOTU2303	0	0	0	0	0	0	0	0	0	0	0	0	0	0	0	1	0	0	1	0	0	0	0	0	0	0	0	0	0	0	Fungi; Ascomycota; Sordariomycetes; Microascales; Microascaceae; Graphium; Graphium_carbonarium
New.ReferenceOTU4702	0	0	0	0	0	0	0	0	0	0	0	0	0	0	2	0	0	0	0	0	0	6	0	0	2	0	0	0	0	0	Fungi; Ascomycota; Sordariomycetes; Microascales; Microascaceae; Graphium; Graphium_penicillioides
New.ReferenceOTU5952	0	0	0	0	0	0	0	0	4	0	0	0	0	0	0	0	0	0	0	0	0	0	0	0	0	0	0	0	0	0	Fungi; Ascomycota; Sordariomycetes; Microascales; Microascaceae; Graphium; Graphium_sp_3PG8P1_G2
New.ReferenceOTU5778	0	0	0	0	0	0	0	0	0	3	0	0	0	0	0	0	0	0	0	0	0	0	0	0	0	0	0	0	0	0	Fungi; Ascomycota; Sordariomycetes; Microascales; Microascaceae; Parascedosporium; Parascedosporium_putredinis
SH133819.06FU_HQ185348_refs	0	0	0	0	0	0	0	0	0	6	0	0	0	0	0	0	0	0	0	0	0	0	0	0	0	0	0	0	0	0	Fungi; Ascomycota; Sordariomycetes; Microascales; Microascaceae; Parascedosporium; Parascedosporium_putredinis
New.ReferenceOTU1820	0	0	0	0	0	0	0	0	0	0	0	0	0	0	0	0	0	0	8	0	0	0	0	0	0	0	0	0	0	0	Fungi; Ascomycota; Sordariomycetes; Microascales; Microascaceae; Petriella; Petriella_sordida
New.ReferenceOTU1284	0	0	0	0	0	0	0	0	1	3	0	0	0	0	0	0	0	1	0	0	0	0	0	0	0	0	0	0	0	0	Fungi; Ascomycota; Sordariomycetes; Microascales; Microascaceae; Pseudallescheria; Pseudallescheria_angusta
New.ReferenceOTU1787	0	0	0	0	0	0	0	0	0	0	0	1	0	0	0	0	0	0	0	0	0	0	0	0	0	0	0	0	0	0	Fungi; Ascomycota; Sordariomycetes; Microascales; Microascaceae; Pseudallescheria; Pseudallescheria_angusta
New.ReferenceOTU3484	1	0	0	2	0	3	2	0	1	3	0	3	1	0	1	1	0	2	3	0	1	4	2	1	0	0	0	0	0	3	Fungi; Ascomycota; Sordariomycetes; Microascales; Microascaceae; Pseudallescheria; Pseudallescheria_angusta
SH014564.06FU_JQ409645_reps_singleton	0	0	0	0	0	2	0	0	0	0	0	0	0	0	0	0	0	0	0	0	0	1	0	0	0	0	0	0	0	0	Fungi; Ascomycota; Sordariomycetes; Microascales; Microascaceae; Pseudallescheria; Pseudallescheria_angusta
SH014566.06FU_FN813733_reps_singleton	6	2	1	4	0	2	2	0	1	0	0	0	0	0	0	0	1	0	2	1	1	1	3	1	0	0	1	0	0	2	Fungi; Ascomycota; Sordariomycetes; Microascales; Microascaceae; Pseudallescheria; Pseudallescheria_angusta
SH109824.06FU_AJ888414_refs	0	0	0	2	0	0	0	0	2	2	0	3	0	0	1	0	0	2	0	1	2	1	1	1	1	0	0	0	0	0	Fungi; Ascomycota; Sordariomycetes; Microascales; Microascaceae; Pseudallescheria; Pseudallescheria_angusta
SH109826.06FU_HQ185354_refs	1	0	0	1	0	2	2	0	1	0	0	3	0	4	1	0	1	8	1	1	9	11	2	1	0	0	0	0	0	5	Fungi; Ascomycota; Sordariomycetes; Microascales; Microascaceae; Pseudallescheria; Pseudallescheria_angusta
SH109827.06FU_AB489074_reps	0	2	0	1	0	6	0	0	1	0	1	1	1	1	2	0	0	1	0	0	5	5	1	0	0	0	2	0	1	1	Fungi; Ascomycota; Sordariomycetes; Microascales; Microascaceae; Pseudallescheria; Pseudallescheria_angusta
SH109828.06FU_AB567745_reps	3	1	0	1	0	0	3	2	0	0	1	1	0	0	2	0	0	1	0	0	3	5	0	1	0	0	0	0	0	0	Fungi; Ascomycota; Sordariomycetes; Microascales; Microascaceae; Pseudallescheria; Pseudallescheria_angusta
SH109829.06FU_AY877351_reps	0	1	0	1	0	1	0	0	0	0	0	0	0	0	0	0	0	4	1	0	1	0	0	0	0	0	0	0	1	0	Fungi; Ascomycota; Sordariomycetes; Microascales; Microascaceae; Pseudallescheria; Pseudallescheria_angusta
SH109830.06FU_AY228122_reps	1	0	0	1	0	2	0	0	0	0	0	2	0	0	1	0	0	0	0	0	0	5	0	0	0	0	0	0	0	0	Fungi; Ascomycota; Sordariomycetes; Microascales; Microascaceae; Pseudallescheria; Pseudallescheria_angusta
New.ReferenceOTU5927	0	0	0	0	0	0	0	0	0	0	0	0	0	0	0	0	0	0	0	0	0	4	0	1	0	1	0	0	0	0	Fungi; Ascomycota; Sordariomycetes; Microascales; Microascaceae; Scedosporium; Scedosporium_aurantiacum
SH014567.06FU_HQ231818_reps_singleton	0	0	0	0	0	0	0	0	0	0	0	0	0	1	0	0	0	0	0	0	0	2	0	4	0	0	0	0	0	0	Fungi; Ascomycota; Sordariomycetes; Microascales; Microascaceae; Scedosporium; Scedosporium_aurantiacum
New.ReferenceOTU5608	0	0	0	0	0	1	0	1	0	0	0	0	0	0	1	0	4	2	0	0	0	0	1	0	0	0	0	0	0	0	Fungi; Ascomycota; Sordariomycetes; Microascales; Microascaceae; Scedosporium; Scedosporium_minutispora
SH141717.06FU_FJ713089_reps	0	0	0	0	0	0	0	0	0	0	0	0	0	2	0	0	0	0	0	0	0	0	0	0	0	0	0	0	0	0	Fungi; Ascomycota; Sordariomycetes; Microascales; Microascaceae; Scedosporium; Scedosporium_prolificans
New.ReferenceOTU2888	0	2	0	0	0	0	0	1	0	0	0	0	1	0	3	1	0	0	0	1	0	0	1	0	0	0	0	1	0	0	Fungi; Ascomycota; Sordariomycetes; Microascales; Microascaceae; Scopulariopsis; Scopulariopsis_humicola
SH026309.06FU_HQ607921_reps_singleton	0	1	0	0	0	0	0	0	0	0	0	0	0	0	1	0	1	0	1	0	0	0	0	0	0	0	0	0	0	0	Fungi; Ascomycota; Sordariomycetes; Microascales; Microascaceae; Scopulariopsis; Scopulariopsis_humicola
SH026310.06FU_HE584946_reps_singleton	0	0	0	0	0	0	0	0	0	0	0	0	0	1	1	0	0	0	0	0	0	0	0	0	0	0	0	0	0	0	Fungi; Ascomycota; Sordariomycetes; Microascales; Microascaceae; Scopulariopsis; Scopulariopsis_humicola
New.ReferenceOTU5984	0	0	0	0	0	0	0	0	0	0	0	0	0	0	0	0	0	0	0	0	0	0	0	0	0	0	2	0	0	5	Fungi; Ascomycota; Sordariomycetes; Microascales; Microascaceae; Scopulariopsis; Scopulariopsis_sp_2468
SH095789.06FU_AY625065_reps	0	0	0	0	0	0	0	0	0	0	0	0	0	0	0	0	0	0	0	0	0	0	0	0	0	0	1	0	0	2	Fungi; Ascomycota; Sordariomycetes; Microascales; Microascaceae; Scopulariopsis; Scopulariopsis_sp_2468
New.CleanUp.ReferenceOTU1373	0	0	0	0	0	0	0	0	0	0	0	0	0	0	0	0	0	0	0	0	0	0	1	0	0	0	0	0	0	0	Fungi; Ascomycota; Sordariomycetes; Ophiostomatales; Ophiostomataceae; Hyalorhinocladiella; Hyalorhinocladiella_sp_C_C2303
New.ReferenceOTU3629	0	2	0	0	0	2	0	0	0	1	0	0	0	0	0	0	0	0	0	0	0	0	5	0	0	0	0	0	4	2	Fungi; Ascomycota; Sordariomycetes; Sordariales; Cephalothecaceae; Cephalotheca; Cephalotheca_sp
New.ReferenceOTU594	0	0	0	0	0	0	0	0	0	0	0	0	0	0	0	0	0	0	2	0	0	0	0	0	0	0	0	0	0	0	Fungi; Ascomycota; Sordariomycetes; Sordariales; Cephalothecaceae; Cephalotheca; Cephalotheca_sp
New.ReferenceOTU62	0	0	0	0	0	0	0	0	0	1	0	0	0	0	0	0	0	0	0	0	0	0	0	0	0	0	0	0	0	0	Fungi; Ascomycota; Sordariomycetes; Sordariales; Cephalothecaceae; Cephalotheca; Cephalotheca_sp
New.ReferenceOTU6282	0	1	0	0	0	3	0	0	0	0	2	0	1	2	0	1	0	0	0	0	0	0	0	6	0	0	0	0	10	2	Fungi; Ascomycota; Sordariomycetes; Sordariales; Cephalothecaceae; Cephalotheca; Cephalotheca_sp
New.ReferenceOTU88	0	0	0	0	0	0	0	0	0	0	0	0	0	0	0	0	0	0	0	0	0	0	0	0	0	0	0	0	0	1	Fungi; Ascomycota; Sordariomycetes; Sordariales; Cephalothecaceae; Cephalotheca; Cephalotheca_sp
SH442025.06FU_AY273336_reps_singleton	0	0	0	1	0	9	0	0	0	0	0	0	2	0	0	0	0	0	0	0	0	0	0	3	0	0	0	0	0	2	Fungi; Ascomycota; Sordariomycetes; Sordariales; Cephalothecaceae; Cephalotheca; Cephalotheca_sp
New.ReferenceOTU1030	0	0	0	0	0	0	0	0	0	0	0	0	0	0	9	8	0	0	0	0	1	17	3	0	0	0	11	0	0	2	Fungi; Ascomycota; Sordariomycetes; Sordariales; Chaetomiaceae; Chaetomium; Chaetomium_atrobrunneum
New.ReferenceOTU2024	0	0	0	0	0	0	0	0	0	0	0	0	0	0	4	0	0	0	0	0	0	1	0	0	0	0	1	0	0	0	Fungi; Ascomycota; Sordariomycetes; Sordariales; Chaetomiaceae; Chaetomium; Chaetomium_atrobrunneum
New.ReferenceOTU8016	0	0	0	0	0	0	0	0	0	0	0	0	0	0	1	0	0	0	0	0	0	0	0	0	0	0	0	0	0	0	Fungi; Ascomycota; Sordariomycetes; Sordariales; Chaetomiaceae; Chaetomium; Chaetomium_atrobrunneum
New.CleanUp.ReferenceOTU764	0	0	0	0	0	0	1	0	0	0	0	0	0	0	0	0	0	0	0	0	0	0	0	0	0	0	0	0	0	0	Fungi; Ascomycota; Sordariomycetes; Sordariales; Chaetomiaceae; Chaetomium; Chaetomium_aureum
SH123788.06FU_KF245432_reps	0	0	0	0	0	0	0	0	0	0	0	0	0	0	0	0	0	0	0	0	0	0	0	0	0	0	0	7	0	0	Fungi; Ascomycota; Sordariomycetes; Sordariales; Chaetomiaceae; Chaetomium; Chaetomium_aureum
New.ReferenceOTU2861	0	0	0	0	0	0	0	0	0	2	0	0	0	0	0	0	0	0	0	0	1	0	1	0	6	0	1	0	0	4	Fungi; Ascomycota; Sordariomycetes; Sordariales; Chaetomiaceae; Chaetomium; Chaetomium_bostrychodes
SH139454.06FU_JX280779_reps	0	0	0	2	0	0	1	1	0	0	1	1	0	0	1	0	0	0	0	0	0	1	0	0	49	0	1	3	0	8	Fungi; Ascomycota; Sordariomycetes; Sordariales; Chaetomiaceae; Chaetomium; Chaetomium_bostrychodes
New.CleanUp.ReferenceOTU503	0	0	0	0	0	0	0	0	0	0	0	1	0	0	0	0	0	0	0	0	0	0	0	0	0	0	0	0	0	0	Fungi; Ascomycota; Sordariomycetes; Sordariales; Chaetomiaceae; Chaetomium; Chaetomium_cupreum
New.ReferenceOTU5719	0	0	1	0	4	33	2	0	0	1	0	29	4	0	1	0	2	0	0	0	0	3	2	0	0	0	0	23	0	0	Fungi; Ascomycota; Sordariomycetes; Sordariales; Chaetomiaceae; Chaetomium; Chaetomium_cupreum
New.ReferenceOTU5884	0	0	0	0	0	0	0	0	0	0	0	0	0	0	0	0	2	0	0	0	0	0	0	0	0	0	0	0	0	0	Fungi; Ascomycota; Sordariomycetes; Sordariales; Chaetomiaceae; Chaetomium; Chaetomium_cupreum
New.ReferenceOTU6539	0	0	0	0	0	0	0	0	0	0	0	2	0	0	0	0	0	0	0	0	0	0	0	0	0	0	0	4	0	0	Fungi; Ascomycota; Sordariomycetes; Sordariales; Chaetomiaceae; Chaetomium; Chaetomium_cupreum
New.ReferenceOTU6177	0	0	19	0	3	0	49	0	6	1	0	17	1	16	0	0	11	0	0	1	65	0	22	1	0	0	1	26	0	13	Fungi; Ascomycota; Sordariomycetes; Sordariales; Chaetomiaceae; Chaetomium; Chaetomium_erectum
New.ReferenceOTU3461	0	0	0	0	0	0	0	0	0	0	0	0	0	0	0	0	0	0	0	0	1	3	3	0	17	0	6	0	0	1	Fungi; Ascomycota; Sordariomycetes; Sordariales; Chaetomiaceae; Chaetomium; Chaetomium_globosum
SH033392.06FU_AY303604_reps_singleton	0	0	0	0	0	0	0	0	0	0	0	0	0	0	0	0	0	0	0	0	0	0	0	0	1	0	0	0	0	0	Fungi; Ascomycota; Sordariomycetes; Sordariales; Chaetomiaceae; Chaetomium; Chaetomium_globosum
SH139458.06FU_JX280846_reps	0	0	0	0	0	0	0	0	0	0	0	0	0	0	0	0	0	0	0	0	0	0	0	0	1	0	0	0	0	2	Fungi; Ascomycota; Sordariomycetes; Sordariales; Chaetomiaceae; Chaetomium; Chaetomium_globosum
SH139462.06FU_KC800568_reps	0	0	0	0	0	0	0	0	0	0	0	0	0	0	0	0	0	0	0	0	0	1	0	0	0	0	0	0	0	0	Fungi; Ascomycota; Sordariomycetes; Sordariales; Chaetomiaceae; Chaetomium; Chaetomium_globosum
New.ReferenceOTU7716	0	7	0	0	0	0	0	0	0	0	0	0	0	0	0	0	0	0	0	0	0	0	0	0	0	0	0	0	0	0	Fungi; Ascomycota; Sordariomycetes; Sordariales; Chaetomiaceae; Chaetomium; Chaetomium_grande
New.ReferenceOTU7310	0	0	0	0	0	0	0	0	0	2	0	0	0	0	0	0	0	0	0	0	0	1	0	0	0	0	0	0	0	0	Fungi; Ascomycota; Sordariomycetes; Sordariales; Chaetomiaceae; Chaetomium; Chaetomium_incomptum
New.ReferenceOTU7717	0	1	0	0	0	0	0	0	0	0	0	0	1	0	0	0	0	0	0	0	0	0	0	0	0	0	0	0	0	0	Fungi; Ascomycota; Sordariomycetes; Sordariales; Chaetomiaceae; Chaetomium; Chaetomium_mareoticum
SH139445.06FU_HM449051_refs	0	0	0	0	0	0	0	0	0	1	0	0	0	0	0	0	0	0	0	0	0	0	0	0	0	0	0	0	0	0	Fungi; Ascomycota; Sordariomycetes; Sordariales; Chaetomiaceae; Chaetomium; Chaetomium_reflexum
New.CleanUp.ReferenceOTU1719	1	0	0	0	0	0	0	0	0	0	0	0	0	0	0	0	0	0	0	0	0	0	0	0	0	0	0	0	0	0	Fungi; Ascomycota; Sordariomycetes; Sordariales; Chaetomiaceae; Chaetomium; Chaetomium_sp
New.ReferenceOTU1021	2	1	1	0	2	8	0	0	4	1	2	4	0	0	0	0	1	12	0	5	0	2	0	1	0	0	0	0	4	0	Fungi; Ascomycota; Sordariomycetes; Sordariales; Chaetomiaceae; Chaetomium; Chaetomium_sp
New.ReferenceOTU1398	0	0	0	0	0	0	0	0	0	9	8	0	0	0	0	0	2	0	10	3	0	0	0	0	0	0	0	0	0	0	Fungi; Ascomycota; Sordariomycetes; Sordariales; Chaetomiaceae; Chaetomium; Chaetomium_sp
New.ReferenceOTU1491	0	0	0	0	2	0	1	0	0	0	0	1	1	0	0	1	0	0	0	0	0	0	0	1	0	0	0	0	1	0	Fungi; Ascomycota; Sordariomycetes; Sordariales; Chaetomiaceae; Chaetomium; Chaetomium_sp
New.ReferenceOTU1552	0	0	0	0	0	0	0	0	0	0	0	0	0	0	0	0	0	0	0	1	0	0	0	0	0	0	0	0	0	0	Fungi; Ascomycota; Sordariomycetes; Sordariales; Chaetomiaceae; Chaetomium; Chaetomium_sp
New.ReferenceOTU1897	0	0	0	0	0	0	1	0	0	0	0	0	0	0	0	0	0	0	0	0	0	1	0	0	0	0	0	3	0	0	Fungi; Ascomycota; Sordariomycetes; Sordariales; Chaetomiaceae; Chaetomium; Chaetomium_sp
New.ReferenceOTU1992	1	1	0	1	3	0	0	0	10	1	3	0	0	1	1	0	0	3	4	6	1	2	1	0	1	0	0	0	0	0	Fungi; Ascomycota; Sordariomycetes; Sordariales; Chaetomiaceae; Chaetomium; Chaetomium_sp
New.ReferenceOTU1999	0	0	0	0	5	0	0	0	6	0	0	0	0	0	2	0	1	1	0	1	0	0	1	0	0	0	0	0	1	0	Fungi; Ascomycota; Sordariomycetes; Sordariales; Chaetomiaceae; Chaetomium; Chaetomium_sp
New.ReferenceOTU2078	0	0	0	0	0	0	0	0	0	0	0	1	0	0	0	0	0	0	1	0	0	0	0	2	1	0	0	1	0	0	Fungi; Ascomycota; Sordariomycetes; Sordariales; Chaetomiaceae; Chaetomium; Chaetomium_sp
New.ReferenceOTU2702	0	0	0	0	0	0	0	0	0	0	2	0	0	0	1	0	2	0	0	0	0	0	0	0	0	0	0	0	0	0	Fungi; Ascomycota; Sordariomycetes; Sordariales; Chaetomiaceae; Chaetomium; Chaetomium_sp
New.ReferenceOTU3532	4	3	0	0	2	4	1	0	0	1	0	2	1	0	0	2	0	1	0	0	0	1	0	0	0	2	0	0	1	0	Fungi; Ascomycota; Sordariomycetes; Sordariales; Chaetomiaceae; Chaetomium; Chaetomium_sp
New.ReferenceOTU3593	0	0	0	0	0	0	0	0	0	0	0	0	0	0	0	0	0	0	0	0	0	0	0	0	0	0	0	3	0	0	Fungi; Ascomycota; Sordariomycetes; Sordariales; Chaetomiaceae; Chaetomium; Chaetomium_sp
New.ReferenceOTU3828	0	0	0	0	1	1	2	1	0	2	4	2	0	0	1	0	0	2	1	2	0	3	0	0	2	0	1	0	0	0	Fungi; Ascomycota; Sordariomycetes; Sordariales; Chaetomiaceae; Chaetomium; Chaetomium_sp
New.ReferenceOTU3840	0	5	0	0	0	4	1	0	0	0	0	0	0	0	1	0	0	0	0	0	0	0	0	34	0	3	0	0	8	0	Fungi; Ascomycota; Sordariomycetes; Sordariales; Chaetomiaceae; Chaetomium; Chaetomium_sp
New.ReferenceOTU3938	0	0	0	0	0	0	0	0	0	0	0	2	0	0	0	0	0	1	0	0	0	0	0	0	0	0	0	0	0	0	Fungi; Ascomycota; Sordariomycetes; Sordariales; Chaetomiaceae; Chaetomium; Chaetomium_sp
New.ReferenceOTU4449	0	0	0	0	3	0	4	0	1	0	2	0	0	0	0	2	0	0	0	1	0	0	0	0	0	0	0	5	0	0	Fungi; Ascomycota; Sordariomycetes; Sordariales; Chaetomiaceae; Chaetomium; Chaetomium_sp
New.ReferenceOTU5281	0	0	0	0	0	0	1	2	14	0	0	0	0	0	1	0	0	0	0	0	0	0	1	0	0	0	0	0	0	5	Fungi; Ascomycota; Sordariomycetes; Sordariales; Chaetomiaceae; Chaetomium; Chaetomium_sp
New.ReferenceOTU5531	0	0	0	0	0	0	0	0	0	0	0	0	0	0	3	0	0	0	0	0	0	0	0	0	0	0	0	0	0	0	Fungi; Ascomycota; Sordariomycetes; Sordariales; Chaetomiaceae; Chaetomium; Chaetomium_sp
New.ReferenceOTU5647	0	0	0	0	0	0	0	0	0	0	0	0	0	0	0	0	0	1	0	0	0	0	0	0	0	0	0	0	0	0	Fungi; Ascomycota; Sordariomycetes; Sordariales; Chaetomiaceae; Chaetomium; Chaetomium_sp
New.ReferenceOTU599	0	0	0	0	0	0	0	0	1	0	0	0	0	0	0	0	0	0	0	0	0	0	0	0	0	0	0	0	0	0	Fungi; Ascomycota; Sordariomycetes; Sordariales; Chaetomiaceae; Chaetomium; Chaetomium_sp
New.ReferenceOTU6210	0	0	0	0	0	0	0	0	0	0	0	0	0	0	0	0	0	13	0	0	0	0	0	1	0	0	0	0	0	0	Fungi; Ascomycota; Sordariomycetes; Sordariales; Chaetomiaceae; Chaetomium; Chaetomium_sp
New.ReferenceOTU6471	0	0	0	0	0	0	0	0	0	1	0	0	0	0	0	0	0	0	0	0	0	0	0	0	0	0	0	0	0	0	Fungi; Ascomycota; Sordariomycetes; Sordariales; Chaetomiaceae; Chaetomium; Chaetomium_sp
New.ReferenceOTU6786	0	0	0	0	0	0	0	0	0	1	0	0	0	0	0	0	2	0	0	0	0	0	0	0	0	0	0	0	0	0	Fungi; Ascomycota; Sordariomycetes; Sordariales; Chaetomiaceae; Chaetomium; Chaetomium_sp
New.ReferenceOTU7881	0	0	0	0	0	0	0	0	0	0	0	0	0	0	0	0	0	0	1	0	0	0	0	0	0	0	0	0	0	0	Fungi; Ascomycota; Sordariomycetes; Sordariales; Chaetomiaceae; Chaetomium; Chaetomium_sp
New.ReferenceOTU963	0	0	0	0	0	0	0	0	0	0	0	0	0	0	0	0	0	1	0	0	0	0	0	0	0	0	0	0	0	0	Fungi; Ascomycota; Sordariomycetes; Sordariales; Chaetomiaceae; Chaetomium; Chaetomium_sp
SH139400.06FU_FJ612679_reps	18	8	3	5	4	16	2	0	5	12	6	2	2	7	2	3	2	26	4	9	0	2	0	8	0	8	0	1	5	0	Fungi; Ascomycota; Sordariomycetes; Sordariales; Chaetomiaceae; Chaetomium; Chaetomium_sp
SH139401.06FU_EU622257_reps	0	0	0	0	1	0	2	0	4	1	1	1	1	0	0	0	0	0	4	3	0	0	0	3	0	4	1	2	1	0	Fungi; Ascomycota; Sordariomycetes; Sordariales; Chaetomiaceae; Chaetomium; Chaetomium_sp
New.ReferenceOTU5994	0	0	0	0	0	0	0	0	0	0	0	0	0	0	0	0	0	0	0	0	0	0	0	0	0	2	0	0	0	0	Fungi; Ascomycota; Sordariomycetes; Sordariales; Chaetomiaceae; Chaetomium; Chaetomium_sp_3_SA_2013
SH139397.06FU_AB625592_refs	0	0	0	0	0	0	0	0	0	0	0	0	0	0	0	2	0	0	0	0	0	0	0	0	0	0	0	0	0	0	Fungi; Ascomycota; Sordariomycetes; Sordariales; Chaetomiaceae; Humicola; Humicola_nigrescens
New.ReferenceOTU1816	0	0	0	0	0	0	0	1	0	0	0	0	0	0	0	0	0	0	0	0	0	0	0	0	0	0	0	0	0	0	Fungi; Ascomycota; Sordariomycetes; Sordariales; Chaetomiaceae; Thielavia; Thielavia_intermedia
New.ReferenceOTU4400	0	0	0	0	0	0	0	0	0	0	0	0	0	0	0	0	1	0	0	0	0	0	0	0	0	0	0	0	0	0	Fungi; Ascomycota; Sordariomycetes; Sordariales; Chaetomiaceae; Thielavia; Thielavia_minuta
New.ReferenceOTU6661	0	0	0	0	0	0	0	0	0	0	0	0	0	0	0	0	0	0	0	0	0	0	0	0	0	0	8	0	0	1	Fungi; Ascomycota; Sordariomycetes; Sordariales; Chaetomiaceae; Trichocladium; Trichocladium_pyriforme
SH024384.06FU_AM292048_reps_singleton	0	1	2	0	0	4	7	0	1	6	1	0	2	40	3	3	8	0	0	0	2	0	4	1	2	33	55	3	0	19	Fungi; Ascomycota; Sordariomycetes; Sordariales; Chaetomiaceae; Trichocladium; Trichocladium_pyriforme
New.ReferenceOTU51	0	0	0	0	0	0	0	0	4	0	0	0	0	0	0	0	0	0	0	0	0	0	0	0	0	0	0	0	0	0	Fungi; Ascomycota; Sordariomycetes; Sordariales; Helminthosphaeriaceae; Spadicoides; Spadicoides_bina
SH091853.06FU_DQ420778_reps	0	0	0	0	2	0	1	0	0	0	0	1	0	0	0	0	0	0	0	0	0	0	0	0	0	0	0	0	0	0	Fungi; Ascomycota; Sordariomycetes; Sordariales; Lasiosphaeriaceae; Arnium; Arnium_sp
SH122455.06FU_GU055576_reps	0	0	0	0	0	0	0	0	0	0	0	0	0	0	0	0	0	0	0	0	0	1	0	0	0	0	0	0	0	0	Fungi; Ascomycota; Sordariomycetes; Sordariales; Lasiosphaeriaceae; Cercophora; Cercophora_caudata
New.CleanUp.ReferenceOTU631	0	0	0	0	0	0	0	0	0	0	0	0	0	0	0	0	0	0	1	0	0	0	0	0	0	0	0	0	0	0	Fungi; Ascomycota; Sordariomycetes; Sordariales; Lasiosphaeriaceae; Cladorrhinum; Cladorrhinum_sp
SH126104.06FU_JF432978_reps	0	0	0	0	0	0	0	0	0	0	0	0	0	0	0	0	0	0	1	0	0	0	0	0	0	0	0	0	0	0	Fungi; Ascomycota; Sordariomycetes; Sordariales; Lasiosphaeriaceae; Cladorrhinum; Cladorrhinum_sp

New.ReferenceOTU3206	0	0	0	0	0	0	0	0	0	0	0	0	0	0	0	0	2	0	0	0	0	0	0	0	0	0	0	0	0	0	Fungi; Ascomycota; Sordariomycetes; Sordariales; Lasiosphaeriaceae; Podospora; Podospora_sp
New.ReferenceOTU6417	0	0	0	0	0	0	0	0	0	0	0	0	0	0	0	0	0	0	0	1	0	0	0	0	0	0	0	0	0	0	Fungi; Ascomycota; Sordariomycetes; Sordariales; Lasiosphaeriaceae; Podospora; Podospora_sp
SH139403.06FU_FJ175159_refs	0	0	0	0	0	0	0	0	0	0	0	0	0	0	0	0	0	0	0	0	0	0	0	0	1	0	0	0	0	0	Fungi; Ascomycota; Sordariomycetes; Sordariales; Lasiosphaeriaceae; Zopfiella; Zopfiella_marina
SH139452.06FU_EU685998_reps	0	0	1	0	0	0	0	0	0	0	0	0	0	0	0	0	0	0	0	0	0	0	0	0	0	0	0	0	0	0	Fungi; Ascomycota; Sordariomycetes; Sordariales; Lasiosphaeriaceae; Zopfiella; Zopfiella_sp
New.ReferenceOTU2195	0	0	0	0	0	0	0	0	0	0	0	0	0	0	0	0	0	0	0	0	0	0	0	1	0	0	0	0	0	0	Fungi; Ascomycota; Sordariomycetes; Sordariales; Lasiosphaeriaceae; Zopfiella; Zopfiella_sp_TMS_2011
New.ReferenceOTU2533	8	2	0	0	0	0	1	0	0	0	0	2	0	0	0	0	0	1	1	0	0	4	0	2	0	8	0	0	7	0	Fungi; Ascomycota; Sordariomycetes; Sordariales; Sordariaceae; Coronatomyces; Coronatomyces_cubensis
New.ReferenceOTU3658	0	0	0	0	0	5	2	1	0	1	0	0	1	0	0	0	0	0	0	0	0	0	0	0	0	0	0	0	0	0	Fungi; Ascomycota; Sordariomycetes; Sordariales; Sordariaceae; Neurospora; Neurospora_terricola
New.ReferenceOTU7194	0	0	0	0	2	39	43	0	0	3	2	9	1	5	1	1	0	0	0	0	2	2	1	1	2	0	1	20	0	3	Fungi; Ascomycota; Sordariomycetes; Sordariales; Sordariaceae; Neurospora; Neurospora_terricola
SH016572.06FU_JQ759806_reps_singleton	0	0	0	0	0	0	0	0	0	0	0	0	0	0	0	0	0	0	0	0	0	0	0	0	0	0	0	1	0	0	Fungi; Ascomycota; Sordariomycetes; Sordariales; Sordariaceae; Neurospora; Neurospora_terricola
SH016573.06FU_JQ759755_reps_singleton	0	0	0	0	0	0	0	0	0	0	0	0	1	0	0	0	0	0	0	0	0	0	0	0	0	0	0	0	0	0	Fungi; Ascomycota; Sordariomycetes; Sordariales; Sordariaceae; Neurospora; Neurospora_terricola
SH113365.06FU_AY805577_reps	0	0	0	0	0	7	8	0	0	1	0	0	0	1	0	1	0	0	0	0	0	0	0	1	0	0	1	8	0	0	Fungi; Ascomycota; Sordariomycetes; Sordariales; Sordariaceae; Neurospora; Neurospora_terricola
SH113366.06FU_EU551188_reps	0	0	1	0	0	0	0	2	0	0	0	1	0	0	0	0	0	0	1	0	0	0	0	0	0	0	0	0	0	0	Fungi; Ascomycota; Sordariomycetes; Sordariales; Sordariaceae; Neurospora; Neurospora_terricola
SH113367.06FU_AF388928_refs	1	0	2	3	0	0	2	0	0	0	0	0	0	0	0	0	0	0	0	0	0	0	2	0	0	0	0	3	0	0	Fungi; Ascomycota; Sordariomycetes; Sordariales; Sordariaceae; Neurospora; Neurospora_terricola
New.ReferenceOTU2736	0	0	0	0	0	0	0	0	0	0	0	0	0	0	0	1	0	0	0	0	0	0	0	0	0	0	0	0	0	0	Fungi; Ascomycota; Sordariomycetes; Trichosphaeriales; Incertae_sedis; Khuskia; Khuskia_oryzae
SH034960.06FU_JX966550_reps_singleton	0	0	0	0	0	0	0	1	0	0	0	0	0	0	0	0	0	0	0	0	0	0	0	0	0	0	0	0	0	0	Fungi; Ascomycota; Sordariomycetes; Trichosphaeriales; Incertae_sedis; Khuskia; Khuskia_oryzae
SH034973.06FU_HQ904082_reps_singleton	0	0	0	0	0	0	0	0	0	0	0	0	0	1	0	0	0	0	0	0	0	0	0	0	0	0	0	0	0	0	Fungi; Ascomycota; Sordariomycetes; Trichosphaeriales; Incertae_sedis; Khuskia; Khuskia_oryzae
SH034976.06FU_JQ814364_reps_singleton	0	0	0	0	0	0	0	0	0	0	0	1	0	0	0	0	0	0	0	0	0	0	0	0	0	0	0	0	1	1	Fungi; Ascomycota; Sordariomycetes; Trichosphaeriales; Incertae_sedis; Khuskia; Khuskia_oryzae
SH034977.06FU_JQ814305_reps_singleton	0	0	1	0	0	1	3	5	0	2	0	1	0	1	0	3	6	0	0	0	0	2	0	0	0	0	2	1	0	1	Fungi; Ascomycota; Sordariomycetes; Trichosphaeriales; Incertae_sedis; Khuskia; Khuskia_oryzae
SH142000.06FU_KC806231_reps	0	0	0	0	0	0	0	1	0	0	0	0	0	2	0	0	0	0	0	0	0	0	0	0	0	0	0	0	0	0	Fungi; Ascomycota; Sordariomycetes; Trichosphaeriales; Incertae_sedis; Khuskia; Khuskia_oryzae
SH142011.06FU_JX006067_reps	0	0	0	0	0	0	0	1	0	0	0	0	0	0	0	0	0	0	0	0	0	0	0	0	0	0	0	0	0	0	Fungi; Ascomycota; Sordariomycetes; Trichosphaeriales; Incertae_sedis; Khuskia; Khuskia_oryzae
New.ReferenceOTU3496	0	0	0	0	0	0	0	0	2	0	1	0	0	0	0	0	0	1	0	1	0	0	0	0	0	0	0	0	0	0	Fungi; Ascomycota; Sordariomycetes; Trichosphaeriales; Incertae_sedis; Nigrospora; Nigrospora_sp
New.ReferenceOTU5344	0	1	3	0	0	0	2	4	1	1	0	0	0	0	0	1	1	0	0	0	2	1	0	1	1	0	1	0	0	1	Fungi; Ascomycota; Sordariomycetes; Trichosphaeriales; Incertae_sedis; Nigrospora; Nigrospora_sp_CASMB_SEF_29
New.ReferenceOTU3899	0	0	0	0	1	0	0	0	0	0	0	0	0	0	0	0	0	0	0	0	0	0	0	0	0	0	0	0	0	0	Fungi; Ascomycota; Sordariomycetes; Xylariales; Amphisphaeriaceae; Bartalinia; Bartalinia_sp_MM_101
SH034596.06FU_GU722596_reps_singleton	0	0	0	0	0	0	0	0	1	0	1	0	0	0	0	0	0	0	0	0	0	0	0	0	0	0	0	0	0	0	Fungi; Ascomycota; Sordariomycetes; Xylariales; Amphisphaeriaceae; Bartalinia; Bartalinia_sp_MM_101
New.ReferenceOTU1053	0	0	0	0	0	0	0	0	0	0	0	0	0	0	0	1	0	0	0	0	0	0	0	0	0	0	0	0	0	0	Fungi; Ascomycota; Sordariomycetes; Xylariales; Hyponectriaceae; Physalospora; Physalospora_sp
New.ReferenceOTU1964	0	0	0	0	0	0	0	0	0	0	0	0	0	0	0	0	0	0	1	0	0	0	0	0	0	0	0	0	0	0	Fungi; Ascomycota; Sordariomycetes; Xylariales; Incertae_sedis; Dinemasporium; Dinemasporium_americana
New.ReferenceOTU2595	0	0	0	0	0	0	0	0	0	0	0	0	0	0	0	0	0	0	0	0	0	0	0	0	0	0	0	0	6	0	Fungi; Ascomycota; Sordariomycetes; Xylariales; Xylariaceae; Annulohypoxylon; Annulohypoxylon_sp
New.ReferenceOTU609	0	0	0	0	0	0	0	0	0	0	0	0	0	0	0	0	0	0	0	0	0	0	1	0	0	0	0	0	0	0	Fungi; Ascomycota; Sordariomycetes; Xylariales; Xylariaceae; Annulohypoxylon; Annulohypoxylon_sp
SH139771.06FU_EU818915_reps	0	0	1	0	2	0	0	0	0	0	2	0	0	0	0	0	0	0	0	0	0	0	0	0	0	0	0	0	0	0	Fungi; Ascomycota; Sordariomycetes; Xylariales; Xylariaceae; Annulohypoxylon; Annulohypoxylon_sp
New.ReferenceOTU557	0	0	0	0	0	0	0	0	0	0	0	0	0	0	0	0	0	0	0	0	0	0	0	0	0	0	0	0	0	1	Fungi; Ascomycota; Sordariomycetes; Xylariales; Xylariaceae; Anthostomella; Anthostomella_sp
SH032717.06FU_JQ411351_reps_singleton	0	0	0	0	0	0	0	0	0	0	0	0	0	0	0	0	0	0	0	0	0	0	0	0	1	0	0	0	0	0	Fungi; Ascomycota; Sordariomycetes; Xylariales; Xylariaceae; Anthostomella; Anthostomella_sp_1_CCG_2012
New.ReferenceOTU5441	0	0	0	0	0	0	0	0	0	0	0	0	0	0	0	0	0	0	0	0	0	2	0	0	0	0	1	0	0	0	Fungi; Ascomycota; Sordariomycetes; Xylariales; Xylariaceae; Ascotricha; Ascotricha_sp_OUCMB0118
New.ReferenceOTU5303	0	0	0	0	0	0	1	0	0	0	0	0	0	0	0	0	0	0	0	0	0	0	0	0	0	0	0	0	0	0	Fungi; Ascomycota; Sordariomycetes; Xylariales; Xylariaceae; Biscogniauxia; Biscogniauxia_arima
New.ReferenceOTU6012	0	0	0	0	0	0	0	1	0	0	0	0	0	0	0	0	0	0	0	0	0	0	0	0	0	0	0	0	0	0	Fungi; Ascomycota; Sordariomycetes; Xylariales; Xylariaceae; Biscogniauxia; Biscogniauxia_nummularia
SH144424.06FU_AJ246229_reps	0	0	0	0	0	0	0	0	0	0	0	0	0	3	0	2	0	0	0	0	0	0	0	0	0	0	0	0	0	0	Fungi; Ascomycota; Sordariomycetes; Xylariales; Xylariaceae; Biscogniauxia; Biscogniauxia_nummularia
SH144425.06FU_AJ390415_reps	0	0	0	0	1	0	0	0	0	0	0	0	0	1	1	0	0	0	0	0	0	0	0	1	0	0	0	0	0	0	Fungi; Ascomycota; Sordariomycetes; Xylariales; Xylariaceae; Biscogniauxia; Biscogniauxia_nummularia
New.ReferenceOTU1945	0	1	0	2	0	0	0	0	0	0	2	0	0	1	0	0	0	0	0	0	0	0	0	0	0	0	0	0	0	0	Fungi; Ascomycota; Sordariomycetes; Xylariales; Xylariaceae; Biscogniauxia; Biscogniauxia_philippinensis_var_microspora
New.ReferenceOTU6130	0	0	0	0	0	0	0	0	0	0	0	0	0	0	0	1	0	0	0	0	0	0	0	0	0	0	0	0	0	0	Fungi; Ascomycota; Sordariomycetes; Xylariales; Xylariaceae; Biscogniauxia; Biscogniauxia_philippinensis_var_microspora
New.ReferenceOTU7890	0	0	0	2	0	0	0	0	0	0	0	0	0	0	0	0	0	0	0	0	0	0	0	0	0	0	0	0	0	0	Fungi; Ascomycota; Sordariomycetes; Xylariales; Xylariaceae; Biscogniauxia; Biscogniauxia_sp
New.ReferenceOTU4098	0	0	0	0	1	0	0	0	3	0	0	0	0	0	0	0	0	1	0	0	0	0	0	1	0	0	0	0	1	0	Fungi; Ascomycota; Sordariomycetes; Xylariales; Xylariaceae; Biscogniauxia; Biscogniauxia_sp_2_ICMP_18793
New.ReferenceOTU429	0	0	0	0	0	0	0	0	0	1	6	0	0	0	0	0	0	0	0	0	0	0	0	0	0	0	0	0	0	0	Fungi; Ascomycota; Sordariomycetes; Xylariales; Xylariaceae; Biscogniauxia; Biscogniauxia_sp_2_ICMP_18793
New.ReferenceOTU987	0	0	0	0	0	0	0	0	0	0	1	0	0	0	0	0	0	0	0	0	0	0	0	0	0	0	0	0	0	0	Fungi; Ascomycota; Sordariomycetes; Xylariales; Xylariaceae; Biscogniauxia; Biscogniauxia_sp_2_ICMP_18793
New.ReferenceOTU5767	0	1	0	0	0	0	0	0	0	0	1	0	0	0	0	0	0	0	0	0	0	0	0	0	0	0	0	0	0	0	Fungi; Ascomycota; Sordariomycetes; Xylariales; Xylariaceae; Daldinia; Daldinia_caldariorum
New.ReferenceOTU5396	0	0	0	0	0	0	0	0	0	0	0	0	0	0	0	0	0	0	0	0	0	0	1	0	0	0	0	0	0	0	Fungi; Ascomycota; Sordariomycetes; Xylariales; Xylariaceae; Daldinia; Daldinia_fissa
New.ReferenceOTU6936	0	0	0	0	0	1	0	0	1	0	0	0	2	0	1	0	2	0	0	0	1	0	0	0	0	0	0	1	0	0	Fungi; Ascomycota; Sordariomycetes; Xylariales; Xylariaceae; Daldinia; Daldinia_fissa
New.ReferenceOTU3586	0	0	0	0	1	0	0	0	0	0	0	0	0	0	0	0	0	0	0	0	0	0	0	0	0	0	0	0	0	0	Fungi; Ascomycota; Sordariomycetes; Xylariales; Xylariaceae; Geniculisynnema; Geniculisynnema_termiticola
New.ReferenceOTU2390	0	0	0	0	0	0	0	2	0	0	0	0	0	0	0	0	0	0	0	0	0	0	0	0	0	0	0	0	0	0	Fungi; Ascomycota; Sordariomycetes; Xylariales; Xylariaceae; Hypoxylon; Hypoxylon_aeruginosum
SH440751.06FU_KC968941_reps_singleton	0	0	0	0	0	0	0	1	0	0	0	0	1	0	0	0	0	0	0	0	0	0	0	0	0	0	0	0	0	0	Fungi; Ascomycota; Sordariomycetes; Xylariales; Xylariaceae; Hypoxylon; Hypoxylon_aeruginosum
SH117677.06FU_JQ697538_reps	0	0	0	1	0	0	0	0	0	0	0	0	0	0	0	0	0	0	0	0	0	0	0	0	0	0	0	0	0	0	Fungi; Ascomycota; Sordariomycetes; Xylariales; Xylariaceae; Hypoxylon; Hypoxylon_fragiforme
New.ReferenceOTU7973	0	0	0	0	0	0	1	0	1	0	1	1	0	0	1	0	0	0	0	0	0	0	0	0	0	0	0	0	0	0	Fungi; Ascomycota; Sordariomycetes; Xylariales; Xylariaceae; Hypoxylon; Hypoxylon_investiens
SH116796.06FU_KC968925_reps	0	0	1	0	0	0	0	0	1	0	1	1	0	0	1	0	0	0	0	0	0	0	0	0	0	0	0	0	0	0	Fungi; Ascomycota; Sordariomycetes; Xylariales; Xylariaceae; Hypoxylon; Hypoxylon_investiens
New.ReferenceOTU7523	0	0	0	0	0	0	0	0	0	1	0	0	0	0	0	0	0	0	0	0	0	0	0	0	0	0	0	0	0	0	Fungi; Ascomycota; Sordariomycetes; Xylariales; Xylariaceae; Hypoxylon; Hypoxylon_musceum
New.ReferenceOTU1417	0	0	0	0	1	0	0	0	0	0	0	0	0	0	0	0	0	0	0	0	0	0	0	0	0	0	0	0	0	0	Fungi; Ascomycota; Sordariomycetes; Xylariales; Xylariaceae; Hypoxylon; Hypoxylon_ochraceum
New.ReferenceOTU2722	0	0	0	0	0	0	0	0	0	0	1	0	0	0	0	0	0	0	0	0	0	0	0	0	0	0	0	0	0	0	Fungi; Ascomycota; Sordariomycetes; Xylariales; Xylariaceae; Hypoxylon; Hypoxylon_samuelsii
SH122891.06FU_JQ388253_reps	0	0	0	1	0	0	0	0	0	0	0	0	0	0	0	0	0	0	0	0	0	0	0	0	0	0	0	0	0	0	Fungi; Ascomycota; Sordariomycetes; Xylariales; Xylariaceae; Hypoxylon; Hypoxylon_sp_DoF8
SH024956.06FU_AB741594_reps_singleton	0	0	0	1	0	0	0	0	0	0	0	0	0	0	0	0	0	0	0	0	0	0	0	0	0	0	0	0	0	0	Fungi; Ascomycota; Sordariomycetes; Xylariales; Xylariaceae; Hypoxylon; Hypoxylon_sp_GZ_AT_M050
New.ReferenceOTU3904	0	0	0	0	0	0	0	0	0	0	0	0	0	0	0	1	0	0	0	0	0	0	0	0	0	0	0	0	0	1	Fungi; Ascomycota; Sordariomycetes; Xylariales; Xylariaceae; Hypoxylon; Hypoxylon_sp_HK_AT_PTC015
New.ReferenceOTU1544	0	0	0	0	0	0	0	0	0	0	0	1	0	0	0	0	0	0	0	0	0	0	0	0	0	0	0	0	0	0	Fungi; Ascomycota; Sordariomycetes; Xylariales; Xylariaceae; Hypoxylon; Hypoxylon_sp_HS_EF24
New.ReferenceOTU2919	0	0	0	0	0	0	0	0	0	2	0	1	0	0	0	0	0	0	0	0	0	0	0	1	0	0	0	0	0	0	Fungi; Ascomycota; Sordariomycetes; Xylariales; Xylariaceae; Hypoxylon; Hypoxylon_sp_MA156
New.ReferenceOTU3848	0	0	0	0	1	0	0	0	0	0	0	0	0	0	0	0	0	0	0	0	0	0	0	0	0	0	0	0	0	0	Fungi; Ascomycota; Sordariomycetes; Xylariales; Xylariaceae; Hypoxylon; Hypoxylon_sp_SUT148
New.ReferenceOTU902	1	0	0	0	0	0	0	0	0	0	0	0	0	0	0	0	0	0	0	0	0	0	0	0	0	0	0	0	0	0	Fungi; Ascomycota; Sordariomycetes; Xylariales; Xylariaceae; Hypoxylon; Hypoxylon_sp_SUT166
SH016453.06FU_JQ411355_reps_singleton	0	0	0	0	0	0	0	0	0	0	0	0	0	0	0	0	0	0	0	0	0	1	0	0	0	0	0	0	0	0	Fungi; Ascomycota; Sordariomycetes; Xylariales; Xylariaceae; Hypoxylon; Hypoxylon_sp_SUT166
SH113203.06FU_DQ322114_reps	2	0	1	0	0	1	0	0	1	0	2	5	0	1	0	0	0	0	0	0	0	0	0	1	0	0	0	0	0	0	Fungi; Ascomycota; Sordariomycetes; Xylariales; Xylariaceae; Hypoxylon; Hypoxylon_sp_SUT166
New.ReferenceOTU5485	0	0	0	0	2	0	0	0	0	0	0	0	0	0	0	0	0	0	0	0	0	0	0	0	0	0	0	0	0	0	Fungi; Ascomycota; Sordariomycetes; Xylariales; Xylariaceae; Kretzschmaria; Kretzschmaria_guyanensis
New.ReferenceOTU7486	0	0	0	0	0	0	0	0	0	0	0	0	0	0	0	0	0	0	0	4	0	0	0	0	0	0	0	0	0	0	Fungi; Ascomycota; Sordariomycetes; Xylariales; Xylariaceae; Kretzschmaria; Kretzschmaria_pavimentosa
New.ReferenceOTU2291	0	0	0	0	1	0	0	0	1	0	0	0	0	0	0	0	0	0	0	0	0	0	0	0	0	0	0	0	0	0	Fungi; Ascomycota; Sordariomycetes; Xylariales; Xylariaceae; Kretzschmaria; Kretzschmaria_sandvicensis
New.ReferenceOTU2933	0	0	0	0	0	0	0	0	0	0	0	0	0	1	0	0	0	0	0	0	0	0	0	0	0	0	0	0	0	0	Fungi; Ascomycota; Sordariomycetes; Xylariales; Xylariaceae; Kretzschmaria; Kretzschmaria_sandvicensis
SH108337.06FU_JQ862661_reps	0	0	0	0	0	0	0	0	0	0	0	0	0	0	0	0	0	0	1	0	0	0	0	0	0	0	0	3	0	0	Fungi; Ascomycota; Sordariomycetes; Xylariales; Xylariaceae; Nemania; Nemania_bipapillata
New.ReferenceOTU3679	0	0	0	0	0	0	0	1	0	0	0	0	0	0	0	0	0	0	0	0	0	0	0	0	0	0	0	0	0	0	Fungi; Ascomycota; Sordariomycetes; Xylariales; Xylariaceae; Nemania; Nemania_chestersii
New.ReferenceOTU2321	0	0	0	0	0	0	0	0	0	1	0	0	0	0	0	0	0	0	0	0	0	0	0	0	0	0	0	0	0	0	Fungi; Ascomycota; Sordariomycetes; Xylariales; Xylariaceae; Nemania; Nemania_serpens
New.ReferenceOTU1299	0	0	0	0	0	0	0	0	0	0	0	0	3	0	0	0	0	0	0	0	0	0	0	0	0	0	0	0	0	0	Fungi; Ascomycota; Sordariomycetes; Xylariales; Xylariaceae; Nodulisporium; Nodulisporium_sp
New.ReferenceOTU1720	0	0	0	0	0	0	0	0	0	0	0	0	0	0	0	0	0	0	0	0	0	0	0	0	0	0	1	0	0	0	Fungi; Ascomycota; Sordariomycetes; Xylariales; Xylariaceae; Nodulisporium; Nodulisporium_sp
SH095286.06FU_EU686008_reps	0	0	0	0	0	0	0	2	0	0	4	0	0	0	0	0	0	0	0	0	0	0	0	0	0	0	0	0	0	0	Fungi; Ascomycota; Sordariomycetes; Xylariales; Xylariaceae; Nodulisporium; Nodulisporium_sp
New.CleanUp.ReferenceOTU2030	0	0	0	0	0	0	1	0	0	0	0	0	0	0	0	0	0	0	0	0	0	0	0	0	0	0	0	0	0	0	Fungi; Ascomycota; Sordariomycetes; Xylariales; Xylariaceae; Nodulisporium; Nodulisporium_sp_Agha3
New.ReferenceOTU440	0	0	0	0	0	0	0	0	0	0	0	0	0	0	0	0	0	0	0	0	0	0	0	5	0	0	0	0	0	0	Fungi; Ascomycota; Sordariomycetes; Xylariales; Xylariaceae; Obolarina; Obolarina_dryophila
New.ReferenceOTU4168	0	0	0	0	0	0	0	0	1	0	0	0	0	0	0	0	0	0	0	0	0	0	0	0	1	0	0	0	0	0	Fungi; Ascomycota; Sordariomycetes; Xylariales; Xylariaceae; Rosellinia; Rosellinia_necatrix
SH126720.06FU_JQ809677_reps	0	0	0	0	0	0	0	0	0	0	0	0	0	1	0	0	0	0	0	0	0	0	0	0	0	0	0	0	0	0	Fungi; Ascomycota; Sordariomycetes; Xylariales; Xylariaceae; Rosellinia; Rosellinia_sp_U01
New.ReferenceOTU2695	0	0	0	0	0	0	0	0	0	0	0	0	0	0	2	0	0	0	0	0	0	1	0	0	0	0	1	0	0	0	Fungi; Ascomycota; Sordariomycetes; Xylariales; Xylariaceae; Rosellinia; Rosellinia_subiculata
New.ReferenceOTU1576	0	0	0	0	0	0	0	0	0	0	0	0	0	0	0	0	0	0	0	0	0	0	0	0	0	0	0	1	0	0	Fungi; Ascomycota; Sordariomycetes; Xylariales; Xylariaceae; Stromatoneurospora; Stromatoneurospora_phoenix
New.ReferenceOTU2990	0	0	0	0	0	0	0	0	0	0	0	0	0	0	0	0	0	0	0	0	0	0	1	0	0	0	0	0	0	0	Fungi; Ascomycota; Sordariomycetes; Xylariales; Xylariaceae; Stromatoneurospora; Stromatoneurospora_phoenix
SH024718.06FU_AY909004_reps_singleton	0	0	0	0	0	0	0	0	0	4	0	0	0	0	0	1	0	0	0	0	0	0	0	0	0	0	0	0	0	0	Fungi; Ascomycota; Sordariomycetes; Xylariales; Xylariaceae; Stromatoneurospora; Stromatoneurospora_phoenix
New.ReferenceOTU7777	0	0	0	0	0	0	0	0	0	0	1	0	0	0	0	0	0	0	0	0	0	0	0	0	0	0	0	0	0	0	Fungi; Ascomycota; Sordariomycetes; Xylariales; Xylariaceae; Virgaria; Virgaria_boninensis
New.ReferenceOTU345	0	0	2	0	0	0	0	0	0	0	1	0	0	0	0	0	0	0	0	0	0	0	0	0	0	0	0	0	0	0	Fungi; Ascomycota; Sordariomycetes; Xylariales; Xylariaceae; Virgaria; Virgaria_sp
New.ReferenceOTU3623	0	0	0	0	0	0	0	0	0	0	0	0	0	0	0	0	0	0	0	0	0	1	0	0	0	0	0	0	0	0	Fungi; Ascomycota; Sordariomycetes; Xylariales; Xylariaceae; Virgaria; Virgaria_sp
New.ReferenceOTU5797	0	0	1	0	0	0	0	0	0	0	0	0	0	0	0	0	0	0	0	0	0	0	0	0	0	0	0	0	0	0	Fungi; Ascomycota; Sordariomycetes; Xylariales; Xylariaceae; Virgaria; Virgaria_sp
New.ReferenceOTU3530	0	0	0	1	0	0	0	0	0	0	0	0	0	1	0	0	0	0	0	0	0	1	1	0	0	0	0	0	0	0	Fungi; Ascomycota; Sordariomycetes; Xylariales; Xylariaceae; Whalleya; Whalleya_sp
New.ReferenceOTU11	0	0	0	0	0	0	0	0	0	0	0	0	0	0	0	0	2	0	0	0	0	0	0	0	0	0	0	0	0	0	Fungi; Ascomycota; Sordariomycetes; Xylariales; Xylariaceae; Xylaria; Xylaria_globosa
New.ReferenceOTU4528	0	0	0	0	0	0	0	0	0	0	0	0	1	0	0	0	0	0	0	1	0	0	0	1	0	0	0	0	0	0	Fungi; Ascomycota; Sordariomycetes; Xylariales; Xylariaceae; Xylaria; Xylaria_griseosepiacea
New.ReferenceOTU1632	0	0	0	0	0	0	0	0	0	0	0	0	0	0	0	0	0	0	0	0	0	0	0	0	0	0	0	0	1	0	Fungi; Ascomycota; Sordariomycetes; Xylariales; Xylariaceae; Xylaria; Xylaria_hypoxylon
New.ReferenceOTU2671	0	0	0	0	0	0	0	0	0	0	0	0	0	0	0	0	0	0	0	0	1	0	0	0	0	0	0	0	0	0	Fungi; Ascomycota; Sordariomycetes; Xylariales; Xylariaceae; Xylaria; Xylaria_hypoxylon
New.ReferenceOTU3902	0	0	0	0	0	0	0	0	0	0	0	0	0	0	0	0	1	0	0	0	0	2	0	0	0	0	0	1	0	0	Fungi; Ascomycota; Sordariomycetes; Xylariales; Xylariaceae; Xylaria; Xylaria_hypoxylon
New.ReferenceOTU6106	0	2	0	0	0	0	0	0	0	0	0	0	0	0	0	0	0	0	0	0	0	0	0	0	0	0	0	0	0	0	Fungi; Ascomycota; Sordariomycetes; Xylariales; Xylariaceae; Xylaria; Xylaria_hypoxylon
New.ReferenceOTU742	0	0	0	0	0	0	0	0	0	0	0	0	0	1	0	0	0	0	0	0	0	0	0	1	0	0	0	3	0	0	Fungi; Ascomycota; Sordariomycetes; Xylariales; Xylariaceae; Xylaria; Xylaria_juruensis
New.ReferenceOTU4565	0	0	0	0	0	0	0	0	0	0	0	0	0	0	0	0	0	0	0	0	0	0	0	0	0	0	0	0	1	0	Fungi; Ascomycota; Sordariomycetes; Xylariales; Xylariaceae; Xylaria; Xylaria_laevis
New.ReferenceOTU2872	0	0	0	0	0	0	0	0	0	0	1	0	0	0	0	0	0	0	0	0	0	0	0	0	0	0	0	0	0	0	Fungi; Ascomycota; Sordariomycetes; Xylariales; Xylariaceae; Xylaria; Xylaria_longipes
New.ReferenceOTU4294	0	0	0	0	4	0	0	0	0	0	0	0	0	0	0	0	0	0	0	0	0	0	0	0	0	0	0	0	0	0	Fungi; Ascomycota; Sordariomycetes; Xylariales; Xylariaceae; Xylaria; Xylaria_muscula
New.ReferenceOTU3892	0	0	0	0	0	0	0	0	1	0	0	0	0	0	0	0	0	2	0	2	0	0	0	0	0	0	0	0	1	0	Fungi; Ascomycota; Sordariomycetes; Xylariales; Xylariaceae; Xylaria; Xylaria_nigripes
New.ReferenceOTU5008	0	0	0	0	0	0	0	0	0	0	0	0	0	0	0	0	0	5	0	0	0	0	0	0	0	0	0	0	0	0	Fungi; Ascomycota; Sordariomycetes; Xylariales; Xylariaceae; Xylaria; Xylaria_nigripes
New.ReferenceOTU535	0	0	0	0	0	0	0	0	0	0	0	0	0	0	0	0	0	0	0	2	0	0	0	0	0	0	0	0	0	0	Fungi; Ascomycota; Sordariomycetes; Xylariales; Xylariaceae; Xylaria; Xylaria_nigripes
New.ReferenceOTU6913	0	1	0	0	0	0	0	0	0	0	0	0	0	0	0	0	0	0	0	0	0	0	0	1	0	0	0	0	0	0	Fungi; Ascomycota; Sordariomycetes; Xylariales; Xylariaceae; Xylaria; Xylaria_nigripes

SH092339.06FU_JQ979095_reps	0	0	0	0	0	0	0	0	1	0	0	0	0	0	0	0	0	2	0	1	0	0	0	0	0	0	0	0	1	0	Fungi; Ascomycota; Sordariomycetes; Xylariales; Xylariaceae; Xylaria; Xylaria_nigripes
New.ReferenceOTU110	0	0	0	0	0	0	0	0	4	0	0	0	0	0	0	1	0	0	0	0	0	0	2	0	0	0	3	2	0	0	Fungi; Ascomycota; Sordariomycetes; Xylariales; Xylariaceae; Xylaria; Xylaria_sp
New.ReferenceOTU1289	0	0	0	0	0	0	0	0	0	0	0	0	0	0	0	0	0	0	1	0	0	0	0	0	0	0	0	0	0	0	Fungi; Ascomycota; Sordariomycetes; Xylariales; Xylariaceae; Xylaria; Xylaria_sp
New.ReferenceOTU2553	0	0	0	0	0	0	0	0	0	0	0	0	0	0	0	0	0	0	1	0	0	0	0	0	0	0	0	0	0	0	Fungi; Ascomycota; Sordariomycetes; Xylariales; Xylariaceae; Xylaria; Xylaria_sp
New.ReferenceOTU3278	0	0	0	0	0	0	3	0	0	1	0	0	0	0	0	0	0	0	0	0	0	0	0	0	0	0	0	0	0	0	Fungi; Ascomycota; Sordariomycetes; Xylariales; Xylariaceae; Xylaria; Xylaria_sp
New.ReferenceOTU3465	0	0	0	0	0	0	0	0	1	0	0	0	0	0	0	0	0	0	0	0	0	0	0	0	0	0	0	0	0	0	Fungi; Ascomycota; Sordariomycetes; Xylariales; Xylariaceae; Xylaria; Xylaria_sp
New.ReferenceOTU3566	0	0	0	0	0	0	1	0	0	0	0	0	0	0	0	0	0	0	0	0	0	0	0	0	0	0	0	0	0	0	Fungi; Ascomycota; Sordariomycetes; Xylariales; Xylariaceae; Xylaria; Xylaria_sp
New.ReferenceOTU4935	0	0	0	0	0	0	0	0	0	0	0	0	1	0	0	0	0	0	0	0	0	0	0	0	0	0	0	0	0	0	Fungi; Ascomycota; Sordariomycetes; Xylariales; Xylariaceae; Xylaria; Xylaria_sp
New.ReferenceOTU593	0	0	0	0	0	0	0	0	0	0	2	0	0	0	0	0	0	0	0	0	0	0	0	0	0	0	0	0	0	0	Fungi; Ascomycota; Sordariomycetes; Xylariales; Xylariaceae; Xylaria; Xylaria_sp
SH089404.06FU_FM200654_reps	0	0	0	0	0	0	0	0	0	1	0	0	0	0	0	0	0	0	0	0	0	0	0	0	0	0	0	0	0	0	Fungi; Ascomycota; Sordariomycetes; Xylariales; Xylariaceae; Xylaria; Xylaria_sp
SH089406.06FU_FJ175174_reps	0	0	0	0	0	0	0	0	0	1	0	0	0	0	0	0	0	0	0	0	0	0	0	0	0	0	0	0	0	0	Fungi; Ascomycota; Sordariomycetes; Xylariales; Xylariaceae; Xylaria; Xylaria_sp
SH117320.06FU_DQ480355_reps	0	0	0	0	0	0	0	0	0	0	1	0	0	0	0	0	0	0	0	0	0	0	0	0	0	0	0	0	0	0	Fungi; Ascomycota; Sordariomycetes; Xylariales; Xylariaceae; Xylaria; Xylaria_sp
SH146376.06FU_JQ760150_reps	0	0	0	0	0	0	0	0	0	0	0	0	0	0	0	0	0	0	0	0	0	0	0	0	0	0	1	0	0	0	Fungi; Ascomycota; Sordariomycetes; Xylariales; Xylariaceae; Xylaria; Xylaria_sp
SH146378.06FU_FJ612800_reps	0	0	0	2	0	0	0	0	0	0	0	0	2	0	1	0	0	0	0	0	0	0	0	0	0	0	0	0	0	0	Fungi; Ascomycota; Sordariomycetes; Xylariales; Xylariaceae; Xylaria; Xylaria_sp
New.ReferenceOTU1480	0	0	0	0	0	0	0	0	0	0	0	0	0	0	0	0	0	0	0	0	0	0	0	1	0	0	0	0	0	0	Fungi; Ascomycota; Sordariomycetes; Xylariales; Xylariaceae; Xylaria; Xylaria_sp_070611CAA
New.ReferenceOTU5965	0	3	0	0	0	0	0	0	0	0	0	0	0	0	0	0	0	0	0	0	0	0	0	0	0	0	0	0	0	0	Fungi; Ascomycota; Sordariomycetes; Xylariales; Xylariaceae; Xylaria; Xylaria_sp_10b_amb
New.ReferenceOTU2433	0	0	0	0	0	0	0	0	1	0	0	0	0	0	0	0	0	0	0	0	0	0	0	0	0	0	0	0	0	0	Fungi; Ascomycota; Sordariomycetes; Xylariales; Xylariaceae; Xylaria; Xylaria_sp_9_GDS_2013
New.ReferenceOTU2999	0	0	0	0	0	0	0	0	0	0	0	0	0	0	0	0	0	0	1	0	0	0	0	0	0	0	0	0	0	0	Fungi; Ascomycota; Sordariomycetes; Xylariales; Xylariaceae; Xylaria; Xylaria_sp_9_GDS_2013
New.ReferenceOTU3182	0	0	0	0	0	0	0	0	0	0	0	0	0	0	0	0	0	0	0	0	0	0	0	0	0	0	0	0	0	1	Fungi; Ascomycota; Sordariomycetes; Xylariales; Xylariaceae; Xylaria; Xylaria_sp_AA01
SH002644.06FU_FJ612827_reps_singleton	0	0	0	0	0	0	0	0	0	0	1	0	0	0	0	0	0	0	0	0	0	0	0	0	0	0	0	0	0	0	Fungi; Ascomycota; Sordariomycetes; Xylariales; Xylariaceae; Xylaria; Xylaria_sp_AA01
SH089445.06FU_AB701348_reps	0	0	0	0	0	0	0	0	0	0	1	0	0	2	0	0	0	0	0	0	0	0	0	0	0	0	0	0	0	0	Fungi; Ascomycota; Sordariomycetes; Xylariales; Xylariaceae; Xylaria; Xylaria_sp_AA01
New.ReferenceOTU4751	0	0	0	0	0	0	2	0	0	0	0	0	0	0	0	0	0	0	0	0	0	0	0	0	0	0	0	0	0	0	Fungi; Ascomycota; Sordariomycetes; Xylariales; Xylariaceae; Xylaria; Xylaria_sp_ASMC3
New.ReferenceOTU6196	0	1	0	0	0	0	0	0	0	0	0	0	0	0	0	0	0	0	0	0	0	0	0	0	0	0	0	1	0	0	Fungi; Ascomycota; Sordariomycetes; Xylariales; Xylariaceae; Xylaria; Xylaria_sp_AX114
SH134031.06FU_KC507203_reps	0	0	0	0	0	0	0	0	0	0	0	0	0	1	0	0	0	0	0	0	0	0	0	1	0	0	0	0	0	0	Fungi; Ascomycota; Sordariomycetes; Xylariales; Xylariaceae; Xylaria; Xylaria_sp_AX114
SH089399.06FU_AB741609_reps	0	0	0	0	0	0	0	0	0	0	0	1	0	0	0	0	0	0	0	0	0	0	0	0	0	0	0	0	0	0	Fungi; Ascomycota; Sordariomycetes; Xylariales; Xylariaceae; Xylaria; Xylaria_sp_F_064_695
SH089424.06FU_KC354590_reps	0	0	0	0	0	0	1	0	0	0	0	0	0	0	0	0	0	0	0	0	0	0	0	0	0	0	0	0	0	0	Fungi; Ascomycota; Sordariomycetes; Xylariales; Xylariaceae; Xylaria; Xylaria_sp_F_064_695
New.ReferenceOTU1789	0	0	0	0	0	0	0	0	0	0	0	0	0	0	0	1	0	0	2	0	0	0	0	0	0	0	0	0	0	0	Fungi; Ascomycota; Sordariomycetes; Xylariales; Xylariaceae; Xylaria; Xylaria_sp_F_254_988
SH089432.06FU_JQ623492_reps	0	0	0	0	1	0	0	0	0	0	0	0	0	0	0	0	0	0	0	0	0	0	0	0	0	0	0	2	0	1	Fungi; Ascomycota; Sordariomycetes; Xylariales; Xylariaceae; Xylaria; Xylaria_sp_G30
New.ReferenceOTU4819	0	0	0	0	0	0	0	0	0	0	1	0	0	0	0	0	0	0	0	0	0	0	0	0	0	0	0	0	0	0	Fungi; Ascomycota; Sordariomycetes; Xylariales; Xylariaceae; Xylaria; Xylaria_sp_PUTA_1
New.ReferenceOTU3776	0	0	0	0	0	0	0	0	0	0	0	0	0	0	0	0	0	0	1	0	0	0	0	0	0	0	0	0	0	0	Fungi; Ascomycota; Sordariomycetes; Xylariales; Xylariaceae; Xylaria; Xylaria_sp_RS062
SH146388.06FU_AB524024_reps	0	0	1	0	0	0	0	3	0	0	0	0	0	0	0	0	0	0	0	0	0	0	0	0	0	0	0	0	0	0	Fungi; Ascomycota; Sordariomycetes; Xylariales; Xylariaceae; Xylaria; Xylaria_sp_TC041101
SH126319.06FU_JX523620_reps	0	0	0	0	0	0	0	0	0	0	0	0	0	0	0	0	0	0	0	0	0	0	0	0	0	0	1	0	0	0	Fungi; Ascomycota; Sordariomycetes; Xylariales; Xylariaceae; Xylaria; Xylaria_sp_WR1
New.CleanUp.ReferenceOTU34	0	0	0	0	0	0	0	0	1	0	0	0	0	0	0	0	0	0	0	0	0	0	0	0	0	0	0	0	0	0	Fungi; Ascomycota; Sordariomycetes; Xylariales; Xylariaceae; Xylaria; Xylaria_sp_XSCH17
New.ReferenceOTU3098	0	0	0	0	0	0	0	0	0	0	0	0	0	0	0	0	0	0	0	0	0	0	0	0	1	0	0	0	0	0	Fungi; Ascomycota; Sordariomycetes; Xylariales; Xylariaceae; Xylaria; Xylaria_sp_XSCH17
SH124316.06FU_AB073533_reps	6	0	0	0	0	0	1	0	0	0	0	0	0	0	0	0	1	0	0	0	0	0	0	0	0	0	0	0	0	0	Fungi; Ascomycota; Sordariomycetes; Xylariales; Xylariaceae; Xylaria; Xylaria_sp_ZJ19_B
New.ReferenceOTU2840	0	0	0	0	1	0	1	0	0	0	0	0	0	0	0	0	1	0	0	0	0	0	0	0	0	0	0	0	0	0	Fungi; Basidiomycota; Agaricomycetes; Agaricales; Agaricaceae; Calvatia; Calvatia_gigantea
New.ReferenceOTU3793	2	0	0	0	0	0	0	0	0	0	0	0	0	0	0	0	0	0	0	0	0	0	0	0	0	0	0	0	0	0	Fungi; Basidiomycota; Agaricomycetes; Agaricales; Agaricaceae; Cystolepiota; Cystolepiota_seminuda
New.ReferenceOTU5972	2	0	0	0	0	0	0	0	0	0	0	0	0	0	0	0	0	0	0	0	0	0	0	0	0	0	0	0	0	0	Fungi; Basidiomycota; Agaricomycetes; Agaricales; Agaricaceae; Cystolepiota; Cystolepiota_sp
New.ReferenceOTU4686	9	0	0	0	0	0	0	0	0	0	0	0	0	0	0	0	0	0	0	0	0	0	0	0	0	0	0	0	0	0	Fungi; Basidiomycota; Agaricomycetes; Agaricales; Agaricaceae; Lepiota; Lepiota_magnispora
SH120730.06FU_UDB015103_reps	0	0	0	0	0	0	0	0	0	0	0	0	0	0	0	0	0	0	0	0	0	0	0	0	0	0	0	1	0	0	Fungi; Basidiomycota; Agaricomycetes; Agaricales; Agaricaceae; Lepiota; Lepiota_sp
New.ReferenceOTU5848	0	0	0	0	0	0	0	0	0	0	0	0	0	0	0	0	0	0	0	0	0	1	0	0	0	0	0	0	0	0	Fungi; Basidiomycota; Agaricomycetes; Agaricales; Agaricaceae; Lepiota; Lepiota_sp_MFLU_09_0142
New.ReferenceOTU3161	0	0	0	0	0	0	0	0	0	0	0	0	0	0	0	0	0	0	1	0	0	0	0	0	0	0	0	0	0	0	Fungi; Basidiomycota; Agaricomycetes; Agaricales; Agaricaceae; Lycoperdon; Lycoperdon_norvegicum
New.ReferenceOTU2635	0	0	0	0	0	0	0	0	0	0	0	0	0	0	0	0	0	0	0	0	0	0	1	0	1	0	0	0	0	0	Fungi; Basidiomycota; Agaricomycetes; Agaricales; Agaricaceae; Lycoperdon; Lycoperdon_sp_CMU55_Ly1
New.ReferenceOTU5232	0	0	0	0	0	0	0	0	0	0	0	0	0	0	0	0	0	0	0	0	0	0	2	0	0	0	0	0	0	0	Fungi; Basidiomycota; Agaricomycetes; Agaricales; Agaricaceae; Lycoperdon; Lycoperdon_sp_CMU55_Ly1
New.ReferenceOTU5579	0	0	0	0	0	0	1	0	0	0	0	0	0	0	0	0	0	0	0	0	0	0	0	0	0	0	4	0	0	0	Fungi; Basidiomycota; Agaricomycetes; Agaricales; Agaricaceae; Lycoperdon; Lycoperdon_sp_CMU55_Ly1
New.ReferenceOTU5477	0	0	0	0	1	0	1	0	0	0	0	0	0	0	0	0	0	0	0	0	0	0	0	0	0	0	0	0	0	0	Fungi; Basidiomycota; Agaricomycetes; Agaricales; Agaricaceae; Lycoperdon; Lycoperdon_subcretaceum
New.ReferenceOTU2795	0	0	0	0	0	0	0	0	0	0	0	0	0	0	0	0	0	0	0	0	0	0	0	0	2	0	0	0	0	0	Fungi; Basidiomycota; Agaricomycetes; Agaricales; Agaricaceae; Melanophyllum; Melanophyllum_haematospermum
New.ReferenceOTU5311	0	0	0	0	0	0	0	0	0	0	0	0	0	0	0	0	0	0	0	0	0	1	0	0	0	0	0	0	0	0	Fungi; Basidiomycota; Agaricomycetes; Agaricales; Agaricaceae; Melanophyllum; Melanophyllum_haematospermum
New.ReferenceOTU5653	0	0	0	0	0	0	0	0	0	0	0	0	0	0	0	0	0	0	0	0	0	0	0	0	0	0	0	0	3	0	Fungi; Basidiomycota; Agaricomycetes; Agaricales; Agaricaceae; Micropsalliota; Micropsalliota_furfuracea
New.ReferenceOTU6829	0	0	0	0	0	0	0	0	0	0	0	0	0	0	0	0	0	0	0	1	0	0	0	0	0	0	0	0	10	0	Fungi; Basidiomycota; Agaricomycetes; Agaricales; Agaricaceae; Micropsalliota; Micropsalliota_subarginea
SH088986.06FU_HM436610_reps	0	0	0	0	0	0	0	0	0	0	0	0	0	0	0	0	0	0	0	1	0	0	0	0	0	0	0	0	1	0	Fungi; Basidiomycota; Agaricomycetes; Agaricales; Agaricaceae; Micropsalliota; Micropsalliota_subarginea
New.ReferenceOTU4305	0	0	0	0	0	0	1	0	0	0	0	0	0	0	0	0	0	0	0	0	0	0	0	0	0	0	0	0	0	0	Fungi; Basidiomycota; Agaricomycetes; Agaricales; Agaricaceae; Tulostoma; Tulostoma_xerophilum
New.ReferenceOTU2868	0	0	0	0	0	0	0	0	0	0	0	0	0	0	0	0	0	0	0	0	0	0	0	0	0	0	0	1	0	0	Fungi; Basidiomycota; Agaricomycetes; Agaricales; Bolbitiaceae; Galerella; Galerella_nigeriensis
New.ReferenceOTU6630	0	0	0	0	0	0	0	0	0	0	0	0	0	0	8	0	0	0	0	0	0	0	1	0	2	0	0	0	0	0	Fungi; Basidiomycota; Agaricomycetes; Agaricales; Clavariaceae; Clavaria; Clavaria_atrofusca
New.ReferenceOTU6992	0	0	0	0	0	0	0	0	0	0	0	0	0	0	0	0	0	0	0	1	0	0	0	0	0	0	0	0	0	0	Fungi; Basidiomycota; Agaricomycetes; Agaricales; Clavariaceae; Clavulinopsis; Clavulinopsis_sp
New.ReferenceOTU3559	0	0	0	0	0	0	0	0	0	0	0	0	0	0	0	0	0	0	0	0	0	0	1	0	0	0	0	0	0	0	Fungi; Basidiomycota; Agaricomycetes; Agaricales; Entolomataceae; Clitopilus; Clitopilus_sp
New.ReferenceOTU5754	0	1	0	0	0	0	0	0	0	0	0	0	0	0	0	0	0	0	0	0	0	0	0	0	0	0	0	0	0	0	Fungi; Basidiomycota; Agaricomycetes; Agaricales; Entolomataceae; Clitopilus; Clitopilus_sp
New.ReferenceOTU5964	0	0	0	0	0	0	0	0	0	0	0	0	0	0	0	0	0	0	0	0	0	0	0	0	1	0	0	0	0	0	Fungi; Basidiomycota; Agaricomycetes; Agaricales; Entolomataceae; Clitopilus; Clitopilus_sp
New.ReferenceOTU7413	2	0	0	0	0	0	0	0	0	0	0	0	0	0	0	0	0	0	0	0	0	0	0	0	0	0	0	0	0	0	Fungi; Basidiomycota; Agaricomycetes; Agaricales; Entolomataceae; Clitopilus; Clitopilus_sp
New.ReferenceOTU7504	0	0	0	0	1	0	0	0	0	0	0	0	0	0	0	0	0	0	0	0	0	0	0	0	0	0	0	0	0	0	Fungi; Basidiomycota; Agaricomycetes; Agaricales; Entolomataceae; Clitopilus; Clitopilus_sp
SH010907.06FU_GQ851895_reps_singleton	1	0	0	0	0	0	0	0	0	0	0	0	0	0	0	0	0	0	0	0	0	0	0	0	0	0	0	0	0	0	Fungi; Basidiomycota; Agaricomycetes; Agaricales; Entolomataceae; Clitopilus; Clitopilus_sp
New.ReferenceOTU3871	0	0	0	0	0	0	0	0	1	0	1	0	0	0	0	0	0	0	0	0	0	0	0	0	0	0	0	0	0	0	Fungi; Basidiomycota; Agaricomycetes; Agaricales; Entolomataceae; Clitopilus; Clitopilus_sp_1_T_778
New.ReferenceOTU7478	4	0	0	0	0	0	0	0	0	0	0	0	0	0	0	0	0	0	0	0	0	0	0	0	0	0	0	0	0	0	Fungi; Basidiomycota; Agaricomycetes; Agaricales; Entolomataceae; Leptonia; Leptonia_occidentalis
New.ReferenceOTU4096	0	0	0	0	0	0	0	0	0	0	0	0	0	0	0	0	0	0	0	0	0	1	0	0	0	0	0	0	0	0	Fungi; Basidiomycota; Agaricomycetes; Agaricales; Entolomataceae; Nolanea; Nolanea_strictior
New.ReferenceOTU6234	0	0	0	0	0	0	0	0	0	0	0	0	0	0	2	0	0	0	0	0	2	3	0	0	0	0	0	0	0	0	Fungi; Basidiomycota; Agaricomycetes; Agaricales; Incertae_sedis; Panaeolus; Panaeolus_fimicola
New.ReferenceOTU1683	0	0	0	0	0	0	0	0	0	0	0	0	0	0	0	0	0	0	0	0	0	0	0	0	0	0	0	3	0	0	Fungi; Basidiomycota; Agaricomycetes; Agaricales; Inocybaceae; Tubaria; Tubaria_confragosa
New.ReferenceOTU6161	0	0	0	0	0	0	0	0	0	0	0	0	0	0	0	0	0	0	0	0	0	2	0	0	0	0	0	0	0	0	Fungi; Basidiomycota; Agaricomycetes; Agaricales; Marasmiaceae; Amyloflagellula; Amyloflagellula_inflata
New.ReferenceOTU3326	1	0	0	0	0	0	0	0	0	0	0	0	0	0	0	0	0	0	0	0	0	0	0	0	0	0	0	0	0	0	Fungi; Basidiomycota; Agaricomycetes; Agaricales; Marasmiaceae; Marasmius; Marasmius_sp
New.ReferenceOTU2589	0	0	0	1	0	0	0	0	0	0	0	0	0	0	0	0	0	0	0	0	3	0	0	3	0	0	0	0	0	0	Fungi; Basidiomycota; Agaricomycetes; Agaricales; Marasmiaceae; Rectipilus; Rectipilus_natalensis
New.ReferenceOTU4065	0	0	0	0	0	0	0	0	1	0	0	0	0	0	0	0	0	0	0	0	0	0	0	0	0	0	0	0	0	0	Fungi; Basidiomycota; Agaricomycetes; Agaricales; Marasmiaceae; Rectipilus; Rectipilus_natalensis
New.ReferenceOTU4314	0	0	1	1	0	1	0	0	0	0	0	0	0	0	0	0	0	0	0	0	0	0	0	0	0	0	0	1	0	0	Fungi; Basidiomycota; Agaricomycetes; Agaricales; Marasmiaceae; Rectipilus; Rectipilus_natalensis
New.ReferenceOTU6875	0	0	0	0	0	0	0	0	2	0	0	0	0	0	0	0	0	0	0	0	0	0	0	0	0	0	0	0	0	0	Fungi; Basidiomycota; Agaricomycetes; Agaricales; Marasmiaceae; Rectipilus; Rectipilus_natalensis
New.ReferenceOTU7638	1	0	0	0	0	0	0	0	0	0	0	0	0	0	0	0	0	0	0	0	0	0	0	0	0	0	0	0	0	0	Fungi; Basidiomycota; Agaricomycetes; Agaricales; Marasmiaceae; Rectipilus; Rectipilus_natalensis
New.ReferenceOTU807	0	0	0	4	0	0	0	0	0	0	0	0	0	0	0	0	0	0	0	0	0	0	0	0	0	0	0	0	0	0	Fungi; Basidiomycota; Agaricomycetes; Agaricales; Marasmiaceae; Rectipilus; Rectipilus_natalensis
New.ReferenceOTU5020	0	0	0	0	0	0	0	0	0	0	0	0	0	0	1	0	0	0	0	0	0	0	0	0	0	0	0	0	0	0	Fungi; Basidiomycota; Agaricomycetes; Agaricales; Mycenaceae; Hemimycena; Hemimycena_cucullata
New.ReferenceOTU2354	0	0	0	0	0	0	0	0	0	0	1	0	0	0	0	0	0	0	0	0	0	0	0	0	0	0	0	0	0	0	Fungi; Basidiomycota; Agaricomycetes; Agaricales; Mycenaceae; Mycena; Mycena_galopus
New.ReferenceOTU7243	0	0	0	0	0	0	0	0	0	0	0	0	0	0	0	0	0	0	0	1	0	0	0	0	0	0	0	0	0	0	Fungi; Basidiomycota; Agaricomycetes; Agaricales; Mycenaceae; Mycena; Mycena_pura
New.ReferenceOTU961	0	0	0	0	0	0	0	0	0	0	0	0	0	0	0	0	0	0	1	0	0	0	0	0	0	0	0	0	0	0	Fungi; Basidiomycota; Agaricomycetes; Agaricales; Mycenaceae; Mycena; Mycena_pura
New.ReferenceOTU3324	3	0	0	0	0	0	0	0	0	0	0	0	0	0	0	0	0	0	0	0	0	0	0	0	0	0	0	0	0	0	Fungi; Basidiomycota; Agaricomycetes; Agaricales; Mycenaceae; Mycena; Mycena_sp
New.ReferenceOTU5603	0	0	0	0	0	0	0	0	0	0	0	0	0	0	0	0	0	0	0	0	0	0	1	0	0	0	0	0	0	0	Fungi; Basidiomycota; Agaricomycetes; Agaricales; Pluteaceae; Pluteus; Pluteus_heteromarginatus
New.ReferenceOTU1495	0	0	0	0	0	0	0	0	0	0	1	0	0	0	0	0	0	0	0	0	0	0	0	0	0	1	0	0	0	0	Fungi; Basidiomycota; Agaricomycetes; Agaricales; Pluteaceae; Pluteus; Pluteus_losulus
New.ReferenceOTU827	0	0	1	0	0	0	0	0	0	0	0	0	0	0	0	0	0	0	0	0	0	0	0	0	0	0	0	0	0	0	Fungi; Basidiomycota; Agaricomycetes; Agaricales; Pluteaceae; Pluteus; Pluteus_roseipes
New.ReferenceOTU3198	0	0	0	0	0	0	0	0	0	0	0	0	0	0	0	0	0	0	0	0	0	0	0	0	0	0	0	0	0	1	Fungi; Basidiomycota; Agaricomycetes; Agaricales; Pluteaceae; Pluteus; Pluteus_seticeps
New.ReferenceOTU2430	0	0	0	0	0	0	0	0	0	0	0	0	0	0	0	0	0	0	0	1	0	0	0	0	0	0	0	0	0	0	Fungi; Basidiomycota; Agaricomycetes; Agaricales; Pluteaceae; Pluteus; Pluteus_xylophilus
New.ReferenceOTU5298	0	0	0	0	0	0	0	0	0	0	0	0	0	0	1	0	0	0	0	0	0	0	0	0	0	0	0	0	0	0	Fungi; Basidiomycota; Agaricomycetes; Agaricales; Psathyrellaceae; Coprinopsis; Coprinopsis_cinerea
New.ReferenceOTU6681	1	0	0	0	0	0	1	0	0	0	0	0	0	0	0	0	0	0	0	0	0	0	0	0	0	0	0	0	0	0	Fungi; Basidiomycota; Agaricomycetes; Agaricales; Psathyrellaceae; Coprinopsis; Coprinopsis_cinerea
New.ReferenceOTU1397	0	0	0	0	0	0	0	0	0	0	0	0	0	0	0	0	0	0	0	0	0	1	0	0	0	0	0	0	0	0	Fungi; Basidiomycota; Agaricomycetes; Agaricales; Psathyrellaceae; Psathyrella; Psathyrella_candolleana
New.ReferenceOTU6157	3	0	0	0	0	0	0	0	0	0	0	0	0	0	0	0	0	0	0	0	0	0	0	0	0	0	0	0	0	0	Fungi; Basidiomycota; Agaricomycetes; Agaricales; Psathyrellaceae; Psathyrella; Psathyrella_pygmaea
New.ReferenceOTU2706	3	0	0	0	0	0	0	0	0	0	0	0	0	0	0	0	0	0	0	0	0	0	0	0	0	0	0	0	0	0	Fungi; Basidiomycota; Agaricomycetes; Agaricales; Psathyrellaceae; Psathyrella; Psathyrella_sp
New.ReferenceOTU5924	0	0	0	0	0	0	0	0	0	0	0	0	0	0	0	0	0	0	0	0	1	0	0	0	0	0	0	0	0	0	Fungi; Basidiomycota; Agaricomycetes; Agaricales; Psathyrellaceae; Psathyrella; Psathyrella_uskensis
New.ReferenceOTU2885	0	0	0	0	0	0	0	0	0	0	0	0	0	0	0	0	0	0	0	0	0	0	0	0	0	0	0	0	0	1	Fungi; Basidiomycota; Agaricomycetes; Agaricales; Strophariaceae; Gymnopilus; Gymnopilus_dilepis
New.ReferenceOTU465	0	0	0	0	0	0	0	0	0	0	0	0	0	0	0	0	0	0	1	0	0	0	0	0	0	0	0	0	0	0	Fungi; Basidiomycota; Agaricomycetes; Agaricales; Strophariaceae; Gymnopilus; Gymnopilus_dilepis
New.ReferenceOTU5362	0	0	0	0	0	0	9	2	0	0	0	0	0	0	0	0	0	0	0	0	3	1	0	0	0	0	0	0	0	0	Fungi; Basidiomycota; Agaricomycetes; Agaricales; Strophariaceae; Gymnopilus; Gymnopilus_purpureosquamulosus
SH006966.06FU_EU401713_reps_singleton	1	0	0	0	0	1	10	0	0	1	0	0	0	0	1	0	0	0	0	0	0	2	0	0	0	0	0	0	0	0	Fungi; Basidiomycota; Agaricomycetes; Agaricales; Strophariaceae; Gymnopilus; Gymnopilus_purpureosquamulosus
SH096934.06FU_AY280975_reps	0	0	0	0	0	0	2	0	0	0	0	0	0	0	0	0	0	0	0	0	0	0	0	0	0	0	0	0	0	0	Fungi; Basidiomycota; Agaricomycetes; Agaricales; Strophariaceae; Gymnopilus; Gymnopilus_purpureosquamulosus
SH096939.06FU_AY280979_reps	1	0	0	0	0	3	4	0	0	0	0	0	0	0	1	0	0	0	0	0	0	0	0	0	0	0	0	8	0	0	Fungi; Basidiomycota; Agaricomycetes; Agaricales; Strophariaceae; Gymnopilus; Gymnopilus_purpureosquamulosus
New.ReferenceOTU6494	0	0	0	0	0	0	0	0	0	4	0	0	0	0	0	0	0	0	0	0	0	0	0	0	0	0	0	0	0	0	Fungi; Basidiomycota; Agaricomycetes; Agaricales; Strophariaceae; Gymnopilus; Gymnopilus_sp_Spooner_290
New.ReferenceOTU2901	0	0	0	0	0	0	0	0	2	0	0	0	0	0	0	0	0	0	0	0	0	0	0	0	0	0	0	0	0	0	Fungi; Basidiomycota; Agaricomycetes; Agaricales; Strophariaceae; Psilocybe; Psilocybe_phyllogena
New.ReferenceOTU4930	0	0	1	0	0	0	0	0	0	0	0	0	0	0	0	0	0	0	0	0	0	0	0	0	0	0	0	0	0	1	Fungi; Basidiomycota; Agaricomycetes; Agaricales; Strophariaceae; Psilocybe; Psilocybe_phyllogena
SH120230.06FU_AB512337_reps	0	0	0	0	0	0	0	0	3	0	0	0	0	0	0	0	0	0	0	0	0	0	0	0	0	0	0	0	0	0	Fungi; Basidiomycota; Agaricomycetes; Agaricales; Strophariaceae; Psilocybe; Psilocybe_phyllogena

New.ReferenceOTU5661	0	0	0	0	0	0	0	0	0	0	0	0	0	0	0	0	0	0	0	1	0	0	0	0	0	0	0	0	0	0	Fungi; Basidiomycota; Agaricomycetes; Agaricales; Strophariaceae; Psilocybe; Psilocybe_sp_TENN61695
New.ReferenceOTU1485	0	0	0	0	1	0	1	0	0	0	0	0	0	0	0	0	0	0	0	0	0	0	0	1	0	0	0	0	0	0	Fungi; Basidiomycota; Agaricomycetes; Agaricales; Tricholomataceae; Cantharellula; Cantharellula_foetida
New.ReferenceOTU2667	13	0	0	0	0	0	0	0	0	0	0	0	0	0	0	0	0	0	0	0	0	0	0	0	0	0	0	0	0	0	Fungi; Basidiomycota; Agaricomycetes; Agaricales; Tricholomataceae; Cantharellula; Cantharellula_humicola
New.ReferenceOTU1402	0	0	0	0	0	0	0	0	0	0	0	0	0	0	0	0	0	0	0	0	0	0	0	2	0	0	0	0	0	0	Fungi; Basidiomycota; Agaricomycetes; Boletales; Coniophoraceae; Coniophora; Coniophora_arida
New.ReferenceOTU2608	1	0	0	0	0	0	1	1	0	0	0	0	0	0	0	0	0	0	0	0	0	0	1	0	0	0	0	0	0	0	Fungi; Basidiomycota; Agaricomycetes; Boletales; Coniophoraceae; Coniophora; Coniophora_olivacea
New.ReferenceOTU1807	1	0	0	0	0	0	0	0	0	0	0	0	0	0	0	0	0	0	0	0	0	0	0	0	0	0	0	0	0	0	Fungi; Basidiomycota; Agaricomycetes; Boletales; Coniophoraceae; unidentified; cf_Coniophora_sp_1_AJMH_2010
New.ReferenceOTU1445	0	0	0	0	0	0	0	0	0	0	0	0	0	0	0	0	0	0	1	0	0	0	0	0	0	0	0	0	0	0	Fungi; Basidiomycota; Agaricomycetes; Geastrales; Geastraceae; Geastrum; Geastrum_pectinatum
New.ReferenceOTU5942	0	0	0	0	0	0	0	0	0	0	0	0	0	0	0	0	0	1	0	0	0	0	0	0	0	0	0	0	0	0	Fungi; Basidiomycota; Agaricomycetes; Geastrales; Geastraceae; Geastrum; Geastrum_pectinatum
New.ReferenceOTU2962	0	0	0	0	0	0	0	0	5	0	0	0	0	2	3	0	0	0	0	0	0	0	0	0	0	0	0	0	0	0	Fungi; Basidiomycota; Agaricomycetes; Geastrales; Geastraceae; Geastrum; Geastrum_sp_TNS_KH_AUS10_72
New.ReferenceOTU409	0	0	0	0	0	0	0	4	0	1	7	0	0	0	1	1	0	0	0	0	0	0	0	2	0	0	0	0	0	0	Fungi; Basidiomycota; Agaricomycetes; Geastrales; Geastraceae; Geastrum; Geastrum_sp_TNS_KH_JPN09_203
New.ReferenceOTU4172	0	0	0	0	0	0	0	0	1	0	0	0	0	0	0	0	0	0	0	0	0	0	0	0	0	0	0	0	0	0	Fungi; Basidiomycota; Agaricomycetes; Geastrales; Geastraceae; Geastrum; Geastrum_sp_TNS_KH_JPN09_203
SH147322.06FU_JN845181_reps	0	1	0	0	0	0	0	6	0	0	5	0	0	0	0	0	0	0	0	0	0	0	0	2	0	0	0	0	0	0	Fungi; Basidiomycota; Agaricomycetes; Geastrales; Geastraceae; Geastrum; Geastrum_sp_TNS_KH_JPN09_203
New.ReferenceOTU4517	0	0	0	0	0	0	0	0	0	0	0	0	0	0	0	0	0	0	0	0	0	0	0	0	0	0	0	0	0	1	Fungi; Basidiomycota; Agaricomycetes; Geastrales; Geastraceae; Geastrum; Geastrum_sp_TNS_KH_JPN10_693
New.ReferenceOTU7404	0	0	0	0	0	0	0	0	0	0	0	0	0	0	0	0	0	0	0	0	0	1	0	0	0	0	0	0	0	0	Fungi; Basidiomycota; Agaricomycetes; Geastrales; Geastraceae; Geastrum; Geastrum_sp_TNS_KH_JPN10_693
SH027020.06FU_JN845186_reps_singleton	0	0	0	0	0	0	0	0	0	0	0	0	0	1	0	0	0	0	0	0	0	0	0	0	0	0	0	0	0	0	Fungi; Basidiomycota; Agaricomycetes; Geastrales; Geastraceae; Geastrum; Geastrum_sp_TNS_KH_JPN10_693
New.ReferenceOTU6136	0	0	0	0	0	0	0	0	6	0	0	0	0	0	0	0	0	0	0	0	0	0	0	0	0	0	0	0	0	0	Fungi; Basidiomycota; Agaricomycetes; Geastrales; Geastraceae; Geastrum; Geastrum_sp_TNS_KH_JPN10_710
SH134556.06FU_JN845187_reps	0	0	0	0	0	0	0	0	0	0	0	0	0	0	0	0	0	0	0	0	0	0	0	0	0	0	1	0	0	0	Fungi; Basidiomycota; Agaricomycetes; Geastrales; Geastraceae; Geastrum; Geastrum_sp_TNS_KH_JPN10_710
New.ReferenceOTU2289	0	0	0	0	2	0	0	0	0	0	0	0	0	0	0	0	0	0	0	0	0	0	0	0	0	0	0	0	0	0	Fungi; Basidiomycota; Agaricomycetes; Geastrales; Geastraceae; Geastrum; Geastrum_sp_TNS_KH_JPN10_731
SH134923.06FU_JN845191_reps	0	0	0	1	0	0	0	0	0	0	0	0	0	0	0	0	0	0	0	0	0	0	0	0	0	0	0	0	0	0	Fungi; Basidiomycota; Agaricomycetes; Geastrales; Geastraceae; Geastrum; Geastrum_sp_TNS_KH_JPN10_747
New.ReferenceOTU2910	0	0	0	0	0	0	0	0	2	0	0	0	0	0	0	0	0	0	0	0	0	0	0	0	0	0	0	0	0	0	Fungi; Basidiomycota; Agaricomycetes; Geastrales; Geastraceae; Geastrum; Geastrum_sp_TNS_KH_JPN10_748
SH130248.06FU_JN845192_reps	0	2	1	0	0	0	0	0	1	0	0	0	0	0	0	0	1	0	1	0	0	1	0	1	0	0	0	0	1	0	Fungi; Basidiomycota; Agaricomycetes; Geastrales; Geastraceae; Geastrum; Geastrum_sp_TNS_KH_JPN10_748
SH038670.06FU_JN845202_reps_singleton	0	0	0	0	0	0	0	0	2	0	0	0	0	0	0	0	0	0	0	0	0	0	0	0	0	0	0	0	0	0	Fungi; Basidiomycota; Agaricomycetes; Geastrales; Geastraceae; Geastrum; Geastrum_sp_TNS_KH_TH09_108
New.ReferenceOTU292	0	0	0	0	0	0	0	0	0	0	0	0	0	0	0	0	0	0	0	1	0	0	0	0	0	0	0	0	0	0	Fungi; Basidiomycota; Agaricomycetes; Geastrales; Geastraceae; Geastrum; Geastrum_sp_TNS_Sakamoto154
New.ReferenceOTU72	0	0	0	0	0	0	0	0	0	0	0	0	0	0	0	0	0	0	0	2	0	0	0	0	0	0	0	0	0	0	Fungi; Basidiomycota; Agaricomycetes; Geastrales; Geastraceae; Geastrum; Geastrum_sp_TNS_Sakamoto154
New.ReferenceOTU6076	0	0	0	0	4	0	0	0	0	0	0	0	0	0	0	0	0	0	0	0	0	0	0	0	0	0	0	0	0	0	Fungi; Basidiomycota; Agaricomycetes; Geastrales; Geastraceae; Geastrum; Geastrum_sp_TNS_Sakamoto207
New.ReferenceOTU1073	0	0	0	0	0	0	0	0	0	0	0	0	0	0	0	0	0	0	2	0	0	0	0	0	0	0	0	0	0	0	Fungi; Basidiomycota; Agaricomycetes; Geastrales; Geastraceae; Geastrum; Geastrum_triplex
New.ReferenceOTU2236	0	0	0	0	0	0	0	0	0	0	0	0	0	0	0	0	0	0	0	2	0	0	0	0	0	0	0	0	0	0	Fungi; Basidiomycota; Agaricomycetes; Geastrales; Geastraceae; Geastrum; Geastrum_triplex
New.ReferenceOTU6005	0	0	0	0	0	0	0	0	0	0	0	0	0	0	0	0	0	0	0	0	0	0	0	13	0	0	0	0	0	0	Fungi; Basidiomycota; Agaricomycetes; Geastrales; Geastraceae; Geastrum; Geastrum_triplex
New.ReferenceOTU6823	0	0	0	0	0	0	0	0	0	0	0	0	0	0	0	0	0	0	1	0	0	0	0	0	0	0	0	0	0	0	Fungi; Basidiomycota; Agaricomycetes; Geastrales; Geastraceae; Geastrum; Geastrum_triplex
New.ReferenceOTU869	3	0	0	0	0	0	0	0	0	0	0	0	0	2	0	0	0	0	0	0	0	0	0	1	0	0	0	0	1	0	Fungi; Basidiomycota; Agaricomycetes; Geastrales; Geastraceae; Radiigera; Radiigera_sp_1_RG_2012
New.ReferenceOTU6893	0	0	0	0	0	0	0	0	0	0	1	0	0	0	0	0	0	0	0	1	0	0	0	0	0	0	0	0	0	0	Fungi; Basidiomycota; Agaricomycetes; Geastrales; Geastraceae; Schenella; Schenella_simplex
New.ReferenceOTU494	0	0	0	0	0	0	0	0	0	0	0	0	0	0	2	0	0	0	0	0	0	0	0	0	0	0	0	0	1	0	Fungi; Basidiomycota; Agaricomycetes; Hymenochaetales; Hymenochaetaceae; Fomitiporia; Fomitiporia_polymorpha
New.ReferenceOTU2267	0	0	1	0	0	0	0	0	0	0	0	0	0	0	0	0	0	0	0	0	0	0	0	0	0	0	0	0	0	0	Fungi; Basidiomycota; Agaricomycetes; Hymenochaetales; Hymenochaetaceae; Fulvifomes; Fulvifomes_nilgheriensis
New.ReferenceOTU4803	0	0	14	0	2	0	0	0	0	0	0	0	0	0	0	0	1	0	0	0	0	0	0	0	0	0	0	0	0	0	Fungi; Basidiomycota; Agaricomycetes; Hymenochaetales; Hymenochaetaceae; Fulvifomes; Fulvifomes_nilgheriensis
New.ReferenceOTU7038	0	0	1	0	0	0	0	0	0	4	0	0	0	0	0	0	0	0	0	0	0	0	0	0	0	0	0	0	0	0	Fungi; Basidiomycota; Agaricomycetes; Hymenochaetales; Hymenochaetaceae; Fulvifomes; Fulvifomes_nilgheriensis
New.ReferenceOTU915	0	0	0	0	1	0	0	0	0	0	0	0	0	0	0	0	0	0	0	0	0	0	0	0	0	0	0	0	0	0	Fungi; Basidiomycota; Agaricomycetes; Hymenochaetales; Hymenochaetaceae; Hymenochaete; Hymenochaete_megaspora
New.ReferenceOTU2877	0	0	0	0	0	0	0	0	1	0	0	0	0	0	0	0	0	0	0	0	0	0	0	0	0	0	0	0	0	0	Fungi; Basidiomycota; Agaricomycetes; Hymenochaetales; Hymenochaetaceae; Inonotus; Inonotus_tropicalis
New.ReferenceOTU6390	0	0	0	0	0	0	0	0	4	0	0	0	0	0	0	0	0	0	0	0	0	0	0	0	0	0	0	0	0	0	Fungi; Basidiomycota; Agaricomycetes; Phallales; Phallaceae; Clathrus; Clathrus_ruber
New.ReferenceOTU761	0	0	5	0	0	0	0	0	0	0	0	0	0	0	2	4	0	0	0	0	0	1	0	1	1	0	3	0	0	10	Fungi; Basidiomycota; Agaricomycetes; Phallales; Phallaceae; Dictyophora; Dictyophora_echinovolvata
New.ReferenceOTU1269	0	0	0	0	0	0	0	0	0	0	1	0	0	0	0	0	0	0	1	2	0	0	0	0	0	0	0	0	0	0	Fungi; Basidiomycota; Agaricomycetes; Phallales; Phallaceae; Dictyophora; Dictyophora_indusiata
New.ReferenceOTU2836	0	0	1	0	0	0	0	0	0	0	0	0	0	0	0	0	0	0	0	0	0	0	0	0	0	0	2	0	0	0	Fungi; Basidiomycota; Agaricomycetes; Phallales; Phallaceae; Dictyophora; Dictyophora_indusiata
New.ReferenceOTU3315	0	0	0	0	0	0	0	0	0	0	1	0	0	0	0	0	0	0	0	0	0	0	0	0	0	0	0	0	0	0	Fungi; Basidiomycota; Agaricomycetes; Phallales; Phallaceae; Dictyophora; Dictyophora_indusiata
New.ReferenceOTU3774	0	0	1	0	0	0	0	0	0	0	0	0	0	0	0	0	0	0	0	0	4	0	3	0	0	0	0	5	0	0	Fungi; Basidiomycota; Agaricomycetes; Phallales; Phallaceae; Phallus; Phallus_hadriani
New.ReferenceOTU3036	0	0	1	0	0	0	0	0	0	0	0	0	0	0	0	0	0	0	0	0	0	0	0	0	0	0	0	0	1	1	Fungi; Basidiomycota; Agaricomycetes; Phallales; Phallaceae; Phallus; Phallus_sp_2_LL_2013
New.ReferenceOTU3922	1	0	3	0	1	0	0	0	1	1	1	2	0	0	0	0	1	2	0	1	0	0	0	22	0	5	0	0	11	6	Fungi; Basidiomycota; Agaricomycetes; Phallales; Phallaceae; Phallus; Phallus_sp_2_LL_2013
New.ReferenceOTU7775	0	0	0	0	0	0	0	0	0	0	0	0	0	0	0	0	0	1	0	0	0	0	0	2	0	0	0	0	0	0	Fungi; Basidiomycota; Agaricomycetes; Polyporales; Fomitopsidaceae; Antrodia; Antrodia_leucaena
New.ReferenceOTU5949	0	0	0	0	0	5	0	0	0	0	0	0	0	0	0	0	0	0	0	0	0	0	0	0	0	0	0	0	0	2	Fungi; Basidiomycota; Agaricomycetes; Polyporales; Fomitopsidaceae; Fibroporia; Fibroporia_gossypium
New.ReferenceOTU107	31	0	0	0	0	0	0	0	0	0	0	0	0	0	0	0	0	0	0	0	0	0	0	0	0	0	0	0	0	0	Fungi; Basidiomycota; Agaricomycetes; Polyporales; Fomitopsidaceae; Fomitopsis; Fomitopsis_dochmia
SH118111.06FU_JN411120_refs	0	0	0	0	0	0	0	0	1	0	0	0	0	0	0	0	0	0	0	0	0	0	0	0	0	0	0	0	0	0	Fungi; Basidiomycota; Agaricomycetes; Polyporales; Grammotheleaceae; Theleporus; Theleporus_membranaceus
New.ReferenceOTU1610	0	0	0	0	0	0	0	0	0	0	0	0	0	0	0	0	0	0	0	0	0	0	0	0	0	0	0	1	0	0	Fungi; Basidiomycota; Agaricomycetes; Polyporales; Meripilaceae; Physisporinus; Physisporinus_vitreus
New.ReferenceOTU1031	0	0	0	0	0	0	0	0	0	0	0	0	0	0	0	0	1	0	1	0	0	0	0	0	0	0	0	0	0	2	Fungi; Basidiomycota; Agaricomycetes; Polyporales; Meripilaceae; Rigidoporus; Rigidoporus_sp
SH118041.06FU_GQ999294_reps	1	0	0	0	0	0	0	0	0	0	2	0	0	0	0	0	0	0	0	0	0	0	0	0	0	0	0	0	0	0	Fungi; Basidiomycota; Agaricomycetes; Polyporales; Meripilaceae; Rigidoporus; Rigidoporus_sp
New.ReferenceOTU3158	7	0	0	0	0	0	0	0	0	0	0	0	0	0	0	0	0	0	0	0	0	0	0	0	0	0	0	0	0	0	Fungi; Basidiomycota; Agaricomycetes; Polyporales; Meruliaceae; Hyphoderma; Hyphoderma_puberum
New.ReferenceOTU4617	0	0	0	0	1	0	0	0	0	0	0	0	0	0	0	0	0	0	0	0	0	0	0	0	0	0	0	0	0	0	Fungi; Basidiomycota; Agaricomycetes; Polyporales; Meruliaceae; Mycoacia; Mycoacia_nothofagi_var._nothofagi
New.ReferenceOTU2325	3	0	0	0	0	0	0	0	0	0	0	0	0	0	0	0	0	0	0	0	0	0	0	0	0	0	0	0	0	0	Fungi; Basidiomycota; Agaricomycetes; Polyporales; Meruliaceae; Phlebia; Phlebia_sp
New.ReferenceOTU7598	0	0	0	0	0	0	0	0	0	0	0	1	0	0	0	0	0	0	0	0	0	0	0	0	0	0	0	0	0	0	Fungi; Basidiomycota; Agaricomycetes; Polyporales; Phanerochaetaceae; Ceriporia; Ceriporia_inflata
SH115531.06FU_AY219372_reps	0	0	0	0	0	0	0	0	0	0	0	1	0	0	0	0	0	0	0	0	0	0	0	0	0	0	0	0	0	0	Fungi; Basidiomycota; Agaricomycetes; Polyporales; Phanerochaetaceae; Ceriporia; Ceriporia_inflata
New.ReferenceOTU727	0	0	0	0	0	0	0	0	2	0	0	0	0	0	0	0	0	0	0	0	0	0	0	0	0	0	0	0	0	0	Fungi; Basidiomycota; Agaricomycetes; Polyporales; Phanerochaetaceae; Hyphodermella; Hyphodermella_sp
SH115528.06FU_FJ820651_reps	0	0	0	0	0	0	0	1	0	0	0	0	0	0	0	0	0	0	0	0	0	0	0	0	0	0	0	0	0	0	Fungi; Basidiomycota; Agaricomycetes; Polyporales; Phanerochaetaceae; Phanerochaete; Phanerochaete_australis
New.ReferenceOTU7806	0	0	0	2	0	0	0	0	0	0	0	0	0	0	0	0	0	0	0	0	0	0	0	0	0	0	0	0	0	0	Fungi; Basidiomycota; Agaricomycetes; Polyporales; Phanerochaetaceae; Phanerochaete; Phanerochaete_stereoides
New.ReferenceOTU1821	0	0	0	0	0	0	0	0	0	0	0	0	0	0	0	0	0	2	0	0	0	0	0	0	0	0	0	0	0	0	Fungi; Basidiomycota; Agaricomycetes; Polyporales; Polyporaceae; Dichomitus; Dichomitus_albidofuscus
New.ReferenceOTU5047	3	0	0	0	0	0	0	0	0	0	0	0	0	0	0	0	0	0	0	0	0	0	0	0	0	0	0	0	0	0	Fungi; Basidiomycota; Agaricomycetes; Polyporales; Polyporaceae; Diplomitoporus; Diplomitoporus_rimosus
New.ReferenceOTU3677	0	0	0	0	0	0	0	0	0	0	0	0	0	0	0	0	0	0	0	0	0	0	0	0	0	0	0	1	0	1	Fungi; Basidiomycota; Agaricomycetes; Polyporales; Polyporaceae; Epithele; Epithele_typhae
New.ReferenceOTU7960	0	0	0	0	0	0	0	0	0	0	0	0	0	0	0	0	0	0	0	0	0	0	0	1	0	0	0	0	0	0	Fungi; Basidiomycota; Agaricomycetes; Polyporales; Polyporaceae; Perenniporia; Perenniporia_martius
New.ReferenceOTU1344	0	0	0	2	0	0	0	0	0	0	0	0	0	0	0	0	0	0	0	0	0	0	0	0	0	0	0	0	0	0	Fungi; Basidiomycota; Agaricomycetes; Polyporales; Polyporaceae; Perenniporia; Perenniporia_truncatospora
New.ReferenceOTU1377	0	0	0	1	0	0	0	0	0	0	0	0	0	0	0	0	0	0	0	0	0	0	0	0	0	0	0	0	0	0	Fungi; Basidiomycota; Agaricomycetes; Polyporales; Polyporaceae; Perenniporia; Perenniporia_truncatospora
New.ReferenceOTU1428	0	0	0	0	0	0	0	0	0	0	0	0	0	0	0	0	0	0	0	0	0	0	0	3	0	3	0	0	0	0	Fungi; Basidiomycota; Agaricomycetes; Polyporales; Polyporaceae; Perenniporia; Perenniporia_truncatospora
New.ReferenceOTU1279	0	0	0	0	0	0	0	0	0	0	0	0	0	0	0	0	0	0	0	0	0	0	0	0	0	0	0	0	1	0	Fungi; Basidiomycota; Agaricomycetes; Polyporales; Polyporaceae; Tinctoporellus; Tinctoporellus_epimiltinus
SH118107.06FU_KF435681_reps	0	0	0	0	0	0	0	0	0	0	0	0	0	0	0	0	0	0	0	0	0	0	0	0	0	0	0	2	0	0	Fungi; Basidiomycota; Agaricomycetes; Polyporales; Polyporaceae; Tinctoporellus; Tinctoporellus_epimiltinus
New.ReferenceOTU3864	0	0	0	0	0	0	0	0	0	0	1	0	0	0	0	0	0	0	0	1	0	0	0	0	0	0	0	0	0	0	Fungi; Basidiomycota; Agaricomycetes; Polyporales; Xenasmataceae; Xenasmatella; Xenasmatella_sp
New.ReferenceOTU3540	0	0	0	0	0	0	0	0	0	0	0	0	0	0	0	0	0	3	0	0	0	0	0	0	0	0	0	0	0	0	Fungi; Basidiomycota; Agaricomycetes; Russulales; Lachnocladiaceae; Asterostroma; Asterostroma_cervicolor
New.ReferenceOTU5758	0	0	1	0	0	0	0	0	0	0	0	3	0	0	0	0	0	0	0	0	0	0	0	0	0	0	0	0	0	0	Fungi; Basidiomycota; Agaricomycetes; Russulales; Peniophoraceae; Peniophora; Peniophora_sp
New.ReferenceOTU891	2	0	0	0	0	0	0	0	0	0	0	0	0	0	0	0	0	0	0	0	0	0	0	0	0	0	0	0	0	0	Fungi; Basidiomycota; Agaricomycetes; Russulales; Stephanosporaceae; Lindtneria; Lindtneria_sp
New.ReferenceOTU132	1	0	2	0	0	0	0	0	0	0	0	0	0	0	0	1	0	0	0	0	0	2	0	0	0	0	0	2	0	0	Fungi; Basidiomycota; Agaricomycetes; Trechisporales; Hydnodontaceae; Trechispora; Trechispora_sp
New.ReferenceOTU3114	1	1	0	0	0	0	0	0	0	0	0	0	0	0	0	0	0	0	0	0	0	0	0	0	0	0	0	0	0	0	Fungi; Basidiomycota; Agaricomycetes; Trechisporales; Hydnodontaceae; Trechispora; Trechispora_sp
New.ReferenceOTU3304	0	0	0	0	0	0	0	0	0	0	0	0	0	0	0	0	0	0	1	0	0	0	0	0	0	0	0	0	0	0	Fungi; Basidiomycota; Agaricomycetes; Trechisporales; Hydnodontaceae; Trechispora; Trechispora_sp
New.ReferenceOTU4025	0	0	0	0	0	0	0	0	0	0	0	0	0	0	0	0	0	0	0	0	0	0	0	0	0	0	0	0	2	0	Fungi; Basidiomycota; Agaricomycetes; Trechisporales; Hydnodontaceae; Trechispora; Trechispora_sp
New.ReferenceOTU4071	0	0	0	0	0	0	0	0	1	0	0	0	0	0	0	0	0	0	0	0	0	0	0	0	0	0	0	0	0	0	Fungi; Basidiomycota; Agaricomycetes; Trechisporales; Hydnodontaceae; Trechispora; Trechispora_sp
New.ReferenceOTU4444	0	1	0	0	0	0	0	0	0	0	0	0	0	0	0	0	0	0	0	0	0	0	0	0	0	0	0	0	0	0	Fungi; Basidiomycota; Agaricomycetes; Trechisporales; Hydnodontaceae; Trechispora; Trechispora_sp
New.ReferenceOTU4923	0	0	0	0	0	0	0	0	0	0	0	0	0	0	1	0	0	0	0	0	0	0	0	0	0	0	0	0	0	0	Fungi; Basidiomycota; Agaricomycetes; Trechisporales; Hydnodontaceae; Trechispora; Trechispora_sp
New.ReferenceOTU5171	1	0	0	0	0	0	0	0	0	0	0	0	0	0	0	0	0	0	0	0	0	0	0	0	0	0	0	0	0	0	Fungi; Basidiomycota; Agaricomycetes; Trechisporales; Hydnodontaceae; Trechispora; Trechispora_sp
New.ReferenceOTU5345	0	0	0	0	0	0	0	0	0	0	0	0	0	0	0	0	0	0	0	0	0	0	0	1	0	0	0	0	0	0	Fungi; Basidiomycota; Agaricomycetes; Trechisporales; Hydnodontaceae; Trechispora; Trechispora_sp
New.ReferenceOTU5373	1	0	0	0	0	0	0	0	0	0	0	0	0	0	0	0	0	0	0	0	0	0	0	0	0	0	0	0	0	0	Fungi; Basidiomycota; Agaricomycetes; Trechisporales; Hydnodontaceae; Trechispora; Trechispora_sp
New.ReferenceOTU5421	0	1	0	0	0	0	0	0	0	0	0	0	0	0	0	0	0	0	0	0	0	0	0	0	0	0	0	0	0	0	Fungi; Basidiomycota; Agaricomycetes; Trechisporales; Hydnodontaceae; Trechispora; Trechispora_sp
New.ReferenceOTU5938	1	0	0	0	0	0	0	0	0	0	0	0	0	0	0	0	0	0	0	0	0	0	0	0	0	0	0	0	0	0	Fungi; Basidiomycota; Agaricomycetes; Trechisporales; Hydnodontaceae; Trechispora; Trechispora_sp
New.ReferenceOTU6876	0	0	0	0	0	0	0	0	0	0	0	0	0	0	0	0	0	0	0	0	0	10	0	0	0	0	0	0	0	0	Fungi; Basidiomycota; Agaricomycetes; Trechisporales; Hydnodontaceae; Trechispora; Trechispora_sp
New.ReferenceOTU762	0	0	0	0	0	0	0	0	2	0	0	0	0	0	0	0	0	0	0	0	0	0	0	0	0	0	0	0	0	0	Fungi; Basidiomycota; Agaricomycetes; Trechisporales; Hydnodontaceae; Trechispora; Trechispora_sp
New.ReferenceOTU7809	0	0	0	0	0	0	0	0	0	0	0	0	0	0	0	0	0	0	0	0	0	0	0	2	0	0	0	0	0	0	Fungi; Basidiomycota; Agaricomycetes; Trechisporales; Hydnodontaceae; Trechispora; Trechispora_sp
New.ReferenceOTU3399	0	0	0	0	0	0	0	0	0	1	0	0	0	0	0	0	0	0	0	0	0	0	0	0	0	0	0	0	0	0	Fungi; Basidiomycota; Agaricomycetes; Trechisporales; Hydnodontaceae; Trechispora; Trechispora_sp_1_MPM_2013
New.ReferenceOTU861	1	0	0	0	0	0	0	0	0	0	0	0	0	0	0	0	0	0	0	0	0	0	0	0	0	0	0	0	0	0	Fungi; Basidiomycota; Agaricomycetes; Trechisporales; Hydnodontaceae; Trechispora; Trechispora_sp_DLL2009_138
New.ReferenceOTU6726	0	0	0	0	1	0	0	0	0	0	0	0	0	0	0	0	0	0	0	0	0	0	0	0	0	0	0	0	0	0	Fungi; Basidiomycota; Agaricostilbomycetes; Incertae_sedis; Incertae_sedis; Cystobasidiopsis; Cystobasidiopsis_sp_RB_2009a
New.ReferenceOTU4668	0	0	0	0	1	0	0	0	0	0	0	0	0	0	0	0	0	0	0	0	0	0	0	0	0	0	0	0	0	0	Fungi; Basidiomycota; Dacrymycetes; Dacrymycetales; Dacrymycetaceae; Calocera; Calocera_cornea
New.ReferenceOTU1211	0	3	0	0	0	0	0	0	0	0	0	0	0	0	0	0	1	1	0	8	0	0	0	1	0	0	0	0	0	0	Fungi; Basidiomycota; Dacrymycetes; Dacrymycetales; Dacrymycetaceae; Dacryopinax; Dacryopinax_indacocheae
New.ReferenceOTU877	0	1	0	0	0	0	0	0	0	0	0	0	0	0	0	0	0	0	1	0	0	0	0	0	0	0	0	0	0	0	Fungi; Basidiomycota; Dacrymycetes; Dacrymycetales; Dacrymycetaceae; Dacryopinax; Dacryopinax_indacocheae
New.ReferenceOTU4232	0	0	0	0	0	0	0	0	0	0	0	0	0	0	0	1	0	0	0	0	0	0	0	0	0	0	0	0	0	0	Fungi; Basidiomycota; Tremellomycetes; Tremellales; Incertae_sedis; Bandoniozyma; Bandoniozyma_visegradensis
New.CleanUp.ReferenceOTU1424	0	0	0	0	0	0	0	0	0	0	0	0	0	0	0	0	0	0	0	0	0	0	0	0	0	0	0	0	0	1	Fungi; Basidiomycota; Tremellomycetes; Tremellales; Incertae_sedis; Bullera; Bullera_miyagiana
New.ReferenceOTU8057	0	0	0	0	0	0	0	8	0	0	0	0	0	0	0	0	0	0	0	0	0	0	0	0	0	4	0	0	1	0	Fungi; Basidiomycota; Wallemiomycetes; Geminibasidiales; Geminibasidiaceae; Geminibasidium; Geminibasidium_sp
SH010365.06FU_AY970255_reps_singleton	0	0	0	0	0	1	1	10	0	0	1	0	0	0	0	0	0	0	0	0	0	0	0	0	0	3	0	0	4	0	Fungi; Basidiomycota; Wallemiomycetes; Geminibasidiales; Geminibasidiaceae; Geminibasidium; Geminibasidium_sp
SH102561.06FU_JN889968_reps	0	0	0	0	0	0	0	18	0	0	3	0	0	0	0	0	0	0	0	0	0	0	0	0	0	0	0	0	1	0	Fungi; Basidiomycota; Wallemiomycetes; Geminibasidiales; Geminibasidiaceae; Geminibasidium; Geminibasidium_sp
SH102562.06FU_FJ553224_reps	0	0	0	0	0	0	0	2	0	0	0	0	0	0	0	0	0	0	0	0	0	0	0	0	0	0	0	0	0	0	Fungi; Basidiomycota; Wallemiomycetes; Geminibasidiales; Geminibasidiaceae; Geminibasidium; Geminibasidium_sp

SH102563.06FU_KC818301_reps	0	0	0	0	0	1	0	7	0	0	3	0	0	1	0	0	0	0	0	0	0	0	0	0	0	0	0	0	0	0	Fungi; Basidiomycota; Wallemiomycetes; Geminibasidiales; Geminibasidiaceae; Geminibasidium; Geminibasidium_sp
New.ReferenceOTU4897	0	0	0	0	1	0	0	0	0	0	0	0	0	0	0	0	0	0	0	0	0	0	0	0	0	0	0	0	0	0	Fungi; Chytridiomycota; Chytridiomycetes; Rhizophlyctidales; Rhizophlyctidaceae; Rhizophlyctis; Rhizophlyctis_rosea
New.ReferenceOTU6973	0	0	0	0	0	0	0	0	0	0	0	0	0	0	0	0	0	0	0	0	0	2	0	0	0	0	0	0	0	0	Fungi; Chytridiomycota; Chytridiomycetes; Rhizophlyctidales; Rhizophlyctidaceae; Rhizophlyctis; Rhizophlyctis_rosea
New.ReferenceOTU2193	0	0	0	0	0	2	0	0	0	0	0	0	0	0	0	0	0	0	0	0	0	0	0	0	0	0	0	0	0	0	Fungi; Chytridiomycota; Chytridiomycetes; Rhizophlyctidales; Rhizophlyctidaceae; Rhizophlyctis; Rhizophlyctis_sp
New.ReferenceOTU4865	0	0	0	0	0	0	1	0	0	0	0	0	0	0	0	0	0	0	0	0	0	0	0	0	0	0	0	0	0	0	Fungi; Chytridiomycota; Chytridiomycetes; Rhizophlyctidales; Rhizophlyctidaceae; Rhizophlyctis; Rhizophlyctis_sp
New.ReferenceOTU3351	0	0	0	0	0	0	0	0	0	0	0	1	0	0	0	0	0	0	0	0	0	0	0	0	0	0	0	0	0	0	Fungi; Zygomycota; Incertae_sedis; Basidiobolales; Basidiobolaceae; Basidiobolus; Basidiobolus_ranarum
New.ReferenceOTU4647	0	0	0	0	0	0	0	0	0	0	0	0	0	0	0	0	0	0	1	0	0	0	0	0	0	0	0	0	0	0	Fungi; Zygomycota; Incertae_sedis; Basidiobolales; Basidiobolaceae; Basidiobolus; Basidiobolus_sp_ARSEF_5903
New.ReferenceOTU2425	1	0	0	0	0	0	0	0	0	0	0	0	0	0	0	0	0	0	0	0	0	0	0	0	0	0	0	0	0	0	Fungi; Zygomycota; Incertae_sedis; Basidiobolales; Basidiobolaceae; Basidiobolus; Basidiobolus_sp_BCU1
New.ReferenceOTU3343	0	0	0	0	0	0	0	0	0	0	0	0	0	0	0	0	0	0	0	0	0	0	0	1	0	0	0	0	0	0	Fungi; Zygomycota; Incertae_sedis; Kickxellales; Kickxellaceae; Coemansia; Coemansia_furcata
New.ReferenceOTU2512	1	0	0	0	0	0	0	0	0	0	0	0	0	0	0	0	0	0	0	0	0	0	0	0	0	0	0	0	0	0	Fungi; Zygomycota; Incertae_sedis; Kickxellales; Kickxellaceae; Ramicandelaber; Ramicandelaber_sp
New.ReferenceOTU1865	0	0	0	0	1	0	0	0	0	0	0	0	0	0	0	0	0	0	0	0	0	0	0	0	0	0	0	0	0	0	Fungi; Zygomycota; Incertae_sedis; Kickxellales; Kickxellaceae; Ramicandelaber; Ramicandelaber_sp_HMH_2010a
New.ReferenceOTU5299	1	0	0	0	0	0	0	0	0	0	0	0	0	0	0	0	0	0	0	0	0	0	0	0	0	0	0	0	0	0	Fungi; Zygomycota; Incertae_sedis; Mortierellales; Mortierellaceae; Mortierella; Mortierella_biramosa
New.ReferenceOTU6309	5	0	0	0	0	0	0	0	0	0	0	0	0	0	0	0	0	0	0	0	0	0	0	0	0	0	0	0	0	0	Fungi; Zygomycota; Incertae_sedis; Mortierellales; Mortierellaceae; Mortierella; Mortierella_biramosa
SH115626.06FU_HQ630311_reps	1	0	0	0	0	0	0	0	0	0	0	0	0	0	0	0	0	0	0	0	0	0	0	0	0	0	0	0	0	0	Fungi; Zygomycota; Incertae_sedis; Mortierellales; Mortierellaceae; Mortierella; Mortierella_biramosa
SH115632.06FU_JX975963_reps	3	0	0	2	0	0	0	0	0	0	0	0	0	0	0	0	0	0	0	0	0	0	0	0	0	0	0	0	0	0	Fungi; Zygomycota; Incertae_sedis; Mortierellales; Mortierellaceae; Mortierella; Mortierella_biramosa
SH115633.06FU_JX976094_reps	1	0	0	3	0	0	0	0	0	0	0	0	0	0	0	0	0	0	0	0	0	0	0	0	0	0	0	0	0	0	Fungi; Zygomycota; Incertae_sedis; Mortierellales; Mortierellaceae; Mortierella; Mortierella_biramosa
New.ReferenceOTU4456	3	0	0	0	0	0	0	0	0	0	0	0	0	0	0	0	0	0	0	0	0	0	0	0	0	0	0	0	0	0	Fungi; Zygomycota; Incertae_sedis; Mortierellales; Mortierellaceae; Mortierella; Mortierella_kuhlmanii
SH100052.06FU_HQ630294_refs	2	0	0	0	0	0	0	0	0	0	0	0	0	0	0	0	0	0	2	0	0	0	0	0	0	0	0	0	0	0	Fungi; Zygomycota; Incertae_sedis; Mortierellales; Mortierellaceae; Mortierella; Mortierella_kuhlmanii
New.ReferenceOTU2148	0	0	0	0	0	0	0	0	0	0	0	0	0	0	0	0	0	0	1	0	0	0	0	0	0	0	0	0	0	0	Fungi; Zygomycota; Incertae_sedis; Mortierellales; Mortierellaceae; Mortierella; Mortierella_stylospora
New.ReferenceOTU716	0	0	0	0	0	0	0	0	0	0	0	0	0	0	0	1	0	0	0	0	0	0	0	0	0	0	0	0	0	0	Fungi; Zygomycota; Incertae_sedis; Mucorales; Backusellaceae; Backusella; Backusella_recurva
New.ReferenceOTU2997	0	0	0	0	0	0	0	0	0	0	0	0	0	0	0	0	0	0	0	0	0	0	2	0	0	0	0	0	0	0	Fungi; Zygomycota; Incertae_sedis; Mucorales; Cunninghamellaceae; Absidia; Absidia_sp_A_7
New.ReferenceOTU2485	0	0	0	0	0	0	0	0	0	0	0	1	0	0	0	0	0	0	0	0	0	0	0	0	0	0	0	0	0	1	Fungi; Zygomycota; Incertae_sedis; Mucorales; Cunninghamellaceae; Gongronella; Gongronella_butleri
New.ReferenceOTU4223	0	0	0	0	4	0	0	0	0	0	0	1	0	0	0	0	0	0	0	0	0	0	2	0	0	0	0	0	0	1	Fungi; Zygomycota; Incertae_sedis; Mucorales; Cunninghamellaceae; Gongronella; Gongronella_butleri
New.ReferenceOTU1661	1	0	3	2	4	0	0	0	0	0	0	0	0	0	0	0	0	0	0	1	0	0	0	0	0	0	0	0	0	0	Fungi; Zygomycota; Incertae_sedis; Mucorales; Cunninghamellaceae; Gongronella; Gongronella_sp_11_KF_2011a
New.ReferenceOTU2265	0	0	0	0	4	0	0	0	0	0	0	0	0	0	0	0	0	0	0	0	0	0	0	0	0	0	0	0	0	0	Fungi; Zygomycota; Incertae_sedis; Mucorales; Cunninghamellaceae; Gongronella; Gongronella_sp_11_KF_2011a
New.ReferenceOTU2898	1	1	3	1	0	0	0	0	0	0	0	0	0	0	0	0	0	0	0	0	0	0	0	1	0	0	0	0	2	0	Fungi; Zygomycota; Incertae_sedis; Mucorales; Cunninghamellaceae; Gongronella; Gongronella_sp_11_KF_2011a
New.ReferenceOTU3513	0	0	0	0	0	0	0	0	0	0	0	0	0	0	0	0	0	0	0	0	0	0	0	1	0	0	0	0	0	0	Fungi; Zygomycota; Incertae_sedis; Mucorales; Cunninghamellaceae; Gongronella; Gongronella_sp_11_KF_2011a
New.ReferenceOTU5002	0	0	0	3	0	0	0	0	0	0	0	0	0	0	0	0	0	0	0	0	0	0	0	0	0	0	0	0	0	0	Fungi; Zygomycota; Incertae_sedis; Mucorales; Cunninghamellaceae; Gongronella; Gongronella_sp_11_KF_2011a
New.ReferenceOTU5250	0	0	0	2	0	0	0	0	0	0	0	0	0	0	0	0	0	0	0	0	0	0	0	0	0	0	0	0	0	0	Fungi; Zygomycota; Incertae_sedis; Mucorales; Cunninghamellaceae; Gongronella; Gongronella_sp_11_KF_2011a
New.ReferenceOTU5960	0	0	0	1	0	0	0	0	0	0	0	0	0	0	0	0	0	0	0	0	0	0	0	0	0	0	0	0	0	0	Fungi; Zygomycota; Incertae_sedis; Mucorales; Cunninghamellaceae; Gongronella; Gongronella_sp_11_KF_2011a
New.ReferenceOTU976	0	0	0	0	0	0	0	0	0	0	0	0	0	0	0	0	0	0	0	0	0	0	0	1	0	0	0	0	0	0	Fungi; Zygomycota; Incertae_sedis; Mucorales; Cunninghamellaceae; Gongronella; Gongronella_sp_11_KF_2011a
SH126734.06FU_JF728246_reps	0	0	0	4	0	0	0	0	0	0	0	0	0	0	0	0	0	0	0	0	0	0	0	0	0	0	0	0	0	0	Fungi; Zygomycota; Incertae_sedis; Mucorales; Cunninghamellaceae; Gongronella; Gongronella_sp_11_KF_2011a
SH020688.06FU_FJ713077_reps_singleton	0	0	0	0	0	0	0	0	0	0	0	0	0	0	0	0	0	0	0	2	0	0	0	0	0	0	0	0	0	0	Fungi; Zygomycota; Incertae_sedis; Mucorales; Lichtheimiaceae; Rhizomucor; Rhizomucor_pusillus
New.ReferenceOTU5794	0	0	1	0	0	0	1	1	0	2	0	1	0	0	0	0	0	0	0	0	0	0	3	0	0	0	0	0	0	0	Fungi; Zygomycota; Incertae_sedis; Mucorales; Mucoraceae; Hyphomucor; Hyphomucor_assamensis
SH032751.06FU_JN206211_refs_singleton	0	0	0	0	0	0	2	0	0	0	0	0	0	0	0	0	0	0	0	0	0	0	0	0	0	0	0	0	0	0	Fungi; Zygomycota; Incertae_sedis; Mucorales; Mucoraceae; Hyphomucor; Hyphomucor_assamensis
New.ReferenceOTU6606	0	0	0	0	0	0	0	0	0	0	0	0	0	0	0	0	0	0	0	0	0	0	1	0	0	0	0	0	0	0	Fungi; Zygomycota; Incertae_sedis; Mucorales; Mucoraceae; Mucor; Mucor_ardhlaengiktus
SH026519.06FU_JF299211_reps_singleton	0	0	0	0	0	1	1	0	0	0	0	0	0	0	0	0	0	0	0	0	0	0	0	0	0	0	0	0	0	0	Fungi; Zygomycota; Incertae_sedis; Mucorales; Mucoraceae; Mucor; Mucor_ardhlaengiktus
SH129460.06FU_JN206172_refs	0	0	0	0	0	1	0	0	0	0	0	0	0	0	0	0	0	0	0	0	0	0	0	0	0	0	0	0	0	0	Fungi; Zygomycota; Incertae_sedis; Mucorales; Mucoraceae; Mucor; Mucor_ardhlaengiktus
New.ReferenceOTU1853	0	0	0	0	0	0	0	0	0	0	0	0	0	0	0	0	0	0	0	0	0	0	0	0	0	0	0	0	1	0	Fungi; Zygomycota; Incertae_sedis; Mucorales; Mucoraceae; Mucor; Mucor_fuscus
New.ReferenceOTU3008	0	0	0	0	0	0	0	0	0	0	0	0	0	0	0	0	0	0	0	0	0	0	0	0	0	0	0	0	1	0	Fungi; Zygomycota; Incertae_sedis; Mucorales; Mucoraceae; Mucor; Mucor_fuscus
New.CleanUp.ReferenceOTU1760	0	0	0	0	0	0	0	0	0	0	1	0	0	0	0	0	0	0	0	0	0	0	0	0	0	0	0	0	0	0	Fungi; Zygomycota; Incertae_sedis; Mucorales; Mucoraceae; Mucor; Mucor_indicus
New.ReferenceOTU1123	0	0	0	0	0	0	0	0	0	0	0	0	0	0	0	0	0	0	0	0	0	1	0	0	1	0	0	0	0	0	Fungi; Zygomycota; Incertae_sedis; Mucorales; Mucoraceae; Mucor; Mucor_sp
New.ReferenceOTU2283	0	0	0	0	0	0	0	0	0	0	0	0	0	0	0	0	0	0	0	0	0	0	1	0	0	0	0	0	0	0	Fungi; Zygomycota; Incertae_sedis; Mucorales; Mucoraceae; Mucor; Mucor_sp_L_5
New.ReferenceOTU1096	0	0	0	0	0	0	0	0	0	0	0	0	1	0	0	0	0	0	0	0	0	0	0	0	0	0	0	0	0	0	Fungi; Zygomycota; Incertae_sedis; Mucorales; Mucoraceae; Parasitella; Parasitella_sp
New.ReferenceOTU3974	0	0	0	0	0	0	0	0	0	0	0	0	0	0	0	0	0	0	0	0	0	6	0	0	0	0	0	1	0	0	Fungi; Zygomycota; Incertae_sedis; Mucorales; Umbelopsidaceae; Umbelopsis; Umbelopsis_angularis
SH104285.06FU_JN206380_refs	0	0	0	0	0	0	0	0	0	0	0	0	0	0	0	0	0	0	0	0	0	1	0	0	0	0	0	0	0	0	Fungi; Zygomycota; Incertae_sedis; Mucorales; Umbelopsidaceae; Umbelopsis; Umbelopsis_angularis
New.ReferenceOTU6086	0	0	0	0	0	0	0	0	0	0	0	0	0	0	0	0	0	0	0	0	0	2	0	0	0	0	0	0	0	0	Fungi; Zygomycota; Incertae_sedis; Mucorales; Umbelopsidaceae; Umbelopsis; Umbelopsis_gibberispora
New.ReferenceOTU22	0	0	0	0	2	0	0	1	0	0	0	0	0	0	0	0	0	0	0	0	0	0	0	0	0	0	0	0	0	0	Fungi; Zygomycota; Incertae_sedis; Mucorales; Umbelopsidaceae; Umbelopsis; Umbelopsis_isabellina
New.ReferenceOTU4755	1	0	0	0	0	0	0	0	0	0	0	0	0	0	0	0	0	0	0	0	0	0	0	0	0	0	0	0	0	0	Fungi; Zygomycota; Incertae_sedis; Mucorales; Umbelopsidaceae; Umbelopsis; Umbelopsis_isabellina
SH109228.06FU_HQ608128_reps	0	0	2	0	0	0	0	0	0	0	0	0	0	0	0	0	0	0	0	0	0	0	0	0	0	0	0	0	0	0	Fungi; Zygomycota; Incertae_sedis; Mucorales; Umbelopsidaceae; Umbelopsis; Umbelopsis_isabellina
SH109229.06FU_AY805542_reps	0	0	0	0	0	0	0	0	0	0	0	0	0	0	0	0	0	0	0	0	0	1	0	0	0	0	0	0	0	0	Fungi; Zygomycota; Incertae_sedis; Mucorales; Umbelopsidaceae; Umbelopsis; Umbelopsis_isabellina
New.ReferenceOTU2212	0	0	0	0	0	0	0	0	0	0	0	0	0	0	0	0	0	0	0	0	0	3	0	0	0	0	0	0	0	0	Fungi; Zygomycota; Incertae_sedis; Mucorales; Umbelopsidaceae; Umbelopsis; Umbelopsis_ramanniana
New.ReferenceOTU4344	0	0	0	0	0	0	0	0	0	0	0	0	0	0	1	0	0	0	0	0	0	3	0	0	0	0	0	0	0	0	Fungi; Zygomycota; Incertae_sedis; Mucorales; Umbelopsidaceae; Umbelopsis; Umbelopsis_ramanniana
New.CleanUp.ReferenceOTU1884	0	0	0	0	0	0	0	0	0	0	0	0	0	0	0	0	0	1	0	0	0	0	0	0	0	0	0	0	0	0	Fungi; Zygomycota; Incertae_sedis; Mucorales; Umbelopsidaceae; Umbelopsis; Umbelopsis_sp
New.CleanUp.ReferenceOTU472	0	0	0	0	3	0	1	0	0	0	0	0	0	0	0	0	0	0	0	0	0	0	0	0	0	0	0	0	0	0	Fungi; Zygomycota; Incertae_sedis; Mucorales; Umbelopsidaceae; Umbelopsis; Umbelopsis_sp
New.ReferenceOTU101	0	0	0	0	0	0	0	0	5	0	0	0	0	0	0	0	0	0	0	0	0	0	0	0	0	0	0	0	0	0	Fungi; Zygomycota; Incertae_sedis; Mucorales; Umbelopsidaceae; Umbelopsis; Umbelopsis_sp
New.ReferenceOTU1023	5	0	0	0	0	0	1	0	0	0	0	0	0	0	0	0	0	1	0	0	4	0	0	0	0	2	0	0	0	0	Fungi; Zygomycota; Incertae_sedis; Mucorales; Umbelopsidaceae; Umbelopsis; Umbelopsis_sp
New.ReferenceOTU1050	0	0	0	0	0	0	0	0	0	0	0	0	0	0	0	0	0	0	0	0	0	0	0	0	0	2	0	0	0	0	Fungi; Zygomycota; Incertae_sedis; Mucorales; Umbelopsidaceae; Umbelopsis; Umbelopsis_sp
New.ReferenceOTU1051	1	0	0	0	2	0	0	0	0	0	0	0	0	0	0	0	0	0	0	1	0	0	0	0	0	0	0	0	0	0	Fungi; Zygomycota; Incertae_sedis; Mucorales; Umbelopsidaceae; Umbelopsis; Umbelopsis_sp
New.ReferenceOTU1102	0	0	0	0	0	0	0	0	0	0	0	0	0	0	0	0	0	0	0	0	0	0	0	0	0	0	0	0	1	0	Fungi; Zygomycota; Incertae_sedis; Mucorales; Umbelopsidaceae; Umbelopsis; Umbelopsis_sp
New.ReferenceOTU1196	0	0	0	0	0	0	0	0	0	0	0	1	0	0	0	0	0	0	0	0	0	0	0	0	0	0	0	0	0	0	Fungi; Zygomycota; Incertae_sedis; Mucorales; Umbelopsidaceae; Umbelopsis; Umbelopsis_sp
New.ReferenceOTU1332	0	0	0	0	1	0	0	0	0	0	0	1	0	0	0	0	0	0	0	0	0	0	0	0	0	0	0	0	0	0	Fungi; Zygomycota; Incertae_sedis; Mucorales; Umbelopsidaceae; Umbelopsis; Umbelopsis_sp
New.ReferenceOTU1391	5	0	0	0	0	0	0	0	0	0	0	0	0	0	0	0	0	0	0	0	0	0	0	0	0	0	0	0	0	0	Fungi; Zygomycota; Incertae_sedis; Mucorales; Umbelopsidaceae; Umbelopsis; Umbelopsis_sp
New.ReferenceOTU1508	0	0	0	1	4	1	1	1	0	0	0	5	0	0	0	0	0	0	0	0	2	0	0	1	3	1	0	0	1	1	Fungi; Zygomycota; Incertae_sedis; Mucorales; Umbelopsidaceae; Umbelopsis; Umbelopsis_sp
New.ReferenceOTU1519	1	0	0	1	0	5	2	1	0	1	0	0	0	0	1	0	0	0	0	0	0	0	0	0	0	0	0	0	1	0	Fungi; Zygomycota; Incertae_sedis; Mucorales; Umbelopsidaceae; Umbelopsis; Umbelopsis_sp
New.ReferenceOTU159	0	0	0	0	1	0	1	0	0	0	0	1	0	0	1	0	0	0	0	0	1	1	0	0	0	0	0	0	0	0	Fungi; Zygomycota; Incertae_sedis; Mucorales; Umbelopsidaceae; Umbelopsis; Umbelopsis_sp
New.ReferenceOTU1670	0	0	0	0	0	0	0	0	0	0	0	0	0	0	0	0	0	1	0	0	0	0	0	0	0	0	0	0	0	0	Fungi; Zygomycota; Incertae_sedis; Mucorales; Umbelopsidaceae; Umbelopsis; Umbelopsis_sp
New.ReferenceOTU1763	0	0	0	0	0	0	0	0	1	0	0	0	0	0	0	0	0	0	0	0	0	0	0	0	0	0	0	0	0	0	Fungi; Zygomycota; Incertae_sedis; Mucorales; Umbelopsidaceae; Umbelopsis; Umbelopsis_sp
New.ReferenceOTU1839	0	0	0	0	1	0	0	0	0	0	0	1	0	0	0	0	0	0	0	0	0	0	0	0	0	0	0	0	0	0	Fungi; Zygomycota; Incertae_sedis; Mucorales; Umbelopsidaceae; Umbelopsis; Umbelopsis_sp
New.ReferenceOTU1899	0	0	0	0	0	0	0	0	0	0	0	0	0	0	0	0	0	0	0	0	0	0	0	0	0	1	0	0	0	0	Fungi; Zygomycota; Incertae_sedis; Mucorales; Umbelopsidaceae; Umbelopsis; Umbelopsis_sp
New.ReferenceOTU1911	4	0	0	0	0	0	0	0	0	0	1	0	0	0	0	0	0	0	0	0	0	0	0	1	0	0	0	0	1	0	Fungi; Zygomycota; Incertae_sedis; Mucorales; Umbelopsidaceae; Umbelopsis; Umbelopsis_sp
New.ReferenceOTU1988	0	0	0	0	0	0	0	0	5	0	0	0	0	0	0	0	0	0	0	0	0	0	0	0	0	0	0	0	0	0	Fungi; Zygomycota; Incertae_sedis; Mucorales; Umbelopsidaceae; Umbelopsis; Umbelopsis_sp
New.ReferenceOTU2017	0	0	0	0	0	0	0	0	0	0	0	2	0	0	0	0	0	0	0	0	0	0	0	0	0	0	0	0	0	0	Fungi; Zygomycota; Incertae_sedis; Mucorales; Umbelopsidaceae; Umbelopsis; Umbelopsis_sp
New.ReferenceOTU2098	2	1	0	0	0	0	0	0	0	0	0	0	0	0	0	0	0	0	0	0	0	0	0	4	0	0	0	0	0	0	Fungi; Zygomycota; Incertae_sedis; Mucorales; Umbelopsidaceae; Umbelopsis; Umbelopsis_sp
New.ReferenceOTU2132	0	0	0	0	0	0	0	0	0	0	0	0	0	0	0	0	0	1	0	0	0	0	0	1	0	0	0	0	0	0	Fungi; Zygomycota; Incertae_sedis; Mucorales; Umbelopsidaceae; Umbelopsis; Umbelopsis_sp
New.ReferenceOTU2270	2	0	0	0	2	0	0	1	0	0	0	2	0	0	1	0	0	0	0	0	0	0	0	0	0	0	0	0	1	0	Fungi; Zygomycota; Incertae_sedis; Mucorales; Umbelopsidaceae; Umbelopsis; Umbelopsis_sp
New.ReferenceOTU2493	1	0	0	0	0	0	0	0	0	0	0	0	0	0	0	0	0	0	0	0	0	0	0	0	0	0	0	0	0	0	Fungi; Zygomycota; Incertae_sedis; Mucorales; Umbelopsidaceae; Umbelopsis; Umbelopsis_sp
New.ReferenceOTU2503	0	0	0	0	0	0	0	0	0	0	2	0	0	0	0	0	0	0	0	0	0	0	0	0	0	0	0	0	0	0	Fungi; Zygomycota; Incertae_sedis; Mucorales; Umbelopsidaceae; Umbelopsis; Umbelopsis_sp
New.ReferenceOTU2545	0	0	0	0	0	0	0	0	0	0	1	0	0	0	0	0	0	0	0	0	0	0	0	0	0	0	0	0	0	0	Fungi; Zygomycota; Incertae_sedis; Mucorales; Umbelopsidaceae; Umbelopsis; Umbelopsis_sp
New.ReferenceOTU2825	0	0	0	0	0	0	0	0	0	1	0	0	0	0	0	0	0	0	1	0	0	0	0	0	0	0	0	0	0	0	Fungi; Zygomycota; Incertae_sedis; Mucorales; Umbelopsidaceae; Umbelopsis; Umbelopsis_sp
New.ReferenceOTU3039	0	0	0	0	0	0	0	0	0	0	0	1	0	0	0	0	0	0	0	0	0	0	0	0	0	0	0	0	0	0	Fungi; Zygomycota; Incertae_sedis; Mucorales; Umbelopsidaceae; Umbelopsis; Umbelopsis_sp
New.ReferenceOTU3118	0	0	0	0	14	0	0	0	0	0	0	0	0	0	0	0	0	0	0	0	0	0	0	0	0	0	0	0	0	0	Fungi; Zygomycota; Incertae_sedis; Mucorales; Umbelopsidaceae; Umbelopsis; Umbelopsis_sp
New.ReferenceOTU317	2	1	0	0	1	0	4	1	0	0	0	2	1	0	0	0	0	0	0	0	2	0	0	0	0	0	0	0	0	0	Fungi; Zygomycota; Incertae_sedis; Mucorales; Umbelopsidaceae; Umbelopsis; Umbelopsis_sp
New.ReferenceOTU3186	0	0	0	0	0	0	0	0	1	0	0	0	0	0	0	0	0	0	0	0	0	0	0	0	0	0	0	0	0	0	Fungi; Zygomycota; Incertae_sedis; Mucorales; Umbelopsidaceae; Umbelopsis; Umbelopsis_sp
New.ReferenceOTU3342	2	0	0	0	0	0	0	0	0	0	0	0	0	0	0	0	0	0	0	0	1	0	0	0	0	0	0	0	0	0	Fungi; Zygomycota; Incertae_sedis; Mucorales; Umbelopsidaceae; Umbelopsis; Umbelopsis_sp
New.ReferenceOTU3534	4	0	0	0	0	0	0	0	0	0	0	0	0	0	0	0	0	0	0	0	0	0	0	0	0	0	0	0	0	0	Fungi; Zygomycota; Incertae_sedis; Mucorales; Umbelopsidaceae; Umbelopsis; Umbelopsis_sp
New.ReferenceOTU3614	0	0	0	0	0	0	0	0	0	0	0	0	0	0	0	0	0	5	0	0	0	0	0	0	0	0	0	0	0	0	Fungi; Zygomycota; Incertae_sedis; Mucorales; Umbelopsidaceae; Umbelopsis; Umbelopsis_sp
New.ReferenceOTU3617	0	0	0	0	0	0	0	0	0	0	0	1	0	0	0	0	0	0	0	0	0	0	0	0	0	0	0	0	0	0	Fungi; Zygomycota; Incertae_sedis; Mucorales; Umbelopsidaceae; Umbelopsis; Umbelopsis_sp
New.ReferenceOTU3796	57	1	0	0	0	0	7	0	0	1	0	0	0	0	0	0	1	1	0	0	4	0	0	5	0	0	0	0	6	0	Fungi; Zygomycota; Incertae_sedis; Mucorales; Umbelopsidaceae; Umbelopsis; Umbelopsis_sp
New.ReferenceOTU3808	0	0	0	0	1	0	1	0	0	1	0	0	0	0	0	0	0	0	0	0	0	0	0	0	0	0	0	0	0	0	Fungi; Zygomycota; Incertae_sedis; Mucorales; Umbelopsidaceae; Umbelopsis; Umbelopsis_sp
New.ReferenceOTU4155	5	0	0	1	0	0	0	0	0	0	0	0	0	0	0	0	0	0	0	0	0	0	0	0	0	0	0	0	2	0	Fungi; Zygomycota; Incertae_sedis; Mucorales; Umbelopsidaceae; Umbelopsis; Umbelopsis_sp
New.ReferenceOTU4158	0	2	0	0	1	0	0	0	0	0	0	2	0	0	0	0	0	0	0	0	0	0	0	0	0	0	0	0	0	0	Fungi; Zygomycota; Incertae_sedis; Mucorales; Umbelopsidaceae; Umbelopsis; Umbelopsis_sp
New.ReferenceOTU4280	0	0	0	0	1	0	1	0	0	0	0	1	0	0	0	0	0	0	0	0	0	0	0	0	1	1	0	0	0	0	Fungi; Zygomycota; Incertae_sedis; Mucorales; Umbelopsidaceae; Umbelopsis; Umbelopsis_sp
New.ReferenceOTU4313	0	0	0	0	0	0	0	0	0	0	0	1	0	0	0	0	0	0	0	0	0	0	0	0	0	0	0	0	0	0	Fungi; Zygomycota; Incertae_sedis; Mucorales; Umbelopsidaceae; Umbelopsis; Umbelopsis_sp
New.ReferenceOTU4637	0	0	0	0	0	0	0	0	4	0	0	0	0	0	0	0	0	0	0	0	0	0	0	0	0	0	0	0	0	0	Fungi; Zygomycota; Incertae_sedis; Mucorales; Umbelopsidaceae; Umbelopsis; Umbelopsis_sp
New.ReferenceOTU471	0	0	0	0	8	0	0	0	0	0	0	0	0	0	0	0	0	0	0	0	0	0	0	0	0	0	0	0	0	0	Fungi; Zygomycota; Incertae_sedis; Mucorales; Umbelopsidaceae; Umbelopsis; Umbelopsis_sp
New.ReferenceOTU4723	0	0	0	1	0	0	0	0	0	0	0	0	0	0	0	0	0	0	0	0	0	0	0	0	0	0	3	0	0	0	Fungi; Zygomycota; Incertae_sedis; Mucorales; Umbelopsidaceae; Umbelopsis; Umbelopsis_sp
New.ReferenceOTU4878	0	0	0	1	0	0	0	0	0	0	0	0	0	0	0	0	0	0	0	0	0	0	0	0	0	0	0	0	0	0	Fungi; Zygomycota; Incertae_sedis; Mucorales; Umbelopsidaceae; Umbelopsis; Umbelopsis_sp
New.ReferenceOTU4885	0	0	0	0	0	0	0	1	0	0	0	0	0	0	0	0	0	0	0	0	0	0	0	0	0	0	0	0	1	0	Fungi; Zygomycota; Incertae_sedis; Mucorales; Umbelopsidaceae; Umbelopsis; Umbelopsis_sp
New.ReferenceOTU490	0	0	0	0	0	0	0	0	0	0	0	0	0	0	0	0	0	0	0	0	0	0	1	0	0	0	0	0	0	0	Fungi; Zygomycota; Incertae_sedis; Mucorales; Umbelopsidaceae; Umbelopsis; Umbelopsis_sp

New.ReferenceOTU491	1	0	0	0	0	0	0	0	0	0	0	0	0	0	0	0	0	0	0	0	0	0	0	0	0	0	0	0	0	0	Fungi; Zygomycota; Incertae_sedis; Mucorales; Umbelopsidaceae; Umbelopsis; Umbelopsis_sp
New.ReferenceOTU4975	0	0	0	2	0	0	2	1	0	0	0	0	0	0	0	0	0	0	0	0	0	0	0	0	0	0	0	0	0	0	Fungi; Zygomycota; Incertae_sedis; Mucorales; Umbelopsidaceae; Umbelopsis; Umbelopsis_sp
New.ReferenceOTU5038	0	0	0	0	0	0	0	0	0	0	0	0	0	0	0	0	0	0	3	0	0	0	0	0	0	0	0	0	0	0	Fungi; Zygomycota; Incertae_sedis; Mucorales; Umbelopsidaceae; Umbelopsis; Umbelopsis_sp
New.ReferenceOTU5039	0	0	0	1	0	0	0	0	0	0	0	0	0	0	0	0	0	0	0	0	0	0	0	0	0	0	0	0	0	0	Fungi; Zygomycota; Incertae_sedis; Mucorales; Umbelopsidaceae; Umbelopsis; Umbelopsis_sp
New.ReferenceOTU5095	0	1	0	0	1	0	1	0	0	0	0	0	0	0	0	0	0	0	0	0	1	0	0	0	0	0	0	0	0	0	Fungi; Zygomycota; Incertae_sedis; Mucorales; Umbelopsidaceae; Umbelopsis; Umbelopsis_sp
New.ReferenceOTU5235	4	0	0	0	0	0	0	0	0	0	0	0	0	0	0	0	0	0	0	0	0	0	0	0	0	3	0	0	2	0	Fungi; Zygomycota; Incertae_sedis; Mucorales; Umbelopsidaceae; Umbelopsis; Umbelopsis_sp
New.ReferenceOTU5280	0	0	0	0	0	0	0	0	0	0	2	0	0	0	0	0	0	0	0	0	0	0	0	0	0	0	0	0	0	0	Fungi; Zygomycota; Incertae_sedis; Mucorales; Umbelopsidaceae; Umbelopsis; Umbelopsis_sp
New.ReferenceOTU5426	1	0	0	0	0	0	0	0	0	0	0	0	0	0	0	0	0	1	0	0	1	0	0	0	0	0	0	0	1	0	Fungi; Zygomycota; Incertae_sedis; Mucorales; Umbelopsidaceae; Umbelopsis; Umbelopsis_sp
New.ReferenceOTU5456	0	0	0	0	0	0	0	0	0	0	1	0	0	0	0	0	0	0	0	0	0	0	0	0	0	0	0	0	0	0	Fungi; Zygomycota; Incertae_sedis; Mucorales; Umbelopsidaceae; Umbelopsis; Umbelopsis_sp
New.ReferenceOTU5509	0	0	0	0	0	0	0	0	1	0	0	0	0	0	0	0	0	0	0	0	0	0	0	0	0	0	0	0	0	0	Fungi; Zygomycota; Incertae_sedis; Mucorales; Umbelopsidaceae; Umbelopsis; Umbelopsis_sp
New.ReferenceOTU5535	0	0	0	0	0	0	0	0	0	0	2	0	0	0	0	0	0	0	0	0	0	0	0	0	0	0	0	0	0	0	Fungi; Zygomycota; Incertae_sedis; Mucorales; Umbelopsidaceae; Umbelopsis; Umbelopsis_sp
New.ReferenceOTU5596	0	0	0	0	0	0	0	0	1	0	0	1	0	0	0	0	0	0	0	0	0	0	0	0	0	0	0	0	0	0	Fungi; Zygomycota; Incertae_sedis; Mucorales; Umbelopsidaceae; Umbelopsis; Umbelopsis_sp
New.ReferenceOTU5604	0	0	0	0	0	0	0	0	2	0	0	0	0	0	0	0	0	0	0	0	0	0	0	0	0	0	0	0	0	0	Fungi; Zygomycota; Incertae_sedis; Mucorales; Umbelopsidaceae; Umbelopsis; Umbelopsis_sp
New.ReferenceOTU5655	0	0	0	0	0	0	1	0	0	0	0	0	0	1	0	0	0	0	0	1	0	0	0	1	0	0	0	0	1	0	Fungi; Zygomycota; Incertae_sedis; Mucorales; Umbelopsidaceae; Umbelopsis; Umbelopsis_sp
New.ReferenceOTU578	0	0	0	0	0	0	0	0	0	0	0	0	0	0	0	0	0	0	0	0	0	0	1	0	0	0	0	0	0	0	Fungi; Zygomycota; Incertae_sedis; Mucorales; Umbelopsidaceae; Umbelopsis; Umbelopsis_sp
New.ReferenceOTU5818	0	0	0	0	0	0	0	0	0	0	0	0	0	0	0	0	0	2	0	0	0	0	0	0	0	0	0	0	0	0	Fungi; Zygomycota; Incertae_sedis; Mucorales; Umbelopsidaceae; Umbelopsis; Umbelopsis_sp
New.ReferenceOTU5820	0	0	0	0	0	0	0	0	0	0	0	0	0	0	0	0	1	0	0	0	0	0	0	0	0	0	0	0	0	0	Fungi; Zygomycota; Incertae_sedis; Mucorales; Umbelopsidaceae; Umbelopsis; Umbelopsis_sp
New.ReferenceOTU5914	1	0	0	0	1	0	1	0	0	0	0	1	0	0	0	0	1	0	0	0	1	0	0	0	0	3	0	0	0	0	Fungi; Zygomycota; Incertae_sedis; Mucorales; Umbelopsidaceae; Umbelopsis; Umbelopsis_sp
New.ReferenceOTU5944	2	2	0	0	3	0	1	2	0	1	0	4	0	0	0	0	0	0	0	0	4	0	0	1	0	0	0	0	2	0	Fungi; Zygomycota; Incertae_sedis; Mucorales; Umbelopsidaceae; Umbelopsis; Umbelopsis_sp
New.ReferenceOTU5986	0	0	0	0	0	0	0	0	1	0	0	0	0	1	0	0	0	0	0	0	0	0	0	0	0	0	0	0	0	0	Fungi; Zygomycota; Incertae_sedis; Mucorales; Umbelopsidaceae; Umbelopsis; Umbelopsis_sp
New.ReferenceOTU6025	0	0	0	0	1	0	0	0	0	0	0	0	0	0	0	0	0	0	0	0	0	0	0	0	0	2	0	0	0	0	Fungi; Zygomycota; Incertae_sedis; Mucorales; Umbelopsidaceae; Umbelopsis; Umbelopsis_sp
New.ReferenceOTU6151	0	0	0	0	0	0	1	0	1	1	0	0	0	0	0	1	0	0	0	0	0	0	0	0	0	0	0	0	0	0	Fungi; Zygomycota; Incertae_sedis; Mucorales; Umbelopsidaceae; Umbelopsis; Umbelopsis_sp
New.ReferenceOTU6258	0	0	0	0	1	0	0	0	0	0	0	0	0	0	0	0	0	0	0	0	0	0	0	0	0	0	0	0	0	0	Fungi; Zygomycota; Incertae_sedis; Mucorales; Umbelopsidaceae; Umbelopsis; Umbelopsis_sp
New.ReferenceOTU6347	0	0	0	0	0	0	0	0	0	0	0	0	0	0	0	0	1	0	0	0	0	0	0	0	0	0	0	0	0	0	Fungi; Zygomycota; Incertae_sedis; Mucorales; Umbelopsidaceae; Umbelopsis; Umbelopsis_sp
New.ReferenceOTU6389	0	2	0	0	0	0	0	0	0	0	0	0	0	0	0	0	0	0	0	0	0	0	0	0	0	0	0	0	1	0	Fungi; Zygomycota; Incertae_sedis; Mucorales; Umbelopsidaceae; Umbelopsis; Umbelopsis_sp
New.ReferenceOTU6514	0	0	0	0	0	0	0	0	0	0	0	0	0	1	1	0	0	0	0	0	0	0	0	0	0	0	0	0	0	0	Fungi; Zygomycota; Incertae_sedis; Mucorales; Umbelopsidaceae; Umbelopsis; Umbelopsis_sp
New.ReferenceOTU6518	1	0	0	0	0	0	0	0	0	0	0	0	0	0	0	0	0	0	0	0	0	0	0	0	0	0	0	0	0	0	Fungi; Zygomycota; Incertae_sedis; Mucorales; Umbelopsidaceae; Umbelopsis; Umbelopsis_sp
New.ReferenceOTU6664	0	2	0	0	0	0	0	0	0	0	0	0	0	0	0	0	0	0	0	0	0	0	0	0	0	0	0	0	0	0	Fungi; Zygomycota; Incertae_sedis; Mucorales; Umbelopsidaceae; Umbelopsis; Umbelopsis_sp
New.ReferenceOTU6695	0	0	0	0	0	0	0	0	0	0	1	0	0	0	0	0	0	0	0	0	0	0	0	0	0	0	0	0	0	0	Fungi; Zygomycota; Incertae_sedis; Mucorales; Umbelopsidaceae; Umbelopsis; Umbelopsis_sp
New.ReferenceOTU688	0	0	0	0	0	0	0	0	0	0	1	0	0	0	0	0	0	0	0	0	0	0	0	0	0	0	0	0	0	0	Fungi; Zygomycota; Incertae_sedis; Mucorales; Umbelopsidaceae; Umbelopsis; Umbelopsis_sp
New.ReferenceOTU7054	0	0	0	0	0	0	0	0	0	0	0	0	0	0	0	0	0	3	0	0	0	0	0	0	0	0	0	0	0	0	Fungi; Zygomycota; Incertae_sedis; Mucorales; Umbelopsidaceae; Umbelopsis; Umbelopsis_sp
New.ReferenceOTU7185	0	1	0	0	0	0	0	0	0	0	0	0	0	0	0	0	0	0	0	0	0	0	0	0	0	0	0	0	0	0	Fungi; Zygomycota; Incertae_sedis; Mucorales; Umbelopsidaceae; Umbelopsis; Umbelopsis_sp
New.ReferenceOTU7281	0	0	0	0	0	0	0	0	0	0	0	1	0	0	0	0	0	0	0	0	0	0	0	0	0	0	0	0	0	0	Fungi; Zygomycota; Incertae_sedis; Mucorales; Umbelopsidaceae; Umbelopsis; Umbelopsis_sp
New.ReferenceOTU7406	0	0	0	0	0	0	0	0	3	0	0	0	0	0	0	0	0	0	0	0	0	0	0	0	0	0	0	0	0	0	Fungi; Zygomycota; Incertae_sedis; Mucorales; Umbelopsidaceae; Umbelopsis; Umbelopsis_sp
New.ReferenceOTU7447	0	0	0	0	3	0	0	0	0	0	0	0	0	0	0	0	0	0	0	0	0	0	0	0	0	0	0	0	0	0	Fungi; Zygomycota; Incertae_sedis; Mucorales; Umbelopsidaceae; Umbelopsis; Umbelopsis_sp
New.ReferenceOTU7569	0	0	0	0	0	0	0	0	0	0	0	0	0	0	0	0	0	0	0	1	0	0	0	0	0	0	0	0	0	0	Fungi; Zygomycota; Incertae_sedis; Mucorales; Umbelopsidaceae; Umbelopsis; Umbelopsis_sp
New.ReferenceOTU7647	1	1	0	1	2	0	0	0	0	0	0	1	0	0	0	0	0	0	0	0	0	0	0	0	0	0	0	0	0	0	Fungi; Zygomycota; Incertae_sedis; Mucorales; Umbelopsidaceae; Umbelopsis; Umbelopsis_sp
New.ReferenceOTU7692	0	0	0	0	2	0	0	0	0	0	4	0	0	0	0	0	0	0	0	0	0	0	0	0	0	0	0	0	0	0	Fungi; Zygomycota; Incertae_sedis; Mucorales; Umbelopsidaceae; Umbelopsis; Umbelopsis_sp
New.ReferenceOTU7702	0	0	0	0	0	0	1	0	0	0	0	0	0	0	0	0	0	0	0	0	0	0	0	0	0	0	0	0	0	0	Fungi; Zygomycota; Incertae_sedis; Mucorales; Umbelopsidaceae; Umbelopsis; Umbelopsis_sp
New.ReferenceOTU7715	0	0	0	0	0	0	0	0	4	0	0	0	0	0	0	0	0	0	0	0	0	0	0	0	0	0	0	0	0	0	Fungi; Zygomycota; Incertae_sedis; Mucorales; Umbelopsidaceae; Umbelopsis; Umbelopsis_sp
New.ReferenceOTU7921	0	0	0	0	0	0	0	0	0	0	0	0	0	0	0	0	0	3	0	0	0	0	0	0	0	0	0	0	0	0	Fungi; Zygomycota; Incertae_sedis; Mucorales; Umbelopsidaceae; Umbelopsis; Umbelopsis_sp
New.ReferenceOTU7980	2	0	0	0	0	0	2	0	0	0	0	0	0	0	0	0	0	0	0	0	0	0	0	1	0	9	0	0	0	0	Fungi; Zygomycota; Incertae_sedis; Mucorales; Umbelopsidaceae; Umbelopsis; Umbelopsis_sp
New.ReferenceOTU8037	0	0	0	0	4	0	3	0	0	2	0	5	0	0	0	0	0	0	0	0	0	0	0	0	0	2	0	0	0	0	Fungi; Zygomycota; Incertae_sedis; Mucorales; Umbelopsidaceae; Umbelopsis; Umbelopsis_sp
New.ReferenceOTU971	0	0	0	0	0	2	0	0	0	0	0	0	0	0	0	0	0	0	0	0	0	0	0	0	0	0	0	0	0	0	Fungi; Zygomycota; Incertae_sedis; Mucorales; Umbelopsidaceae; Umbelopsis; Umbelopsis_sp
New.ReferenceOTU3341	0	0	0	0	0	0	0	1	0	0	0	1	0	0	0	0	0	0	0	0	0	0	0	0	0	0	0	0	0	0	Fungi; Zygomycota; Incertae_sedis; Mucorales; unidentified; unidentified; Mucorales_sp
New.ReferenceOTU2439	1	0	0	1	0	0	0	0	0	0	0	0	0	0	0	0	0	0	0	0	0	0	0	0	0	0	0	0	0	0	Fungi; Ascomycota; Dothideomycetes; Incertae_sedis; Myxotrichaceae; Oidiodendron; Oidiodendron_griseum
New.ReferenceOTU3479	0	0	0	0	0	0	0	0	0	0	0	0	0	6	0	0	0	0	0	0	0	0	0	0	0	0	0	0	0	0	Fungi; Ascomycota; Dothideomycetes; Incertae_sedis; Myxotrichaceae; Oidiodendron; Oidiodendron_griseum
New.ReferenceOTU3715	0	0	0	1	4	0	1	0	0	0	0	0	2	0	0	0	0	0	0	0	0	0	0	0	0	3	1	0	0	1	Fungi; Ascomycota; Dothideomycetes; Incertae_sedis; Myxotrichaceae; Oidiodendron; Oidiodendron_griseum
New.ReferenceOTU764	0	0	1	0	0	0	0	0	0	0	0	0	0	0	0	0	0	0	0	0	0	0	0	0	0	0	0	0	0	0	Fungi; Ascomycota; Dothideomycetes; Incertae_sedis; Myxotrichaceae; Oidiodendron; Oidiodendron_griseum
New.ReferenceOTU6199	0	1	0	0	0	4	4	0	0	7	0	1	0	0	0	0	0	0	0	0	1	0	0	1	0	0	0	0	2	0	Fungi; Ascomycota; Dothideomycetes; Incertae_sedis; Myxotrichaceae; Oidiodendron; Oidiodendron_maius
New.ReferenceOTU92	0	0	1	0	0	0	0	0	0	0	0	0	0	0	0	0	0	0	0	0	0	0	0	0	0	0	0	0	0	0	Fungi; Ascomycota; Dothideomycetes; Incertae_sedis; Myxotrichaceae; Oidiodendron; Oidiodendron_maius
New.ReferenceOTU6134	0	0	0	1	0	0	0	0	0	0	0	0	0	0	0	0	0	0	0	0	0	0	0	0	0	0	0	0	0	0	Fungi; Ascomycota; Dothideomycetes; Incertae_sedis; Myxotrichaceae; Oidiodendron; Oidiodendron_pilicola
New.ReferenceOTU2404	0	4	0	0	0	0	0	0	0	0	0	0	0	0	0	0	0	11	0	0	0	0	0	10	0	0	0	0	1	0	Fungi; Ascomycota; Dothideomycetes; Incertae_sedis; Myxotrichaceae; Oidiodendron; Oidiodendron_sp
New.ReferenceOTU2713	0	0	0	0	0	1	0	0	2	2	2	0	2	0	1	0	0	0	1	0	0	0	0	0	0	0	0	0	0	0	Fungi; Ascomycota; Dothideomycetes; Incertae_sedis; Myxotrichaceae; Oidiodendron; Oidiodendron_sp
New.ReferenceOTU3822	0	0	0	0	0	0	0	0	0	0	0	0	0	0	0	0	0	0	3	0	0	0	0	0	0	0	0	0	0	0	Fungi; Ascomycota; Dothideomycetes; Incertae_sedis; Myxotrichaceae; Oidiodendron; Oidiodendron_sp
New.ReferenceOTU4317	0	0	0	10	3	3	0	0	0	2	2	1	0	0	0	0	0	6	0	1	0	0	0	2	0	0	0	0	1	0	Fungi; Ascomycota; Dothideomycetes; Incertae_sedis; Myxotrichaceae; Oidiodendron; Oidiodendron_sp
New.ReferenceOTU4425	0	6	0	0	0	0	0	0	0	0	0	0	0	0	0	0	0	0	0	0	0	0	0	0	0	0	0	0	0	0	Fungi; Ascomycota; Dothideomycetes; Incertae_sedis; Myxotrichaceae; Oidiodendron; Oidiodendron_sp
New.ReferenceOTU5106	0	0	0	0	0	0	0	0	3	0	0	0	0	0	0	0	0	0	0	0	0	0	0	0	0	0	0	0	0	0	Fungi; Ascomycota; Dothideomycetes; Incertae_sedis; Myxotrichaceae; Oidiodendron; Oidiodendron_sp
New.ReferenceOTU5499	0	0	1	2	5	0	0	0	3	1	9	0	0	0	2	1	0	0	0	1	0	0	0	0	0	0	0	0	0	0	Fungi; Ascomycota; Dothideomycetes; Incertae_sedis; Myxotrichaceae; Oidiodendron; Oidiodendron_sp
New.ReferenceOTU601	0	0	0	0	0	0	0	0	0	0	0	3	0	0	0	0	0	0	0	0	0	0	0	0	0	0	0	0	0	0	Fungi; Ascomycota; Dothideomycetes; Incertae_sedis; Myxotrichaceae; Oidiodendron; Oidiodendron_sp
New.ReferenceOTU7546	0	0	1	0	2	0	0	0	10	3	1	0	0	0	0	0	0	0	9	2	0	0	0	0	0	0	0	0	0	0	Fungi; Ascomycota; Dothideomycetes; Incertae_sedis; Myxotrichaceae; Oidiodendron; Oidiodendron_sp
New.ReferenceOTU7684	0	0	0	0	0	0	0	0	0	0	0	0	0	0	0	0	0	0	0	0	0	0	0	0	1	0	0	0	0	0	Fungi; Ascomycota; Dothideomycetes; Incertae_sedis; Myxotrichaceae; Oidiodendron; Oidiodendron_sp
New.ReferenceOTU7891	0	0	0	0	0	0	1	0	0	0	0	0	1	0	0	0	0	0	0	0	0	0	0	0	0	0	0	0	0	0	Fungi; Ascomycota; Dothideomycetes; Incertae_sedis; Myxotrichaceae; Oidiodendron; Oidiodendron_sp
New.ReferenceOTU2725	0	0	0	0	0	0	0	0	1	0	0	0	0	0	0	0	0	0	0	0	0	0	0	0	0	0	0	0	0	0	Fungi; Ascomycota; Dothideomycetes; Incertae_sedis; Myxotrichaceae; Oidiodendron; Oidiodendron_sp_07MA11
New.ReferenceOTU1598	0	0	0	0	0	0	0	0	0	0	0	0	0	2	0	5	0	0	0	0	0	0	0	0	0	0	0	0	0	0	Fungi; Ascomycota; Dothideomycetes; Incertae_sedis; Myxotrichaceae; Oidiodendron; Oidiodendron_sp_12216
New.ReferenceOTU2920	0	0	0	0	0	0	1	0	0	0	2	0	0	0	0	0	0	0	0	0	1	0	0	0	0	0	0	0	0	0	Fungi; Ascomycota; Dothideomycetes; Incertae_sedis; Myxotrichaceae; Oidiodendron; Oidiodendron_sp_12216
New.ReferenceOTU3051	0	0	0	0	0	0	0	0	0	3	0	0	0	0	0	0	0	0	0	0	0	0	0	0	0	0	0	0	0	0	Fungi; Ascomycota; Dothideomycetes; Incertae_sedis; Myxotrichaceae; Oidiodendron; Oidiodendron_sp_12216
New.ReferenceOTU4152	1	7	0	4	0	6	4	0	0	17	1	0	0	0	0	0	0	3	0	1	6	0	0	1	0	1	0	0	0	0	Fungi; Ascomycota; Dothideomycetes; Incertae_sedis; Myxotrichaceae; Oidiodendron; Oidiodendron_sp_12216
New.ReferenceOTU5371	3	0	0	0	0	0	0	0	0	0	0	0	0	0	0	0	0	0	0	0	0	0	0	0	0	0	0	0	0	0	Fungi; Ascomycota; Dothideomycetes; Incertae_sedis; Myxotrichaceae; Oidiodendron; Oidiodendron_sp_12216
New.ReferenceOTU6907	0	0	0	0	0	0	0	0	0	0	0	0	0	0	0	0	0	0	0	1	0	0	0	0	0	0	0	0	0	0	Fungi; Ascomycota; Dothideomycetes; Incertae_sedis; Myxotrichaceae; Oidiodendron; Oidiodendron_sp_12216
New.ReferenceOTU6939	1	0	1	0	5	0	0	0	1	8	0	0	2	3	1	1	4	23	1	2	3	0	1	5	0	1	0	0	4	0	Fungi; Ascomycota; Dothideomycetes; Incertae_sedis; Myxotrichaceae; Oidiodendron; Oidiodendron_sp_12216
New.ReferenceOTU7654	1	0	0	0	0	0	0	0	0	0	0	0	0	0	0	0	0	0	0	0	0	0	0	0	0	0	0	0	0	0	Fungi; Ascomycota; Dothideomycetes; Incertae_sedis; Myxotrichaceae; Oidiodendron; Oidiodendron_sp_12216
New.ReferenceOTU2008	0	0	0	0	1	0	0	0	0	0	0	0	0	0	0	0	0	5	0	0	0	0	0	0	0	0	0	0	0	0	Fungi; Ascomycota; Dothideomycetes; Incertae_sedis; Myxotrichaceae; Oidiodendron; Oidiodendron_sp_15PA18
New.ReferenceOTU2484	0	5	0	0	0	0	0	0	0	0	0	0	0	0	0	0	0	0	0	0	0	0	0	0	0	0	0	0	0	0	Fungi; Ascomycota; Dothideomycetes; Incertae_sedis; Myxotrichaceae; Oidiodendron; Oidiodendron_sp_18VA18
New.ReferenceOTU2550	0	0	0	0	0	0	0	0	1	1	0	0	0	0	0	0	0	0	2	0	0	0	0	0	0	0	0	0	0	0	Fungi; Ascomycota; Dothideomycetes; Incertae_sedis; Myxotrichaceae; Oidiodendron; Oidiodendron_sp_18VA18
New.ReferenceOTU4845	0	5	0	0	0	0	0	0	0	0	0	0	0	0	0	0	0	0	0	0	0	0	0	2	0	0	0	0	1	0	Fungi; Ascomycota; Dothideomycetes; Incertae_sedis; Myxotrichaceae; Oidiodendron; Oidiodendron_sp_18VA18
New.ReferenceOTU5771	0	7	0	0	0	0	0	0	0	0	0	0	0	0	0	0	0	0	0	0	0	0	0	1	0	0	0	1	0	0	Fungi; Ascomycota; Dothideomycetes; Incertae_sedis; Myxotrichaceae; Oidiodendron; Oidiodendron_sp_18VA18
New.ReferenceOTU1557	0	0	0	3	0	0	0	0	0	0	0	0	0	0	0	0	0	0	0	0	0	0	0	0	0	0	0	0	0	0	Fungi; Ascomycota; Dothideomycetes; Incertae_sedis; Myxotrichaceae; Oidiodendron; Oidiodendron_sp_3559
New.ReferenceOTU42	0	2	0	2	0	0	1	0	0	0	0	0	0	0	0	0	0	0	0	1	0	0	0	0	0	0	0	0	3	0	Fungi; Ascomycota; Dothideomycetes; Incertae_sedis; Myxotrichaceae; Oidiodendron; Oidiodendron_sp_3559
New.ReferenceOTU4977	0	0	0	0	0	0	0	0	0	0	0	0	0	0	0	0	0	0	0	0	0	0	0	0	0	0	0	0	1	0	Fungi; Ascomycota; Dothideomycetes; Incertae_sedis; Myxotrichaceae; Oidiodendron; Oidiodendron_sp_3559
New.ReferenceOTU6325	0	0	0	0	0	0	0	0	0	0	0	1	0	0	0	0	0	0	0	0	0	0	0	0	0	0	0	0	0	0	Fungi; Ascomycota; Dothideomycetes; Incertae_sedis; Myxotrichaceae; Oidiodendron; Oidiodendron_sp_3559
New.ReferenceOTU2959	0	3	0	0	0	0	0	0	0	0	0	0	0	0	0	0	0	0	0	0	0	0	0	0	0	0	0	0	0	0	Fungi; Ascomycota; Dothideomycetes; Incertae_sedis; Myxotrichaceae; Oidiodendron; Oidiodendron_sp_6808_S5
New.ReferenceOTU4448	0	0	0	0	0	0	0	0	0	0	0	0	0	0	0	0	0	3	0	0	0	0	0	0	0	0	0	0	0	0	Fungi; Ascomycota; Dothideomycetes; Incertae_sedis; Myxotrichaceae; Oidiodendron; Oidiodendron_sp_6808_S5
New.ReferenceOTU4893	0	0	0	2	0	2	0	0	0	0	0	0	0	0	0	0	0	0	0	0	0	0	0	9	0	0	0	1	4	0	Fungi; Ascomycota; Dothideomycetes; Incertae_sedis; Myxotrichaceae; Oidiodendron; Oidiodendron_sp_6808_S5
New.ReferenceOTU6659	0	0	0	0	0	0	0	0	0	0	0	0	0	0	1	0	0	1	0	0	0	0	0	0	0	0	0	0	0	0	Fungi; Ascomycota; Dothideomycetes; Incertae_sedis; Myxotrichaceae; Oidiodendron; Oidiodendron_sp_6808_S5
New.ReferenceOTU7782	0	0	0	0	1	0	0	0	0	0	0	0	0	0	0	0	0	0	0	0	0	0	0	0	0	0	0	0	0	0	Fungi; Ascomycota; Dothideomycetes; Incertae_sedis; Myxotrichaceae; Oidiodendron; Oidiodendron_sp_6808_S5
New.ReferenceOTU1019	0	0	0	2	0	0	0	0	0	0	0	0	0	0	0	0	0	0	0	0	0	0	0	0	0	0	0	0	0	0	Fungi; Ascomycota; Dothideomycetes; Incertae_sedis; Myxotrichaceae; Oidiodendron; Oidiodendron_sp_CY253
New.ReferenceOTU1422	11	0	0	0	0	0	0	0	0	0	0	0	0	0	0	0	0	0	0	0	0	0	0	0	0	0	0	0	0	0	Fungi; Ascomycota; Dothideomycetes; Incertae_sedis; Myxotrichaceae; Oidiodendron; Oidiodendron_sp_CY253
New.ReferenceOTU362	0	3	0	2	0	4	2	0	0	0	0	0	0	0	0	0	0	0	0	0	0	0	0	11	0	0	0	0	6	0	Fungi; Ascomycota; Dothideomycetes; Incertae_sedis; Myxotrichaceae; Oidiodendron; Oidiodendron_sp_CY253
New.ReferenceOTU6082	0	0	0	0	0	0	0	0	0	0	0	0	0	0	0	0	0	0	0	1	0	0	0	0	0	0	0	0	0	0	Fungi; Ascomycota; Dothideomycetes; Incertae_sedis; Myxotrichaceae; Oidiodendron; Oidiodendron_sp_CY253
New.ReferenceOTU6585	4	1	0	10	11	7	0	1	0	0	3	16	0	0	2	0	0	0	0	0	0	0	0	1	0	0	0	0	0	0	Fungi; Ascomycota; Dothideomycetes; Incertae_sedis; Myxotrichaceae; Oidiodendron; Oidiodendron_sp_CY253
New.ReferenceOTU941	0	0	0	0	0	0	0	0	0	1	0	0	0	0	0	0	0	0	0	0	0	0	0	0	0	0	0	0	0	0	Fungi; Ascomycota; Dothideomycetes; Incertae_sedis; Myxotrichaceae; Oidiodendron; Oidiodendron_sp_CY253
New.ReferenceOTU2118	0	0	0	0	0	0	0	0	0	0	0	1	0	0	0	0	0	0	0	0	0	0	0	1	0	0	0	0	1	0	Fungi; Ascomycota; Dothideomycetes; Incertae_sedis; Myxotrichaceae; Oidiodendron; Oidiodendron_sp_CZ_2011
New.ReferenceOTU6206	0	5	0	0	0	0	0	0	0	0	0	0	0	0	0	0	0	0	0	0	0	0	0	0	0	0	0	0	0	0	Fungi; Ascomycota; Dothideomycetes; Incertae_sedis; Myxotrichaceae; Oidiodendron; Oidiodendron_sp_CZ_2011
New.ReferenceOTU6601	0	0	0	0	2	0	0	0	0	0	0	0	0	0	0	0	0	0	0	0	0	0	0	0	0	0	0	0	0	0	Fungi; Ascomycota; Leotiomycetes; Helotiales; Incertae_sedis; Cadophora; Cadophora_luteo-olivacea
New.ReferenceOTU2090	0	0	0	0	0	0	0	0	0	0	0	0	0	0	0	0	0	0	0	0	0	0	0	0	0	0	0	0	1	0	Fungi; Ascomycota; Leotiomycetes; Helotiales; Incertae_sedis; Leptodontidium; Leptodontidium_sp
New.ReferenceOTU6449	0	0	0	0	0	0	0	3	1	3	0	0	2	1	0	3	1	3	0	0	0	0	1	0	0	0	0	0	0	3	Fungi; Ascomycota; Leotiomycetes; Helotiales; Vibrisseaceae; Phialocephala; Phialocephala_sp
New.ReferenceOTU6669	0	0	0	0	1	0	0	0	0	0	0	1	1	0	1	0	0	0	0	0	0	0	1	0	1	0	0	0	0	3	Fungi; Ascomycota; Leotiomycetes; Helotiales; Vibrisseaceae; Phialocephala; Phialocephala_sp
SH138846.06FU_AY273304_reps	3	7	7	2	0	0	16	13	7	12	2	4	4	12	3	14	7	1	5	2	0	0	2	15	2	0	2	1	0	9	Fungi; Ascomycota; Leotiomycetes; Helotiales; Vibrisseaceae; Phialocephala; Phialocephala_sp
New.ReferenceOTU1803	0	0	0	0	0	0	0	0	0	17	4	0	0	0	0	0	0	0	0	0	0	0	0	0	0	0	0	0	0	0	Fungi; Ascomycota; Sordariomycetes; Coniochaetales; Coniochaetaceae; Lecythophora; Lecythophora_sp

New.ReferenceOTU2907	0	0	1	0	0	0	0	0	0	0	0	0	0	0	0	0	0	0	0	0	0	0	0	0	0	0	0	0	0	0	Fungi; Ascomycota; Sordariomycetes; Coniochaetales; Coniochaetaceae; Lecythophora; Lecythophora_sp
New.ReferenceOTU2938	0	0	0	0	0	0	0	0	0	0	1	0	0	0	0	0	0	0	0	0	0	0	0	0	0	0	0	0	0	0	Fungi; Ascomycota; Sordariomycetes; Coniochaetales; Coniochaetaceae; Lecythophora; Lecythophora_sp
New.ReferenceOTU3234	0	0	0	0	0	0	1	0	0	2	0	2	0	0	1	1	0	0	0	0	0	0	0	0	0	0	0	0	0	0	Fungi; Ascomycota; Sordariomycetes; Coniochaetales; Coniochaetaceae; Lecythophora; Lecythophora_sp
New.ReferenceOTU5206	0	0	0	0	0	0	0	0	9	0	0	0	0	0	0	0	0	0	0	0	0	0	0	0	0	0	0	0	0	0	Fungi; Ascomycota; Sordariomycetes; Coniochaetales; Coniochaetaceae; Lecythophora; Lecythophora_sp
New.ReferenceOTU7149	0	0	0	0	0	0	0	0	0	0	0	0	0	0	0	0	0	0	0	1	0	0	0	0	0	0	0	0	0	0	Fungi; Ascomycota; Sordariomycetes; Coniochaetales; Coniochaetaceae; Lecythophora; Lecythophora_sp
New.ReferenceOTU260	0	0	0	0	0	0	0	0	0	2	0	0	0	0	0	0	0	0	0	0	0	0	0	0	0	0	0	0	0	0	Fungi; Basidiomycota; Tremellomycetes; Tremellales; Incertae_sedis; Tremella; Tremella_aurantialba
New.ReferenceOTU4474	0	0	0	3	0	0	0	0	0	3	1	0	0	3	0	0	0	0	0	0	0	1	0	1	0	1	0	0	0	0	Fungi; Basidiomycota; Tremellomycetes; Tremellales; Incertae_sedis; Tremella; Tremella_exigua
New.ReferenceOTU1804	0	0	0	1	0	0	0	0	0	0	0	0	0	0	0	0	0	17	0	0	0	0	0	0	0	0	0	0	1	0	Fungi; Basidiomycota; Tremellomycetes; Tremellales; Incertae_sedis; Tremella; Tremella_polyporina
New.ReferenceOTU2083	0	0	0	0	0	0	0	0	0	0	0	0	0	0	0	0	0	0	0	0	0	0	0	1	0	0	0	0	0	0	Fungi; Basidiomycota; Tremellomycetes; Tremellales; Incertae_sedis; Tremella; Tremella_sp
New.ReferenceOTU5494	0	0	0	0	0	0	0	0	0	0	0	0	0	0	0	0	0	0	2	0	0	0	0	0	0	0	0	0	0	0	Fungi; Basidiomycota; Tremellomycetes; Tremellales; Incertae_sedis; Tremella; Tremella_sp
SH024163.06FU_AB629029_reps_singleton	0	0	0	1	6	0	0	0	5	0	0	0	0	0	0	2	1	4	0	0	0	0	0	1	0	5	0	0	3	0	Fungi; Basidiomycota; Tremellomycetes; Tremellales; Incertae_sedis; Tremella; Tremella_sp
New.ReferenceOTU2115	0	0	0	0	0	0	0	0	0	0	0	0	0	0	0	0	0	0	0	0	0	0	0	0	0	0	0	0	0	1	Fungi; Basidiomycota; Tremellomycetes; Tremellales; Incertae_sedis; Fellomyces; Fellomyces_lichenicola
New.ReferenceOTU6988	0	0	0	0	0	0	0	2	0	0	0	0	0	0	0	0	0	0	0	0	0	0	0	0	0	0	0	0	0	0	Fungi; Basidiomycota; Tremellomycetes; Tremellales; Incertae_sedis; Fellomyces; Fellomyces_thailandicus
SH094198.06FU_AF444323_refs	0	0	0	0	0	0	1	0	0	0	0	0	0	0	0	0	6	0	0	0	0	0	0	0	0	0	1	0	0	0	Fungi; Basidiomycota; Tremellomycetes; Tremellales; Incertae_sedis; Hannaella; Hannaella_luteola
New.ReferenceOTU7052	0	0	0	0	0	0	0	0	1	0	0	0	0	0	0	0	0	0	0	1	0	0	0	0	0	0	0	0	0	0	Fungi; Basidiomycota; Tremellomycetes; Tremellales; Incertae_sedis; Kockovaella; Kockovaella_schimae
SH005380.06FU_FN428915_reps_singleton	0	0	0	0	0	0	0	0	0	0	0	0	0	0	1	0	1	0	0	0	0	0	0	0	0	0	0	0	0	0	Fungi; Basidiomycota; Tremellomycetes; Tremellales; Incertae_sedis; unidentified; Tremellales_sp
SH094199.06FU_KC193595_reps	0	0	0	0	0	0	0	0	0	0	0	0	0	0	0	1	1	0	0	0	0	0	0	0	0	0	0	0	0	0	Fungi; Basidiomycota; Tremellomycetes; Tremellales; Incertae_sedis; unidentified; Tremellales_sp
New.ReferenceOTU3395	0	0	0	0	0	0	0	0	0	0	0	0	0	0	0	0	0	3	0	0	0	0	0	0	0	0	0	0	0	0	Fungi; Basidiomycota; Tremellomycetes; Tremellales; unidentified; unidentified; Tremellales_sp
New.ReferenceOTU3489	0	0	0	0	0	0	0	0	0	0	0	0	1	0	0	0	0	0	0	0	0	0	0	0	0	0	0	0	0	0	Fungi; Basidiomycota; Tremellomycetes; Tremellales; unidentified; unidentified; Tremellales_sp
New.ReferenceOTU454	0	0	0	0	0	0	0	0	0	0	0	0	0	0	0	1	0	1	0	0	0	0	0	0	0	0	0	0	0	0	Fungi; Basidiomycota; Tremellomycetes; Tremellales; unidentified; unidentified; Tremellales_sp
New.ReferenceOTU556	0	0	0	0	0	0	0	0	0	0	0	0	0	0	0	0	0	14	0	0	0	0	0	0	0	0	0	0	0	0	Fungi; Basidiomycota; Tremellomycetes; Tremellales; unidentified; unidentified; Tremellales_sp
New.ReferenceOTU5815	0	0	0	0	0	0	0	0	0	0	0	0	0	0	1	0	0	0	0	0	0	0	0	0	0	0	0	0	0	0	Fungi; Basidiomycota; Tremellomycetes; Tremellales; unidentified; unidentified; Tremellales_sp
SH024164.06FU_AB507852_reps_singleton	0	1	0	0	0	0	0	0	0	0	0	0	0	0	0	0	0	1	0	0	0	0	0	0	0	0	0	0	0	0	Fungi; Basidiomycota; Tremellomycetes; Tremellales; unidentified; unidentified; Tremellales_sp
SH024165.06FU_FJ528729_reps_singleton	0	0	0	0	3	0	0	0	0	0	0	0	0	1	0	0	0	1	0	0	0	0	0	0	0	0	0	0	0	0	Fungi; Basidiomycota; Tremellomycetes; Tremellales; unidentified; unidentified; Tremellales_sp
New.ReferenceOTU3579	0	0	0	0	0	0	0	0	0	0	0	0	0	0	0	0	0	2	0	0	0	0	0	0	0	0	0	0	0	0	Fungi; Basidiomycota; Tremellomycetes; Tremellales; unidentified; unidentified; Tremellales_sp_LH28
New.ReferenceOTU4342	0	0	0	0	0	0	0	0	0	0	0	0	0	0	0	0	0	3	0	0	0	0	0	0	0	0	0	0	0	0	Fungi; Basidiomycota; Tremellomycetes; Tremellales; unidentified; unidentified; Tremellales_sp_LH28
New.ReferenceOTU8046	0	0	0	0	0	0	0	0	0	0	0	0	0	0	0	0	0	0	0	0	0	0	0	0	1	0	0	0	0	0	Fungi; Basidiomycota; Tremellomycetes; Tremellales; unidentified; unidentified; Tremellales_sp_TG05
New.ReferenceOTU6673	0	5	0	0	0	0	0	0	0	0	0	0	0	0	0	0	0	0	0	0	0	0	0	0	0	0	0	0	0	0	Fungi; Basidiomycota; unidentified; unidentified; unidentified; unidentified; Basidiomycota_cf_Tremellales_OTU_033
New.ReferenceOTU2580	0	0	0	0	0	0	0	1	0	0	0	0	0	0	0	1	3	0	0	0	0	0	0	0	0	0	0	0	0	0	Fungi; Ascomycota; Dothideomycetes; Pleosporales; Incertae_sedis; Periconia; Periconia_sp
SH135985.06FU_JX984754_reps	0	0	0	0	0	0	0	1	0	0	0	0	0	0	0	2	1	0	0	0	0	1	0	1	0	0	0	0	0	0	Fungi; Ascomycota; Dothideomycetes; Pleosporales; Incertae_sedis; Periconia; Periconia_sp
SH093492.06FU_JX982370_reps	0	0	0	0	0	0	0	0	0	0	0	0	0	0	0	0	0	0	0	0	0	0	1	0	0	0	0	0	0	0	Fungi; Ascomycota; Dothideomycetes; Pleosporales; Incertae_sedis; Phoma; Phoma_exigua
SH100181.06FU_GU237724_reps	0	0	0	0	0	0	0	0	0	0	0	0	0	0	6	0	0	0	0	0	0	1	11	0	0	0	2	0	0	3	Fungi; Ascomycota; Dothideomycetes; Pleosporales; Incertae_sedis; Phoma; Phoma_exigua
New.CleanUp.ReferenceOTU1866	0	0	0	0	0	0	0	0	0	0	1	0	0	0	0	0	0	0	0	0	0	0	0	0	0	0	0	0	0	0	Fungi; Ascomycota; Dothideomycetes; Pleosporales; Incertae_sedis; Phoma; Phoma_sp
New.ReferenceOTU1365	0	0	0	0	0	0	0	0	0	0	0	0	0	0	0	0	1	0	0	0	0	0	0	0	0	0	0	0	0	0	Fungi; Ascomycota; Dothideomycetes; Pleosporales; Incertae_sedis; Phoma; Phoma_sp
New.ReferenceOTU2983	0	0	0	0	0	3	23	0	0	1	0	1	0	1	0	3	0	0	0	0	0	0	0	0	0	0	0	0	0	0	Fungi; Ascomycota; Dothideomycetes; Pleosporales; Incertae_sedis; Phoma; Phoma_sp
New.ReferenceOTU3203	0	0	0	0	0	0	0	0	0	0	0	0	0	0	0	0	0	0	0	0	0	0	1	0	0	0	0	0	0	0	Fungi; Ascomycota; Dothideomycetes; Pleosporales; Incertae_sedis; Phoma; Phoma_sp
New.ReferenceOTU4015	0	0	0	0	0	0	0	0	0	0	0	0	0	0	0	0	0	0	0	0	0	0	1	0	0	0	0	0	0	0	Fungi; Ascomycota; Dothideomycetes; Pleosporales; Incertae_sedis; Phoma; Phoma_sp
New.ReferenceOTU5810	0	0	0	0	0	0	0	0	0	0	0	0	0	0	8	0	0	0	0	0	4	0	2	0	2	0	4	0	0	4	Fungi; Ascomycota; Dothideomycetes; Pleosporales; Incertae_sedis; Phoma; Phoma_sp
New.ReferenceOTU7440	0	0	0	0	0	0	0	0	0	0	0	0	0	0	0	0	0	0	0	0	0	0	0	0	1	0	0	0	0	0	Fungi; Ascomycota; Dothideomycetes; Pleosporales; Incertae_sedis; Phoma; Phoma_sp
SH004255.06FU_AF502664_reps_singleton	1	0	0	18	0	0	0	0	0	0	0	0	0	0	0	0	0	0	0	0	0	0	1	0	7	0	0	3	0	0	Fungi; Ascomycota; Dothideomycetes; Pleosporales; Incertae_sedis; Phoma; Phoma_sp
SH004259.06FU_AF502832_reps_singleton	0	0	0	0	1	0	0	0	0	0	0	0	0	0	0	0	0	0	0	0	0	0	0	0	0	0	0	0	0	0	Fungi; Ascomycota; Dothideomycetes; Pleosporales; Incertae_sedis; Phoma; Phoma_sp
SH004265.06FU_AF502627_reps_singleton	0	0	0	0	0	0	0	0	0	0	0	0	0	0	0	0	0	0	0	0	0	0	0	0	1	0	0	0	0	0	Fungi; Ascomycota; Dothideomycetes; Pleosporales; Incertae_sedis; Phoma; Phoma_sp
SH004266.06FU_AF502770_reps_singleton	0	0	0	0	0	1	1	0	0	0	0	0	0	1	0	1	0	0	0	0	0	0	0	0	1	0	0	0	0	0	Fungi; Ascomycota; Dothideomycetes; Pleosporales; Incertae_sedis; Phoma; Phoma_sp
SH104585.06FU_JQ905798_reps	1	0	0	0	0	0	0	0	0	0	0	0	0	1	0	0	2	0	0	0	0	0	0	0	0	0	0	0	0	0	Fungi; Ascomycota; Dothideomycetes; Pleosporales; Incertae_sedis; Phoma; Phoma_sp
SH126642.06FU_JQ922156_reps	0	0	0	0	0	0	0	0	0	0	0	1	0	0	10	0	0	0	0	0	0	0	1	0	20	0	3	0	0	4	Fungi; Ascomycota; Dothideomycetes; Pleosporales; Incertae_sedis; Phoma; Phoma_sp
SH126644.06FU_JX174139_reps	0	0	0	0	0	0	0	0	0	1	0	0	0	0	1	0	0	0	0	0	1	0	0	0	1	0	1	0	0	0	Fungi; Ascomycota; Dothideomycetes; Pleosporales; Incertae_sedis; Phoma; Phoma_sp
New.ReferenceOTU5750	2	0	3	0	0	0	0	0	0	1	0	0	0	1	0	1	0	0	0	0	0	0	0	0	0	0	0	0	0	0	Fungi; Ascomycota; Dothideomycetes; Pleosporales; Incertae_sedis; Phoma; Phoma_sp_20_2
New.ReferenceOTU6207	0	0	3	0	0	0	0	0	0	0	0	0	0	0	0	2	0	0	0	0	0	0	0	5	0	0	0	0	0	0	Fungi; Ascomycota; Dothideomycetes; Pleosporales; Incertae_sedis; Phoma; Phoma_sp_20_2
SH094616.06FU_GU721704_reps	0	0	0	0	0	0	0	0	0	0	0	0	0	0	0	0	0	0	0	0	0	0	0	0	0	0	0	0	0	1	Fungi; Ascomycota; Dothideomycetes; Pleosporales; Incertae_sedis; Phoma; Phoma_sp_878_GP_2013
New.ReferenceOTU7025	0	0	0	0	0	0	0	0	0	1	0	0	0	0	0	0	0	0	0	0	0	0	0	0	0	0	0	0	0	0	Fungi; Ascomycota; Dothideomycetes; Pleosporales; Leptosphaeriaceae; Coniothyrium; Coniothyrium_nitidae
New.ReferenceOTU1314	0	0	0	0	3	4	0	0	2	0	0	0	1	0	0	0	1	0	0	0	0	0	3	0	0	0	1	0	0	1	Fungi; Ascomycota; Dothideomycetes; Pleosporales; Leptosphaeriaceae; Coniothyrium; Coniothyrium_sp_FF_2011
New.ReferenceOTU7846	0	0	0	0	0	0	0	0	0	0	0	0	0	0	0	0	0	0	0	0	0	0	0	0	0	0	0	0	0	1	Fungi; Ascomycota; Dothideomycetes; Pleosporales; Leptosphaeriaceae; Coniothyrium; Coniothyrium_sp_FF_2011
SH024934.06FU_HE584866_reps_singleton	0	0	0	0	7	16	5	1	0	0	0	1	8	0	4	0	0	0	0	0	0	0	8	1	1	0	0	5	0	1	Fungi; Ascomycota; Dothideomycetes; Pleosporales; Leptosphaeriaceae; Coniothyrium; Coniothyrium_sp_FF_2011
SH126627.06FU_JQ905822_reps	0	0	0	0	1	7	4	0	2	0	0	0	6	0	1	1	0	0	0	0	0	0	8	2	0	0	0	1	0	3	Fungi; Ascomycota; Dothideomycetes; Pleosporales; Leptosphaeriaceae; Coniothyrium; Coniothyrium_sp_FF_2011
New.ReferenceOTU2915	0	0	0	0	0	0	0	0	0	0	0	0	0	0	0	3	0	0	0	0	0	0	0	0	0	0	0	0	0	0	Fungi; Ascomycota; Incertae_sedis; Incertae_sedis; Incertae_sedis; Chalara; Chalara_sp_YTH_2012a
New.ReferenceOTU5555	0	0	0	0	0	0	0	0	0	0	0	0	0	0	0	0	0	2	0	0	0	0	0	0	0	0	0	0	0	0	Fungi; Ascomycota; Incertae_sedis; Incertae_sedis; Incertae_sedis; Cordana; Cordana_ellipsoidea
SH121053.06FU_HE672145_reps	0	0	0	0	0	0	0	0	0	0	1	0	0	0	0	0	0	0	0	0	0	0	0	0	0	0	1	1	0	0	Fungi; Ascomycota; Incertae_sedis; Incertae_sedis; Incertae_sedis; Cordana; Cordana_ellipsoidea
New.ReferenceOTU3378	0	0	0	2	0	0	0	0	0	0	0	0	0	0	0	0	0	0	0	0	0	0	0	0	0	0	0	0	0	0	Fungi; Ascomycota; Incertae_sedis; Incertae_sedis; Incertae_sedis; Cordana; Cordana_solitaria
New.ReferenceOTU637	0	0	1	0	0	0	0	0	0	0	0	0	0	0	0	0	0	0	0	0	0	0	0	0	0	0	0	0	0	0	Fungi; Ascomycota; Incertae_sedis; Incertae_sedis; Incertae_sedis; Cordana; Cordana_solitaria
New.ReferenceOTU1642	0	0	0	0	0	0	0	0	0	0	0	0	0	0	1	0	0	0	0	0	0	0	0	0	0	0	0	0	0	0	Fungi; Ascomycota; Incertae_sedis; Incertae_sedis; Incertae_sedis; Cordana; Cordana_sp_HRM_2012
New.ReferenceOTU3571	0	0	0	0	0	0	0	0	0	0	0	0	0	0	0	0	0	0	1	0	0	0	0	0	0	0	0	0	0	0	Fungi; Ascomycota; Sordariomycetes; Diaporthales; Diaporthaceae; Phomopsis; Phomopsis_sp
SH091186.06FU_HM211270_reps	0	0	0	0	0	0	0	0	0	0	0	1	0	0	0	0	0	0	0	0	0	0	0	0	0	0	0	0	0	1	Fungi; Ascomycota; Sordariomycetes; Diaporthales; Diaporthaceae; Phomopsis; Phomopsis_sp
SH025659.06FU_AF210667_reps_singleton	0	0	0	0	0	0	0	0	0	0	0	0	0	0	0	0	0	0	0	0	0	0	0	2	0	0	0	0	0	0	Fungi; Ascomycota; Sordariomycetes; Hypocreales; Bionectriaceae; Bionectria; Bionectria_coronata
New.ReferenceOTU2061	0	3	0	0	0	0	0	0	0	0	0	0	0	0	0	0	0	0	0	0	0	0	0	0	0	0	0	0	0	0	Fungi; Ascomycota; Sordariomycetes; Hypocreales; Bionectriaceae; Bionectria; Bionectria_sp
New.ReferenceOTU6962	2	0	0	0	0	0	0	0	0	1	0	0	0	0	0	0	0	0	0	0	0	0	0	0	0	0	0	0	0	0	Fungi; Ascomycota; Sordariomycetes; Hypocreales; Bionectriaceae; Bionectria; Bionectria_sp_ASR_301
New.ReferenceOTU7008	0	0	0	0	0	0	0	0	0	0	0	0	0	1	0	0	0	0	0	1	0	0	0	0	0	0	0	0	0	1	Fungi; Ascomycota; Sordariomycetes; Hypocreales; Bionectriaceae; Bionectria; Bionectria_sp_ASR_301
New.ReferenceOTU8018	0	0	0	1	0	0	0	0	0	0	0	0	0	0	0	0	0	0	0	0	0	0	0	0	0	0	0	0	0	0	Fungi; Ascomycota; Sordariomycetes; Hypocreales; Bionectriaceae; Bionectria; Bionectria_zelandiaenovae
SH033764.06FU_KC787036_reps_singleton	0	0	0	0	0	0	0	0	0	0	0	0	0	0	0	0	0	0	1	0	0	0	0	0	0	0	0	0	0	0	Fungi; Ascomycota; Sordariomycetes; Hypocreales; Bionectriaceae; Clonostachys; Bionectria_sp_MIAE01299
New.ReferenceOTU2330	0	0	0	0	0	0	0	0	0	1	0	0	0	0	0	0	0	0	1	0	0	0	0	0	0	0	0	0	0	0	Fungi; Ascomycota; Sordariomycetes; Hypocreales; Hypocreaceae; Gliocladium; Gliocladium_cibotii
New.ReferenceOTU4103	0	0	1	0	0	0	0	0	0	0	0	0	0	0	0	0	0	0	0	0	0	2	1	0	1	0	0	0	0	0	Fungi; Ascomycota; Sordariomycetes; Hypocreales; Hypocreaceae; Gliocladium; Gliocladium_sp_NRRL_22971
SH027235.06FU_JN093257_reps_singleton	0	0	1	0	1	0	0	0	0	0	0	0	0	0	0	1	0	0	0	0	0	0	0	0	0	0	0	0	0	0	Fungi; Ascomycota; Sordariomycetes; Hypocreales; Hypocreaceae; Gliocladium; Gliocladium_sp_NRRL_22971
New.ReferenceOTU1455	0	1	0	0	0	0	0	0	0	0	0	0	0	0	3	1	0	0	0	0	3	2	1	0	4	0	11	0	0	3	Fungi; Ascomycota; Sordariomycetes; Hypocreales; Nectriaceae; Fusarium; Fusarium_oxysporum
New.ReferenceOTU4454	0	0	0	0	0	0	0	0	0	0	0	0	0	0	1	0	0	0	0	0	0	0	0	0	0	0	0	0	0	0	Fungi; Ascomycota; Sordariomycetes; Hypocreales; Nectriaceae; Fusarium; Fusarium_oxysporum
SH019714.06FU_DQ790553_reps_singleton	0	0	0	0	0	0	0	0	0	0	0	0	0	0	0	0	0	0	0	0	0	0	0	0	0	0	2	0	0	0	Fungi; Ascomycota; Sordariomycetes; Hypocreales; Nectriaceae; Fusarium; Fusarium_oxysporum
SH019737.06FU_KC428399_reps_singleton	0	0	0	0	0	0	0	0	0	0	0	0	0	0	1	0	0	0	0	0	3	0	0	0	1	0	4	0	0	1	Fungi; Ascomycota; Sordariomycetes; Hypocreales; Nectriaceae; Fusarium; Fusarium_oxysporum
SH118559.06FU_DQ452451_refs	0	0	2	0	0	0	1	0	0	0	0	0	0	4	8	0	0	0	0	0	26	7	16	0	9	0	24	0	0	16	Fungi; Ascomycota; Sordariomycetes; Hypocreales; Nectriaceae; Fusarium; Fusarium_oxysporum
SH118623.06FU_DQ655714_reps	0	0	0	0	0	0	1	0	0	0	0	0	0	1	15	3	0	0	0	0	19	22	12	0	20	0	24	2	0	18	Fungi; Ascomycota; Sordariomycetes; Hypocreales; Nectriaceae; Fusarium; Fusarium_oxysporum
SH118663.06FU_JQ039331_reps	0	0	0	0	0	0	0	0	0	0	0	0	0	0	1	0	0	0	0	0	0	1	0	0	0	0	1	0	0	0	Fungi; Ascomycota; Sordariomycetes; Hypocreales; Nectriaceae; Fusarium; Fusarium_oxysporum
SH143537.06FU_EU336986_reps	0	0	0	0	0	0	0	0	0	0	0	1	0	0	1	0	0	0	0	0	5	1	1	0	3	0	10	0	0	1	Fungi; Ascomycota; Sordariomycetes; Hypocreales; Nectriaceae; Fusarium; Fusarium_oxysporum
New.CleanUp.ReferenceOTU190	0	0	0	0	0	0	0	0	1	0	0	0	0	0	0	0	0	0	0	0	0	0	0	0	0	0	0	0	0	0	Fungi; Ascomycota; Sordariomycetes; Hypocreales; Nectriaceae; Fusarium; Fusarium_sp
New.ReferenceOTU2392	0	0	0	0	0	0	0	0	0	0	0	0	0	0	0	0	0	0	0	1	0	0	0	0	0	0	0	0	0	0	Fungi; Ascomycota; Sordariomycetes; Hypocreales; Nectriaceae; Fusarium; Fusarium_sp
New.ReferenceOTU2847	0	0	0	0	0	0	0	0	0	0	0	0	0	0	0	0	0	1	0	0	0	0	0	0	0	0	0	0	0	0	Fungi; Ascomycota; Sordariomycetes; Hypocreales; Nectriaceae; Fusarium; Fusarium_sp
New.ReferenceOTU3569	0	0	0	0	0	0	0	0	0	10	0	0	0	0	0	0	0	1	0	2	0	0	0	0	0	0	0	0	0	0	Fungi; Ascomycota; Sordariomycetes; Hypocreales; Nectriaceae; Fusarium; Fusarium_sp
New.ReferenceOTU3884	0	0	0	0	0	0	0	0	0	0	4	0	0	0	0	0	0	0	0	0	0	0	0	0	0	0	0	0	0	0	Fungi; Ascomycota; Sordariomycetes; Hypocreales; Nectriaceae; Fusarium; Fusarium_sp
New.ReferenceOTU4102	0	0	0	0	0	0	0	0	0	0	0	0	0	2	0	0	0	0	0	0	0	0	0	0	0	0	0	0	0	0	Fungi; Ascomycota; Sordariomycetes; Hypocreales; Nectriaceae; Fusarium; Fusarium_sp
SH112166.06FU_JX155888_reps	0	0	3	0	4	0	0	0	0	0	0	0	0	0	0	0	0	0	0	0	0	0	0	0	0	0	0	0	0	0	Fungi; Ascomycota; Sordariomycetes; Hypocreales; Nectriaceae; Fusarium; Fusarium_sp
New.ReferenceOTU6835	0	0	0	0	0	0	0	0	0	0	0	0	0	0	0	0	0	0	0	3	0	0	0	0	0	0	0	0	0	0	Fungi; Ascomycota; Sordariomycetes; Hypocreales; Nectriaceae; Fusarium; Fusarium_sp_419
New.ReferenceOTU1385	0	0	12	0	0	0	0	0	0	0	0	0	0	0	0	1	0	0	0	0	0	0	0	0	1	0	0	0	0	0	Fungi; Ascomycota; Sordariomycetes; Hypocreales; Nectriaceae; Fusarium; Fusarium_sp_9mpc
New.ReferenceOTU6218	0	0	0	0	0	0	0	0	0	0	0	0	0	0	0	0	0	0	0	0	4	4	0	0	5	0	0	0	0	0	Fungi; Ascomycota; Sordariomycetes; Hypocreales; Nectriaceae; Fusarium; Fusarium_sp_9mpc
SH024516.06FU_JQ922188_reps_singleton	0	0	0	0	0	0	0	0	0	0	0	1	0	0	0	0	0	0	0	0	0	0	0	0	0	0	0	1	0	0	Fungi; Ascomycota; Sordariomycetes; Hypocreales; Nectriaceae; Fusarium; Fusarium_sp_CCG_2012
New.ReferenceOTU7783	0	0	0	2	0	0	0	0	0	0	0	0	0	2	0	2	0	0	0	0	0	0	0	0	0	0	0	0	0	0	Fungi; Ascomycota; Sordariomycetes; Hypocreales; Nectriaceae; Fusarium; Fusarium_sp_CY246
SH019751.06FU_HQ608055_reps_singleton	0	0	0	2	0	0	0	0	0	0	0	0	0	0	0	0	0	0	0	0	0	0	0	0	0	0	0	0	0	0	Fungi; Ascomycota; Sordariomycetes; Hypocreales; Nectriaceae; Fusarium; Fusarium_sp_CY246
New.ReferenceOTU899	0	0	0	0	0	0	0	0	0	0	0	0	0	0	0	0	0	0	0	0	0	1	0	0	0	0	0	0	0	0	Fungi; Ascomycota; Sordariomycetes; Hypocreales; Nectriaceae; Fusarium; Fusarium_sp_FRC_S1760
SH137915.06FU_JF323002_reps	0	0	0	0	0	0	0	0	0	0	0	0	0	0	0	0	0	0	0	0	0	0	0	0	1	0	2	0	0	2	Fungi; Ascomycota; Sordariomycetes; Hypocreales; Nectriaceae; Fusarium; Fusarium_sp_FRC_S1760
SH137949.06FU_JQ364970_reps	0	0	0	0	0	0	0	0	0	0	0	0	0	0	0	0	0	0	0	0	0	0	0	0	1	0	2	0	0	2	Fungi; Ascomycota; Sordariomycetes; Hypocreales; Nectriaceae; Fusarium; Fusarium_sp_FRC_S1760
New.ReferenceOTU4428	0	0	0	0	0	0	0	0	0	0	0	0	0	0	0	0	0	0	0	0	0	1	0	0	0	0	0	0	0	0	Fungi; Ascomycota; Sordariomycetes; Hypocreales; Nectriaceae; Fusarium; Fusarium_sp_FRC_S1925
New.ReferenceOTU4205	0	0	0	0	0	0	0	0	0	0	0	0	0	0	0	0	0	0	0	0	0	0	1	0	0	0	0	0	0	0	Fungi; Ascomycota; Sordariomycetes; Hypocreales; Nectriaceae; Fusarium; Fusarium_sp_MS294
New.ReferenceOTU6010	3	0	0	0	0	0	0	0	4	0	1	0	0	0	0	0	0	0	0	0	0	0	0	0	0	0	0	1	0	4	Fungi; Ascomycota; Sordariomycetes; Hypocreales; Nectriaceae; Fusarium; Fusarium_sp_MS294
New.ReferenceOTU2196	0	0	0	0	0	0	0	1	0	0	0	0	0	0	0	0	0	0	0	0	0	0	0	0	0	0	0	0	0	0	Fungi; Ascomycota; Sordariomycetes; Hypocreales; Nectriaceae; Fusarium; Fusarium_sp_MX271
New.ReferenceOTU4044	0	0	0	2	0	0	0	0	0	0	0	0	0	0	1	0	0	0	0	0	0	2	0	0	0	0	0	1	0	0	Fungi; Ascomycota; Sordariomycetes; Hypocreales; Nectriaceae; Fusarium; Fusarium_sp_NRRL_28541

SH032325.06FU_EU329684_reps_singleton	0	0	0	0	0	0	0	0	0	0	0	0	0	0	0	0	0	0	0	0	0	1	0	0	0	0	0	11	0	0	Fungi; Ascomycota; Sordariomycetes; Hypocreales; Nectriaceae; Fusarium; Fusarium_sp_NRRL_28541
SH137914.06FU_HM211219_reps	0	0	0	14	0	0	0	0	0	0	0	0	0	0	0	1	0	0	0	22	0	9	0	0	6	6	1	115	0	0	Fungi; Ascomycota; Sordariomycetes; Hypocreales; Nectriaceae; Fusarium; Fusarium_sp_NRRL_28541
New.ReferenceOTU6212	0	0	0	0	0	0	0	0	0	0	0	0	0	0	0	0	0	0	0	0	0	1	0	0	0	0	0	0	0	0	Fungi; Ascomycota; Sordariomycetes; Hypocreales; Nectriaceae; Fusarium; Fusarium_sp_NRRL_46703
SH137912.06FU_AY569560_reps	0	0	8	0	0	1	3	2	0	0	0	3	0	0	9	1	0	0	3	1	2	2	1	2	8	0	1	19	0	6	Fungi; Ascomycota; Sordariomycetes; Hypocreales; Nectriaceae; Fusarium; Fusarium_sp_NRRL_46703
SH032195.06FU_JN604449_reps_singleton	0	0	0	0	0	0	0	0	0	0	0	0	0	0	0	0	0	0	0	0	0	0	0	0	1	0	0	0	0	0	Fungi; Ascomycota; Sordariomycetes; Microascales; Ceratocystidaceae; Ceratocystis; Ceratocystis_paradoxa
SH137832.06FU_FN668726_reps	0	0	0	0	0	0	0	0	0	0	0	0	0	0	1	0	0	0	0	0	2	9	7	0	2	0	0	1	0	0	Fungi; Ascomycota; Sordariomycetes; Microascales; Ceratocystidaceae; Ceratocystis; Ceratocystis_paradoxa
New.ReferenceOTU172	0	0	0	0	0	8	0	0	0	0	0	0	0	0	0	1	0	0	0	0	0	0	0	0	0	0	0	0	0	0	Fungi; Ascomycota; Sordariomycetes; Microascales; Ceratocystidaceae; Ceratocystis; Ceratocystis_sp
New.ReferenceOTU2332	0	0	0	0	0	0	0	1	0	0	0	0	0	0	0	0	0	0	0	0	0	0	0	0	0	0	0	0	0	0	Fungi; Ascomycota; Sordariomycetes; Microascales; Ceratocystidaceae; Ceratocystis; Ceratocystis_sp
New.ReferenceOTU7237	0	0	0	0	0	0	0	0	0	0	0	0	0	0	0	0	0	0	0	2	0	0	0	0	0	0	0	0	0	0	Fungi; Basidiomycota; Agaricomycetes; Agaricales; Tricholomataceae; Clitocybe; Clitocybe_hesleri
New.CleanUp.ReferenceOTU1618	0	0	0	0	0	0	0	0	0	0	0	0	0	0	0	0	0	0	1	0	0	0	0	0	0	0	0	0	0	0	Fungi; Basidiomycota; Agaricomycetes; Hymenochaetales; Hymenochaetaceae; Phellinus; Phellinus_repandus
New.ReferenceOTU87	0	0	0	0	0	3	0	0	0	0	0	0	0	0	0	0	0	0	0	0	0	0	0	0	0	0	0	0	0	0	Fungi; Basidiomycota; Agaricomycetes; Hymenochaetales; Hymenochaetaceae; Phellinus; Phellinus_sp_DLL2010_102
New.ReferenceOTU2575	1	0	0	0	0	0	0	0	2	0	1	0	0	0	0	0	0	0	3	4	0	0	0	0	0	0	0	0	0	0	Fungi; Basidiomycota; Agaricomycetes; Polyporales; Ganodermataceae; Ganoderma; Ganoderma_australe
SH136561.06FU_JX195205_reps	0	0	0	0	0	0	0	0	0	0	0	0	0	0	0	0	0	0	0	1	0	0	0	0	0	0	0	0	0	0	Fungi; Basidiomycota; Agaricomycetes; Polyporales; Ganodermataceae; Ganoderma; Ganoderma_australe
New.ReferenceOTU2520	0	0	0	0	0	0	0	0	0	0	0	0	0	0	1	0	0	0	2	0	0	0	0	2	0	1	0	0	3	0	Fungi; Basidiomycota; Agaricomycetes; Polyporales; Ganodermataceae; Ganoderma; Ganoderma_cupreum
SH116459.06FU_AY569450_reps	1	1	0	0	0	0	0	0	0	1	0	0	0	0	0	0	0	0	0	0	0	0	0	0	0	1	0	1	0	0	Fungi; Basidiomycota; Agaricomycetes; Polyporales; Ganodermataceae; Ganoderma; Ganoderma_cupreum
New.ReferenceOTU2784	1	0	0	0	0	0	0	0	0	0	0	0	0	0	0	0	0	0	0	0	0	0	0	0	0	0	0	0	0	0	Fungi; Basidiomycota; Agaricomycetes; Polyporales; Ganodermataceae; Ganoderma; Ganoderma_japonicum
New.ReferenceOTU6553	0	2	0	0	0	0	0	0	0	0	0	0	0	0	0	0	0	0	0	0	0	0	0	0	0	0	0	0	0	0	Fungi; Basidiomycota; Agaricomycetes; Polyporales; Ganodermataceae; Ganoderma; Ganoderma_resinaceum
New.ReferenceOTU4472	1	0	0	1	0	0	0	0	0	0	0	0	0	0	0	0	0	0	0	0	0	0	0	0	0	0	0	0	0	0	Fungi; Basidiomycota; Agaricomycetes; Polyporales; Ganodermataceae; Ganoderma; Ganoderma_sp_E7094
SH116457.06FU_FJ392283_reps	1	0	0	4	0	0	0	0	0	0	0	0	0	0	0	0	0	0	0	0	0	0	0	0	0	0	0	0	0	0	Fungi; Basidiomycota; Agaricomycetes; Polyporales; Ganodermataceae; Ganoderma; Ganoderma_sp_E7094
New.ReferenceOTU2253	0	0	0	0	0	0	0	0	0	1	0	0	0	0	0	0	0	0	7	0	0	0	0	0	0	0	0	0	0	0	Fungi; Basidiomycota; Agaricomycetes; Polyporales; Ganodermataceae; Ganoderma; Ganoderma_sp_FRIM_138
New.ReferenceOTU3062	0	0	0	0	0	0	0	0	0	0	0	0	0	0	0	0	0	0	5	0	0	0	0	0	0	0	0	0	0	0	Fungi; Basidiomycota; Agaricomycetes; Polyporales; Ganodermataceae; Ganoderma; Ganoderma_sp_FRIM_138
New.ReferenceOTU4649	0	0	0	0	0	0	0	0	0	0	0	0	0	0	0	0	0	0	1	0	0	0	0	0	0	0	0	0	0	0	Fungi; Basidiomycota; Agaricomycetes; Polyporales; Ganodermataceae; Ganoderma; Ganoderma_sp_FRIM_138
SH018438.06FU_AJ698114_reps_singleton	1	2	0	0	0	0	0	0	1	0	0	0	0	0	0	0	0	0	4	0	0	0	0	0	1	0	0	2	0	0	Fungi; Basidiomycota; Agaricomycetes; Polyporales; Ganodermataceae; Ganoderma; Ganoderma_sp_FRIM_138
New.ReferenceOTU590	0	0	0	0	0	0	0	0	0	0	0	0	0	0	0	0	0	0	0	0	1	0	0	0	0	0	0	0	0	0	Fungi; Basidiomycota; Agaricomycetes; Polyporales; Ganodermataceae; Ganoderma; Ganoderma_sp_JM97/31
SH116499.06FU_AF255146_reps	3	0	0	0	0	0	0	0	0	0	0	0	0	0	0	0	0	0	0	0	0	0	0	0	0	0	0	0	0	0	Fungi; Basidiomycota; Agaricomycetes; Polyporales; Ganodermataceae; Ganoderma; Ganoderma_sp_JM98/338
New.ReferenceOTU5749	0	0	0	0	0	0	0	0	0	0	0	0	0	0	0	0	0	0	0	0	0	0	0	0	0	0	0	0	1	0	Fungi; Basidiomycota; Agaricomycetes; Polyporales; Ganodermataceae; Ganoderma; Ganoderma_sp_SUT_H3
SH018435.06FU_AY569452_reps_singleton	0	0	1	0	0	0	0	0	0	0	0	0	0	0	0	0	0	0	0	0	0	0	0	0	0	0	0	0	0	0	Fungi; Basidiomycota; Agaricomycetes; Polyporales; Ganodermataceae; Ganoderma; Ganoderma_sp_SUT_H3
New.ReferenceOTU406	0	0	0	0	0	0	0	0	0	0	0	0	0	0	3	0	0	0	0	0	0	0	0	0	0	0	0	0	0	0	Fungi; Basidiomycota; Microbotryomycetes; Sporidiobolales; Incertae_sedis; Rhodotorula; Rhodotorula_lamellibrachiae
New.ReferenceOTU7200	0	0	0	0	0	0	0	0	0	0	0	0	0	0	0	0	0	0	0	0	0	0	0	0	1	0	0	0	0	0	Fungi; Basidiomycota; Microbotryomycetes; Sporidiobolales; Incertae_sedis; Rhodotorula; Rhodotorula_lamellibrachiae
New.ReferenceOTU76	0	0	0	0	0	0	0	0	0	0	0	0	0	0	0	0	0	0	0	1	0	0	0	0	0	0	0	0	2	0	Fungi; Basidiomycota; Microbotryomycetes; Sporidiobolales; Incertae_sedis; Rhodotorula; Rhodotorula_retinophila
New.ReferenceOTU4451	1	0	0	0	0	0	0	0	0	0	0	0	0	0	0	0	0	7	0	0	0	0	0	0	0	0	0	0	0	0	Fungi; Basidiomycota; Microbotryomycetes; Sporidiobolales; Incertae_sedis; Rhodotorula; Rhodotorula_sp
New.ReferenceOTU1407	0	0	0	0	0	0	0	0	0	0	0	0	0	0	0	0	0	0	9	0	0	0	0	0	0	0	0	0	0	0	Fungi; Basidiomycota; Microbotryomycetes; Sporidiobolales; Incertae_sedis; Rhodotorula; Rhodotorula_sp_CBS_8594
New.ReferenceOTU5985	0	0	0	0	0	0	0	0	0	0	0	0	0	0	0	0	0	0	0	1	0	0	0	0	0	0	0	0	1	0	Fungi; Basidiomycota; Microbotryomycetes; Sporidiobolales; Incertae_sedis; Rhodotorula; Rhodotorula_sp_CECT_11976
SH135024.06FU_AF145321_refs	0	0	0	0	0	0	0	0	0	0	0	0	0	0	0	0	0	0	0	0	0	0	0	0	1	0	0	0	0	0	Fungi; Basidiomycota; Tremellomycetes; Filobasidiales; Filobasidiaceae; Cryptococcus; Cryptococcus_albidus
SH125658.06FU_JN572151_reps	0	0	0	0	0	0	0	0	0	0	0	0	0	0	0	0	1	0	0	0	0	0	0	0	0	0	0	0	0	0	Fungi; Basidiomycota; Tremellomycetes; Filobasidiales; Filobasidiaceae; Cryptococcus; Cryptococcus_flavus
New.ReferenceOTU7320	0	4	0	0	0	0	0	0	0	0	0	0	0	0	0	0	0	0	0	0	0	0	0	0	0	0	0	0	0	0	Fungi; Basidiomycota; Tremellomycetes; Tremellales; Incertae_sedis; Cryptococcus; Cryptococcus_cf_cellulolyticus_ATT064
New.ReferenceOTU3372	0	0	0	0	1	0	0	0	0	0	0	0	0	0	0	0	0	0	0	0	0	0	0	0	0	0	0	0	0	0	Fungi; Basidiomycota; Tremellomycetes; Tremellales; Incertae_sedis; Cryptococcus; Cryptococcus_laurentii
SH091973.06FU_JN675354_reps	0	0	0	0	0	1	0	0	0	0	0	0	0	0	0	0	0	0	0	0	0	0	0	0	0	0	0	0	0	0	Fungi; Basidiomycota; Tremellomycetes; Tremellales; Incertae_sedis; Cryptococcus; Cryptococcus_laurentii
New.ReferenceOTU2202	0	0	0	0	0	0	0	1	0	0	0	0	0	0	0	0	0	1	0	1	0	0	0	0	0	0	0	0	0	0	Fungi; Basidiomycota; Tremellomycetes; Tremellales; Incertae_sedis; Cryptococcus; Cryptococcus_podzolicus
New.ReferenceOTU5213	0	0	0	0	1	0	1	0	0	0	1	0	3	0	0	0	0	0	3	5	0	0	0	0	0	0	0	0	0	0	Fungi; Basidiomycota; Tremellomycetes; Tremellales; Incertae_sedis; Cryptococcus; Cryptococcus_podzolicus
New.ReferenceOTU6043	0	0	0	0	0	0	0	0	0	0	0	0	1	0	0	0	0	0	0	0	0	0	0	0	0	0	0	0	0	0	Fungi; Basidiomycota; Tremellomycetes; Tremellales; Incertae_sedis; Cryptococcus; Cryptococcus_podzolicus
New.ReferenceOTU7482	0	0	0	0	0	0	2	0	0	0	0	0	2	0	0	0	0	0	0	1	0	0	0	0	0	0	0	0	0	0	Fungi; Basidiomycota; Tremellomycetes; Tremellales; Incertae_sedis; Cryptococcus; Cryptococcus_podzolicus
New.ReferenceOTU8093	6	3	5	5	4	2	6	1	3	2	6	6	12	0	1	0	1	5	12	4	0	0	0	3	0	0	0	0	2	4	Fungi; Basidiomycota; Tremellomycetes; Tremellales; Incertae_sedis; Cryptococcus; Cryptococcus_podzolicus
New.ReferenceOTU982	0	0	0	0	0	0	0	0	0	1	0	0	0	0	0	0	0	0	0	0	0	0	0	0	0	0	0	0	0	0	Fungi; Basidiomycota; Tremellomycetes; Tremellales; Incertae_sedis; Cryptococcus; Cryptococcus_podzolicus
SH094001.06FU_AF444321_refs	2	15	28	23	16	17	14	7	12	1	16	38	37	10	19	0	6	29	23	49	6	2	8	9	4	4	0	0	25	9	Fungi; Basidiomycota; Tremellomycetes; Tremellales; Incertae_sedis; Cryptococcus; Cryptococcus_podzolicus
New.ReferenceOTU4961	0	0	1	0	0	0	0	0	0	0	0	0	0	2	0	0	0	0	0	1	0	0	0	0	0	0	0	0	0	0	Fungi; Basidiomycota; Tremellomycetes; Tremellales; Incertae_sedis; Cryptococcus; Cryptococcus_sp
New.ReferenceOTU3135	5	3	0	2	4	1	0	0	6	6	5	1	13	4	0	0	3	0	0	0	0	0	0	7	0	0	0	2	1	0	Fungi; Basidiomycota; Tremellomycetes; Tremellales; Incertae_sedis; Cryptococcus; Cryptococcus_sp_ATCC_MYA_4669
New.ReferenceOTU3644	0	0	0	0	0	0	0	0	0	0	0	0	0	0	1	0	0	0	0	0	0	0	0	0	0	0	0	0	0	0	Fungi; Basidiomycota; Tremellomycetes; Tremellales; Incertae_sedis; Cryptococcus; Cryptococcus_sp_ATCC_MYA_4669
New.ReferenceOTU7668	0	0	0	0	0	0	0	0	0	0	0	0	0	0	0	0	0	0	0	0	0	0	0	1	0	0	0	0	0	0	Fungi; Basidiomycota; Tremellomycetes; Tremellales; Incertae_sedis; Cryptococcus; Cryptococcus_sp_AY_59
New.ReferenceOTU6431	0	0	0	0	0	0	0	0	0	0	0	0	0	0	0	0	0	0	0	0	0	0	0	0	0	0	0	1	0	0	Fungi; Basidiomycota; Tremellomycetes; Tremellales; Incertae_sedis; Cryptococcus; Cryptococcus_sp_BC_2011
New.ReferenceOTU1334	0	0	0	0	0	0	0	0	0	0	0	0	0	0	0	0	0	0	1	1	0	0	0	0	0	0	0	0	0	0	Fungi; Basidiomycota; Tremellomycetes; Tremellales; Incertae_sedis; Cryptococcus; Cryptococcus_sp_CBS_7890
New.ReferenceOTU5857	0	0	0	0	0	0	0	0	0	0	0	0	0	0	0	0	0	0	0	1	0	0	0	0	0	0	0	0	0	0	Fungi; Basidiomycota; Tremellomycetes; Tremellales; Incertae_sedis; Cryptococcus; Cryptococcus_sp_CBS_7890
New.ReferenceOTU2857	0	0	0	0	0	0	0	0	0	0	0	0	0	0	0	0	0	3	0	0	0	0	0	0	0	0	0	0	0	0	Fungi; Basidiomycota; Tremellomycetes; Tremellales; Incertae_sedis; Cryptococcus; Cryptococcus_sp_CBS_8358
SH121014.06FU_AF444387_reps	0	0	0	0	0	0	0	0	0	0	0	0	0	1	0	0	0	0	0	0	0	0	0	0	0	0	0	0	0	0	Fungi; Basidiomycota; Tremellomycetes; Tremellales; Incertae_sedis; Cryptococcus; Cryptococcus_sp_CBS_8358
New.CleanUp.ReferenceOTU1838	0	0	0	0	0	0	0	0	0	0	0	1	0	0	0	0	0	0	0	0	0	0	0	0	0	0	0	0	0	0	Fungi; Basidiomycota; Tremellomycetes; Tremellales; Incertae_sedis; Cryptococcus; Cryptococcus_sp_LH42
New.ReferenceOTU186	0	1	0	0	0	0	0	0	0	0	0	0	0	0	0	0	0	0	0	0	0	0	0	0	0	0	0	0	0	0	Fungi; Basidiomycota; Tremellomycetes; Tremellales; Incertae_sedis; Cryptococcus; Cryptococcus_sp_LH42
New.ReferenceOTU5029	0	0	0	0	0	0	0	0	0	0	0	0	0	0	0	0	0	5	0	0	0	0	0	0	0	0	0	0	0	0	Fungi; Basidiomycota; Tremellomycetes; Tremellales; Incertae_sedis; Cryptococcus; Cryptococcus_sp_LH42
New.ReferenceOTU6470	0	0	0	0	0	0	0	1	0	0	0	0	0	0	0	0	0	0	0	0	0	0	0	0	0	0	0	0	0	0	Fungi; Basidiomycota; Tremellomycetes; Tremellales; Incertae_sedis; Cryptococcus; Cryptococcus_sp_SJ4L02
SH094203.06FU_FJ153174_reps	0	0	0	0	0	0	0	4	0	0	0	0	0	2	0	0	1	0	1	0	0	0	0	0	0	0	0	0	0	1	Fungi; Basidiomycota; Tremellomycetes; Tremellales; Incertae_sedis; Cryptococcus; Cryptococcus_sp_SJ4L02
New.ReferenceOTU6189	0	0	0	0	0	0	0	0	0	0	0	0	0	0	0	0	0	0	0	0	0	0	0	0	0	0	0	0	0	1	Fungi; Basidiomycota; Tremellomycetes; Tremellales; Incertae_sedis; Cryptococcus; Cryptococcus_sp_UFMG_BRO443
New.ReferenceOTU7323	5	0	0	0	0	0	0	0	0	0	0	0	0	0	0	0	0	0	0	0	0	0	0	0	0	0	0	0	0	0	Fungi; Basidiomycota; Tremellomycetes; Tremellales; Incertae_sedis; Cryptococcus; Cryptococcus_sp_UFMG_BRO443
New.ReferenceOTU7884	0	0	0	0	0	0	0	0	0	0	0	0	0	0	0	0	0	0	0	0	0	0	1	0	0	0	0	0	0	0	Fungi; Basidiomycota; Tremellomycetes; Tremellales; Incertae_sedis; Cryptococcus; Cryptococcus_sp_UFMG_BRO443
New.ReferenceOTU8038	0	0	0	0	0	0	0	0	0	0	1	0	0	0	0	0	0	0	0	0	0	0	0	0	0	0	0	0	0	0	Fungi; Basidiomycota; Tremellomycetes; Tremellales; Incertae_sedis; Cryptococcus; Cryptococcus_sp_UFMG_BRO443
New.ReferenceOTU2579	0	0	0	0	0	0	0	0	0	0	0	0	0	0	0	0	0	0	0	0	0	3	0	0	0	0	0	0	0	0	Fungi; Chytridiomycota; Chytridiomycetes; Spizellomycetales; Spizellomycetaceae; Spizellomyces; Spizellomyces_sp
New.ReferenceOTU3390	0	0	0	0	0	0	0	0	0	0	0	0	0	0	1	0	0	0	0	0	0	0	0	0	0	0	0	0	0	0	Fungi; Chytridiomycota; Chytridiomycetes; Spizellomycetales; Spizellomycetaceae; Spizellomyces; Spizellomyces_sp
New.ReferenceOTU3829	0	0	0	0	0	0	0	0	0	0	0	0	0	0	0	0	0	0	0	0	0	1	0	0	1	0	0	0	0	0	Fungi; Chytridiomycota; Chytridiomycetes; Spizellomycetales; Spizellomycetaceae; Spizellomyces; Spizellomyces_sp
New.ReferenceOTU1577	0	2	0	0	0	0	0	0	0	0	0	0	0	0	0	0	0	0	0	0	0	0	0	0	0	0	0	0	0	0	Fungi; Ascomycota; Dothideomycetes; Botryosphaeriales; Botryosphaeriaceae; Botryosphaeria; Botryosphaeria_sp
New.ReferenceOTU3189	0	0	0	0	0	0	0	0	0	0	0	1	0	0	0	0	0	0	0	0	0	0	0	0	0	0	0	0	0	0	Fungi; Ascomycota; Dothideomycetes; Botryosphaeriales; Botryosphaeriaceae; Botryosphaeria; Botryosphaeria_sp
New.ReferenceOTU2052	0	0	0	0	1	0	0	0	0	0	0	0	0	0	0	0	0	0	0	0	0	0	0	1	0	0	0	0	0	0	Fungi; Ascomycota; Dothideomycetes; Botryosphaeriales; Botryosphaeriaceae; Endomelanconiopsis; Endomelanconiopsis_endophytica
New.ReferenceOTU5957	0	0	0	0	0	0	0	0	0	0	1	0	0	0	0	0	0	0	0	0	0	0	0	0	0	0	0	0	0	0	Fungi; Ascomycota; Dothideomycetes; Botryosphaeriales; Botryosphaeriaceae; Endomelanconiopsis; Endomelanconiopsis_endophytica
SH140840.06FU_EU683656_refs	0	0	0	0	1	0	0	0	4	0	4	0	0	0	0	0	1	0	0	0	0	0	0	0	0	0	0	0	1	0	Fungi; Ascomycota; Dothideomycetes; Botryosphaeriales; Botryosphaeriaceae; Endomelanconiopsis; Endomelanconiopsis_endophytica
New.ReferenceOTU6996	0	0	0	0	0	0	0	0	0	2	0	0	0	0	0	0	0	0	0	0	0	0	0	0	0	0	0	0	0	0	Fungi; Ascomycota; Dothideomycetes; Botryosphaeriales; Botryosphaeriaceae; Endomelanconiopsis; Endomelanconiopsis_microspora
New.ReferenceOTU7493	0	0	0	0	0	0	0	0	0	0	1	0	0	1	0	0	3	0	0	0	0	0	0	1	0	0	0	0	0	0	Fungi; Ascomycota; Dothideomycetes; Botryosphaeriales; Botryosphaeriaceae; Endomelanconiopsis; Endomelanconiopsis_microspora
SH140861.06FU_EU683655_refs	0	0	0	0	2	0	0	0	0	0	0	0	0	0	0	0	0	0	0	0	0	0	0	0	0	0	0	0	0	0	Fungi; Ascomycota; Dothideomycetes; Botryosphaeriales; Botryosphaeriaceae; Endomelanconiopsis; Endomelanconiopsis_microspora
SH106573.06FU_EU144050_refs	0	0	0	0	0	0	0	1	0	3	1	0	0	0	0	2	2	0	0	0	0	0	1	0	1	0	0	0	0	0	Fungi; Ascomycota; Dothideomycetes; Botryosphaeriales; Botryosphaeriaceae; Lasiodiplodia; Lasiodiplodia_margaritacea
New.ReferenceOTU6142	0	0	1	0	0	0	0	0	0	0	0	0	0	0	0	0	0	0	0	0	0	0	0	0	0	0	0	0	0	0	Fungi; Ascomycota; Dothideomycetes; Botryosphaeriales; Botryosphaeriaceae; Lasiodiplodia; Lasiodiplodia_rubropurpurea
SH106570.06FU_DQ458892_refs	0	0	4	0	0	0	0	0	0	0	0	0	0	0	0	0	0	0	0	0	0	0	0	0	0	0	0	0	0	0	Fungi; Ascomycota; Dothideomycetes; Botryosphaeriales; Botryosphaeriaceae; Lasiodiplodia; Lasiodiplodia_rubropurpurea
SH106571.06FU_DQ103554_reps	0	0	10	0	0	1	0	0	0	1	0	1	1	1	0	0	1	0	0	0	0	0	0	1	1	0	0	0	0	1	Fungi; Ascomycota; Dothideomycetes; Botryosphaeriales; Botryosphaeriaceae; Lasiodiplodia; Lasiodiplodia_rubropurpurea
New.ReferenceOTU153	0	0	1	1	0	0	2	0	0	0	0	2	3	0	1	2	0	0	0	0	0	1	0	0	0	0	0	0	0	1	Fungi; Ascomycota; Dothideomycetes; Botryosphaeriales; Botryosphaeriaceae; Lasiodiplodia; Lasiodiplodia_theobromae
SH012638.06FU_FJ545345_reps_singleton	0	0	0	0	0	0	0	1	0	0	0	0	0	0	0	0	0	0	0	0	0	0	0	0	0	0	0	0	0	0	Fungi; Ascomycota; Dothideomycetes; Botryosphaeriales; Botryosphaeriaceae; Lasiodiplodia; Lasiodiplodia_theobromae
SH012646.06FU_JX868750_reps_singleton	0	0	2	1	0	0	0	0	0	3	0	0	1	0	0	0	0	0	0	0	0	0	0	0	0	0	0	0	0	0	Fungi; Ascomycota; Dothideomycetes; Botryosphaeriales; Botryosphaeriaceae; Lasiodiplodia; Lasiodiplodia_theobromae
SH106568.06FU_HQ288227_refs	2	8	7	35	2	4	7	13	0	4	10	11	5	22	7	17	12	0	0	0	0	1	14	4	7	0	1	1	0	13	Fungi; Ascomycota; Dothideomycetes; Botryosphaeriales; Botryosphaeriaceae; Lasiodiplodia; Lasiodiplodia_theobromae
SH140860.06FU_GU199370_reps	0	0	0	1	0	0	0	0	0	0	0	0	1	0	0	0	0	0	0	0	0	0	0	0	0	0	2	0	0	0	Fungi; Ascomycota; Dothideomycetes; Botryosphaeriales; Botryosphaeriaceae; Lasiodiplodia; Lasiodiplodia_theobromae
New.ReferenceOTU7183	0	0	1	0	0	0	0	0	0	0	0	0	0	0	0	0	0	0	0	0	0	0	0	0	0	0	0	0	0	0	Fungi; Ascomycota; Dothideomycetes; Botryosphaeriales; Botryosphaeriaceae; Melanops; Melanops_sp_CBS11839
New.ReferenceOTU364	0	0	0	0	0	0	0	0	0	0	0	0	0	0	0	0	0	0	0	0	0	0	0	0	0	0	0	0	0	3	Fungi; Ascomycota; Dothideomycetes; Botryosphaeriales; Botryosphaeriaceae; Microdiplodia; Microdiplodia_sp
New.ReferenceOTU7711	0	0	0	0	0	0	0	0	0	1	0	0	0	0	0	0	0	0	0	0	0	0	0	0	0	0	0	0	0	0	Fungi; Ascomycota; Dothideomycetes; Botryosphaeriales; Botryosphaeriaceae; Microdiplodia; Microdiplodia_sp
SH126635.06FU_EU686121_reps	0	0	0	0	1	0	0	0	0	0	0	0	1	0	0	0	0	0	0	0	0	0	0	0	0	0	0	0	0	2	Fungi; Ascomycota; Dothideomycetes; Botryosphaeriales; Botryosphaeriaceae; Microdiplodia; Microdiplodia_sp
SH143936.06FU_EU272515_reps	0	0	0	0	0	0	0	0	0	0	2	0	0	0	0	0	2	0	0	0	0	0	0	0	0	0	0	0	0	0	Fungi; Ascomycota; Dothideomycetes; Botryosphaeriales; Botryosphaeriaceae; Microdiplodia; Microdiplodia_sp
New.ReferenceOTU449	0	0	0	0	0	1	0	0	0	0	0	0	0	0	0	0	0	0	0	0	0	0	0	0	0	0	0	0	0	0	Fungi; Ascomycota; Dothideomycetes; Capnodiales; Davidiellaceae; Cladosporium; Cladosporium_sp_1_GP_2013
New.ReferenceOTU3162	0	0	0	0	0	0	0	0	0	0	0	0	1	0	0	0	1	0	0	0	0	0	0	0	1	0	0	0	0	0	Fungi; Ascomycota; Dothideomycetes; Capnodiales; Davidiellaceae; Cladosporium; Cladosporium_sp_4_MU_2012
New.ReferenceOTU6343	0	0	0	0	0	1	0	0	0	0	0	0	2	0	0	0	0	0	0	0	0	0	0	0	0	0	0	0	0	0	Fungi; Ascomycota; Dothideomycetes; Capnodiales; Davidiellaceae; Cladosporium; Cladosporium_sp_4_MU_2012
SH093668.06FU_EF679363_refs	0	0	0	0	0	0	1	2	0	0	0	0	5	1	0	0	0	0	0	0	0	0	0	0	0	0	0	0	5	0	Fungi; Ascomycota; Dothideomycetes; Capnodiales; Davidiellaceae; Cladosporium; Cladosporium_sp_4_MU_2012
SH093680.06FU_FN435677_reps	0	0	4	2	1	3	4	5	0	0	1	2	32	3	4	1	19	0	0	0	0	2	0	3	4	0	1	0	0	0	Fungi; Ascomycota; Dothideomycetes; Capnodiales; Davidiellaceae; Cladosporium; Cladosporium_sp_4_MU_2012
SH093669.06FU_DQ780364_refs	0	0	0	0	0	0	0	0	0	0	0	0	0	0	0	0	0	0	0	0	0	4	0	0	0	0	0	0	0	0	Fungi; Ascomycota; Dothideomycetes; Capnodiales; Davidiellaceae; Cladosporium; Cladosporium_sphaerospermum
SH093672.06FU_FR774127_reps	0	0	0	0	0	0	0	0	0	0	0	0	1	0	0	0	0	0	0	0	0	0	0	0	0	0	0	0	0	0	Fungi; Ascomycota; Dothideomycetes; Capnodiales; Davidiellaceae; Cladosporium; Cladosporium_sphaerospermum
New.CleanUp.ReferenceOTU942	0	0	1	0	0	0	0	0	0	0	0	0	0	0	0	0	0	0	0	0	0	0	0	0	0	0	0	0	0	0	Fungi; Ascomycota; Dothideomycetes; Capnodiales; Davidiellaceae; Cladosporium; Cladosporium_tenuissimum
New.ReferenceOTU1154	0	0	1	0	0	0	0	0	0	0	0	0	1	0	0	0	2	0	0	0	0	0	0	0	0	0	0	0	0	0	Fungi; Ascomycota; Dothideomycetes; Capnodiales; Davidiellaceae; Cladosporium; Cladosporium_tenuissimum
New.ReferenceOTU5418	0	0	3	0	0	0	0	0	0	0	0	2	1	0	0	0	1	0	0	0	0	0	0	0	0	0	0	0	0	0	Fungi; Ascomycota; Dothideomycetes; Capnodiales; Davidiellaceae; Cladosporium; Cladosporium_tenuissimum
SH005108.06FU_KF212243_reps_singleton	0	0	0	0	0	0	0	0	0	0	0	0	0	0	0	0	1	0	0	0	0	0	0	0	0	0	0	0	0	0	Fungi; Ascomycota; Dothideomycetes; Capnodiales; Davidiellaceae; Cladosporium; Cladosporium_tenuissimum
SH005120.06FU_AF502854_reps_singleton	0	0	39	2	0	7	4	11	0	1	3	7	51	102	2	9	47	0	0	0	2	11	2	2	2	0	4	0	0	2	Fungi; Ascomycota; Dothideomycetes; Capnodiales; Davidiellaceae; Cladosporium; Cladosporium_tenuissimum

SH005141.06FU_FR774125_reps_singleton	0	0	0	0	0	0	1	0	0	0	1	0	0	0	0	1	0	0	0	0	0	0	0	0	0	0	0	0	0	0	Fungi; Ascomycota; Dothideomycetes; Capnodiales; Davidiellaceae; Cladosporium; Cladosporium_tenuissimum
SH093683.06FU_GU721677_reps	0	0	4	0	0	0	1	0	0	1	0	1	11	10	0	3	7	0	0	0	0	1	0	0	0	0	0	0	0	0	Fungi; Ascomycota; Dothideomycetes; Capnodiales; Davidiellaceae; Cladosporium; Cladosporium_tenuissimum
New.ReferenceOTU2312	0	0	0	0	0	0	0	0	0	0	0	0	0	0	0	0	0	0	4	0	0	0	0	0	0	0	0	0	0	0	Fungi; Ascomycota; Dothideomycetes; Capnodiales; Mycosphaerellaceae; Pseudocercospora; Pseudocercospora_sphaerellae_eugeniae
SH112887.06FU_JQ324983_refs	0	0	0	0	0	0	0	0	0	0	0	0	0	0	0	0	0	0	0	1	0	0	0	0	0	0	0	0	0	0	Fungi; Ascomycota; Dothideomycetes; Capnodiales; Mycosphaerellaceae; Pseudocercospora; Pseudocercospora_xanthocercidis
New.ReferenceOTU2779	0	0	0	0	0	0	0	0	0	0	0	0	0	0	0	0	0	0	0	0	0	0	0	0	0	0	0	0	0	1	Fungi; Ascomycota; Dothideomycetes; Capnodiales; Mycosphaerellaceae; Ramularia; Ramularia_rumicis_crispi
New.ReferenceOTU1614	0	0	0	0	0	0	0	4	0	0	0	0	0	0	0	0	0	0	0	0	0	0	0	0	0	0	0	0	0	0	Fungi; Ascomycota; Dothideomycetes; Capnodiales; Teratosphaeriaceae; Catenulostroma; Catenulostroma_corymbiae
New.ReferenceOTU310	0	0	0	0	0	0	0	0	0	0	0	0	0	0	0	0	0	0	0	0	0	0	0	0	0	0	0	0	0	2	Fungi; Ascomycota; Dothideomycetes; Capnodiales; Teratosphaeriaceae; Catenulostroma; Catenulostroma_corymbiae
SH036429.06FU_KC005783_refs_singleton	0	0	0	0	0	0	0	0	0	0	0	0	0	0	0	0	0	0	0	0	0	0	0	0	0	0	0	0	0	4	Fungi; Ascomycota; Dothideomycetes; Capnodiales; Teratosphaeriaceae; Catenulostroma; Catenulostroma_corymbiae
New.ReferenceOTU134	0	0	0	0	0	0	0	0	0	0	0	0	0	0	3	0	0	0	0	0	0	2	0	0	0	0	0	0	0	0	Fungi; Ascomycota; Dothideomycetes; Capnodiales; Teratosphaeriaceae; Devriesia; Devriesia_sp
New.ReferenceOTU2415	0	0	0	0	0	0	0	0	0	0	0	0	0	0	0	0	0	0	0	0	1	0	0	0	0	0	0	0	0	0	Fungi; Ascomycota; Dothideomycetes; Capnodiales; Teratosphaeriaceae; Devriesia; Devriesia_sp
New.ReferenceOTU5626	0	0	0	0	0	0	0	0	0	0	0	0	0	0	1	0	0	0	0	0	0	0	0	0	0	0	0	0	0	0	Fungi; Ascomycota; Dothideomycetes; Capnodiales; Teratosphaeriaceae; Devriesia; Devriesia_sp
SH033995.06FU_JQ905788_reps_singleton	0	0	0	0	0	0	0	0	0	0	0	0	0	0	1	0	0	0	0	0	0	1	0	0	0	0	0	1	0	0	Fungi; Ascomycota; Dothideomycetes; Capnodiales; Teratosphaeriaceae; Devriesia; Devriesia_sp
New.ReferenceOTU6587	0	0	1	0	0	0	0	0	0	0	0	0	0	0	0	0	0	0	0	0	0	0	0	0	0	0	0	0	0	0	Fungi; Ascomycota; Dothideomycetes; Capnodiales; Teratosphaeriaceae; Devriesia; Devriesia_staurophora
New.ReferenceOTU3007	0	0	0	0	0	0	0	0	1	0	0	0	0	0	0	0	0	2	1	0	0	0	1	0	0	0	0	0	0	0	Fungi; Ascomycota; Dothideomycetes; Capnodiales; Teratosphaeriaceae; Readeriella; Readeriella_sp
New.ReferenceOTU4351	0	0	0	0	0	0	0	0	0	0	0	0	1	0	0	0	0	0	1	0	0	0	0	0	0	0	0	0	0	0	Fungi; Ascomycota; Dothideomycetes; Capnodiales; Teratosphaeriaceae; Readeriella; Readeriella_sp
New.ReferenceOTU4490	0	0	2	0	0	0	0	0	1	0	0	0	0	0	0	0	0	0	0	0	0	0	0	0	0	0	0	0	0	0	Fungi; Ascomycota; Dothideomycetes; Capnodiales; Teratosphaeriaceae; Readeriella; Readeriella_sp
SH119329.06FU_JX317505_reps	1	0	7	3	0	3	0	0	16	3	3	0	0	0	0	0	0	1	7	2	1	0	10	4	0	0	1	0	0	0	Fungi; Ascomycota; Dothideomycetes; Capnodiales; Teratosphaeriaceae; Readeriella; Readeriella_sp
SH119330.06FU_JX317504_reps	0	0	8	0	0	0	0	0	4	0	0	0	0	0	0	0	0	0	0	2	0	0	0	0	0	0	0	0	0	0	Fungi; Ascomycota; Dothideomycetes; Capnodiales; Teratosphaeriaceae; Readeriella; Readeriella_sp
New.ReferenceOTU3844	0	0	0	0	0	0	0	0	0	0	0	0	0	0	0	0	0	0	0	0	0	6	0	0	0	0	0	0	0	0	Fungi; Ascomycota; Dothideomycetes; Capnodiales; Teratosphaeriaceae; Teratosphaeria; Teratosphaeria_jonkershoekensis
New.ReferenceOTU3369	0	0	0	0	0	0	0	0	0	0	0	0	0	0	0	0	0	0	0	0	0	0	0	0	0	0	1	0	0	0	Fungi; Ascomycota; Dothideomycetes; Incertae_sedis; Incertae_sedis; Asteromella; Asteromella_sp
New.ReferenceOTU542	0	0	0	0	0	0	0	1	0	0	0	2	0	1	2	0	0	2	0	1	0	0	0	0	0	0	0	0	0	0	Fungi; Ascomycota; Dothideomycetes; Incertae_sedis; Parodiopsidaceae; Perisporiopsis; Perisporiopsis_sp
New.ReferenceOTU2203	0	0	0	0	0	0	0	0	0	0	0	0	0	0	2	0	0	0	0	0	0	0	10	0	0	0	2	0	0	25	Fungi; Ascomycota; Dothideomycetes; Pleosporales; Incertae_sedis; Stagonosporopsis; Stagonosporopsis_ajacis
New.ReferenceOTU2995	0	0	0	0	0	0	0	0	0	0	0	0	0	0	0	0	0	0	0	0	0	0	0	0	0	0	0	2	0	0	Fungi; Ascomycota; Dothideomycetes; Pleosporales; Incertae_sedis; Stagonosporopsis; Stagonosporopsis_ajacis
SH100170.06FU_FJ037752_reps	0	0	0	0	0	0	0	0	0	0	0	0	0	0	0	0	0	0	0	0	0	0	1	0	0	0	0	0	0	1	Fungi; Ascomycota; Dothideomycetes; Pleosporales; Incertae_sedis; Stagonosporopsis; Stagonosporopsis_lupini
SH093006.06FU_AB693792_reps	0	0	0	0	0	0	0	0	0	0	0	0	0	0	0	0	0	0	3	0	0	0	0	0	0	0	0	0	0	0	Fungi; Ascomycota; Dothideomycetes; Pleosporales; Leptosphaeriaceae; Leptosphaeria; Leptosphaeria_sp_B86_8
New.ReferenceOTU3082	0	0	0	0	0	0	0	3	0	0	0	0	0	0	0	0	0	0	0	0	0	0	1	0	0	0	0	0	0	1	Fungi; Ascomycota; Dothideomycetes; Pleosporales; Massarinaceae; Helminthosporium; Helminthosporium_velutinum
New.ReferenceOTU1919	0	0	0	0	0	0	1	0	0	0	0	0	0	0	0	0	0	0	0	0	0	0	0	0	0	0	0	0	0	0	Fungi; Ascomycota; Dothideomycetes; Pleosporales; Pleosporaceae; Bipolaris; Bipolaris_sp_L14
New.ReferenceOTU4852	0	0	0	0	0	0	0	0	0	0	0	0	0	0	0	0	0	0	3	1	0	0	0	0	0	0	0	0	0	0	Fungi; Ascomycota; Dothideomycetes; Pleosporales; Pleosporaceae; Cochliobolus; Cochliobolus_heliconiae
New.ReferenceOTU7739	0	0	0	0	1	0	0	0	0	0	0	0	0	0	0	0	0	0	0	0	0	0	0	0	0	0	0	0	0	0	Fungi; Ascomycota; Dothideomycetes; Pleosporales; Pleosporaceae; Cochliobolus; Cochliobolus_lunatus
SH127391.06FU_JX256429_refs	0	0	0	0	0	0	0	0	0	0	0	0	0	0	0	0	0	0	0	0	0	0	0	0	0	0	0	0	0	2	Fungi; Ascomycota; Dothideomycetes; Pleosporales; Pleosporaceae; Cochliobolus; Cochliobolus_lunatus
SH132854.06FU_KF435172_reps	0	0	0	0	0	0	0	0	0	0	0	0	0	0	3	0	0	0	0	0	0	2	0	0	0	0	0	0	0	0	Fungi; Ascomycota; Dothideomycetes; Pleosporales; Pleosporaceae; Edenia; Edenia_sp
New.ReferenceOTU2670	0	0	0	0	0	0	1	0	0	0	7	0	0	0	0	2	0	0	0	32	0	0	0	0	0	0	0	0	0	0	Fungi; Ascomycota; Dothideomycetes; Pleosporales; Pleosporaceae; Pithomyces; Pithomyces_valparadisiacus
New.ReferenceOTU13	0	0	0	0	0	0	0	0	0	0	0	0	0	0	0	0	0	1	4	4	0	0	0	0	0	0	0	0	0	0	Fungi; Ascomycota; Dothideomycetes; Pleosporales; Pleosporaceae; Stemphylium; Stemphylium_solani
New.ReferenceOTU4005	0	0	5	0	0	0	2	0	0	26	40	4	0	3	0	0	6	0	9	1	1	0	11	0	0	0	22	5	0	1	Fungi; Ascomycota; Dothideomycetes; Pleosporales; Pleosporaceae; Stemphylium; Stemphylium_solani
New.ReferenceOTU1774	0	0	0	0	0	0	0	0	0	0	0	0	0	0	1	0	0	0	0	0	0	0	0	0	0	0	0	0	0	0	Fungi; Ascomycota; Dothideomycetes; Pleosporales; Venturiaceae; Fusicladium; Fusicladium_rhodense
New.ReferenceOTU5288	0	0	0	0	0	0	0	0	0	0	0	0	0	0	0	0	0	0	0	1	0	0	0	0	0	0	0	0	0	0	Fungi; Ascomycota; Eurotiomycetes; Chaetothyriales; Coccodiniaceae; Coccodinium; Coccodinium_bartschii
New.ReferenceOTU821	0	0	0	0	0	0	0	0	0	0	0	0	0	0	0	0	0	0	0	0	0	0	0	0	0	0	0	0	0	1	Fungi; Ascomycota; Eurotiomycetes; Chaetothyriales; Incertae_sedis; Strelitziana; Strelitziana_africana
New.ReferenceOTU5327	0	0	0	0	0	0	0	0	0	0	0	0	0	0	0	0	0	0	0	0	0	0	0	0	0	0	0	0	0	1	Fungi; Ascomycota; Eurotiomycetes; Chaetothyriales; Incertae_sedis; Strelitziana; Strelitziana_albiziae
New.ReferenceOTU3447	0	0	0	0	0	0	0	0	0	0	0	0	0	0	0	0	0	0	0	0	0	0	0	3	0	0	0	0	0	0	Fungi; Ascomycota; Eurotiomycetes; Coryneliales; Coryneliaceae; Caliciopsis; Caliciopsis_indica
New.ReferenceOTU1007	0	0	0	0	0	0	0	0	0	0	0	0	2	0	0	0	0	0	0	0	0	0	0	0	1	0	0	0	0	0	Fungi; Ascomycota; Incertae_sedis; Incertae_sedis; Incertae_sedis; Pyriculariopsis; Pyriculariopsis_sp_OF55a
New.ReferenceOTU5487	0	0	0	0	0	0	0	0	0	0	0	0	0	0	0	0	1	0	0	0	0	0	0	0	0	0	0	0	0	0	Fungi; Ascomycota; Incertae_sedis; Incertae_sedis; Incertae_sedis; Pyriculariopsis; Pyriculariopsis_sp_OF55a
New.ReferenceOTU8067	0	0	1	0	0	0	0	0	0	0	0	0	0	0	0	0	0	0	0	0	0	0	0	0	0	0	0	0	0	0	Fungi; Ascomycota; Incertae_sedis; Incertae_sedis; Incertae_sedis; Pyriculariopsis; Pyriculariopsis_sp_OF55a
SH133871.06FU_EU041816_refs	0	0	0	0	0	0	0	0	0	0	0	0	0	0	1	0	0	0	0	0	0	0	0	0	0	0	0	0	0	0	Fungi; Ascomycota; Incertae_sedis; Incertae_sedis; Incertae_sedis; Veronaea; Veronaea_botryosa
New.ReferenceOTU4333	0	0	0	0	0	0	0	0	0	0	0	0	0	0	0	0	0	0	0	0	0	0	0	0	0	8	0	0	0	0	Fungi; Ascomycota; Leotiomycetes; Erysiphales; Erysiphaceae; Parauncinula; Parauncinula_septata
New.ReferenceOTU5035	0	0	0	0	0	0	0	0	0	0	0	0	0	0	0	0	0	0	0	0	0	0	0	3	0	0	0	0	0	0	Fungi; Ascomycota; Leotiomycetes; Helotiales; Incertae_sedis; Dactylaria; Dactylaria_ampulliformis
New.ReferenceOTU1764	0	0	0	0	0	0	0	0	0	0	0	0	0	0	0	0	0	1	0	0	0	0	0	0	0	0	0	0	3	0	Fungi; Ascomycota; Leotiomycetes; Helotiales; Incertae_sedis; Xylogone; Xylogone_ganodermophthora
New.ReferenceOTU2241	0	2	0	0	0	0	0	0	0	0	0	0	0	0	0	0	0	0	0	0	0	0	0	0	0	0	0	0	0	0	Fungi; Ascomycota; Leotiomycetes; Helotiales; Incertae_sedis; Xylogone; Xylogone_ganodermophthora
New.ReferenceOTU3669	0	0	0	0	0	0	0	0	0	0	0	0	0	0	0	0	1	0	1	0	0	0	0	0	0	0	0	0	0	0	Fungi; Ascomycota; Leotiomycetes; Helotiales; Incertae_sedis; Xylogone; Xylogone_ganodermophthora
New.ReferenceOTU3897	0	0	0	2	0	0	0	0	0	0	2	0	0	0	0	1	1	0	2	0	3	0	0	0	1	0	0	0	0	0	Fungi; Ascomycota; Leotiomycetes; Helotiales; Incertae_sedis; Xylogone; Xylogone_ganodermophthora
New.ReferenceOTU4161	0	0	0	1	0	0	0	0	0	3	0	0	1	0	1	0	0	1	1	0	0	4	1	2	3	0	0	2	4	0	Fungi; Ascomycota; Leotiomycetes; Helotiales; Incertae_sedis; Xylogone; Xylogone_ganodermophthora
New.ReferenceOTU4492	0	1	0	0	0	0	0	0	0	0	0	0	0	0	0	0	1	0	0	1	0	0	0	0	0	0	0	0	0	0	Fungi; Ascomycota; Leotiomycetes; Helotiales; Incertae_sedis; Xylogone; Xylogone_ganodermophthora
New.ReferenceOTU510	0	0	0	0	0	0	3	0	0	0	0	0	0	0	0	0	0	0	0	0	0	0	0	0	0	0	0	0	0	0	Fungi; Ascomycota; Leotiomycetes; Helotiales; Incertae_sedis; Xylogone; Xylogone_ganodermophthora
New.ReferenceOTU7869	0	0	0	0	0	0	0	0	1	0	0	0	0	0	0	0	0	0	0	0	0	0	0	0	0	0	0	0	0	0	Fungi; Ascomycota; Leotiomycetes; Helotiales; Incertae_sedis; Xylogone; Xylogone_ganodermophthora
SH132281.06FU_GQ272612_reps	0	0	0	1	0	0	0	0	0	0	0	0	0	1	0	0	0	0	0	0	0	0	0	0	0	0	0	0	0	0	Fungi; Ascomycota; Leotiomycetes; Helotiales; Incertae_sedis; Xylogone; Xylogone_ganodermophthora
New.ReferenceOTU133	0	0	0	0	0	0	0	0	0	0	0	0	0	0	0	0	2	0	0	0	0	0	0	0	0	0	0	0	0	0	Fungi; Ascomycota; Leotiomycetes; Helotiales; Incertae_sedis; Xylogone; Xylogone_sp
SH132282.06FU_FJ362268_reps	0	0	0	0	0	2	2	0	0	0	1	6	0	0	0	0	0	0	0	0	0	0	0	0	0	0	0	2	0	1	Fungi; Ascomycota; Leotiomycetes; Helotiales; Incertae_sedis; Xylogone; Xylogone_sp
New.ReferenceOTU4335	0	0	0	0	0	0	0	0	0	0	1	0	0	0	0	0	0	0	0	0	0	0	0	0	0	0	0	0	0	0	Fungi; Ascomycota; Leotiomycetes; Helotiales; Incertae_sedis; Xylogone; Xylogone_sphaerospora
New.ReferenceOTU1226	7	0	0	0	0	0	0	0	0	0	0	0	0	0	0	0	0	0	0	0	0	0	0	0	0	0	0	0	0	0	Fungi; Ascomycota; Leotiomycetes; Rhytismatales; Rhytismataceae; Lophodermium; Lophodermium_sp
SH006136.06FU_AY100662_reps_singleton	0	0	0	0	0	0	0	0	0	0	0	0	0	0	0	0	0	0	0	0	0	0	0	0	0	0	0	0	4	0	Fungi; Ascomycota; Leotiomycetes; Rhytismatales; Rhytismataceae; Lophodermium; Lophodermium_sp_0705HL1
SH095477.06FU_AB512299_reps	0	0	0	0	3	0	0	0	0	0	0	0	0	0	0	0	0	0	0	0	0	0	0	0	0	0	0	0	0	0	Fungi; Ascomycota; Leotiomycetes; Rhytismatales; Rhytismataceae; Lophodermium; Lophodermium_sp_0705HL1
New.ReferenceOTU2534	0	0	0	0	0	0	0	0	0	0	0	0	0	0	0	0	0	1	0	0	1	1	0	0	0	0	0	0	0	0	Fungi; Ascomycota; Leotiomycetes; Rhytismatales; Rhytismataceae; Lophodermium; Lophodermium_sp_H25
New.ReferenceOTU3760	0	0	0	0	0	0	0	0	0	0	0	0	1	1	0	0	0	0	0	0	0	0	0	0	0	0	0	0	0	0	Fungi; Ascomycota; Pezizomycetes; Pezizales; Sarcoscyphaceae; Pithya; Pithya_sp
New.ReferenceOTU103	0	0	0	0	0	0	0	0	0	0	0	0	0	0	0	0	0	0	1	0	0	0	0	0	0	0	0	0	0	0	Fungi; Ascomycota; Sordariomycetes; Coniochaetales; Incertae_sedis; Porosphaerella; Porosphaerella_cordanophora
New.ReferenceOTU2391	0	0	0	0	1	0	0	0	0	0	0	0	0	0	0	0	0	0	0	0	0	0	0	0	0	0	0	0	0	0	Fungi; Ascomycota; Sordariomycetes; Coniochaetales; Incertae_sedis; Porosphaerella; Porosphaerella_cordanophora
New.ReferenceOTU6000	1	0	0	1	2	0	0	0	5	0	2	0	0	1	0	0	0	0	0	0	0	0	0	0	0	0	0	0	0	0	Fungi; Ascomycota; Sordariomycetes; Coniochaetales; Incertae_sedis; Porosphaerella; Porosphaerella_cordanophora
SH021253.06FU_FJ903361_reps_singleton	0	0	0	0	0	0	0	0	0	0	0	1	0	0	0	0	0	0	0	0	0	0	0	0	0	0	0	0	0	0	Fungi; Ascomycota; Sordariomycetes; Coniochaetales; Incertae_sedis; Porosphaerella; Porosphaerella_cordanophora
New.ReferenceOTU3181	0	0	13	0	0	0	0	0	0	0	0	0	0	2	0	0	0	0	0	0	0	0	0	0	0	0	0	0	0	0	Fungi; Ascomycota; Sordariomycetes; Diaporthales; Cryphonectriaceae; Amphilogia; Amphilogia_gyrosa
New.ReferenceOTU4178	0	0	0	0	0	0	0	0	0	1	1	0	0	0	0	0	0	0	0	0	0	0	0	0	0	0	0	0	0	0	Fungi; Ascomycota; Sordariomycetes; Diaporthales; Cryphonectriaceae; Amphilogia; Amphilogia_gyrosa
New.ReferenceOTU6332	0	0	0	2	0	0	0	1	0	0	0	1	0	0	0	0	0	0	0	0	0	0	0	0	0	0	0	0	0	12	Fungi; Ascomycota; Sordariomycetes; Diaporthales; Cryphonectriaceae; Amphilogia; Amphilogia_gyrosa
New.ReferenceOTU7707	0	0	0	0	0	0	0	0	0	0	0	0	0	0	0	0	0	0	0	0	1	0	0	0	0	0	0	0	0	0	Fungi; Ascomycota; Sordariomycetes; Diaporthales; Cryphonectriaceae; Prosopidicola; Prosopidicola_mexicana
SH091143.06FU_JX069862_refs	0	0	0	0	0	0	0	0	0	0	0	0	11	0	1	0	0	0	0	0	0	0	0	0	0	0	0	0	0	1	Fungi; Ascomycota; Sordariomycetes; Diaporthales; Diaporthaceae; Diaporthe; Diaporthe_eucalyptorum
New.ReferenceOTU2201	0	0	0	0	0	0	0	0	0	0	1	0	2	0	0	0	0	0	0	0	0	0	0	0	0	0	0	0	0	6	Fungi; Ascomycota; Sordariomycetes; Diaporthales; Diaporthaceae; Diaporthe; Diaporthe_hongkongensis
SH003788.06FU_AB247171_reps_singleton	0	0	0	0	0	0	0	0	0	0	0	0	2	0	0	0	0	0	0	0	0	0	0	0	0	0	0	0	0	4	Fungi; Ascomycota; Sordariomycetes; Diaporthales; Diaporthaceae; Diaporthe; Diaporthe_hongkongensis
SH091164.06FU_KC343119_refs	0	0	0	0	0	0	0	0	0	0	0	0	0	0	0	0	0	0	0	0	0	0	0	0	0	0	0	0	0	3	Fungi; Ascomycota; Sordariomycetes; Diaporthales; Diaporthaceae; Diaporthe; Diaporthe_hongkongensis
New.ReferenceOTU3364	0	0	0	0	0	0	0	0	0	0	0	0	1	0	0	0	0	0	0	0	0	0	0	0	0	0	0	0	0	0	Fungi; Ascomycota; Sordariomycetes; Diaporthales; Diaporthaceae; Diaporthe; Diaporthe_melonis_var._brevistylospora
New.ReferenceOTU2399	0	0	0	0	0	0	0	0	0	0	0	0	0	0	0	0	0	0	2	0	3	0	0	10	0	0	0	1	0	0	Fungi; Ascomycota; Sordariomycetes; Diaporthales; Diaporthaceae; Diaporthe; Diaporthe_sp
New.ReferenceOTU503	0	0	0	0	0	0	0	0	0	0	0	0	0	0	0	0	0	0	0	0	0	0	0	0	0	0	0	0	4	0	Fungi; Ascomycota; Sordariomycetes; Diaporthales; Diaporthaceae; Diaporthe; Diaporthe_sp_1_41
New.ReferenceOTU7779	0	0	0	0	0	0	0	0	0	1	0	0	0	0	0	0	0	0	0	0	0	0	0	0	0	0	0	1	0	0	Fungi; Ascomycota; Sordariomycetes; Diaporthales; Diaporthaceae; Diaporthe; Diaporthe_sp_1_41
New.ReferenceOTU782	0	0	0	0	0	0	0	0	0	0	1	0	0	1	0	0	0	0	0	0	0	0	0	0	0	0	0	0	0	0	Fungi; Ascomycota; Sordariomycetes; Diaporthales; Diaporthaceae; Diaporthe; Diaporthe_sp_1_41
SH003779.06FU_HM992814_reps_singleton	0	0	0	0	0	0	0	0	0	0	0	0	1	0	0	0	0	0	0	0	0	0	0	0	0	0	0	0	0	0	Fungi; Ascomycota; Sordariomycetes; Diaporthales; Diaporthaceae; Diaporthe; Diaporthe_sp_NY6961a
New.ReferenceOTU4334	0	0	0	0	0	0	0	0	0	4	0	0	0	0	0	0	0	0	0	0	0	0	0	0	0	0	0	0	0	0	Fungi; Ascomycota; Sordariomycetes; Diaporthales; Pseudoplagiostomataceae; Pseudoplagiostoma; Pseudoplagiostoma_sp
New.ReferenceOTU4236	0	0	0	0	0	0	0	0	0	0	0	0	0	0	0	0	0	0	0	1	0	0	0	0	0	0	0	0	0	0	Fungi; Ascomycota; Sordariomycetes; Diaporthales; Schizoparmaceae; Coniella; Coniella_sp
New.ReferenceOTU3233	0	0	0	0	0	0	0	0	0	0	6	0	0	0	0	0	0	0	0	0	0	0	0	2	0	0	0	0	0	0	Fungi; Ascomycota; Sordariomycetes; Diaporthales; Schizoparmaceae; Pilidiella; Pilidiella_eucalyptorum
SH110674.06FU_FJ624259_reps	0	0	0	0	1	0	0	0	0	0	0	0	0	0	0	0	0	0	0	0	0	0	0	0	0	0	0	0	0	0	Fungi; Ascomycota; Sordariomycetes; Diaporthales; Schizoparmaceae; Pilidiella; Pilidiella_eucalyptorum
New.ReferenceOTU1298	0	0	20	0	0	0	0	0	0	0	0	0	0	0	0	0	0	0	0	0	0	0	0	0	0	0	0	0	0	0	Fungi; Ascomycota; Sordariomycetes; Diaporthales; Schizoparmaceae; Pilidiella; Pilidiella_quercicola
New.ReferenceOTU5847	0	0	0	0	0	0	0	0	0	1	0	0	0	3	0	1	0	0	0	1	0	0	0	3	0	0	0	0	0	0	Fungi; Ascomycota; Sordariomycetes; Diaporthales; Schizoparmaceae; Pilidiella; Pilidiella_quercicola
New.ReferenceOTU4363	0	0	0	0	0	0	0	0	0	0	2	0	0	0	0	0	0	0	0	0	0	0	0	0	0	0	0	0	0	0	Fungi; Ascomycota; Sordariomycetes; Diaporthales; Schizoparmaceae; Pilidiella; Pilidiella_tibouchinae
New.ReferenceOTU5177	0	0	0	0	0	0	0	0	0	0	0	0	0	0	0	0	0	0	0	0	0	0	1	0	0	0	0	0	0	0	Fungi; Ascomycota; Sordariomycetes; Diaporthales; Togniniaceae; Phaeoacremonium; Phaeoacremonium_inflatipes
New.ReferenceOTU2047	0	0	0	0	0	0	0	0	2	0	0	0	0	0	0	0	0	0	0	0	0	0	0	0	0	0	0	0	0	0	Fungi; Ascomycota; Sordariomycetes; Diaporthales; Togniniaceae; Phaeoacremonium; Phaeoacremonium_sp
New.ReferenceOTU2317	6	0	0	2	0	0	0	0	0	0	2	0	0	0	0	0	0	0	0	0	0	0	0	0	0	0	0	0	0	0	Fungi; Ascomycota; Sordariomycetes; Diaporthales; Togniniaceae; Phaeoacremonium; Phaeoacremonium_sp
New.ReferenceOTU2889	0	0	0	0	0	0	0	0	0	0	0	0	0	0	0	0	0	0	0	0	0	0	0	3	0	0	0	0	0	0	Fungi; Ascomycota; Sordariomycetes; Diaporthales; Togniniaceae; Phaeoacremonium; Phaeoacremonium_sp
New.ReferenceOTU3214	1	0	0	0	0	0	0	0	0	0	0	0	0	0	0	0	0	0	0	0	0	1	0	0	0	0	0	0	0	0	Fungi; Ascomycota; Sordariomycetes; Diaporthales; Togniniaceae; Phaeoacremonium; Phaeoacremonium_sp
New.ReferenceOTU343	0	0	0	0	0	0	0	0	0	0	0	0	0	0	0	0	0	0	0	0	0	0	0	0	0	3	0	0	0	0	Fungi; Ascomycota; Sordariomycetes; Diaporthales; Togniniaceae; Phaeoacremonium; Phaeoacremonium_sp
New.ReferenceOTU3825	0	0	0	0	0	0	0	0	0	0	0	2	0	0	0	0	0	0	0	0	0	0	0	0	0	0	0	0	0	0	Fungi; Ascomycota; Sordariomycetes; Diaporthales; Togniniaceae; Phaeoacremonium; Phaeoacremonium_sp
New.ReferenceOTU4461	0	0	1	0	0	0	0	0	0	0	0	0	0	0	0	0	0	1	1	1	0	0	0	0	0	0	0	0	0	0	Fungi; Ascomycota; Sordariomycetes; Diaporthales; Togniniaceae; Phaeoacremonium; Phaeoacremonium_sp
New.ReferenceOTU4777	0	0	0	0	0	0	0	0	0	0	0	0	0	0	0	0	0	0	0	0	0	0	1	0	0	0	0	0	0	0	Fungi; Ascomycota; Sordariomycetes; Diaporthales; Togniniaceae; Phaeoacremonium; Phaeoacremonium_sp
New.ReferenceOTU5482	0	0	0	0	0	0	0	0	0	0	0	0	0	0	0	0	0	0	0	0	0	0	1	0	0	0	0	0	0	0	Fungi; Ascomycota; Sordariomycetes; Diaporthales; Togniniaceae; Phaeoacremonium; Phaeoacremonium_sp
New.ReferenceOTU5570	5	0	0	1	0	0	0	0	0	0	0	1	0	0	0	0	0	0	0	0	1	0	0	0	0	0	0	0	0	0	Fungi; Ascomycota; Sordariomycetes; Diaporthales; Togniniaceae; Phaeoacremonium; Phaeoacremonium_sp
New.ReferenceOTU6053	0	0	0	0	0	0	0	0	0	0	0	0	0	0	0	0	0	0	3	0	0	0	0	0	0	0	0	0	0	0	Fungi; Ascomycota; Sordariomycetes; Diaporthales; Togniniaceae; Phaeoacremonium; Phaeoacremonium_sp
New.ReferenceOTU6116	0	0	0	0	0	0	0	0	1	0	0	0	0	0	0	0	0	0	0	0	0	0	0	0	0	0	0	0	0	0	Fungi; Ascomycota; Sordariomycetes; Diaporthales; Togniniaceae; Phaeoacremonium; Phaeoacremonium_sp

New.ReferenceOTU6647	0	0	0	0	0	0	0	0	0	0	0	0	0	1	0	1	0	0	0	0	0	0	0	0	0	0	0	0	0	0	Fungi; Ascomycota; Sordariomycetes; Diaporthales; Togniniaceae; Phaeoacremonium; Phaeoacremonium_sp
New.ReferenceOTU2804	0	0	0	0	0	0	0	0	0	0	0	0	0	0	0	0	0	0	3	0	0	0	0	0	0	0	0	0	0	0	Fungi; Ascomycota; Sordariomycetes; Hypocreales; Bionectriaceae; Clonostachys; Clonostachys_chlorina
New.ReferenceOTU1356	0	0	0	0	0	0	0	0	0	0	0	0	0	0	0	0	0	0	0	0	0	0	0	0	0	0	0	0	1	0	Fungi; Ascomycota; Sordariomycetes; Hypocreales; Bionectriaceae; Clonostachys; Clonostachys_phyllophila
New.ReferenceOTU4861	0	0	0	0	0	0	0	0	0	0	0	0	0	0	0	0	1	0	0	0	0	0	0	0	0	0	0	0	0	0	Fungi; Ascomycota; Sordariomycetes; Hypocreales; Bionectriaceae; Clonostachys; Clonostachys_phyllophila
New.ReferenceOTU4324	0	0	0	0	0	0	0	0	2	0	0	0	0	0	0	0	0	0	0	0	0	0	0	0	0	0	0	0	0	0	Fungi; Ascomycota; Sordariomycetes; Hypocreales; Bionectriaceae; Clonostachys; Clonostachys_rosea_f._catenulata
SH034588.06FU_JX155904_reps_singleton	0	0	3	18	2	0	0	0	3	0	1	0	0	0	15	0	0	0	2	0	4	0	0	2	2	1	0	0	5	0	Fungi; Ascomycota; Sordariomycetes; Hypocreales; Bionectriaceae; Clonostachys; Clonostachys_rosea_f._catenulata
SH141467.06FU_AF358233_refs	0	0	0	2	1	0	0	0	0	0	0	1	0	0	0	0	0	0	0	2	0	0	0	1	1	0	0	0	1	0	Fungi; Ascomycota; Sordariomycetes; Hypocreales; Bionectriaceae; Clonostachys; Clonostachys_rosea_f._catenulata
SH141480.06FU_AF358229_reps	0	0	0	0	0	0	0	0	0	0	0	0	0	0	1	0	0	0	0	0	0	0	0	0	0	0	0	0	0	0	Fungi; Ascomycota; Sordariomycetes; Hypocreales; Bionectriaceae; Clonostachys; Clonostachys_rosea_f._catenulata
New.ReferenceOTU621	1	0	0	0	0	0	0	0	1	0	0	0	0	0	0	0	0	0	0	0	0	0	0	0	0	0	0	0	0	0	Fungi; Ascomycota; Sordariomycetes; Hypocreales; Bionectriaceae; Clonostachys; Clonostachys_sp
SH141473.06FU_AY273321_reps	0	5	2	5	0	0	0	0	2	0	1	0	1	0	6	0	0	3	0	8	0	0	0	0	0	0	0	0	0	0	Fungi; Ascomycota; Sordariomycetes; Hypocreales; Bionectriaceae; Clonostachys; Clonostachys_sp
New.ReferenceOTU3665	0	0	0	0	0	0	0	0	0	0	0	0	1	0	0	0	0	0	0	0	0	0	0	0	0	0	0	0	0	0	Fungi; Ascomycota; Sordariomycetes; Hypocreales; Bionectriaceae; Clonostachys; Clonostachys_sp_ATT061
SH025037.06FU_HQ607824_reps_singleton	0	0	0	0	0	0	1	0	0	0	0	0	0	1	0	0	0	0	0	0	0	0	0	0	0	0	0	0	0	0	Fungi; Ascomycota; Sordariomycetes; Hypocreales; Bionectriaceae; Clonostachys; Clonostachys_sp_ATT061
New.ReferenceOTU2969	0	0	0	0	0	0	0	0	0	0	0	0	0	0	0	0	0	6	0	0	0	0	0	0	0	0	0	0	0	0	Fungi; Ascomycota; Sordariomycetes; Hypocreales; Clavicipitaceae; Chamaeleomyces; Chamaeleomyces_granulomatis
New.ReferenceOTU6473	1	0	0	0	0	0	0	0	0	0	0	0	0	0	0	0	0	0	0	0	0	0	0	0	0	0	0	0	0	0	Fungi; Ascomycota; Sordariomycetes; Hypocreales; Clavicipitaceae; Chamaeleomyces; Chamaeleomyces_granulomatis
New.ReferenceOTU235	0	0	0	0	0	0	0	0	0	0	0	0	0	0	1	0	0	0	0	0	0	0	0	0	0	0	0	0	0	0	Fungi; Ascomycota; Sordariomycetes; Hypocreales; Clavicipitaceae; Claviceps; Claviceps_digitariae
New.CleanUp.ReferenceOTU314	0	0	0	0	0	0	0	0	1	0	0	0	0	0	0	0	0	0	0	0	0	0	0	0	0	0	0	0	0	0	Fungi; Ascomycota; Sordariomycetes; Hypocreales; Clavicipitaceae; Claviceps; Claviceps_sp_ICMP_16980
New.ReferenceOTU1248	0	0	0	0	0	0	0	0	0	0	0	0	0	0	0	1	0	0	0	0	0	0	0	0	0	0	0	0	0	0	Fungi; Ascomycota; Sordariomycetes; Hypocreales; Clavicipitaceae; Claviceps; Claviceps_sp_ICMP_16980
New.ReferenceOTU2252	0	0	0	0	0	0	0	0	0	0	0	0	0	0	0	0	0	0	0	0	0	0	0	0	0	0	0	1	0	0	Fungi; Ascomycota; Sordariomycetes; Hypocreales; Clavicipitaceae; Claviceps; Claviceps_sp_ICMP_16980
New.ReferenceOTU4068	0	0	0	0	0	0	0	0	0	0	0	0	0	0	0	1	0	0	0	0	0	3	0	0	0	0	0	0	0	0	Fungi; Ascomycota; Sordariomycetes; Hypocreales; Clavicipitaceae; Claviceps; Claviceps_sp_ICMP_16980
New.ReferenceOTU4166	0	0	0	0	0	0	0	0	0	0	0	0	0	0	0	0	0	0	0	0	0	3	0	0	0	0	0	0	0	0	Fungi; Ascomycota; Sordariomycetes; Hypocreales; Clavicipitaceae; Claviceps; Claviceps_sp_ICMP_16980
New.ReferenceOTU7963	0	0	0	0	0	0	0	0	0	1	0	0	0	0	0	0	0	0	0	0	0	0	0	0	0	0	0	0	0	0	Fungi; Ascomycota; Sordariomycetes; Hypocreales; Clavicipitaceae; Claviceps; Claviceps_sp_SG
New.ReferenceOTU7621	0	0	0	0	0	0	0	0	0	0	0	0	0	0	0	0	0	0	0	0	0	0	1	0	0	0	0	0	0	0	Fungi; Ascomycota; Sordariomycetes; Hypocreales; Clavicipitaceae; Metacordyceps; Metacordyceps_chlamydosporia
SH111198.06FU_KC786989_reps	0	0	0	0	0	0	0	0	0	1	1	0	0	0	0	0	0	0	0	1	0	0	0	0	1	0	0	0	0	0	Fungi; Ascomycota; Sordariomycetes; Hypocreales; Clavicipitaceae; Metacordyceps; Pochonia_chlamydosporia_var_spinulospora
SH111204.06FU_AB709852_reps	0	0	0	0	0	0	0	0	0	0	0	0	0	4	0	1	0	0	0	1	0	0	0	1	0	0	0	0	0	0	Fungi; Ascomycota; Sordariomycetes; Hypocreales; Clavicipitaceae; Metacordyceps; Pochonia_chlamydosporia_var_spinulospora
New.ReferenceOTU617	0	0	0	1	0	0	0	0	0	0	0	0	0	0	0	0	0	2	0	0	0	0	0	0	0	0	0	0	0	0	Fungi; Ascomycota; Sordariomycetes; Hypocreales; Clavicipitaceae; Metarhizium; Metarhizium_album
New.ReferenceOTU959	0	0	0	0	0	0	0	0	0	0	0	0	0	0	0	0	1	0	0	0	0	0	1	0	0	0	0	0	0	0	Fungi; Ascomycota; Sordariomycetes; Hypocreales; Clavicipitaceae; Metarhizium; Metarhizium_album
New.ReferenceOTU2170	0	0	0	0	0	0	0	0	0	0	0	0	0	0	0	0	0	0	2	0	0	0	0	0	0	0	0	0	0	0	Fungi; Ascomycota; Sordariomycetes; Hypocreales; Clavicipitaceae; Metarhizium; Metarhizium_anisopliae
New.ReferenceOTU6425	3	0	0	0	0	0	0	0	0	0	0	1	0	0	2	3	0	0	1	0	1	4	13	1	9	0	1	0	3	2	Fungi; Ascomycota; Sordariomycetes; Hypocreales; Clavicipitaceae; Metarhizium; Metarhizium_anisopliae
SH099648.06FU_HQ331450_refs	3	0	0	0	0	0	1	1	0	2	0	2	0	0	3	3	0	2	3	2	2	0	1	0	21	0	0	0	6	1	Fungi; Ascomycota; Sordariomycetes; Hypocreales; Clavicipitaceae; Metarhizium; Metarhizium_anisopliae
SH099649.06FU_AB524431_reps	0	0	0	0	0	0	0	1	0	0	0	0	0	0	0	0	0	0	0	0	0	0	0	0	2	0	0	0	0	1	Fungi; Ascomycota; Sordariomycetes; Hypocreales; Clavicipitaceae; Metarhizium; Metarhizium_anisopliae
SH099652.06FU_FJ609215_reps	0	0	0	0	0	0	0	0	0	1	0	0	0	0	0	0	0	0	0	0	0	0	0	0	1	0	0	0	0	0	Fungi; Ascomycota; Sordariomycetes; Hypocreales; Clavicipitaceae; Metarhizium; Metarhizium_anisopliae
SH099656.06FU_EF495099_reps	1	0	0	0	0	0	0	0	0	0	0	0	0	1	3	3	1	0	0	3	0	2	11	0	15	0	1	1	4	3	Fungi; Ascomycota; Sordariomycetes; Hypocreales; Clavicipitaceae; Metarhizium; Metarhizium_anisopliae
SH111221.06FU_AY646379_reps	0	0	0	0	0	0	0	0	0	0	0	0	0	1	0	0	0	0	0	0	0	0	0	0	0	0	0	0	0	0	Fungi; Ascomycota; Sordariomycetes; Hypocreales; Clavicipitaceae; Metarhizium; Metarhizium_flavoviride
SH132445.06FU_EF113335_reps	0	0	0	0	0	0	0	0	0	0	0	1	0	0	0	1	0	0	0	0	0	0	0	0	1	0	0	2	0	0	Fungi; Ascomycota; Sordariomycetes; Hypocreales; Clavicipitaceae; Metarhizium; Metarhizium_flavoviride
New.ReferenceOTU660	0	0	0	0	0	0	0	0	0	0	0	0	0	0	0	0	0	0	0	0	0	0	0	0	0	0	0	1	0	0	Fungi; Ascomycota; Sordariomycetes; Hypocreales; Clavicipitaceae; Metarhizium; Metarhizium_flavoviride_var_novazealandicum
New.ReferenceOTU913	0	0	0	0	0	0	0	0	1	0	0	0	0	0	0	0	0	0	0	0	0	0	0	0	0	0	0	0	0	0	Fungi; Ascomycota; Sordariomycetes; Hypocreales; Clavicipitaceae; Metarhizium; Metarhizium_lepidiotae
SH099663.06FU_AY373632_reps	0	0	0	0	0	0	0	0	0	0	0	0	0	0	0	0	0	0	0	0	0	0	0	0	2	0	0	0	0	0	Fungi; Ascomycota; Sordariomycetes; Hypocreales; Clavicipitaceae; Metarhizium; Metarhizium_pinghaense
New.ReferenceOTU4505	0	0	0	0	0	0	0	0	0	0	0	0	0	0	0	0	0	0	0	0	1	0	0	0	0	0	0	0	0	0	Fungi; Ascomycota; Sordariomycetes; Hypocreales; Clavicipitaceae; Metarhizium; Metarhizium_sp_Fkk26_1
New.ReferenceOTU6530	0	0	0	0	0	0	0	0	0	0	0	0	0	0	0	0	0	0	0	0	0	0	0	0	1	0	0	0	0	0	Fungi; Ascomycota; Sordariomycetes; Hypocreales; Clavicipitaceae; Metarhizium; Metarhizium_sp_Fkk26_1
SH093063.06FU_HQ880789_refs	0	0	0	0	0	0	0	0	0	0	0	0	0	0	0	0	0	5	0	0	0	0	0	0	0	0	0	0	0	0	Fungi; Ascomycota; Sordariomycetes; Hypocreales; Cordycipitaceae; Beauveria; Beauveria_australis
New.ReferenceOTU1701	0	0	0	0	1	0	0	0	0	0	0	0	0	0	0	0	0	0	0	0	0	0	0	0	0	0	0	0	0	0	Fungi; Ascomycota; Sordariomycetes; Hypocreales; Cordycipitaceae; Beauveria; Beauveria_sp
New.ReferenceOTU2855	0	0	0	0	0	0	0	0	0	0	0	0	0	0	0	0	0	0	1	0	0	0	0	0	0	0	0	0	0	0	Fungi; Ascomycota; Sordariomycetes; Hypocreales; Cordycipitaceae; Beauveria; Beauveria_sp
New.ReferenceOTU4757	0	0	0	0	0	0	0	0	0	1	0	0	0	0	0	0	0	0	0	0	0	0	0	0	0	0	0	0	0	0	Fungi; Ascomycota; Sordariomycetes; Hypocreales; Cordycipitaceae; Beauveria; Beauveria_sp
New.ReferenceOTU7729	0	0	0	0	0	0	0	0	0	0	0	0	1	1	0	0	7	0	0	0	0	0	0	0	0	0	0	0	0	0	Fungi; Ascomycota; Sordariomycetes; Hypocreales; Cordycipitaceae; Beauveria; Beauveria_sp
New.ReferenceOTU7778	0	0	0	0	0	0	0	0	0	0	0	0	0	0	0	0	2	0	0	0	0	0	0	0	0	0	0	0	0	0	Fungi; Ascomycota; Sordariomycetes; Hypocreales; Cordycipitaceae; Beauveria; Beauveria_sp
SH124354.06FU_KF435924_reps	0	0	0	0	0	0	0	0	0	0	0	0	0	0	0	0	0	0	1	0	0	0	0	0	0	0	0	0	0	0	Fungi; Ascomycota; Sordariomycetes; Hypocreales; Cordycipitaceae; Beauveria; Beauveria_sp
New.ReferenceOTU6642	0	0	0	0	0	0	0	0	0	0	0	0	0	3	0	0	1	0	0	0	0	0	1	0	0	0	1	0	0	0	Fungi; Ascomycota; Sordariomycetes; Hypocreales; Cordycipitaceae; Beauveria; Beauveria_sp_RSL_III
SH127926.06FU_FJ895306_reps	0	0	0	1	0	0	0	0	0	0	0	0	0	0	0	0	0	0	0	0	0	2	1	0	0	0	1	5	0	0	Fungi; Ascomycota; Sordariomycetes; Hypocreales; Cordycipitaceae; Beauveria; Beauveria_sp_RSL_III
SH093062.06FU_AF347612_refs	0	0	0	0	0	0	0	0	0	0	0	0	0	0	0	0	0	0	0	0	0	0	1	0	0	0	0	0	0	0	Fungi; Ascomycota; Sordariomycetes; Hypocreales; Cordycipitaceae; Cordyceps; Cordyceps_bassiana
New.ReferenceOTU3022	0	0	0	0	0	0	0	0	0	0	0	0	0	0	0	0	0	0	0	0	0	1	0	0	0	0	0	0	0	0	Fungi; Ascomycota; Sordariomycetes; Hypocreales; Cordycipitaceae; Cordyceps; Cordyceps_fratricida
New.ReferenceOTU4564	0	0	0	0	0	0	0	0	0	0	0	0	0	0	0	0	1	0	0	0	0	0	0	0	0	0	0	0	0	0	Fungi; Ascomycota; Sordariomycetes; Hypocreales; Cordycipitaceae; Cordyceps; Cordyceps_pleuricapitata
New.ReferenceOTU1710	0	0	0	0	1	0	0	0	0	0	0	0	0	0	0	0	0	0	0	0	0	0	0	0	0	0	0	0	0	0	Fungi; Ascomycota; Sordariomycetes; Hypocreales; Cordycipitaceae; Cordyceps; Cordyceps_sp_CUHK_CSM1
SH093068.06FU_AJ292429_refs	0	0	0	0	0	0	0	0	0	0	0	0	0	1	0	1	0	0	0	0	0	0	0	0	0	0	0	0	0	0	Fungi; Ascomycota; Sordariomycetes; Hypocreales; Cordycipitaceae; Lecanicillium; Lecanicillium_dimorphum
New.ReferenceOTU6368	0	0	0	0	0	0	0	0	0	1	0	0	0	0	0	0	0	0	0	0	0	0	0	0	0	0	0	0	0	0	Fungi; Ascomycota; Sordariomycetes; Hypocreales; Cordycipitaceae; Lecanicillium; Lecanicillium_sp
New.ReferenceOTU7560	0	0	0	0	0	0	0	0	0	0	0	0	0	0	0	0	1	0	0	0	0	0	0	0	0	0	0	0	0	0	Fungi; Ascomycota; Sordariomycetes; Hypocreales; Cordycipitaceae; Rotiferophthora; Rotiferophthora_minutispora
New.CleanUp.ReferenceOTU1305	0	0	0	0	0	0	0	0	0	0	0	0	0	0	0	0	0	0	0	0	0	0	1	0	0	0	0	0	0	0	Fungi; Ascomycota; Sordariomycetes; Hypocreales; Hypocreaceae; Cladobotryum; Cladobotryum_sp_MX535
New.ReferenceOTU1755	0	0	0	0	0	0	0	0	0	0	1	0	0	0	0	2	0	0	0	0	0	0	0	0	0	0	0	0	0	0	Fungi; Ascomycota; Sordariomycetes; Hypocreales; Hypocreaceae; Cladobotryum; Cladobotryum_sp_MX535
New.ReferenceOTU2457	0	0	0	0	0	0	0	0	0	0	0	0	0	0	0	0	0	0	1	0	0	0	0	0	0	0	0	0	0	0	Fungi; Ascomycota; Sordariomycetes; Hypocreales; Hypocreaceae; Hypocrea; Hypocrea_atrogelatinosa
New.ReferenceOTU2366	0	0	0	0	4	0	0	0	0	0	0	0	0	0	0	0	0	0	0	0	0	0	0	0	0	0	0	0	0	0	Fungi; Ascomycota; Sordariomycetes; Hypocreales; Hypocreaceae; Hypocrea; Hypocrea_avellanea
New.ReferenceOTU6952	0	0	0	0	0	0	0	0	0	0	0	0	0	0	0	0	0	0	0	1	0	0	0	0	0	0	0	0	0	0	Fungi; Ascomycota; Sordariomycetes; Hypocreales; Hypocreaceae; Hypocrea; Hypocrea_catoptron
New.ReferenceOTU384	0	0	0	0	0	0	0	0	0	0	0	0	0	0	0	0	0	0	0	0	0	0	0	0	0	0	0	0	2	0	Fungi; Ascomycota; Sordariomycetes; Hypocreales; Hypocreaceae; Hypocrea; Hypocrea_delicatula
New.ReferenceOTU1182	0	0	0	0	0	0	0	0	1	0	0	0	0	0	0	0	0	0	0	0	0	0	0	1	0	0	0	0	0	0	Fungi; Ascomycota; Sordariomycetes; Hypocreales; Hypocreaceae; Hypocrea; Hypocrea_epimyces
SH099953.06FU_AF408065_reps	0	0	0	0	0	0	0	0	1	0	0	0	0	0	0	0	0	0	0	0	0	0	0	0	0	0	0	0	0	0	Fungi; Ascomycota; Sordariomycetes; Hypocreales; Hypocreaceae; Hypocrea; Hypocrea_epimyces
SH111703.06FU_DQ835467_reps	0	0	0	0	0	0	0	0	0	0	0	0	0	0	0	0	0	0	0	1	0	0	0	0	0	0	0	0	0	0	Fungi; Ascomycota; Sordariomycetes; Hypocreales; Hypocreaceae; Hypocrea; Hypocrea_eucorticioides
New.ReferenceOTU3011	1	0	1	0	0	0	0	0	2	0	4	0	0	0	0	0	0	0	0	0	0	0	0	0	0	0	0	0	0	0	Fungi; Ascomycota; Sordariomycetes; Hypocreales; Hypocreaceae; Hypocrea; Hypocrea_megalocitrina
New.ReferenceOTU6538	0	0	0	0	0	0	0	0	0	0	0	0	0	0	0	0	0	0	0	0	0	0	1	0	0	0	0	0	0	0	Fungi; Ascomycota; Sordariomycetes; Hypocreales; Hypocreaceae; Hypocrea; Hypocrea_pilulifera
New.ReferenceOTU5492	0	0	0	0	0	0	0	0	0	0	0	0	0	0	0	0	0	0	1	0	0	0	0	0	0	0	0	0	0	0	Fungi; Ascomycota; Sordariomycetes; Hypocreales; Hypocreaceae; Hypocrea; Hypocrea_seppoi
New.ReferenceOTU1282	0	0	0	1	1	0	1	0	1	0	1	0	1	0	0	0	0	0	0	0	0	0	0	0	0	0	0	0	0	0	Fungi; Ascomycota; Sordariomycetes; Hypocreales; Hypocreaceae; Hypocrea; Hypocrea_sp_F31
SH019839.06FU_JF439479_reps_singleton	0	0	0	1	0	0	0	0	2	1	1	1	0	0	0	0	0	0	0	0	0	0	0	0	0	0	0	1	0	0	Fungi; Ascomycota; Sordariomycetes; Hypocreales; Hypocreaceae; Hypocrea; Hypocrea_sp_F31
New.ReferenceOTU5458	0	0	0	0	0	0	0	0	0	0	0	0	0	0	3	0	0	0	0	0	0	0	0	0	0	0	0	0	0	0	Fungi; Ascomycota; Sordariomycetes; Hypocreales; Hypocreaceae; Hypocrea; Hypocrea_spinulosa
New.ReferenceOTU6645	0	0	0	0	1	0	0	0	0	0	0	0	0	0	0	0	0	0	0	0	0	0	0	0	0	0	0	0	0	0	Fungi; Ascomycota; Sordariomycetes; Hypocreales; Hypocreaceae; Hypocrea; Hypocrea_voglmayrii
New.ReferenceOTU2947	0	0	0	0	0	0	0	0	0	0	0	0	0	0	0	0	0	0	0	0	0	0	0	0	0	3	0	0	0	0	Fungi; Ascomycota; Sordariomycetes; Hypocreales; Hypocreaceae; Hypomyces; Hypomyces_chlorinigenus
New.ReferenceOTU6118	0	0	0	0	0	0	0	0	0	0	0	0	0	0	0	0	0	0	4	0	0	0	0	0	0	0	0	0	0	0	Fungi; Ascomycota; Sordariomycetes; Hypocreales; Hypocreaceae; Hypomyces; Hypomyces_chlorinigenus
New.ReferenceOTU2412	0	0	0	0	0	0	0	0	1	1	0	0	0	0	0	0	0	0	0	0	0	0	0	0	0	0	0	9	1	0	Fungi; Ascomycota; Sordariomycetes; Hypocreales; Hypocreaceae; Hypomyces; Hypomyces_chrysospermus
New.ReferenceOTU2691	0	0	0	0	0	0	0	0	4	0	0	0	0	0	0	0	0	0	0	0	0	0	0	0	0	0	0	0	0	0	Fungi; Ascomycota; Sordariomycetes; Hypocreales; Hypocreaceae; Hypomyces; Hypomyces_chrysospermus
New.ReferenceOTU4854	0	0	0	0	0	0	0	0	0	0	0	0	0	0	0	0	0	0	0	0	1	0	0	0	0	0	0	0	0	0	Fungi; Ascomycota; Sordariomycetes; Hypocreales; Hypocreaceae; Hypomyces; Hypomyces_gabonensis
New.ReferenceOTU3565	0	0	0	0	0	0	0	0	0	0	0	0	0	0	0	0	0	0	0	0	0	0	0	0	0	0	0	0	0	2	Fungi; Ascomycota; Sordariomycetes; Hypocreales; Hypocreaceae; Hypomyces; Hypomyces_sp
New.CleanUp.ReferenceOTU117	0	0	0	0	0	0	0	0	0	0	0	0	0	0	0	1	0	0	0	0	0	0	0	0	0	0	0	0	0	0	Fungi; Ascomycota; Sordariomycetes; Hypocreales; Hypocreaceae; Hypomyces; Hypomyces_sp_15PA19
New.ReferenceOTU5336	1	0	1	0	0	0	0	1	1	0	0	0	0	0	0	0	0	0	0	0	0	1	0	5	0	0	0	0	0	1	Fungi; Ascomycota; Sordariomycetes; Hypocreales; Hypocreaceae; Hypomyces; Hypomyces_sp_15PA19
New.ReferenceOTU7800	0	0	0	0	0	0	0	0	0	0	0	0	0	0	1	0	0	0	0	0	0	0	0	0	1	0	0	0	0	0	Fungi; Ascomycota; Sordariomycetes; Hypocreales; Hypocreaceae; Hypomyces; Hypomyces_sp_15PA19
SH125503.06FU_GQ280547_refs	4	0	0	0	0	0	0	0	0	0	0	0	0	0	0	0	0	0	0	0	0	0	0	0	0	0	0	0	0	0	Fungi; Ascomycota; Sordariomycetes; Hypocreales; Nectriaceae; Calonectria; Calonectria_acicola
New.ReferenceOTU6945	0	0	0	1	0	0	0	0	0	0	0	0	0	0	0	0	0	0	0	0	0	1	0	0	0	0	0	0	0	0	Fungi; Ascomycota; Sordariomycetes; Hypocreales; Nectriaceae; Calonectria; Calonectria_angustata
New.ReferenceOTU2014	3	0	0	0	0	0	0	0	0	0	0	0	0	0	0	0	0	1	0	0	0	0	0	0	0	0	0	0	0	0	Fungi; Ascomycota; Sordariomycetes; Hypocreales; Nectriaceae; Calonectria; Calonectria_asiatica
SH125508.06FU_AF502827_reps	1	0	0	0	0	0	0	0	0	0	0	0	0	0	0	0	0	0	0	0	0	0	0	0	0	0	0	0	0	0	Fungi; Ascomycota; Sordariomycetes; Hypocreales; Nectriaceae; Calonectria; Calonectria_sp
New.ReferenceOTU3271	0	0	0	0	0	0	0	0	0	2	0	0	0	0	0	0	0	0	0	0	0	0	0	0	0	0	0	0	0	0	Fungi; Ascomycota; Sordariomycetes; Hypocreales; Nectriaceae; Cosmospora; Cosmospora_coccinea
New.ReferenceOTU917	0	0	0	0	0	0	0	0	0	0	0	0	0	0	0	0	0	0	1	3	0	0	0	0	0	0	0	0	0	0	Fungi; Ascomycota; Sordariomycetes; Hypocreales; Nectriaceae; Cosmospora; Cosmospora_sp
SH125859.06FU_JQ922192_reps	0	0	0	10	0	0	0	0	0	0	0	0	0	0	0	0	0	0	0	0	0	0	0	0	0	0	0	0	0	0	Fungi; Ascomycota; Sordariomycetes; Hypocreales; Nectriaceae; Cosmospora; Cosmospora_sp
New.ReferenceOTU1871	0	0	0	1	1	0	1	0	0	0	0	0	0	0	0	7	0	0	0	0	1	8	0	0	0	0	0	0	0	0	Fungi; Ascomycota; Sordariomycetes; Hypocreales; Nectriaceae; Cylindrocarpon; Cylindrocarpon_decumbens
New.ReferenceOTU2095	0	0	1	0	2	0	6	1	0	4	12	1	0	2	0	9	6	0	1	3	17	5	2	0	0	0	0	2	0	1	Fungi; Ascomycota; Sordariomycetes; Hypocreales; Nectriaceae; Cylindrocarpon; Cylindrocarpon_decumbens
New.ReferenceOTU2450	0	0	2	0	0	1	2	0	0	0	0	1	0	0	0	3	2	0	0	0	10	1	4	0	0	0	3	1	0	5	Fungi; Ascomycota; Sordariomycetes; Hypocreales; Nectriaceae; Cylindrocarpon; Cylindrocarpon_decumbens
New.ReferenceOTU2879	0	0	0	0	0	0	0	0	0	0	0	0	0	0	0	0	0	0	0	0	3	0	1	0	0	0	0	0	0	1	Fungi; Ascomycota; Sordariomycetes; Hypocreales; Nectriaceae; Cylindrocarpon; Cylindrocarpon_decumbens
New.ReferenceOTU3079	0	0	2	0	1	1	2	0	0	0	0	1	0	2	0	1	2	0	0	0	5	0	2	0	0	0	2	0	0	2	Fungi; Ascomycota; Sordariomycetes; Hypocreales; Nectriaceae; Cylindrocarpon; Cylindrocarpon_decumbens
New.ReferenceOTU3525	0	0	10	0	1	0	4	0	1	1	0	5	0	0	0	5	5	0	0	3	41	5	8	0	0	0	5	0	0	8	Fungi; Ascomycota; Sordariomycetes; Hypocreales; Nectriaceae; Cylindrocarpon; Cylindrocarpon_decumbens
New.ReferenceOTU5059	0	0	6	0	0	0	3	0	0	0	3	0	0	1	0	3	2	0	0	1	6	0	4	0	0	0	3	0	0	2	Fungi; Ascomycota; Sordariomycetes; Hypocreales; Nectriaceae; Cylindrocarpon; Cylindrocarpon_decumbens
New.ReferenceOTU6760	0	0	43	3	3	0	55	0	4	3	3	16	0	51	0	18	31	0	4	13	104	8	40	1	0	0	4	18	0	21	Fungi; Ascomycota; Sordariomycetes; Hypocreales; Nectriaceae; Cylindrocarpon; Cylindrocarpon_decumbens
SH118581.06FU_JN100643_refs	0	0	0	0	0	0	0	0	0	0	0	0	0	0	0	0	0	0	0	2	0	0	0	0	0	0	0	0	0	0	Fungi; Ascomycota; Sordariomycetes; Hypocreales; Nectriaceae; Cylindrocladiella; Cylindrocladiella_variabilis
New.ReferenceOTU3128	0	0	0	0	0	0	0	0	0	0	0	0	2	0	0	2	0	0	1	0	0	0	0	0	0	0	0	0	0	0	Fungi; Ascomycota; Sordariomycetes; Hypocreales; Nectriaceae; Fusarium; Fusarium_merismoides_var_crassum
New.ReferenceOTU3165	0	0	0	0	0	0	0	0	0	0	0	0	0	0	0	0	1	0	0	0	0	0	0	0	0	0	0	0	0	0	Fungi; Ascomycota; Sordariomycetes; Hypocreales; Nectriaceae; Fusarium; Fusarium_merismoides_var_crassum
New.ReferenceOTU7135	0	0	0	0	0	0	0	0	0	0	0	0	0	0	0	0	0	0	0	0	0	0	1	0	0	0	0	0	0	0	Fungi; Ascomycota; Sordariomycetes; Hypocreales; Nectriaceae; Fusarium; Fusarium_merismoides_var_crassum
New.ReferenceOTU7971	0	0	1	0	0	0	0	0	0	0	0	0	0	0	0	0	0	0	0	2	0	0	0	0	0	0	0	0	0	0	Fungi; Ascomycota; Sordariomycetes; Hypocreales; Nectriaceae; Fusarium; Fusarium_merismoides_var_crassum

SH118562.06FU_AB587006_refs	0	0	0	1	0	0	0	0	0	0	0	0	0	0	0	0	0	0	0	0	0	0	0	0	0	0	0	0	0	0	Fungi; Ascomycota; Sordariomycetes; Hypocreales; Nectriaceae; Gibberella; Fusarium_subglutinans
SH118566.06FU_AY928413_reps	0	0	0	0	0	0	0	0	0	0	0	0	0	0	0	0	0	0	1	0	0	0	1	0	0	0	0	0	0	0	Fungi; Ascomycota; Sordariomycetes; Hypocreales; Nectriaceae; Gibberella; Fusarium_subglutinans
SH118568.06FU_GU257902_reps	0	0	0	0	0	0	0	0	0	0	6	0	3	0	0	0	0	0	0	0	0	0	0	0	0	0	0	0	0	0	Fungi; Ascomycota; Sordariomycetes; Hypocreales; Nectriaceae; Gibberella; Fusarium_subglutinans
SH092357.06FU_HQ649905_reps	0	0	2	0	0	0	2	0	0	0	0	3	1	2	1	1	10	0	0	0	0	0	0	0	2	0	0	0	0	0	Fungi; Ascomycota; Sordariomycetes; Hypocreales; Nectriaceae; Gibberella; Gibberella_intricans
SH118560.06FU_GQ505688_refs	0	0	0	0	0	0	0	0	0	0	0	0	1	0	0	0	4	0	0	0	0	0	2	0	0	0	0	0	0	0	Fungi; Ascomycota; Sordariomycetes; Hypocreales; Nectriaceae; Gibberella; Gibberella_intricans
New.ReferenceOTU1715	0	0	0	0	0	0	0	0	0	0	0	0	0	0	0	0	0	0	0	0	0	0	0	0	3	0	0	0	0	0	Fungi; Ascomycota; Sordariomycetes; Hypocreales; Nectriaceae; Nectria; Nectria_albida
New.ReferenceOTU265	0	0	0	0	2	0	0	0	0	0	0	0	0	0	0	0	0	0	0	0	0	0	0	0	0	0	0	0	0	0	Fungi; Ascomycota; Sordariomycetes; Hypocreales; Nectriaceae; Nectria; Nectria_bactridioides
New.ReferenceOTU3944	0	0	0	0	0	0	0	0	0	0	1	0	0	0	0	0	0	0	0	0	0	0	0	0	0	0	0	0	9	0	Fungi; Ascomycota; Sordariomycetes; Hypocreales; Nectriaceae; Nectria; Nectria_bactridioides
New.ReferenceOTU5195	0	0	0	0	0	0	0	0	0	0	0	0	0	0	0	0	0	0	1	0	0	0	0	0	0	0	0	0	0	0	Fungi; Ascomycota; Sordariomycetes; Hypocreales; Nectriaceae; Nectria; Nectria_bactridioides
New.ReferenceOTU7761	0	0	0	0	0	0	0	0	0	0	0	1	0	0	0	1	1	0	0	0	0	0	0	0	0	0	0	0	0	0	Fungi; Ascomycota; Sordariomycetes; Hypocreales; Nectriaceae; Nectria; Nectria_bactridioides
New.ReferenceOTU2463	0	0	0	0	0	0	0	0	0	0	0	0	0	0	0	0	0	0	0	0	0	0	0	0	0	0	0	0	0	1	Fungi; Ascomycota; Sordariomycetes; Hypocreales; Nectriaceae; Nectria; Nectria_cinnabarina
New.ReferenceOTU2719	0	0	0	0	0	0	0	0	0	0	0	0	0	1	0	0	0	0	0	0	0	0	0	0	0	0	0	0	0	0	Fungi; Ascomycota; Sordariomycetes; Hypocreales; Nectriaceae; Nectria; Nectria_magnispora
New.ReferenceOTU7780	0	0	0	0	0	1	0	0	0	0	0	0	0	0	0	0	0	0	0	0	0	0	0	0	0	0	0	1	0	4	Fungi; Ascomycota; Sordariomycetes; Hypocreales; Nectriaceae; Nectria; Nectria_mariae
New.ReferenceOTU2177	0	0	0	0	0	0	0	0	0	0	0	0	0	0	0	2	0	0	0	0	0	0	0	0	0	0	0	0	0	0	Fungi; Ascomycota; Sordariomycetes; Hypocreales; Nectriaceae; Nectria; Nectria_mauritiicola
New.ReferenceOTU3413	9	2	1	2	0	0	9	0	4	6	8	0	0	0	0	49	0	1	3	9	3	0	1	3	0	1	0	0	3	1	Fungi; Ascomycota; Sordariomycetes; Hypocreales; Nectriaceae; Nectria; Nectria_mauritiicola
New.ReferenceOTU7065	0	6	9	0	0	6	3	5	0	4	4	0	0	7	0	19	1	0	0	0	52	0	1	0	3	0	0	0	0	13	Fungi; Ascomycota; Sordariomycetes; Hypocreales; Nectriaceae; Nectria; Nectria_mauritiicola
New.ReferenceOTU3998	0	0	0	0	0	0	1	0	0	0	0	0	0	0	0	0	0	0	0	0	0	0	0	0	0	0	0	0	0	0	Fungi; Ascomycota; Sordariomycetes; Hypocreales; Nectriaceae; Nectria; Nectria_pseudotrichia
New.ReferenceOTU4512	0	1	0	0	0	0	0	0	1	0	0	0	0	0	0	0	0	0	1	0	0	0	0	0	0	0	0	0	0	0	Fungi; Ascomycota; Sordariomycetes; Hypocreales; Nectriaceae; Nectria; Nectria_pseudotrichia
SH118606.06FU_JN995626_reps	0	0	0	0	0	0	0	0	0	0	0	0	0	0	0	0	0	0	0	0	0	0	0	1	0	0	0	0	0	0	Fungi; Ascomycota; Sordariomycetes; Hypocreales; Nectriaceae; Nectria; Nectria_pseudotrichia
SH118613.06FU_EF121865_reps	0	0	2	0	0	0	0	0	0	0	0	0	0	0	0	0	0	0	1	0	0	0	0	0	0	0	0	0	0	0	Fungi; Ascomycota; Sordariomycetes; Hypocreales; Nectriaceae; Nectria; Nectria_pseudotrichia
SH118630.06FU_HM054154_reps	0	0	0	0	0	0	0	0	0	0	0	0	0	0	0	0	0	0	0	0	0	0	0	1	0	0	0	0	0	0	Fungi; Ascomycota; Sordariomycetes; Hypocreales; Nectriaceae; Nectria; Nectria_pseudotrichia
New.CleanUp.ReferenceOTU194	0	0	0	0	0	0	0	0	0	0	0	0	0	0	0	0	0	0	0	1	0	0	0	0	0	0	0	0	0	0	Fungi; Ascomycota; Sordariomycetes; Hypocreales; Nectriaceae; Nectria; Nectria_rubropeziza
New.ReferenceOTU1859	0	0	4	2	0	0	2	0	0	2	1	1	0	0	0	9	1	0	2	3	14	1	1	0	0	0	1	3	1	3	Fungi; Ascomycota; Sordariomycetes; Hypocreales; Nectriaceae; Nectria; Nectria_rubropeziza
New.ReferenceOTU2046	0	0	0	0	0	0	0	0	0	3	0	0	0	0	0	0	0	0	0	0	0	0	0	0	0	0	0	0	0	0	Fungi; Ascomycota; Sordariomycetes; Hypocreales; Nectriaceae; Nectria; Nectria_rubropeziza
New.ReferenceOTU2112	1	0	0	0	0	0	1	0	0	0	0	0	0	0	0	0	0	0	1	0	0	0	0	0	0	0	0	0	0	0	Fungi; Ascomycota; Sordariomycetes; Hypocreales; Nectriaceae; Nectria; Nectria_rubropeziza
New.ReferenceOTU4157	0	0	1	0	0	0	0	0	0	0	0	0	0	0	0	0	0	0	0	0	1	0	0	0	0	0	0	0	0	0	Fungi; Ascomycota; Sordariomycetes; Hypocreales; Nectriaceae; Nectria; Nectria_rubropeziza
New.ReferenceOTU4634	0	0	1	0	0	0	0	0	0	0	0	0	0	0	0	0	0	0	0	0	0	0	0	0	0	0	0	0	0	0	Fungi; Ascomycota; Sordariomycetes; Hypocreales; Nectriaceae; Nectria; Nectria_rubropeziza
New.ReferenceOTU4713	0	0	0	0	0	0	0	0	0	0	0	0	0	0	1	0	0	0	0	1	0	0	0	0	0	0	0	0	0	0	Fungi; Ascomycota; Sordariomycetes; Hypocreales; Nectriaceae; Nectria; Nectria_rubropeziza
New.ReferenceOTU5248	0	0	0	0	0	0	0	0	0	0	0	0	0	0	0	0	0	0	1	0	0	0	0	0	0	0	0	0	0	0	Fungi; Ascomycota; Sordariomycetes; Hypocreales; Nectriaceae; Nectria; Nectria_rubropeziza
New.ReferenceOTU5393	11	17	4	16	0	2	30	0	34	31	35	1	3	4	8	84	16	4	4	71	28	0	4	16	3	8	0	9	11	0	Fungi; Ascomycota; Sordariomycetes; Hypocreales; Nectriaceae; Nectria; Nectria_rubropeziza
New.ReferenceOTU6515	0	0	0	1	0	0	0	0	0	0	2	0	0	5	0	8	5	0	2	8	1	0	2	0	0	0	0	0	0	0	Fungi; Ascomycota; Sordariomycetes; Hypocreales; Nectriaceae; Nectria; Nectria_rubropeziza
New.ReferenceOTU6575	0	0	0	0	0	0	0	0	0	0	0	0	0	0	0	0	0	0	1	0	0	0	0	0	0	0	0	0	0	0	Fungi; Ascomycota; Sordariomycetes; Hypocreales; Nectriaceae; Nectria; Nectria_rubropeziza
New.ReferenceOTU6982	0	0	0	0	0	0	0	0	0	0	0	0	0	0	0	1	0	0	0	0	0	0	0	0	0	0	0	0	0	0	Fungi; Ascomycota; Sordariomycetes; Hypocreales; Nectriaceae; Nectria; Nectria_rubropeziza
New.ReferenceOTU750	0	0	0	0	0	0	0	0	0	0	0	0	0	0	0	0	0	0	0	1	0	0	0	0	0	0	0	0	0	0	Fungi; Ascomycota; Sordariomycetes; Hypocreales; Nectriaceae; Nectria; Nectria_rubropeziza
New.ReferenceOTU7882	0	0	0	0	0	0	0	0	0	0	0	0	0	0	0	0	0	0	1	0	0	0	0	0	0	0	0	0	0	0	Fungi; Ascomycota; Sordariomycetes; Hypocreales; Nectriaceae; Nectria; Nectria_rubropeziza
New.CleanUp.ReferenceOTU413	0	0	0	0	0	0	0	0	0	0	1	0	0	0	0	0	0	0	0	0	0	0	0	0	0	0	0	0	0	0	Fungi; Ascomycota; Sordariomycetes; Hypocreales; Nectriaceae; Nectria; Nectria_sp
New.ReferenceOTU1380	0	0	0	0	0	0	2	0	13	0	0	0	0	0	0	2	0	0	3	1	1	0	0	1	0	0	0	0	0	1	Fungi; Ascomycota; Sordariomycetes; Hypocreales; Nectriaceae; Nectria; Nectria_sp
New.ReferenceOTU1404	0	1	0	0	0	0	1	0	3	5	1	0	0	0	0	22	1	1	1	5	2	0	0	1	1	0	0	0	1	0	Fungi; Ascomycota; Sordariomycetes; Hypocreales; Nectriaceae; Nectria; Nectria_sp
New.ReferenceOTU1620	0	1	0	0	0	0	0	0	0	0	0	0	0	0	1	2	1	0	0	3	0	0	0	0	0	0	0	0	2	0	Fungi; Ascomycota; Sordariomycetes; Hypocreales; Nectriaceae; Nectria; Nectria_sp
New.ReferenceOTU1993	0	0	0	0	0	0	0	0	0	0	0	0	0	0	0	0	0	2	15	0	0	0	0	0	0	0	0	0	0	0	Fungi; Ascomycota; Sordariomycetes; Hypocreales; Nectriaceae; Nectria; Nectria_sp
New.ReferenceOTU2159	0	0	0	0	1	0	0	0	0	0	0	0	0	0	0	0	0	0	0	0	0	0	0	0	0	0	0	0	0	0	Fungi; Ascomycota; Sordariomycetes; Hypocreales; Nectriaceae; Nectria; Nectria_sp
New.ReferenceOTU2216	0	0	2	0	0	3	10	0	2	1	1	0	0	0	0	4	2	0	3	0	28	1	3	0	0	0	3	4	0	4	Fungi; Ascomycota; Sordariomycetes; Hypocreales; Nectriaceae; Nectria; Nectria_sp
New.ReferenceOTU2311	1	0	0	0	0	0	0	0	5	0	0	0	0	0	0	2	0	0	0	0	0	0	0	0	0	0	0	0	0	0	Fungi; Ascomycota; Sordariomycetes; Hypocreales; Nectriaceae; Nectria; Nectria_sp
New.ReferenceOTU2676	0	0	0	0	0	0	0	0	0	0	0	0	0	0	0	0	0	0	0	0	0	0	0	0	0	0	0	0	1	0	Fungi; Ascomycota; Sordariomycetes; Hypocreales; Nectriaceae; Nectria; Nectria_sp
New.ReferenceOTU2814	2	6	0	1	0	0	2	0	2	3	0	0	0	0	1	13	0	0	2	2	1	0	0	2	0	1	0	0	3	0	Fungi; Ascomycota; Sordariomycetes; Hypocreales; Nectriaceae; Nectria; Nectria_sp
New.ReferenceOTU3597	1	0	0	0	0	0	0	0	0	0	0	0	0	0	0	0	0	0	0	0	0	0	0	0	0	0	0	0	0	0	Fungi; Ascomycota; Sordariomycetes; Hypocreales; Nectriaceae; Nectria; Nectria_sp
New.ReferenceOTU4278	0	0	0	0	0	0	2	0	0	0	0	0	0	1	0	0	1	0	0	0	6	0	1	0	0	0	0	1	0	0	Fungi; Ascomycota; Sordariomycetes; Hypocreales; Nectriaceae; Nectria; Nectria_sp
New.ReferenceOTU4644	0	0	0	0	0	0	0	0	2	0	0	0	0	0	0	1	0	0	0	0	0	0	0	0	0	0	0	0	0	0	Fungi; Ascomycota; Sordariomycetes; Hypocreales; Nectriaceae; Nectria; Nectria_sp
New.ReferenceOTU5070	0	0	0	0	0	0	0	0	0	0	0	0	0	0	0	0	0	0	0	1	0	0	0	0	0	0	0	0	0	0	Fungi; Ascomycota; Sordariomycetes; Hypocreales; Nectriaceae; Nectria; Nectria_sp
New.ReferenceOTU5645	0	0	0	0	0	0	0	0	0	0	0	0	0	0	0	0	0	0	0	0	0	0	0	0	0	0	0	0	1	0	Fungi; Ascomycota; Sordariomycetes; Hypocreales; Nectriaceae; Nectria; Nectria_sp
New.ReferenceOTU5731	0	0	1	0	0	1	0	0	0	1	0	0	0	0	1	1	0	0	0	0	21	0	0	0	1	0	0	0	0	1	Fungi; Ascomycota; Sordariomycetes; Hypocreales; Nectriaceae; Nectria; Nectria_sp
New.ReferenceOTU6074	0	0	0	0	0	0	0	0	0	0	0	0	0	0	0	0	0	0	0	0	0	3	0	0	0	0	0	0	0	0	Fungi; Ascomycota; Sordariomycetes; Hypocreales; Nectriaceae; Nectria; Nectria_sp
New.ReferenceOTU6790	0	1	0	0	0	0	0	0	0	0	0	0	0	0	0	0	0	0	0	0	0	0	0	0	0	0	0	0	0	0	Fungi; Ascomycota; Sordariomycetes; Hypocreales; Nectriaceae; Nectria; Nectria_sp
New.ReferenceOTU6815	0	0	0	0	0	0	0	0	0	0	0	0	0	0	0	0	0	0	0	0	0	0	0	0	0	0	0	0	3	0	Fungi; Ascomycota; Sordariomycetes; Hypocreales; Nectriaceae; Nectria; Nectria_sp
New.ReferenceOTU947	0	0	0	0	0	0	0	0	0	0	0	0	0	1	0	0	0	0	0	0	0	0	0	0	0	0	0	0	0	0	Fungi; Ascomycota; Sordariomycetes; Hypocreales; Nectriaceae; Nectria; Nectria_sp
SH035598.06FU_HQ607876_reps_singleton	0	0	0	0	0	0	0	0	0	0	0	0	0	0	0	0	2	0	0	0	0	0	0	0	0	0	0	0	0	0	Fungi; Ascomycota; Sordariomycetes; Hypocreales; Nectriaceae; Nectria; Nectria_sp
New.ReferenceOTU7527	0	0	2	0	0	0	0	0	0	0	0	0	0	0	0	0	0	0	0	0	0	0	0	0	0	0	0	0	0	0	Fungi; Ascomycota; Sordariomycetes; Hypocreales; Nectriaceae; Nectria; Nectria_sp_HLS206
New.ReferenceOTU6679	0	0	0	0	0	0	0	0	0	0	0	0	0	0	0	1	0	0	0	0	0	0	0	0	0	0	0	0	0	0	Fungi; Ascomycota; Sordariomycetes; Hypocreales; Nectriaceae; Nectria; Nectria_sp_ICMP_13358
New.ReferenceOTU302	0	0	0	0	0	0	0	0	0	0	0	0	0	0	0	0	0	0	0	2	0	0	0	0	0	0	0	0	0	0	Fungi; Ascomycota; Sordariomycetes; Hypocreales; Nectriaceae; Nectria; Nectria_ventricosa
New.ReferenceOTU124	0	1	0	0	0	0	0	0	0	0	0	0	0	0	0	0	0	0	0	0	0	0	0	0	0	0	0	0	0	0	Fungi; Ascomycota; Sordariomycetes; Hypocreales; Nectriaceae; Volutella; Volutella_sp
New.ReferenceOTU2528	0	0	0	0	1	0	0	0	0	0	0	0	0	0	0	0	0	0	0	0	0	0	0	0	0	0	0	0	0	0	Fungi; Ascomycota; Sordariomycetes; Hypocreales; Nectriaceae; Volutella; Volutella_sp
New.ReferenceOTU2563	0	0	0	0	3	1	0	0	1	0	0	0	0	0	0	0	0	0	0	0	0	0	0	0	1	0	2	0	0	0	Fungi; Ascomycota; Sordariomycetes; Hypocreales; Nectriaceae; Volutella; Volutella_sp
New.ReferenceOTU4936	0	0	1	0	0	0	0	0	0	0	0	0	1	0	0	3	0	0	0	0	0	0	0	0	0	0	0	0	0	0	Fungi; Ascomycota; Sordariomycetes; Hypocreales; Nectriaceae; Volutella; Volutella_sp
New.ReferenceOTU5864	0	0	0	0	0	0	0	0	3	0	0	0	0	0	0	0	0	0	0	0	0	0	0	0	0	0	0	0	0	0	Fungi; Ascomycota; Sordariomycetes; Hypocreales; Nectriaceae; Volutella; Volutella_sp
New.ReferenceOTU6576	0	0	0	0	0	0	0	0	2	0	0	0	0	0	0	0	0	0	1	0	0	0	0	0	0	0	0	0	0	0	Fungi; Ascomycota; Sordariomycetes; Hypocreales; Nectriaceae; Volutella; Volutella_sp
New.ReferenceOTU487	0	0	0	2	0	0	0	0	0	0	0	0	0	0	0	0	0	0	1	0	0	0	0	0	0	0	0	0	0	0	Fungi; Ascomycota; Sordariomycetes; Hypocreales; Nectriaceae; Volutella; Volutella_sp_CABI_IMI265311
SH033743.06FU_JQ693161_reps_singleton	0	0	0	5	0	0	0	0	0	0	0	0	0	0	0	0	0	0	0	0	0	0	0	0	0	0	0	0	0	0	Fungi; Ascomycota; Sordariomycetes; Hypocreales; Nectriaceae; Volutella; Volutella_sp_CABI_IMI265311
New.ReferenceOTU1210	0	0	0	0	0	0	0	0	0	0	0	0	0	0	0	0	0	0	1	0	0	0	0	0	0	0	0	0	0	0	Fungi; Ascomycota; Sordariomycetes; Hypocreales; Nectriaceae; Volutella; Volutella_sp_DoF21
SH140184.06FU_JQ388266_reps	0	0	0	0	0	0	0	0	0	0	0	0	0	0	0	0	0	0	0	0	0	6	0	0	0	0	0	0	0	0	Fungi; Ascomycota; Sordariomycetes; Hypocreales; Nectriaceae; Volutella; Volutella_sp_DoF21
New.ReferenceOTU1983	0	0	0	0	0	0	0	0	0	0	5	0	0	0	0	0	0	0	0	0	0	0	0	0	0	0	0	0	0	0	Fungi; Ascomycota; Sordariomycetes; Hypocreales; Ophiocordycipitaceae; Chaunopycnis; Chaunopycnis_pustulata
New.ReferenceOTU6574	0	0	0	0	0	0	0	0	0	0	0	0	0	1	0	0	0	0	0	0	0	0	0	0	0	0	0	0	0	0	Fungi; Ascomycota; Sordariomycetes; Hypocreales; Ophiocordycipitaceae; Elaphocordyceps; Elaphocordyceps_capitata
New.ReferenceOTU7694	0	2	0	0	0	0	0	0	0	0	0	0	0	0	0	0	0	0	0	0	0	0	0	0	0	0	0	0	0	0	Fungi; Ascomycota; Sordariomycetes; Hypocreales; Ophiocordycipitaceae; Elaphocordyceps; Elaphocordyceps_sp_ENF7
New.ReferenceOTU2140	0	0	0	0	0	0	0	0	0	0	0	0	0	0	0	0	1	0	0	0	0	0	0	0	0	0	0	2	0	1	Fungi; Ascomycota; Sordariomycetes; Hypocreales; Ophiocordycipitaceae; Haptocillium; Haptocillium_glocklingiae
New.ReferenceOTU2498	0	0	0	0	0	0	0	0	0	0	0	0	0	0	0	0	0	0	0	0	0	0	0	0	0	0	0	0	0	2	Fungi; Ascomycota; Sordariomycetes; Hypocreales; Ophiocordycipitaceae; Haptocillium; Haptocillium_glocklingiae
New.ReferenceOTU3594	0	0	0	0	1	0	0	0	0	0	0	0	0	0	0	0	0	2	0	0	0	0	0	0	0	0	2	0	0	0	Fungi; Ascomycota; Sordariomycetes; Hypocreales; Ophiocordycipitaceae; Haptocillium; Haptocillium_glocklingiae
New.ReferenceOTU5874	0	0	0	0	1	0	0	0	0	0	0	0	0	0	0	0	0	0	0	0	0	0	1	0	0	0	0	0	0	0	Fungi; Ascomycota; Sordariomycetes; Hypocreales; Ophiocordycipitaceae; Haptocillium; Haptocillium_glocklingiae
New.ReferenceOTU6073	0	0	0	0	0	0	0	0	0	0	0	0	0	0	0	0	0	1	0	0	0	0	0	0	0	0	0	0	0	0	Fungi; Ascomycota; Sordariomycetes; Hypocreales; Ophiocordycipitaceae; Haptocillium; Haptocillium_glocklingiae
New.ReferenceOTU6249	0	0	0	0	0	1	0	0	0	0	0	0	0	0	0	0	0	0	0	0	0	0	0	0	0	0	5	0	0	1	Fungi; Ascomycota; Sordariomycetes; Hypocreales; Ophiocordycipitaceae; Haptocillium; Haptocillium_glocklingiae
New.ReferenceOTU2530	0	0	0	0	0	1	0	0	0	0	0	0	0	0	0	0	0	0	0	1	0	0	0	0	0	0	1	0	0	0	Fungi; Ascomycota; Sordariomycetes; Hypocreales; Ophiocordycipitaceae; Hirsutella; Hirsutella_sp_ICMP14250
New.ReferenceOTU4678	0	0	0	0	0	0	0	0	0	0	0	0	0	0	0	0	0	0	0	0	0	0	2	0	0	0	0	0	0	0	Fungi; Ascomycota; Sordariomycetes; Hypocreales; Ophiocordycipitaceae; Hirsutella; Hirsutella_sp_RS_2012
New.ReferenceOTU3272	0	0	1	0	0	0	0	0	0	0	0	0	0	0	0	0	0	0	0	0	0	0	0	0	0	0	0	0	0	0	Fungi; Ascomycota; Sordariomycetes; Hypocreales; Ophiocordycipitaceae; Hirsutella; Hirsutella_uncinata
New.ReferenceOTU4621	0	0	0	0	0	0	0	1	0	0	0	0	1	0	0	0	0	0	0	0	0	0	0	0	0	0	0	0	0	0	Fungi; Ascomycota; Sordariomycetes; Hypocreales; Ophiocordycipitaceae; Hirsutella; Hirsutella_uncinata
New.ReferenceOTU6279	0	0	0	0	0	0	0	0	0	0	0	0	0	0	0	0	0	1	0	3	0	0	0	0	0	0	0	0	0	0	Fungi; Ascomycota; Sordariomycetes; Hypocreales; Ophiocordycipitaceae; Hirsutella; Hirsutella_uncinata
New.ReferenceOTU6741	0	0	0	0	0	0	0	0	0	0	0	0	0	1	0	0	0	0	0	0	0	0	0	0	0	0	0	0	0	0	Fungi; Ascomycota; Sordariomycetes; Hypocreales; Ophiocordycipitaceae; Hirsutella; Hirsutella_uncinata
SH126456.06FU_FR734106_refs	0	0	0	0	0	0	0	0	0	0	0	0	0	0	0	0	0	0	0	0	0	1	0	0	0	0	0	0	0	0	Fungi; Ascomycota; Sordariomycetes; Hypocreales; Ophiocordycipitaceae; Purpureocillium; Purpureocillium_lavendulum
New.ReferenceOTU1157	0	0	0	0	0	0	0	0	0	0	0	0	0	0	0	0	0	0	0	0	0	0	0	0	0	0	1	0	0	0	Fungi; Ascomycota; Sordariomycetes; Incertae_sedis; Glomerellaceae; Colletotrichum; Colletotrichum_acutatum
SH133127.06FU_GU227894_refs	0	0	0	0	0	0	0	0	0	0	0	0	0	0	1	0	0	0	0	0	0	0	0	0	0	0	0	0	0	0	Fungi; Ascomycota; Sordariomycetes; Incertae_sedis; Glomerellaceae; Colletotrichum; Colletotrichum_chlorophyti
New.ReferenceOTU7737	0	0	0	0	0	0	0	0	0	0	0	0	0	0	0	0	0	1	0	0	0	0	0	0	0	0	0	0	0	0	Fungi; Ascomycota; Sordariomycetes; Incertae_sedis; Glomerellaceae; Colletotrichum; Colletotrichum_circinans
SH137701.06FU_KC293581_refs	0	0	0	0	0	0	2	0	0	0	0	0	0	0	0	0	0	0	0	0	0	0	0	0	0	0	0	0	0	0	Fungi; Ascomycota; Sordariomycetes; Incertae_sedis; Glomerellaceae; Colletotrichum; Colletotrichum_citri
New.ReferenceOTU4455	0	0	0	0	0	0	0	0	0	0	0	0	0	0	0	0	0	0	2	1	0	0	0	0	0	0	0	0	0	0	Fungi; Ascomycota; Sordariomycetes; Incertae_sedis; Glomerellaceae; Colletotrichum; Colletotrichum_sp
New.ReferenceOTU6253	0	0	0	0	0	0	0	0	0	0	0	0	0	0	0	0	0	0	0	0	0	0	1	0	1	0	0	0	0	0	Fungi; Ascomycota; Sordariomycetes; Incertae_sedis; Glomerellaceae; Colletotrichum; Colletotrichum_sp
New.ReferenceOTU6990	0	0	0	0	0	0	0	0	0	0	0	0	0	0	0	2	1	0	0	0	0	0	0	0	1	0	0	0	0	0	Fungi; Ascomycota; Sordariomycetes; Incertae_sedis; Glomerellaceae; Colletotrichum; Colletotrichum_sp
New.ReferenceOTU7949	0	0	0	0	0	0	0	0	0	0	0	0	0	0	0	0	0	0	0	0	0	0	0	1	0	0	0	3	0	0	Fungi; Ascomycota; Sordariomycetes; Incertae_sedis; Glomerellaceae; Colletotrichum; Colletotrichum_sp
SH038128.06FU_EU726301_reps_singleton	0	0	0	0	0	0	0	0	0	0	0	0	0	0	0	0	1	0	0	0	0	0	0	0	0	0	0	0	0	0	Fungi; Ascomycota; Sordariomycetes; Incertae_sedis; Glomerellaceae; Colletotrichum; Colletotrichum_sp
New.ReferenceOTU1771	0	3	0	4	0	1	0	0	0	2	0	0	1	0	0	3	1	0	0	0	0	1	0	0	0	1	0	0	3	0	Fungi; Ascomycota; Sordariomycetes; Incertae_sedis; Glomerellaceae; Colletotrichum; Colletotrichum_sp_JP27
SH122070.06FU_AF411767_reps	0	0	1	1	0	0	0	0	0	0	0	0	0	0	0	0	0	0	0	0	0	0	0	0	0	0	0	0	0	0	Fungi; Ascomycota; Sordariomycetes; Incertae_sedis; Glomerellaceae; Colletotrichum; Colletotrichum_theobromicola
New.ReferenceOTU3359	0	0	0	2	0	2	0	0	0	2	0	0	0	0	0	0	1	0	0	0	0	0	0	0	0	0	0	0	0	0	Fungi; Ascomycota; Sordariomycetes; Incertae_sedis; Glomerellaceae; Glomerella; Glomerella_cingulata
New.ReferenceOTU4078	0	0	0	0	0	0	0	0	0	0	0	0	0	0	0	0	0	0	0	1	0	0	0	0	0	0	0	0	0	0	Fungi; Ascomycota; Sordariomycetes; Incertae_sedis; Magnaporthaceae; Gaeumannomyces; Gaeumannomyces_cylindrosporus
New.ReferenceOTU4286	0	0	0	0	0	0	0	0	0	0	1	0	0	0	0	0	0	0	0	0	0	0	0	0	0	0	0	0	0	0	Fungi; Ascomycota; Sordariomycetes; Incertae_sedis; Magnaporthaceae; Mycoleptodiscus; Mycoleptodiscus_sp
New.ReferenceOTU5783	5	0	0	1	0	0	0	0	0	0	0	0	0	0	0	0	0	0	0	0	0	0	0	0	0	0	0	0	0	0	Fungi; Ascomycota; Sordariomycetes; Incertae_sedis; Magnaporthaceae; Mycoleptodiscus; Mycoleptodiscus_sp

SH114616.06FU_AJ542526_reps	1	0	0	0	0	0	0	0	0	0	0	0	0	0	0	0	0	0	0	0	0	0	0	0	0	0	0	0	0	0	Fungi; Ascomycota; Sordariomycetes; Incertae_sedis; Magnaporthaceae; Mycoleptodiscus; Mycoleptodiscus_sp
New.ReferenceOTU1459	0	0	0	0	0	0	0	0	0	2	0	0	0	0	0	0	0	0	0	0	0	0	0	0	0	0	0	0	0	0	Fungi; Ascomycota; Sordariomycetes; Incertae_sedis; Magnaporthaceae; Pyricularia; Pyricularia_commelinicola
New.ReferenceOTU3712	0	0	0	0	0	0	0	0	0	0	0	1	0	0	0	0	0	0	0	0	0	0	0	0	0	0	0	0	0	0	Fungi; Ascomycota; Sordariomycetes; Incertae_sedis; Magnaporthaceae; Pyricularia; Pyricularia_longispora
New.ReferenceOTU4105	0	0	0	0	0	0	0	0	0	0	0	0	0	0	0	0	1	0	0	0	0	0	0	0	0	0	0	0	0	0	Fungi; Ascomycota; Sordariomycetes; Incertae_sedis; Plectosphaerellaceae; Lectera; Lectera_longa
SH130640.06FU_EF641857_reps	0	0	0	0	0	0	0	0	0	0	0	0	0	0	0	0	0	0	0	0	0	0	0	1	0	0	0	0	0	0	Fungi; Ascomycota; Sordariomycetes; Incertae_sedis; Plectosphaerellaceae; Verticillium; Verticillium_biguttatum
New.ReferenceOTU5098	4	0	0	0	0	0	0	0	0	0	0	0	0	0	0	0	0	0	0	0	0	0	0	0	0	0	0	0	0	0	Fungi; Ascomycota; Sordariomycetes; Incertae_sedis; Plectosphaerellaceae; Verticillium; Verticillium_griseum
New.ReferenceOTU5931	0	0	0	0	0	0	0	0	0	0	0	0	0	0	0	0	0	0	1	0	0	0	0	0	0	0	0	0	0	0	Fungi; Ascomycota; Sordariomycetes; Incertae_sedis; Plectosphaerellaceae; Verticillium; Verticillium_griseum
New.ReferenceOTU5147	0	0	0	1	0	0	0	0	0	0	0	0	0	0	0	0	0	0	0	0	2	0	0	0	0	0	0	0	0	0	Fungi; Ascomycota; Sordariomycetes; Incertae_sedis; Plectosphaerellaceae; Verticillium; Verticillium_pseudohemipterigenum
New.ReferenceOTU1855	0	0	0	0	0	0	0	0	0	0	0	0	0	0	0	0	6	1	0	0	0	0	1	0	0	0	0	0	0	0	Fungi; Ascomycota; Sordariomycetes; Ophiostomatales; Ophiostomataceae; Ophiostoma; Ophiostoma_fumeum
New.ReferenceOTU6618	0	0	0	1	0	0	0	0	0	0	0	0	0	0	0	0	0	0	0	0	0	0	0	0	0	0	0	0	0	0	Fungi; Ascomycota; Sordariomycetes; Ophiostomatales; Ophiostomataceae; Ophiostoma; Ophiostoma_fumeum
New.ReferenceOTU6932	0	0	0	0	0	0	0	0	0	0	0	0	0	0	0	0	0	0	1	0	0	0	0	0	0	0	0	0	0	0	Fungi; Ascomycota; Sordariomycetes; Ophiostomatales; Ophiostomataceae; Ophiostoma; Ophiostoma_protea-sedis
New.ReferenceOTU4629	0	0	0	0	2	0	0	0	0	0	0	0	0	0	0	0	0	0	0	0	0	0	0	0	0	0	0	0	0	0	Fungi; Ascomycota; Sordariomycetes; Ophiostomatales; Ophiostomataceae; Ophiostoma; Ophiostoma_sp_C527
New.ReferenceOTU451	0	0	0	0	0	0	0	0	0	0	0	0	0	0	0	0	0	2	0	0	0	0	0	0	0	0	0	0	0	0	Fungi; Ascomycota; Sordariomycetes; Ophiostomatales; Ophiostomataceae; Sporothrix; Sporothrix_inflata
New.ReferenceOTU2427	3	0	0	0	0	0	0	0	0	0	1	0	0	1	2	1	0	0	0	4	0	0	0	1	0	0	1	3	0	0	Fungi; Ascomycota; Sordariomycetes; Ophiostomatales; Ophiostomataceae; Sporothrix; Sporothrix_schenckii
New.ReferenceOTU3599	1	26	0	0	0	0	1	0	0	0	1	0	0	0	0	0	1	2	0	2	2	0	1	7	0	0	0	0	2	0	Fungi; Ascomycota; Sordariomycetes; Ophiostomatales; Ophiostomataceae; Sporothrix; Sporothrix_schenckii
New.ReferenceOTU6444	1	0	0	0	0	0	0	0	0	0	0	0	0	0	1	0	0	0	0	0	0	0	0	0	0	0	0	0	2	0	Fungi; Ascomycota; Sordariomycetes; Ophiostomatales; Ophiostomataceae; Sporothrix; Sporothrix_schenckii
New.ReferenceOTU2057	0	0	0	0	0	2	0	0	0	0	0	0	0	0	0	0	0	0	0	0	0	0	0	0	0	0	0	0	0	0	Fungi; Ascomycota; Sordariomycetes; Ophiostomatales; Ophiostomataceae; Sporothrix; Sporothrix_sp
New.ReferenceOTU3457	0	0	0	0	0	0	0	0	0	0	0	0	0	0	0	0	0	0	1	0	0	0	0	0	0	0	0	0	0	0	Fungi; Ascomycota; Sordariomycetes; Ophiostomatales; Ophiostomataceae; Sporothrix; Sporothrix_sp
New.ReferenceOTU7471	0	0	0	0	0	0	0	0	0	0	0	0	0	0	0	0	0	0	4	0	0	0	0	0	0	0	0	3	0	0	Fungi; Ascomycota; Sordariomycetes; Ophiostomatales; Ophiostomataceae; Sporothrix; Sporothrix_sp
New.ReferenceOTU4640	0	0	5	0	0	0	1	0	0	0	0	0	0	0	0	0	0	0	0	0	0	0	0	0	0	0	0	1	0	0	Fungi; Ascomycota; Sordariomycetes; Ophiostomatales; Ophiostomataceae; Sporothrix; Sporothrix_sp_FMR_9336
New.ReferenceOTU747	0	0	0	0	0	0	0	0	0	0	1	0	0	0	0	0	1	0	0	0	0	0	0	0	0	0	0	0	0	0	Fungi; Ascomycota; Sordariomycetes; Ophiostomatales; Ophiostomataceae; Sporothrix; Sporothrix_sp_FMR_9336
New.ReferenceOTU3664	0	1	1	2	1	0	0	1	0	0	0	0	0	0	0	0	0	0	1	1	0	0	0	2	2	0	0	1	1	1	Fungi; Ascomycota; Sordariomycetes; Ophiostomatales; Ophiostomataceae; Sporothrix; Sporothrix_sp_KUC4053
New.ReferenceOTU3982	0	0	0	0	0	0	0	0	0	0	0	0	0	0	0	0	0	3	0	0	0	0	0	1	0	0	0	0	0	0	Fungi; Ascomycota; Sordariomycetes; Ophiostomatales; Ophiostomataceae; Sporothrix; Sporothrix_sp_KUC4053
New.ReferenceOTU6044	0	0	0	0	0	0	0	0	0	0	0	0	0	0	0	0	0	0	0	2	0	0	0	0	0	0	0	0	0	0	Fungi; Ascomycota; Sordariomycetes; Ophiostomatales; Ophiostomataceae; Sporothrix; Sporothrix_sp_KUC4053
New.ReferenceOTU6537	0	0	0	0	0	0	0	0	0	0	1	0	0	1	1	1	0	9	3	4	3	2	0	2	0	0	0	1	0	0	Fungi; Ascomycota; Sordariomycetes; Ophiostomatales; Ophiostomataceae; Sporothrix; Sporothrix_sp_KUC4053
New.ReferenceOTU6692	0	0	0	0	0	0	0	0	0	0	0	0	0	0	0	0	0	4	0	0	0	0	0	0	0	0	0	0	0	0	Fungi; Ascomycota; Sordariomycetes; Ophiostomatales; Ophiostomataceae; Sporothrix; Sporothrix_sp_KUC4053
New.ReferenceOTU8084	0	0	0	0	0	0	0	0	0	0	0	0	0	0	0	0	0	0	1	1	0	0	0	0	0	0	0	0	0	0	Fungi; Ascomycota; Sordariomycetes; Ophiostomatales; Ophiostomataceae; Sporothrix; Sporothrix_sp_KUC4053
New.CleanUp.ReferenceOTU656	0	0	0	0	0	1	0	0	0	0	0	0	0	0	0	0	0	0	0	0	0	1	0	0	0	0	0	0	0	0	Fungi; Ascomycota; Sordariomycetes; Xylariales; Amphisphaeriaceae; Adisciso; Adisciso_yakushimense
SH110095.06FU_AB594789_refs	0	0	0	0	0	0	0	0	0	0	0	0	0	0	0	0	0	0	0	0	0	1	0	0	0	0	0	0	0	0	Fungi; Ascomycota; Sordariomycetes; Xylariales; Amphisphaeriaceae; Adisciso; Adisciso_yakushimense
New.ReferenceOTU18	1	1	5	2	4	2	2	3	2	9	1	2	0	0	0	0	3	1	0	0	1	1	1	1	0	2	0	2	0	0	Fungi; Ascomycota; Sordariomycetes; Xylariales; Amphisphaeriaceae; Pestalotiopsis; Pestalotiopsis_foedans
SH005746.06FU_HE584979_reps_singleton	0	0	0	0	0	0	0	0	0	0	0	0	1	0	0	0	0	0	0	0	0	0	0	0	0	0	0	1	0	0	Fungi; Ascomycota; Sordariomycetes; Xylariales; Amphisphaeriaceae; Pestalotiopsis; Pestalotiopsis_foedans
SH120483.06FU_HQ107993_reps	0	0	0	0	2	0	0	1	0	1	2	2	1	2	0	1	0	0	0	0	0	0	0	0	0	0	0	2	0	2	Fungi; Ascomycota; Sordariomycetes; Xylariales; Amphisphaeriaceae; Pestalotiopsis; Pestalotiopsis_foedans
SH124198.06FU_JX398987_refs	0	2	1	2	2	0	0	2	1	4	7	4	1	7	2	0	3	0	0	3	2	0	1	3	0	1	0	2	3	1	Fungi; Ascomycota; Sordariomycetes; Xylariales; Amphisphaeriaceae; Pestalotiopsis; Pestalotiopsis_foedans
New.ReferenceOTU2721	1	0	0	0	0	0	0	5	0	0	0	0	0	0	0	0	0	0	0	0	0	0	0	0	0	0	0	0	0	2	Fungi; Ascomycota; Sordariomycetes; Xylariales; Amphisphaeriaceae; Pestalotiopsis; Pestalotiopsis_hangzhouensis
New.CleanUp.ReferenceOTU750	0	0	0	0	0	0	0	0	0	1	0	0	0	0	0	0	0	0	0	0	0	0	0	0	0	0	0	0	0	0	Fungi; Ascomycota; Sordariomycetes; Xylariales; Amphisphaeriaceae; Pestalotiopsis; Pestalotiopsis_microspora
New.ReferenceOTU7519	0	0	0	0	0	0	0	0	1	2	0	0	0	0	0	0	1	0	0	0	0	0	0	0	0	0	0	0	0	0	Fungi; Ascomycota; Sordariomycetes; Xylariales; Amphisphaeriaceae; Pestalotiopsis; Pestalotiopsis_olivacea
New.ReferenceOTU7309	0	0	0	0	0	0	0	0	1	0	0	0	0	0	0	0	0	0	0	0	2	0	0	0	4	0	0	0	0	0	Fungi; Ascomycota; Sordariomycetes; Xylariales; Amphisphaeriaceae; Pestalotiopsis; Pestalotiopsis_pallidotheae
SH110094.06FU_KC537804_refs	0	0	0	0	0	0	0	0	0	0	0	0	0	0	0	0	1	0	0	0	0	0	0	0	0	0	0	0	0	0	Fungi; Ascomycota; Sordariomycetes; Xylariales; Amphisphaeriaceae; Pestalotiopsis; Pestalotiopsis_rhododendri
SH110101.06FU_AY526870_reps	0	0	0	0	0	0	0	0	0	0	0	1	0	1	1	0	1	0	0	0	0	0	0	0	0	0	0	1	0	0	Fungi; Ascomycota; Sordariomycetes; Xylariales; Amphisphaeriaceae; Pestalotiopsis; Pestalotiopsis_rhododendri
SH110110.06FU_AF405295_reps	0	0	0	1	0	0	0	0	0	1	0	0	0	0	0	0	0	0	0	0	0	0	0	0	0	0	0	0	0	0	Fungi; Ascomycota; Sordariomycetes; Xylariales; Amphisphaeriaceae; Pestalotiopsis; Pestalotiopsis_rhododendri
New.ReferenceOTU514	3	0	0	0	0	0	0	1	0	0	0	0	0	0	0	0	1	0	0	0	0	0	0	0	0	0	0	0	0	0	Fungi; Ascomycota; Sordariomycetes; Xylariales; Amphisphaeriaceae; Pestalotiopsis; Pestalotiopsis_sp_FF_2011
SH023319.06FU_HE584965_reps_singleton	0	0	0	0	0	0	0	0	0	0	0	0	0	0	0	1	1	0	0	0	0	2	0	0	0	0	0	0	0	0	Fungi; Ascomycota; Sordariomycetes; Xylariales; Amphisphaeriaceae; Pestalotiopsis; Pestalotiopsis_theae
SH023320.06FU_HE584966_reps_singleton	0	0	0	0	0	0	0	0	0	1	0	0	0	0	0	0	1	0	0	0	1	0	0	0	0	0	0	0	0	1	Fungi; Ascomycota; Sordariomycetes; Xylariales; Amphisphaeriaceae; Pestalotiopsis; Pestalotiopsis_theae
SH124201.06FU_JQ683727_refs	9	1	0	3	4	2	2	3	1	4	10	4	2	1	1	1	4	0	0	2	2	4	0	1	2	0	0	7	0	2	Fungi; Ascomycota; Sordariomycetes; Xylariales; Amphisphaeriaceae; Pestalotiopsis; Pestalotiopsis_theae
SH124204.06FU_AB482200_reps	0	1	0	2	0	0	0	0	2	5	2	2	0	0	1	1	0	0	0	2	0	0	0	5	0	0	0	0	0	0	Fungi; Ascomycota; Sordariomycetes; Xylariales; Amphisphaeriaceae; Pestalotiopsis; Pestalotiopsis_theae
New.ReferenceOTU3659	0	0	0	0	0	0	0	0	0	0	0	0	0	0	0	0	0	0	1	0	0	0	0	0	0	0	0	0	0	0	Fungi; Ascomycota; Sordariomycetes; Xylariales; Diatrypaceae; Eutypella; Eutypella_vitis
New.ReferenceOTU3285	0	0	0	0	2	0	0	0	2	0	0	0	0	0	0	0	0	0	0	0	0	0	0	0	0	0	0	0	0	0	Fungi; Ascomycota; Sordariomycetes; Xylariales; Diatrypaceae; Libertella; Libertella_sp_EXP0557F
New.ReferenceOTU1454	0	0	0	0	0	0	0	0	1	0	0	0	0	0	0	0	0	0	0	0	0	0	0	0	0	0	0	0	0	0	Fungi; Ascomycota; Sordariomycetes; Xylariales; Incertae_sedis; Microdochium; Microdochium_sp
New.ReferenceOTU7000	0	0	0	0	0	0	0	0	3	0	0	0	0	0	0	0	0	0	0	0	0	0	0	0	0	0	0	0	0	0	Fungi; Ascomycota; Sordariomycetes; Xylariales; Incertae_sedis; Microdochium; Microdochium_sp
SH118137.06FU_JN601149_reps	0	0	0	0	0	0	0	0	0	0	0	0	0	0	0	0	0	0	0	0	0	3	0	0	0	0	0	0	0	0	Fungi; Ascomycota; Sordariomycetes; Xylariales; Incertae_sedis; Microdochium; Microdochium_sp_E9023a
New.ReferenceOTU6942	0	0	0	0	0	0	0	0	0	0	0	0	0	0	0	0	0	0	0	0	0	1	0	0	0	0	0	0	0	0	Fungi; Ascomycota; Sordariomycetes; Xylariales; Incertae_sedis; Monosporascus; Monosporascus_sp_28_YS_2010
New.ReferenceOTU7097	0	0	0	0	0	0	0	0	0	0	0	0	0	0	0	0	0	0	0	0	0	0	0	0	0	0	0	0	1	0	Fungi; Basidiomycota; Agaricomycetes; Corticiales; Corticiaceae; Laetisaria; Laetisaria_arvalis
New.ReferenceOTU2351	0	0	0	0	0	2	0	0	0	0	0	0	0	0	0	0	0	0	0	0	0	0	0	0	0	0	0	0	0	0	Fungi; Basidiomycota; Exobasidiomycetes; Microstromatales; Microstromataceae; Sympodiomycopsis; Sympodiomycopsis_sp_NYNU_121010
New.ReferenceOTU3045	0	0	0	0	0	1	0	0	0	0	0	0	0	0	0	0	0	0	0	0	0	0	0	0	0	0	0	0	0	0	Fungi; Basidiomycota; Exobasidiomycetes; Microstromatales; Microstromataceae; Sympodiomycopsis; Sympodiomycopsis_sp_NYNU_121010
New.ReferenceOTU6968	0	0	0	0	0	0	0	0	0	0	0	0	0	0	0	0	0	1	9	0	0	0	0	0	0	0	0	0	0	0	Fungi; Basidiomycota; Microbotryomycetes; Sporidiobolales; Incertae_sedis; Sporobolomyces; Sporobolomyces_linderae
New.ReferenceOTU871	0	0	0	2	0	0	0	0	0	0	0	0	0	0	0	0	0	0	0	5	0	0	0	0	0	0	0	0	0	0	Fungi; Basidiomycota; Microbotryomycetes; Sporidiobolales; Incertae_sedis; Sporobolomyces; Sporobolomyces_linderae
New.ReferenceOTU3728	0	0	0	0	0	0	0	0	0	0	0	0	0	0	0	0	0	0	0	0	0	0	0	0	0	0	0	2	0	0	Fungi; Basidiomycota; Microbotryomycetes; Sporidiobolales; Incertae_sedis; Sporobolomyces; Sporobolomyces_sp_TY_215
New.ReferenceOTU6262	0	0	0	0	0	0	0	0	0	0	0	0	0	0	0	0	0	0	0	0	0	0	0	0	0	0	2	0	0	0	Fungi; Basidiomycota; Tremellomycetes; Trichosporonales; Trichosporonaceae; Trichosporon; Trichosporon_asahii
SH091931.06FU_AF444439_refs	0	0	1	0	0	0	0	0	0	0	10	0	0	0	0	0	0	0	0	0	0	0	1	0	0	0	5	1	0	0	Fungi; Basidiomycota; Tremellomycetes; Trichosporonales; Trichosporonaceae; Trichosporon; Trichosporon_asahii
SH091934.06FU_AB031520_reps	0	0	0	0	0	0	0	0	0	0	1	0	0	0	0	0	0	0	0	0	0	0	0	0	0	0	1	0	0	0	Fungi; Basidiomycota; Tremellomycetes; Trichosporonales; Trichosporonaceae; Trichosporon; Trichosporon_asahii
New.ReferenceOTU1192	0	0	0	0	0	0	0	0	0	1	0	0	1	0	0	0	0	0	0	0	0	0	0	0	0	0	0	0	0	0	Fungi; Basidiomycota; Tremellomycetes; Trichosporonales; Trichosporonaceae; Trichosporon; Trichosporon_laibachii
New.ReferenceOTU5854	0	0	0	0	0	0	0	0	3	3	2	1	0	0	0	0	1	0	1	1	0	0	0	0	0	0	4	0	0	0	Fungi; Basidiomycota; Tremellomycetes; Trichosporonales; Trichosporonaceae; Trichosporon; Trichosporon_laibachii
SH120279.06FU_AF444440_refs	0	0	0	0	0	0	0	0	0	1	0	0	0	0	0	0	0	0	0	0	0	0	0	0	0	0	1	0	0	0	Fungi; Basidiomycota; Tremellomycetes; Trichosporonales; Trichosporonaceae; Trichosporon; Trichosporon_laibachii
SH120280.06FU_AF444421_refs	3	2	6	1	6	1	0	4	8	5	44	6	0	2	1	1	0	0	0	5	0	0	4	8	0	0	28	0	1	0	Fungi; Basidiomycota; Tremellomycetes; Trichosporonales; Trichosporonaceae; Trichosporon; Trichosporon_laibachii
SH095961.06FU_FJ528710_reps	0	0	0	0	0	0	0	1	0	0	0	0	0	0	0	0	0	0	0	0	0	0	8	0	0	0	0	0	0	0	Fungi; Basidiomycota; Tremellomycetes; Trichosporonales; Trichosporonaceae; Trichosporon; Trichosporon_sp
New.ReferenceOTU1034	1	0	1	1	1	0	0	0	2	1	0	0	0	0	0	0	0	1	5	3	0	0	1	0	0	0	0	0	0	0	Fungi; Basidiomycota; Tremellomycetes; Trichosporonales; Trichosporonaceae; Trichosporon; Trichosporon_sporotrichoides
New.ReferenceOTU4891	0	0	0	0	0	0	0	0	0	0	0	0	0	0	0	0	0	0	0	1	0	0	0	0	0	0	0	0	0	0	Fungi; Basidiomycota; Tremellomycetes; Trichosporonales; Trichosporonaceae; Trichosporon; Trichosporon_sporotrichoides
SH004071.06FU_HE975032_reps_singleton	0	0	0	0	0	0	0	0	0	0	0	0	0	0	0	0	0	1	0	0	0	0	0	0	0	0	0	0	0	0	Fungi; Basidiomycota; Tremellomycetes; Trichosporonales; Trichosporonaceae; Trichosporon; Trichosporon_sporotrichoides
SH091932.06FU_HM627166_reps	8	0	13	8	5	0	0	0	92	1	6	2	1	1	0	0	0	14	37	107	0	0	2	2	0	0	0	0	0	0	Fungi; Basidiomycota; Tremellomycetes; Trichosporonales; Trichosporonaceae; Trichosporon; Trichosporon_sporotrichoides
SH091940.06FU_NR_073261_reps	7	0	8	5	0	0	0	0	35	0	9	0	1	0	0	1	0	3	10	14	0	0	0	0	0	0	0	0	0	0	Fungi; Basidiomycota; Tremellomycetes; Trichosporonales; Trichosporonaceae; Trichosporon; Trichosporon_sporotrichoides
SH091935.06FU_JF680904_refs	0	0	0	0	0	0	0	0	0	0	0	0	0	0	0	0	0	0	0	0	0	0	0	0	0	0	0	0	2	0	Fungi; Basidiomycota; Tremellomycetes; Trichosporonales; Trichosporonaceae; Trichosporon; Trichosporon_vanderwaltii
New.ReferenceOTU387	0	0	0	0	0	0	0	0	0	1	0	0	0	0	0	0	0	0	0	0	0	0	0	0	0	0	0	0	0	0	Fungi; Chytridiomycota; Chytridiomycetes; Chytridiales; Chytridiaceae; Polyphlyctis; Polyphlyctis_unispina
New.ReferenceOTU1127	0	0	0	1	0	0	0	0	0	0	0	0	0	0	0	0	0	0	0	0	0	0	0	0	0	0	0	0	0	0	Fungi; Chytridiomycota; Chytridiomycetes; Rhizophydiales; Rhizophydiaceae; Rhizophydium; Rhizophydium_carpophilum
New.ReferenceOTU6193	0	0	0	0	0	0	0	0	0	0	2	0	0	0	0	0	0	0	0	0	0	0	0	0	0	0	0	0	0	0	Fungi; Chytridiomycota; Chytridiomycetes; Rhizophydiales; Rhizophydiaceae; Rhizophydium; Rhizophydium_carpophilum
New.ReferenceOTU1177	0	0	0	0	0	0	0	0	1	0	0	0	0	0	0	0	0	0	0	0	0	0	0	0	0	0	0	0	0	0	Fungi; Chytridiomycota; Chytridiomycetes; Rhizophydiales; Rhizophydiaceae; Rhizophydium; Rhizophydium_sp
New.ReferenceOTU275	0	0	0	0	0	0	1	0	0	0	0	0	0	0	0	0	0	0	0	0	0	0	0	0	0	0	0	0	0	0	Fungi; Chytridiomycota; Chytridiomycetes; Rhizophydiales; Rhizophydiaceae; Rhizophydium; Rhizophydium_sp_PL_104
New.ReferenceOTU6583	0	0	0	0	0	3	0	0	0	0	0	0	0	0	0	0	0	0	0	0	0	0	0	0	0	0	0	0	0	0	Fungi; Chytridiomycota; Chytridiomycetes; Rhizophydiales; Rhizophydiaceae; Rhizophydium; Rhizophydium_sp_PL_104
New.ReferenceOTU1325	0	0	0	6	6	0	0	0	0	2	0	0	0	0	1	1	0	0	0	0	1	0	0	0	0	0	0	0	1	0	Fungi; Chytridiomycota; Chytridiomycetes; Spizellomycetales; Spizellomycetaceae; Triparticalcar; Triparticalcar_sp_JEL_560
New.CleanUp.ReferenceOTU1931	0	0	0	0	0	0	0	0	0	0	0	0	0	0	0	0	0	0	0	0	0	1	0	0	0	0	0	0	0	0	Fungi; Ascomycota; Archaeorhizomycetes; Archaeorhizomycetales; Archaeorhizomycetaceae; Archaeorhizomyces; Archaeorhizomyces_sp
New.ReferenceOTU1116	0	0	0	0	0	0	0	0	0	1	0	0	0	0	0	0	0	0	0	0	0	0	0	0	0	0	0	0	0	0	Fungi; Ascomycota; Archaeorhizomycetes; Archaeorhizomycetales; Archaeorhizomycetaceae; Archaeorhizomyces; Archaeorhizomyces_sp
New.ReferenceOTU1244	0	0	0	0	0	0	0	0	0	0	0	0	0	0	1	0	0	0	0	0	0	0	0	0	0	0	0	0	0	0	Fungi; Ascomycota; Archaeorhizomycetes; Archaeorhizomycetales; Archaeorhizomycetaceae; Archaeorhizomyces; Archaeorhizomyces_sp
New.ReferenceOTU1484	0	0	0	0	0	0	0	0	0	0	0	0	0	0	0	0	0	0	0	0	0	0	6	0	0	0	1	0	0	0	Fungi; Ascomycota; Archaeorhizomycetes; Archaeorhizomycetales; Archaeorhizomycetaceae; Archaeorhizomyces; Archaeorhizomyces_sp
New.ReferenceOTU1913	0	0	0	0	0	0	0	0	0	0	0	0	0	0	0	4	1	0	0	0	0	0	0	1	0	0	0	0	0	0	Fungi; Ascomycota; Archaeorhizomycetes; Archaeorhizomycetales; Archaeorhizomycetaceae; Archaeorhizomyces; Archaeorhizomyces_sp
New.ReferenceOTU2176	0	0	0	0	0	0	0	0	0	0	0	0	0	0	1	0	0	0	0	0	0	0	0	0	0	0	0	0	0	0	Fungi; Ascomycota; Archaeorhizomycetes; Archaeorhizomycetales; Archaeorhizomycetaceae; Archaeorhizomyces; Archaeorhizomyces_sp
New.ReferenceOTU2249	0	0	0	0	0	0	0	0	0	0	0	0	0	1	0	0	0	0	0	0	0	0	0	0	0	0	0	0	0	0	Fungi; Ascomycota; Archaeorhizomycetes; Archaeorhizomycetales; Archaeorhizomycetaceae; Archaeorhizomyces; Archaeorhizomyces_sp
New.ReferenceOTU2300	0	0	0	0	0	0	0	0	0	0	0	0	0	0	1	0	0	0	0	0	0	0	0	0	0	0	0	0	0	0	Fungi; Ascomycota; Archaeorhizomycetes; Archaeorhizomycetales; Archaeorhizomycetaceae; Archaeorhizomyces; Archaeorhizomyces_sp
New.ReferenceOTU255	0	0	0	0	0	0	0	4	0	0	0	0	0	0	0	0	0	0	0	0	0	0	0	0	0	0	0	0	0	0	Fungi; Ascomycota; Archaeorhizomycetes; Archaeorhizomycetales; Archaeorhizomycetaceae; Archaeorhizomyces; Archaeorhizomyces_sp
New.ReferenceOTU2655	0	0	0	0	1	0	0	0	0	0	0	0	0	0	0	0	0	0	0	0	0	0	0	0	0	0	0	0	0	0	Fungi; Ascomycota; Archaeorhizomycetes; Archaeorhizomycetales; Archaeorhizomycetaceae; Archaeorhizomyces; Archaeorhizomyces_sp
New.ReferenceOTU2756	0	0	0	0	0	0	0	0	0	0	0	0	0	1	0	6	0	0	0	0	0	0	0	0	0	0	0	0	0	0	Fungi; Ascomycota; Archaeorhizomycetes; Archaeorhizomycetales; Archaeorhizomycetaceae; Archaeorhizomyces; Archaeorhizomyces_sp
New.ReferenceOTU3355	0	0	0	0	0	0	0	8	0	0	0	0	0	0	0	0	0	0	0	0	0	0	0	0	0	0	0	0	0	0	Fungi; Ascomycota; Archaeorhizomycetes; Archaeorhizomycetales; Archaeorhizomycetaceae; Archaeorhizomyces; Archaeorhizomyces_sp
New.ReferenceOTU3445	0	0	0	0	0	0	0	0	0	0	0	0	0	0	0	0	0	0	1	0	0	0	0	0	0	0	0	0	0	0	Fungi; Ascomycota; Archaeorhizomycetes; Archaeorhizomycetales; Archaeorhizomycetaceae; Archaeorhizomyces; Archaeorhizomyces_sp
New.ReferenceOTU3488	0	0	0	0	0	0	0	0	0	0	0	0	0	0	0	0	1	0	0	0	0	0	0	0	0	0	0	0	0	0	Fungi; Ascomycota; Archaeorhizomycetes; Archaeorhizomycetales; Archaeorhizomycetaceae; Archaeorhizomyces; Archaeorhizomyces_sp
New.ReferenceOTU3558	0	0	0	0	0	0	0	0	0	0	0	0	1	0	0	0	0	0	0	0	0	0	0	0	0	0	0	0	0	0	Fungi; Ascomycota; Archaeorhizomycetes; Archaeorhizomycetales; Archaeorhizomycetaceae; Archaeorhizomyces; Archaeorhizomyces_sp
New.ReferenceOTU3730	0	0	0	0	0	0	0	0	0	0	0	0	0	1	0	0	0	0	0	0	0	0	0	0	0	0	0	0	0	0	Fungi; Ascomycota; Archaeorhizomycetes; Archaeorhizomycetales; Archaeorhizomycetaceae; Archaeorhizomyces; Archaeorhizomyces_sp
New.ReferenceOTU3853	0	1	0	0	0	0	0	0	0	0	0	0	0	0	0	0	0	0	0	0	0	0	0	0	0	0	0	0	0	1	Fungi; Ascomycota; Archaeorhizomycetes; Archaeorhizomycetales; Archaeorhizomycetaceae; Archaeorhizomyces; Archaeorhizomyces_sp
New.ReferenceOTU3880	1	0	0	0	0	0	1	0	0	0	0	0	0	0	0	0	0	0	0	0	0	0	0	0	0	0	0	0	0	0	Fungi; Ascomycota; Archaeorhizomycetes; Archaeorhizomycetales; Archaeorhizomycetaceae; Archaeorhizomyces; Archaeorhizomyces_sp
New.ReferenceOTU4681	0	0	0	0	0	0	1	0	0	0	0	0	0	0	0	0	0	0	0	0	0	0	0	0	0	0	0	0	0	0	Fungi; Ascomycota; Archaeorhizomycetes; Archaeorhizomycetales; Archaeorhizomycetaceae; Archaeorhizomyces; Archaeorhizomyces_sp
New.ReferenceOTU472	0	0	0	0	0	0	0	0	0	0	1	0	2	0	0	0	0	0	0	0	0	0	0	0	0	0	0	0	0	0	Fungi; Ascomycota; Archaeorhizomycetes; Archaeorhizomycetales; Archaeorhizomycetaceae; Archaeorhizomyces; Archaeorhizomyces_sp
New.ReferenceOTU4834	0	0	0	0	0	0	0	0	0	0	0	0	0	0	0	0	0	0	0	0	0	0	0	0	0	0	0	0	0	1	Fungi; Ascomycota; Archaeorhizomycetes; Archaeorhizomycetales; Archaeorhizomycetaceae; Archaeorhizomyces; Archaeorhizomyces_sp
New.ReferenceOTU5309	0	0	0	0	0	0	0	0	0	0	0	0	0	0	0	0	0	0	0	0	1	0	0	0	0	0	0	0	0	0	Fungi; Ascomycota; Archaeorhizomycetes; Archaeorhizomycetales; Archaeorhizomycetaceae; Archaeorhizomyces; Archaeorhizomyces_sp

New.ReferenceOTU6149	0	0	0	0	0	0	0	0	0	0	0	0	0	0	0	0	0	0	0	0	0	0	0	0	0	0	0	0	1	0	Fungi; Ascomycota; Archaeorhizomycetes; Archaeorhizomycetales; Archaeorhizomycetaceae; Archaeorhizomyces; Archaeorhizomyces_sp
New.ReferenceOTU7292	0	0	0	0	0	0	0	0	0	0	0	0	0	3	0	0	0	0	0	0	0	0	0	0	0	0	0	0	0	0	Fungi; Ascomycota; Archaeorhizomycetes; Archaeorhizomycetales; Archaeorhizomycetaceae; Archaeorhizomyces; Archaeorhizomyces_sp
New.ReferenceOTU7414	4	0	0	0	0	0	0	0	0	0	0	0	0	0	0	0	0	0	0	0	0	0	0	0	0	0	0	0	0	0	Fungi; Ascomycota; Archaeorhizomycetes; Archaeorhizomycetales; Archaeorhizomycetaceae; Archaeorhizomyces; Archaeorhizomyces_sp
New.ReferenceOTU7433	1	0	0	0	0	0	0	0	0	0	0	0	0	0	0	0	0	0	0	0	0	0	0	0	0	0	0	0	0	0	Fungi; Ascomycota; Archaeorhizomycetes; Archaeorhizomycetales; Archaeorhizomycetaceae; Archaeorhizomyces; Archaeorhizomyces_sp
New.ReferenceOTU7837	0	0	0	0	0	0	0	0	0	0	0	0	1	0	0	0	0	0	0	0	0	0	0	0	0	0	0	0	0	0	Fungi; Ascomycota; Archaeorhizomycetes; Archaeorhizomycetales; Archaeorhizomycetaceae; Archaeorhizomyces; Archaeorhizomyces_sp
New.ReferenceOTU903	2	0	0	0	2	0	0	0	0	0	0	0	0	1	1	0	0	0	0	0	0	0	0	1	0	0	0	0	0	0	Fungi; Ascomycota; Archaeorhizomycetes; Archaeorhizomycetales; Archaeorhizomycetaceae; Archaeorhizomyces; Archaeorhizomyces_sp
SH094172.06FU_GQ160037_reps	0	0	0	0	0	0	0	7	0	0	1	0	0	0	0	0	0	0	0	0	0	0	0	0	0	0	0	0	0	0	Fungi; Ascomycota; Archaeorhizomycetes; Archaeorhizomycetales; Archaeorhizomycetaceae; Archaeorhizomyces; Archaeorhizomyces_sp
New.ReferenceOTU1494	0	0	0	1	0	0	0	0	0	0	0	0	0	0	0	0	0	0	0	0	0	0	0	0	0	0	0	0	0	0	Fungi; Ascomycota; Archaeorhizomycetes; unidentified; unidentified; unidentified; Archaeorhizomycetes_sp
New.ReferenceOTU1621	0	0	0	0	0	0	0	0	0	0	0	0	0	0	0	0	1	0	0	0	0	0	0	0	0	0	0	0	0	0	Fungi; Ascomycota; Archaeorhizomycetes; unidentified; unidentified; unidentified; Archaeorhizomycetes_sp
New.ReferenceOTU3626	2	0	0	0	0	0	0	0	0	0	0	0	0	0	0	0	0	0	0	0	0	0	0	0	0	0	0	0	0	0	Fungi; Ascomycota; Archaeorhizomycetes; unidentified; unidentified; unidentified; Archaeorhizomycetes_sp
New.ReferenceOTU4215	0	0	0	0	0	0	0	0	0	0	1	0	0	0	0	0	1	0	0	0	0	0	0	0	0	0	1	0	0	0	Fungi; Ascomycota; Archaeorhizomycetes; unidentified; unidentified; unidentified; Archaeorhizomycetes_sp
New.ReferenceOTU4570	0	0	0	0	1	0	0	0	0	0	0	0	0	0	0	0	0	0	0	0	0	0	0	0	0	1	0	0	0	0	Fungi; Ascomycota; Archaeorhizomycetes; unidentified; unidentified; unidentified; Archaeorhizomycetes_sp
New.ReferenceOTU5568	0	0	0	0	0	0	0	0	0	0	0	0	1	0	0	0	0	0	0	0	0	0	0	0	0	0	0	0	0	0	Fungi; Ascomycota; Archaeorhizomycetes; unidentified; unidentified; unidentified; Archaeorhizomycetes_sp
New.ReferenceOTU65	0	0	0	0	0	0	0	0	0	0	0	0	0	0	0	1	0	0	0	0	0	0	0	0	0	0	0	0	0	0	Fungi; Ascomycota; Archaeorhizomycetes; unidentified; unidentified; unidentified; Archaeorhizomycetes_sp
New.ReferenceOTU6909	0	0	0	0	0	0	0	0	0	0	0	0	0	0	0	0	0	9	0	0	0	0	0	0	0	0	0	0	0	0	Fungi; Ascomycota; Archaeorhizomycetes; unidentified; unidentified; unidentified; Archaeorhizomycetes_sp
New.ReferenceOTU7776	0	0	0	0	0	0	0	0	0	0	0	0	0	0	0	0	0	5	0	0	0	0	0	0	0	0	0	0	0	0	Fungi; Ascomycota; Archaeorhizomycetes; unidentified; unidentified; unidentified; Archaeorhizomycetes_sp
New.ReferenceOTU8076	0	0	0	0	0	3	0	0	0	0	0	0	1	0	0	0	0	0	0	0	0	0	0	0	0	0	0	0	0	0	Fungi; Ascomycota; Archaeorhizomycetes; unidentified; unidentified; unidentified; Archaeorhizomycetes_sp
New.ReferenceOTU909	0	0	0	0	0	0	0	0	0	0	0	0	0	0	0	0	0	4	0	0	0	0	0	0	0	0	0	0	0	0	Fungi; Ascomycota; Archaeorhizomycetes; unidentified; unidentified; unidentified; Archaeorhizomycetes_sp
New.ReferenceOTU7790	0	1	0	0	0	0	0	0	0	0	0	0	0	0	0	0	0	0	0	0	0	0	0	0	0	0	0	0	0	0	Fungi; Ascomycota; Dothideomycetes; Botryosphaeriales; unidentified; unidentified; Botryosphaeriales_sp
New.ReferenceOTU7125	0	0	0	0	0	0	0	1	0	0	0	0	0	0	0	0	0	0	0	0	0	0	0	0	0	0	0	0	0	0	Fungi; Ascomycota; Dothideomycetes; Capnodiales; Mycosphaerellaceae; unidentified; Mycosphaerellaceae_sp_SD_01
New.ReferenceOTU1231	0	0	0	0	0	0	0	0	0	0	0	0	0	0	0	0	1	0	0	0	0	0	0	0	0	0	0	0	0	0	Fungi; Ascomycota; Dothideomycetes; Capnodiales; Teratosphaeriaceae; unidentified; Teratosphaeriaceae_sp
New.ReferenceOTU1599	0	0	0	0	0	0	0	0	0	0	0	0	0	0	0	0	0	0	1	0	0	0	0	0	0	0	0	0	0	0	Fungi; Ascomycota; Dothideomycetes; Capnodiales; Teratosphaeriaceae; unidentified; Teratosphaeriaceae_sp
New.ReferenceOTU1776	0	0	0	0	0	0	0	1	0	0	0	0	0	0	0	0	0	0	0	0	0	0	0	0	0	0	0	0	0	0	Fungi; Ascomycota; Dothideomycetes; Capnodiales; unidentified; unidentified; Capnodiales_sp
New.ReferenceOTU1915	0	0	0	0	0	0	0	0	4	0	0	1	0	0	0	0	0	1	0	0	0	0	0	2	0	0	0	0	0	1	Fungi; Ascomycota; Dothideomycetes; Capnodiales; unidentified; unidentified; Capnodiales_sp
New.ReferenceOTU2507	0	0	0	0	0	0	0	0	0	0	0	0	2	0	8	0	0	0	4	0	0	0	0	0	0	0	0	0	0	0	Fungi; Ascomycota; Dothideomycetes; Capnodiales; unidentified; unidentified; Capnodiales_sp
New.ReferenceOTU2653	0	0	0	0	0	0	0	0	0	0	0	0	0	0	0	0	0	0	0	0	0	0	0	0	0	0	4	0	0	0	Fungi; Ascomycota; Dothideomycetes; Capnodiales; unidentified; unidentified; Capnodiales_sp
New.ReferenceOTU3083	0	0	1	0	0	0	0	0	0	0	0	0	0	0	0	0	0	0	0	0	0	0	1	0	0	0	0	0	0	0	Fungi; Ascomycota; Dothideomycetes; Capnodiales; unidentified; unidentified; Capnodiales_sp
New.ReferenceOTU3454	0	0	0	0	0	0	0	0	0	0	0	0	0	0	0	0	0	0	0	0	0	0	0	0	0	0	0	0	0	2	Fungi; Ascomycota; Dothideomycetes; Capnodiales; unidentified; unidentified; Capnodiales_sp
New.ReferenceOTU4133	0	0	0	1	0	0	0	0	0	0	0	0	0	0	0	0	0	0	0	0	0	0	0	0	0	0	0	0	0	1	Fungi; Ascomycota; Dothideomycetes; Capnodiales; unidentified; unidentified; Capnodiales_sp
New.ReferenceOTU444	0	0	0	0	0	0	0	0	0	0	0	0	0	0	0	0	0	0	0	0	0	0	0	0	0	0	0	0	1	0	Fungi; Ascomycota; Dothideomycetes; Capnodiales; unidentified; unidentified; Capnodiales_sp
New.ReferenceOTU5678	0	0	0	0	0	0	0	0	0	0	0	0	0	1	0	0	0	0	0	0	0	0	0	0	0	0	0	0	0	0	Fungi; Ascomycota; Dothideomycetes; Capnodiales; unidentified; unidentified; Capnodiales_sp
New.ReferenceOTU5877	0	0	0	2	0	0	0	0	0	0	0	0	0	0	0	0	0	0	0	0	0	0	0	0	0	0	0	0	0	0	Fungi; Ascomycota; Dothideomycetes; Capnodiales; unidentified; unidentified; Capnodiales_sp
New.ReferenceOTU6412	0	0	1	0	0	0	0	0	0	0	0	0	0	0	0	0	0	0	0	0	0	0	0	0	0	0	0	0	0	0	Fungi; Ascomycota; Dothideomycetes; Capnodiales; unidentified; unidentified; Capnodiales_sp
New.ReferenceOTU6846	0	0	0	1	0	0	0	0	0	0	0	0	0	0	0	0	0	0	0	0	0	0	0	0	0	0	0	0	0	0	Fungi; Ascomycota; Dothideomycetes; Capnodiales; unidentified; unidentified; Capnodiales_sp
New.ReferenceOTU7016	0	0	0	0	2	0	0	0	0	0	0	0	0	0	0	0	0	1	0	0	0	0	0	0	0	2	0	2	0	0	Fungi; Ascomycota; Dothideomycetes; Capnodiales; unidentified; unidentified; Capnodiales_sp
New.ReferenceOTU7021	0	0	0	0	0	0	0	0	0	0	0	0	0	2	0	0	0	0	0	0	0	0	0	0	0	0	0	0	0	0	Fungi; Ascomycota; Dothideomycetes; Capnodiales; unidentified; unidentified; Capnodiales_sp
New.ReferenceOTU7046	0	0	1	2	0	0	0	1	0	0	0	1	0	0	0	0	0	0	0	0	0	0	0	0	0	0	0	0	0	0	Fungi; Ascomycota; Dothideomycetes; Capnodiales; unidentified; unidentified; Capnodiales_sp
New.ReferenceOTU7089	0	0	0	0	0	0	1	0	0	0	0	0	0	0	0	0	0	0	0	0	0	0	0	0	0	0	0	0	0	0	Fungi; Ascomycota; Dothideomycetes; Capnodiales; unidentified; unidentified; Capnodiales_sp
New.ReferenceOTU760	0	0	0	0	1	0	0	0	0	0	0	0	0	0	0	0	0	0	0	0	0	0	0	0	0	0	0	0	0	0	Fungi; Ascomycota; Dothideomycetes; Capnodiales; unidentified; unidentified; Capnodiales_sp
New.ReferenceOTU7945	0	0	0	0	0	0	0	0	0	0	0	0	0	0	0	0	0	0	0	0	0	0	0	0	0	0	0	0	0	1	Fungi; Ascomycota; Dothideomycetes; Capnodiales; unidentified; unidentified; Capnodiales_sp
SH092960.06FU_HQ634615_reps	0	0	0	0	0	0	0	0	0	0	3	0	0	0	0	0	0	1	0	0	0	0	0	0	0	0	0	0	0	2	Fungi; Ascomycota; Dothideomycetes; Capnodiales; unidentified; unidentified; Capnodiales_sp
SH004722.06FU_HQ634623_reps_singleton	0	0	0	0	0	0	0	0	0	0	0	0	0	1	0	0	0	0	0	0	0	0	0	0	0	0	0	0	0	0	Fungi; Ascomycota; Dothideomycetes; Capnodiales; unidentified; unidentified; Capnodiales_sp_M_Camp1
New.ReferenceOTU240	0	0	0	0	0	2	0	0	0	0	0	0	0	0	0	0	0	0	0	0	0	0	0	0	0	0	0	0	0	0	Fungi; Ascomycota; Dothideomycetes; Capnodiales; unidentified; unidentified; Capnodiales_sp_np0805F
New.ReferenceOTU4626	0	0	0	0	0	0	1	0	2	0	0	2	0	3	0	0	0	0	0	0	0	0	0	0	0	0	0	0	0	0	Fungi; Ascomycota; Dothideomycetes; Capnodiales; unidentified; unidentified; Capnodiales_sp_np0805F
New.ReferenceOTU197	0	0	0	0	0	0	0	0	0	0	0	0	0	0	3	0	0	0	0	0	0	0	0	0	0	0	0	0	0	0	Fungi; Ascomycota; Dothideomycetes; Capnodiales; unidentified; unidentified; uncultured_Capnodiales
New.ReferenceOTU2274	0	0	0	0	0	0	0	0	0	0	0	1	0	0	0	0	0	0	0	0	0	0	0	0	0	0	0	0	0	0	Fungi; Ascomycota; Dothideomycetes; Capnodiales; unidentified; unidentified; uncultured_Capnodiales
New.ReferenceOTU5908	0	0	0	0	0	0	0	0	0	0	0	0	0	0	0	0	0	0	1	0	0	0	0	0	0	0	0	0	0	0	Fungi; Ascomycota; Dothideomycetes; Capnodiales; unidentified; unidentified; uncultured_Capnodiales
New.ReferenceOTU7732	0	0	0	0	0	0	0	0	0	0	0	0	1	0	0	0	0	0	0	0	0	0	0	0	0	0	0	0	0	0	Fungi; Ascomycota; Dothideomycetes; Capnodiales; unidentified; unidentified; uncultured_Capnodiales
New.ReferenceOTU777	0	0	0	0	0	0	0	3	0	0	0	0	0	0	0	0	0	0	0	0	0	0	0	0	0	0	0	0	0	0	Fungi; Ascomycota; Dothideomycetes; Capnodiales; unidentified; unidentified; uncultured_Capnodiales
New.ReferenceOTU7785	0	0	0	0	0	0	1	0	0	0	0	0	0	0	3	0	3	0	0	0	0	0	1	0	0	2	0	0	0	1	Fungi; Ascomycota; Dothideomycetes; Capnodiales; unidentified; unidentified; uncultured_Capnodiales
New.ReferenceOTU5505	0	0	0	0	1	0	0	0	0	0	0	0	0	0	0	0	0	0	0	0	0	0	0	0	0	0	0	0	0	0	Fungi; Ascomycota; Dothideomycetes; Dothideales; unidentified; unidentified; unidentified_dothidealean_sp_MS316
New.ReferenceOTU447	0	0	0	1	0	0	0	0	0	0	0	0	0	0	0	0	0	0	0	0	0	0	0	0	0	0	0	0	0	0	Fungi; Ascomycota; Dothideomycetes; Hysteriales; unidentified; unidentified; Hysteriales_sp
New.ReferenceOTU585	0	0	0	0	0	0	0	0	0	0	0	0	1	0	0	0	0	0	0	0	0	0	0	0	0	0	0	0	0	0	Fungi; Ascomycota; Dothideomycetes; Hysteriales; unidentified; unidentified; Hysteriales_sp
New.ReferenceOTU1005	0	0	0	0	0	0	0	0	0	0	0	0	0	0	0	0	0	0	0	0	0	0	0	0	0	0	0	0	2	0	Fungi; Ascomycota; Dothideomycetes; Incertae_sedis; Myxotrichaceae; unidentified; Myxotrichaceae_sp
New.ReferenceOTU1156	2	0	0	0	0	0	0	0	0	0	0	0	0	0	0	0	0	0	0	0	0	0	0	0	0	0	0	0	0	0	Fungi; Ascomycota; Dothideomycetes; Incertae_sedis; Myxotrichaceae; unidentified; Myxotrichaceae_sp
New.ReferenceOTU1872	0	4	0	0	0	0	0	0	0	0	0	0	0	0	0	0	0	0	0	0	0	0	0	0	0	0	0	0	1	0	Fungi; Ascomycota; Dothideomycetes; Incertae_sedis; Myxotrichaceae; unidentified; Myxotrichaceae_sp
New.ReferenceOTU283	0	0	0	0	0	0	0	0	0	0	0	1	0	0	0	0	0	0	0	0	0	0	0	0	0	0	0	0	0	0	Fungi; Ascomycota; Dothideomycetes; Incertae_sedis; Myxotrichaceae; unidentified; Myxotrichaceae_sp
New.ReferenceOTU3811	0	0	0	0	0	0	0	0	0	0	0	0	0	0	0	0	0	0	0	0	0	0	0	0	0	0	0	0	1	0	Fungi; Ascomycota; Dothideomycetes; Incertae_sedis; Myxotrichaceae; unidentified; Myxotrichaceae_sp
New.ReferenceOTU461	0	8	0	3	0	0	0	0	0	0	0	0	0	0	0	0	0	0	0	0	0	0	0	0	0	21	0	0	1	0	Fungi; Ascomycota; Dothideomycetes; Incertae_sedis; Myxotrichaceae; unidentified; Myxotrichaceae_sp
New.ReferenceOTU4965	0	1	0	2	5	0	2	0	0	1	1	0	0	0	0	0	0	0	0	0	0	0	0	0	0	0	0	0	0	0	Fungi; Ascomycota; Dothideomycetes; Incertae_sedis; Myxotrichaceae; unidentified; Myxotrichaceae_sp
New.ReferenceOTU5063	0	0	0	0	7	0	0	0	0	1	0	0	0	0	0	0	0	0	0	0	0	0	0	0	0	0	0	0	0	0	Fungi; Ascomycota; Dothideomycetes; Incertae_sedis; Myxotrichaceae; unidentified; Myxotrichaceae_sp
New.ReferenceOTU546	0	1	5	0	0	0	1	0	0	1	0	1	0	0	0	0	0	0	1	0	0	0	1	2	0	0	0	0	1	0	Fungi; Ascomycota; Dothideomycetes; Incertae_sedis; Myxotrichaceae; unidentified; Myxotrichaceae_sp
New.ReferenceOTU5495	0	0	0	0	0	0	0	0	0	1	0	0	0	0	0	0	0	0	0	0	0	0	0	0	0	0	0	0	0	0	Fungi; Ascomycota; Dothideomycetes; Incertae_sedis; Myxotrichaceae; unidentified; Myxotrichaceae_sp
New.ReferenceOTU7060	0	0	0	0	0	0	0	0	0	0	0	0	0	0	0	0	0	0	0	0	0	0	0	0	0	0	0	0	1	0	Fungi; Ascomycota; Dothideomycetes; Incertae_sedis; Myxotrichaceae; unidentified; Myxotrichaceae_sp
New.ReferenceOTU7710	0	0	0	0	0	0	0	0	0	0	0	0	0	0	0	0	0	0	0	0	0	0	0	0	0	0	0	0	2	0	Fungi; Ascomycota; Dothideomycetes; Incertae_sedis; Myxotrichaceae; unidentified; Myxotrichaceae_sp
New.ReferenceOTU493	0	6	0	0	0	0	0	0	0	0	0	0	0	0	0	0	0	0	0	0	0	0	0	0	0	0	0	0	0	0	Fungi; Ascomycota; Dothideomycetes; Incertae_sedis; Myxotrichaceae; unidentified; Myxotrichaceae_sp_BESC246d
New.ReferenceOTU5237	0	4	0	0	0	0	0	0	0	0	0	0	0	0	0	0	0	0	0	0	0	0	0	0	0	0	0	0	0	0	Fungi; Ascomycota; Dothideomycetes; Incertae_sedis; Myxotrichaceae; unidentified; Myxotrichaceae_sp_BESC246d
New.ReferenceOTU2124	0	0	0	0	0	0	0	0	0	0	1	0	0	0	0	0	0	0	0	0	1	1	4	0	0	0	0	0	0	0	Fungi; Ascomycota; Dothideomycetes; Pleosporales; Arthopyreniaceae; unidentified; Arthopyreniaceae_sp
SH011454.06FU_EU686797_reps_singleton	0	0	0	0	0	0	0	0	0	0	0	0	0	0	0	0	0	0	0	0	1	0	6	0	0	0	0	0	0	0	Fungi; Ascomycota; Dothideomycetes; Pleosporales; Arthopyreniaceae; unidentified; Arthopyreniaceae_sp
New.ReferenceOTU4213	0	0	0	0	0	0	0	0	0	0	0	0	0	0	0	0	0	0	0	0	0	0	3	0	0	0	0	3	0	0	Fungi; Ascomycota; Dothideomycetes; Pleosporales; Incertae_sedis; Berkleasmium; Berkleasmium_sp
New.ReferenceOTU674	0	0	0	0	0	0	0	0	0	0	0	0	0	0	0	0	0	0	0	0	0	1	0	0	2	0	3	0	0	0	Fungi; Ascomycota; Dothideomycetes; Pleosporales; Incertae_sedis; Berkleasmium; Berkleasmium_sp
SH140251.06FU_DQ280263_reps	0	0	0	0	0	0	0	0	0	0	0	0	0	0	0	0	0	0	0	0	0	0	0	0	0	0	1	0	0	8	Fungi; Ascomycota; Dothideomycetes; Pleosporales; Incertae_sedis; Berkleasmium; Berkleasmium_sp_BCC_17003
New.ReferenceOTU3866	1	0	1	0	0	0	0	3	0	0	0	0	7	2	0	1	1	0	0	0	1	1	3	0	0	0	0	0	0	0	Fungi; Ascomycota; Dothideomycetes; Pleosporales; Incertae_sedis; Letendraea; Letendraea_helminthicola
New.ReferenceOTU7551	0	0	0	0	0	4	1	0	0	1	0	1	4	1	1	0	0	0	0	0	0	0	0	0	0	0	0	2	0	0	Fungi; Ascomycota; Dothideomycetes; Pleosporales; Incertae_sedis; Letendraea; Letendraea_helminthicola
SH119967.06FU_JQ026217_reps	0	0	2	0	0	0	3	0	0	0	1	2	5	1	0	0	1	0	0	0	0	0	0	0	0	0	0	0	0	0	Fungi; Ascomycota; Dothideomycetes; Pleosporales; Incertae_sedis; Letendraea; Letendraea_helminthicola
New.ReferenceOTU5700	1	0	0	0	0	3	0	0	0	0	0	0	2	0	0	0	0	0	0	0	0	0	0	0	0	0	1	0	0	0	Fungi; Ascomycota; Dothideomycetes; Pleosporales; Incertae_sedis; Letendraea; Letendraea_sp
SH119968.06FU_GQ999180_reps	1	0	0	0	0	0	0	0	0	0	0	0	0	0	0	0	0	0	0	0	0	0	0	0	0	0	0	0	0	0	Fungi; Ascomycota; Dothideomycetes; Pleosporales; Incertae_sedis; Letendraea; Letendraea_sp
New.ReferenceOTU4593	0	0	0	0	0	0	0	0	0	0	0	0	0	0	1	0	0	0	0	0	5	0	0	0	0	0	1	0	0	0	Fungi; Ascomycota; Dothideomycetes; Pleosporales; Incertae_sedis; unidentified; Pleosporales_sp
New.ReferenceOTU167	0	0	0	0	0	0	0	0	0	0	0	0	0	0	0	0	0	0	1	0	0	0	0	0	0	0	0	0	0	0	Fungi; Ascomycota; Dothideomycetes; Pleosporales; Montagnulaceae; unidentified; Montagnulaceae_sp
New.ReferenceOTU2297	0	0	0	0	0	0	0	0	0	0	0	0	0	0	0	0	0	0	0	0	0	0	0	0	0	0	0	0	0	3	Fungi; Ascomycota; Dothideomycetes; Pleosporales; Montagnulaceae; unidentified; Montagnulaceae_sp
SH094617.06FU_FJ037726_reps	0	0	0	0	0	0	0	0	0	0	0	0	1	0	0	0	0	0	0	0	0	8	0	0	0	0	0	0	0	5	Fungi; Ascomycota; Dothideomycetes; Pleosporales; Montagnulaceae; unidentified; Montagnulaceae_sp
SH126643.06FU_EU917125_reps	0	0	0	0	0	0	0	0	0	0	0	0	0	0	0	0	0	0	0	10	0	0	1	0	1	0	0	0	0	0	Fungi; Ascomycota; Dothideomycetes; Pleosporales; Montagnulaceae; unidentified; Montagnulaceae_sp
SH113811.06FU_HE585000_reps	0	0	0	0	0	0	0	0	0	0	0	0	0	0	0	0	0	0	0	0	0	2	0	0	0	0	0	0	0	0	Fungi; Ascomycota; Dothideomycetes; Pleosporales; Phaeosphaeriaceae; unidentified; Phaeosphaeriaceae_sp
New.ReferenceOTU4519	0	0	0	0	0	0	0	0	0	0	0	0	4	0	0	0	0	0	0	0	0	0	0	0	0	0	0	0	0	0	Fungi; Ascomycota; Dothideomycetes; Pleosporales; Pleosporaceae; unidentified; Pleosporaceae_sp
SH127401.06FU_JX868648_reps	0	0	0	0	0	0	0	0	0	0	0	0	6	0	0	0	0	0	0	0	0	0	0	0	0	0	0	0	0	0	Fungi; Ascomycota; Dothideomycetes; Pleosporales; Pleosporaceae; unidentified; Pleosporaceae_sp
SH127402.06FU_JN006771_reps	0	0	2	0	0	0	0	0	0	0	0	0	0	0	0	0	0	0	0	0	0	0	0	0	0	0	0	0	0	0	Fungi; Ascomycota; Dothideomycetes; Pleosporales; Pleosporaceae; unidentified; Pleosporaceae_sp
SH127407.06FU_JX966628_reps	0	0	0	0	0	0	0	0	0	0	0	0	0	0	1	0	0	0	0	0	0	0	0	0	0	0	0	0	0	0	Fungi; Ascomycota; Dothideomycetes; Pleosporales; Pleosporaceae; unidentified; Pleosporaceae_sp
SH127420.06FU_EF452444_reps	0	0	0	0	0	0	0	1	0	0	0	0	0	0	0	0	0	0	0	0	0	0	0	0	0	0	0	0	0	1	Fungi; Ascomycota; Dothideomycetes; Pleosporales; Pleosporaceae; unidentified; Pleosporaceae_sp
New.ReferenceOTU5588	0	0	0	0	0	0	0	0	0	0	0	0	0	0	0	0	0	0	0	0	0	0	2	0	0	0	0	0	0	0	Fungi; Ascomycota; Dothideomycetes; Pleosporales; Pleosporaceae; unidentified; uncultured_Cochliobolus
New.ReferenceOTU410	0	0	0	0	0	0	0	0	0	0	0	0	0	0	0	0	0	2	1	0	0	0	0	0	0	0	0	0	0	0	Fungi; Ascomycota; Dothideomycetes; Pleosporales; Sporormiaceae; unidentified; Sporormiaceae_sp
New.ReferenceOTU4259	0	0	0	0	0	0	0	0	1	0	0	0	7	0	0	0	3	0	0	0	0	0	0	0	0	0	0	0	0	0	Fungi; Ascomycota; Dothideomycetes; Pleosporales; Sporormiaceae; unidentified; Sporormiaceae_sp
New.ReferenceOTU446	0	0	0	0	0	0	0	0	0	0	1	0	0	0	0	0	1	0	0	0	0	0	0	0	0	0	0	0	0	0	Fungi; Ascomycota; Dothideomycetes; Pleosporales; Sporormiaceae; unidentified; Sporormiaceae_sp
New.ReferenceOTU520	0	0	0	1	0	0	0	0	0	0	0	0	0	0	0	0	0	0	0	0	0	0	0	0	0	0	0	0	0	0	Fungi; Ascomycota; Dothideomycetes; Pleosporales; Sporormiaceae; unidentified; Sporormiaceae_sp
New.ReferenceOTU5688	0	0	0	0	0	0	0	0	1	0	0	0	0	0	0	0	0	0	0	0	0	0	0	0	0	0	0	0	0	0	Fungi; Ascomycota; Dothideomycetes; Pleosporales; Sporormiaceae; unidentified; Sporormiaceae_sp
New.ReferenceOTU6377	0	0	0	1	0	0	0	0	0	0	2	0	0	2	0	0	2	0	0	0	0	0	0	0	0	0	0	0	0	0	Fungi; Ascomycota; Dothideomycetes; Pleosporales; Sporormiaceae; unidentified; Sporormiaceae_sp
New.ReferenceOTU7750	0	0	0	0	0	0	0	0	0	0	0	0	0	0	5	0	0	0	0	0	1	1	0	0	0	0	1	2	0	5	Fungi; Ascomycota; Dothideomycetes; Pleosporales; Sporormiaceae; unidentified; Sporormiaceae_sp
New.CleanUp.ReferenceOTU2005	0	0	0	0	0	0	0	0	0	0	0	0	0	0	0	0	0	0	0	0	0	0	0	0	1	0	0	0	0	0	Fungi; Ascomycota; Dothideomycetes; Pleosporales; unidentified; unidentified; Pleosporales_sp
New.ReferenceOTU1207	0	0	0	0	0	0	0	0	0	0	0	0	0	0	1	0	0	0	0	0	0	0	0	0	0	0	0	0	0	0	Fungi; Ascomycota; Dothideomycetes; Pleosporales; unidentified; unidentified; Pleosporales_sp
New.ReferenceOTU149	0	0	1	0	0	0	0	0	0	1	0	0	0	2	0	0	0	0	0	0	0	0	0	0	0	0	0	0	0	0	Fungi; Ascomycota; Dothideomycetes; Pleosporales; unidentified; unidentified; Pleosporales_sp
New.ReferenceOTU1664	0	0	0	0	0	0	0	0	0	0	0	0	0	0	0	1	0	0	0	0	0	0	0	0	0	0	0	0	0	0	Fungi; Ascomycota; Dothideomycetes; Pleosporales; unidentified; unidentified; Pleosporales_sp

New.ReferenceOTU2916	0	0	0	0	0	0	0	0	0	0	0	0	1	0	0	2	0	0	0	0	0	0	0	0	0	0	0	0	0	0	Fungi; Ascomycota; Dothideomycetes; Pleosporales; unidentified; unidentified; Pleosporales_sp
New.ReferenceOTU4327	0	0	0	0	0	0	0	0	0	0	0	0	0	0	0	0	0	0	0	0	0	1	0	0	0	0	0	0	0	0	Fungi; Ascomycota; Dothideomycetes; Pleosporales; unidentified; unidentified; Pleosporales_sp
New.ReferenceOTU4659	0	0	0	0	0	0	0	0	0	0	0	0	1	0	0	0	0	0	0	0	0	0	0	0	0	0	0	0	0	0	Fungi; Ascomycota; Dothideomycetes; Pleosporales; unidentified; unidentified; Pleosporales_sp
New.ReferenceOTU4698	0	0	0	0	0	0	0	0	1	0	0	0	0	0	0	0	0	0	0	0	0	0	0	0	0	0	0	0	0	0	Fungi; Ascomycota; Dothideomycetes; Pleosporales; unidentified; unidentified; Pleosporales_sp
New.ReferenceOTU5529	0	0	0	0	0	0	0	0	0	0	0	0	0	0	0	0	0	0	0	1	0	0	0	0	0	0	0	0	0	0	Fungi; Ascomycota; Dothideomycetes; Pleosporales; unidentified; unidentified; Pleosporales_sp
New.ReferenceOTU5701	0	0	0	1	1	0	0	0	0	0	0	0	0	0	0	1	0	0	1	1	0	0	0	0	0	0	0	0	0	0	Fungi; Ascomycota; Dothideomycetes; Pleosporales; unidentified; unidentified; Pleosporales_sp
New.ReferenceOTU5995	0	0	0	0	0	0	0	0	0	0	0	0	0	0	0	0	0	0	0	0	0	0	0	0	3	0	0	0	0	0	Fungi; Ascomycota; Dothideomycetes; Pleosporales; unidentified; unidentified; Pleosporales_sp
New.ReferenceOTU6335	0	0	0	0	0	0	0	0	0	0	0	0	0	0	0	0	0	0	0	0	0	0	0	2	0	0	0	0	0	0	Fungi; Ascomycota; Dothideomycetes; Pleosporales; unidentified; unidentified; Pleosporales_sp
New.ReferenceOTU691	0	0	0	0	0	0	0	0	0	0	0	0	0	0	0	0	0	0	0	0	0	0	3	0	0	0	0	0	0	0	Fungi; Ascomycota; Dothideomycetes; Pleosporales; unidentified; unidentified; Pleosporales_sp
New.ReferenceOTU7226	0	0	0	0	2	3	0	0	0	0	0	0	0	0	0	0	0	0	0	0	0	0	0	0	0	0	0	0	0	0	Fungi; Ascomycota; Dothideomycetes; Pleosporales; unidentified; unidentified; Pleosporales_sp
New.ReferenceOTU7616	2	0	2	0	0	0	0	0	0	0	0	0	0	0	0	4	0	0	0	0	0	0	0	0	0	0	0	0	0	0	Fungi; Ascomycota; Dothideomycetes; Pleosporales; unidentified; unidentified; Pleosporales_sp
New.ReferenceOTU771	0	0	0	0	0	0	0	0	0	0	0	0	0	1	0	0	0	0	0	0	0	0	0	0	0	0	0	0	0	0	Fungi; Ascomycota; Dothideomycetes; Pleosporales; unidentified; unidentified; Pleosporales_sp
New.ReferenceOTU7853	0	0	0	0	0	0	0	0	0	0	0	0	0	0	0	0	0	0	3	0	0	0	0	0	0	0	0	0	0	0	Fungi; Ascomycota; Dothideomycetes; Pleosporales; unidentified; unidentified; Pleosporales_sp
New.ReferenceOTU787	0	0	0	2	0	0	0	0	1	0	0	0	0	0	0	0	0	0	0	0	0	0	0	0	0	0	0	0	0	0	Fungi; Ascomycota; Dothideomycetes; Pleosporales; unidentified; unidentified; Pleosporales_sp
SH030701.06FU_DQ993618_reps_singleton	0	0	1	0	0	0	0	0	0	0	0	0	0	0	0	0	0	0	0	0	0	0	0	0	0	0	0	0	0	0	Fungi; Ascomycota; Dothideomycetes; Pleosporales; unidentified; unidentified; Pleosporales_sp
SH092997.06FU_AB520485_reps	0	0	0	0	0	0	0	0	0	0	0	0	0	0	0	0	0	0	0	0	0	0	0	0	0	0	0	0	0	1	Fungi; Ascomycota; Dothideomycetes; Pleosporales; unidentified; unidentified; Pleosporales_sp
SH102856.06FU_EU686119_reps	1	0	0	0	0	7	1	0	1	0	0	1	0	0	0	0	0	0	0	1	0	0	0	0	0	0	0	0	0	0	Fungi; Ascomycota; Dothideomycetes; Pleosporales; unidentified; unidentified; Pleosporales_sp
SH104583.06FU_KC771502_reps	0	0	0	2	0	0	0	0	0	0	0	0	0	0	0	0	0	0	0	0	0	0	0	0	0	0	0	0	0	0	Fungi; Ascomycota; Dothideomycetes; Pleosporales; unidentified; unidentified; Pleosporales_sp
SH104584.06FU_JQ649380_reps	0	0	0	0	0	0	0	0	0	0	0	0	4	0	4	0	10	1	0	0	0	1	1	0	2	13	0	1	0	1	Fungi; Ascomycota; Dothideomycetes; Pleosporales; unidentified; unidentified; Pleosporales_sp
New.ReferenceOTU3845	0	0	0	0	0	0	0	0	0	1	0	0	0	0	0	0	0	0	0	0	0	0	0	0	0	0	0	0	0	0	Fungi; Ascomycota; Dothideomycetes; Pleosporales; unidentified; unidentified; Pleosporales_sp_E7212g
New.ReferenceOTU1141	0	0	0	0	0	0	0	0	0	0	0	0	0	1	0	0	0	0	0	0	0	0	0	0	0	0	0	0	0	0	Fungi; Ascomycota; Dothideomycetes; Pleosporales; unidentified; unidentified; Pleosporales_sp_REF142
New.CleanUp.ReferenceOTU1588	0	0	0	0	1	0	0	0	0	0	0	0	0	0	0	0	0	0	0	0	0	0	0	0	0	0	0	0	0	0	Fungi; Ascomycota; Dothideomycetes; unidentified; unidentified; unidentified; Dothideomycetes_sp
New.ReferenceOTU1011	0	0	0	0	0	0	3	0	0	0	0	0	0	0	0	0	0	0	0	0	0	0	0	0	0	0	0	0	0	0	Fungi; Ascomycota; Dothideomycetes; unidentified; unidentified; unidentified; Dothideomycetes_sp
New.ReferenceOTU1097	0	0	0	0	0	1	0	0	0	0	0	0	0	0	0	0	1	0	0	0	0	0	0	0	0	0	0	0	0	0	Fungi; Ascomycota; Dothideomycetes; unidentified; unidentified; unidentified; Dothideomycetes_sp
New.ReferenceOTU1147	0	0	0	0	0	1	0	0	0	0	0	0	1	0	0	0	0	0	0	0	0	0	0	0	0	0	0	0	0	0	Fungi; Ascomycota; Dothideomycetes; unidentified; unidentified; unidentified; Dothideomycetes_sp
New.ReferenceOTU127	0	0	0	1	0	0	0	0	0	0	0	0	0	0	0	0	0	0	0	0	0	0	0	0	0	0	0	0	0	0	Fungi; Ascomycota; Dothideomycetes; unidentified; unidentified; unidentified; Dothideomycetes_sp
New.ReferenceOTU1676	0	0	0	7	0	0	0	0	0	0	0	0	0	0	0	0	0	0	0	0	0	0	0	0	0	0	0	0	0	0	Fungi; Ascomycota; Dothideomycetes; unidentified; unidentified; unidentified; Dothideomycetes_sp
New.ReferenceOTU2104	0	0	0	0	0	0	0	0	0	0	0	0	0	0	0	0	1	0	0	0	0	0	0	0	0	0	0	0	0	0	Fungi; Ascomycota; Dothideomycetes; unidentified; unidentified; unidentified; Dothideomycetes_sp
New.ReferenceOTU230	0	0	0	0	0	0	0	1	0	0	0	0	0	0	0	0	0	0	0	0	0	0	0	0	0	0	0	0	0	0	Fungi; Ascomycota; Dothideomycetes; unidentified; unidentified; unidentified; Dothideomycetes_sp
New.ReferenceOTU2586	0	0	0	0	0	0	0	0	0	0	0	0	0	0	0	0	1	0	0	0	0	0	0	0	0	0	0	0	0	0	Fungi; Ascomycota; Dothideomycetes; unidentified; unidentified; unidentified; Dothideomycetes_sp
New.ReferenceOTU278	0	0	0	0	0	3	0	0	0	0	0	0	0	0	0	0	0	0	0	0	0	0	0	0	0	0	0	0	0	2	Fungi; Ascomycota; Dothideomycetes; unidentified; unidentified; unidentified; Dothideomycetes_sp
New.ReferenceOTU2798	0	1	0	0	0	0	0	0	0	1	0	0	0	0	0	1	2	0	0	0	0	0	0	2	0	0	0	0	0	0	Fungi; Ascomycota; Dothideomycetes; unidentified; unidentified; unidentified; Dothideomycetes_sp
New.ReferenceOTU2956	0	0	0	0	0	0	0	0	2	0	0	0	0	0	0	0	0	0	0	0	0	0	0	0	0	0	0	0	0	0	Fungi; Ascomycota; Dothideomycetes; unidentified; unidentified; unidentified; Dothideomycetes_sp
New.ReferenceOTU3052	5	0	1	3	3	3	0	4	0	0	0	5	0	0	1	0	1	0	0	0	0	0	0	1	0	1	1	3	2	0	Fungi; Ascomycota; Dothideomycetes; unidentified; unidentified; unidentified; Dothideomycetes_sp
New.ReferenceOTU3177	0	0	0	0	1	0	0	0	0	0	0	0	0	0	0	0	0	0	0	0	0	0	0	0	0	0	0	0	0	0	Fungi; Ascomycota; Dothideomycetes; unidentified; unidentified; unidentified; Dothideomycetes_sp
New.ReferenceOTU3598	0	0	0	0	0	0	0	0	0	0	0	0	0	0	0	0	0	0	0	0	0	0	0	0	0	0	0	1	0	0	Fungi; Ascomycota; Dothideomycetes; unidentified; unidentified; unidentified; Dothideomycetes_sp
New.ReferenceOTU4180	1	0	0	7	0	0	0	0	0	1	0	0	0	0	0	0	0	0	0	0	0	0	0	0	0	0	0	0	0	0	Fungi; Ascomycota; Dothideomycetes; unidentified; unidentified; unidentified; Dothideomycetes_sp
New.ReferenceOTU4309	0	0	0	0	0	0	0	0	0	0	0	0	0	0	0	0	0	0	0	0	0	0	0	0	0	0	0	0	0	5	Fungi; Ascomycota; Dothideomycetes; unidentified; unidentified; unidentified; Dothideomycetes_sp
New.ReferenceOTU4763	0	0	0	0	0	0	0	0	0	0	1	0	0	0	0	0	0	0	0	0	0	0	0	0	0	0	0	0	0	0	Fungi; Ascomycota; Dothideomycetes; unidentified; unidentified; unidentified; Dothideomycetes_sp
New.ReferenceOTU5107	0	0	0	0	0	0	0	0	0	0	0	0	0	0	0	0	0	0	0	0	0	2	0	0	0	0	0	0	0	0	Fungi; Ascomycota; Dothideomycetes; unidentified; unidentified; unidentified; Dothideomycetes_sp
New.ReferenceOTU5211	0	0	0	0	0	0	0	0	0	0	0	0	0	0	0	0	0	0	0	0	0	0	0	0	0	0	0	0	0	1	Fungi; Ascomycota; Dothideomycetes; unidentified; unidentified; unidentified; Dothideomycetes_sp
New.ReferenceOTU5677	0	0	0	0	0	0	0	0	0	0	1	0	0	0	0	0	0	0	0	0	0	0	0	0	0	0	0	0	0	0	Fungi; Ascomycota; Dothideomycetes; unidentified; unidentified; unidentified; Dothideomycetes_sp
New.ReferenceOTU6030	0	2	0	0	0	0	0	0	1	0	0	0	0	0	0	0	0	0	0	0	0	0	0	0	0	0	0	0	0	0	Fungi; Ascomycota; Dothideomycetes; unidentified; unidentified; unidentified; Dothideomycetes_sp
New.ReferenceOTU6203	0	0	1	0	0	0	0	0	0	0	0	0	0	0	0	0	0	0	0	0	0	0	0	0	0	0	0	0	0	0	Fungi; Ascomycota; Dothideomycetes; unidentified; unidentified; unidentified; Dothideomycetes_sp
New.ReferenceOTU6350	0	0	1	0	0	0	0	0	0	0	0	0	0	0	0	0	0	0	0	0	0	0	0	0	0	0	0	0	0	0	Fungi; Ascomycota; Dothideomycetes; unidentified; unidentified; unidentified; Dothideomycetes_sp
New.ReferenceOTU6466	0	0	0	0	0	0	0	0	0	0	0	0	0	0	0	0	0	0	0	0	1	0	0	0	0	0	0	0	0	0	Fungi; Ascomycota; Dothideomycetes; unidentified; unidentified; unidentified; Dothideomycetes_sp
New.ReferenceOTU6510	1	0	0	1	0	0	0	0	0	0	1	0	0	0	0	0	0	0	0	0	0	0	0	0	0	0	0	0	2	0	Fungi; Ascomycota; Dothideomycetes; unidentified; unidentified; unidentified; Dothideomycetes_sp
New.ReferenceOTU6555	0	0	0	0	0	0	0	0	0	0	0	0	0	0	0	0	0	0	1	0	0	0	0	0	0	0	0	0	0	0	Fungi; Ascomycota; Dothideomycetes; unidentified; unidentified; unidentified; Dothideomycetes_sp
New.ReferenceOTU6596	0	0	0	0	0	0	0	0	0	0	0	0	0	0	0	0	0	11	0	0	0	0	0	0	0	0	0	0	0	0	Fungi; Ascomycota; Dothideomycetes; unidentified; unidentified; unidentified; Dothideomycetes_sp
New.ReferenceOTU664	0	0	0	0	0	0	0	0	0	0	0	0	0	0	0	0	0	0	0	0	0	7	0	0	0	0	0	0	0	0	Fungi; Ascomycota; Dothideomycetes; unidentified; unidentified; unidentified; Dothideomycetes_sp
New.ReferenceOTU6838	0	0	0	0	0	0	0	0	0	0	0	0	0	0	0	0	0	0	0	0	0	0	0	0	2	0	0	0	0	1	Fungi; Ascomycota; Dothideomycetes; unidentified; unidentified; unidentified; Dothideomycetes_sp
New.ReferenceOTU7233	1	0	0	1	0	0	0	0	0	0	0	0	0	0	0	0	0	0	0	0	0	0	0	0	0	0	0	0	0	0	Fungi; Ascomycota; Dothideomycetes; unidentified; unidentified; unidentified; Dothideomycetes_sp
New.ReferenceOTU741	0	0	0	0	0	0	0	0	0	0	0	0	0	0	0	0	0	0	4	0	0	0	0	0	0	0	0	0	0	0	Fungi; Ascomycota; Dothideomycetes; unidentified; unidentified; unidentified; Dothideomycetes_sp
New.ReferenceOTU7555	1	0	0	0	0	0	0	0	0	0	0	0	0	0	0	0	0	0	0	0	0	0	0	0	0	0	0	0	0	0	Fungi; Ascomycota; Dothideomycetes; unidentified; unidentified; unidentified; Dothideomycetes_sp
New.ReferenceOTU7588	0	0	0	0	0	0	0	1	0	0	0	0	0	0	0	0	0	0	0	0	0	0	0	0	0	0	0	0	0	0	Fungi; Ascomycota; Dothideomycetes; unidentified; unidentified; unidentified; Dothideomycetes_sp
New.ReferenceOTU7639	0	0	0	0	0	0	0	0	0	0	0	0	0	0	0	0	0	0	0	0	0	0	0	0	0	0	0	0	0	1	Fungi; Ascomycota; Dothideomycetes; unidentified; unidentified; unidentified; Dothideomycetes_sp
New.ReferenceOTU7673	0	0	0	0	0	0	0	0	0	0	0	0	0	0	0	0	0	0	0	0	1	0	0	0	0	0	0	0	0	0	Fungi; Ascomycota; Dothideomycetes; unidentified; unidentified; unidentified; Dothideomycetes_sp
New.ReferenceOTU7872	10	0	0	0	0	0	0	0	0	0	0	0	0	0	0	0	0	0	0	0	0	0	0	0	0	0	0	0	0	0	Fungi; Ascomycota; Dothideomycetes; unidentified; unidentified; unidentified; Dothideomycetes_sp
SH018009.06FU_JN890259_reps_singleton	0	0	0	0	0	0	0	0	0	0	0	0	0	1	0	0	0	0	0	0	0	0	0	0	0	0	0	0	0	0	Fungi; Ascomycota; Dothideomycetes; unidentified; unidentified; unidentified; Dothideomycetes_sp
SH023841.06FU_EU917081_reps_singleton	0	0	0	0	0	0	0	0	0	0	9	0	0	3	0	0	0	0	0	0	0	0	0	0	0	0	0	0	0	0	Fungi; Ascomycota; Dothideomycetes; unidentified; unidentified; unidentified; Dothideomycetes_sp
SH111441.06FU_JQ867364_reps	0	0	0	0	0	2	1	0	0	0	0	0	0	0	1	0	0	0	0	0	0	0	0	0	0	0	0	0	0	1	Fungi; Ascomycota; Dothideomycetes; unidentified; unidentified; unidentified; Dothideomycetes_sp
SH127387.06FU_JX966607_reps	0	0	0	0	0	0	0	0	0	0	0	0	0	0	0	0	0	0	0	0	0	0	2	0	0	0	0	0	0	0	Fungi; Ascomycota; Dothideomycetes; unidentified; unidentified; unidentified; Dothideomycetes_sp
SH145428.06FU_KC978016_reps	0	0	0	0	0	0	0	0	0	0	0	1	0	0	0	0	0	0	0	0	0	0	0	0	0	0	0	0	0	0	Fungi; Ascomycota; Dothideomycetes; unidentified; unidentified; unidentified; Dothideomycetes_sp
SH004764.06FU_JQ388930_reps_singleton	0	0	0	0	0	0	0	0	0	0	0	0	0	0	0	0	0	0	0	0	0	0	0	0	0	0	0	0	0	2	Fungi; Ascomycota; Dothideomycetes; unidentified; unidentified; unidentified; Dothideomycetes_sp_CCG_2012
New.ReferenceOTU7219	0	0	0	0	1	0	0	0	7	0	0	3	0	0	0	0	0	0	0	0	0	0	1	0	22	0	0	1	0	0	Fungi; Ascomycota; Dothideomycetes; unidentified; unidentified; unidentified; Dothideomycetes_sp_CHTAM23
New.ReferenceOTU7423	0	0	0	0	0	0	0	0	0	0	0	0	0	0	0	0	0	0	5	0	0	0	0	0	0	0	0	0	0	0	Fungi; Ascomycota; Dothideomycetes; unidentified; unidentified; unidentified; Dothideomycetes_sp_genotype_375
New.ReferenceOTU2554	0	0	0	0	0	0	0	0	0	0	0	0	0	0	0	1	0	0	0	0	0	0	0	0	0	0	0	0	0	0	Fungi; Ascomycota; Dothideomycetes; unidentified; unidentified; unidentified; Dothideomycetes_sp_MX139
New.ReferenceOTU3086	0	0	0	0	0	1	3	0	1	0	0	2	0	0	0	7	1	0	0	0	0	0	0	0	0	0	0	0	0	0	Fungi; Ascomycota; Dothideomycetes; unidentified; unidentified; unidentified; Dothideomycetes_sp_MX139
New.ReferenceOTU4575	3	0	0	1	0	0	0	12	2	0	0	0	2	3	0	4	0	0	0	0	0	2	0	0	0	0	0	0	0	0	Fungi; Ascomycota; Dothideomycetes; unidentified; unidentified; unidentified; Dothideomycetes_sp_MX139
New.ReferenceOTU7529	0	0	0	0	0	1	0	0	0	0	0	0	0	0	0	0	0	0	0	0	0	0	0	0	0	0	0	0	0	0	Fungi; Ascomycota; Dothideomycetes; unidentified; unidentified; unidentified; Dothideomycetes_sp_MX139
SH449949.06FU_JQ905740_reps_singleton	1	0	5	1	2	4	10	3	10	1	2	5	21	2	0	32	9	0	0	0	0	0	0	2	0	0	0	0	0	0	Fungi; Ascomycota; Dothideomycetes; unidentified; unidentified; unidentified; Dothideomycetes_sp_MX139
New.CleanUp.ReferenceOTU1743	0	0	0	0	0	0	0	0	0	0	0	0	0	0	0	0	0	0	0	0	0	0	0	0	0	0	0	0	0	1	Fungi; Ascomycota; Dothideomycetes; Venturiales; Sympoventuriaceae; unidentified; Sympoventuriaceae_sp
New.ReferenceOTU1867	0	0	0	0	0	0	0	1	0	0	0	0	0	3	0	0	0	0	0	0	0	0	0	0	0	0	0	0	0	0	Fungi; Ascomycota; Dothideomycetes; Venturiales; Sympoventuriaceae; unidentified; Sympoventuriaceae_sp
New.ReferenceOTU2016	0	0	0	0	0	0	0	0	0	0	0	0	0	0	1	0	0	0	0	0	0	0	0	0	0	0	0	0	0	0	Fungi; Ascomycota; Dothideomycetes; Venturiales; unidentified; unidentified; Venturiales_sp
New.ReferenceOTU4061	0	0	0	0	0	0	0	0	0	1	0	0	0	0	0	3	1	0	0	0	0	0	0	0	0	0	0	0	0	0	Fungi; Ascomycota; Dothideomycetes; Venturiales; unidentified; unidentified; Venturiales_sp
New.CleanUp.ReferenceOTU496	1	0	0	0	0	0	0	0	0	0	0	0	0	0	0	0	0	0	0	0	0	0	0	0	0	0	0	0	0	0	Fungi; Ascomycota; Dothideomycetes; Venturiales; Venturiaceae; unidentified; Venturiaceae_sp
New.ReferenceOTU1366	0	0	0	0	0	0	0	0	0	0	0	1	0	0	0	0	0	0	0	0	0	0	0	0	0	0	0	0	0	0	Fungi; Ascomycota; Dothideomycetes; Venturiales; Venturiaceae; unidentified; Venturiaceae_sp
New.ReferenceOTU2811	0	0	0	0	0	0	0	0	0	0	0	0	0	0	0	2	0	0	0	0	0	0	0	0	0	0	0	0	0	0	Fungi; Ascomycota; Dothideomycetes; Venturiales; Venturiaceae; unidentified; Venturiaceae_sp
New.ReferenceOTU3933	0	0	0	0	0	0	0	1	0	0	0	0	0	0	0	0	0	0	0	0	0	0	0	0	0	0	0	0	0	0	Fungi; Ascomycota; Dothideomycetes; Venturiales; Venturiaceae; unidentified; Venturiaceae_sp
New.ReferenceOTU3988	0	0	0	0	0	0	0	0	0	1	0	0	0	0	0	0	0	0	0	0	0	0	0	0	0	0	0	0	0	0	Fungi; Ascomycota; Dothideomycetes; Venturiales; Venturiaceae; unidentified; Venturiaceae_sp
New.ReferenceOTU4804	1	0	0	0	0	0	0	0	0	0	0	0	0	0	0	0	0	0	0	0	0	0	0	0	0	0	0	0	0	0	Fungi; Ascomycota; Dothideomycetes; Venturiales; Venturiaceae; unidentified; Venturiaceae_sp
New.ReferenceOTU5977	1	0	0	0	0	0	0	0	0	0	0	0	0	0	0	0	0	0	0	0	0	0	0	0	0	0	0	0	0	0	Fungi; Ascomycota; Dothideomycetes; Venturiales; Venturiaceae; unidentified; Venturiaceae_sp
New.ReferenceOTU7296	1	0	0	0	0	0	0	0	0	0	0	0	0	0	0	0	0	0	0	0	0	0	0	0	0	0	0	0	0	0	Fungi; Ascomycota; Dothideomycetes; Venturiales; Venturiaceae; unidentified; Venturiaceae_sp
New.ReferenceOTU7439	2	0	0	0	0	0	0	0	0	0	0	0	0	0	0	0	0	0	0	0	0	0	0	0	0	0	0	0	0	0	Fungi; Ascomycota; Dothideomycetes; Venturiales; Venturiaceae; unidentified; Venturiaceae_sp
New.ReferenceOTU7494	1	0	0	1	0	0	0	0	0	0	0	0	0	0	0	0	0	0	0	0	0	0	0	0	0	0	0	0	0	0	Fungi; Ascomycota; Dothideomycetes; Venturiales; Venturiaceae; unidentified; Venturiaceae_sp
New.ReferenceOTU4717	0	0	0	0	0	0	0	0	0	0	0	0	0	0	27	0	0	0	0	0	0	0	0	0	0	0	0	0	0	0	Fungi; Ascomycota; Eurotiomycetes; Chaetothyriales; Chaetothyriaceae; Brunneodinemasporium; Brunneodinemasporium_brasiliense
New.ReferenceOTU5044	0	0	0	0	0	0	0	0	0	0	0	0	0	0	0	0	0	0	0	0	0	0	0	0	0	0	0	0	3	0	Fungi; Ascomycota; Eurotiomycetes; Chaetothyriales; Chaetothyriaceae; Brunneodinemasporium; Brunneodinemasporium_brasiliense
New.CleanUp.ReferenceOTU1714	1	0	0	0	0	0	0	0	0	0	0	0	0	0	0	0	0	0	0	0	0	0	0	0	0	0	0	0	0	0	Fungi; Ascomycota; Eurotiomycetes; Chaetothyriales; Herpotrichiellaceae; unidentified; Herpotrichiellaceae_sp
New.ReferenceOTU1648	0	0	0	0	0	0	0	0	0	0	0	0	0	0	0	0	0	0	0	0	0	0	0	0	0	0	0	0	0	1	Fungi; Ascomycota; Eurotiomycetes; Chaetothyriales; Herpotrichiellaceae; unidentified; Herpotrichiellaceae_sp
New.ReferenceOTU1740	0	0	0	0	0	0	0	0	0	0	0	0	0	3	0	0	0	0	0	0	0	0	0	0	0	0	0	0	0	0	Fungi; Ascomycota; Eurotiomycetes; Chaetothyriales; Herpotrichiellaceae; unidentified; Herpotrichiellaceae_sp
New.ReferenceOTU1930	3	0	0	0	0	0	0	0	0	0	1	0	1	0	0	0	1	0	0	0	0	0	0	0	0	0	0	0	0	0	Fungi; Ascomycota; Eurotiomycetes; Chaetothyriales; Herpotrichiellaceae; unidentified; Herpotrichiellaceae_sp
New.ReferenceOTU2144	1	0	0	0	0	0	0	0	0	0	0	0	0	0	0	0	0	0	0	0	0	0	0	1	0	0	0	0	0	0	Fungi; Ascomycota; Eurotiomycetes; Chaetothyriales; Herpotrichiellaceae; unidentified; Herpotrichiellaceae_sp
New.ReferenceOTU2537	0	0	0	0	0	0	0	0	0	0	0	0	0	0	0	0	0	0	0	0	0	0	1	0	0	0	0	0	0	0	Fungi; Ascomycota; Eurotiomycetes; Chaetothyriales; Herpotrichiellaceae; unidentified; Herpotrichiellaceae_sp
New.ReferenceOTU2544	0	0	0	0	0	0	0	0	0	0	0	0	0	0	0	0	0	0	1	0	0	0	0	0	0	0	0	0	0	0	Fungi; Ascomycota; Eurotiomycetes; Chaetothyriales; Herpotrichiellaceae; unidentified; Herpotrichiellaceae_sp
New.ReferenceOTU346	0	0	1	0	0	0	0	0	0	0	0	0	0	0	0	0	0	0	0	0	0	0	0	0	0	0	0	0	0	0	Fungi; Ascomycota; Eurotiomycetes; Chaetothyriales; Herpotrichiellaceae; unidentified; Herpotrichiellaceae_sp
New.ReferenceOTU374	1	0	0	0	0	0	0	0	0	0	0	0	0	0	0	0	0	0	0	0	1	0	0	0	0	0	0	0	0	0	Fungi; Ascomycota; Eurotiomycetes; Chaetothyriales; Herpotrichiellaceae; unidentified; Herpotrichiellaceae_sp
New.ReferenceOTU4084	0	0	2	0	0	0	0	0	0	0	0	0	0	0	0	0	0	0	1	1	0	0	0	0	0	0	0	0	0	0	Fungi; Ascomycota; Eurotiomycetes; Chaetothyriales; Herpotrichiellaceae; unidentified; Herpotrichiellaceae_sp
New.ReferenceOTU483	0	0	0	0	0	0	0	0	0	0	0	0	0	0	1	0	0	0	0	0	0	0	0	0	0	0	0	0	0	0	Fungi; Ascomycota; Eurotiomycetes; Chaetothyriales; Herpotrichiellaceae; unidentified; Herpotrichiellaceae_sp
New.ReferenceOTU4871	1	0	0	0	0	0	0	0	1	0	0	0	0	0	0	0	0	0	0	0	0	0	0	0	0	0	0	0	0	0	Fungi; Ascomycota; Eurotiomycetes; Chaetothyriales; Herpotrichiellaceae; unidentified; Herpotrichiellaceae_sp
New.ReferenceOTU5318	0	0	0	0	0	0	0	0	0	0	0	0	2	0	0	0	0	0	0	0	0	0	0	0	0	0	0	0	0	0	Fungi; Ascomycota; Eurotiomycetes; Chaetothyriales; Herpotrichiellaceae; unidentified; Herpotrichiellaceae_sp

New.ReferenceOTU5727	0	0	0	0	0	0	0	0	0	0	0	0	1	0	0	0	0	0	0	0	0	0	0	0	0	0	0	0	0	0	Fungi; Ascomycota; Eurotiomycetes; Chaetothyriales; Herpotrichiellaceae; unidentified; Herpotrichiellaceae_sp
New.ReferenceOTU5980	0	0	1	0	0	0	0	0	0	0	0	0	3	0	0	0	0	0	0	0	0	0	0	0	0	0	0	0	0	0	Fungi; Ascomycota; Eurotiomycetes; Chaetothyriales; Herpotrichiellaceae; unidentified; Herpotrichiellaceae_sp
New.ReferenceOTU600	0	0	0	0	1	0	0	0	0	0	0	0	0	0	0	0	0	0	0	0	0	0	0	0	0	0	0	0	0	0	Fungi; Ascomycota; Eurotiomycetes; Chaetothyriales; Herpotrichiellaceae; unidentified; Herpotrichiellaceae_sp
New.ReferenceOTU6731	2	0	0	0	0	0	0	0	0	0	0	0	0	0	0	0	0	0	0	0	0	0	0	0	0	0	0	0	0	0	Fungi; Ascomycota; Eurotiomycetes; Chaetothyriales; Herpotrichiellaceae; unidentified; Herpotrichiellaceae_sp
New.ReferenceOTU6733	0	0	1	0	0	0	0	0	0	0	0	0	0	0	0	0	0	0	0	0	0	0	0	0	0	0	0	0	0	0	Fungi; Ascomycota; Eurotiomycetes; Chaetothyriales; Herpotrichiellaceae; unidentified; Herpotrichiellaceae_sp
New.ReferenceOTU703	0	0	0	0	1	0	0	0	0	0	0	0	0	0	0	0	0	0	0	0	0	0	0	0	0	0	0	0	0	0	Fungi; Ascomycota; Eurotiomycetes; Chaetothyriales; Herpotrichiellaceae; unidentified; Herpotrichiellaceae_sp
New.ReferenceOTU7129	0	0	0	5	0	0	0	0	0	0	0	1	0	4	0	0	1	0	0	0	0	0	0	0	0	0	0	0	0	0	Fungi; Ascomycota; Eurotiomycetes; Chaetothyriales; Herpotrichiellaceae; unidentified; Herpotrichiellaceae_sp
New.ReferenceOTU7374	0	0	0	0	0	0	0	0	0	0	0	0	2	0	0	0	0	0	0	0	0	0	0	0	0	0	0	0	0	0	Fungi; Ascomycota; Eurotiomycetes; Chaetothyriales; Herpotrichiellaceae; unidentified; Herpotrichiellaceae_sp
New.ReferenceOTU7743	0	0	0	0	1	0	0	0	0	0	0	0	0	0	0	0	0	0	0	0	0	0	0	0	0	0	0	0	0	0	Fungi; Ascomycota; Eurotiomycetes; Chaetothyriales; Herpotrichiellaceae; unidentified; Herpotrichiellaceae_sp
New.ReferenceOTU778	0	0	0	0	0	0	0	0	0	0	0	0	0	0	0	0	0	0	0	2	0	0	0	0	0	0	0	0	0	0	Fungi; Ascomycota; Eurotiomycetes; Chaetothyriales; Herpotrichiellaceae; unidentified; Herpotrichiellaceae_sp
New.ReferenceOTU2640	0	0	0	0	0	0	0	0	0	0	0	0	0	0	0	0	1	0	0	0	0	0	0	0	0	0	0	0	0	0	Fungi; Ascomycota; Eurotiomycetes; Chaetothyriales; Herpotrichiellaceae; unidentified; Herpotrichiellaceae_sp_E9000c
New.ReferenceOTU4064	0	0	0	0	0	0	0	0	3	0	0	0	0	1	0	0	0	0	0	0	0	0	0	0	0	0	0	0	0	0	Fungi; Ascomycota; Eurotiomycetes; Chaetothyriales; Herpotrichiellaceae; unidentified; uncultured_Cladophialophora
SH085860.06FU_KF435240_reps	0	0	0	0	0	0	0	0	0	0	0	0	0	0	1	0	0	0	0	0	0	0	0	0	0	0	0	4	0	0	Fungi; Ascomycota; Eurotiomycetes; Chaetothyriales; Incertae_sedis; Coniosporium; Coniosporium_sp
New.ReferenceOTU6793	0	0	0	0	0	0	0	0	0	0	0	0	0	0	0	0	0	0	0	0	1	0	0	0	0	0	0	0	0	0	Fungi; Ascomycota; Eurotiomycetes; Chaetothyriales; Incertae_sedis; Coniosporium; Coniosporium_sp_SL1131
New.ReferenceOTU1319	0	0	0	0	0	0	0	0	0	0	0	0	0	0	3	0	0	0	0	0	0	0	0	0	0	0	0	0	0	0	Fungi; Ascomycota; Eurotiomycetes; Chaetothyriales; unidentified; unidentified; Chaetothyriales_sp
New.ReferenceOTU1967	0	0	0	0	0	0	0	0	0	0	0	0	0	0	0	0	0	0	0	0	0	0	1	0	0	0	0	0	0	0	Fungi; Ascomycota; Eurotiomycetes; Chaetothyriales; unidentified; unidentified; Chaetothyriales_sp
New.ReferenceOTU1976	0	0	0	0	1	0	0	0	0	0	0	0	0	0	0	0	0	0	0	0	0	0	0	0	0	0	0	0	0	0	Fungi; Ascomycota; Eurotiomycetes; Chaetothyriales; unidentified; unidentified; Chaetothyriales_sp
New.ReferenceOTU2056	0	0	0	0	0	0	0	0	0	0	0	0	0	0	0	0	0	0	0	0	0	0	0	0	0	0	0	0	0	1	Fungi; Ascomycota; Eurotiomycetes; Chaetothyriales; unidentified; unidentified; Chaetothyriales_sp
New.ReferenceOTU3072	0	0	0	0	0	0	0	0	0	0	0	0	0	0	0	0	0	0	0	0	0	0	0	0	0	0	0	2	0	0	Fungi; Ascomycota; Eurotiomycetes; Chaetothyriales; unidentified; unidentified; Chaetothyriales_sp
New.ReferenceOTU3215	0	0	0	0	0	0	0	0	0	0	0	0	0	0	0	0	0	0	0	0	0	1	0	0	0	0	0	0	0	1	Fungi; Ascomycota; Eurotiomycetes; Chaetothyriales; unidentified; unidentified; Chaetothyriales_sp
New.ReferenceOTU3412	2	0	0	0	0	0	0	0	0	0	0	0	0	0	0	0	0	0	0	0	0	0	0	0	0	0	0	0	0	0	Fungi; Ascomycota; Eurotiomycetes; Chaetothyriales; unidentified; unidentified; Chaetothyriales_sp
New.ReferenceOTU3747	0	0	0	0	0	0	0	2	0	0	0	0	0	0	0	0	0	0	0	0	0	0	0	0	0	0	0	0	0	0	Fungi; Ascomycota; Eurotiomycetes; Chaetothyriales; unidentified; unidentified; Chaetothyriales_sp
New.ReferenceOTU5307	0	0	0	0	0	0	0	0	0	0	0	0	0	0	0	0	0	0	0	0	0	0	0	0	0	0	0	0	0	1	Fungi; Ascomycota; Eurotiomycetes; Chaetothyriales; unidentified; unidentified; Chaetothyriales_sp
New.ReferenceOTU7387	0	0	0	3	0	0	0	0	0	0	0	0	0	0	0	0	0	0	0	0	0	0	0	0	0	0	0	0	0	0	Fungi; Ascomycota; Eurotiomycetes; Chaetothyriales; unidentified; unidentified; Chaetothyriales_sp
New.ReferenceOTU7512	0	0	0	0	0	0	0	0	0	0	0	0	1	0	0	0	0	0	0	0	0	1	0	0	0	0	0	1	0	0	Fungi; Ascomycota; Eurotiomycetes; Chaetothyriales; unidentified; unidentified; Chaetothyriales_sp
SH109291.06FU_EU888622_reps	0	0	0	0	0	0	0	0	0	0	0	0	0	0	0	0	0	0	0	0	0	1	0	0	0	0	0	0	0	0	Fungi; Ascomycota; Eurotiomycetes; Chaetothyriales; unidentified; unidentified; Chaetothyriales_sp
SH109298.06FU_UDB002648_reps	0	0	0	0	0	0	0	0	0	0	0	0	0	0	0	0	0	0	0	0	0	1	0	0	0	0	0	0	0	1	Fungi; Ascomycota; Eurotiomycetes; Chaetothyriales; unidentified; unidentified; Chaetothyriales_sp
New.ReferenceOTU2599	0	0	0	0	1	0	0	0	0	0	0	0	0	0	0	0	0	0	0	0	0	0	0	0	0	0	0	0	0	0	Fungi; Ascomycota; Eurotiomycetes; Chaetothyriales; unidentified; unidentified; Chaetothyriales_sp_CN_Cre_Bo3_5
SH017738.06FU_HQ634621_reps_singleton	0	0	0	0	2	0	0	0	0	0	0	0	0	0	0	0	0	0	0	0	0	0	0	0	0	0	0	0	0	0	Fungi; Ascomycota; Eurotiomycetes; Chaetothyriales; unidentified; unidentified; Chaetothyriales_sp_CN_Cre_Bo3_5
New.ReferenceOTU3248	0	0	0	0	0	0	0	0	0	0	6	0	0	0	0	0	0	0	0	0	0	0	0	0	0	0	0	0	0	0	Fungi; Ascomycota; Eurotiomycetes; Chaetothyriales; unidentified; unidentified; Chaetothyriales_sp_EXP0491F
New.ReferenceOTU6723	0	0	0	0	0	0	0	0	0	0	0	0	0	0	0	0	2	0	0	0	0	0	0	0	0	0	0	0	0	0	Fungi; Ascomycota; Eurotiomycetes; Chaetothyriales; unidentified; unidentified; Chaetothyriales_sp_M_Mo9
New.ReferenceOTU1514	0	0	0	0	0	0	0	0	0	0	1	0	0	0	0	0	0	0	0	0	0	0	0	0	0	0	0	0	0	0	Fungi; Ascomycota; Eurotiomycetes; Chaetothyriales; unidentified; unidentified; Chaetothyriales_sp_MSX_47445
New.ReferenceOTU2921	0	0	0	0	0	0	0	0	0	0	0	0	0	0	0	0	0	0	0	0	0	2	1	0	1	0	0	0	0	0	Fungi; Ascomycota; Eurotiomycetes; Chaetothyriales; unidentified; unidentified; Chaetothyriales_sp_MSX_47445
New.ReferenceOTU6993	0	0	0	0	0	0	0	0	0	0	0	0	0	0	0	0	0	0	0	1	0	0	0	0	0	0	0	0	0	0	Fungi; Ascomycota; Eurotiomycetes; Chaetothyriales; unidentified; unidentified; Chaetothyriales_sp_MSX_47445
New.ReferenceOTU7618	0	0	0	0	0	0	0	0	0	0	0	0	0	0	0	0	0	0	0	0	0	6	0	0	1	0	0	0	0	0	Fungi; Ascomycota; Eurotiomycetes; Chaetothyriales; unidentified; unidentified; Chaetothyriales_sp_MSX_47445
New.ReferenceOTU996	0	0	0	0	0	0	0	0	0	0	0	0	0	0	2	0	0	0	0	0	7	0	1	0	0	0	0	0	0	0	Fungi; Ascomycota; Eurotiomycetes; Chaetothyriales; unidentified; unidentified; uncultured_Chaetothyriales
New.ReferenceOTU4925	0	0	0	0	0	0	0	0	0	0	0	0	0	0	0	0	0	0	6	0	0	0	0	0	0	0	0	0	0	0	Fungi; Ascomycota; Eurotiomycetes; Onygenales; Incertae_sedis; Arachnotheca; Arachnotheca_glomerata
New.ReferenceOTU843	0	0	0	0	0	0	0	0	0	0	0	0	0	2	0	0	0	0	0	0	1	0	0	0	0	0	0	0	0	0	Fungi; Ascomycota; Eurotiomycetes; Onygenales; Incertae_sedis; Arachnotheca; Arachnotheca_glomerata
SH439624.06FU_KF477241_reps_singleton	0	0	0	0	0	0	0	0	0	0	0	0	0	0	0	0	0	0	1	0	0	0	0	0	0	0	0	0	0	0	Fungi; Ascomycota; Eurotiomycetes; Onygenales; Incertae_sedis; Arachnotheca; Arachnotheca_glomerata
New.ReferenceOTU1843	0	0	0	0	0	0	0	0	0	0	0	0	0	0	0	0	0	0	0	4	0	1	0	0	0	0	0	0	0	0	Fungi; Ascomycota; Eurotiomycetes; Onygenales; Onygenaceae; unidentified; Onygenaceae_sp
New.ReferenceOTU2451	0	0	0	0	0	0	0	0	0	0	0	0	0	0	0	0	0	0	0	1	0	0	0	0	0	0	0	0	0	0	Fungi; Ascomycota; Eurotiomycetes; Onygenales; Onygenaceae; unidentified; Onygenaceae_sp
New.ReferenceOTU2491	0	0	0	0	0	0	0	0	0	0	0	0	0	0	0	0	0	0	0	1	0	0	0	0	0	0	0	0	0	0	Fungi; Ascomycota; Eurotiomycetes; Onygenales; Onygenaceae; unidentified; Onygenaceae_sp
New.ReferenceOTU3339	0	0	0	0	0	0	0	0	0	0	0	0	0	0	0	0	0	0	0	1	0	0	0	0	0	0	0	0	0	0	Fungi; Ascomycota; Eurotiomycetes; Onygenales; Onygenaceae; unidentified; Onygenaceae_sp
New.ReferenceOTU3701	0	0	0	0	0	0	0	0	0	0	0	0	0	0	0	0	0	15	0	0	0	0	0	1	0	0	0	0	0	0	Fungi; Ascomycota; Eurotiomycetes; Onygenales; Onygenaceae; unidentified; Onygenaceae_sp
New.ReferenceOTU6432	0	0	2	1	0	0	0	0	4	3	2	0	0	1	0	0	1	0	2	6	0	2	0	0	0	0	0	3	0	0	Fungi; Ascomycota; Eurotiomycetes; Onygenales; Onygenaceae; unidentified; Onygenaceae_sp
New.ReferenceOTU7664	0	0	0	1	0	0	0	0	0	0	0	0	0	0	0	0	0	5	0	0	0	0	0	0	0	0	0	0	0	0	Fungi; Ascomycota; Eurotiomycetes; Onygenales; Onygenaceae; unidentified; Onygenaceae_sp
New.ReferenceOTU2878	0	0	0	0	0	0	0	0	0	0	0	0	0	0	0	0	0	1	0	0	0	1	1	0	0	0	0	0	0	0	Fungi; Ascomycota; Eurotiomycetes; Onygenales; Onygenaceae; unidentified; Onygenaceae_sp_UAMH_3124
New.ReferenceOTU1965	0	0	0	0	0	0	0	0	0	0	0	0	0	0	0	0	0	0	1	0	0	0	0	0	0	0	0	0	0	0	Fungi; Ascomycota; Eurotiomycetes; Onygenales; unidentified; unidentified; Onygenales_sp
New.ReferenceOTU2902	0	0	0	0	0	0	0	0	0	0	0	0	0	1	0	0	0	0	0	0	0	0	0	0	0	0	0	4	0	0	Fungi; Ascomycota; Eurotiomycetes; Onygenales; unidentified; unidentified; Onygenales_sp
New.ReferenceOTU5905	0	0	1	0	0	0	0	1	0	0	0	0	2	0	0	0	0	0	0	0	0	0	0	0	0	0	0	0	0	0	Fungi; Ascomycota; Eurotiomycetes; Onygenales; unidentified; unidentified; Onygenales_sp
SH109923.06FU_JX545204_reps	0	0	0	0	0	0	0	0	0	0	0	0	0	0	0	0	0	0	0	0	0	0	0	0	0	0	0	3	0	0	Fungi; Ascomycota; Eurotiomycetes; Onygenales; unidentified; unidentified; Onygenales_sp
New.ReferenceOTU117	1	0	0	0	0	0	0	0	0	0	0	0	0	0	0	0	0	3	0	2	0	0	0	0	0	0	0	0	0	0	Fungi; Ascomycota; Eurotiomycetes; Onygenales; unidentified; unidentified; uncultured_Emmonsia
New.ReferenceOTU1215	0	0	0	0	0	0	0	0	0	0	0	0	0	0	0	0	0	0	0	0	0	2	0	0	0	0	0	0	0	0	Fungi; Ascomycota; Eurotiomycetes; unidentified; unidentified; unidentified; Eurotiomycetes_sp
New.ReferenceOTU3442	0	0	0	0	1	0	0	0	0	0	0	0	0	0	0	0	0	0	0	0	0	0	0	0	0	0	0	0	0	0	Fungi; Ascomycota; Eurotiomycetes; unidentified; unidentified; unidentified; Eurotiomycetes_sp
New.ReferenceOTU4850	0	0	0	0	0	0	0	0	0	0	0	0	0	0	1	0	0	0	0	0	0	0	0	0	0	0	0	0	0	0	Fungi; Ascomycota; Eurotiomycetes; unidentified; unidentified; unidentified; Eurotiomycetes_sp_genotype_400
New.ReferenceOTU7063	0	0	0	0	0	0	0	0	0	0	0	0	0	0	1	0	0	0	0	0	0	0	0	0	0	0	0	0	0	0	Fungi; Ascomycota; Eurotiomycetes; unidentified; unidentified; unidentified; Eurotiomycetes_sp_genotype_400
New.ReferenceOTU1481	0	0	0	0	0	0	0	0	0	0	0	0	0	0	0	0	0	0	0	0	0	0	0	0	0	0	0	0	1	0	Fungi; Ascomycota; Incertae_sedis; Incertae_sedis; Incertae_sedis; Amplistroma; Amplistroma_erinaceum
New.ReferenceOTU3849	0	0	0	0	0	0	0	0	3	2	0	0	0	0	0	0	0	3	2	0	0	0	2	8	0	16	0	0	22	0	Fungi; Ascomycota; Incertae_sedis; Incertae_sedis; Incertae_sedis; Amplistroma; Amplistroma_erinaceum
New.ReferenceOTU4767	0	0	0	0	0	0	2	0	0	0	2	2	0	0	0	0	0	0	0	0	0	0	0	0	0	0	0	0	0	4	Fungi; Ascomycota; Incertae_sedis; Incertae_sedis; Incertae_sedis; Amplistroma; Amplistroma_erinaceum
New.ReferenceOTU5260	0	1	0	0	0	0	0	0	2	0	0	0	0	0	0	0	0	0	0	0	0	0	0	0	0	0	0	0	0	0	Fungi; Ascomycota; Incertae_sedis; Incertae_sedis; Incertae_sedis; Amplistroma; Amplistroma_erinaceum
New.ReferenceOTU5765	0	0	0	0	0	0	0	0	0	0	0	0	0	0	0	0	0	0	0	0	0	0	0	0	0	4	0	0	0	0	Fungi; Ascomycota; Incertae_sedis; Incertae_sedis; Incertae_sedis; Amplistroma; Amplistroma_erinaceum
New.ReferenceOTU6454	0	0	0	0	0	0	0	0	0	0	0	0	0	0	0	0	0	0	0	0	0	0	0	0	0	0	0	0	2	0	Fungi; Ascomycota; Incertae_sedis; Incertae_sedis; Incertae_sedis; Amplistroma; Amplistroma_erinaceum
New.ReferenceOTU7907	0	0	0	0	2	0	0	0	0	0	0	0	0	0	0	0	0	0	0	0	0	0	0	0	0	0	0	0	0	0	Fungi; Ascomycota; Incertae_sedis; Incertae_sedis; Incertae_sedis; Bactrodesmiastrum; Bactrodesmiastrum_obovatum
New.ReferenceOTU413	3	0	0	0	0	0	0	0	0	0	0	0	0	0	0	0	0	0	0	0	0	0	0	1	0	0	0	0	0	0	Fungi; Ascomycota; Incertae_sedis; Incertae_sedis; Incertae_sedis; Calcarisporiella; Calcarisporiella_sp
New.ReferenceOTU1253	0	0	0	0	0	0	0	0	0	0	1	0	0	0	0	0	0	0	0	0	0	0	0	0	0	0	0	0	0	0	Fungi; Ascomycota; Incertae_sedis; Incertae_sedis; Incertae_sedis; Eriosporella; Eriosporella_sp_DQD_2013
New.ReferenceOTU458	0	0	0	0	0	0	0	0	0	0	0	0	0	0	0	0	0	0	0	0	0	0	0	0	0	0	0	0	3	0	Fungi; Ascomycota; Incertae_sedis; Incertae_sedis; Incertae_sedis; Goidanichiella; Goidanichiella_sphaerospora
New.ReferenceOTU1958	0	0	0	0	0	0	0	0	0	0	0	0	0	1	0	0	0	0	0	0	0	0	0	0	0	0	0	0	0	0	Fungi; Ascomycota; Incertae_sedis; Incertae_sedis; Incertae_sedis; Heliocephala; Heliocephala_natarajanii
New.ReferenceOTU1316	2	0	2	0	1	0	0	2	0	6	2	0	0	1	0	4	0	0	0	2	0	2	0	24	0	1	0	0	3	0	Fungi; Ascomycota; Incertae_sedis; Incertae_sedis; Incertae_sedis; Kiliophora; Kiliophora_ubiensis
New.ReferenceOTU2022	0	0	0	0	0	0	0	0	0	0	0	0	0	1	0	0	0	0	0	0	0	0	0	0	0	0	0	0	0	0	Fungi; Ascomycota; Incertae_sedis; Incertae_sedis; Incertae_sedis; Kiliophora; Kiliophora_ubiensis
New.ReferenceOTU3541	0	0	0	0	0	0	0	0	0	0	0	0	0	0	0	0	0	10	0	0	0	0	0	0	0	0	0	0	0	0	Fungi; Ascomycota; Incertae_sedis; Incertae_sedis; Incertae_sedis; Myriodontium; Myriodontium_keratinophilum
SH024450.06FU_JN662486_reps_singleton	0	0	0	0	0	0	0	0	0	0	0	0	0	0	0	0	0	1	0	0	0	0	0	0	0	0	0	0	0	0	Fungi; Ascomycota; Incertae_sedis; Incertae_sedis; Incertae_sedis; Phialemoniopsis; Phialemoniopsis_ocularis
SH125937.06FU_HE599279_refs	0	0	0	0	0	0	0	0	0	0	0	0	0	0	0	1	0	0	0	0	0	0	0	0	0	0	0	0	0	0	Fungi; Ascomycota; Incertae_sedis; Incertae_sedis; Incertae_sedis; Phialemoniopsis; Phialemoniopsis_ocularis
New.CleanUp.ReferenceOTU1198	0	0	0	0	0	0	0	0	0	0	0	0	0	0	0	0	0	0	1	0	0	0	0	0	0	0	0	0	0	0	Fungi; Ascomycota; Incertae_sedis; Incertae_sedis; Incertae_sedis; Phialotubus; Phialotubus_microsporus
New.ReferenceOTU1280	1	6	15	7	0	1	0	0	0	6	0	0	0	2	0	1	1	4	0	1	0	0	0	1	0	3	0	4	1	0	Fungi; Ascomycota; Incertae_sedis; Incertae_sedis; Incertae_sedis; Phialotubus; Phialotubus_microsporus
New.ReferenceOTU3069	0	0	0	0	0	0	0	0	0	0	0	0	0	0	0	0	0	0	0	0	0	0	0	0	1	0	0	0	0	0	Fungi; Ascomycota; Incertae_sedis; Incertae_sedis; Incertae_sedis; Phialotubus; Phialotubus_microsporus
New.ReferenceOTU4234	0	0	0	0	2	0	0	0	0	0	0	0	0	0	0	0	0	0	0	0	0	0	0	0	0	0	0	0	0	0	Fungi; Ascomycota; Incertae_sedis; Incertae_sedis; Incertae_sedis; Phialotubus; Phialotubus_microsporus
New.ReferenceOTU1055	0	0	0	0	0	0	0	0	0	1	0	0	0	0	0	0	1	0	0	0	0	0	0	0	0	9	0	0	5	0	Fungi; Ascomycota; Incertae_sedis; Incertae_sedis; Incertae_sedis; Pseudolachnea; Pseudolachnea_fraxini
New.ReferenceOTU3095	0	0	0	0	0	0	0	0	0	0	0	0	0	0	0	1	0	0	0	0	0	0	0	0	0	0	0	0	0	0	Fungi; Ascomycota; Incertae_sedis; Incertae_sedis; Incertae_sedis; Pseudolachnea; Pseudolachnea_fraxini
New.ReferenceOTU6174	0	0	0	0	0	0	0	0	0	0	0	0	0	1	0	0	0	0	0	0	0	0	0	0	0	0	0	0	0	0	Fungi; Ascomycota; Incertae_sedis; Incertae_sedis; Incertae_sedis; Pseudolachnea; Pseudolachnea_fraxini
New.ReferenceOTU7700	0	0	0	0	0	0	0	0	0	0	0	0	0	0	0	0	0	0	0	0	0	0	0	0	0	0	0	0	0	2	Fungi; Ascomycota; Incertae_sedis; Incertae_sedis; Incertae_sedis; Pseudolachnea; Pseudolachnea_fraxini
New.ReferenceOTU3163	0	0	0	0	0	0	0	0	0	0	0	0	0	0	0	0	0	0	1	0	0	0	0	0	0	0	0	0	0	0	Fungi; Ascomycota; Incertae_sedis; Incertae_sedis; Incertae_sedis; Pseudorobillarda; Pseudorobillarda_texana
New.ReferenceOTU4233	0	0	0	0	0	0	0	0	0	0	0	0	0	0	0	0	0	0	0	0	0	0	2	0	0	0	0	0	0	0	Fungi; Ascomycota; Incertae_sedis; Incertae_sedis; Incertae_sedis; Pyrigemmula; Pyrigemmula_aurantiaca
New.CleanUp.ReferenceOTU751	0	0	0	0	0	0	2	0	0	0	0	0	0	0	0	0	0	0	0	0	0	0	0	0	0	0	0	0	0	0	Fungi; Ascomycota; Incertae_sedis; Incertae_sedis; Incertae_sedis; Retroconis; Retroconis_fusiformis
New.ReferenceOTU2294	0	0	0	0	0	0	3	0	1	0	0	0	0	0	0	0	1	0	0	0	1	0	0	0	0	0	0	1	0	1	Fungi; Ascomycota; Incertae_sedis; Incertae_sedis; Incertae_sedis; Retroconis; Retroconis_fusiformis
SH129065.06FU_FJ752621_reps	0	2	2	0	0	0	0	1	0	0	0	0	1	2	1	0	0	0	0	0	1	0	1	0	0	0	0	0	0	0	Fungi; Ascomycota; Incertae_sedis; Incertae_sedis; Incertae_sedis; unidentified; Ascomycota_sp
New.ReferenceOTU4917	0	0	0	0	0	0	0	0	0	0	0	0	0	0	0	0	0	0	0	0	0	0	1	0	0	0	0	0	0	0	Fungi; Ascomycota; Lecanoromycetes; Ostropales; unidentified; unidentified; Ostropales_sp
New.ReferenceOTU6851	0	0	0	0	0	0	0	0	0	0	0	0	0	0	0	0	0	0	0	0	0	0	0	0	0	0	0	2	0	0	Fungi; Ascomycota; Lecanoromycetes; Ostropales; unidentified; unidentified; Ostropales_sp
New.ReferenceOTU2431	0	0	0	0	0	0	0	0	0	0	0	0	0	0	0	0	0	0	0	0	0	0	0	0	0	0	8	0	0	0	Fungi; Ascomycota; Lecanoromycetes; unidentified; unidentified; unidentified; Lecanoromycetes_sp
New.ReferenceOTU4619	0	0	0	0	0	0	0	0	0	4	8	0	1	9	0	2	3	0	0	0	0	0	0	5	0	0	0	0	0	0	Fungi; Ascomycota; Lecanoromycetes; unidentified; unidentified; unidentified; Lecanoromycetes_sp
New.ReferenceOTU6306	0	0	0	0	0	4	0	0	0	0	0	0	0	0	0	0	0	0	0	0	0	0	0	0	0	0	0	0	0	0	Fungi; Ascomycota; Lecanoromycetes; unidentified; unidentified; unidentified; Lecanoromycetes_sp
New.ReferenceOTU6522	0	0	0	0	0	0	0	0	0	0	0	0	0	0	0	0	0	1	0	0	0	0	0	0	0	0	0	0	0	0	Fungi; Ascomycota; Lecanoromycetes; unidentified; unidentified; unidentified; Lecanoromycetes_sp
New.ReferenceOTU7528	0	0	0	0	0	0	0	0	2	0	0	0	0	0	0	0	0	0	0	0	0	0	0	0	0	0	0	0	0	0	Fungi; Ascomycota; Lecanoromycetes; unidentified; unidentified; unidentified; Lecanoromycetes_sp
New.ReferenceOTU5088	0	0	0	0	0	0	0	0	0	0	0	0	0	0	0	0	0	0	0	1	0	0	0	0	0	0	0	0	0	0	Fungi; Ascomycota; Leotiomycetes; Geoglossales; unidentified; unidentified; Geoglossales_sp
New.ReferenceOTU4837	0	0	0	0	0	0	0	0	0	0	1	0	0	0	0	0	0	0	0	1	0	0	0	0	0	0	0	0	0	0	Fungi; Ascomycota; Leotiomycetes; Helotiales; Dermateaceae; unidentified; Dermateaceae_sp
New.ReferenceOTU5829	0	0	0	0	0	0	0	0	1	0	0	0	0	0	0	0	0	0	0	0	0	0	0	0	0	0	0	0	0	0	Fungi; Ascomycota; Leotiomycetes; Helotiales; Dermateaceae; unidentified; Dermateaceae_sp
New.ReferenceOTU3648	0	0	1	0	0	0	0	0	0	0	0	0	0	0	0	0	0	0	0	0	0	0	0	0	0	0	0	0	0	0	Fungi; Ascomycota; Leotiomycetes; Helotiales; Dermateaceae; unidentified; uncultured_Cryptosporiopsis
New.ReferenceOTU2874	0	1	0	0	0	0	0	0	0	0	0	0	0	0	0	0	0	0	0	0	0	0	0	0	0	2	0	0	35	0	Fungi; Ascomycota; Leotiomycetes; Helotiales; Dermateaceae; unidentified; uncultured_Dermateaceae
SH116620.06FU_FJ475732_refs	0	0	0	0	0	0	0	0	0	0	0	0	0	0	0	0	0	0	0	0	0	0	0	0	0	5	0	0	12	0	Fungi; Ascomycota; Leotiomycetes; Helotiales; Dermateaceae; unidentified; uncultured_Dermateaceae
SH116654.06FU_JX857178_reps	0	0	0	0	0	0	0	0	0	0	0	0	0	0	0	0	0	0	0	0	0	0	0	0	0	0	0	0	1	0	Fungi; Ascomycota; Leotiomycetes; Helotiales; Dermateaceae; unidentified; uncultured_Dermateaceae
SH139174.06FU_JF424305_reps	0	0	0	0	0	0	0	0	0	0	0	0	0	0	0	0	0	0	0	0	0	0	0	0	0	0	0	0	1	0	Fungi; Ascomycota; Leotiomycetes; Helotiales; Incertae_sedis; unidentified; Helotiales_sp

New.ReferenceOTU1108	0	0	0	0	0	0	0	0	0	0	0	0	0	0	0	0	0	0	0	0	0	0	0	0	0	0	0	0	1	0	Fungi; Ascomycota; Leotiomycetes; Helotiales; Sclerotiniaceae; unidentified; Sclerotiniaceae_sp
New.ReferenceOTU6014	0	0	0	0	0	0	0	0	0	1	1	0	0	0	0	0	0	0	0	0	0	0	0	0	0	0	0	0	0	0	Fungi; Ascomycota; Leotiomycetes; Helotiales; Sclerotiniaceae; unidentified; Sclerotiniaceae_sp
New.ReferenceOTU7468	0	0	0	0	0	0	0	0	0	0	0	0	0	0	0	0	0	0	0	0	0	0	1	0	0	0	0	0	0	0	Fungi; Ascomycota; Leotiomycetes; Helotiales; Sclerotiniaceae; unidentified; Sclerotiniaceae_sp
New.ReferenceOTU1362	0	0	0	0	0	0	0	0	0	0	0	0	0	0	0	0	0	0	1	0	0	0	0	0	0	0	0	0	0	0	Fungi; Ascomycota; Leotiomycetes; Helotiales; Sclerotiniaceae; unidentified; uncultured_Monilinia
New.ReferenceOTU1470	0	0	0	0	0	0	0	0	0	2	0	0	6	3	0	0	0	0	2	0	0	0	2	2	0	0	0	0	0	0	Fungi; Ascomycota; Leotiomycetes; Helotiales; unidentified; unidentified; Helotiales_sp
New.ReferenceOTU160	0	0	0	0	0	0	0	0	0	0	0	0	1	0	0	0	0	0	0	0	0	0	0	0	0	0	0	0	0	0	Fungi; Ascomycota; Leotiomycetes; Helotiales; unidentified; unidentified; Helotiales_sp
New.ReferenceOTU1845	0	0	0	0	0	0	0	0	1	0	0	0	0	0	0	0	0	0	0	0	0	0	1	0	0	0	0	2	0	0	Fungi; Ascomycota; Leotiomycetes; Helotiales; unidentified; unidentified; Helotiales_sp
New.ReferenceOTU2161	2	0	0	0	0	0	0	0	0	0	0	0	0	0	0	0	0	0	0	0	0	0	0	1	0	0	0	0	0	0	Fungi; Ascomycota; Leotiomycetes; Helotiales; unidentified; unidentified; Helotiales_sp
New.ReferenceOTU2535	0	0	0	0	0	0	0	0	0	0	0	0	0	0	0	1	0	0	0	0	0	0	1	0	0	0	0	0	0	4	Fungi; Ascomycota; Leotiomycetes; Helotiales; unidentified; unidentified; Helotiales_sp
New.ReferenceOTU2827	0	0	0	0	4	0	0	0	0	0	0	0	0	0	0	0	0	0	0	0	0	0	0	0	0	0	0	0	0	0	Fungi; Ascomycota; Leotiomycetes; Helotiales; unidentified; unidentified; Helotiales_sp
New.ReferenceOTU2951	0	0	1	3	5	0	0	0	0	0	2	0	6	0	0	0	0	0	0	0	0	0	0	0	0	0	0	0	1	0	Fungi; Ascomycota; Leotiomycetes; Helotiales; unidentified; unidentified; Helotiales_sp
New.ReferenceOTU3314	0	0	0	0	3	0	0	0	3	2	0	0	1	1	1	1	1	1	0	1	0	0	0	1	1	0	0	0	0	1	Fungi; Ascomycota; Leotiomycetes; Helotiales; unidentified; unidentified; Helotiales_sp
New.ReferenceOTU420	0	0	0	0	0	0	0	0	0	0	0	0	0	0	0	0	1	0	0	0	0	0	0	0	0	0	0	0	0	0	Fungi; Ascomycota; Leotiomycetes; Helotiales; unidentified; unidentified; Helotiales_sp
New.ReferenceOTU4421	0	0	0	2	0	0	0	0	0	0	0	0	0	0	0	0	0	0	0	0	0	0	0	0	0	0	0	0	0	0	Fungi; Ascomycota; Leotiomycetes; Helotiales; unidentified; unidentified; Helotiales_sp
New.ReferenceOTU4612	0	0	0	0	2	0	0	0	0	0	0	0	0	0	0	0	0	0	0	0	0	0	0	0	0	0	0	0	0	0	Fungi; Ascomycota; Leotiomycetes; Helotiales; unidentified; unidentified; Helotiales_sp
New.ReferenceOTU4729	0	0	0	0	0	0	0	2	0	0	0	0	0	0	0	0	0	0	0	0	0	0	0	0	0	0	0	0	0	0	Fungi; Ascomycota; Leotiomycetes; Helotiales; unidentified; unidentified; Helotiales_sp
New.ReferenceOTU4909	0	0	0	0	0	0	0	0	0	0	0	0	0	0	0	0	0	0	0	0	0	0	1	0	0	0	0	0	0	0	Fungi; Ascomycota; Leotiomycetes; Helotiales; unidentified; unidentified; Helotiales_sp
New.ReferenceOTU5031	0	0	2	0	0	0	0	0	0	0	7	0	0	0	0	0	0	0	0	0	0	0	0	0	0	0	0	0	0	0	Fungi; Ascomycota; Leotiomycetes; Helotiales; unidentified; unidentified; Helotiales_sp
New.ReferenceOTU513	0	0	0	0	2	0	0	0	2	0	0	0	0	0	0	0	0	0	0	0	0	0	0	0	0	0	0	0	0	0	Fungi; Ascomycota; Leotiomycetes; Helotiales; unidentified; unidentified; Helotiales_sp
New.ReferenceOTU5143	0	0	0	0	0	0	0	0	0	0	0	0	0	0	0	0	0	0	1	0	0	0	0	0	0	0	0	0	0	0	Fungi; Ascomycota; Leotiomycetes; Helotiales; unidentified; unidentified; Helotiales_sp
New.ReferenceOTU5197	2	0	0	0	0	0	0	0	0	0	0	0	0	0	0	0	0	0	0	0	0	0	0	0	0	0	0	0	0	0	Fungi; Ascomycota; Leotiomycetes; Helotiales; unidentified; unidentified; Helotiales_sp
New.ReferenceOTU5612	0	0	0	1	0	0	0	3	0	0	0	0	0	0	0	0	0	0	0	0	0	0	0	0	0	0	0	0	0	0	Fungi; Ascomycota; Leotiomycetes; Helotiales; unidentified; unidentified; Helotiales_sp
New.ReferenceOTU577	0	7	0	0	0	0	0	0	0	0	0	2	0	0	0	0	0	0	0	0	0	0	0	0	0	0	0	0	0	0	Fungi; Ascomycota; Leotiomycetes; Helotiales; unidentified; unidentified; Helotiales_sp
New.ReferenceOTU5826	0	0	0	0	1	0	0	0	0	0	0	0	0	0	0	0	0	0	0	0	0	0	0	0	0	0	0	0	0	0	Fungi; Ascomycota; Leotiomycetes; Helotiales; unidentified; unidentified; Helotiales_sp
New.ReferenceOTU7047	0	0	0	0	0	0	0	0	0	0	0	0	0	0	1	0	0	0	0	0	0	0	0	0	0	0	0	0	0	0	Fungi; Ascomycota; Leotiomycetes; Helotiales; unidentified; unidentified; Helotiales_sp
New.ReferenceOTU7358	0	0	0	0	0	0	0	0	0	0	0	0	0	0	0	0	0	0	0	0	0	0	0	10	0	0	0	0	0	0	Fungi; Ascomycota; Leotiomycetes; Helotiales; unidentified; unidentified; Helotiales_sp
New.ReferenceOTU7970	0	0	0	0	2	0	0	0	0	0	0	0	0	0	0	0	0	0	0	0	0	0	0	0	0	0	0	0	0	0	Fungi; Ascomycota; Leotiomycetes; Helotiales; unidentified; unidentified; Helotiales_sp
New.ReferenceOTU8045	0	0	0	0	0	0	0	0	0	0	0	0	0	0	0	0	0	0	0	0	0	0	0	0	0	0	0	0	0	4	Fungi; Ascomycota; Leotiomycetes; Helotiales; unidentified; unidentified; Helotiales_sp
SH014167.06FU_AY268187_reps_singleton	0	0	0	0	0	0	0	0	0	4	0	0	0	0	0	0	0	0	0	0	0	0	0	0	0	0	0	0	0	0	Fungi; Ascomycota; Leotiomycetes; Helotiales; unidentified; unidentified; Helotiales_sp
SH014225.06FU_GQ268560_reps_singleton	1	0	0	0	0	0	0	0	0	4	0	0	1	1	0	0	1	0	0	0	0	0	0	1	0	0	0	0	0	0	Fungi; Ascomycota; Leotiomycetes; Helotiales; unidentified; unidentified; Helotiales_sp
SH028472.06FU_HM770996_reps_singleton	0	0	0	0	0	0	0	0	3	0	0	0	0	0	0	0	0	0	0	0	0	0	0	0	0	0	0	0	0	0	Fungi; Ascomycota; Leotiomycetes; Helotiales; unidentified; unidentified; Helotiales_sp
SH095625.06FU_JQ666638_reps	0	0	0	0	0	0	0	0	0	0	0	0	0	0	0	0	0	6	0	8	0	0	0	0	0	0	0	0	0	0	Fungi; Ascomycota; Leotiomycetes; Helotiales; unidentified; unidentified; Helotiales_sp
SH108740.06FU_HM230869_reps	0	0	0	0	0	0	0	0	0	0	0	0	0	1	0	0	0	0	0	0	0	0	0	0	0	0	0	0	0	6	Fungi; Ascomycota; Leotiomycetes; Helotiales; unidentified; unidentified; Helotiales_sp
SH108780.06FU_AY969797_reps	0	0	0	0	0	0	0	0	0	0	0	1	0	0	0	0	0	0	0	0	0	0	0	0	0	0	0	0	0	0	Fungi; Ascomycota; Leotiomycetes; Helotiales; unidentified; unidentified; Helotiales_sp
New.ReferenceOTU2510	0	14	0	0	0	0	0	0	0	0	0	0	0	0	0	0	1	0	1	0	0	0	0	1	0	0	0	0	9	0	Fungi; Ascomycota; Leotiomycetes; Helotiales; unidentified; unidentified; Helotiales_sp_38_14_1
New.ReferenceOTU765	0	0	0	0	0	0	0	0	0	0	0	0	0	0	0	0	0	0	0	0	0	0	0	1	0	0	0	0	0	0	Fungi; Ascomycota; Leotiomycetes; Helotiales; unidentified; unidentified; Helotiales_sp_r411
New.CleanUp.ReferenceOTU450	0	0	0	0	0	0	0	0	0	0	0	0	0	0	0	0	0	1	0	0	0	0	0	0	0	0	0	0	0	0	Fungi; Ascomycota; Leotiomycetes; Helotiales; unidentified; unidentified; uncultured_Helotiales
New.ReferenceOTU1292	0	0	0	0	0	0	0	0	0	0	3	0	0	0	0	0	0	0	0	0	0	0	0	0	0	0	0	0	0	0	Fungi; Ascomycota; Leotiomycetes; Helotiales; unidentified; unidentified; uncultured_Helotiales
New.ReferenceOTU3417	6	0	0	0	0	0	0	0	1	0	1	0	0	0	0	0	0	0	0	0	0	0	0	0	0	0	0	0	1	0	Fungi; Ascomycota; Leotiomycetes; Helotiales; unidentified; unidentified; uncultured_Helotiales
New.ReferenceOTU860	0	0	0	0	0	0	0	0	0	0	0	0	0	0	0	0	0	0	0	0	0	0	0	0	0	1	0	0	0	0	Fungi; Ascomycota; Leotiomycetes; Helotiales; unidentified; unidentified; uncultured_Helotiales
SH018524.06FU_JN847501_reps_singleton	0	0	0	0	0	0	0	0	1	0	0	0	0	0	0	0	0	0	0	0	0	0	0	0	0	0	0	0	0	0	Fungi; Ascomycota; Leotiomycetes; Helotiales; unidentified; unidentified; uncultured_Helotiales
SH116635.06FU_AF097312_refs	0	0	0	0	0	0	0	0	0	0	0	0	0	0	0	0	0	0	1	0	0	0	0	0	0	0	0	0	0	0	Fungi; Ascomycota; Leotiomycetes; Helotiales; unidentified; unidentified; uncultured_Helotiales
New.ReferenceOTU6216	0	0	2	0	0	0	0	0	0	0	0	0	5	0	0	2	0	0	0	0	0	0	0	0	0	0	0	0	0	0	Fungi; Ascomycota; Leotiomycetes; Helotiales; unidentified; unidentified; uncultured_Hyaloscyphaceae
SH018553.06FU_JX317338_reps_singleton	0	0	3	0	0	0	0	0	0	0	0	0	16	0	0	10	0	0	0	0	0	0	0	0	0	0	0	0	0	0	Fungi; Ascomycota; Leotiomycetes; Helotiales; unidentified; unidentified; uncultured_Hyaloscyphaceae
New.ReferenceOTU2508	0	0	0	0	0	0	0	0	0	1	0	0	0	0	0	0	0	0	0	0	0	0	0	0	0	0	0	0	0	0	Fungi; Ascomycota; Leotiomycetes; Leotiales; Leotiaceae; unidentified; Leotiaceae_sp
New.ReferenceOTU3394	0	0	0	0	0	0	0	0	0	0	0	0	0	0	0	0	0	0	0	0	0	0	1	0	0	0	0	0	0	0	Fungi; Ascomycota; Leotiomycetes; Leotiales; Leotiaceae; unidentified; Leotiaceae_sp
New.ReferenceOTU4303	0	0	0	0	0	0	0	0	1	0	0	0	0	0	0	0	2	0	0	0	0	0	0	0	0	0	0	0	0	0	Fungi; Ascomycota; Leotiomycetes; Leotiales; Leotiaceae; unidentified; Leotiaceae_sp
New.ReferenceOTU4338	0	0	0	0	0	0	0	0	0	0	0	0	0	0	0	0	0	0	0	0	0	0	0	0	0	0	0	0	0	1	Fungi; Ascomycota; Leotiomycetes; Leotiales; Leotiaceae; unidentified; Leotiaceae_sp
New.ReferenceOTU4389	0	0	0	0	1	0	0	0	0	0	0	0	0	0	0	0	0	0	0	0	0	0	0	1	0	0	0	0	0	0	Fungi; Ascomycota; Leotiomycetes; Leotiales; Leotiaceae; unidentified; Leotiaceae_sp
New.ReferenceOTU6057	0	0	0	0	0	0	0	0	0	0	0	1	2	0	0	0	0	0	0	0	0	0	0	0	0	0	0	0	0	0	Fungi; Ascomycota; Leotiomycetes; Leotiales; Leotiaceae; unidentified; Leotiaceae_sp
New.ReferenceOTU6182	0	0	0	0	0	3	0	0	0	0	0	0	0	0	0	0	0	0	0	0	0	0	0	0	0	0	0	0	0	0	Fungi; Ascomycota; Leotiomycetes; Leotiales; Leotiaceae; unidentified; Leotiaceae_sp
New.ReferenceOTU6766	0	0	0	0	1	0	0	0	0	0	0	0	0	0	0	0	0	0	0	0	0	0	0	0	0	0	0	0	0	0	Fungi; Ascomycota; Leotiomycetes; Leotiales; Leotiaceae; unidentified; Leotiaceae_sp
New.ReferenceOTU6959	0	0	0	0	0	0	0	0	0	1	0	0	0	0	0	0	0	0	0	0	0	0	0	0	0	0	0	0	0	0	Fungi; Ascomycota; Leotiomycetes; Leotiales; Leotiaceae; unidentified; Leotiaceae_sp
New.ReferenceOTU1925	0	0	0	0	0	6	0	0	0	0	0	1	16	0	0	0	0	0	0	0	0	0	0	0	0	0	0	0	0	0	Fungi; Ascomycota; Leotiomycetes; unidentified; unidentified; unidentified; Leotiomycetes_sp
New.ReferenceOTU3802	0	0	0	0	0	0	0	0	2	0	0	0	0	0	0	0	0	0	0	0	0	0	0	0	0	0	0	0	0	0	Fungi; Ascomycota; Leotiomycetes; unidentified; unidentified; unidentified; Leotiomycetes_sp
New.ReferenceOTU7169	0	0	0	0	0	0	1	0	0	0	0	0	0	0	0	0	0	0	0	0	0	0	0	0	0	0	0	0	0	0	Fungi; Ascomycota; Leotiomycetes; unidentified; unidentified; unidentified; Leotiomycetes_sp
New.ReferenceOTU893	0	0	1	0	0	0	0	0	0	0	0	0	0	0	0	0	0	0	0	0	0	0	0	0	0	0	0	0	0	0	Fungi; Ascomycota; Leotiomycetes; unidentified; unidentified; unidentified; Leotiomycetes_sp
New.ReferenceOTU6254	0	0	0	0	0	0	0	0	0	0	0	0	0	0	0	0	0	0	0	0	0	0	1	0	0	0	16	0	0	0	Fungi; Ascomycota; Leotiomycetes; unidentified; unidentified; unidentified; uncultured_Leotiomycetes
New.ReferenceOTU6971	0	0	0	0	0	0	0	0	0	0	0	0	0	0	0	0	0	0	0	0	0	0	0	0	0	0	0	0	0	1	Fungi; Ascomycota; Leotiomycetes; unidentified; unidentified; unidentified; uncultured_Leotiomycetes
New.ReferenceOTU4199	1	0	0	0	0	0	0	0	0	0	0	0	0	0	0	0	0	0	0	0	0	0	0	0	0	0	0	0	0	0	Fungi; Ascomycota; Orbiliomycetes; Orbiliales; Orbiliaceae; unidentified; Orbiliaceae_sp
New.ReferenceOTU5050	0	0	0	0	0	0	0	0	0	0	0	0	0	0	0	0	1	0	0	0	0	0	0	0	0	0	0	0	0	0	Fungi; Ascomycota; Orbiliomycetes; Orbiliales; Orbiliaceae; unidentified; Orbiliaceae_sp
New.ReferenceOTU1791	2	0	0	0	0	0	0	0	0	0	0	0	0	0	0	0	0	0	0	0	0	0	0	0	0	0	0	0	1	0	Fungi; Ascomycota; Orbiliomycetes; unidentified; unidentified; unidentified; Orbiliomycetes_sp
New.ReferenceOTU425	0	0	0	0	0	0	0	0	0	0	0	0	0	0	0	0	0	0	1	0	0	0	0	0	0	0	0	0	0	0	Fungi; Ascomycota; Orbiliomycetes; unidentified; unidentified; unidentified; Orbiliomycetes_sp
New.ReferenceOTU5830	0	0	0	0	0	0	0	0	0	0	0	0	0	0	0	0	0	0	0	0	0	1	0	0	0	0	0	0	0	0	Fungi; Ascomycota; Orbiliomycetes; unidentified; unidentified; unidentified; Orbiliomycetes_sp
New.ReferenceOTU886	0	0	0	0	0	0	0	0	0	0	0	0	0	3	0	0	0	0	0	0	0	0	0	0	0	0	0	0	0	0	Fungi; Ascomycota; Pezizomycetes; Pezizales; Pezizaceae; unidentified; Pezizaceae_sp
New.ReferenceOTU2500	0	0	1	0	0	0	0	0	0	0	0	0	0	0	0	0	0	0	0	0	0	0	0	0	0	0	0	0	0	0	Fungi; Ascomycota; Pezizomycetes; Pezizales; Pezizaceae; unidentified; Pezizaceae_sp_DMH_2010
New.ReferenceOTU7159	0	0	0	0	0	0	0	0	0	0	0	0	0	0	0	0	0	0	0	0	0	0	0	0	0	0	0	0	0	1	Fungi; Ascomycota; Pezizomycetes; Pezizales; Pezizaceae; unidentified; Pezizaceae_sp_DMH_2010
New.ReferenceOTU3819	0	0	0	0	1	0	0	0	0	0	0	0	0	0	0	0	0	0	0	0	0	0	0	0	0	0	0	0	0	0	Fungi; Ascomycota; Pezizomycetes; Pezizales; Pezizaceae; unidentified; uncultured_Peziza
New.ReferenceOTU1360	0	0	0	0	0	0	0	0	0	0	1	0	0	0	1	0	0	0	0	0	0	0	0	0	0	0	0	0	0	0	Fungi; Ascomycota; Pezizomycetes; Pezizales; Pyronemataceae; unidentified; Pyronemataceae_sp
New.ReferenceOTU1449	1	0	1	2	5	1	4	0	0	0	0	3	2	3	2	0	2	0	0	0	0	2	1	0	0	6	3	0	0	0	Fungi; Ascomycota; Pezizomycetes; Pezizales; Pyronemataceae; unidentified; Pyronemataceae_sp
New.ReferenceOTU1656	0	0	0	1	0	0	0	0	0	3	0	0	0	0	0	0	1	0	0	0	0	0	0	0	5	0	0	0	0	0	Fungi; Ascomycota; Pezizomycetes; Pezizales; Pyronemataceae; unidentified; Pyronemataceae_sp
New.ReferenceOTU2178	0	0	0	0	3	0	0	0	0	0	0	0	0	0	0	0	0	0	0	0	0	0	0	0	0	0	0	0	0	0	Fungi; Ascomycota; Pezizomycetes; Pezizales; Pyronemataceae; unidentified; Pyronemataceae_sp
New.ReferenceOTU3837	1	0	0	0	0	0	0	0	0	0	0	0	0	0	1	0	0	0	0	0	0	0	0	1	0	0	0	0	0	0	Fungi; Ascomycota; Pezizomycetes; Pezizales; Pyronemataceae; unidentified; Pyronemataceae_sp
New.ReferenceOTU4216	0	0	0	0	0	0	1	0	0	0	0	0	0	0	0	0	0	0	0	0	0	0	0	0	0	0	0	0	0	0	Fungi; Ascomycota; Pezizomycetes; Pezizales; Pyronemataceae; unidentified; Pyronemataceae_sp
New.ReferenceOTU4434	0	0	0	0	0	0	0	0	0	0	0	0	1	0	0	0	0	0	0	0	0	0	0	0	0	0	0	0	0	0	Fungi; Ascomycota; Pezizomycetes; Pezizales; Pyronemataceae; unidentified; Pyronemataceae_sp
New.ReferenceOTU500	0	0	0	1	0	0	0	0	0	0	0	0	0	3	0	0	0	0	0	0	0	0	0	0	0	0	1	0	0	0	Fungi; Ascomycota; Pezizomycetes; Pezizales; Pyronemataceae; unidentified; Pyronemataceae_sp
New.ReferenceOTU5449	5	0	0	0	5	0	0	1	0	6	1	0	4	1	0	0	0	0	0	0	0	0	1	1	2	0	0	0	0	1	Fungi; Ascomycota; Pezizomycetes; Pezizales; Pyronemataceae; unidentified; Pyronemataceae_sp
New.ReferenceOTU6286	0	1	0	0	0	0	0	0	0	0	0	0	0	0	0	0	0	0	0	0	0	0	0	0	0	0	0	0	0	0	Fungi; Ascomycota; Pezizomycetes; Pezizales; Pyronemataceae; unidentified; Pyronemataceae_sp
New.ReferenceOTU6344	0	0	0	0	0	0	0	0	0	0	0	0	0	0	0	0	1	0	0	0	0	0	0	0	0	0	0	0	0	0	Fungi; Ascomycota; Pezizomycetes; Pezizales; Pyronemataceae; unidentified; Pyronemataceae_sp
New.ReferenceOTU4778	0	0	0	0	0	0	0	0	0	1	0	0	0	0	0	0	0	0	0	0	0	0	0	0	0	0	0	0	0	0	Fungi; Ascomycota; Pezizomycetes; unidentified; unidentified; unidentified; Pezizomycetes_sp
New.ReferenceOTU1149	0	0	0	0	0	0	0	4	0	0	0	0	0	0	0	0	0	0	0	0	0	0	0	0	0	0	0	0	0	0	Fungi; Ascomycota; Sordariomycetes; Boliniales; Boliniaceae; unidentified; Boliniaceae_sp
New.ReferenceOTU2029	0	0	0	0	0	0	0	0	0	0	0	0	1	0	0	0	0	0	0	0	0	0	0	0	0	0	0	0	0	0	Fungi; Ascomycota; Sordariomycetes; Boliniales; Boliniaceae; unidentified; Boliniaceae_sp
New.ReferenceOTU2634	0	0	0	0	0	0	0	0	0	0	0	0	0	0	0	0	0	0	0	0	0	0	0	0	0	0	0	0	2	0	Fungi; Ascomycota; Sordariomycetes; Boliniales; Boliniaceae; unidentified; Boliniaceae_sp
New.ReferenceOTU4734	0	0	0	0	0	2	0	0	0	1	1	0	4	0	0	1	0	0	0	0	1	0	0	2	3	0	0	0	1	0	Fungi; Ascomycota; Sordariomycetes; Boliniales; Boliniaceae; unidentified; Boliniaceae_sp
New.ReferenceOTU655	0	0	0	0	0	0	0	73	0	0	0	0	0	0	0	0	0	0	0	0	0	0	0	0	0	0	0	0	0	0	Fungi; Ascomycota; Sordariomycetes; Boliniales; Boliniaceae; unidentified; Boliniaceae_sp
New.ReferenceOTU7227	0	0	0	0	1	0	0	0	0	0	0	0	0	0	0	0	0	0	0	0	0	0	0	0	0	0	0	0	0	0	Fungi; Ascomycota; Sordariomycetes; Boliniales; Boliniaceae; unidentified; Boliniaceae_sp
New.ReferenceOTU321	0	0	0	0	0	0	1	0	0	2	0	0	0	0	0	0	0	0	1	0	0	0	0	0	0	0	0	0	0	0	Fungi; Ascomycota; Sordariomycetes; Boliniales; unidentified; unidentified; Boliniales_sp_108_PH
New.ReferenceOTU4722	0	0	0	3	0	3	0	0	0	0	0	0	0	0	0	0	0	0	0	1	0	0	0	0	0	0	0	2	0	0	Fungi; Ascomycota; Sordariomycetes; Boliniales; unidentified; unidentified; Boliniales_sp_108_PH
New.ReferenceOTU5565	3	0	0	13	2	0	1	0	0	0	0	0	0	2	0	0	1	0	0	0	0	0	0	0	0	0	0	0	0	0	Fungi; Ascomycota; Sordariomycetes; Boliniales; unidentified; unidentified; Boliniales_sp_108_PH
New.ReferenceOTU1384	2	0	0	0	1	0	0	0	0	0	0	2	0	0	0	0	0	0	0	1	0	0	0	0	0	0	0	0	0	0	Fungi; Ascomycota; Sordariomycetes; Chaetosphaeriales; Chaetosphaeriaceae; unidentified; Chaetosphaeriaceae_sp
New.ReferenceOTU4745	0	0	0	0	0	0	0	0	0	0	0	4	0	0	0	0	0	0	0	0	0	0	0	0	0	0	0	0	0	0	Fungi; Ascomycota; Sordariomycetes; Chaetosphaeriales; Chaetosphaeriaceae; unidentified; Chaetosphaeriaceae_sp
New.ReferenceOTU5471	0	0	0	0	1	0	0	0	0	0	0	0	0	0	0	0	0	0	0	0	0	0	0	0	0	0	0	0	0	0	Fungi; Ascomycota; Sordariomycetes; Chaetosphaeriales; Chaetosphaeriaceae; unidentified; Chaetosphaeriaceae_sp
New.ReferenceOTU6153	0	0	0	0	0	0	1	0	0	0	0	0	0	0	0	0	0	0	0	0	0	0	0	0	0	0	0	0	0	0	Fungi; Ascomycota; Sordariomycetes; Chaetosphaeriales; Chaetosphaeriaceae; unidentified; Chaetosphaeriaceae_sp
SH032914.06FU_EU754937_reps_singleton	0	0	0	0	0	0	0	0	0	0	0	0	0	0	0	0	0	0	0	0	0	0	0	0	1	0	0	0	0	0	Fungi; Ascomycota; Sordariomycetes; Chaetosphaeriales; Chaetosphaeriaceae; unidentified; Chaetosphaeriaceae_sp
SH138842.06FU_AY699658_reps	3	6	0	0	19	1	0	1	0	0	4	1	1	0	0	1	0	0	0	0	0	0	0	2	0	2	0	0	16	0	Fungi; Ascomycota; Sordariomycetes; Chaetosphaeriales; Chaetosphaeriaceae; unidentified; Chaetosphaeriaceae_sp
SH138844.06FU_HQ611333_reps	0	0	0	0	0	0	0	0	0	0	0	0	0	0	0	0	0	0	0	0	0	0	0	0	0	0	1	0	0	0	Fungi; Ascomycota; Sordariomycetes; Chaetosphaeriales; Chaetosphaeriaceae; unidentified; Chaetosphaeriaceae_sp
New.ReferenceOTU3006	0	0	1	2	0	0	0	0	0	0	0	0	0	0	0	0	0	0	0	0	0	0	0	0	0	0	0	0	0	0	Fungi; Ascomycota; Sordariomycetes; Coniochaetales; Incertae_sedis; Wallrothiella; Wallrothiella_sp
SH121365.06FU_EU687048_reps	0	0	0	1	0	0	0	0	1	0	0	0	0	0	0	0	0	0	0	1	0	0	0	0	0	0	0	0	0	0	Fungi; Ascomycota; Sordariomycetes; Coniochaetales; Incertae_sedis; Wallrothiella; Wallrothiella_sp
New.ReferenceOTU1694	0	0	0	1	0	0	0	0	0	0	0	0	0	0	0	0	0	0	0	0	0	0	0	0	0	0	0	0	0	0	Fungi; Ascomycota; Sordariomycetes; Coniochaetales; unidentified; unidentified; Coniochaetales_sp
New.ReferenceOTU3742	0	0	0	0	0	0	0	0	0	0	0	0	0	0	0	0	0	0	8	7	0	0	0	1	0	0	0	0	0	0	Fungi; Ascomycota; Sordariomycetes; Coniochaetales; unidentified; unidentified; Coniochaetales_sp

New.ReferenceOTU5162	1	0	0	0	0	0	0	0	0	0	0	0	0	0	0	0	0	0	0	0	0	0	0	1	0	0	0	0	0	0	Fungi; Ascomycota; Sordariomycetes; Coniochaetales; unidentified; unidentified; Coniochaetales_sp
New.ReferenceOTU783	0	0	0	0	0	0	0	0	0	0	0	0	0	0	0	0	0	0	0	0	0	0	1	0	0	0	0	0	0	0	Fungi; Ascomycota; Sordariomycetes; Coniochaetales; unidentified; unidentified; Coniochaetales_sp
New.ReferenceOTU7836	0	0	0	0	0	0	0	0	0	0	0	0	0	0	0	0	0	0	0	0	0	0	1	0	3	0	0	0	0	0	Fungi; Ascomycota; Sordariomycetes; Coniochaetales; unidentified; unidentified; Coniochaetales_sp
New.ReferenceOTU2181	0	0	0	0	0	0	0	0	0	0	0	0	0	0	0	0	0	0	0	0	0	0	0	1	0	0	0	0	0	0	Fungi; Ascomycota; Sordariomycetes; Diaporthales; Cryphonectriaceae; unidentified; Cryphonectriaceae_sp
New.ReferenceOTU4273	0	0	0	0	0	0	0	0	0	0	1	0	0	0	0	0	0	0	0	0	0	0	0	0	0	0	0	0	0	0	Fungi; Ascomycota; Sordariomycetes; Diaporthales; Diaporthaceae; unidentified; Diaporthaceae_sp
New.ReferenceOTU5168	0	0	0	0	2	0	0	0	0	0	0	0	0	0	0	0	0	0	0	0	0	0	0	0	0	0	0	0	0	0	Fungi; Ascomycota; Sordariomycetes; Diaporthales; Diaporthaceae; unidentified; Diaporthaceae_sp
SH091157.06FU_GU066635_reps	0	0	0	0	0	0	0	0	0	0	0	0	0	0	0	0	0	0	0	0	0	1	0	0	0	0	0	0	0	0	Fungi; Ascomycota; Sordariomycetes; Diaporthales; Diaporthaceae; unidentified; Diaporthaceae_sp
SH094082.06FU_JX156011_reps	0	0	0	0	0	0	1	0	0	0	0	0	0	1	0	0	0	0	0	0	0	0	0	0	0	0	0	0	0	0	Fungi; Ascomycota; Sordariomycetes; Diaporthales; Diaporthaceae; unidentified; Diaporthaceae_sp
New.ReferenceOTU1229	0	0	0	0	1	0	0	0	0	0	0	0	0	0	0	0	0	0	0	0	0	0	0	0	0	0	0	0	0	0	Fungi; Ascomycota; Sordariomycetes; Diaporthales; Incertae_sedis; Jobellisia; Jobellisia_guangdongensis
New.ReferenceOTU6493	0	0	0	0	0	0	1	0	1	1	0	2	0	0	0	0	0	0	0	0	0	0	0	0	1	0	0	0	0	0	Fungi; Ascomycota; Sordariomycetes; Diaporthales; Incertae_sedis; Jobellisia; Jobellisia_guangdongensis
New.ReferenceOTU268	0	0	0	1	0	0	2	0	0	0	0	1	0	0	0	0	0	0	0	0	0	0	0	0	0	0	0	0	0	0	Fungi; Ascomycota; Sordariomycetes; Diaporthales; unidentified; unidentified; Diaporthales_sp
New.ReferenceOTU2169	0	0	0	0	0	0	0	0	0	0	0	0	0	0	0	0	0	0	0	0	0	0	0	0	0	0	2	0	0	2	Fungi; Ascomycota; Sordariomycetes; Incertae_sedis; Annulatascaceae; unidentified; Cateractispora_recepticuli
New.ReferenceOTU3443	0	0	0	0	0	0	0	0	0	0	0	0	0	0	0	0	0	0	0	0	0	1	0	0	0	0	0	0	0	0	Fungi; Ascomycota; Sordariomycetes; Incertae_sedis; Annulatascaceae; unidentified; Cateractispora_recepticuli
New.ReferenceOTU4026	0	0	0	0	0	0	0	0	0	0	0	0	0	0	0	0	0	0	1	0	0	0	0	0	0	0	0	0	0	0	Fungi; Ascomycota; Sordariomycetes; Incertae_sedis; Annulatascaceae; unidentified; Cateractispora_recepticuli
New.ReferenceOTU4581	0	0	0	0	3	0	0	0	0	0	0	0	0	0	0	0	0	0	0	0	0	0	0	0	0	0	0	0	0	0	Fungi; Ascomycota; Sordariomycetes; Incertae_sedis; Annulatascaceae; unidentified; Cateractispora_recepticuli
New.ReferenceOTU6520	0	0	0	0	0	0	0	2	0	0	0	0	0	0	1	0	0	0	0	0	0	0	1	0	2	0	0	0	0	2	Fungi; Ascomycota; Sordariomycetes; Incertae_sedis; Annulatascaceae; unidentified; Cateractispora_recepticuli
New.ReferenceOTU6663	0	0	0	0	0	0	0	0	0	0	1	0	0	1	1	0	0	0	0	1	0	0	0	1	0	0	0	0	0	0	Fungi; Ascomycota; Sordariomycetes; Incertae_sedis; Annulatascaceae; unidentified; Cateractispora_recepticuli
New.ReferenceOTU6940	0	0	0	0	0	0	0	0	0	0	0	0	0	0	0	0	0	0	0	0	0	0	1	0	0	0	0	0	0	0	Fungi; Ascomycota; Sordariomycetes; Incertae_sedis; Annulatascaceae; unidentified; Cateractispora_recepticuli
New.ReferenceOTU7	0	0	0	0	0	0	0	0	1	2	0	0	0	1	0	0	1	0	1	2	0	0	0	1	0	0	0	0	0	0	Fungi; Ascomycota; Sordariomycetes; Incertae_sedis; Annulatascaceae; unidentified; Cateractispora_recepticuli
New.ReferenceOTU732	0	0	0	0	0	0	0	0	0	0	0	0	0	0	0	0	0	0	0	0	0	0	0	0	0	0	0	0	0	1	Fungi; Ascomycota; Sordariomycetes; Incertae_sedis; Annulatascaceae; unidentified; Cateractispora_recepticuli
SH021033.06FU_AF502620_reps_singleton	0	0	0	0	0	0	0	0	1	0	2	0	0	0	0	0	0	0	0	0	0	0	0	0	0	0	0	0	0	0	Fungi; Ascomycota; Sordariomycetes; Incertae_sedis; Apiosporaceae; unidentified; Apiosporaceae_sp
SH032168.06FU_JN890282_reps_singleton	0	0	0	0	0	0	0	0	0	0	3	0	0	0	0	0	0	0	0	0	0	0	0	0	0	0	0	0	0	0	Fungi; Ascomycota; Sordariomycetes; Incertae_sedis; Apiosporaceae; unidentified; Apiosporaceae_sp
SH121328.06FU_JQ647434_reps	0	0	0	0	0	0	0	0	0	0	0	0	0	0	0	0	0	0	0	0	0	0	0	1	0	0	0	0	0	0	Fungi; Ascomycota; Sordariomycetes; Incertae_sedis; Plectosphaerellaceae; unidentified; Plectosphaerellaceae_sp
New.CleanUp.ReferenceOTU684	0	0	0	0	0	0	0	0	0	1	0	0	0	4	0	2	0	0	0	3	0	0	0	0	0	1	1	2	0	1	Fungi; Ascomycota; Sordariomycetes; Microascales; Halosphaeriaceae; unidentified; Halosphaeriaceae_sp
New.ReferenceOTU157	0	0	0	0	0	0	0	0	0	0	3	0	0	0	0	0	0	0	0	0	0	0	0	0	0	0	0	0	0	0	Fungi; Ascomycota; Sordariomycetes; Microascales; Halosphaeriaceae; unidentified; Halosphaeriaceae_sp
New.ReferenceOTU5343	0	0	0	0	0	0	0	0	1	0	0	0	1	0	0	0	0	0	0	0	0	0	0	0	0	0	0	0	1	0	Fungi; Ascomycota; Sordariomycetes; Microascales; Halosphaeriaceae; unidentified; Halosphaeriaceae_sp
New.ReferenceOTU612	0	0	0	0	0	0	0	0	0	0	0	0	0	0	0	0	0	0	0	0	0	0	1	0	0	0	0	0	0	0	Fungi; Ascomycota; Sordariomycetes; Microascales; Halosphaeriaceae; unidentified; Halosphaeriaceae_sp
New.ReferenceOTU6435	0	0	0	0	0	0	0	0	0	0	0	0	0	0	0	0	0	0	0	0	0	0	0	0	1	0	0	0	0	0	Fungi; Ascomycota; Sordariomycetes; Microascales; Halosphaeriaceae; unidentified; Halosphaeriaceae_sp
New.ReferenceOTU7245	0	0	3	0	0	1	1	1	0	3	0	1	1	1	0	7	0	0	0	2	0	0	0	0	0	0	0	0	2	0	Fungi; Ascomycota; Sordariomycetes; Microascales; Halosphaeriaceae; unidentified; Halosphaeriaceae_sp
New.ReferenceOTU7252	0	0	0	0	0	0	0	0	0	0	0	0	0	0	0	1	0	0	0	0	0	1	0	0	0	0	0	0	0	0	Fungi; Ascomycota; Sordariomycetes; Microascales; Halosphaeriaceae; unidentified; Halosphaeriaceae_sp
New.ReferenceOTU3250	0	0	0	0	0	0	0	0	0	0	0	0	0	0	0	0	0	0	0	0	0	0	0	0	1	0	0	0	0	0	Fungi; Ascomycota; Sordariomycetes; Microascales; Microascaceae; unidentified; Microascaceae_sp
SH126811.06FU_AM774160_reps	0	0	0	0	0	0	0	0	0	0	0	0	0	0	0	0	0	0	0	0	0	1	0	0	0	0	0	0	0	0	Fungi; Ascomycota; Sordariomycetes; Microascales; Microascaceae; unidentified; Microascaceae_sp
SH136729.06FU_JN604456_reps	0	0	0	0	0	0	0	0	0	1	0	0	0	0	0	0	0	0	0	0	0	0	0	1	0	0	0	0	0	0	Fungi; Ascomycota; Sordariomycetes; Ophiostomatales; Ophiostomataceae; unidentified; Ophiostomataceae_sp_YTH_2012b
New.ReferenceOTU2442	0	0	0	0	0	0	0	0	0	0	0	0	0	1	0	0	0	0	0	0	0	0	0	0	0	0	0	0	0	0	Fungi; Ascomycota; Sordariomycetes; Phyllachorales; Phaeochoraceae; unidentified; uncultured_Serenomyces
New.ReferenceOTU3549	0	0	0	0	0	0	0	0	0	0	0	0	1	0	0	0	0	0	0	0	0	0	0	0	0	0	0	0	0	0	Fungi; Ascomycota; Sordariomycetes; Phyllachorales; Phaeochoraceae; unidentified; uncultured_Serenomyces
New.ReferenceOTU6371	1	0	0	0	0	0	0	0	0	0	0	0	0	0	0	0	0	0	0	0	0	0	0	0	0	0	0	0	0	0	Fungi; Ascomycota; Sordariomycetes; Phyllachorales; Phaeochoraceae; unidentified; uncultured_Serenomyces
New.ReferenceOTU6662	0	0	0	0	0	0	0	0	0	0	0	0	0	0	0	0	0	0	1	0	0	0	0	0	0	0	0	0	0	0	Fungi; Ascomycota; Sordariomycetes; Phyllachorales; Phaeochoraceae; unidentified; uncultured_Serenomyces
New.ReferenceOTU7162	0	1	0	0	0	0	0	0	0	0	0	0	0	0	0	0	0	0	0	0	0	0	0	0	0	0	0	0	0	0	Fungi; Ascomycota; Sordariomycetes; Phyllachorales; Phaeochoraceae; unidentified; uncultured_Serenomyces
New.CleanUp.ReferenceOTU1120	0	0	0	0	0	0	0	0	2	0	0	0	0	0	0	0	0	0	0	0	0	0	0	0	0	0	0	0	0	0	Fungi; Ascomycota; Sordariomycetes; Sordariales; Cephalothecaceae; unidentified; Cephalothecaceae_sp
New.ReferenceOTU2131	0	0	0	0	0	0	0	1	0	0	0	0	0	0	0	0	0	0	0	0	0	0	0	0	0	0	0	0	0	0	Fungi; Ascomycota; Sordariomycetes; Sordariales; Cephalothecaceae; unidentified; Cephalothecaceae_sp
New.ReferenceOTU35	0	0	0	2	0	0	0	0	0	0	0	0	0	0	0	0	0	0	0	0	0	0	0	0	0	0	0	0	0	0	Fungi; Ascomycota; Sordariomycetes; Sordariales; Cephalothecaceae; unidentified; Cephalothecaceae_sp
New.ReferenceOTU3751	1	0	0	0	0	0	0	0	0	0	0	0	0	0	0	0	0	0	0	0	0	1	0	0	0	0	0	0	0	0	Fungi; Ascomycota; Sordariomycetes; Sordariales; Cephalothecaceae; unidentified; Cephalothecaceae_sp
New.ReferenceOTU397	0	0	0	0	0	0	0	1	0	0	0	0	0	0	0	0	0	0	0	0	0	0	0	0	0	0	0	0	0	0	Fungi; Ascomycota; Sordariomycetes; Sordariales; Cephalothecaceae; unidentified; Cephalothecaceae_sp
New.ReferenceOTU4844	1	0	0	0	0	0	0	0	0	0	0	0	0	0	0	0	0	0	0	0	0	0	0	0	0	0	0	0	0	0	Fungi; Ascomycota; Sordariomycetes; Sordariales; Cephalothecaceae; unidentified; Cephalothecaceae_sp
New.ReferenceOTU5073	0	0	0	0	0	0	0	1	0	1	0	0	0	0	0	0	0	0	0	0	0	0	0	1	0	0	0	0	0	0	Fungi; Ascomycota; Sordariomycetes; Sordariales; Cephalothecaceae; unidentified; Cephalothecaceae_sp
New.ReferenceOTU5432	0	0	0	0	0	0	0	0	0	0	0	0	0	0	1	0	0	0	2	0	0	0	0	0	0	0	0	0	0	0	Fungi; Ascomycota; Sordariomycetes; Sordariales; Cephalothecaceae; unidentified; Cephalothecaceae_sp
New.ReferenceOTU552	0	0	0	0	1	3	0	0	0	0	0	1	0	0	0	0	0	0	0	0	0	0	0	0	0	0	0	1	0	0	Fungi; Ascomycota; Sordariomycetes; Sordariales; Cephalothecaceae; unidentified; Cephalothecaceae_sp
New.ReferenceOTU5698	0	0	0	0	0	0	8	0	1	0	0	2	0	0	1	0	1	0	0	0	0	0	2	0	0	0	0	0	0	0	Fungi; Ascomycota; Sordariomycetes; Sordariales; Cephalothecaceae; unidentified; Cephalothecaceae_sp
New.ReferenceOTU6261	0	0	0	0	0	0	0	0	0	0	0	0	0	0	0	0	1	0	0	0	0	0	0	0	0	0	0	0	0	0	Fungi; Ascomycota; Sordariomycetes; Sordariales; Cephalothecaceae; unidentified; Cephalothecaceae_sp
New.ReferenceOTU7028	0	0	0	0	0	0	0	0	0	3	0	0	0	0	0	0	2	0	0	0	0	0	0	0	0	0	0	0	0	0	Fungi; Ascomycota; Sordariomycetes; Sordariales; Cephalothecaceae; unidentified; Cephalothecaceae_sp
New.ReferenceOTU728	0	0	1	0	0	0	1	0	0	0	0	0	1	0	0	0	0	0	0	0	0	0	0	0	0	0	0	0	0	0	Fungi; Ascomycota; Sordariomycetes; Sordariales; Cephalothecaceae; unidentified; Cephalothecaceae_sp
New.ReferenceOTU7585	0	0	0	0	0	0	0	0	0	0	0	0	0	0	0	0	0	0	0	0	0	0	0	0	1	0	0	0	0	0	Fungi; Ascomycota; Sordariomycetes; Sordariales; Cephalothecaceae; unidentified; Cephalothecaceae_sp
New.ReferenceOTU977	0	0	0	0	0	0	0	0	0	0	0	0	0	0	0	0	0	0	0	0	0	0	2	0	0	0	0	0	0	0	Fungi; Ascomycota; Sordariomycetes; Sordariales; Cephalothecaceae; unidentified; Cephalothecaceae_sp
New.ReferenceOTU1421	0	0	0	0	0	0	0	0	0	0	0	0	0	0	0	0	0	0	0	0	0	0	0	0	0	0	1	0	0	0	Fungi; Ascomycota; Sordariomycetes; Sordariales; Chaetomiaceae; unidentified; Chaetomiaceae_sp
New.ReferenceOTU243	0	0	0	0	5	0	0	0	0	0	0	1	0	0	0	1	0	0	1	0	0	0	0	0	0	3	0	0	0	0	Fungi; Ascomycota; Sordariomycetes; Sordariales; Chaetomiaceae; unidentified; Chaetomiaceae_sp
New.ReferenceOTU2912	0	0	0	0	0	0	0	0	0	1	0	0	0	0	0	1	0	0	0	0	0	0	0	0	0	0	0	0	0	0	Fungi; Ascomycota; Sordariomycetes; Sordariales; Chaetomiaceae; unidentified; Chaetomiaceae_sp
New.ReferenceOTU4793	0	2	0	0	0	0	0	0	0	0	0	0	0	0	0	0	0	4	0	0	0	0	0	0	0	0	0	0	0	0	Fungi; Ascomycota; Sordariomycetes; Sordariales; Chaetomiaceae; unidentified; Chaetomiaceae_sp
New.ReferenceOTU555	0	0	1	0	0	0	0	0	0	0	0	0	0	0	0	0	0	1	0	0	0	0	0	0	0	0	0	0	0	0	Fungi; Ascomycota; Sordariomycetes; Sordariales; Chaetomiaceae; unidentified; Chaetomiaceae_sp
New.ReferenceOTU5551	0	0	0	0	0	0	0	0	0	0	0	0	0	0	0	0	0	0	0	0	0	2	0	0	2	0	0	1	0	2	Fungi; Ascomycota; Sordariomycetes; Sordariales; Chaetomiaceae; unidentified; Chaetomiaceae_sp
SH139396.06FU_GQ922575_reps	0	2	0	1	5	4	6	5	0	8	5	19	0	3	5	0	2	0	1	0	5	15	4	8	22	2	8	19	19	3	Fungi; Ascomycota; Sordariomycetes; Sordariales; Chaetomiaceae; unidentified; Chaetomiaceae_sp
New.ReferenceOTU46	0	0	0	0	0	0	0	0	0	0	0	0	0	0	0	0	0	0	0	0	0	0	0	2	0	0	0	0	0	0	Fungi; Ascomycota; Sordariomycetes; Sordariales; Incertae_sedis; Ascotaiwania; Ascotaiwania_lignicola
New.ReferenceOTU2198	0	0	0	0	3	0	0	0	0	0	0	0	0	0	0	0	0	0	0	0	0	0	0	0	0	0	0	0	0	0	Fungi; Ascomycota; Sordariomycetes; Sordariales; Incertae_sedis; Diplogelasinospora; Diplogelasinospora_grovesii
SH134067.06FU_AY681202_refs	0	0	0	0	2	0	0	0	0	0	0	0	0	0	0	0	0	0	0	0	0	0	0	0	0	0	0	2	0	0	Fungi; Ascomycota; Sordariomycetes; Sordariales; Incertae_sedis; Diplogelasinospora; Diplogelasinospora_grovesii
New.ReferenceOTU2904	0	0	0	3	2	4	0	0	1	0	0	4	2	0	3	0	0	0	0	0	0	0	1	0	0	0	16	2	0	12	Fungi; Ascomycota; Sordariomycetes; Sordariales; Incertae_sedis; Diplogelasinospora; Diplogelasinospora_inaequalis
SH029686.06FU_AY681201_reps_singleton	0	0	0	0	0	5	1	0	2	0	0	11	0	0	1	0	0	0	0	0	0	0	1	0	0	0	7	4	0	8	Fungi; Ascomycota; Sordariomycetes; Sordariales; Incertae_sedis; Diplogelasinospora; Diplogelasinospora_inaequalis
New.ReferenceOTU1837	0	0	0	0	0	3	1	0	0	0	0	0	0	0	0	0	0	0	0	0	0	0	0	0	0	0	0	0	0	0	Fungi; Ascomycota; Sordariomycetes; Sordariales; Incertae_sedis; Diplogelasinospora; Diplogelasinospora_sp
New.ReferenceOTU3019	0	0	0	0	0	0	0	0	0	0	0	0	0	0	0	0	0	0	0	1	0	0	0	0	0	0	0	0	0	0	Fungi; Ascomycota; Sordariomycetes; Sordariales; Lasiosphaeriaceae; unidentified; Lasiosphaeriaceae_sp
New.ReferenceOTU3212	0	0	0	0	0	0	0	0	0	0	0	0	0	0	0	0	0	0	0	0	0	2	0	0	0	0	0	0	0	0	Fungi; Ascomycota; Sordariomycetes; Sordariales; Lasiosphaeriaceae; unidentified; Lasiosphaeriaceae_sp
New.ReferenceOTU5445	0	0	0	0	0	0	0	2	0	0	0	0	0	0	0	0	0	0	0	0	0	3	5	0	1	0	2	0	0	0	Fungi; Ascomycota; Sordariomycetes; Sordariales; Lasiosphaeriaceae; unidentified; Lasiosphaeriaceae_sp
SH122452.06FU_AY999135_reps	0	0	0	0	0	0	0	0	0	0	0	0	0	0	0	0	0	0	0	0	0	3	0	0	0	0	0	0	0	1	Fungi; Ascomycota; Sordariomycetes; Sordariales; Lasiosphaeriaceae; unidentified; Lasiosphaeriaceae_sp
New.ReferenceOTU2710	0	0	0	0	0	0	0	2	0	0	1	0	0	0	0	0	0	0	0	0	0	0	0	0	0	0	0	0	0	0	Fungi; Ascomycota; Sordariomycetes; Sordariales; Lasiosphaeriaceae; unidentified; uncultured_Cercophora
New.ReferenceOTU7139	0	0	0	0	1	1	1	0	0	0	0	0	0	0	0	0	0	0	0	0	0	0	0	0	0	0	0	0	0	0	Fungi; Ascomycota; Sordariomycetes; Sordariales; Sordariaceae; unidentified; Sordariaceae_sp
New.ReferenceOTU1335	0	1	2	0	0	0	0	0	0	0	0	0	0	0	0	1	0	4	0	0	0	0	0	0	0	0	0	0	0	0	Fungi; Ascomycota; Sordariomycetes; Sordariales; unidentified; unidentified; Sordariales_sp
New.ReferenceOTU1527	0	0	0	0	0	0	0	0	0	0	0	0	0	0	0	0	0	0	0	1	0	0	0	0	0	0	0	0	0	0	Fungi; Ascomycota; Sordariomycetes; Sordariales; unidentified; unidentified; Sordariales_sp
New.ReferenceOTU17	0	0	0	0	0	0	0	0	0	0	0	0	0	0	0	0	0	0	1	0	0	0	0	0	0	0	0	0	0	0	Fungi; Ascomycota; Sordariomycetes; Sordariales; unidentified; unidentified; Sordariales_sp
New.ReferenceOTU192	0	0	0	0	0	0	0	0	0	0	0	0	0	0	0	0	0	0	0	0	0	0	1	0	0	0	0	0	0	0	Fungi; Ascomycota; Sordariomycetes; Sordariales; unidentified; unidentified; Sordariales_sp
New.ReferenceOTU2141	0	5	0	0	0	0	2	0	0	1	0	0	0	3	2	1	2	0	0	0	0	0	0	0	0	0	0	0	0	0	Fungi; Ascomycota; Sordariomycetes; Sordariales; unidentified; unidentified; Sordariales_sp
New.ReferenceOTU2357	0	0	0	1	0	0	0	0	0	0	0	0	0	0	0	0	0	0	0	0	0	0	0	0	0	0	0	0	0	0	Fungi; Ascomycota; Sordariomycetes; Sordariales; unidentified; unidentified; Sordariales_sp
New.ReferenceOTU2475	0	0	0	0	0	0	0	0	0	0	0	0	0	0	0	2	0	0	1	0	0	0	0	0	0	0	0	0	0	0	Fungi; Ascomycota; Sordariomycetes; Sordariales; unidentified; unidentified; Sordariales_sp
New.ReferenceOTU2688	0	0	0	0	0	0	0	0	0	1	0	0	0	0	0	0	0	0	0	0	0	0	0	0	0	0	0	0	0	0	Fungi; Ascomycota; Sordariomycetes; Sordariales; unidentified; unidentified; Sordariales_sp
New.ReferenceOTU2981	0	0	0	0	0	0	0	0	0	0	2	0	0	0	0	0	0	0	0	0	0	0	0	0	0	0	0	0	0	0	Fungi; Ascomycota; Sordariomycetes; Sordariales; unidentified; unidentified; Sordariales_sp
New.ReferenceOTU324	0	0	0	0	0	0	0	0	0	0	0	0	0	0	0	0	0	0	0	0	0	0	0	0	1	0	0	0	0	0	Fungi; Ascomycota; Sordariomycetes; Sordariales; unidentified; unidentified; Sordariales_sp
New.ReferenceOTU380	0	0	0	0	0	0	0	0	0	0	0	0	0	0	0	0	0	0	0	0	0	0	0	0	0	4	0	0	1	0	Fungi; Ascomycota; Sordariomycetes; Sordariales; unidentified; unidentified; Sordariales_sp
New.ReferenceOTU4235	0	0	0	0	0	0	0	0	0	0	0	0	0	0	0	0	0	0	1	0	0	0	0	0	0	0	0	0	0	0	Fungi; Ascomycota; Sordariomycetes; Sordariales; unidentified; unidentified; Sordariales_sp
New.ReferenceOTU4460	0	0	0	0	1	0	0	0	0	0	0	0	0	0	0	0	0	0	0	0	0	0	0	0	0	0	0	0	0	0	Fungi; Ascomycota; Sordariomycetes; Sordariales; unidentified; unidentified; Sordariales_sp
New.ReferenceOTU4513	0	0	0	0	0	0	0	0	0	0	0	0	0	0	1	0	0	0	0	0	0	0	0	0	0	0	0	0	0	1	Fungi; Ascomycota; Sordariomycetes; Sordariales; unidentified; unidentified; Sordariales_sp
New.ReferenceOTU4680	0	0	0	0	0	0	0	0	0	0	0	0	0	0	0	0	0	0	0	0	0	0	0	0	6	0	0	0	0	0	Fungi; Ascomycota; Sordariomycetes; Sordariales; unidentified; unidentified; Sordariales_sp
New.ReferenceOTU4694	0	0	0	0	0	0	0	0	0	2	0	0	0	1	0	0	0	0	0	0	0	0	0	1	0	0	0	0	0	0	Fungi; Ascomycota; Sordariomycetes; Sordariales; unidentified; unidentified; Sordariales_sp
New.ReferenceOTU5004	0	0	1	0	0	0	0	0	0	0	0	0	0	0	0	0	0	0	0	0	0	0	0	0	0	0	0	0	0	0	Fungi; Ascomycota; Sordariomycetes; Sordariales; unidentified; unidentified; Sordariales_sp
New.ReferenceOTU5093	0	0	0	0	0	0	0	0	0	1	0	0	0	0	0	0	0	0	0	0	0	0	0	0	0	0	0	0	0	0	Fungi; Ascomycota; Sordariomycetes; Sordariales; unidentified; unidentified; Sordariales_sp
New.ReferenceOTU5123	0	0	0	0	2	0	0	0	0	0	0	1	0	0	0	0	0	0	0	0	0	0	0	0	0	0	0	0	0	0	Fungi; Ascomycota; Sordariomycetes; Sordariales; unidentified; unidentified; Sordariales_sp
New.ReferenceOTU5254	0	0	0	0	0	0	0	0	0	0	0	0	0	0	0	0	0	0	0	0	0	0	0	1	0	0	0	0	0	0	Fungi; Ascomycota; Sordariomycetes; Sordariales; unidentified; unidentified; Sordariales_sp
New.ReferenceOTU5470	0	0	1	0	0	0	0	0	0	0	0	0	0	0	0	0	0	0	0	0	0	0	0	0	0	0	0	0	0	0	Fungi; Ascomycota; Sordariomycetes; Sordariales; unidentified; unidentified; Sordariales_sp
New.ReferenceOTU5714	0	0	0	0	0	0	0	0	1	0	0	0	0	0	0	0	0	0	0	0	0	0	0	0	0	0	0	0	0	0	Fungi; Ascomycota; Sordariomycetes; Sordariales; unidentified; unidentified; Sordariales_sp
New.ReferenceOTU6123	0	0	0	0	0	0	0	0	0	0	0	0	0	0	0	0	1	0	0	0	0	0	0	1	0	0	0	0	0	0	Fungi; Ascomycota; Sordariomycetes; Sordariales; unidentified; unidentified; Sordariales_sp
New.ReferenceOTU6505	0	0	0	0	0	0	0	0	0	0	0	0	0	0	0	0	0	0	1	0	0	0	0	0	0	0	0	0	0	0	Fungi; Ascomycota; Sordariomycetes; Sordariales; unidentified; unidentified; Sordariales_sp
New.ReferenceOTU6605	0	0	0	0	0	0	0	0	1	0	0	0	0	0	0	0	0	0	0	0	0	0	0	0	0	0	0	0	0	0	Fungi; Ascomycota; Sordariomycetes; Sordariales; unidentified; unidentified; Sordariales_sp
New.ReferenceOTU6752	0	0	0	0	0	0	0	0	0	0	0	0	1	0	0	0	0	0	0	0	0	0	0	0	0	0	0	0	0	0	Fungi; Ascomycota; Sordariomycetes; Sordariales; unidentified; unidentified; Sordariales_sp
New.ReferenceOTU7266	0	0	2	0	0	0	0	0	0	0	0	0	0	0	0	0	0	0	0	0	0	0	0	0	0	0	0	0	0	0	Fungi; Ascomycota; Sordariomycetes; Sordariales; unidentified; unidentified; Sordariales_sp
New.ReferenceOTU7744	0	0	0	0	0	0	0	0	0	0	0	3	0	0	0	0	0	0	0	0	0	2	3	0	0	0	0	0	0	0	Fungi; Ascomycota; Sordariomycetes; Sordariales; unidentified; unidentified; Sordariales_sp

New.ReferenceOTU7845	0	0	0	0	0	0	0	0	0	0	0	0	0	0	1	0	0	1	0	0	0	0	0	0	0	0	0	0	0	0	Fungi; Ascomycota; Sordariomycetes; Sordariales; unidentified; unidentified; Sordariales_sp
New.ReferenceOTU989	0	0	0	0	0	0	0	0	0	0	0	0	0	0	0	0	0	0	0	0	0	0	0	0	0	0	0	0	0	2	Fungi; Ascomycota; Sordariomycetes; Sordariales; unidentified; unidentified; Sordariales_sp
SH091846.06FU_EU372828_reps	0	0	0	0	0	0	0	0	0	1	0	0	0	0	0	0	0	0	0	0	0	0	0	0	0	0	0	0	0	0	Fungi; Ascomycota; Sordariomycetes; Sordariales; unidentified; unidentified; Sordariales_sp
SH146004.06FU_JQ081850_reps	0	0	0	0	0	2	0	2	3	2	1	1	1	1	2	1	0	0	0	0	2	0	0	0	0	0	0	0	0	0	Fungi; Ascomycota; Sordariomycetes; Sordariales; unidentified; unidentified; Sordariales_sp
SH454877.06FU_AY969595_reps_singleton	0	0	0	0	0	0	0	0	0	1	0	0	0	0	0	0	0	0	0	0	0	0	0	0	0	0	0	0	0	0	Fungi; Ascomycota; Sordariomycetes; Sordariales; unidentified; unidentified; Sordariales_sp
New.ReferenceOTU181	0	1	1	0	3	0	3	12	0	2	0	1	2	0	1	0	4	0	0	0	2	4	2	1	0	0	2	2	0	2	Fungi; Ascomycota; Sordariomycetes; Trichosphaeriales; Incertae_sedis; unidentified; Trichosphaeriales_sp
New.ReferenceOTU5400	0	0	0	0	0	0	0	0	0	0	0	0	0	0	0	0	1	0	0	0	0	0	0	0	0	0	0	0	0	0	Fungi; Ascomycota; Sordariomycetes; Trichosphaeriales; Incertae_sedis; unidentified; Trichosphaeriales_sp
New.ReferenceOTU6302	0	0	0	0	0	0	0	0	1	0	0	0	0	0	0	1	0	0	0	0	0	0	0	1	0	0	0	0	0	0	Fungi; Ascomycota; Sordariomycetes; Trichosphaeriales; Incertae_sedis; unidentified; Trichosphaeriales_sp
New.ReferenceOTU7083	0	0	0	0	0	0	0	0	0	1	0	0	0	0	0	0	0	0	0	0	0	0	0	0	0	0	0	0	0	0	Fungi; Ascomycota; Sordariomycetes; Trichosphaeriales; Incertae_sedis; unidentified; Trichosphaeriales_sp
SH141998.06FU_EU685984_reps	0	0	0	0	0	0	0	0	0	0	0	0	0	0	0	0	0	0	0	0	0	0	0	0	0	0	0	0	0	1	Fungi; Ascomycota; Sordariomycetes; Trichosphaeriales; Incertae_sedis; unidentified; Trichosphaeriales_sp
SH141999.06FU_GQ919075_reps	0	0	0	1	0	0	0	0	0	0	0	0	0	0	0	0	0	0	0	0	0	0	0	0	0	0	0	0	0	0	Fungi; Ascomycota; Sordariomycetes; Trichosphaeriales; Incertae_sedis; unidentified; Trichosphaeriales_sp
SH142002.06FU_DQ219433_reps	0	0	0	0	0	0	0	0	0	1	0	0	0	0	0	0	0	0	0	0	0	0	0	0	0	0	0	0	0	0	Fungi; Ascomycota; Sordariomycetes; Trichosphaeriales; Incertae_sedis; unidentified; Trichosphaeriales_sp
SH142004.06FU_JX559559_reps	0	1	4	0	2	1	5	7	0	4	4	3	0	8	1	3	5	0	0	0	3	5	4	2	0	0	3	6	0	1	Fungi; Ascomycota; Sordariomycetes; Trichosphaeriales; Incertae_sedis; unidentified; Trichosphaeriales_sp
SH142005.06FU_EU002898_reps	0	1	4	3	3	4	9	18	0	5	1	12	2	5	2	3	12	0	0	0	2	5	0	1	7	0	10	19	0	11	Fungi; Ascomycota; Sordariomycetes; Trichosphaeriales; Incertae_sedis; unidentified; Trichosphaeriales_sp
SH142006.06FU_JN687964_reps	0	0	0	0	2	0	0	0	0	0	0	1	0	1	0	0	1	0	0	0	1	0	0	0	1	0	0	2	0	0	Fungi; Ascomycota; Sordariomycetes; Trichosphaeriales; Incertae_sedis; unidentified; Trichosphaeriales_sp
SH142019.06FU_FR668016_reps	1	0	1	0	2	0	2	5	0	2	0	1	2	1	1	1	5	0	0	0	0	1	1	0	0	0	3	4	0	2	Fungi; Ascomycota; Sordariomycetes; Trichosphaeriales; Incertae_sedis; unidentified; Trichosphaeriales_sp
SH142017.06FU_GQ999534_reps	0	0	0	0	0	0	0	0	0	0	0	0	0	0	0	0	0	0	0	0	0	1	0	0	0	0	0	0	0	0	Fungi; Ascomycota; Sordariomycetes; Trichosphaeriales; unidentified; unidentified; Trichosphaeriales_sp
New.CleanUp.ReferenceOTU1325	0	0	0	0	0	0	0	0	0	0	0	0	0	0	0	1	0	0	0	0	0	0	0	0	0	0	0	0	0	0	Fungi; Ascomycota; Sordariomycetes; unidentified; unidentified; unidentified; Sordariomycetes_sp
New.CleanUp.ReferenceOTU1581	0	0	0	0	0	0	0	0	0	0	0	0	0	0	0	0	0	2	0	0	0	0	0	0	0	0	0	0	0	0	Fungi; Ascomycota; Sordariomycetes; unidentified; unidentified; unidentified; Sordariomycetes_sp
New.CleanUp.ReferenceOTU2076	0	0	0	0	0	0	0	0	0	0	0	0	0	0	0	0	0	0	0	2	0	0	0	0	0	0	0	0	0	0	Fungi; Ascomycota; Sordariomycetes; unidentified; unidentified; unidentified; Sordariomycetes_sp
New.CleanUp.ReferenceOTU985	0	0	0	0	0	0	0	0	0	0	0	0	0	0	0	0	0	0	0	0	0	0	0	0	0	0	0	1	0	0	Fungi; Ascomycota; Sordariomycetes; unidentified; unidentified; unidentified; Sordariomycetes_sp
New.ReferenceOTU1045	0	0	0	0	0	0	0	0	0	0	0	0	0	0	0	0	0	0	0	0	0	0	0	0	0	14	0	0	0	0	Fungi; Ascomycota; Sordariomycetes; unidentified; unidentified; unidentified; Sordariomycetes_sp
New.ReferenceOTU1169	0	0	0	0	2	0	0	0	0	0	0	0	0	0	0	0	0	0	0	0	0	0	0	0	0	0	0	0	0	0	Fungi; Ascomycota; Sordariomycetes; unidentified; unidentified; unidentified; Sordariomycetes_sp
New.ReferenceOTU1190	1	0	1	0	0	0	0	0	1	0	0	0	0	0	0	1	0	0	0	0	0	0	0	0	0	0	0	0	0	0	Fungi; Ascomycota; Sordariomycetes; unidentified; unidentified; unidentified; Sordariomycetes_sp
New.ReferenceOTU1259	0	2	3	0	2	2	0	3	0	0	0	0	0	0	0	0	0	1	0	0	2	0	0	0	1	3	0	4	11	0	Fungi; Ascomycota; Sordariomycetes; unidentified; unidentified; unidentified; Sordariomycetes_sp
New.ReferenceOTU1308	0	1	0	0	6	1	0	0	0	0	0	0	0	0	0	0	0	0	0	0	0	0	0	0	0	0	0	0	0	0	Fungi; Ascomycota; Sordariomycetes; unidentified; unidentified; unidentified; Sordariomycetes_sp
New.ReferenceOTU1317	0	0	0	0	0	0	0	0	0	0	1	0	0	0	0	0	0	0	0	0	0	0	0	1	0	0	0	0	0	0	Fungi; Ascomycota; Sordariomycetes; unidentified; unidentified; unidentified; Sordariomycetes_sp
New.ReferenceOTU1437	0	0	0	0	0	0	0	0	0	0	0	0	0	0	0	0	0	0	0	0	0	0	1	0	0	0	0	0	0	0	Fungi; Ascomycota; Sordariomycetes; unidentified; unidentified; unidentified; Sordariomycetes_sp
New.ReferenceOTU147	0	5	0	0	0	0	0	0	0	2	0	0	0	2	0	1	0	2	3	0	0	0	0	1	0	0	0	0	0	0	Fungi; Ascomycota; Sordariomycetes; unidentified; unidentified; unidentified; Sordariomycetes_sp
New.ReferenceOTU1517	0	0	0	0	0	0	0	0	0	0	2	0	0	0	0	0	0	0	0	0	0	0	0	0	0	0	0	0	0	0	Fungi; Ascomycota; Sordariomycetes; unidentified; unidentified; unidentified; Sordariomycetes_sp
New.ReferenceOTU1629	0	0	0	0	0	0	0	0	0	0	0	0	2	0	0	0	0	0	0	0	0	0	0	0	0	0	0	1	0	0	Fungi; Ascomycota; Sordariomycetes; unidentified; unidentified; unidentified; Sordariomycetes_sp
New.ReferenceOTU1675	0	0	0	0	0	0	0	0	0	0	0	1	0	0	0	1	0	0	0	0	0	0	0	0	0	0	0	0	0	0	Fungi; Ascomycota; Sordariomycetes; unidentified; unidentified; unidentified; Sordariomycetes_sp
New.ReferenceOTU1705	0	0	0	0	0	0	0	0	0	0	0	0	1	0	0	0	0	0	0	0	0	0	0	0	0	0	0	0	0	0	Fungi; Ascomycota; Sordariomycetes; unidentified; unidentified; unidentified; Sordariomycetes_sp
New.ReferenceOTU1721	0	0	0	0	0	0	0	0	0	1	0	0	0	0	0	0	0	0	0	0	0	1	0	0	0	0	0	0	0	0	Fungi; Ascomycota; Sordariomycetes; unidentified; unidentified; unidentified; Sordariomycetes_sp
New.ReferenceOTU1728	1	0	0	0	0	0	0	0	0	0	0	0	0	0	0	0	0	0	0	0	0	0	0	0	0	0	0	0	0	0	Fungi; Ascomycota; Sordariomycetes; unidentified; unidentified; unidentified; Sordariomycetes_sp
New.ReferenceOTU1737	0	0	0	0	0	0	0	0	0	0	0	0	0	0	0	0	0	0	0	0	0	0	0	0	0	0	0	0	0	1	Fungi; Ascomycota; Sordariomycetes; unidentified; unidentified; unidentified; Sordariomycetes_sp
New.ReferenceOTU1778	0	0	0	0	3	0	0	0	5	2	6	2	0	0	0	0	0	0	0	0	0	0	0	0	0	0	0	0	0	0	Fungi; Ascomycota; Sordariomycetes; unidentified; unidentified; unidentified; Sordariomycetes_sp
New.ReferenceOTU1862	0	0	0	0	0	0	0	0	0	0	0	0	0	0	0	0	0	0	0	0	0	0	0	0	2	0	0	0	0	0	Fungi; Ascomycota; Sordariomycetes; unidentified; unidentified; unidentified; Sordariomycetes_sp
New.ReferenceOTU1972	0	0	0	0	0	0	0	0	0	0	0	1	0	1	0	0	0	0	0	0	0	0	0	0	0	0	0	0	0	0	Fungi; Ascomycota; Sordariomycetes; unidentified; unidentified; unidentified; Sordariomycetes_sp
New.ReferenceOTU2102	0	0	0	0	0	0	0	0	0	0	0	0	0	0	0	0	0	6	0	0	0	0	0	0	0	0	0	0	0	0	Fungi; Ascomycota; Sordariomycetes; unidentified; unidentified; unidentified; Sordariomycetes_sp
New.ReferenceOTU2281	0	0	0	0	0	0	0	0	0	0	0	0	0	1	0	0	0	0	0	0	0	0	0	0	0	0	0	0	0	0	Fungi; Ascomycota; Sordariomycetes; unidentified; unidentified; unidentified; Sordariomycetes_sp
New.ReferenceOTU2285	0	0	0	0	0	2	0	0	0	0	0	0	0	0	3	0	0	0	0	0	0	0	0	0	0	0	0	1	0	0	Fungi; Ascomycota; Sordariomycetes; unidentified; unidentified; unidentified; Sordariomycetes_sp
New.ReferenceOTU2302	0	0	0	0	0	0	0	0	0	0	0	0	0	0	0	0	0	8	0	0	0	0	0	0	0	0	0	0	0	0	Fungi; Ascomycota; Sordariomycetes; unidentified; unidentified; unidentified; Sordariomycetes_sp
New.ReferenceOTU2360	0	0	1	0	0	0	0	0	0	0	0	0	0	0	0	0	0	0	0	0	0	0	0	0	0	0	0	0	0	0	Fungi; Ascomycota; Sordariomycetes; unidentified; unidentified; unidentified; Sordariomycetes_sp
New.ReferenceOTU2398	0	0	0	0	1	0	0	0	0	0	0	0	0	0	0	0	0	0	0	0	0	0	0	0	0	0	0	0	0	0	Fungi; Ascomycota; Sordariomycetes; unidentified; unidentified; unidentified; Sordariomycetes_sp
New.ReferenceOTU2401	0	0	0	0	0	0	0	0	3	0	0	0	0	0	0	0	0	6	12	5	0	0	8	0	41	0	8	0	0	0	Fungi; Ascomycota; Sordariomycetes; unidentified; unidentified; unidentified; Sordariomycetes_sp
New.ReferenceOTU2467	0	0	0	0	0	0	0	0	0	1	0	0	0	0	1	0	0	0	0	0	0	0	0	0	0	0	0	0	0	0	Fungi; Ascomycota; Sordariomycetes; unidentified; unidentified; unidentified; Sordariomycetes_sp
New.ReferenceOTU2470	0	0	0	0	0	0	0	0	2	0	0	0	0	0	0	0	0	0	0	0	0	0	0	0	0	0	0	0	0	0	Fungi; Ascomycota; Sordariomycetes; unidentified; unidentified; unidentified; Sordariomycetes_sp
New.ReferenceOTU2472	0	0	0	0	4	0	1	0	0	0	2	0	2	0	0	0	0	0	0	0	1	0	1	0	1	0	0	0	0	0	Fungi; Ascomycota; Sordariomycetes; unidentified; unidentified; unidentified; Sordariomycetes_sp
New.ReferenceOTU2480	0	0	0	0	0	0	0	0	0	0	0	0	0	0	0	0	0	3	0	0	0	0	0	0	0	0	0	0	0	0	Fungi; Ascomycota; Sordariomycetes; unidentified; unidentified; unidentified; Sordariomycetes_sp
New.ReferenceOTU2509	0	0	0	0	0	0	0	0	5	0	0	0	0	0	0	0	0	0	0	0	0	0	0	0	0	0	0	0	0	0	Fungi; Ascomycota; Sordariomycetes; unidentified; unidentified; unidentified; Sordariomycetes_sp
New.ReferenceOTU2513	0	0	0	0	0	0	0	0	0	0	0	0	0	0	0	0	0	0	0	1	0	0	0	0	0	0	0	0	0	0	Fungi; Ascomycota; Sordariomycetes; unidentified; unidentified; unidentified; Sordariomycetes_sp
New.ReferenceOTU2741	0	0	0	0	0	0	0	0	0	0	0	0	0	0	3	0	0	0	0	2	0	0	1	0	5	0	5	0	0	0	Fungi; Ascomycota; Sordariomycetes; unidentified; unidentified; unidentified; Sordariomycetes_sp
New.ReferenceOTU2774	0	0	0	0	0	0	0	0	0	0	0	0	0	0	0	0	0	0	0	0	0	2	0	0	0	0	0	0	0	0	Fungi; Ascomycota; Sordariomycetes; unidentified; unidentified; unidentified; Sordariomycetes_sp
New.ReferenceOTU2802	6	0	0	1	0	0	0	0	0	0	0	0	0	0	0	0	0	0	0	0	0	0	0	0	0	0	0	0	0	0	Fungi; Ascomycota; Sordariomycetes; unidentified; unidentified; unidentified; Sordariomycetes_sp
New.ReferenceOTU2831	1	0	0	0	0	0	0	0	0	0	0	0	0	0	0	0	0	0	0	0	0	0	0	0	0	0	0	0	0	0	Fungi; Ascomycota; Sordariomycetes; unidentified; unidentified; unidentified; Sordariomycetes_sp
New.ReferenceOTU294	0	0	0	0	0	0	0	0	0	0	0	0	0	0	0	0	0	0	0	1	0	0	0	1	0	0	0	0	0	0	Fungi; Ascomycota; Sordariomycetes; unidentified; unidentified; unidentified; Sordariomycetes_sp
New.ReferenceOTU3092	0	0	0	0	0	0	0	0	0	0	0	0	0	0	0	0	0	0	0	0	0	0	0	1	0	0	0	0	0	0	Fungi; Ascomycota; Sordariomycetes; unidentified; unidentified; unidentified; Sordariomycetes_sp
New.ReferenceOTU3188	0	0	0	0	0	0	0	0	2	0	0	0	0	0	0	0	0	0	0	0	0	0	0	0	0	0	0	0	0	0	Fungi; Ascomycota; Sordariomycetes; unidentified; unidentified; unidentified; Sordariomycetes_sp
New.ReferenceOTU3200	0	0	0	0	0	0	0	0	0	0	0	0	1	0	0	0	0	0	0	0	0	0	0	0	0	0	0	0	0	0	Fungi; Ascomycota; Sordariomycetes; unidentified; unidentified; unidentified; Sordariomycetes_sp
New.ReferenceOTU3238	0	0	0	0	0	0	0	0	0	0	0	0	0	0	0	0	0	1	0	0	0	0	0	0	0	0	0	0	0	0	Fungi; Ascomycota; Sordariomycetes; unidentified; unidentified; unidentified; Sordariomycetes_sp
New.ReferenceOTU3245	1	2	0	0	0	1	0	0	0	0	0	0	0	0	0	0	0	0	0	0	0	0	0	0	0	1	0	5	2	0	Fungi; Ascomycota; Sordariomycetes; unidentified; unidentified; unidentified; Sordariomycetes_sp
New.ReferenceOTU3293	0	0	0	0	0	0	0	0	0	4	0	0	1	2	0	1	1	0	0	0	0	0	0	2	0	0	0	0	0	0	Fungi; Ascomycota; Sordariomycetes; unidentified; unidentified; unidentified; Sordariomycetes_sp
New.ReferenceOTU3317	1	0	3	9	6	22	20	1	0	9	0	3	13	0	1	1	7	1	0	0	1	4	0	5	1	0	1	3	0	14	Fungi; Ascomycota; Sordariomycetes; unidentified; unidentified; unidentified; Sordariomycetes_sp
New.ReferenceOTU3475	0	0	0	0	7	0	0	0	0	0	0	0	0	0	0	0	0	0	0	0	0	0	0	0	0	0	0	0	0	0	Fungi; Ascomycota; Sordariomycetes; unidentified; unidentified; unidentified; Sordariomycetes_sp
New.ReferenceOTU3483	0	5	0	0	0	0	0	0	0	0	0	0	0	0	0	0	0	0	0	1	0	0	0	0	0	0	0	0	0	0	Fungi; Ascomycota; Sordariomycetes; unidentified; unidentified; unidentified; Sordariomycetes_sp
New.ReferenceOTU3498	0	0	0	0	0	0	0	0	0	0	0	0	0	0	0	0	0	1	0	0	0	0	0	0	0	0	0	0	0	0	Fungi; Ascomycota; Sordariomycetes; unidentified; unidentified; unidentified; Sordariomycetes_sp
New.ReferenceOTU3650	0	0	0	0	0	0	0	0	0	0	0	0	0	2	0	0	0	0	0	0	0	0	0	0	0	0	0	0	0	0	Fungi; Ascomycota; Sordariomycetes; unidentified; unidentified; unidentified; Sordariomycetes_sp
New.ReferenceOTU3667	0	0	0	0	1	0	0	0	0	0	0	0	0	0	0	0	0	0	0	0	0	0	0	0	0	0	0	0	0	0	Fungi; Ascomycota; Sordariomycetes; unidentified; unidentified; unidentified; Sordariomycetes_sp
New.ReferenceOTU3852	0	0	0	0	1	0	0	0	0	1	0	0	0	0	2	1	0	0	0	1	0	0	0	2	0	0	5	1	0	3	Fungi; Ascomycota; Sordariomycetes; unidentified; unidentified; unidentified; Sordariomycetes_sp
New.ReferenceOTU4041	0	0	0	0	0	0	0	0	0	0	0	0	1	0	0	0	0	0	0	0	0	0	0	0	0	0	0	0	0	0	Fungi; Ascomycota; Sordariomycetes; unidentified; unidentified; unidentified; Sordariomycetes_sp
New.ReferenceOTU4066	0	0	0	0	0	0	0	1	0	0	0	0	0	0	0	0	0	0	0	0	0	0	0	0	0	0	0	0	0	0	Fungi; Ascomycota; Sordariomycetes; unidentified; unidentified; unidentified; Sordariomycetes_sp
New.ReferenceOTU4277	0	0	0	0	53	0	0	0	0	0	0	1	0	0	0	0	1	0	0	0	0	0	0	0	0	1	0	0	0	0	Fungi; Ascomycota; Sordariomycetes; unidentified; unidentified; unidentified; Sordariomycetes_sp
New.ReferenceOTU4288	0	0	0	0	0	0	0	0	0	0	0	0	0	0	0	0	0	0	0	0	0	7	1	0	8	0	0	0	0	0	Fungi; Ascomycota; Sordariomycetes; unidentified; unidentified; unidentified; Sordariomycetes_sp
New.ReferenceOTU4343	0	0	0	0	2	0	0	0	0	0	0	0	0	0	0	0	0	0	0	0	0	0	0	0	0	4	0	0	0	0	Fungi; Ascomycota; Sordariomycetes; unidentified; unidentified; unidentified; Sordariomycetes_sp
New.ReferenceOTU4366	0	0	3	0	0	0	0	0	0	0	2	0	0	0	0	0	0	0	0	0	0	0	0	0	0	0	0	0	0	0	Fungi; Ascomycota; Sordariomycetes; unidentified; unidentified; unidentified; Sordariomycetes_sp
New.ReferenceOTU4383	0	0	2	0	0	0	0	0	4	3	1	0	0	0	0	0	2	0	3	5	0	0	0	0	0	0	0	0	0	0	Fungi; Ascomycota; Sordariomycetes; unidentified; unidentified; unidentified; Sordariomycetes_sp
New.ReferenceOTU4462	0	0	0	0	0	0	0	0	0	0	0	0	0	0	0	0	0	0	0	0	0	0	0	0	0	0	0	0	0	1	Fungi; Ascomycota; Sordariomycetes; unidentified; unidentified; unidentified; Sordariomycetes_sp
New.ReferenceOTU4495	0	0	1	0	0	0	0	0	1	0	1	0	0	0	1	0	0	0	0	0	0	0	0	0	0	0	0	0	0	0	Fungi; Ascomycota; Sordariomycetes; unidentified; unidentified; unidentified; Sordariomycetes_sp
New.ReferenceOTU466	0	0	0	0	0	0	0	0	0	0	0	0	0	0	0	0	0	0	0	0	0	0	0	0	0	2	0	0	0	0	Fungi; Ascomycota; Sordariomycetes; unidentified; unidentified; unidentified; Sordariomycetes_sp
New.ReferenceOTU4677	1	0	0	0	0	0	0	0	0	0	0	0	0	0	0	0	0	0	0	0	0	0	0	0	0	0	0	0	0	0	Fungi; Ascomycota; Sordariomycetes; unidentified; unidentified; unidentified; Sordariomycetes_sp
New.ReferenceOTU4683	0	0	0	0	0	0	0	0	0	0	0	0	0	0	0	0	0	0	0	0	0	0	0	0	3	0	0	0	0	0	Fungi; Ascomycota; Sordariomycetes; unidentified; unidentified; unidentified; Sordariomycetes_sp
New.ReferenceOTU4739	0	0	0	0	0	0	0	0	0	0	0	0	0	0	1	0	0	0	0	0	0	0	0	0	0	0	0	0	0	0	Fungi; Ascomycota; Sordariomycetes; unidentified; unidentified; unidentified; Sordariomycetes_sp
New.ReferenceOTU5025	0	0	0	0	0	0	0	0	0	0	0	1	0	0	0	0	1	0	0	0	0	2	0	0	0	0	1	0	0	4	Fungi; Ascomycota; Sordariomycetes; unidentified; unidentified; unidentified; Sordariomycetes_sp
New.ReferenceOTU5132	0	0	0	0	0	0	0	0	0	0	0	0	0	0	0	0	0	0	0	1	0	0	0	0	0	0	0	0	0	0	Fungi; Ascomycota; Sordariomycetes; unidentified; unidentified; unidentified; Sordariomycetes_sp
New.ReferenceOTU52	0	0	0	0	0	0	0	0	0	0	0	0	0	1	0	0	0	1	0	0	0	0	0	1	0	0	0	0	0	0	Fungi; Ascomycota; Sordariomycetes; unidentified; unidentified; unidentified; Sordariomycetes_sp
New.ReferenceOTU530	0	4	0	0	1	0	0	0	0	0	0	2	0	0	0	0	0	1	0	0	0	1	0	0	1	1	0	2	4	0	Fungi; Ascomycota; Sordariomycetes; unidentified; unidentified; unidentified; Sordariomycetes_sp
New.ReferenceOTU5312	0	0	0	0	1	0	0	0	0	0	0	0	0	0	0	0	0	0	0	0	0	0	0	0	0	0	0	0	0	0	Fungi; Ascomycota; Sordariomycetes; unidentified; unidentified; unidentified; Sordariomycetes_sp
New.ReferenceOTU5372	0	0	0	0	0	0	0	0	0	0	0	0	0	0	0	0	0	0	0	0	1	0	0	0	0	0	0	0	0	0	Fungi; Ascomycota; Sordariomycetes; unidentified; unidentified; unidentified; Sordariomycetes_sp
New.ReferenceOTU5475	0	0	0	0	4	1	0	0	0	1	0	0	0	0	0	0	0	0	0	0	0	0	0	0	0	0	0	1	0	0	Fungi; Ascomycota; Sordariomycetes; unidentified; unidentified; unidentified; Sordariomycetes_sp
New.ReferenceOTU5503	0	0	0	0	0	0	0	0	2	0	0	0	0	0	0	0	0	0	0	0	0	0	0	0	0	0	0	0	0	0	Fungi; Ascomycota; Sordariomycetes; unidentified; unidentified; unidentified; Sordariomycetes_sp
New.ReferenceOTU5567	0	0	0	0	0	0	0	0	0	0	0	0	0	0	0	0	0	0	0	0	0	0	1	0	1	0	0	0	0	0	Fungi; Ascomycota; Sordariomycetes; unidentified; unidentified; unidentified; Sordariomycetes_sp
New.ReferenceOTU5593	0	0	0	0	0	0	0	0	0	1	0	1	0	0	0	1	0	0	0	0	0	0	0	0	0	0	0	0	0	0	Fungi; Ascomycota; Sordariomycetes; unidentified; unidentified; unidentified; Sordariomycetes_sp
New.ReferenceOTU5635	0	0	0	0	0	0	0	0	11	0	3	0	0	0	0	0	0	0	1	0	0	0	0	0	0	0	0	0	0	0	Fungi; Ascomycota; Sordariomycetes; unidentified; unidentified; unidentified; Sordariomycetes_sp
New.ReferenceOTU5699	0	0	0	0	0	0	0	0	0	0	1	0	0	0	0	0	0	0	0	0	0	0	0	0	0	0	0	0	0	0	Fungi; Ascomycota; Sordariomycetes; unidentified; unidentified; unidentified; Sordariomycetes_sp
New.ReferenceOTU5742	0	5	0	1	0	0	0	0	0	0	0	0	0	0	0	0	0	4	0	0	0	0	0	0	0	0	0	0	3	0	Fungi; Ascomycota; Sordariomycetes; unidentified; unidentified; unidentified; Sordariomycetes_sp
New.ReferenceOTU5789	0	0	0	0	0	0	0	0	0	0	0	0	0	0	0	0	0	0	0	0	0	0	0	0	0	0	0	0	0	1	Fungi; Ascomycota; Sordariomycetes; unidentified; unidentified; unidentified; Sordariomycetes_sp
New.ReferenceOTU5811	0	0	0	0	0	0	0	0	0	0	0	0	0	0	0	0	0	0	0	1	0	0	0	0	0	0	0	0	0	0	Fungi; Ascomycota; Sordariomycetes; unidentified; unidentified; unidentified; Sordariomycetes_sp
New.ReferenceOTU584	0	4	2	0	20	0	0	0	7	0	0	2	0	0	0	0	0	0	0	0	0	1	0	0	2	3	0	2	1	0	Fungi; Ascomycota; Sordariomycetes; unidentified; unidentified; unidentified; Sordariomycetes_sp
New.ReferenceOTU6102	0	0	0	0	0	0	0	0	1	0	0	0	0	0	0	1	0	0	0	0	0	0	0	0	0	0	0	0	0	0	Fungi; Ascomycota; Sordariomycetes; unidentified; unidentified; unidentified; Sordariomycetes_sp

New.ReferenceOTU620	0	0	0	0	0	0	0	0	0	0	0	0	0	0	0	1	0	0	0	0	0	0	0	0	0	0	0	0	0	0	Fungi; Ascomycota; Sordariomycetes; unidentified; unidentified; unidentified; Sordariomycetes_sp
New.ReferenceOTU6265	0	0	0	4	0	0	0	0	0	0	0	0	0	0	0	0	0	0	0	0	0	0	0	4	0	0	0	0	0	0	Fungi; Ascomycota; Sordariomycetes; unidentified; unidentified; unidentified; Sordariomycetes_sp
New.ReferenceOTU6388	0	0	0	0	0	0	0	0	0	0	0	0	0	0	0	0	0	0	0	1	0	0	0	1	0	0	0	0	0	0	Fungi; Ascomycota; Sordariomycetes; unidentified; unidentified; unidentified; Sordariomycetes_sp
New.ReferenceOTU6476	0	0	0	0	0	0	0	0	0	0	0	0	0	0	0	0	0	0	0	0	0	0	0	1	0	0	0	0	0	0	Fungi; Ascomycota; Sordariomycetes; unidentified; unidentified; unidentified; Sordariomycetes_sp
New.ReferenceOTU6545	0	0	0	0	0	0	0	0	0	0	0	0	0	0	0	0	0	0	0	0	0	1	0	0	0	0	0	0	0	0	Fungi; Ascomycota; Sordariomycetes; unidentified; unidentified; unidentified; Sordariomycetes_sp
New.ReferenceOTU6548	0	1	0	0	0	0	0	0	0	0	0	0	0	0	0	0	0	0	0	0	0	0	0	0	0	0	0	0	3	0	Fungi; Ascomycota; Sordariomycetes; unidentified; unidentified; unidentified; Sordariomycetes_sp
New.ReferenceOTU6568	0	0	0	0	0	0	0	0	0	0	0	1	0	0	0	0	0	0	0	0	0	0	0	0	0	1	0	0	1	0	Fungi; Ascomycota; Sordariomycetes; unidentified; unidentified; unidentified; Sordariomycetes_sp
New.ReferenceOTU6690	0	0	0	0	0	0	0	0	2	0	0	0	0	0	0	0	0	0	0	0	0	0	0	0	0	0	0	0	0	0	Fungi; Ascomycota; Sordariomycetes; unidentified; unidentified; unidentified; Sordariomycetes_sp
New.ReferenceOTU6768	0	0	0	0	0	0	0	0	0	0	2	0	0	0	0	0	0	0	0	0	0	0	0	0	0	0	0	0	0	0	Fungi; Ascomycota; Sordariomycetes; unidentified; unidentified; unidentified; Sordariomycetes_sp
New.ReferenceOTU6901	0	0	0	0	0	0	0	0	5	0	0	0	0	0	0	0	0	0	0	0	0	0	0	0	0	0	0	0	0	0	Fungi; Ascomycota; Sordariomycetes; unidentified; unidentified; unidentified; Sordariomycetes_sp
New.ReferenceOTU7088	0	0	0	0	0	0	0	0	0	0	0	0	0	0	0	0	0	0	0	0	0	1	0	0	0	0	1	0	0	0	Fungi; Ascomycota; Sordariomycetes; unidentified; unidentified; unidentified; Sordariomycetes_sp
New.ReferenceOTU7092	0	0	0	0	0	0	0	0	0	0	0	0	0	0	0	0	0	11	0	0	0	0	0	0	0	0	0	0	0	0	Fungi; Ascomycota; Sordariomycetes; unidentified; unidentified; unidentified; Sordariomycetes_sp
New.ReferenceOTU7105	0	0	0	0	0	0	0	1	0	0	0	0	0	0	0	0	0	0	0	0	0	0	0	0	0	0	0	0	0	0	Fungi; Ascomycota; Sordariomycetes; unidentified; unidentified; unidentified; Sordariomycetes_sp
New.ReferenceOTU725	0	0	0	0	0	0	0	0	0	0	0	0	0	0	0	0	0	0	0	5	0	0	0	0	0	0	0	0	0	0	Fungi; Ascomycota; Sordariomycetes; unidentified; unidentified; unidentified; Sordariomycetes_sp
New.ReferenceOTU7420	0	0	0	0	0	0	0	0	0	0	0	0	0	0	0	0	0	0	0	0	0	0	0	36	0	0	0	0	0	0	Fungi; Ascomycota; Sordariomycetes; unidentified; unidentified; unidentified; Sordariomycetes_sp
New.ReferenceOTU7470	0	0	0	0	0	0	0	0	0	0	0	0	0	0	0	0	0	0	0	0	0	0	0	3	0	0	0	0	0	0	Fungi; Ascomycota; Sordariomycetes; unidentified; unidentified; unidentified; Sordariomycetes_sp
New.ReferenceOTU7570	0	0	0	0	0	0	0	0	0	0	0	0	0	0	0	0	0	0	0	1	0	0	0	0	0	0	0	0	0	0	Fungi; Ascomycota; Sordariomycetes; unidentified; unidentified; unidentified; Sordariomycetes_sp
New.ReferenceOTU767	0	0	0	0	0	0	0	0	0	0	0	0	0	0	1	0	0	0	0	0	0	0	0	0	0	0	0	0	0	0	Fungi; Ascomycota; Sordariomycetes; unidentified; unidentified; unidentified; Sordariomycetes_sp
New.ReferenceOTU7708	0	0	0	0	0	0	0	0	0	0	0	1	0	0	0	0	0	0	0	0	0	0	0	0	0	0	0	0	0	0	Fungi; Ascomycota; Sordariomycetes; unidentified; unidentified; unidentified; Sordariomycetes_sp
New.ReferenceOTU78	0	0	0	0	0	0	0	0	0	0	0	0	0	0	0	0	0	0	0	0	1	0	0	0	0	0	1	0	0	0	Fungi; Ascomycota; Sordariomycetes; unidentified; unidentified; unidentified; Sordariomycetes_sp
New.ReferenceOTU7916	0	0	0	0	0	0	0	0	0	0	0	0	0	0	0	0	0	0	0	0	0	0	0	2	0	0	0	0	0	0	Fungi; Ascomycota; Sordariomycetes; unidentified; unidentified; unidentified; Sordariomycetes_sp
New.ReferenceOTU822	0	0	0	0	0	0	0	0	0	0	0	0	0	0	0	2	0	0	0	0	0	0	0	0	0	0	0	0	0	0	Fungi; Ascomycota; Sordariomycetes; unidentified; unidentified; unidentified; Sordariomycetes_sp
New.ReferenceOTU841	0	0	0	1	0	0	0	0	0	0	0	0	0	0	0	0	0	0	0	0	0	0	0	0	0	0	0	0	0	0	Fungi; Ascomycota; Sordariomycetes; unidentified; unidentified; unidentified; Sordariomycetes_sp
New.ReferenceOTU980	0	0	0	0	0	0	0	0	0	1	5	0	0	0	4	0	0	0	1	0	1	0	0	0	0	0	0	0	0	0	Fungi; Ascomycota; Sordariomycetes; unidentified; unidentified; unidentified; Sordariomycetes_sp
SH021247.06FU_AY273301_reps_singleton	0	0	2	0	2	0	0	0	0	2	0	0	0	1	1	1	2	0	1	7	0	0	0	8	0	0	9	5	0	3	Fungi; Ascomycota; Sordariomycetes; unidentified; unidentified; unidentified; Sordariomycetes_sp
SH022779.06FU_DQ124120_reps_singleton	0	0	0	0	0	0	1	0	0	0	0	0	0	0	0	0	0	0	0	0	0	0	0	0	0	0	0	0	0	0	Fungi; Ascomycota; Sordariomycetes; unidentified; unidentified; unidentified; Sordariomycetes_sp
SH091852.06FU_EU686840_reps	0	0	0	0	0	0	0	0	0	0	0	0	0	0	0	0	0	0	0	0	0	1	0	0	1	0	0	0	0	0	Fungi; Ascomycota; Sordariomycetes; unidentified; unidentified; unidentified; Sordariomycetes_sp
SH093388.06FU_EU687187_reps	0	0	0	0	1	0	0	0	0	0	0	0	0	0	0	1	0	0	0	0	0	0	0	0	0	0	0	0	0	0	Fungi; Ascomycota; Sordariomycetes; unidentified; unidentified; unidentified; Sordariomycetes_sp
SH112678.06FU_EU686846_reps	0	0	0	0	0	0	0	0	0	0	0	0	0	0	1	0	0	0	0	0	0	0	0	0	0	0	0	0	0	0	Fungi; Ascomycota; Sordariomycetes; unidentified; unidentified; unidentified; Sordariomycetes_sp
SH124199.06FU_JN943630_reps	0	1	0	0	4	0	1	0	0	1	0	2	0	0	0	0	0	0	0	0	0	0	0	0	0	0	0	0	0	0	Fungi; Ascomycota; Sordariomycetes; unidentified; unidentified; unidentified; Sordariomycetes_sp
SH129545.06FU_JQ760218_reps	0	0	3	3	1	19	13	0	0	1	0	11	4	6	4	6	5	1	0	0	1	7	0	9	2	0	1	9	0	6	Fungi; Ascomycota; Sordariomycetes; unidentified; unidentified; unidentified; Sordariomycetes_sp
SH137917.06FU_KC748301_reps	0	0	0	0	0	0	0	0	0	0	0	0	0	0	0	0	0	0	0	0	0	0	0	0	0	0	1	2	0	2	Fungi; Ascomycota; Sordariomycetes; unidentified; unidentified; unidentified; Sordariomycetes_sp
SH138708.06FU_FJ624265_reps	0	0	0	0	0	0	0	0	1	1	0	0	0	1	0	0	2	0	0	0	0	0	0	0	0	0	0	0	0	0	Fungi; Ascomycota; Sordariomycetes; unidentified; unidentified; unidentified; Sordariomycetes_sp
SH140038.06FU_JQ425380_reps	1	0	0	0	0	0	0	0	0	0	0	0	0	0	3	0	0	0	0	0	0	0	0	0	0	0	0	0	0	0	Fungi; Ascomycota; Sordariomycetes; unidentified; unidentified; unidentified; Sordariomycetes_sp
SH141766.06FU_KC507201_reps	0	0	0	0	0	0	0	0	0	0	0	0	0	0	0	0	0	1	0	0	2	0	1	0	0	0	0	1	0	0	Fungi; Ascomycota; Sordariomycetes; unidentified; unidentified; unidentified; Sordariomycetes_sp
SH142001.06FU_DQ993641_reps	0	0	1	0	0	0	0	1	0	0	0	0	0	0	0	0	0	0	0	0	0	0	0	0	0	0	0	0	0	0	Fungi; Ascomycota; Sordariomycetes; unidentified; unidentified; unidentified; Sordariomycetes_sp
SH142003.06FU_JX624289_reps	0	0	0	0	0	1	0	1	0	1	1	0	0	0	0	1	0	0	0	0	0	0	0	0	0	0	0	0	0	0	Fungi; Ascomycota; Sordariomycetes; unidentified; unidentified; unidentified; Sordariomycetes_sp
SH450761.06FU_EU977288_reps_singleton	0	0	0	0	2	0	0	0	0	0	0	0	0	0	0	0	0	0	0	0	0	0	0	0	0	0	0	0	0	0	Fungi; Ascomycota; Sordariomycetes; unidentified; unidentified; unidentified; Sordariomycetes_sp
New.ReferenceOTU4031	0	0	0	2	0	0	0	0	0	0	0	0	0	0	0	0	0	0	0	0	0	0	0	0	0	0	0	0	0	0	Fungi; Ascomycota; Sordariomycetes; unidentified; unidentified; unidentified; Sordariomycetes_sp_11155
New.ReferenceOTU1119	0	0	0	0	0	0	0	0	0	0	0	0	1	0	0	0	0	0	0	0	0	0	0	0	0	0	0	0	0	0	Fungi; Ascomycota; Sordariomycetes; unidentified; unidentified; unidentified; Sordariomycetes_sp_genotype_155
New.ReferenceOTU5410	0	0	0	0	0	0	0	0	0	0	0	0	0	0	0	0	1	0	0	0	0	0	0	0	0	0	0	0	0	0	Fungi; Ascomycota; Sordariomycetes; unidentified; unidentified; unidentified; Sordariomycetes_sp_genotype_155
New.ReferenceOTU5586	0	0	0	0	0	0	0	0	0	0	1	0	0	0	0	0	0	0	5	0	0	0	0	0	0	0	0	0	0	0	Fungi; Ascomycota; Sordariomycetes; unidentified; unidentified; unidentified; Sordariomycetes_sp_genotype_155
New.ReferenceOTU5222	0	0	0	0	0	0	0	0	0	0	0	0	0	0	0	0	0	2	0	0	0	0	0	0	0	0	0	0	0	0	Fungi; Ascomycota; Sordariomycetes; unidentified; unidentified; unidentified; Sordariomycetes_sp_genotype_250
New.ReferenceOTU819	0	0	0	0	0	0	0	0	0	0	0	0	0	0	0	0	0	0	0	0	1	0	0	0	0	0	0	0	0	1	Fungi; Ascomycota; Sordariomycetes; unidentified; unidentified; unidentified; Sordariomycetes_sp_genotype_306
New.ReferenceOTU1924	0	0	0	0	0	0	0	0	0	0	0	0	1	0	0	0	0	0	0	0	0	0	1	0	0	0	2	0	0	7	Fungi; Ascomycota; Sordariomycetes; unidentified; unidentified; unidentified; Sordariomycetes_sp_genotype_394
SH014444.06FU_JQ760548_reps_singleton	0	0	0	0	0	0	0	0	0	0	0	0	0	0	0	0	0	0	0	0	0	0	0	0	0	0	0	0	0	1	Fungi; Ascomycota; Sordariomycetes; unidentified; unidentified; unidentified; Sordariomycetes_sp_genotype_406
New.ReferenceOTU5538	1	0	0	0	0	0	0	0	0	0	0	0	0	0	0	0	0	0	0	0	0	0	0	6	0	0	0	0	0	0	Fungi; Ascomycota; Sordariomycetes; unidentified; unidentified; unidentified; Sordariomycetes_sp_genotype_440
New.ReferenceOTU4682	0	0	0	0	0	0	0	1	0	0	1	0	0	0	1	1	0	0	0	0	0	0	0	1	0	0	0	0	0	0	Fungi; Ascomycota; Sordariomycetes; unidentified; unidentified; unidentified; Sordariomycetes_sp_genotype_455
New.ReferenceOTU3053	0	0	0	0	0	0	0	0	0	0	0	0	0	0	0	0	0	0	0	0	1	0	0	0	0	0	0	0	0	0	Fungi; Ascomycota; Sordariomycetes; unidentified; unidentified; unidentified; Sordariomycetes_sp_genotype_530
New.ReferenceOTU352	0	1	0	0	0	0	0	0	0	0	0	0	0	0	0	0	0	0	0	0	0	0	0	0	0	0	0	0	0	0	Fungi; Ascomycota; Sordariomycetes; unidentified; unidentified; unidentified; Sordariomycetes_sp_genotype_530
New.ReferenceOTU4379	0	0	3	0	0	6	6	0	0	0	0	0	0	0	0	0	0	0	0	0	0	0	1	0	0	0	0	0	0	1	Fungi; Ascomycota; Sordariomycetes; unidentified; unidentified; unidentified; Sordariomycetes_sp_genotype_83
SH016585.06FU_JQ758986_reps_singleton	0	0	1	0	0	0	0	0	0	0	0	0	0	0	0	0	1	0	0	0	0	0	2	0	0	0	0	1	0	0	Fungi; Ascomycota; Sordariomycetes; unidentified; unidentified; unidentified; Sordariomycetes_sp_genotype_83
New.ReferenceOTU2660	0	1	0	0	0	0	0	0	0	0	0	0	0	0	1	0	0	0	0	0	0	0	0	0	0	0	0	0	0	0	Fungi; Ascomycota; Sordariomycetes; unidentified; unidentified; unidentified; Sordariomycetes_sp_HH56
New.ReferenceOTU879	0	0	0	0	0	0	0	0	0	0	1	0	0	0	0	0	0	0	0	0	0	0	0	0	0	0	0	0	0	0	Fungi; Ascomycota; Sordariomycetes; unidentified; unidentified; unidentified; Sordariomycetes_sp_JDCH2
SH016178.06FU_EU482234_reps_singleton	0	0	0	0	0	0	0	1	0	1	1	1	0	0	0	0	0	0	0	1	0	0	0	0	0	0	0	0	0	0	Fungi; Ascomycota; Sordariomycetes; unidentified; unidentified; unidentified; Sordariomycetes_sp_SAB_2009a
SH112679.06FU_EU687180_reps	0	0	4	1	0	2	2	1	0	4	1	0	0	0	0	1	0	0	0	1	0	0	1	0	0	1	0	0	0	1	Fungi; Ascomycota; Sordariomycetes; unidentified; unidentified; unidentified; Sordariomycetes_sp_SAB_2009a
New.ReferenceOTU2926	0	0	0	0	0	0	0	0	0	0	0	0	0	0	0	0	0	3	0	0	0	0	0	0	0	0	0	0	0	0	Fungi; Ascomycota; Sordariomycetes; unidentified; unidentified; unidentified; uncultured_Myrmecridium
SH110105.06FU_EU030327_reps	0	0	0	0	0	2	0	0	0	0	0	0	0	0	0	0	0	0	0	0	0	0	0	0	0	0	0	0	0	0	Fungi; Ascomycota; Sordariomycetes; Xylariales; Amphisphaeriaceae; unidentified; Amphisphaeriaceae_sp
New.ReferenceOTU686	0	1	0	1	0	0	0	0	0	0	0	0	0	0	0	0	0	1	0	0	0	0	0	0	0	0	0	0	0	0	Fungi; Ascomycota; Sordariomycetes; Xylariales; unidentified; unidentified; uncultured_Hansfordia
SH023368.06FU_KC785554_reps_singleton	0	0	0	0	0	0	0	0	0	0	0	0	0	0	0	0	0	0	0	0	0	0	0	0	0	0	0	1	0	0	Fungi; Ascomycota; Sordariomycetes; Xylariales; unidentified; unidentified; uncultured_Hansfordia
New.ReferenceOTU5730	0	0	0	0	0	0	0	0	0	0	0	3	0	0	0	0	0	0	0	0	0	0	0	0	0	0	0	0	0	0	Fungi; Ascomycota; Sordariomycetes; Xylariales; unidentified; unidentified; uncultured_Xylariales
SH021031.06FU_EF619915_reps_singleton	0	0	0	0	0	4	0	0	0	0	0	1	0	0	0	0	0	0	0	0	0	0	0	0	0	0	0	0	0	0	Fungi; Ascomycota; Sordariomycetes; Xylariales; unidentified; unidentified; uncultured_Xylariales
New.ReferenceOTU1193	0	0	0	0	0	0	1	0	0	0	1	0	0	0	0	0	0	0	0	0	0	0	0	0	0	0	0	0	0	1	Fungi; Ascomycota; Sordariomycetes; Xylariales; unidentified; unidentified; Xylariales_BPV101a
New.ReferenceOTU3972	0	0	0	0	0	0	0	0	0	0	0	0	0	0	0	0	0	0	0	0	0	0	1	0	0	0	0	0	0	1	Fungi; Ascomycota; Sordariomycetes; Xylariales; unidentified; unidentified; Xylariales_BPV101a
New.ReferenceOTU994	0	0	0	0	0	0	0	0	0	0	1	0	0	0	0	0	0	0	0	0	0	0	0	0	0	0	0	0	0	0	Fungi; Ascomycota; Sordariomycetes; Xylariales; unidentified; unidentified; Xylariales_BPV101a
New.ReferenceOTU1140	1	0	0	0	1	0	0	0	0	0	2	0	0	0	0	0	0	0	0	0	0	0	0	0	0	0	0	0	0	0	Fungi; Ascomycota; Sordariomycetes; Xylariales; unidentified; unidentified; Xylariales_sp
New.ReferenceOTU1326	0	0	0	0	1	0	0	0	0	0	0	0	0	0	0	0	0	0	0	0	0	0	0	0	0	0	0	0	0	0	Fungi; Ascomycota; Sordariomycetes; Xylariales; unidentified; unidentified; Xylariales_sp
New.ReferenceOTU1651	0	0	0	0	0	0	0	0	1	0	0	0	0	0	0	0	0	0	0	0	0	0	0	0	0	0	0	0	0	0	Fungi; Ascomycota; Sordariomycetes; Xylariales; unidentified; unidentified; Xylariales_sp
New.ReferenceOTU168	0	0	0	0	0	0	0	0	0	0	0	0	0	0	0	0	1	0	0	0	0	0	0	0	0	0	0	0	0	0	Fungi; Ascomycota; Sordariomycetes; Xylariales; unidentified; unidentified; Xylariales_sp
New.ReferenceOTU1892	0	0	0	0	1	0	0	0	0	0	0	0	0	0	0	0	0	0	0	0	6	0	0	0	0	0	0	0	0	1	Fungi; Ascomycota; Sordariomycetes; Xylariales; unidentified; unidentified; Xylariales_sp
New.ReferenceOTU1927	1	0	0	0	0	0	0	0	0	0	0	0	0	0	0	1	4	0	0	1	0	1	0	0	0	0	0	0	0	0	Fungi; Ascomycota; Sordariomycetes; Xylariales; unidentified; unidentified; Xylariales_sp
New.ReferenceOTU2165	0	0	0	1	0	0	0	0	0	0	0	0	0	0	0	0	0	0	0	0	0	0	0	0	0	0	0	0	0	0	Fungi; Ascomycota; Sordariomycetes; Xylariales; unidentified; unidentified; Xylariales_sp
New.ReferenceOTU2173	0	0	0	0	1	0	0	0	0	0	0	0	0	0	0	0	0	0	0	0	0	0	0	0	0	0	0	0	0	0	Fungi; Ascomycota; Sordariomycetes; Xylariales; unidentified; unidentified; Xylariales_sp
New.ReferenceOTU2305	0	0	0	1	0	0	0	0	0	0	0	0	0	0	0	0	0	0	0	0	0	0	0	0	0	0	0	0	0	0	Fungi; Ascomycota; Sordariomycetes; Xylariales; unidentified; unidentified; Xylariales_sp
New.ReferenceOTU2337	0	0	0	0	0	0	0	0	0	0	0	0	0	0	0	0	0	0	2	0	0	0	0	0	0	0	0	0	0	0	Fungi; Ascomycota; Sordariomycetes; Xylariales; unidentified; unidentified; Xylariales_sp
New.ReferenceOTU2592	0	0	1	0	0	0	0	0	0	0	0	0	0	0	0	0	0	0	0	0	0	0	0	0	0	0	0	0	0	0	Fungi; Ascomycota; Sordariomycetes; Xylariales; unidentified; unidentified; Xylariales_sp
New.ReferenceOTU2744	0	0	0	0	0	0	0	0	0	0	0	0	0	0	0	0	0	0	1	0	0	0	0	0	0	0	0	0	0	0	Fungi; Ascomycota; Sordariomycetes; Xylariales; unidentified; unidentified; Xylariales_sp
New.ReferenceOTU2805	0	0	0	0	0	0	0	0	0	0	0	0	0	1	0	0	0	0	0	0	0	0	0	0	0	0	0	0	0	0	Fungi; Ascomycota; Sordariomycetes; Xylariales; unidentified; unidentified; Xylariales_sp
New.ReferenceOTU3190	1	0	0	0	0	0	0	0	0	0	0	0	1	1	0	0	0	0	0	0	0	0	0	1	0	0	0	0	0	0	Fungi; Ascomycota; Sordariomycetes; Xylariales; unidentified; unidentified; Xylariales_sp
New.ReferenceOTU4226	0	0	0	0	1	0	0	0	1	0	0	0	0	0	0	0	2	0	0	4	0	0	0	0	0	0	0	0	0	0	Fungi; Ascomycota; Sordariomycetes; Xylariales; unidentified; unidentified; Xylariales_sp
New.ReferenceOTU4302	0	0	0	1	0	0	0	0	0	0	0	0	0	0	0	0	0	0	0	0	0	0	0	0	0	0	0	0	0	0	Fungi; Ascomycota; Sordariomycetes; Xylariales; unidentified; unidentified; Xylariales_sp
New.ReferenceOTU443	0	0	0	0	0	0	0	0	0	0	0	0	0	0	0	0	0	0	0	0	0	0	0	0	0	0	0	0	0	2	Fungi; Ascomycota; Sordariomycetes; Xylariales; unidentified; unidentified; Xylariales_sp
New.ReferenceOTU4872	0	0	0	0	0	0	0	0	0	8	0	0	3	7	3	1	1	0	0	0	0	0	0	3	0	1	0	0	0	0	Fungi; Ascomycota; Sordariomycetes; Xylariales; unidentified; unidentified; Xylariales_sp
New.ReferenceOTU4937	0	0	1	0	0	0	0	0	3	1	3	1	0	1	3	0	3	0	1	0	1	0	0	0	0	0	2	0	0	0	Fungi; Ascomycota; Sordariomycetes; Xylariales; unidentified; unidentified; Xylariales_sp
New.ReferenceOTU5041	0	0	0	1	0	0	0	0	0	0	0	0	0	0	0	0	0	0	0	0	0	0	0	0	0	0	0	0	0	0	Fungi; Ascomycota; Sordariomycetes; Xylariales; unidentified; unidentified; Xylariales_sp
New.ReferenceOTU5331	0	0	0	0	0	0	0	0	1	0	0	0	0	0	0	0	0	0	0	0	0	0	0	0	0	0	0	0	0	0	Fungi; Ascomycota; Sordariomycetes; Xylariales; unidentified; unidentified; Xylariales_sp
New.ReferenceOTU5648	0	0	0	0	0	0	0	0	0	0	0	0	0	0	0	0	0	0	1	0	0	0	0	0	0	0	0	0	0	0	Fungi; Ascomycota; Sordariomycetes; Xylariales; unidentified; unidentified; Xylariales_sp
New.ReferenceOTU5683	0	0	0	0	0	0	0	0	0	0	0	0	0	0	0	0	0	0	0	0	1	0	2	0	1	0	0	0	0	0	Fungi; Ascomycota; Sordariomycetes; Xylariales; unidentified; unidentified; Xylariales_sp
New.ReferenceOTU5831	0	0	0	0	0	0	1	0	0	0	1	0	0	0	0	5	0	0	1	0	3	0	0	0	0	0	0	0	1	0	Fungi; Ascomycota; Sordariomycetes; Xylariales; unidentified; unidentified; Xylariales_sp
New.ReferenceOTU5982	0	0	0	0	0	0	0	0	0	0	0	0	0	0	0	0	0	0	0	0	1	0	1	0	0	0	0	0	0	0	Fungi; Ascomycota; Sordariomycetes; Xylariales; unidentified; unidentified; Xylariales_sp
New.ReferenceOTU6099	0	0	0	0	0	0	0	0	0	0	0	0	0	0	0	0	0	8	0	0	0	0	0	0	0	0	0	0	0	0	Fungi; Ascomycota; Sordariomycetes; Xylariales; unidentified; unidentified; Xylariales_sp
New.ReferenceOTU638	0	0	0	0	0	0	0	0	0	0	0	0	0	0	0	0	0	0	0	0	19	1	0	0	0	0	0	2	0	1	Fungi; Ascomycota; Sordariomycetes; Xylariales; unidentified; unidentified; Xylariales_sp
New.ReferenceOTU6382	0	0	0	0	0	0	0	0	0	0	0	0	0	0	0	0	0	2	0	0	0	0	0	0	0	0	0	0	0	0	Fungi; Ascomycota; Sordariomycetes; Xylariales; unidentified; unidentified; Xylariales_sp
New.ReferenceOTU6499	0	0	0	0	0	0	0	0	0	0	0	0	0	0	0	0	0	0	0	0	0	0	0	0	0	0	0	0	0	5	Fungi; Ascomycota; Sordariomycetes; Xylariales; unidentified; unidentified; Xylariales_sp
New.ReferenceOTU6615	0	0	0	0	0	0	0	0	0	0	0	2	0	0	0	0	0	0	0	0	0	0	0	0	0	0	0	0	0	0	Fungi; Ascomycota; Sordariomycetes; Xylariales; unidentified; unidentified; Xylariales_sp
New.ReferenceOTU6704	0	0	0	0	0	0	0	0	0	0	0	0	0	0	0	0	0	0	0	4	0	0	0	0	0	0	0	0	0	0	Fungi; Ascomycota; Sordariomycetes; Xylariales; unidentified; unidentified; Xylariales_sp
New.ReferenceOTU7031	0	0	0	0	0	0	0	0	0	0	0	0	1	0	0	0	0	0	0	0	0	0	0	0	0	0	1	0	0	0	Fungi; Ascomycota; Sordariomycetes; Xylariales; unidentified; unidentified; Xylariales_sp
New.ReferenceOTU7203	0	0	0	0	0	0	0	0	0	0	1	0	0	0	0	0	0	0	0	0	0	0	0	0	0	0	0	0	0	0	Fungi; Ascomycota; Sordariomycetes; Xylariales; unidentified; unidentified; Xylariales_sp
New.ReferenceOTU7208	0	0	0	0	0	0	0	0	0	0	0	0	0	0	0	0	1	0	0	0	0	0	0	0	0	0	0	0	0	0	Fungi; Ascomycota; Sordariomycetes; Xylariales; unidentified; unidentified; Xylariales_sp
New.ReferenceOTU7427	0	0	0	0	0	0	0	0	0	0	0	0	0	0	0	0	0	0	1	0	0	0	0	0	0	0	0	0	0	0	Fungi; Ascomycota; Sordariomycetes; Xylariales; unidentified; unidentified; Xylariales_sp

New.ReferenceOTU7646	0	0	0	0	0	0	0	0	0	0	0	0	0	0	0	0	0	0	1	0	0	0	0	0	0	0	0	0	0	0	Fungi; Ascomycota; Sordariomycetes; Xylariales; unidentified; unidentified; Xylariales_sp
New.ReferenceOTU7813	0	0	0	0	0	0	0	0	0	0	0	0	0	0	0	0	0	0	0	0	0	0	0	0	0	0	0	0	2	0	Fungi; Ascomycota; Sordariomycetes; Xylariales; unidentified; unidentified; Xylariales_sp
New.ReferenceOTU7814	0	0	0	0	0	0	0	0	0	0	0	0	0	0	0	0	0	0	1	0	0	0	0	0	0	0	0	0	0	0	Fungi; Ascomycota; Sordariomycetes; Xylariales; unidentified; unidentified; Xylariales_sp
New.ReferenceOTU806	0	0	0	1	0	0	0	0	0	0	0	0	0	0	0	0	1	6	5	5	1	0	0	1	0	1	0	2	1	0	Fungi; Ascomycota; Sordariomycetes; Xylariales; unidentified; unidentified; Xylariales_sp
New.ReferenceOTU99	0	0	0	0	0	0	0	0	0	0	0	0	0	0	0	0	0	0	0	0	0	0	1	0	0	0	0	0	0	0	Fungi; Ascomycota; Sordariomycetes; Xylariales; unidentified; unidentified; Xylariales_sp
SH018571.06FU_FJ612826_reps_singleton	0	0	0	0	0	0	0	0	0	0	0	0	0	0	0	0	0	0	0	0	0	0	0	0	0	1	0	0	0	0	Fungi; Ascomycota; Sordariomycetes; Xylariales; unidentified; unidentified; Xylariales_sp
SH031027.06FU_AF502847_reps_singleton	0	0	0	0	0	0	0	0	0	0	0	0	0	2	0	0	0	0	0	0	0	0	0	0	0	0	0	0	0	0	Fungi; Ascomycota; Sordariomycetes; Xylariales; unidentified; unidentified; Xylariales_sp
SH089417.06FU_KF435571_reps	0	0	0	0	0	0	0	0	0	0	0	0	0	0	0	0	0	0	0	0	0	0	0	1	0	0	0	0	0	0	Fungi; Ascomycota; Sordariomycetes; Xylariales; unidentified; unidentified; Xylariales_sp
SH112498.06FU_EU686847_reps	0	0	0	4	0	0	0	0	0	0	0	0	0	0	0	0	0	0	0	0	0	0	0	0	0	0	0	0	0	0	Fungi; Ascomycota; Sordariomycetes; Xylariales; unidentified; unidentified; Xylariales_sp
SH116011.06FU_AY699694_reps	0	0	0	0	0	0	0	0	1	0	0	0	0	0	0	0	0	0	0	0	0	0	0	0	0	0	0	0	0	0	Fungi; Ascomycota; Sordariomycetes; Xylariales; unidentified; unidentified; Xylariales_sp
SH118132.06FU_JN890346_reps	1	0	0	2	0	0	0	0	0	0	0	0	0	0	0	0	0	0	0	0	0	0	0	0	0	0	0	0	0	0	Fungi; Ascomycota; Sordariomycetes; Xylariales; unidentified; unidentified; Xylariales_sp
SH118136.06FU_JN890321_reps	0	0	1	0	0	0	0	0	0	0	0	0	0	0	0	1	0	0	0	0	0	0	0	0	0	0	0	0	0	0	Fungi; Ascomycota; Sordariomycetes; Xylariales; unidentified; unidentified; Xylariales_sp
SH120767.06FU_AF502895_reps	0	0	0	0	0	0	0	0	0	0	0	0	0	0	0	0	0	0	0	4	0	0	0	0	0	0	0	0	0	0	Fungi; Ascomycota; Sordariomycetes; Xylariales; unidentified; unidentified; Xylariales_sp
SH120768.06FU_KF019266_reps	0	0	2	1	0	0	0	0	1	0	0	0	1	3	0	1	0	0	0	0	0	0	0	1	0	0	0	0	0	0	Fungi; Ascomycota; Sordariomycetes; Xylariales; unidentified; unidentified; Xylariales_sp
SH120769.06FU_JN890115_reps	0	0	0	1	0	0	0	0	0	0	0	0	0	0	0	0	0	0	0	0	0	0	0	0	0	0	0	0	0	0	Fungi; Ascomycota; Sordariomycetes; Xylariales; unidentified; unidentified; Xylariales_sp
New.ReferenceOTU2944	0	0	1	0	0	0	0	0	0	0	0	0	0	0	0	0	0	0	0	0	0	0	1	0	0	0	0	0	0	0	Fungi; Ascomycota; Sordariomycetes; Xylariales; unidentified; unidentified; Xylariales_sp_VegaE1_52
New.CleanUp.ReferenceOTU249	0	0	0	0	0	0	0	0	0	0	0	0	0	0	0	0	0	1	0	0	0	0	0	0	0	0	0	0	0	0	Fungi; Ascomycota; Sordariomycetes; Xylariales; Xylariaceae; unidentified; Xylariaceae_sp
New.ReferenceOTU1122	0	0	1	0	0	0	0	0	0	0	0	0	0	0	0	0	0	0	0	0	0	0	0	0	0	0	0	0	0	0	Fungi; Ascomycota; Sordariomycetes; Xylariales; Xylariaceae; unidentified; Xylariaceae_sp
New.ReferenceOTU1160	0	0	0	0	0	0	1	0	0	0	0	0	0	0	0	0	0	0	0	1	0	0	0	0	0	0	0	0	0	0	Fungi; Ascomycota; Sordariomycetes; Xylariales; Xylariaceae; unidentified; Xylariaceae_sp
New.ReferenceOTU1331	0	0	0	0	0	0	0	0	0	0	1	0	0	0	0	0	0	0	0	0	0	0	0	0	0	0	0	0	0	0	Fungi; Ascomycota; Sordariomycetes; Xylariales; Xylariaceae; unidentified; Xylariaceae_sp
New.ReferenceOTU1935	0	0	0	0	0	0	0	0	0	0	0	0	0	0	0	0	0	0	0	0	0	0	0	0	0	0	0	0	1	0	Fungi; Ascomycota; Sordariomycetes; Xylariales; Xylariaceae; unidentified; Xylariaceae_sp
New.ReferenceOTU1957	0	2	1	0	0	0	0	0	0	0	0	0	0	0	0	0	0	0	0	0	0	0	0	0	0	0	0	0	0	0	Fungi; Ascomycota; Sordariomycetes; Xylariales; Xylariaceae; unidentified; Xylariaceae_sp
New.ReferenceOTU2146	0	0	0	0	0	0	0	0	0	0	0	0	0	0	0	0	0	1	1	0	0	0	0	0	0	0	0	0	0	0	Fungi; Ascomycota; Sordariomycetes; Xylariales; Xylariaceae; unidentified; Xylariaceae_sp
New.ReferenceOTU2276	0	0	0	0	0	0	0	0	0	0	0	0	0	0	0	0	0	8	23	10	0	0	0	0	0	0	0	0	0	0	Fungi; Ascomycota; Sordariomycetes; Xylariales; Xylariaceae; unidentified; Xylariaceae_sp
New.ReferenceOTU2417	0	0	0	0	0	0	0	0	0	0	0	0	0	0	0	0	2	0	0	0	0	0	1	0	0	0	1	0	0	6	Fungi; Ascomycota; Sordariomycetes; Xylariales; Xylariaceae; unidentified; Xylariaceae_sp
New.ReferenceOTU251	0	0	0	0	0	0	0	0	1	0	0	0	0	0	0	0	0	0	0	0	0	0	0	0	0	0	0	0	0	0	Fungi; Ascomycota; Sordariomycetes; Xylariales; Xylariaceae; unidentified; Xylariaceae_sp
New.ReferenceOTU276	0	0	0	0	0	0	0	0	0	0	0	0	0	0	1	0	0	0	0	0	0	1	1	0	0	0	0	0	0	0	Fungi; Ascomycota; Sordariomycetes; Xylariales; Xylariaceae; unidentified; Xylariaceae_sp
New.ReferenceOTU2766	0	0	0	0	0	0	0	0	0	0	0	0	0	0	0	0	0	0	0	1	0	0	0	0	0	0	0	0	0	0	Fungi; Ascomycota; Sordariomycetes; Xylariales; Xylariaceae; unidentified; Xylariaceae_sp
New.ReferenceOTU309	0	0	0	0	0	0	0	0	0	0	0	0	0	0	0	0	0	0	0	0	0	0	0	0	0	0	0	0	1	0	Fungi; Ascomycota; Sordariomycetes; Xylariales; Xylariaceae; unidentified; Xylariaceae_sp
New.ReferenceOTU3115	0	0	0	0	0	0	0	0	0	1	0	0	0	0	0	0	0	0	0	0	0	0	0	0	0	0	0	0	0	0	Fungi; Ascomycota; Sordariomycetes; Xylariales; Xylariaceae; unidentified; Xylariaceae_sp
New.ReferenceOTU3333	0	0	0	0	2	0	0	0	0	0	0	0	0	0	0	0	0	0	0	0	0	0	0	0	0	0	0	0	0	0	Fungi; Ascomycota; Sordariomycetes; Xylariales; Xylariaceae; unidentified; Xylariaceae_sp
New.ReferenceOTU3396	0	0	1	0	0	0	0	0	0	0	0	0	0	0	0	0	0	0	0	0	0	0	0	2	0	0	0	0	0	0	Fungi; Ascomycota; Sordariomycetes; Xylariales; Xylariaceae; unidentified; Xylariaceae_sp
New.ReferenceOTU4087	0	0	0	0	0	0	0	1	0	0	0	0	0	1	0	0	0	0	0	0	1	2	0	0	0	0	0	0	0	0	Fungi; Ascomycota; Sordariomycetes; Xylariales; Xylariaceae; unidentified; Xylariaceae_sp
New.ReferenceOTU4263	0	0	0	0	0	0	0	0	0	0	0	0	0	0	0	0	0	0	0	0	0	0	1	0	0	0	0	0	0	0	Fungi; Ascomycota; Sordariomycetes; Xylariales; Xylariaceae; unidentified; Xylariaceae_sp
New.ReferenceOTU4478	0	0	0	0	2	0	0	0	0	0	0	0	0	0	0	0	0	0	0	0	0	0	0	0	0	0	0	0	0	0	Fungi; Ascomycota; Sordariomycetes; Xylariales; Xylariaceae; unidentified; Xylariaceae_sp
New.ReferenceOTU4496	0	0	0	0	0	0	0	0	0	0	2	0	0	0	0	0	0	0	0	0	0	0	0	0	0	0	0	0	0	0	Fungi; Ascomycota; Sordariomycetes; Xylariales; Xylariaceae; unidentified; Xylariaceae_sp
New.ReferenceOTU4890	0	0	0	0	0	0	0	0	0	0	0	0	0	0	0	0	0	0	1	1	0	0	0	0	0	0	0	0	0	0	Fungi; Ascomycota; Sordariomycetes; Xylariales; Xylariaceae; unidentified; Xylariaceae_sp
New.ReferenceOTU5200	0	0	1	0	0	0	0	0	0	0	0	0	1	2	0	1	0	4	0	0	0	1	0	12	0	0	0	0	1	0	Fungi; Ascomycota; Sordariomycetes; Xylariales; Xylariaceae; unidentified; Xylariaceae_sp
New.ReferenceOTU5563	0	1	0	1	0	0	0	0	0	0	0	0	0	0	0	0	0	0	0	0	0	0	0	0	0	0	0	0	0	0	Fungi; Ascomycota; Sordariomycetes; Xylariales; Xylariaceae; unidentified; Xylariaceae_sp
New.ReferenceOTU602	0	0	0	0	0	0	0	0	0	0	0	1	0	0	0	0	0	0	0	0	0	0	0	0	0	0	0	0	0	0	Fungi; Ascomycota; Sordariomycetes; Xylariales; Xylariaceae; unidentified; Xylariaceae_sp
New.ReferenceOTU6041	0	0	0	0	0	0	0	0	0	0	1	0	0	0	0	0	0	0	0	0	0	0	0	0	0	0	0	0	0	0	Fungi; Ascomycota; Sordariomycetes; Xylariales; Xylariaceae; unidentified; Xylariaceae_sp
New.ReferenceOTU6056	0	0	0	0	0	0	0	1	0	0	0	0	0	0	0	0	0	0	0	0	0	0	0	0	0	0	0	0	0	0	Fungi; Ascomycota; Sordariomycetes; Xylariales; Xylariaceae; unidentified; Xylariaceae_sp
New.ReferenceOTU6072	0	0	0	1	0	0	0	0	0	0	0	0	0	0	0	0	0	1	2	1	0	0	0	0	0	0	0	0	0	0	Fungi; Ascomycota; Sordariomycetes; Xylariales; Xylariaceae; unidentified; Xylariaceae_sp
New.ReferenceOTU6107	0	0	0	0	0	0	0	0	0	0	0	0	0	0	0	0	0	1	0	0	0	0	0	0	0	0	0	0	1	0	Fungi; Ascomycota; Sordariomycetes; Xylariales; Xylariaceae; unidentified; Xylariaceae_sp
New.ReferenceOTU6551	0	0	0	0	0	0	0	1	0	0	0	0	0	0	0	0	0	0	0	0	0	0	0	0	0	0	0	0	0	0	Fungi; Ascomycota; Sordariomycetes; Xylariales; Xylariaceae; unidentified; Xylariaceae_sp
New.ReferenceOTU6778	0	0	0	0	0	0	0	0	0	0	0	0	0	0	0	0	1	0	1	0	1	0	1	0	0	0	0	0	0	0	Fungi; Ascomycota; Sordariomycetes; Xylariales; Xylariaceae; unidentified; Xylariaceae_sp
New.ReferenceOTU6883	0	1	0	3	0	0	0	1	0	0	0	0	0	1	0	1	0	0	0	0	0	0	0	0	0	0	0	2	0	4	Fungi; Ascomycota; Sordariomycetes; Xylariales; Xylariaceae; unidentified; Xylariaceae_sp
New.ReferenceOTU7454	0	0	0	0	0	0	0	0	0	0	0	0	0	0	0	0	0	0	0	0	1	0	3	0	0	0	5	1	0	0	Fungi; Ascomycota; Sordariomycetes; Xylariales; Xylariaceae; unidentified; Xylariaceae_sp
New.ReferenceOTU8036	0	0	0	0	0	0	0	0	0	0	0	1	0	0	0	0	0	0	0	0	1	0	1	0	0	0	0	0	0	1	Fungi; Ascomycota; Sordariomycetes; Xylariales; Xylariaceae; unidentified; Xylariaceae_sp
SH036090.06FU_UDB014465_reps_singleton	0	0	0	7	0	0	0	0	0	0	0	0	0	0	0	0	0	0	0	0	0	0	0	0	0	0	0	0	0	0	Fungi; Ascomycota; Sordariomycetes; Xylariales; Xylariaceae; unidentified; Xylariaceae_sp
SH089403.06FU_AB285482_reps	13	0	0	0	0	0	0	0	0	0	0	2	0	0	0	0	5	0	0	0	0	0	0	0	0	0	0	0	0	0	Fungi; Ascomycota; Sordariomycetes; Xylariales; Xylariaceae; unidentified; Xylariaceae_sp
SH089407.06FU_JX624277_reps	0	0	0	0	1	0	0	0	0	2	0	1	0	0	0	0	0	0	0	0	0	1	0	0	0	0	0	0	0	0	Fungi; Ascomycota; Sordariomycetes; Xylariales; Xylariaceae; unidentified; Xylariaceae_sp
SH089414.06FU_EU678653_reps	0	0	0	0	0	0	1	0	0	0	0	0	0	0	0	0	0	0	0	0	0	0	0	0	0	0	0	0	0	0	Fungi; Ascomycota; Sordariomycetes; Xylariales; Xylariaceae; unidentified; Xylariaceae_sp
SH134638.06FU_AJ390429_reps	0	0	0	0	0	0	0	0	0	0	0	0	0	0	0	0	0	0	0	2	0	0	0	0	0	0	1	0	0	0	Fungi; Ascomycota; Sordariomycetes; Xylariales; Xylariaceae; unidentified; Xylariaceae_sp
SH143107.06FU_HQ316570_reps	0	0	1	0	0	0	0	0	0	0	0	2	0	0	3	0	0	0	0	0	0	0	0	0	0	0	0	0	0	0	Fungi; Ascomycota; Sordariomycetes; Xylariales; Xylariaceae; unidentified; Xylariaceae_sp
New.ReferenceOTU1879	0	0	0	0	0	0	1	0	0	0	0	0	0	0	0	0	0	0	0	0	0	0	0	0	0	0	0	0	0	0	Fungi; Ascomycota; Sordariomycetes; Xylariales; Xylariaceae; unidentified; Xylariaceae_sp_1_ICMP_18791
New.ReferenceOTU3985	0	0	0	0	0	0	0	0	0	0	0	1	0	0	0	0	0	0	0	0	0	0	0	0	0	0	0	0	0	0	Fungi; Ascomycota; Sordariomycetes; Xylariales; Xylariaceae; unidentified; Xylariaceae_sp_1_ICMP_18791
New.ReferenceOTU7533	1	0	0	0	0	0	0	0	0	0	0	0	0	0	0	0	0	0	0	0	0	0	0	0	0	0	0	0	0	3	Fungi; Ascomycota; Sordariomycetes; Xylariales; Xylariaceae; unidentified; Xylariaceae_sp_18_MLS_2010
New.ReferenceOTU5446	0	0	0	0	0	0	0	0	0	0	1	0	0	0	0	0	0	0	0	0	0	0	0	0	0	0	0	0	0	0	Fungi; Ascomycota; Sordariomycetes; Xylariales; Xylariaceae; unidentified; Xylariaceae_sp_4Y_Pt10_3
New.ReferenceOTU4940	0	0	0	0	0	1	1	0	0	0	0	0	0	0	2	0	0	0	0	0	0	1	0	0	0	0	5	1	0	0	Fungi; Ascomycota; Sordariomycetes; Xylariales; Xylariaceae; unidentified; Xylariaceae_sp_M36_XS_2012
New.ReferenceOTU37	0	0	0	0	0	0	0	0	0	0	0	0	0	0	0	0	0	0	1	0	0	0	0	0	0	0	0	0	0	0	Fungi; Ascomycota; Sordariomycetes; Xylariales; Xylariaceae; unidentified; Xylariaceae_sp_OTU16
SH002639.06FU_FJ425672_reps_singleton	0	0	0	0	0	0	0	0	0	0	0	0	0	0	0	0	0	1	0	0	0	0	1	0	0	0	0	0	0	0	Fungi; Ascomycota; Sordariomycetes; Xylariales; Xylariaceae; unidentified; Xylariaceae_sp_OTU4
New.ReferenceOTU6924	0	0	0	0	0	0	0	0	0	0	0	0	0	0	0	0	0	0	0	2	0	0	0	0	0	0	0	0	0	0	Fungi; Ascomycota; Sordariomycetes; Xylariales; Xylariaceae; unidentified; Xylariaceae_sp_Vega005
SH036207.06FU_EF632079_reps_singleton	0	0	0	0	0	0	0	0	0	0	0	0	1	4	1	1	0	0	0	0	0	0	0	0	0	0	0	0	0	0	Fungi; Ascomycota; unidentified; unidentified; unidentified; unidentified; ascomycete_sp_CRI252
New.ReferenceOTU2576	0	0	0	0	0	0	0	0	0	0	0	0	0	0	10	0	0	0	0	0	0	0	0	0	1	0	0	0	0	0	Fungi; Ascomycota; unidentified; unidentified; unidentified; unidentified; ascomycete_sp_E133
New.ReferenceOTU4904	0	0	0	0	0	0	0	0	0	0	0	0	0	0	0	0	0	0	0	0	0	0	0	0	0	0	0	1	0	0	Fungi; Ascomycota; unidentified; unidentified; unidentified; unidentified; ascomycete_sp_IZ_1111
New.ReferenceOTU1835	0	0	0	0	0	0	0	0	0	2	0	0	0	0	0	1	0	0	0	0	0	0	0	0	0	0	0	0	0	0	Fungi; Ascomycota; unidentified; unidentified; unidentified; unidentified; ascomycete_sp_olrim78
New.ReferenceOTU4657	0	0	0	0	0	0	0	0	0	0	0	0	0	0	0	0	0	0	0	0	0	0	0	0	0	0	0	2	0	0	Fungi; Ascomycota; unidentified; unidentified; unidentified; unidentified; ascomycete_sp_olrim78
New.ReferenceOTU6527	0	8	2	0	0	0	0	0	0	1	1	0	0	0	0	0	0	0	1	0	0	0	0	0	0	0	0	0	3	0	Fungi; Ascomycota; unidentified; unidentified; unidentified; unidentified; ascomycete_sp_olrim78
New.ReferenceOTU7991	0	1	0	0	0	0	0	0	0	0	0	0	0	0	0	0	0	0	0	2	0	0	0	0	0	0	0	0	0	0	Fungi; Ascomycota; unidentified; unidentified; unidentified; unidentified; ascomycete_sp_olrim78
New.ReferenceOTU634	0	0	0	0	0	0	0	0	0	0	0	0	0	0	0	0	0	0	1	0	0	0	0	0	0	0	0	0	0	0	Fungi; Ascomycota; unidentified; unidentified; unidentified; unidentified; ascomycete_sp_olrim79
New.CleanUp.ReferenceOTU1517	0	0	0	0	0	0	0	0	0	0	0	0	0	0	0	0	0	0	0	1	0	0	0	0	0	0	0	0	0	0	Fungi; Ascomycota; unidentified; unidentified; unidentified; unidentified; Ascomycota_sp
New.ReferenceOTU1461	0	1	0	0	0	0	0	0	0	0	0	0	0	1	0	0	0	0	0	0	0	0	0	0	0	0	0	0	0	0	Fungi; Ascomycota; unidentified; unidentified; unidentified; unidentified; Ascomycota_sp
New.ReferenceOTU1625	2	0	2	4	0	0	2	0	0	0	0	3	0	0	0	4	0	0	0	0	0	0	0	0	0	0	0	0	0	0	Fungi; Ascomycota; unidentified; unidentified; unidentified; unidentified; Ascomycota_sp
New.ReferenceOTU1772	1	0	0	0	0	0	0	0	0	0	0	0	0	0	0	0	0	0	0	0	0	0	0	0	0	0	0	0	0	0	Fungi; Ascomycota; unidentified; unidentified; unidentified; unidentified; Ascomycota_sp
New.ReferenceOTU2049	0	0	0	0	0	0	0	0	0	0	0	0	0	3	0	0	0	0	0	0	0	0	0	0	0	0	0	0	0	0	Fungi; Ascomycota; unidentified; unidentified; unidentified; unidentified; Ascomycota_sp
New.ReferenceOTU2654	0	0	0	0	0	0	0	0	0	0	0	2	0	0	0	0	0	0	0	0	0	0	0	0	0	0	0	0	0	0	Fungi; Ascomycota; unidentified; unidentified; unidentified; unidentified; Ascomycota_sp
New.ReferenceOTU2787	0	0	0	0	0	0	0	0	0	0	0	0	0	0	0	0	0	0	0	0	0	0	0	0	0	0	0	0	1	0	Fungi; Ascomycota; unidentified; unidentified; unidentified; unidentified; Ascomycota_sp
New.ReferenceOTU3119	0	0	0	0	0	0	0	0	0	0	0	0	0	0	0	0	0	0	1	0	0	0	0	0	0	0	0	0	0	0	Fungi; Ascomycota; unidentified; unidentified; unidentified; unidentified; Ascomycota_sp
New.ReferenceOTU3349	0	0	0	0	0	0	0	0	0	0	0	0	0	0	0	0	0	0	1	0	0	0	0	0	0	0	0	0	0	0	Fungi; Ascomycota; unidentified; unidentified; unidentified; unidentified; Ascomycota_sp
New.ReferenceOTU3425	0	0	0	0	0	0	0	0	0	0	0	0	0	0	0	0	0	0	2	0	0	0	0	0	0	0	0	0	0	0	Fungi; Ascomycota; unidentified; unidentified; unidentified; unidentified; Ascomycota_sp
New.ReferenceOTU3482	0	0	0	0	0	0	0	0	0	0	0	0	0	0	0	0	2	0	0	2	0	0	0	0	0	0	0	1	0	1	Fungi; Ascomycota; unidentified; unidentified; unidentified; unidentified; Ascomycota_sp
New.ReferenceOTU3495	0	0	0	0	0	0	2	0	0	0	0	9	6	2	0	4	0	0	0	0	0	3	2	0	4	0	3	0	0	32	Fungi; Ascomycota; unidentified; unidentified; unidentified; unidentified; Ascomycota_sp
New.ReferenceOTU3882	0	0	0	0	1	0	0	0	0	0	0	0	0	0	1	0	0	0	0	0	0	0	0	0	0	0	0	0	0	0	Fungi; Ascomycota; unidentified; unidentified; unidentified; unidentified; Ascomycota_sp
New.ReferenceOTU4174	0	0	0	0	0	0	0	0	0	0	0	0	0	0	0	0	0	3	0	0	0	0	0	0	0	11	0	0	0	0	Fungi; Ascomycota; unidentified; unidentified; unidentified; unidentified; Ascomycota_sp
New.ReferenceOTU4179	0	0	0	0	0	0	0	0	0	0	0	0	0	0	0	0	0	0	0	0	0	0	1	0	0	0	1	0	0	0	Fungi; Ascomycota; unidentified; unidentified; unidentified; unidentified; Ascomycota_sp
New.ReferenceOTU4186	0	0	0	0	0	0	0	0	0	1	1	0	0	1	0	0	0	0	0	0	0	0	0	1	0	0	0	0	0	0	Fungi; Ascomycota; unidentified; unidentified; unidentified; unidentified; Ascomycota_sp
New.ReferenceOTU4772	0	0	0	0	0	0	0	0	0	0	0	0	0	0	0	0	0	0	1	0	0	0	0	0	0	0	0	0	0	0	Fungi; Ascomycota; unidentified; unidentified; unidentified; unidentified; Ascomycota_sp
New.ReferenceOTU5175	0	0	1	0	1	0	0	0	0	0	0	0	0	0	0	6	1	1	0	0	0	1	0	0	3	0	0	0	0	0	Fungi; Ascomycota; unidentified; unidentified; unidentified; unidentified; Ascomycota_sp
New.ReferenceOTU5204	0	0	0	0	0	0	0	0	0	0	0	0	0	0	0	0	0	0	0	0	1	0	0	0	0	0	0	0	0	0	Fungi; Ascomycota; unidentified; unidentified; unidentified; unidentified; Ascomycota_sp
New.ReferenceOTU5269	0	0	0	0	1	0	0	0	0	0	0	0	0	0	0	0	0	0	0	0	0	0	0	0	0	0	0	0	0	0	Fungi; Ascomycota; unidentified; unidentified; unidentified; unidentified; Ascomycota_sp
New.ReferenceOTU5452	0	0	0	0	0	0	0	0	0	0	0	0	0	0	0	0	0	0	1	0	0	0	0	0	0	0	0	0	0	0	Fungi; Ascomycota; unidentified; unidentified; unidentified; unidentified; Ascomycota_sp
New.ReferenceOTU5687	0	0	0	0	0	0	0	0	1	0	0	0	0	0	0	0	0	0	0	0	0	0	0	0	0	0	0	0	0	0	Fungi; Ascomycota; unidentified; unidentified; unidentified; unidentified; Ascomycota_sp
New.ReferenceOTU6311	0	0	0	0	0	0	0	0	0	0	2	0	0	0	0	0	0	0	0	0	0	0	0	0	0	0	0	0	0	0	Fungi; Ascomycota; unidentified; unidentified; unidentified; unidentified; Ascomycota_sp
New.ReferenceOTU6367	1	0	0	0	0	0	0	0	0	0	1	0	0	0	0	0	0	0	0	0	0	0	0	0	0	0	0	0	0	0	Fungi; Ascomycota; unidentified; unidentified; unidentified; unidentified; Ascomycota_sp
New.ReferenceOTU6379	0	0	0	0	0	1	0	0	0	0	0	0	0	0	0	0	0	0	0	0	0	0	0	0	0	0	0	0	0	0	Fungi; Ascomycota; unidentified; unidentified; unidentified; unidentified; Ascomycota_sp
New.ReferenceOTU6507	0	0	0	0	0	0	0	0	0	0	2	1	0	0	0	0	0	0	0	0	0	0	0	0	0	0	0	0	0	0	Fungi; Ascomycota; unidentified; unidentified; unidentified; unidentified; Ascomycota_sp
New.ReferenceOTU6715	0	0	0	0	0	0	0	0	0	0	0	0	0	0	0	0	0	0	0	0	0	0	0	0	1	0	0	0	0	0	Fungi; Ascomycota; unidentified; unidentified; unidentified; unidentified; Ascomycota_sp
New.ReferenceOTU702	0	0	0	0	1	1	1	0	0	0	0	0	0	0	0	0	0	0	0	0	0	0	0	0	1	0	0	0	0	0	Fungi; Ascomycota; unidentified; unidentified; unidentified; unidentified; Ascomycota_sp
New.ReferenceOTU7079	0	0	0	0	0	0	0	0	0	0	0	1	0	0	0	0	0	0	0	0	0	0	0	0	0	0	0	0	0	0	Fungi; Ascomycota; unidentified; unidentified; unidentified; unidentified; Ascomycota_sp

New.ReferenceOTU711	0	0	0	0	0	0	0	0	0	0	0	0	0	0	0	0	0	0	0	0	0	0	0	0	0	0	0	0	1	0	Fungi; Ascomycota; unidentified; unidentified; unidentified; unidentified; Ascomycota_sp
New.ReferenceOTU7477	0	0	0	0	0	0	0	0	0	0	0	0	0	0	0	0	0	0	2	0	0	0	0	0	0	0	0	0	0	0	Fungi; Ascomycota; unidentified; unidentified; unidentified; unidentified; Ascomycota_sp
New.ReferenceOTU7696	0	0	0	0	0	0	0	0	1	0	0	0	0	0	0	0	0	0	0	0	0	0	0	0	0	0	0	0	0	0	Fungi; Ascomycota; unidentified; unidentified; unidentified; unidentified; Ascomycota_sp
New.ReferenceOTU7727	0	0	0	0	0	0	0	0	0	0	0	0	0	0	0	4	0	0	0	0	0	0	0	0	0	0	0	0	0	0	Fungi; Ascomycota; unidentified; unidentified; unidentified; unidentified; Ascomycota_sp
New.ReferenceOTU7896	0	0	1	0	0	0	0	0	0	0	0	0	0	0	0	0	1	1	1	0	0	0	0	0	0	0	0	0	0	0	Fungi; Ascomycota; unidentified; unidentified; unidentified; unidentified; Ascomycota_sp
New.ReferenceOTU8032	0	0	0	0	0	0	0	0	0	0	0	0	0	1	0	0	0	0	0	0	0	0	0	0	0	0	0	0	0	0	Fungi; Ascomycota; unidentified; unidentified; unidentified; unidentified; Ascomycota_sp
New.ReferenceOTU81	0	0	0	0	0	0	0	1	0	0	0	0	0	0	0	0	0	0	0	0	0	0	0	0	0	0	0	0	0	0	Fungi; Ascomycota; unidentified; unidentified; unidentified; unidentified; Ascomycota_sp
New.ReferenceOTU836	0	0	1	0	0	0	0	0	0	0	0	0	0	0	0	1	0	0	0	0	0	0	0	0	0	0	0	0	0	0	Fungi; Ascomycota; unidentified; unidentified; unidentified; unidentified; Ascomycota_sp
SH002719.06FU_FJ612605_reps_singleton	0	0	0	0	0	0	0	0	1	0	0	0	0	0	0	0	0	0	0	0	0	0	0	0	0	0	0	0	0	0	Fungi; Ascomycota; unidentified; unidentified; unidentified; unidentified; Ascomycota_sp
SH002722.06FU_FJ612629_reps_singleton	0	0	0	0	0	0	0	0	2	0	0	0	0	0	0	0	0	0	0	0	0	0	0	0	0	0	0	0	0	0	Fungi; Ascomycota; unidentified; unidentified; unidentified; unidentified; Ascomycota_sp
SH011458.06FU_KC218451_reps_singleton	0	0	0	0	0	1	0	0	0	0	0	0	0	0	0	0	0	0	0	0	0	0	1	0	0	0	0	0	0	0	Fungi; Ascomycota; unidentified; unidentified; unidentified; unidentified; Ascomycota_sp
SH024825.06FU_GQ229074_reps_singleton	0	0	0	0	0	1	0	1	0	0	0	1	0	0	0	0	1	0	0	0	0	0	2	0	0	0	0	0	0	0	Fungi; Ascomycota; unidentified; unidentified; unidentified; unidentified; Ascomycota_sp
SH024837.06FU_FJ612822_reps_singleton	0	0	0	0	0	0	0	0	1	1	0	0	1	0	2	1	0	0	0	1	0	1	0	0	1	0	0	0	0	1	Fungi; Ascomycota; unidentified; unidentified; unidentified; unidentified; Ascomycota_sp
SH024957.06FU_DQ631939_reps_singleton	1	0	0	0	0	0	0	0	0	0	1	0	0	0	0	0	0	0	0	0	0	0	0	0	0	0	0	0	0	0	Fungi; Ascomycota; unidentified; unidentified; unidentified; unidentified; Ascomycota_sp
SH024959.06FU_EU443836_reps_singleton	0	0	0	0	0	0	0	0	0	0	1	0	0	0	0	0	0	0	0	0	0	0	0	0	0	0	0	0	0	0	Fungi; Ascomycota; unidentified; unidentified; unidentified; unidentified; Ascomycota_sp
SH032330.06FU_EU329667_reps_singleton	0	0	0	0	0	0	0	0	0	0	0	0	0	0	0	0	0	0	0	1	0	0	0	0	0	0	0	0	0	0	Fungi; Ascomycota; unidentified; unidentified; unidentified; unidentified; Ascomycota_sp
SH085274.06FU_EU041775_reps	0	0	0	0	0	0	0	0	0	0	0	0	0	0	2	0	0	0	0	0	0	0	0	0	0	0	4	0	0	0	Fungi; Ascomycota; unidentified; unidentified; unidentified; unidentified; Ascomycota_sp
SH091831.06FU_HE608647_reps	0	0	0	0	0	0	0	0	0	0	0	0	0	0	0	0	0	0	1	0	0	0	0	0	2	0	0	0	0	0	Fungi; Ascomycota; unidentified; unidentified; unidentified; unidentified; Ascomycota_sp
SH091922.06FU_EU139858_reps	0	0	13	0	0	0	1	0	0	0	0	0	8	10	0	12	17	0	0	0	1	2	1	0	0	0	0	0	0	3	Fungi; Ascomycota; unidentified; unidentified; unidentified; unidentified; Ascomycota_sp
SH091923.06FU_AY273329_reps	0	0	0	0	0	0	0	0	0	0	0	0	0	0	0	0	1	0	0	0	0	0	0	0	0	0	0	0	0	0	Fungi; Ascomycota; unidentified; unidentified; unidentified; unidentified; Ascomycota_sp
SH094606.06FU_HQ328037_reps	0	0	0	0	1	0	1	0	0	0	0	1	0	0	0	0	0	0	0	0	0	0	0	0	0	0	0	0	0	0	Fungi; Ascomycota; unidentified; unidentified; unidentified; unidentified; Ascomycota_sp
SH096976.06FU_JQ905676_reps	0	0	0	0	0	0	0	0	0	0	0	0	0	0	0	0	0	0	0	1	0	0	0	0	0	0	0	0	0	0	Fungi; Ascomycota; unidentified; unidentified; unidentified; unidentified; Ascomycota_sp
SH104581.06FU_KF019272_reps	0	0	0	0	0	0	0	0	0	0	1	0	0	0	0	1	1	0	0	0	0	0	1	0	0	0	0	2	0	0	Fungi; Ascomycota; unidentified; unidentified; unidentified; unidentified; Ascomycota_sp
SH112370.06FU_HF548715_reps	0	0	0	0	0	0	1	0	0	0	0	0	0	0	0	0	0	0	0	0	0	0	0	0	0	0	0	0	0	0	Fungi; Ascomycota; unidentified; unidentified; unidentified; unidentified; Ascomycota_sp
SH118128.06FU_AM999670_reps	0	0	0	0	0	0	0	0	0	0	0	0	0	0	0	0	1	0	0	0	0	0	0	0	0	0	0	0	0	0	Fungi; Ascomycota; unidentified; unidentified; unidentified; unidentified; Ascomycota_sp
SH121124.06FU_FJ612932_reps	0	0	0	0	0	0	0	0	0	1	0	0	1	2	0	2	3	1	1	1	0	0	0	4	0	0	0	0	0	0	Fungi; Ascomycota; unidentified; unidentified; unidentified; unidentified; Ascomycota_sp
SH125935.06FU_AM921722_reps	0	0	0	0	0	0	0	0	0	0	0	0	0	0	0	2	0	0	0	0	0	0	0	0	0	0	0	0	0	0	Fungi; Ascomycota; unidentified; unidentified; unidentified; unidentified; Ascomycota_sp
SH125938.06FU_HE599284_refs	0	0	1	0	0	0	0	0	0	1	0	0	0	1	0	2	0	2	2	0	0	0	0	1	0	0	0	0	0	1	Fungi; Ascomycota; unidentified; unidentified; unidentified; unidentified; Ascomycota_sp
SH125939.06FU_FN393419_reps	0	0	0	0	0	0	0	0	0	0	0	0	0	0	0	1	0	0	0	0	0	0	0	0	0	0	0	0	0	0	Fungi; Ascomycota; unidentified; unidentified; unidentified; unidentified; Ascomycota_sp
SH126032.06FU_FJ037741_reps	0	0	0	0	0	0	0	0	0	1	0	0	0	0	1	0	0	0	0	0	0	0	0	0	0	0	0	0	0	0	Fungi; Ascomycota; unidentified; unidentified; unidentified; unidentified; Ascomycota_sp
SH126455.06FU_FR734101_refs	3	0	1	0	4	4	7	3	1	2	2	5	0	0	2	0	1	0	0	1	4	0	1	5	3	0	1	1	0	4	Fungi; Ascomycota; unidentified; unidentified; unidentified; unidentified; Ascomycota_sp
SH126462.06FU_AY624188_reps	2	0	1	1	0	0	3	0	0	0	1	1	1	0	1	1	0	0	0	1	0	0	1	2	2	0	0	0	0	2	Fungi; Ascomycota; unidentified; unidentified; unidentified; unidentified; Ascomycota_sp
SH126628.06FU_DQ092503_reps	0	0	0	0	0	0	0	0	0	0	0	5	0	0	0	0	0	0	0	0	0	0	0	0	0	0	0	0	0	0	Fungi; Ascomycota; unidentified; unidentified; unidentified; unidentified; Ascomycota_sp
SH126718.06FU_DQ223749_reps	2	0	2	1	0	0	0	0	0	1	2	0	0	2	1	0	0	0	0	0	0	1	0	0	0	0	0	0	0	0	Fungi; Ascomycota; unidentified; unidentified; unidentified; unidentified; Ascomycota_sp
SH129529.06FU_KC507199_reps	0	0	0	0	1	0	0	0	0	1	0	0	0	0	1	0	1	0	0	0	0	0	0	0	0	0	0	0	0	0	Fungi; Ascomycota; unidentified; unidentified; unidentified; unidentified; Ascomycota_sp
SH130386.06FU_HQ293217_reps	0	0	0	0	3	0	0	0	0	0	0	0	0	0	1	0	0	0	0	0	1	0	1	0	9	0	0	0	0	1	Fungi; Ascomycota; unidentified; unidentified; unidentified; unidentified; Ascomycota_sp
SH131571.06FU_AY624196_refs	0	0	0	0	0	0	0	0	1	0	0	0	0	0	0	0	0	0	0	0	0	0	0	0	0	0	0	0	0	0	Fungi; Ascomycota; unidentified; unidentified; unidentified; unidentified; Ascomycota_sp
SH131572.06FU_JQ266120_reps	0	0	0	0	0	0	0	0	0	0	0	0	0	0	0	0	0	4	0	0	0	0	0	0	0	0	0	0	0	0	Fungi; Ascomycota; unidentified; unidentified; unidentified; unidentified; Ascomycota_sp
SH132853.06FU_FJ708614_reps	0	0	0	0	0	0	0	0	0	0	0	0	0	0	0	0	0	0	0	0	0	1	0	0	0	0	0	0	0	0	Fungi; Ascomycota; unidentified; unidentified; unidentified; unidentified; Ascomycota_sp
SH137944.06FU_EF488412_reps	0	0	0	0	0	0	0	0	0	0	0	0	0	0	0	0	0	0	0	3	0	0	0	0	0	0	0	0	0	0	Fungi; Ascomycota; unidentified; unidentified; unidentified; unidentified; Ascomycota_sp
SH139393.06FU_HM776417_reps	0	0	0	0	2	0	0	0	0	0	0	1	0	0	0	0	0	0	0	0	1	2	1	0	2	0	1	0	0	0	Fungi; Ascomycota; unidentified; unidentified; unidentified; unidentified; Ascomycota_sp
SH139395.06FU_HQ829122_reps	0	0	0	0	0	0	0	0	0	0	0	1	0	0	0	0	0	0	0	0	0	3	6	0	21	0	2	11	0	1	Fungi; Ascomycota; unidentified; unidentified; unidentified; unidentified; Ascomycota_sp
SH139446.06FU_KC425454_reps	0	0	0	0	0	0	0	0	0	0	0	0	0	0	1	0	0	0	0	0	0	0	1	0	0	0	0	0	0	0	Fungi; Ascomycota; unidentified; unidentified; unidentified; unidentified; Ascomycota_sp
SH140339.06FU_DQ489301_reps	0	0	0	3	0	0	0	0	0	0	0	1	0	3	0	0	0	0	0	0	0	0	0	1	0	0	0	0	0	0	Fungi; Ascomycota; unidentified; unidentified; unidentified; unidentified; Ascomycota_sp
SH140340.06FU_EF641869_reps	0	0	0	0	0	0	0	0	0	0	0	0	0	0	0	1	0	0	0	0	0	0	0	0	0	0	0	0	0	0	Fungi; Ascomycota; unidentified; unidentified; unidentified; unidentified; Ascomycota_sp
SH140505.06FU_GU566286_reps	0	0	0	0	0	0	0	0	0	0	0	0	0	0	0	0	0	0	0	0	5	0	0	0	0	0	0	0	0	0	Fungi; Ascomycota; unidentified; unidentified; unidentified; unidentified; Ascomycota_sp
SH140656.06FU_KC311499_reps	0	0	0	0	0	0	0	0	0	0	0	0	0	0	0	0	0	1	0	0	0	0	0	0	0	0	0	0	0	0	Fungi; Ascomycota; unidentified; unidentified; unidentified; unidentified; Ascomycota_sp
SH141468.06FU_EU563530_reps	0	0	0	0	0	0	0	0	0	0	0	0	0	0	0	0	1	0	0	0	0	0	0	0	0	0	0	0	0	0	Fungi; Ascomycota; unidentified; unidentified; unidentified; unidentified; Ascomycota_sp
SH141483.06FU_FJ612947_reps	0	0	0	0	1	0	0	0	0	0	0	0	0	0	1	0	0	0	0	0	0	0	0	0	0	0	0	0	0	0	Fungi; Ascomycota; unidentified; unidentified; unidentified; unidentified; Ascomycota_sp
SH146684.06FU_HE962592_reps	0	0	0	0	0	0	0	0	2	0	0	0	0	0	0	0	0	0	0	0	0	1	0	0	0	0	0	0	0	0	Fungi; Ascomycota; unidentified; unidentified; unidentified; unidentified; Ascomycota_sp
New.ReferenceOTU1895	0	0	0	0	0	0	0	0	0	0	0	0	0	0	0	0	0	0	2	0	0	0	0	0	0	0	0	0	0	0	Fungi; Ascomycota; unidentified; unidentified; unidentified; unidentified; Ascomycota_sp_119
SH146024.06FU_AB769884_reps	0	0	0	0	0	0	0	0	0	0	0	0	0	0	1	0	0	0	0	0	0	0	0	0	2	0	0	0	0	0	Fungi; Ascomycota; unidentified; unidentified; unidentified; unidentified; Ascomycota_sp_119
SH030421.06FU_KF019280_reps_singleton	0	0	0	0	0	0	0	0	0	0	0	0	0	2	0	0	0	0	0	0	0	0	0	0	0	0	0	0	0	0	Fungi; Ascomycota; unidentified; unidentified; unidentified; unidentified; Ascomycota_sp_12239
New.ReferenceOTU6849	0	2	0	1	0	0	0	0	0	0	1	0	0	0	0	0	0	0	0	0	0	0	0	0	0	0	0	0	0	0	Fungi; Ascomycota; unidentified; unidentified; unidentified; unidentified; Ascomycota_sp_AR_2010
New.ReferenceOTU6853	0	0	0	0	0	0	0	0	0	0	0	0	0	0	0	0	0	0	0	0	0	0	0	0	0	0	0	0	1	0	Fungi; Ascomycota; unidentified; unidentified; unidentified; unidentified; Ascomycota_sp_AR_2010
SH028886.06FU_JQ649378_reps_singleton	0	0	0	0	0	0	0	0	0	0	0	0	0	0	0	1	0	0	0	0	0	0	0	0	0	0	0	0	0	0	Fungi; Ascomycota; unidentified; unidentified; unidentified; unidentified; Ascomycota_sp_CCG_2012b
New.ReferenceOTU4318	0	0	0	0	0	0	0	0	0	0	0	0	0	0	0	0	0	0	0	0	0	0	0	0	0	0	0	0	0	2	Fungi; Ascomycota; unidentified; unidentified; unidentified; unidentified; Ascomycota_sp_E10016a
New.ReferenceOTU1589	0	0	0	0	1	0	0	0	0	0	0	0	0	0	0	0	0	0	0	0	0	0	0	0	0	0	0	0	0	0	Fungi; Ascomycota; unidentified; unidentified; unidentified; unidentified; Ascomycota_sp_H33
New.ReferenceOTU7484	0	0	0	0	0	0	0	0	0	0	0	0	0	0	0	0	0	2	0	0	0	0	0	0	0	0	0	0	0	0	Fungi; Ascomycota; unidentified; unidentified; unidentified; unidentified; Ascomycota_sp_MS533
New.ReferenceOTU7738	0	0	0	0	1	0	0	0	0	0	0	0	0	0	0	0	0	0	0	0	0	0	0	0	0	0	0	0	0	0	Fungi; Ascomycota; unidentified; unidentified; unidentified; unidentified; Ascomycota_sp_OY17007
New.ReferenceOTU873	0	0	1	0	0	0	0	0	0	0	0	0	1	0	0	0	0	0	0	0	0	0	0	0	0	0	0	0	0	0	Fungi; Ascomycota; unidentified; unidentified; unidentified; unidentified; Ascomycota_sp_OY17007
SH454695.06FU_FJ571426_reps_singleton	0	0	0	0	0	0	0	0	0	0	0	0	0	0	0	0	0	0	0	0	0	0	1	0	0	0	0	0	0	0	Fungi; Ascomycota; unidentified; unidentified; unidentified; unidentified; Ascomycota_sp_OY17007
New.ReferenceOTU1681	0	0	0	0	0	0	0	0	0	0	0	0	0	0	0	0	0	0	0	0	0	0	0	0	0	0	0	1	0	0	Fungi; Ascomycota; unidentified; unidentified; unidentified; unidentified; foliar_endophyte_of_Picea_glauca_sp_L6
New.ReferenceOTU7059	0	0	0	0	0	0	0	0	0	0	0	0	0	0	0	0	0	0	2	0	0	0	0	0	0	0	0	0	0	0	Fungi; Ascomycota; unidentified; unidentified; unidentified; unidentified; leaf_litter_ascomycete_strain_its016
SH034599.06FU_AF502663_reps_singleton	0	1	0	0	0	0	0	1	0	1	0	0	0	2	0	0	1	0	1	0	0	0	0	0	0	0	0	0	0	0	Fungi; Ascomycota; unidentified; unidentified; unidentified; unidentified; leaf_litter_ascomycete_strain_its082
New.ReferenceOTU3861	0	0	0	0	0	0	0	0	16	0	0	0	0	0	0	0	0	0	0	0	0	0	0	0	0	0	0	0	0	0	Fungi; Ascomycota; unidentified; unidentified; unidentified; unidentified; leaf_litter_ascomycete_strain_its167
SH023305.06FU_AF502705_reps_singleton	2	0	0	0	0	0	0	0	0	0	0	0	0	0	0	0	0	0	0	0	0	0	0	4	0	0	0	0	0	0	Fungi; Ascomycota; unidentified; unidentified; unidentified; unidentified; leaf_litter_ascomycete_strain_its167
New.ReferenceOTU1037	3	0	0	0	0	1	2	0	9	12	8	1	0	0	1	5	1	0	1	8	0	0	1	0	0	0	0	0	0	0	Fungi; Ascomycota; unidentified; unidentified; unidentified; unidentified; leaf_litter_ascomycete_strain_its180
New.ReferenceOTU1510	0	0	0	0	0	0	0	0	0	0	0	0	0	0	0	0	0	0	0	0	1	0	0	0	0	0	0	0	0	0	Fungi; Ascomycota; unidentified; unidentified; unidentified; unidentified; leaf_litter_ascomycete_strain_its180
New.ReferenceOTU4004	0	0	0	0	0	0	0	0	0	0	0	0	0	0	0	0	0	1	0	0	0	0	0	0	0	0	0	0	0	0	Fungi; Ascomycota; unidentified; unidentified; unidentified; unidentified; leaf_litter_ascomycete_strain_its341
New.ReferenceOTU320	0	0	0	0	0	0	0	0	0	0	0	0	0	0	0	1	0	0	3	0	0	0	0	0	0	0	1	0	0	0	Fungi; Ascomycota; unidentified; unidentified; unidentified; unidentified; leaf_litter_ascomycete_strain_its348
New.ReferenceOTU2126	2	0	2	0	0	12	4	8	0	9	0	2	31	2	0	10	5	0	0	1	0	1	0	0	0	0	2	0	0	0	Fungi; Ascomycota; unidentified; unidentified; unidentified; unidentified; leaf_litter_ascomycete_strain_its351
SH005137.06FU_HE579352_reps_singleton	0	0	3	0	0	2	4	5	0	2	3	0	17	3	1	9	6	0	0	0	0	0	0	3	1	0	2	0	0	0	Fungi; Ascomycota; unidentified; unidentified; unidentified; unidentified; leaf_litter_ascomycete_strain_its351
SH005140.06FU_GU083033_reps_singleton	0	0	0	0	0	1	0	0	0	0	2	0	0	1	0	0	0	0	0	0	0	0	0	1	0	0	0	0	0	0	Fungi; Ascomycota; unidentified; unidentified; unidentified; unidentified; leaf_litter_ascomycete_strain_its351
SH005143.06FU_GQ120971_reps_singleton	0	0	1	0	1	1	0	0	0	0	0	0	1	0	0	1	0	0	0	0	0	0	0	0	0	0	0	0	0	0	Fungi; Ascomycota; unidentified; unidentified; unidentified; unidentified; leaf_litter_ascomycete_strain_its351
New.CleanUp.ReferenceOTU1952	0	0	0	0	0	0	0	0	0	0	0	0	0	1	0	0	0	0	0	0	0	0	0	0	0	0	0	0	0	0	Fungi; Ascomycota; unidentified; unidentified; unidentified; unidentified; leaf_litter_ascomycete_strain_its352
New.ReferenceOTU3780	0	0	0	0	0	0	0	0	0	2	0	0	0	0	0	0	0	0	0	0	0	0	0	0	0	0	0	0	0	0	Fungi; Ascomycota; unidentified; unidentified; unidentified; unidentified; leaf_litter_ascomycete_strain_its367
New.ReferenceOTU786	0	0	0	0	0	0	0	0	0	0	0	0	0	0	0	0	0	0	1	0	0	0	0	0	0	0	0	0	0	0	Fungi; Ascomycota; unidentified; unidentified; unidentified; unidentified; leaf_litter_ascomycete_strain_its367
New.ReferenceOTU253	0	0	0	0	0	0	0	0	0	0	0	0	0	0	0	0	0	0	0	1	0	0	0	0	0	0	0	0	0	0	Fungi; Ascomycota; unidentified; unidentified; unidentified; unidentified; leaf_litter_ascomycete_strain_its389
New.ReferenceOTU29	0	0	5	0	0	0	0	0	0	0	0	0	0	0	0	0	0	0	0	0	0	0	0	0	0	0	0	0	0	0	Fungi; Ascomycota; unidentified; unidentified; unidentified; unidentified; leaf_litter_ascomycete_strain_its389
New.ReferenceOTU98	3	0	0	5	0	0	0	0	0	0	0	0	0	0	0	0	0	0	0	0	0	0	0	0	0	0	0	0	0	0	Fungi; Ascomycota; unidentified; unidentified; unidentified; unidentified; leaf_litter_ascomycete_strain_its389
New.ReferenceOTU7154	0	0	0	0	0	0	0	0	0	0	0	0	0	0	0	0	1	0	0	0	0	0	0	0	0	0	0	0	0	0	Fungi; Ascomycota; unidentified; unidentified; unidentified; unidentified; melanized_limestone_ascomycete_CR_2004
New.ReferenceOTU119	0	0	0	3	0	0	0	0	0	0	0	0	0	0	0	0	0	0	0	0	0	0	0	0	0	1	0	0	0	0	Fungi; Ascomycota; unidentified; unidentified; unidentified; unidentified; uncultured_Ascomycota
New.ReferenceOTU1572	3	0	0	0	0	0	0	0	0	0	0	0	0	0	0	0	0	0	0	0	0	0	0	0	0	0	0	0	0	0	Fungi; Ascomycota; unidentified; unidentified; unidentified; unidentified; uncultured_Ascomycota
New.ReferenceOTU2	0	0	21	0	0	0	0	0	3	0	0	0	0	0	0	0	0	0	0	0	0	0	0	0	0	0	0	0	0	0	Fungi; Ascomycota; unidentified; unidentified; unidentified; unidentified; uncultured_Ascomycota
New.ReferenceOTU2279	0	0	0	0	0	0	0	0	0	0	0	0	3	0	0	2	0	0	0	0	0	0	0	0	0	0	0	0	0	0	Fungi; Ascomycota; unidentified; unidentified; unidentified; unidentified; uncultured_Ascomycota
New.ReferenceOTU2673	3	0	0	0	0	0	0	0	0	0	0	0	0	0	0	0	0	0	0	0	0	0	0	0	0	0	0	0	0	0	Fungi; Ascomycota; unidentified; unidentified; unidentified; unidentified; uncultured_Ascomycota
New.ReferenceOTU2720	0	0	0	0	0	0	0	0	0	3	0	0	0	4	2	1	1	0	0	0	0	0	0	2	0	1	0	0	6	0	Fungi; Ascomycota; unidentified; unidentified; unidentified; unidentified; uncultured_Ascomycota
New.ReferenceOTU2780	0	0	0	0	0	0	0	0	0	0	0	0	0	0	0	0	0	0	0	0	0	0	0	0	1	0	0	0	0	0	Fungi; Ascomycota; unidentified; unidentified; unidentified; unidentified; uncultured_Ascomycota
New.ReferenceOTU2892	0	0	0	0	0	0	0	0	0	0	6	0	0	0	0	0	0	0	0	0	0	0	0	0	0	0	0	0	0	0	Fungi; Ascomycota; unidentified; unidentified; unidentified; unidentified; uncultured_Ascomycota
New.ReferenceOTU2893	0	0	0	0	1	0	0	0	0	0	0	1	0	0	0	0	0	0	0	0	0	0	0	0	0	0	0	0	0	0	Fungi; Ascomycota; unidentified; unidentified; unidentified; unidentified; uncultured_Ascomycota
New.ReferenceOTU3229	0	1	0	0	0	0	0	0	0	0	0	0	0	0	0	0	0	0	0	0	0	0	0	0	0	0	0	0	0	0	Fungi; Ascomycota; unidentified; unidentified; unidentified; unidentified; uncultured_Ascomycota
New.ReferenceOTU3281	15	0	0	1	0	0	0	0	0	0	0	0	0	0	0	0	0	0	0	0	0	0	0	0	0	0	0	0	0	0	Fungi; Ascomycota; unidentified; unidentified; unidentified; unidentified; uncultured_Ascomycota
New.ReferenceOTU3404	0	0	0	0	0	0	0	0	0	0	1	0	0	0	0	0	0	0	0	0	0	0	0	0	0	0	0	0	0	0	Fungi; Ascomycota; unidentified; unidentified; unidentified; unidentified; uncultured_Ascomycota
New.ReferenceOTU3477	0	0	0	0	0	0	0	0	0	0	0	0	0	1	0	0	0	0	0	0	0	0	0	0	0	0	0	0	0	0	Fungi; Ascomycota; unidentified; unidentified; unidentified; unidentified; uncultured_Ascomycota
New.ReferenceOTU3781	0	0	0	0	0	0	0	0	0	0	0	0	0	0	0	4	0	0	0	0	0	0	0	0	0	0	0	0	0	0	Fungi; Ascomycota; unidentified; unidentified; unidentified; unidentified; uncultured_Ascomycota
New.ReferenceOTU3872	0	0	0	0	10	0	0	0	0	0	0	0	0	0	0	0	0	0	0	0	0	0	0	0	0	0	0	0	0	0	Fungi; Ascomycota; unidentified; unidentified; unidentified; unidentified; uncultured_Ascomycota
New.ReferenceOTU4129	0	0	0	0	0	0	0	1	0	0	0	0	0	0	0	0	0	0	0	0	0	0	0	0	0	0	0	0	0	0	Fungi; Ascomycota; unidentified; unidentified; unidentified; unidentified; uncultured_Ascomycota
New.ReferenceOTU417	0	0	0	0	0	0	0	0	0	0	0	0	0	0	0	0	0	0	1	0	0	0	0	0	0	0	0	0	0	0	Fungi; Ascomycota; unidentified; unidentified; unidentified; unidentified; uncultured_Ascomycota
New.ReferenceOTU4696	0	0	0	0	0	0	0	0	0	0	0	0	0	1	0	0	0	0	0	0	0	0	0	0	0	0	0	1	0	0	Fungi; Ascomycota; unidentified; unidentified; unidentified; unidentified; uncultured_Ascomycota

New.ReferenceOTU4990	1	0	0	0	0	0	0	0	0	0	0	0	0	0	0	0	0	0	0	0	0	0	0	0	0	0	0	0	0	0	Fungi; Ascomycota; unidentified; unidentified; unidentified; unidentified; uncultured_Ascomycota
New.ReferenceOTU4999	0	0	0	0	1	0	0	0	0	0	0	0	0	0	0	0	0	0	0	0	0	0	0	0	0	0	0	0	0	0	Fungi; Ascomycota; unidentified; unidentified; unidentified; unidentified; uncultured_Ascomycota
New.ReferenceOTU5310	0	0	0	2	0	0	0	0	0	0	0	0	0	0	0	0	0	0	0	0	0	0	0	0	0	0	0	0	0	0	Fungi; Ascomycota; unidentified; unidentified; unidentified; unidentified; uncultured_Ascomycota
New.ReferenceOTU539	0	0	0	0	2	0	0	0	0	0	0	0	0	0	0	0	0	0	0	0	0	0	0	0	0	0	0	0	0	0	Fungi; Ascomycota; unidentified; unidentified; unidentified; unidentified; uncultured_Ascomycota
New.ReferenceOTU5641	0	0	0	0	0	0	0	0	0	0	3	0	0	0	0	0	0	0	0	0	0	0	0	0	0	0	0	0	0	0	Fungi; Ascomycota; unidentified; unidentified; unidentified; unidentified; uncultured_Ascomycota
New.ReferenceOTU6322	0	0	0	2	0	0	0	0	0	0	0	0	0	0	0	0	0	0	0	0	0	0	0	0	0	0	0	0	0	0	Fungi; Ascomycota; unidentified; unidentified; unidentified; unidentified; uncultured_Ascomycota
New.ReferenceOTU633	0	0	0	0	0	0	0	0	0	0	0	0	1	0	0	0	0	0	0	0	0	0	0	1	0	0	0	0	0	0	Fungi; Ascomycota; unidentified; unidentified; unidentified; unidentified; uncultured_Ascomycota
New.ReferenceOTU6409	0	0	0	1	0	0	0	0	0	0	0	0	0	0	0	0	0	0	0	0	0	0	0	0	0	0	0	0	0	0	Fungi; Ascomycota; unidentified; unidentified; unidentified; unidentified; uncultured_Ascomycota
New.ReferenceOTU6566	0	0	1	0	0	0	0	0	0	0	0	0	0	0	0	0	0	0	0	0	0	0	0	0	0	0	0	0	0	0	Fungi; Ascomycota; unidentified; unidentified; unidentified; unidentified; uncultured_Ascomycota
New.ReferenceOTU6678	0	0	0	0	0	0	0	0	0	0	0	0	0	0	0	1	0	0	0	0	0	0	0	0	0	0	0	0	0	0	Fungi; Ascomycota; unidentified; unidentified; unidentified; unidentified; uncultured_Ascomycota
New.ReferenceOTU6758	3	4	0	0	0	0	0	0	0	1	1	0	0	0	0	0	0	0	0	0	0	0	0	0	0	0	0	0	0	0	Fungi; Ascomycota; unidentified; unidentified; unidentified; unidentified; uncultured_Ascomycota
New.ReferenceOTU6807	0	0	0	0	0	0	0	0	0	0	0	0	0	0	0	0	0	0	5	0	0	0	0	0	0	0	0	0	0	0	Fungi; Ascomycota; unidentified; unidentified; unidentified; unidentified; uncultured_Ascomycota
New.ReferenceOTU6887	3	0	0	0	0	0	0	0	0	0	0	0	0	0	0	0	0	0	0	0	0	0	0	0	0	0	0	0	0	0	Fungi; Ascomycota; unidentified; unidentified; unidentified; unidentified; uncultured_Ascomycota
New.ReferenceOTU7247	0	0	0	0	0	0	0	0	0	0	0	0	0	0	0	1	0	0	0	0	0	0	0	0	0	0	0	0	0	0	Fungi; Ascomycota; unidentified; unidentified; unidentified; unidentified; uncultured_Ascomycota
New.ReferenceOTU7786	0	0	0	0	0	0	0	0	0	0	0	0	0	0	0	5	0	0	0	0	0	0	0	0	0	0	0	0	0	0	Fungi; Ascomycota; unidentified; unidentified; unidentified; unidentified; uncultured_Ascomycota
New.ReferenceOTU5173	0	0	0	0	0	0	3	0	0	0	0	0	0	0	0	0	0	0	0	0	0	0	0	0	0	0	0	0	0	0	Fungi; Ascomycota; unidentified; unidentified; unidentified; unidentified; uncultured_Saccharomycotina
New.ReferenceOTU4169	0	0	0	0	3	0	1	1	0	0	0	0	0	1	0	0	0	0	0	0	0	0	0	3	0	0	0	1	0	0	Fungi; Basidiomycota; Agaricomycetes; Agaricales; Agaricaceae; unidentified; Agaricaceae_sp
New.ReferenceOTU6250	0	0	0	0	0	0	0	0	0	0	0	0	0	0	0	0	0	0	2	0	0	0	0	0	0	0	0	0	0	0	Fungi; Basidiomycota; Agaricomycetes; Agaricales; Agaricaceae; unidentified; Lepiotaceae_sp_PA480
New.ReferenceOTU371	0	1	0	0	0	0	0	0	0	0	0	0	0	0	0	0	0	0	0	0	0	0	0	0	0	0	0	0	0	0	Fungi; Basidiomycota; Agaricomycetes; Agaricales; Amanitaceae; unidentified; Amanitaceae_sp
New.ReferenceOTU1159	0	0	0	0	0	0	0	0	0	0	0	0	0	0	0	0	0	0	0	0	1	0	0	0	0	0	0	0	0	0	Fungi; Basidiomycota; Agaricomycetes; Agaricales; Clavariaceae; unidentified; Clavariaceae_sp
New.ReferenceOTU1389	0	0	0	0	0	0	0	0	0	0	0	0	0	0	0	0	0	0	1	0	0	0	0	0	0	0	0	0	0	0	Fungi; Basidiomycota; Agaricomycetes; Agaricales; Clavariaceae; unidentified; Clavariaceae_sp
New.ReferenceOTU1415	0	0	0	0	0	0	0	0	0	0	1	0	0	0	0	0	0	0	0	0	0	0	0	0	0	0	0	0	0	0	Fungi; Basidiomycota; Agaricomycetes; Agaricales; Clavariaceae; unidentified; Clavariaceae_sp
New.ReferenceOTU179	0	0	0	0	0	0	0	0	0	0	0	0	0	0	1	0	0	0	0	0	0	0	0	0	8	0	0	0	0	0	Fungi; Basidiomycota; Agaricomycetes; Agaricales; Clavariaceae; unidentified; Clavariaceae_sp
New.ReferenceOTU1815	28	0	0	0	0	0	0	0	0	0	0	0	0	0	0	0	0	0	0	0	0	0	0	0	0	0	0	0	0	0	Fungi; Basidiomycota; Agaricomycetes; Agaricales; Clavariaceae; unidentified; Clavariaceae_sp
New.ReferenceOTU3868	0	0	0	0	0	0	0	0	0	0	0	0	0	0	0	0	0	0	0	0	0	0	0	0	0	0	0	0	0	1	Fungi; Basidiomycota; Agaricomycetes; Agaricales; Clavariaceae; unidentified; Clavariaceae_sp
New.ReferenceOTU4654	0	0	0	0	0	0	0	0	4	0	0	0	0	0	0	0	0	0	0	0	0	0	0	0	0	0	0	0	0	0	Fungi; Basidiomycota; Agaricomycetes; Agaricales; Clavariaceae; unidentified; Clavariaceae_sp
New.ReferenceOTU5823	0	0	0	0	0	0	0	0	0	0	2	0	0	0	0	0	0	0	0	0	0	0	0	3	0	0	0	0	0	0	Fungi; Basidiomycota; Agaricomycetes; Agaricales; Clavariaceae; unidentified; Clavariaceae_sp
New.ReferenceOTU7862	3	0	0	0	0	0	0	0	0	0	0	0	0	0	0	0	0	0	0	0	0	0	0	0	0	0	0	0	0	0	Fungi; Basidiomycota; Agaricomycetes; Agaricales; Clavariaceae; unidentified; Clavariaceae_sp
New.ReferenceOTU2066	0	0	0	0	0	0	0	0	0	0	0	0	0	0	0	0	0	0	0	0	0	0	0	0	1	0	0	0	0	0	Fungi; Basidiomycota; Agaricomycetes; Agaricales; Clavariaceae; unidentified; uncultured_Clavariaceae
New.ReferenceOTU2038	0	0	0	0	0	0	0	0	0	0	0	0	0	0	0	0	0	0	0	0	0	0	0	0	0	0	1	0	0	0	Fungi; Basidiomycota; Agaricomycetes; Agaricales; Entolomataceae; unidentified; Entolomataceae_sp
New.ReferenceOTU4908	0	0	0	0	0	0	0	0	0	0	0	0	0	0	0	0	0	6	0	0	0	0	0	0	0	0	0	0	0	0	Fungi; Basidiomycota; Agaricomycetes; Agaricales; Entolomataceae; unidentified; Entolomataceae_sp
New.ReferenceOTU5621	0	0	1	0	0	0	0	0	0	0	0	0	0	0	0	0	0	0	0	0	0	0	0	0	0	0	0	0	0	0	Fungi; Basidiomycota; Agaricomycetes; Agaricales; Entolomataceae; unidentified; uncultured_Entoloma
New.ReferenceOTU187	0	0	0	0	0	0	1	0	0	0	0	0	0	1	0	0	0	0	0	0	0	0	0	0	0	0	0	0	0	0	Fungi; Basidiomycota; Agaricomycetes; Agaricales; Lyophyllaceae; unidentified; Lyophyllaceae_sp
New.ReferenceOTU6225	0	0	0	0	1	1	3	0	0	0	0	0	0	0	0	0	0	0	0	0	0	0	2	0	0	0	2	0	0	0	Fungi; Basidiomycota; Agaricomycetes; Agaricales; Lyophyllaceae; unidentified; Lyophyllaceae_sp
SH089084.06FU_DQ054571_reps	0	0	0	0	0	0	0	0	0	0	0	1	0	0	0	0	0	0	0	0	0	0	0	0	0	0	1	0	0	0	Fungi; Basidiomycota; Agaricomycetes; Agaricales; Lyophyllaceae; unidentified; Lyophyllaceae_sp
New.ReferenceOTU1724	1	0	0	1	0	0	0	0	0	0	0	0	0	0	0	0	0	0	0	0	0	5	0	0	0	0	0	0	0	0	Fungi; Basidiomycota; Agaricomycetes; Agaricales; Mycenaceae; unidentified; Mycenaceae_sp
New.ReferenceOTU4125	0	0	0	0	0	0	0	0	0	0	0	0	0	0	0	0	0	0	0	0	0	0	0	1	0	0	0	0	0	0	Fungi; Basidiomycota; Agaricomycetes; Agaricales; Tricholomataceae; Delicatula; Delicatula_integrella
New.ReferenceOTU2110	6	0	0	0	0	0	0	0	0	0	0	0	0	0	0	0	0	0	0	0	0	0	0	0	0	0	0	0	0	0	Fungi; Basidiomycota; Agaricomycetes; Agaricales; Tricholomataceae; unidentified; uncultured_Clitocybula
New.ReferenceOTU5006	9	0	0	0	0	0	0	0	0	0	0	0	0	0	0	0	0	0	0	0	0	0	0	0	0	0	0	0	0	0	Fungi; Basidiomycota; Agaricomycetes; Agaricales; Tricholomataceae; unidentified; uncultured_Clitocybula
New.ReferenceOTU4281	1	0	0	0	0	0	0	0	0	0	0	0	0	0	0	0	0	0	0	0	0	0	0	0	0	0	0	0	0	0	Fungi; Basidiomycota; Agaricomycetes; Agaricales; Tricholomataceae; unidentified; uncultured_Tricholoma
New.ReferenceOTU519	0	0	0	0	0	0	0	0	0	0	0	0	0	0	1	0	0	0	0	0	0	0	0	0	0	0	0	0	0	0	Fungi; Basidiomycota; Agaricomycetes; Agaricales; unidentified; unidentified; Agaricales_sp
New.ReferenceOTU7456	19	0	0	0	0	0	1	0	0	0	0	0	0	0	0	0	0	0	0	0	0	0	0	0	0	0	0	0	0	0	Fungi; Basidiomycota; Agaricomycetes; Agaricales; unidentified; unidentified; Agaricales_sp
New.ReferenceOTU7530	0	0	0	0	0	0	0	0	0	0	0	0	0	0	0	0	0	0	0	0	0	0	0	0	3	0	0	0	0	0	Fungi; Basidiomycota; Agaricomycetes; Agaricales; unidentified; unidentified; Agaricales_sp
New.ReferenceOTU96	2	0	0	0	0	0	0	0	0	0	0	0	0	0	0	0	0	0	0	0	0	0	0	0	0	0	0	0	0	0	Fungi; Basidiomycota; Agaricomycetes; Agaricales; unidentified; unidentified; Agaricales_sp
New.ReferenceOTU7111	16	0	0	0	0	0	0	0	0	0	0	0	0	0	0	0	0	0	0	0	0	0	0	0	0	0	0	0	0	0	Fungi; Basidiomycota; Agaricomycetes; Agaricales; unidentified; unidentified; Agaricales_sp_Cs2_1
New.ReferenceOTU1017	0	0	0	1	0	0	0	0	0	0	0	0	0	0	0	0	0	0	0	0	0	0	0	0	0	0	0	0	0	0	Fungi; Basidiomycota; Agaricomycetes; Auriculariales; unidentified; unidentified; Auriculariales_sp
New.ReferenceOTU1066	0	0	0	0	0	0	0	0	0	0	0	0	0	1	0	0	0	0	0	0	0	0	0	0	0	0	0	0	0	0	Fungi; Basidiomycota; Agaricomycetes; Auriculariales; unidentified; unidentified; Auriculariales_sp
New.ReferenceOTU1409	1	0	0	0	0	0	0	0	0	0	0	0	0	0	0	0	0	0	0	0	0	0	0	0	0	0	0	0	0	0	Fungi; Basidiomycota; Agaricomycetes; Auriculariales; unidentified; unidentified; Auriculariales_sp
New.ReferenceOTU1423	3	0	0	1	0	0	0	0	0	0	0	0	0	0	0	0	0	0	0	0	0	0	0	0	0	0	0	0	0	0	Fungi; Basidiomycota; Agaricomycetes; Auriculariales; unidentified; unidentified; Auriculariales_sp
New.ReferenceOTU2464	0	0	0	0	0	0	0	0	0	0	0	0	0	0	0	0	0	0	0	3	0	0	0	0	0	0	0	0	0	0	Fungi; Basidiomycota; Agaricomycetes; Auriculariales; unidentified; unidentified; Auriculariales_sp
New.ReferenceOTU2622	0	0	0	1	0	0	0	0	0	0	0	0	0	0	0	0	0	0	0	0	0	0	0	0	0	0	0	0	0	0	Fungi; Basidiomycota; Agaricomycetes; Auriculariales; unidentified; unidentified; Auriculariales_sp
New.ReferenceOTU2914	0	0	0	0	0	0	0	0	0	0	0	0	0	0	0	0	0	0	0	1	0	0	0	0	0	0	0	0	0	0	Fungi; Basidiomycota; Agaricomycetes; Auriculariales; unidentified; unidentified; Auriculariales_sp
New.ReferenceOTU3492	0	0	0	0	0	0	0	0	0	0	0	0	0	0	0	0	0	1	0	1	0	0	0	0	0	1	0	0	0	0	Fungi; Basidiomycota; Agaricomycetes; Auriculariales; unidentified; unidentified; Auriculariales_sp
New.ReferenceOTU3653	1	2	0	1	0	1	3	0	2	1	3	0	0	0	0	0	0	0	0	0	0	1	0	0	0	0	0	0	0	0	Fungi; Basidiomycota; Agaricomycetes; Auriculariales; unidentified; unidentified; Auriculariales_sp
New.ReferenceOTU3970	0	0	0	0	0	0	0	0	0	0	2	0	0	0	0	0	0	0	0	0	0	0	0	0	0	0	0	0	0	0	Fungi; Basidiomycota; Agaricomycetes; Auriculariales; unidentified; unidentified; Auriculariales_sp
New.ReferenceOTU4403	2	0	0	0	0	0	0	0	0	0	0	0	0	0	0	0	0	0	0	0	0	0	0	0	0	0	0	0	0	0	Fungi; Basidiomycota; Agaricomycetes; Auriculariales; unidentified; unidentified; Auriculariales_sp
New.ReferenceOTU4481	0	0	0	2	0	0	0	0	0	0	0	0	0	0	0	0	0	0	0	0	0	0	0	0	0	0	0	0	0	0	Fungi; Basidiomycota; Agaricomycetes; Auriculariales; unidentified; unidentified; Auriculariales_sp
New.ReferenceOTU4630	0	0	0	0	0	0	0	0	0	1	0	0	0	0	0	0	0	0	0	0	0	0	0	0	0	0	0	0	0	0	Fungi; Basidiomycota; Agaricomycetes; Auriculariales; unidentified; unidentified; Auriculariales_sp
New.ReferenceOTU5353	0	0	0	0	4	0	1	0	0	0	0	1	0	0	0	0	0	0	0	0	0	0	0	0	0	0	0	0	0	0	Fungi; Basidiomycota; Agaricomycetes; Auriculariales; unidentified; unidentified; Auriculariales_sp
New.ReferenceOTU5705	0	0	0	0	0	0	0	0	0	0	0	0	0	0	0	0	0	0	0	1	0	0	0	0	0	0	0	0	0	0	Fungi; Basidiomycota; Agaricomycetes; Auriculariales; unidentified; unidentified; Auriculariales_sp
New.ReferenceOTU619	0	0	0	0	0	0	0	0	0	0	0	0	0	0	0	0	0	0	0	0	0	0	0	5	0	0	0	0	0	0	Fungi; Basidiomycota; Agaricomycetes; Auriculariales; unidentified; unidentified; Auriculariales_sp
New.ReferenceOTU6689	0	0	0	0	0	0	0	0	0	0	0	0	0	0	0	0	0	3	5	0	0	0	0	0	0	0	0	0	0	0	Fungi; Basidiomycota; Agaricomycetes; Auriculariales; unidentified; unidentified; Auriculariales_sp
New.ReferenceOTU7480	0	0	0	0	1	1	0	0	0	0	0	0	0	0	0	0	0	0	0	0	0	0	0	0	0	0	0	0	0	0	Fungi; Basidiomycota; Agaricomycetes; Auriculariales; unidentified; unidentified; Auriculariales_sp
New.ReferenceOTU5702	0	0	0	0	0	0	0	0	0	0	0	0	0	0	0	0	0	0	0	4	0	0	0	0	0	0	0	0	0	0	Fungi; Basidiomycota; Agaricomycetes; Auriculariales; unidentified; unidentified; uncultured_Auriculariales
New.ReferenceOTU5532	3	0	0	0	0	0	0	0	0	0	0	0	0	0	0	0	0	0	0	0	0	0	0	0	0	0	0	0	0	0	Fungi; Basidiomycota; Agaricomycetes; Cantharellales; Ceratobasidiaceae; unidentified; Ceratobasidiaceae_sp
New.ReferenceOTU6804	0	0	0	0	0	0	0	0	0	0	0	0	0	0	0	0	0	0	0	2	0	0	0	0	0	0	0	0	0	0	Fungi; Basidiomycota; Agaricomycetes; Cantharellales; Tulasnellaceae; unidentified; Tulasnellaceae_sp
New.ReferenceOTU7229	0	0	0	0	0	0	0	0	0	1	0	0	0	0	0	0	0	0	0	0	0	0	0	0	0	0	0	0	0	0	Fungi; Basidiomycota; Agaricomycetes; Cantharellales; Tulasnellaceae; unidentified; Tulasnellaceae_sp
New.ReferenceOTU7733	0	0	0	0	0	0	0	0	0	0	0	0	0	0	0	0	0	0	1	0	0	0	0	0	0	0	0	0	0	0	Fungi; Basidiomycota; Agaricomycetes; Cantharellales; Tulasnellaceae; unidentified; uncultured_Tulasnellaceae
New.ReferenceOTU6065	0	0	0	0	0	0	0	0	0	0	0	0	0	0	0	0	0	0	0	0	0	0	0	0	0	0	0	0	0	1	Fungi; Basidiomycota; Agaricomycetes; Corticiales; Corticiaceae; unidentified; uncultured_Hyphodontia
New.ReferenceOTU698	1	0	0	0	0	0	0	0	0	0	0	0	0	0	0	0	0	0	0	0	0	0	0	0	0	0	0	0	0	0	Fungi; Basidiomycota; Agaricomycetes; Corticiales; Corticiaceae; unidentified; uncultured_Hyphodontia
New.ReferenceOTU1239	0	3	2	0	0	2	0	0	2	17	1	0	0	0	0	0	0	1	5	11	0	0	1	0	0	0	0	0	0	0	Fungi; Basidiomycota; Agaricomycetes; Geastrales; Geastraceae; unidentified; Geastraceae_sp
New.ReferenceOTU6826	0	0	0	0	0	0	0	0	0	0	0	0	0	0	0	0	0	0	0	1	0	0	0	0	0	0	0	0	0	0	Fungi; Basidiomycota; Agaricomycetes; Geastrales; Geastraceae; unidentified; Geastraceae_sp
New.ReferenceOTU4171	0	0	0	0	0	0	0	0	2	0	0	0	0	0	0	0	0	0	0	0	0	0	0	0	0	0	0	0	0	0	Fungi; Basidiomycota; Agaricomycetes; Gomphales; Gomphaceae; unidentified; uncultured_Ramaria
New.ReferenceOTU7483	0	1	0	0	0	0	0	0	0	0	0	0	0	0	0	0	0	0	0	0	0	0	0	0	0	0	0	0	0	0	Fungi; Basidiomycota; Agaricomycetes; Gomphales; Gomphaceae; unidentified; uncultured_Ramaria
New.ReferenceOTU749	0	1	0	0	0	0	0	0	0	0	0	0	0	0	0	0	0	0	0	0	0	0	0	0	0	0	0	0	0	0	Fungi; Basidiomycota; Agaricomycetes; Gomphales; Gomphaceae; unidentified; uncultured_Ramaria
New.ReferenceOTU8068	0	0	0	0	0	0	0	0	0	0	0	0	0	0	0	0	0	0	0	0	0	0	0	0	0	0	0	0	3	0	Fungi; Basidiomycota; Agaricomycetes; Hysterangiales; unidentified; unidentified; Hysterangiales_sp
New.ReferenceOTU476	0	0	1	0	0	0	0	0	0	0	0	0	0	0	0	0	0	0	0	2	0	0	0	0	0	0	12	0	0	0	Fungi; Basidiomycota; Agaricomycetes; Phallales; Phallaceae; unidentified; Phallaceae_sp
New.ReferenceOTU3224	1	0	0	0	1	0	0	0	1	0	0	0	0	1	0	0	0	0	0	0	1	0	0	0	0	0	0	1	0	1	Fungi; Basidiomycota; Agaricomycetes; Polyporales; unidentified; unidentified; Polyporales_sp
New.ReferenceOTU7893	0	0	0	0	0	0	0	0	0	0	0	0	0	0	0	0	0	0	0	0	0	0	0	0	0	0	0	4	0	0	Fungi; Basidiomycota; Agaricomycetes; Polyporales; unidentified; unidentified; Polyporales_sp
SH116458.06FU_EU085031_reps	1	0	0	0	0	0	0	0	0	0	0	0	0	0	0	0	0	0	0	0	0	0	0	0	0	0	0	1	0	0	Fungi; Basidiomycota; Agaricomycetes; Polyporales; unidentified; unidentified; Polyporales_sp
New.ReferenceOTU668	0	0	0	0	0	0	0	0	0	0	0	0	0	1	0	0	0	0	0	0	0	0	0	0	0	0	0	0	0	0	Fungi; Basidiomycota; Agaricomycetes; Polyporales; unidentified; unidentified; uncultured_Polyporales
New.ReferenceOTU4684	0	0	0	0	0	0	0	0	0	0	1	0	0	0	0	0	0	0	0	0	0	0	0	0	0	0	0	0	0	0	Fungi; Basidiomycota; Agaricomycetes; Sebacinales; Sebacinaceae; unidentified; uncultured_Sebacina
New.ReferenceOTU4487	1	0	0	0	0	0	0	0	0	0	0	0	0	0	0	0	0	0	0	0	0	0	0	0	0	0	0	0	0	0	Fungi; Basidiomycota; Agaricomycetes; Sebacinales; unidentified; unidentified; Sebacinales_sp
New.ReferenceOTU5798	0	0	0	0	0	0	0	0	0	1	0	0	0	0	0	0	0	0	0	0	0	0	0	0	0	0	0	0	0	0	Fungi; Basidiomycota; Agaricomycetes; Sebacinales; unidentified; unidentified; Sebacinales_sp
New.ReferenceOTU6595	0	0	0	0	0	0	0	0	0	0	0	0	0	0	0	0	0	0	0	0	0	0	0	4	0	0	0	0	0	0	Fungi; Basidiomycota; Agaricomycetes; Thelephorales; Thelephoraceae; unidentified; Thelephoraceae_sp
SH092683.06FU_HE979348_reps	0	0	0	0	0	0	0	0	0	0	0	0	0	0	0	0	0	0	0	0	0	0	0	2	0	0	0	0	0	0	Fungi; Basidiomycota; Agaricomycetes; Thelephorales; Thelephoraceae; unidentified; Thelephoraceae_sp
New.CleanUp.ReferenceOTU1199	0	0	0	0	0	0	0	0	0	0	0	0	0	0	0	0	1	0	0	0	0	0	0	0	0	0	0	0	0	0	Fungi; Basidiomycota; Agaricomycetes; Trechisporales; unidentified; unidentified; Trechisporales_sp
New.ReferenceOTU1150	9	0	0	0	0	0	0	0	0	0	0	0	2	0	0	0	0	0	0	0	0	0	0	0	0	0	0	0	0	0	Fungi; Basidiomycota; Agaricomycetes; Trechisporales; unidentified; unidentified; Trechisporales_sp
New.ReferenceOTU1719	0	0	0	0	0	0	0	0	0	0	0	0	0	0	0	0	0	0	0	0	0	0	0	0	0	2	0	0	0	0	Fungi; Basidiomycota; Agaricomycetes; Trechisporales; unidentified; unidentified; Trechisporales_sp
New.ReferenceOTU1814	0	0	0	0	0	1	0	1	0	0	0	0	0	0	0	0	0	0	0	0	2	1	0	0	0	0	2	3	0	0	Fungi; Basidiomycota; Agaricomycetes; Trechisporales; unidentified; unidentified; Trechisporales_sp
New.ReferenceOTU188	0	0	0	0	0	0	0	0	0	0	0	1	0	0	0	0	0	0	0	0	0	0	0	0	0	0	0	0	0	0	Fungi; Basidiomycota; Agaricomycetes; Trechisporales; unidentified; unidentified; Trechisporales_sp
New.ReferenceOTU1996	1	0	0	0	0	0	0	0	0	0	0	0	0	0	0	0	0	0	0	0	0	0	0	0	0	0	0	0	0	0	Fungi; Basidiomycota; Agaricomycetes; Trechisporales; unidentified; unidentified; Trechisporales_sp
New.ReferenceOTU305	0	0	0	0	0	0	0	0	0	0	0	0	0	0	0	0	0	0	0	0	2	0	0	0	0	0	0	2	0	0	Fungi; Basidiomycota; Agaricomycetes; Trechisporales; unidentified; unidentified; Trechisporales_sp
New.ReferenceOTU3555	0	0	0	0	0	2	0	0	0	0	0	2	0	0	0	0	0	0	0	0	4	1	0	0	0	0	0	0	0	0	Fungi; Basidiomycota; Agaricomycetes; Trechisporales; unidentified; unidentified; Trechisporales_sp
New.ReferenceOTU3591	0	0	0	0	0	0	0	0	0	1	1	0	0	0	0	0	0	0	0	0	0	0	0	0	0	0	0	2	0	0	Fungi; Basidiomycota; Agaricomycetes; Trechisporales; unidentified; unidentified; Trechisporales_sp
New.ReferenceOTU3721	0	0	0	0	0	0	0	0	0	0	0	0	1	0	0	0	0	0	1	0	0	0	1	0	0	0	0	0	0	0	Fungi; Basidiomycota; Agaricomycetes; Trechisporales; unidentified; unidentified; Trechisporales_sp
New.ReferenceOTU589	0	0	0	4	0	0	0	0	0	0	0	0	0	0	0	0	0	0	0	0	0	0	0	0	0	0	0	0	0	0	Fungi; Basidiomycota; Agaricomycetes; Trechisporales; unidentified; unidentified; Trechisporales_sp
SH122917.06FU_AY627800_reps	0	0	0	0	0	0	0	1	0	0	0	0	0	0	0	0	0	0	0	0	6	2	0	0	0	0	0	14	0	0	Fungi; Basidiomycota; Agaricomycetes; Trechisporales; unidentified; unidentified; Trechisporales_sp
SH122918.06FU_JN890153_reps	2	0	0	0	0	0	0	0	0	0	0	0	0	0	2	0	0	0	0	0	0	0	0	0	0	0	0	0	0	0	Fungi; Basidiomycota; Agaricomycetes; Trechisporales; unidentified; unidentified; Trechisporales_sp
New.ReferenceOTU6941	0	0	0	0	0	0	0	0	0	0	1	0	0	0	0	0	0	0	0	0	0	0	0	0	0	0	0	0	0	0	Fungi; Basidiomycota; Agaricomycetes; Trechisporales; unidentified; unidentified; uncultured_Trechisporales

New.ReferenceOTU1206	1	0	0	0	0	0	0	0	0	0	0	0	0	0	0	0	0	0	0	0	0	0	0	0	0	0	0	0	0	0	Fungi; Basidiomycota; Agaricomycetes; unidentified; unidentified; unidentified; Agaricomycetes_sp
New.ReferenceOTU1665	1	0	0	0	0	0	0	0	0	0	0	0	0	0	0	0	0	0	0	1	0	0	0	0	0	0	0	0	0	0	Fungi; Basidiomycota; Agaricomycetes; unidentified; unidentified; unidentified; Agaricomycetes_sp
New.ReferenceOTU1962	0	0	0	0	0	0	0	0	0	0	0	0	0	0	0	0	0	0	0	1	0	0	0	0	0	0	0	0	0	0	Fungi; Basidiomycota; Agaricomycetes; unidentified; unidentified; unidentified; Agaricomycetes_sp
New.ReferenceOTU2180	0	0	0	0	0	0	0	0	0	0	0	1	0	0	0	0	0	0	0	0	0	0	0	0	0	0	0	0	0	0	Fungi; Basidiomycota; Agaricomycetes; unidentified; unidentified; unidentified; Agaricomycetes_sp
New.ReferenceOTU2540	0	0	0	0	0	0	0	0	0	0	0	0	0	0	0	1	0	0	0	3	0	0	0	0	0	0	0	0	0	0	Fungi; Basidiomycota; Agaricomycetes; unidentified; unidentified; unidentified; Agaricomycetes_sp
New.ReferenceOTU3346	0	1	0	0	0	0	0	0	0	0	0	0	0	0	0	0	0	0	0	0	0	0	0	5	0	0	0	0	0	0	Fungi; Basidiomycota; Agaricomycetes; unidentified; unidentified; unidentified; Agaricomycetes_sp
New.ReferenceOTU4374	0	0	0	0	0	0	0	0	0	0	0	0	0	0	2	0	0	0	1	0	0	0	0	0	0	0	0	0	0	0	Fungi; Basidiomycota; Agaricomycetes; unidentified; unidentified; unidentified; Agaricomycetes_sp
New.ReferenceOTU4802	6	0	0	0	0	0	0	0	0	0	0	0	0	0	0	0	0	0	0	0	0	0	0	0	0	0	0	0	0	0	Fungi; Basidiomycota; Agaricomycetes; unidentified; unidentified; unidentified; Agaricomycetes_sp
New.ReferenceOTU5478	1	0	0	0	0	0	0	0	0	0	0	0	0	0	0	0	0	0	0	0	0	0	0	0	0	0	0	0	0	0	Fungi; Basidiomycota; Agaricomycetes; unidentified; unidentified; unidentified; Agaricomycetes_sp
New.ReferenceOTU6213	0	0	0	0	0	0	1	0	0	0	0	0	0	0	2	0	0	0	0	0	0	0	0	0	0	0	0	0	0	0	Fungi; Basidiomycota; Agaricomycetes; unidentified; unidentified; unidentified; Agaricomycetes_sp
New.ReferenceOTU7876	0	0	0	0	0	0	0	0	0	0	0	0	0	0	0	0	0	0	0	0	0	1	0	0	0	0	0	0	0	0	Fungi; Basidiomycota; Agaricomycetes; unidentified; unidentified; unidentified; Agaricomycetes_sp
SH103692.06FU_DQ647440_reps	0	0	0	0	2	0	0	0	0	0	0	0	0	0	0	0	0	0	0	0	0	0	0	0	0	0	0	0	0	0	Fungi; Basidiomycota; Agaricomycetes; unidentified; unidentified; unidentified; Agaricomycetes_sp
New.ReferenceOTU90	0	0	0	0	0	0	0	0	0	0	0	0	0	0	0	0	0	0	1	0	0	0	0	0	0	0	0	0	0	0	Fungi; Basidiomycota; Agaricostilbomycetes; Agaricostilbales; Chionosphaeraceae; Kurtzmanomyces; Kurtzmanomyces_insolitus
SH110187.06FU_FJ524317_reps	0	0	0	0	0	0	0	0	0	0	0	0	0	0	0	0	0	0	0	2	0	0	0	0	0	0	0	0	0	0	Fungi; Basidiomycota; Atractiellomycetes; Atractiellales; unidentified; unidentified; Atractiellales_sp
New.ReferenceOTU3415	0	0	0	0	0	0	0	0	0	0	0	0	0	0	1	0	0	0	0	0	0	0	0	0	1	0	0	0	0	0	Fungi; Basidiomycota; Atractiellomycetes; Atractiellales; unidentified; unidentified; Atractiellales_sp_WIM(M)1397
New.ReferenceOTU1320	0	0	0	0	0	0	0	0	0	0	0	0	0	0	0	0	0	0	0	0	0	1	0	0	0	0	0	0	0	0	Fungi; Basidiomycota; Dacrymycetes; Dacrymycetales; Dacrymycetaceae; Dacrymyces; Dacrymyces_adpressus
New.ReferenceOTU4531	0	0	0	0	1	0	0	1	0	0	0	0	0	0	0	0	0	0	0	0	0	0	0	0	0	0	0	0	0	0	Fungi; Basidiomycota; Dacrymycetes; Dacrymycetales; Dacrymycetaceae; Dacrymyces; Dacrymyces_dendrocalami
New.ReferenceOTU4032	0	0	0	0	0	0	0	0	0	0	0	0	0	0	0	0	0	0	0	1	0	0	0	0	0	0	0	0	0	0	Fungi; Basidiomycota; Dacrymycetes; Dacrymycetales; Dacrymycetaceae; Dacrymyces; Dacrymyces_san_augustinii
SH110868.06FU_JX432963_refs	0	0	0	0	0	0	0	0	0	0	0	0	0	0	0	0	0	0	0	0	0	0	0	0	1	0	0	0	0	0	Fungi; Basidiomycota; Exobasidiomycetes; Incertae_sedis; Incertae_sedis; Meira; Meira_miltonrushii
SH120972.06FU_JX042766_reps	0	0	0	0	0	0	0	1	0	0	0	0	0	0	0	0	0	0	0	0	0	0	0	0	0	0	0	0	0	0	Fungi; Basidiomycota; Incertae_sedis; Malasseziales; unidentified; unidentified; Malasseziales_sp
New.ReferenceOTU1315	0	1	0	2	0	0	0	0	0	0	0	0	0	0	0	0	0	0	0	0	0	0	0	0	0	9	0	0	3	0	Fungi; Basidiomycota; Microbotryomycetes; Sporidiobolales; unidentified; unidentified; Sporidiobolales_sp
New.ReferenceOTU1506	0	0	0	0	0	0	0	0	0	0	0	0	0	0	0	0	0	0	0	0	0	0	0	0	0	2	0	0	3	0	Fungi; Basidiomycota; Microbotryomycetes; Sporidiobolales; unidentified; unidentified; Sporidiobolales_sp
New.ReferenceOTU2244	0	5	0	0	0	0	0	0	0	0	0	0	0	0	0	0	0	0	0	0	0	0	0	0	0	0	0	0	0	0	Fungi; Basidiomycota; Microbotryomycetes; Sporidiobolales; unidentified; unidentified; Sporidiobolales_sp
New.ReferenceOTU3345	0	0	0	0	0	0	0	0	0	0	0	0	0	0	0	0	0	4	0	0	0	0	0	0	0	0	0	0	0	0	Fungi; Basidiomycota; Microbotryomycetes; Sporidiobolales; unidentified; unidentified; Sporidiobolales_sp
New.ReferenceOTU3886	0	1	0	0	0	0	0	0	0	0	0	0	0	0	0	0	0	0	0	0	0	0	0	0	1	55	0	0	26	0	Fungi; Basidiomycota; Microbotryomycetes; Sporidiobolales; unidentified; unidentified; Sporidiobolales_sp
New.ReferenceOTU5163	0	0	0	0	0	0	0	0	0	0	0	0	0	0	0	0	0	2	0	0	0	0	0	0	0	0	0	0	0	0	Fungi; Basidiomycota; Microbotryomycetes; Sporidiobolales; unidentified; unidentified; Sporidiobolales_sp
New.ReferenceOTU6215	0	0	0	0	0	0	0	0	0	0	0	0	0	0	0	0	0	14	0	0	0	0	0	0	0	0	0	0	0	0	Fungi; Basidiomycota; Microbotryomycetes; Sporidiobolales; unidentified; unidentified; Sporidiobolales_sp
New.ReferenceOTU1135	0	0	0	2	0	0	0	0	0	0	0	0	0	0	0	0	0	0	0	0	0	0	0	0	0	0	0	0	0	0	Fungi; Basidiomycota; Pucciniomycetes; unidentified; unidentified; unidentified; Pucciniomycetes_sp_PIMO_247
New.ReferenceOTU6194	0	2	0	0	0	0	0	0	0	0	0	0	0	0	0	0	0	0	0	0	0	0	0	0	0	0	0	0	0	0	Fungi; Basidiomycota; Tremellomycetes; Trichosporonales; Trichosporonaceae; Cryptotrichosporon; Cryptotrichosporon_anacardii
New.ReferenceOTU7327	0	0	0	0	0	0	0	0	0	0	0	0	0	0	0	0	0	0	0	0	0	0	0	0	0	0	0	0	0	1	Fungi; Basidiomycota; Tremellomycetes; Trichosporonales; Trichosporonaceae; Cryptotrichosporon; Cryptotrichosporon_anacardii
New.CleanUp.ReferenceOTU11	0	0	0	1	0	0	0	0	0	0	0	0	0	0	0	0	0	0	0	0	0	0	0	0	0	0	0	0	0	0	Fungi; Basidiomycota; Tremellomycetes; unidentified; unidentified; unidentified; Tremellomycetes_sp
New.ReferenceOTU1178	0	0	0	0	0	0	0	0	0	0	0	0	0	0	0	0	0	0	1	1	0	0	0	0	0	0	0	0	0	0	Fungi; Basidiomycota; Tremellomycetes; unidentified; unidentified; unidentified; Tremellomycetes_sp
New.ReferenceOTU1435	0	0	0	0	0	0	0	0	0	0	0	0	0	0	0	0	0	0	0	0	0	0	0	0	0	0	0	0	1	0	Fungi; Basidiomycota; Tremellomycetes; unidentified; unidentified; unidentified; Tremellomycetes_sp
New.ReferenceOTU173	0	3	1	1	0	0	0	0	0	0	0	1	0	0	0	0	0	0	0	0	0	0	0	4	0	0	0	0	2	0	Fungi; Basidiomycota; Tremellomycetes; unidentified; unidentified; unidentified; Tremellomycetes_sp
New.ReferenceOTU2542	0	0	0	0	0	0	0	1	0	0	0	0	0	0	0	0	0	0	0	0	0	0	0	0	0	0	0	0	0	0	Fungi; Basidiomycota; Tremellomycetes; unidentified; unidentified; unidentified; Tremellomycetes_sp
New.ReferenceOTU2588	0	0	0	0	0	0	0	0	0	0	0	0	0	0	0	0	0	0	0	0	0	0	1	0	0	0	0	0	0	0	Fungi; Basidiomycota; Tremellomycetes; unidentified; unidentified; unidentified; Tremellomycetes_sp
New.ReferenceOTU2924	0	0	0	0	0	0	0	0	0	0	0	0	1	0	0	0	0	0	0	0	0	0	0	0	0	0	0	0	0	0	Fungi; Basidiomycota; Tremellomycetes; unidentified; unidentified; unidentified; Tremellomycetes_sp
New.ReferenceOTU2925	0	0	1	0	1	2	0	0	0	0	0	0	0	0	0	0	0	0	0	0	0	0	0	0	0	0	0	0	0	0	Fungi; Basidiomycota; Tremellomycetes; unidentified; unidentified; unidentified; Tremellomycetes_sp
New.ReferenceOTU3192	1	1	0	0	0	0	0	7	1	0	0	0	0	0	0	0	1	0	1	0	0	0	0	0	0	0	0	0	0	0	Fungi; Basidiomycota; Tremellomycetes; unidentified; unidentified; unidentified; Tremellomycetes_sp
New.ReferenceOTU3236	0	0	0	0	0	0	0	0	0	0	2	0	0	0	0	0	0	0	0	0	0	0	0	0	0	0	0	0	0	0	Fungi; Basidiomycota; Tremellomycetes; unidentified; unidentified; unidentified; Tremellomycetes_sp
New.ReferenceOTU3635	0	0	0	0	0	0	0	0	0	0	0	0	0	0	0	0	0	0	0	1	0	0	0	0	0	0	0	0	0	0	Fungi; Basidiomycota; Tremellomycetes; unidentified; unidentified; unidentified; Tremellomycetes_sp
New.ReferenceOTU3870	0	0	0	0	0	0	0	0	0	0	0	0	0	0	0	0	0	0	0	3	0	0	0	0	0	0	0	0	0	0	Fungi; Basidiomycota; Tremellomycetes; unidentified; unidentified; unidentified; Tremellomycetes_sp
New.ReferenceOTU4010	0	0	0	0	0	0	0	1	0	0	0	0	0	0	0	0	0	0	0	0	0	0	0	0	0	0	0	0	0	0	Fungi; Basidiomycota; Tremellomycetes; unidentified; unidentified; unidentified; Tremellomycetes_sp
New.ReferenceOTU405	1	0	0	1	0	0	0	0	0	0	0	0	0	0	1	0	0	1	1	0	0	1	0	0	0	0	0	0	0	0	Fungi; Basidiomycota; Tremellomycetes; unidentified; unidentified; unidentified; Tremellomycetes_sp
New.ReferenceOTU4117	0	0	0	0	0	0	0	0	1	0	0	0	0	0	0	0	1	0	0	0	0	0	0	1	0	0	0	0	0	0	Fungi; Basidiomycota; Tremellomycetes; unidentified; unidentified; unidentified; Tremellomycetes_sp
New.ReferenceOTU6055	0	0	0	0	0	0	0	0	0	0	0	0	0	0	0	0	0	2	0	0	0	0	0	0	0	0	0	0	0	0	Fungi; Basidiomycota; Tremellomycetes; unidentified; unidentified; unidentified; Tremellomycetes_sp
New.ReferenceOTU657	0	0	0	0	0	0	0	0	0	0	0	0	0	0	0	0	0	0	0	2	0	0	0	0	0	0	0	0	0	0	Fungi; Basidiomycota; Tremellomycetes; unidentified; unidentified; unidentified; Tremellomycetes_sp
New.ReferenceOTU6627	1	4	0	2	0	0	2	0	0	0	0	0	9	0	0	0	0	0	0	0	0	0	0	0	0	0	0	0	0	0	Fungi; Basidiomycota; Tremellomycetes; unidentified; unidentified; unidentified; Tremellomycetes_sp
New.ReferenceOTU6720	0	0	0	0	1	0	0	0	0	0	0	0	0	0	0	0	0	0	0	0	0	0	0	0	0	0	0	0	0	0	Fungi; Basidiomycota; Tremellomycetes; unidentified; unidentified; unidentified; Tremellomycetes_sp
New.ReferenceOTU7398	0	0	0	0	0	0	1	0	0	0	0	0	0	0	0	0	0	0	0	0	0	0	0	0	0	0	0	0	0	0	Fungi; Basidiomycota; Tremellomycetes; unidentified; unidentified; unidentified; Tremellomycetes_sp
New.ReferenceOTU7600	0	0	0	5	0	1	0	0	0	0	0	0	0	0	0	0	0	0	0	0	0	0	0	0	0	0	0	0	0	0	Fungi; Basidiomycota; Tremellomycetes; unidentified; unidentified; unidentified; Tremellomycetes_sp
New.ReferenceOTU810	0	0	0	0	0	0	0	0	0	0	0	0	0	0	0	0	0	0	1	0	0	0	0	0	0	0	0	0	0	0	Fungi; Basidiomycota; Tremellomycetes; unidentified; unidentified; unidentified; Tremellomycetes_sp
New.ReferenceOTU948	1	0	0	0	0	0	0	0	0	0	0	0	0	0	0	0	0	0	0	0	0	0	0	0	0	0	0	0	0	0	Fungi; Basidiomycota; Tremellomycetes; unidentified; unidentified; unidentified; Tremellomycetes_sp
New.ReferenceOTU978	0	0	0	0	0	0	0	0	0	0	0	0	0	0	0	0	0	0	0	0	0	0	1	0	0	0	0	0	0	0	Fungi; Basidiomycota; Tremellomycetes; unidentified; unidentified; unidentified; Tremellomycetes_sp
New.ReferenceOTU993	0	0	0	2	0	0	0	0	0	0	0	0	0	0	0	0	0	0	0	0	0	0	0	0	0	0	0	0	1	0	Fungi; Basidiomycota; Tremellomycetes; unidentified; unidentified; unidentified; Tremellomycetes_sp
New.CleanUp.ReferenceOTU1447	0	0	0	1	0	0	0	0	0	0	0	0	0	0	0	0	0	0	0	0	0	0	0	0	0	0	0	0	0	0	Fungi; Basidiomycota; unidentified; unidentified; unidentified; unidentified; Basidiomycota_sp
New.ReferenceOTU3174	0	0	0	5	0	0	0	0	0	0	0	0	0	0	0	0	0	0	0	0	0	0	0	0	0	0	0	0	0	0	Fungi; Basidiomycota; unidentified; unidentified; unidentified; unidentified; Basidiomycota_sp
New.ReferenceOTU5014	1	0	0	0	0	0	0	0	0	0	0	0	0	0	0	0	0	0	0	0	0	0	0	0	0	0	0	0	0	0	Fungi; Basidiomycota; unidentified; unidentified; unidentified; unidentified; Basidiomycota_sp
New.ReferenceOTU5897	2	0	0	0	1	0	0	0	0	0	0	0	0	0	0	2	1	0	0	0	0	0	0	0	0	0	0	0	0	0	Fungi; Basidiomycota; unidentified; unidentified; unidentified; unidentified; Basidiomycota_sp
New.ReferenceOTU6273	0	0	1	0	0	0	0	0	0	0	0	0	0	0	0	0	0	0	0	0	0	0	0	0	0	0	0	0	0	0	Fungi; Basidiomycota; unidentified; unidentified; unidentified; unidentified; Basidiomycota_sp
New.ReferenceOTU7819	2	0	0	0	0	0	0	0	0	0	0	0	0	0	0	0	0	0	0	0	0	0	0	0	0	0	0	0	0	0	Fungi; Basidiomycota; unidentified; unidentified; unidentified; unidentified; Basidiomycota_sp
SH016074.06FU_JN889960_reps_singleton	6	0	0	1	0	0	0	0	0	0	0	0	0	0	0	2	0	0	0	0	0	0	0	0	0	0	0	0	0	0	Fungi; Basidiomycota; unidentified; unidentified; unidentified; unidentified; Basidiomycota_sp
New.ReferenceOTU6088	2	0	0	0	0	0	0	0	0	0	0	0	0	0	0	0	0	0	0	1	0	0	0	0	0	0	0	0	0	0	Fungi; Basidiomycota; unidentified; unidentified; unidentified; unidentified; Basidiomycota_sp_2_RB_2011
New.CleanUp.ReferenceOTU2139	0	0	0	0	0	0	0	0	0	0	0	0	0	0	0	0	0	0	0	0	0	0	0	1	0	0	0	0	0	0	Fungi; Basidiomycota; unidentified; unidentified; unidentified; unidentified; leaf_litter_basidiomycete_sp_13/2_48
New.ReferenceOTU1351	0	0	0	0	0	0	0	0	0	0	0	0	0	0	0	0	0	0	0	0	0	0	0	0	0	0	0	0	6	0	Fungi; Basidiomycota; unidentified; unidentified; unidentified; unidentified; uncultured_Basidiomycota
New.ReferenceOTU2320	1	0	0	0	0	0	0	0	0	0	0	0	0	0	0	0	0	0	0	0	1	0	0	0	0	0	0	0	0	0	Fungi; Basidiomycota; unidentified; unidentified; unidentified; unidentified; uncultured_Basidiomycota
New.ReferenceOTU316	0	0	0	0	0	0	0	0	0	0	0	0	0	0	0	1	0	0	0	0	0	0	0	0	0	0	0	0	0	0	Fungi; Basidiomycota; unidentified; unidentified; unidentified; unidentified; uncultured_Basidiomycota
New.ReferenceOTU4639	0	0	0	0	0	0	0	0	4	0	0	0	0	0	0	0	0	0	0	0	0	0	0	0	0	0	0	0	0	0	Fungi; Basidiomycota; unidentified; unidentified; unidentified; unidentified; uncultured_Basidiomycota
New.ReferenceOTU5464	1	0	0	0	0	0	0	0	0	0	0	0	0	0	0	0	0	0	0	0	0	0	0	0	0	0	0	0	0	0	Fungi; Basidiomycota; unidentified; unidentified; unidentified; unidentified; uncultured_Basidiomycota
New.ReferenceOTU5536	0	0	0	0	0	0	0	0	0	0	0	0	0	0	0	0	0	0	0	0	0	0	0	0	0	1	0	0	0	0	Fungi; Basidiomycota; unidentified; unidentified; unidentified; unidentified; uncultured_Basidiomycota
New.ReferenceOTU6603	0	0	0	0	0	0	0	0	0	1	0	0	0	0	0	0	0	0	0	0	0	0	0	0	0	0	0	0	0	0	Fungi; Basidiomycota; unidentified; unidentified; unidentified; unidentified; uncultured_Basidiomycota
New.ReferenceOTU6655	0	0	0	0	0	0	0	0	1	0	0	0	0	0	0	0	0	0	0	0	0	0	0	0	0	0	0	0	0	0	Fungi; Basidiomycota; unidentified; unidentified; unidentified; unidentified; uncultured_Basidiomycota
New.ReferenceOTU6794	0	0	0	1	0	0	0	0	0	0	0	0	0	0	0	0	0	0	0	0	0	0	0	0	0	0	0	2	0	0	Fungi; Basidiomycota; unidentified; unidentified; unidentified; unidentified; uncultured_Basidiomycota
New.ReferenceOTU6989	0	0	0	0	0	0	0	0	0	0	0	0	0	0	0	0	0	0	1	0	0	0	0	0	0	0	0	0	0	0	Fungi; Basidiomycota; unidentified; unidentified; unidentified; unidentified; uncultured_Basidiomycota
New.ReferenceOTU7270	0	0	0	0	0	0	0	0	0	0	1	1	0	0	0	0	0	0	0	0	0	0	0	0	0	0	0	0	0	0	Fungi; Basidiomycota; unidentified; unidentified; unidentified; unidentified; uncultured_Basidiomycota
New.ReferenceOTU7275	0	0	0	0	0	0	0	0	0	1	0	0	0	2	0	0	0	0	0	0	0	0	0	0	0	0	0	0	0	0	Fungi; Chytridiomycota; Chytridiomycetes; Chytridiales; unidentified; unidentified; Chytridiales_sp
New.ReferenceOTU6914	0	0	0	0	0	0	2	0	0	0	0	0	0	0	0	0	0	0	0	0	0	0	0	0	0	0	0	0	0	0	Fungi; Chytridiomycota; Chytridiomycetes; Chytridiales; unidentified; unidentified; Chytridiales_sp_1_MV_2011
New.ReferenceOTU7181	0	0	0	0	0	0	0	0	0	0	0	0	0	0	0	0	0	0	0	0	0	0	0	0	2	0	0	0	0	0	Fungi; Chytridiomycota; Chytridiomycetes; Chytridiales; unidentified; unidentified; Chytridiales_sp_1_MV_2011
New.ReferenceOTU3170	0	0	0	0	0	0	0	1	0	0	0	0	0	0	0	0	0	0	0	0	0	0	0	2	0	0	0	0	0	0	Fungi; Chytridiomycota; Chytridiomycetes; Spizellomycetales; unidentified; unidentified; Spizellomycete_sp_JEL355
New.CleanUp.ReferenceOTU1057	0	0	0	0	0	0	0	0	0	0	0	0	0	0	0	0	0	0	0	0	0	0	0	0	0	0	0	0	0	1	Fungi; Chytridiomycota; Chytridiomycetes; unidentified; unidentified; unidentified; Chytridiomycetes_sp
New.ReferenceOTU340	0	0	0	0	0	0	0	2	1	0	0	0	0	0	0	0	0	0	0	0	1	0	0	0	0	0	0	0	0	0	Fungi; Chytridiomycota; Chytridiomycetes; unidentified; unidentified; unidentified; Chytridiomycetes_sp
New.ReferenceOTU4477	0	0	0	0	0	1	0	0	0	0	0	0	1	0	0	0	0	0	0	0	0	0	0	0	0	0	0	0	0	0	Fungi; Chytridiomycota; Chytridiomycetes; unidentified; unidentified; unidentified; Chytridiomycetes_sp
New.ReferenceOTU5741	0	0	0	0	0	0	0	0	0	0	0	0	0	0	0	0	0	0	0	0	0	1	0	0	0	0	0	0	0	0	Fungi; Chytridiomycota; Chytridiomycetes; unidentified; unidentified; unidentified; Chytridiomycetes_sp
New.ReferenceOTU6395	0	0	0	0	0	0	0	0	0	0	0	0	0	0	0	0	0	0	0	0	0	0	0	0	0	0	0	2	0	0	Fungi; Chytridiomycota; Chytridiomycetes; unidentified; unidentified; unidentified; Chytridiomycetes_sp
New.CleanUp.ReferenceOTU1577	0	0	0	1	0	0	0	0	0	0	0	0	0	0	0	0	0	0	0	0	0	0	0	0	0	0	0	0	0	0	Fungi; Chytridiomycota; Monoblepharidomycetes; unidentified; unidentified; unidentified; Monoblepharidomycetes_sp
New.CleanUp.ReferenceOTU2151	0	0	0	0	0	0	0	0	0	0	0	0	0	0	0	0	0	0	0	0	0	1	0	0	0	0	0	0	0	0	Fungi; Chytridiomycota; unidentified; unidentified; unidentified; unidentified; Chytridiomycota_sp
New.ReferenceOTU3881	1	0	0	0	0	0	0	0	0	0	0	0	0	0	0	0	0	0	0	0	0	0	0	0	0	0	0	0	0	0	Fungi; Chytridiomycota; unidentified; unidentified; unidentified; unidentified; Chytridiomycota_sp
New.ReferenceOTU2462	0	0	0	1	0	0	0	0	0	0	0	0	0	0	0	0	0	0	0	0	0	0	0	0	0	0	0	0	0	0	Fungi; Chytridiomycota; unidentified; unidentified; unidentified; unidentified; Chytridiomycota_sp_Mori_B3
New.ReferenceOTU1272	0	0	0	0	0	0	0	0	0	0	0	0	0	0	0	0	0	0	4	0	0	0	0	0	0	0	0	0	0	0	Fungi; Chytridiomycota; unidentified; unidentified; unidentified; unidentified; uncultured_Chytridiomycota
New.ReferenceOTU199	0	0	0	0	0	0	0	0	0	0	0	0	0	0	0	0	0	0	4	2	0	0	0	0	0	0	0	0	0	0	Fungi; Chytridiomycota; unidentified; unidentified; unidentified; unidentified; uncultured_Chytridiomycota
New.ReferenceOTU6352	2	0	0	0	0	0	0	0	0	0	0	0	0	0	0	0	0	0	0	0	0	0	0	0	0	0	0	0	0	0	Fungi; Chytridiomycota; unidentified; unidentified; unidentified; unidentified; uncultured_Chytridiomycota
New.ReferenceOTU7459	0	0	0	0	1	0	0	0	0	0	0	0	0	0	0	0	0	0	0	0	0	0	0	0	0	0	0	0	0	0	Fungi; Chytridiomycota; unidentified; unidentified; unidentified; unidentified; uncultured_Chytridiomycota
New.ReferenceOTU7959	0	0	0	0	0	0	0	0	0	0	0	0	0	0	1	0	0	0	0	0	0	0	0	0	0	0	0	0	0	0	Fungi; unidentified; unidentified; unidentified; unidentified; unidentified; Basal_lineages
New.ReferenceOTU2842	0	0	0	0	0	0	0	0	0	1	1	0	2	0	0	0	0	0	0	0	0	2	0	0	0	0	0	0	0	0	Fungi; unidentified; unidentified; unidentified; unidentified; unidentified; Calluna_vulgaris_root_associated_fungus
New.ReferenceOTU4468	0	0	1	0	0	0	0	0	0	0	0	1	2	0	0	3	0	0	0	0	0	11	0	0	0	0	0	1	0	1	Fungi; unidentified; unidentified; unidentified; unidentified; unidentified; Calluna_vulgaris_root_associated_fungus
New.ReferenceOTU5037	0	0	0	0	0	0	0	0	0	0	0	0	0	0	0	1	0	0	0	0	0	1	0	0	0	0	0	0	0	1	Fungi; unidentified; unidentified; unidentified; unidentified; unidentified; Calluna_vulgaris_root_associated_fungus
SH015872.06FU_FM172811_reps_singleton	0	0	0	0	0	0	0	1	0	11	4	0	1	0	0	0	1	0	0	0	0	1	3	0	1	0	1	1	0	0	Fungi; unidentified; unidentified; unidentified; unidentified; unidentified; Calluna_vulgaris_root_associated_fungus
New.ReferenceOTU1112	0	0	0	1	0	0	0	0	0	0	0	0	0	0	0	0	0	0	0	0	0	0	0	1	0	0	0	0	0	0	Fungi; unidentified; unidentified; unidentified; unidentified; unidentified; Epacris_microphylla_root_associated_fungus_19
New.ReferenceOTU1174	0	0	0	0	1	0	0	1	0	0	0	1	0	3	0	2	0	0	0	0	0	0	0	1	0	0	0	0	0	0	Fungi; unidentified; unidentified; unidentified; unidentified; unidentified; fungal_endophyte
New.ReferenceOTU1493	0	0	0	0	0	0	0	0	0	0	0	1	0	0	0	0	0	0	0	0	0	0	0	0	0	0	0	0	0	0	Fungi; unidentified; unidentified; unidentified; unidentified; unidentified; fungal_endophyte

New.ReferenceOTU1548	0	0	0	0	0	0	0	0	1	0	0	0	0	0	0	0	0	0	0	0	0	0	0	0	0	0	0	0	0	0	Fungi; unidentified; unidentified; unidentified; unidentified; unidentified; fungal_endophyte
New.ReferenceOTU1590	0	0	0	0	0	0	0	0	0	0	0	0	5	0	0	0	0	0	0	0	0	0	0	0	0	0	0	0	0	0	Fungi; unidentified; unidentified; unidentified; unidentified; unidentified; fungal_endophyte
New.ReferenceOTU2445	0	0	0	0	0	0	0	0	0	0	0	0	0	0	0	0	0	0	0	0	0	0	1	0	0	0	0	0	0	1	Fungi; unidentified; unidentified; unidentified; unidentified; unidentified; fungal_endophyte
New.ReferenceOTU2483	0	0	0	0	1	0	1	0	0	0	0	2	0	1	0	0	0	0	0	0	0	0	0	0	0	0	0	0	0	0	Fungi; unidentified; unidentified; unidentified; unidentified; unidentified; fungal_endophyte
New.ReferenceOTU2502	1	0	0	0	0	0	0	0	1	0	0	0	0	0	0	0	0	0	0	0	0	0	0	0	0	0	0	0	0	0	Fungi; unidentified; unidentified; unidentified; unidentified; unidentified; fungal_endophyte
New.ReferenceOTU2819	0	0	0	0	0	0	0	0	0	0	0	0	0	0	0	0	0	0	1	0	0	0	0	0	0	0	0	0	0	0	Fungi; unidentified; unidentified; unidentified; unidentified; unidentified; fungal_endophyte
New.ReferenceOTU3016	0	0	0	0	0	0	0	0	0	2	0	0	0	0	0	0	0	0	0	0	0	0	0	0	0	0	0	0	0	0	Fungi; unidentified; unidentified; unidentified; unidentified; unidentified; fungal_endophyte
New.ReferenceOTU3047	0	0	0	0	0	3	1	0	0	1	0	1	0	0	0	0	1	0	0	0	0	0	0	0	0	0	0	0	0	0	Fungi; unidentified; unidentified; unidentified; unidentified; unidentified; fungal_endophyte
New.ReferenceOTU3232	0	0	0	0	0	0	0	0	0	0	0	0	0	0	0	0	0	0	1	0	0	0	0	0	0	0	0	0	0	0	Fungi; unidentified; unidentified; unidentified; unidentified; unidentified; fungal_endophyte
New.ReferenceOTU3480	0	1	0	0	3	0	0	0	0	1	0	0	0	1	1	0	0	0	0	0	0	0	0	0	0	0	0	0	1	0	Fungi; unidentified; unidentified; unidentified; unidentified; unidentified; fungal_endophyte
New.ReferenceOTU4219	0	0	0	0	1	0	0	0	0	0	0	0	0	0	0	0	0	0	0	0	0	0	0	0	0	0	0	0	0	0	Fungi; unidentified; unidentified; unidentified; unidentified; unidentified; fungal_endophyte
New.ReferenceOTU462	0	0	0	0	0	0	0	0	0	0	0	0	0	0	0	0	0	0	0	0	0	0	0	0	0	0	0	7	0	0	Fungi; unidentified; unidentified; unidentified; unidentified; unidentified; fungal_endophyte
New.ReferenceOTU4934	0	0	0	0	0	0	0	0	0	0	0	0	0	0	0	0	0	0	0	0	0	0	0	0	1	0	0	0	0	0	Fungi; unidentified; unidentified; unidentified; unidentified; unidentified; fungal_endophyte
New.ReferenceOTU4939	0	0	0	0	0	0	0	0	0	0	0	0	0	0	1	0	0	0	0	0	0	0	0	0	0	0	0	0	0	0	Fungi; unidentified; unidentified; unidentified; unidentified; unidentified; fungal_endophyte
New.ReferenceOTU5202	0	0	0	0	2	0	0	0	0	0	0	0	0	0	0	0	0	0	0	0	0	0	0	0	0	0	0	0	0	0	Fungi; unidentified; unidentified; unidentified; unidentified; unidentified; fungal_endophyte
New.ReferenceOTU5259	0	0	0	0	0	0	1	0	0	0	0	0	0	0	0	0	0	0	0	0	0	0	0	0	0	0	0	0	0	0	Fungi; unidentified; unidentified; unidentified; unidentified; unidentified; fungal_endophyte
New.ReferenceOTU5577	0	0	0	0	0	0	0	5	0	0	0	0	0	0	0	0	0	0	0	0	0	0	0	0	0	0	0	0	0	0	Fungi; unidentified; unidentified; unidentified; unidentified; unidentified; fungal_endophyte
New.ReferenceOTU5891	0	0	0	0	1	0	3	0	0	0	0	0	0	0	0	0	1	0	0	0	0	0	1	0	0	0	0	0	0	1	Fungi; unidentified; unidentified; unidentified; unidentified; unidentified; fungal_endophyte
New.ReferenceOTU6243	0	0	0	0	0	0	0	0	0	0	0	0	0	0	0	0	0	0	0	0	0	0	0	0	0	0	0	0	2	0	Fungi; unidentified; unidentified; unidentified; unidentified; unidentified; fungal_endophyte
New.ReferenceOTU6259	0	7	0	0	0	0	0	0	0	0	0	0	0	0	0	0	0	0	0	0	0	0	0	0	0	0	0	0	1	0	Fungi; unidentified; unidentified; unidentified; unidentified; unidentified; fungal_endophyte
New.ReferenceOTU6339	0	0	0	0	0	0	0	0	0	0	0	0	0	0	0	0	0	0	0	0	0	1	0	0	0	0	0	0	0	0	Fungi; unidentified; unidentified; unidentified; unidentified; unidentified; fungal_endophyte
New.ReferenceOTU7029	0	0	0	0	0	0	0	0	0	0	0	0	0	0	0	0	1	0	0	0	0	0	0	0	0	0	0	0	0	0	Fungi; unidentified; unidentified; unidentified; unidentified; unidentified; fungal_endophyte
New.ReferenceOTU7123	0	1	0	0	0	3	0	6	0	0	0	0	0	0	0	1	2	0	0	0	0	2	0	6	3	5	0	0	0	0	Fungi; unidentified; unidentified; unidentified; unidentified; unidentified; fungal_endophyte
New.ReferenceOTU7318	0	0	0	0	0	0	0	0	0	0	0	0	0	0	0	0	0	0	0	0	0	0	0	1	0	0	0	0	0	0	Fungi; unidentified; unidentified; unidentified; unidentified; unidentified; fungal_endophyte
New.ReferenceOTU7599	0	0	0	0	1	0	0	0	0	0	0	0	0	0	0	0	0	0	0	0	0	0	0	0	0	0	0	0	0	0	Fungi; unidentified; unidentified; unidentified; unidentified; unidentified; fungal_endophyte
New.ReferenceOTU792	0	0	0	0	0	0	0	0	1	0	0	0	0	0	0	0	0	0	0	0	0	0	0	0	0	0	0	0	0	0	Fungi; unidentified; unidentified; unidentified; unidentified; unidentified; fungal_endophyte
SH004060.06FU_KF436172_reps_singleton	0	0	0	0	1	0	0	0	0	0	0	0	0	0	0	0	0	0	0	0	0	0	0	0	0	0	0	0	0	0	Fungi; unidentified; unidentified; unidentified; unidentified; unidentified; fungal_endophyte
SH016056.06FU_EU686982_reps_singleton	0	0	0	0	0	0	0	0	0	0	0	0	0	0	0	0	0	0	0	0	0	0	0	0	0	0	0	0	1	0	Fungi; unidentified; unidentified; unidentified; unidentified; unidentified; fungal_endophyte
SH018119.06FU_KF435590_reps_singleton	0	0	0	0	3	0	0	0	0	0	0	0	1	2	0	1	0	0	0	0	0	0	0	0	0	0	0	0	0	0	Fungi; unidentified; unidentified; unidentified; unidentified; unidentified; fungal_endophyte
SH024810.06FU_JX156018_reps_singleton	0	0	0	0	0	0	2	0	0	0	0	0	0	0	0	0	0	0	0	0	0	0	0	0	0	0	0	0	0	0	Fungi; unidentified; unidentified; unidentified; unidentified; unidentified; fungal_endophyte
SH025654.06FU_JX156066_reps_singleton	0	0	0	0	0	0	0	0	0	0	0	0	0	0	3	0	0	0	0	2	2	0	0	0	0	0	0	0	0	0	Fungi; unidentified; unidentified; unidentified; unidentified; unidentified; fungal_endophyte
SH027113.06FU_KF436282_reps_singleton	0	0	0	0	0	0	0	0	0	0	0	0	0	0	0	0	0	0	0	2	0	0	0	0	0	0	0	0	0	0	Fungi; unidentified; unidentified; unidentified; unidentified; unidentified; fungal_endophyte
SH027117.06FU_DQ322095_reps_singleton	0	0	0	0	0	0	0	0	0	0	0	0	0	0	0	0	0	0	0	0	0	0	0	0	0	0	0	1	0	0	Fungi; unidentified; unidentified; unidentified; unidentified; unidentified; fungal_endophyte
SH027249.06FU_JX156033_reps_singleton	0	0	0	0	0	0	0	0	0	0	0	0	0	1	0	0	0	0	0	0	0	0	0	1	0	0	0	0	0	0	Fungi; unidentified; unidentified; unidentified; unidentified; unidentified; fungal_endophyte
SH127994.06FU_KC806297_reps	1	0	0	0	0	0	0	0	0	0	0	0	0	0	1	0	0	0	0	0	0	0	2	0	0	0	1	0	0	1	Fungi; unidentified; unidentified; unidentified; unidentified; unidentified; fungal_endophyte
SH145494.06FU_GU367897_reps	0	0	0	0	2	0	0	0	0	0	0	0	0	0	0	0	0	0	0	0	0	0	0	0	0	0	0	0	0	0	Fungi; unidentified; unidentified; unidentified; unidentified; unidentified; fungal_endophyte
New.ReferenceOTU3787	0	0	1	0	0	0	0	0	0	0	0	0	0	0	0	0	0	0	0	0	0	0	0	0	0	0	0	0	0	0	Fungi; unidentified; unidentified; unidentified; unidentified; unidentified; fungal_endophyte_sp_bn17
New.ReferenceOTU1753	0	0	0	0	0	0	0	0	0	1	0	0	0	0	0	0	0	0	0	0	0	0	0	0	0	0	0	0	0	0	Fungi; unidentified; unidentified; unidentified; unidentified; unidentified; fungal_endophyte_sp_ECD_2008
New.ReferenceOTU2217	0	0	0	0	0	0	0	0	0	0	0	0	0	0	0	0	0	0	1	0	0	0	0	0	0	0	0	0	0	0	Fungi; unidentified; unidentified; unidentified; unidentified; unidentified; fungal_endophyte_sp_ECD_2008
New.ReferenceOTU2504	0	0	0	0	0	0	0	0	0	0	0	0	0	0	0	0	0	0	0	2	0	0	0	0	0	0	0	0	0	0	Fungi; unidentified; unidentified; unidentified; unidentified; unidentified; fungal_endophyte_sp_ECD_2008
New.ReferenceOTU3421	2	0	0	2	0	0	2	1	0	0	0	0	0	1	3	0	1	4	4	0	0	6	2	6	0	0	0	6	12	0	Fungi; unidentified; unidentified; unidentified; unidentified; unidentified; fungal_endophyte_sp_ECD_2008
New.ReferenceOTU3512	0	3	0	0	0	0	0	0	3	0	0	0	0	0	0	0	0	0	0	2	0	0	0	1	0	0	0	0	0	0	Fungi; unidentified; unidentified; unidentified; unidentified; unidentified; fungal_endophyte_sp_ECD_2008
New.ReferenceOTU4274	0	0	0	0	0	0	0	0	0	0	1	0	0	0	0	0	0	0	0	0	0	0	0	0	0	0	0	0	0	0	Fungi; unidentified; unidentified; unidentified; unidentified; unidentified; fungal_endophyte_sp_ECD_2008
New.ReferenceOTU4887	0	0	0	0	0	0	0	0	0	0	0	0	0	0	0	0	0	0	0	2	0	0	0	0	0	0	0	0	0	0	Fungi; unidentified; unidentified; unidentified; unidentified; unidentified; fungal_endophyte_sp_ECD_2008
New.ReferenceOTU7752	0	2	0	0	0	0	0	0	0	0	0	0	0	0	0	0	0	0	0	0	0	0	0	0	0	0	0	0	0	0	Fungi; unidentified; unidentified; unidentified; unidentified; unidentified; fungal_endophyte_sp_ECD_2008
New.ReferenceOTU8006	1	0	0	0	0	0	0	0	0	0	0	0	0	0	0	0	0	0	0	0	0	0	0	0	0	0	0	0	0	0	Fungi; unidentified; unidentified; unidentified; unidentified; unidentified; fungal_endophyte_sp_ECD_2008
New.ReferenceOTU3108	0	0	0	0	0	1	0	0	0	0	0	0	0	0	0	0	1	0	0	0	0	0	1	1	0	0	0	0	0	0	Fungi; unidentified; unidentified; unidentified; unidentified; unidentified; fungal_endophyte_sp_ICMP_16019
New.ReferenceOTU3330	0	0	0	0	0	1	0	0	1	1	0	0	0	0	0	1	1	0	0	0	0	0	2	0	0	0	0	0	0	0	Fungi; unidentified; unidentified; unidentified; unidentified; unidentified; fungal_endophyte_sp_ICMP_16019
New.ReferenceOTU4089	1	0	0	0	0	3	0	2	0	1	1	3	0	0	0	0	0	0	0	0	0	0	2	0	0	0	0	0	0	0	Fungi; unidentified; unidentified; unidentified; unidentified; unidentified; fungal_endophyte_sp_ICMP_16019
New.ReferenceOTU4382	0	0	0	0	0	0	0	0	0	0	0	1	0	0	0	0	0	0	0	0	0	0	0	0	0	0	0	0	0	0	Fungi; unidentified; unidentified; unidentified; unidentified; unidentified; fungal_endophyte_sp_ICMP_16019
New.ReferenceOTU5054	0	0	0	2	0	0	0	0	0	0	0	0	0	0	0	0	0	0	0	0	0	0	0	0	0	0	0	0	0	0	Fungi; unidentified; unidentified; unidentified; unidentified; unidentified; fungal_endophyte_sp_ICMP_16019
SH112685.06FU_JQ760936_reps	0	0	1	0	1	0	0	2	1	0	1	3	0	0	0	1	0	0	0	0	0	0	2	0	0	0	0	0	1	0	Fungi; unidentified; unidentified; unidentified; unidentified; unidentified; fungal_endophyte_sp_ICMP_16019
New.ReferenceOTU1039	0	0	0	0	0	0	0	0	0	0	0	0	0	0	0	0	0	0	0	0	0	0	0	0	0	0	1	0	0	0	Fungi; unidentified; unidentified; unidentified; unidentified; unidentified; fungal_endophyte_sp_P1223A
New.ReferenceOTU3689	0	0	0	0	0	0	0	0	0	0	0	0	0	0	1	0	0	0	0	0	0	0	0	0	0	0	0	0	0	0	Fungi; unidentified; unidentified; unidentified; unidentified; unidentified; fungal_endophyte_sp_P1403G
New.ReferenceOTU3926	0	0	0	0	0	0	0	0	0	0	0	0	0	0	0	0	0	0	0	0	0	0	0	0	0	0	0	0	2	0	Fungi; unidentified; unidentified; unidentified; unidentified; unidentified; fungal_endophyte_sp_P1403G
New.ReferenceOTU1068	0	0	0	0	0	0	0	0	0	0	0	0	0	0	0	0	0	0	0	0	1	0	0	0	0	0	0	0	0	0	Fungi; unidentified; unidentified; unidentified; unidentified; unidentified; fungal_endophyte_sp_P605F
New.ReferenceOTU407	0	0	0	0	0	0	0	0	0	0	1	0	0	0	0	0	0	0	0	0	0	0	0	0	0	0	0	0	0	0	Fungi; unidentified; unidentified; unidentified; unidentified; unidentified; fungal_endophyte_sp_P605F
New.ReferenceOTU525	0	0	0	0	0	0	0	0	0	0	0	0	0	0	0	0	1	0	0	0	0	0	0	0	0	0	0	0	0	0	Fungi; unidentified; unidentified; unidentified; unidentified; unidentified; fungal_endophyte_sp_SS_1519
New.ReferenceOTU6327	0	2	0	0	0	0	0	0	0	0	0	0	0	0	0	0	0	0	0	1	0	0	0	0	0	0	0	0	0	0	Fungi; unidentified; unidentified; unidentified; unidentified; unidentified; fungal_endophyte_sp_SS_1519
New.ReferenceOTU3527	1	0	0	0	0	0	0	0	0	0	0	0	0	0	0	0	0	0	0	0	0	0	0	0	0	0	0	0	0	0	Fungi; unidentified; unidentified; unidentified; unidentified; unidentified; fungal_sp_1_MV_2011
New.ReferenceOTU7343	1	0	0	0	0	0	0	0	0	0	0	0	0	0	0	0	0	0	0	0	0	0	0	0	0	0	0	0	0	0	Fungi; unidentified; unidentified; unidentified; unidentified; unidentified; fungal_sp_1_MV_2011
New.ReferenceOTU8025	0	0	1	0	0	0	0	0	0	6	6	0	0	0	0	0	0	0	0	0	0	0	0	0	0	0	0	0	1	0	Fungi; unidentified; unidentified; unidentified; unidentified; unidentified; fungal_sp_2_MLSC_SO5
New.ReferenceOTU2652	0	0	0	0	0	0	0	0	0	0	0	0	0	0	0	0	0	0	0	0	0	0	0	0	0	0	0	0	0	1	Fungi; unidentified; unidentified; unidentified; unidentified; unidentified; fungal_sp_7_RU025
New.ReferenceOTU7613	0	0	0	0	0	0	0	0	0	0	0	0	0	0	0	0	0	0	0	0	0	1	0	0	0	0	0	0	0	0	Fungi; unidentified; unidentified; unidentified; unidentified; unidentified; fungal_sp_7_RU025
New.ReferenceOTU1882	0	0	0	0	1	0	0	0	0	0	0	0	0	0	0	0	0	0	0	0	0	0	0	0	0	0	0	0	0	0	Fungi; unidentified; unidentified; unidentified; unidentified; unidentified; fungal_sp_agrD417
New.ReferenceOTU6443	0	0	0	0	0	0	0	0	0	0	0	0	0	0	0	0	0	0	1	0	0	0	0	0	0	0	0	0	0	0	Fungi; unidentified; unidentified; unidentified; unidentified; unidentified; fungal_sp_AM2013
New.ReferenceOTU2751	0	0	0	1	0	0	0	0	0	0	0	0	0	0	0	0	0	0	0	0	0	0	0	0	0	0	0	0	0	0	Fungi; unidentified; unidentified; unidentified; unidentified; unidentified; fungal_sp_ARIZ_AZ0934
New.ReferenceOTU1900	0	0	0	0	0	2	2	1	0	0	0	0	0	0	0	1	0	0	0	0	0	0	0	0	0	0	0	1	0	0	Fungi; unidentified; unidentified; unidentified; unidentified; unidentified; fungal_sp_ARIZ_L453
New.ReferenceOTU4422	0	0	0	0	1	0	0	0	0	0	0	0	0	0	0	0	0	0	0	0	0	0	0	0	0	0	0	0	0	0	Fungi; unidentified; unidentified; unidentified; unidentified; unidentified; fungal_sp_ARIZ_L90
New.ReferenceOTU4471	0	0	0	0	1	0	0	0	0	0	0	0	0	0	0	0	0	0	0	0	0	0	0	0	0	0	0	0	0	0	Fungi; unidentified; unidentified; unidentified; unidentified; unidentified; fungal_sp_ARIZ_L90
New.ReferenceOTU2767	0	0	0	0	0	0	0	2	0	0	0	1	1	0	0	0	0	0	0	0	0	0	0	0	0	0	0	0	0	0	Fungi; unidentified; unidentified; unidentified; unidentified; unidentified; fungal_sp_ASR_138
New.ReferenceOTU3335	3	0	0	0	0	0	0	4	0	0	0	0	6	0	0	0	0	0	0	0	0	0	0	0	0	0	0	0	0	0	Fungi; unidentified; unidentified; unidentified; unidentified; unidentified; fungal_sp_ASR_138
New.ReferenceOTU4608	0	0	0	0	0	0	0	0	0	0	0	0	0	0	0	0	0	0	0	0	0	0	0	1	0	0	0	0	0	0	Fungi; unidentified; unidentified; unidentified; unidentified; unidentified; fungal_sp_ASR_138
New.ReferenceOTU5462	0	0	0	0	0	0	1	2	0	1	0	1	0	0	0	0	0	0	0	0	0	0	0	0	0	0	0	0	0	0	Fungi; unidentified; unidentified; unidentified; unidentified; unidentified; fungal_sp_ASR_138
New.ReferenceOTU6013	2	0	7	4	0	0	1	9	0	2	0	2	0	0	0	0	2	0	0	0	0	0	3	5	0	0	1	0	0	2	Fungi; unidentified; unidentified; unidentified; unidentified; unidentified; fungal_sp_ASR_138
New.ReferenceOTU8082	0	0	0	0	0	0	0	0	0	0	0	2	1	0	0	0	0	0	0	0	0	0	0	0	0	0	0	0	0	0	Fungi; unidentified; unidentified; unidentified; unidentified; unidentified; fungal_sp_ASR_138
New.ReferenceOTU4466	0	0	0	1	0	1	0	0	0	0	0	0	0	0	0	0	0	0	0	2	0	0	0	0	0	0	0	0	0	0	Fungi; unidentified; unidentified; unidentified; unidentified; unidentified; fungal_sp_ASR_33
New.ReferenceOTU4655	0	0	0	0	0	0	0	0	0	0	2	0	0	0	0	0	0	0	0	0	0	0	0	0	0	0	0	0	0	0	Fungi; unidentified; unidentified; unidentified; unidentified; unidentified; fungal_sp_ASR_33
New.ReferenceOTU7746	0	0	0	0	0	1	4	0	2	0	0	1	1	0	0	0	0	1	0	1	0	0	0	0	0	0	0	0	0	1	Fungi; unidentified; unidentified; unidentified; unidentified; unidentified; fungal_sp_ASR_33
New.ReferenceOTU8039	0	0	0	0	0	0	0	0	0	0	0	0	0	0	0	0	0	0	0	0	0	0	0	0	0	0	0	0	1	0	Fungi; unidentified; unidentified; unidentified; unidentified; unidentified; fungal_sp_aurim729
SH028992.06FU_HE585020_reps_singleton	0	0	0	0	0	0	0	0	0	0	0	0	0	0	0	0	0	0	0	0	0	0	1	0	0	0	0	0	0	0	Fungi; unidentified; unidentified; unidentified; unidentified; unidentified; fungal_sp_AX128
New.ReferenceOTU5574	0	0	0	0	0	0	0	0	0	0	0	0	0	0	0	0	0	0	0	0	0	0	0	0	0	0	0	0	7	0	Fungi; unidentified; unidentified; unidentified; unidentified; unidentified; fungal_sp_F070
SH028921.06FU_JQ747678_reps_singleton	1	0	0	0	0	0	0	0	0	0	0	1	0	0	0	0	0	0	0	0	0	0	0	0	0	0	0	0	0	0	Fungi; unidentified; unidentified; unidentified; unidentified; unidentified; fungal_sp_F190
New.ReferenceOTU3367	0	0	0	0	0	0	0	0	0	0	0	0	0	0	0	0	0	0	0	0	0	0	1	0	0	0	0	0	0	0	Fungi; unidentified; unidentified; unidentified; unidentified; unidentified; fungal_sp_F195
New.ReferenceOTU5428	0	0	0	0	0	0	0	0	0	0	0	0	0	0	0	0	0	0	0	1	0	0	0	0	0	0	0	0	0	0	Fungi; unidentified; unidentified; unidentified; unidentified; unidentified; fungal_sp_F195
New.ReferenceOTU6399	0	0	0	0	0	0	0	0	0	0	0	0	0	0	0	0	0	0	0	0	0	0	0	0	2	0	1	2	0	1	Fungi; unidentified; unidentified; unidentified; unidentified; unidentified; fungal_sp_F195
New.ReferenceOTU1754	0	0	0	0	0	0	0	0	0	0	0	0	1	0	0	0	0	0	0	0	0	0	0	0	0	0	0	0	0	0	Fungi; unidentified; unidentified; unidentified; unidentified; unidentified; fungal_sp_F253
New.ReferenceOTU4747	0	0	0	0	0	0	0	1	0	0	0	0	0	0	1	0	0	0	0	0	1	1	0	0	1	0	0	0	0	1	Fungi; unidentified; unidentified; unidentified; unidentified; unidentified; fungal_sp_F253
New.ReferenceOTU6864	0	0	0	0	0	0	0	0	0	0	0	0	0	0	1	0	0	0	0	0	0	0	0	0	0	0	1	0	0	0	Fungi; unidentified; unidentified; unidentified; unidentified; unidentified; fungal_sp_F253
New.ReferenceOTU14	0	0	0	0	0	0	0	0	0	0	3	0	0	0	0	0	0	0	0	0	0	0	0	0	0	0	0	0	0	0	Fungi; unidentified; unidentified; unidentified; unidentified; unidentified; fungal_sp_F261
New.ReferenceOTU8050	0	0	0	0	0	0	0	0	0	0	0	0	0	0	0	1	0	0	0	0	0	0	0	0	0	0	0	0	0	0	Fungi; unidentified; unidentified; unidentified; unidentified; unidentified; fungal_sp_F261
New.ReferenceOTU4743	0	0	0	0	0	0	0	0	0	0	0	0	0	0	0	0	0	0	0	0	0	0	0	2	0	0	0	0	0	0	Fungi; unidentified; unidentified; unidentified; unidentified; unidentified; fungal_sp_F355
New.ReferenceOTU5900	0	0	0	0	0	0	0	0	0	0	0	0	0	0	0	0	0	0	1	0	0	0	0	0	0	0	0	0	0	0	Fungi; unidentified; unidentified; unidentified; unidentified; unidentified; fungal_sp_F355
New.ReferenceOTU1702	0	0	0	0	0	0	0	0	0	0	0	0	0	0	0	0	0	0	0	0	0	0	0	0	0	0	0	1	0	0	Fungi; unidentified; unidentified; unidentified; unidentified; unidentified; fungal_sp_F402
New.ReferenceOTU1579	0	0	0	0	0	0	0	0	0	0	0	0	0	0	0	0	0	0	0	0	0	2	0	0	3	0	0	0	0	0	Fungi; unidentified; unidentified; unidentified; unidentified; unidentified; fungal_sp_F498
New.ReferenceOTU3949	0	0	0	0	0	2	0	0	0	0	0	0	0	0	0	0	0	0	0	0	4	0	0	0	0	0	0	0	0	0	Fungi; unidentified; unidentified; unidentified; unidentified; unidentified; fungal_sp_F546
New.ReferenceOTU1593	0	0	0	0	0	0	0	0	0	0	0	0	0	0	0	0	0	0	0	0	0	0	0	2	0	0	0	0	0	0	Fungi; unidentified; unidentified; unidentified; unidentified; unidentified; fungal_sp_FF_2011
New.ReferenceOTU5801	0	0	0	0	0	0	1	0	0	0	0	0	0	0	0	0	0	0	0	0	0	0	0	0	0	0	0	0	0	0	Fungi; unidentified; unidentified; unidentified; unidentified; unidentified; fungal_sp_FF_2011
SH126629.06FU_FJ025245_reps	0	0	0	0	1	0	0	0	0	0	0	0	0	0	0	0	0	0	0	0	0	0	0	0	0	0	0	0	0	1	Fungi; unidentified; unidentified; unidentified; unidentified; unidentified; fungal_sp_FF_2011
New.ReferenceOTU538	2	0	0	0	0	0	1	0	0	0	0	2	0	0	2	0	0	0	0	0	1	1	0	0	2	0	5	0	0	0	Fungi; unidentified; unidentified; unidentified; unidentified; unidentified; fungal_sp_JF44

SH006419.06FU_JX155941_reps_singleton	0	0	0	0	0	0	0	0	0	0	0	0	0	0	0	0	0	0	0	0	1	0	0	0	0	0	0	0	0	0	Fungi; unidentified; unidentified; unidentified; unidentified; unidentified; fungal_sp_JF44
SH095977.06FU_EF488146_reps	2	2	2	0	1	0	2	2	1	0	0	9	2	2	0	1	1	0	0	0	5	9	4	0	19	1	10	8	0	3	Fungi; unidentified; unidentified; unidentified; unidentified; unidentified; fungal_sp_JF44
SH095982.06FU_KC702782_reps	0	0	1	0	0	0	0	0	0	0	0	1	0	0	0	0	0	0	1	0	1	0	1	0	2	1	0	0	0	0	Fungi; unidentified; unidentified; unidentified; unidentified; unidentified; fungal_sp_JF44
SH124975.06FU_DQ677655_refs	0	1	0	0	0	1	0	0	0	0	0	1	0	0	0	0	0	0	0	0	0	1	0	0	1	0	1	0	0	0	Fungi; unidentified; unidentified; unidentified; unidentified; unidentified; fungal_sp_JF44
SH124985.06FU_DQ313156_reps	0	0	0	0	0	0	0	0	0	0	0	0	0	0	0	0	0	0	0	0	0	0	0	0	0	0	2	0	0	0	Fungi; unidentified; unidentified; unidentified; unidentified; unidentified; fungal_sp_JF44
New.ReferenceOTU3088	0	0	0	0	0	0	0	0	0	0	0	0	0	0	0	0	0	0	0	0	0	0	0	0	3	0	0	0	0	0	Fungi; unidentified; unidentified; unidentified; unidentified; unidentified; fungal_sp_KRP24
New.ReferenceOTU2936	0	0	0	0	1	0	0	0	0	0	0	0	0	0	0	0	0	0	0	0	0	0	0	0	0	0	0	0	0	0	Fungi; unidentified; unidentified; unidentified; unidentified; unidentified; fungal_sp_M18
New.ReferenceOTU3533	0	0	0	0	1	0	0	0	0	0	0	4	0	0	0	0	0	0	0	0	0	0	0	0	0	0	0	0	0	0	Fungi; unidentified; unidentified; unidentified; unidentified; unidentified; fungal_sp_M18
New.ReferenceOTU7061	0	0	0	0	0	0	0	0	0	0	0	0	0	1	0	0	0	0	0	0	0	0	0	0	0	0	0	0	0	0	Fungi; unidentified; unidentified; unidentified; unidentified; unidentified; fungal_sp_M18
New.ReferenceOTU3222	0	0	0	0	0	0	0	0	0	0	0	0	0	0	0	0	0	0	0	0	0	0	0	0	0	0	0	0	1	0	Fungi; unidentified; unidentified; unidentified; unidentified; unidentified; fungal_sp_T258
New.ReferenceOTU3469	0	0	0	1	0	0	0	0	0	0	0	0	0	0	0	0	0	0	0	0	0	0	0	0	0	0	0	0	1	0	Fungi; unidentified; unidentified; unidentified; unidentified; unidentified; fungal_sp_T258
New.ReferenceOTU7632	0	0	0	0	0	0	0	0	0	0	0	0	0	0	0	0	0	0	0	0	0	0	0	0	0	0	0	0	1	0	Fungi; unidentified; unidentified; unidentified; unidentified; unidentified; fungal_sp_T258
New.ReferenceOTU4591	0	0	1	0	0	0	0	0	0	0	0	0	0	0	0	0	0	0	0	0	0	0	0	0	0	0	0	0	0	0	Fungi; unidentified; unidentified; unidentified; unidentified; unidentified; fungal_sp_TRN441
New.ReferenceOTU205	0	0	0	0	0	0	0	0	0	0	0	0	0	0	0	0	0	0	0	0	0	0	0	0	0	0	0	0	0	5	Fungi; unidentified; unidentified; unidentified; unidentified; unidentified; fungal_sp_W2_2
SH027502.06FU_HQ889706_reps_singleton	0	1	0	0	0	0	0	0	0	0	0	0	0	0	0	0	0	0	0	0	0	0	0	1	0	0	0	0	0	0	Fungi; unidentified; unidentified; unidentified; unidentified; unidentified; fungal_sp_W2_2
New.CleanUp.ReferenceOTU1761	0	0	0	0	0	0	0	0	1	0	0	0	0	0	0	0	0	0	0	0	0	0	0	0	0	0	0	0	0	0	Fungi; unidentified; unidentified; unidentified; unidentified; unidentified; Fungi_sp
New.CleanUp.ReferenceOTU254	0	0	0	0	0	0	0	0	0	0	0	0	0	0	0	0	0	0	1	0	0	0	0	0	0	0	0	0	0	0	Fungi; unidentified; unidentified; unidentified; unidentified; unidentified; Fungi_sp
New.CleanUp.ReferenceOTU365	0	0	0	0	0	0	0	0	0	0	0	0	0	0	0	0	0	0	0	0	0	0	0	0	0	0	0	0	1	0	Fungi; unidentified; unidentified; unidentified; unidentified; unidentified; Fungi_sp
New.CleanUp.ReferenceOTU617	0	0	0	1	0	0	0	0	0	0	0	0	0	0	0	0	0	0	0	0	0	0	0	0	0	0	0	0	0	0	Fungi; unidentified; unidentified; unidentified; unidentified; unidentified; Fungi_sp
New.CleanUp.ReferenceOTU64	1	0	0	0	0	0	0	0	0	0	0	0	0	0	0	0	0	0	0	0	0	0	0	0	0	0	0	0	0	0	Fungi; unidentified; unidentified; unidentified; unidentified; unidentified; Fungi_sp
New.CleanUp.ReferenceOTU804	0	0	0	0	0	0	0	0	0	0	0	0	0	0	0	0	0	0	1	0	0	0	0	0	0	0	0	0	0	0	Fungi; unidentified; unidentified; unidentified; unidentified; unidentified; Fungi_sp
New.CleanUp.ReferenceOTU969	0	0	0	0	0	0	0	0	0	0	0	0	0	0	0	0	0	0	0	0	1	0	0	0	0	0	0	0	0	0	Fungi; unidentified; unidentified; unidentified; unidentified; unidentified; Fungi_sp
New.ReferenceOTU1072	0	0	0	0	0	0	0	0	0	0	0	0	0	1	0	0	0	0	0	0	0	0	0	0	0	0	0	0	0	0	Fungi; unidentified; unidentified; unidentified; unidentified; unidentified; Fungi_sp
New.ReferenceOTU1081	0	0	0	0	1	0	0	0	0	0	0	0	0	0	0	0	0	0	0	0	0	0	0	0	0	0	0	0	0	0	Fungi; unidentified; unidentified; unidentified; unidentified; unidentified; Fungi_sp
New.ReferenceOTU1166	0	0	0	6	4	0	3	0	0	0	0	0	2	0	0	0	0	0	0	0	0	0	0	0	0	0	0	0	0	0	Fungi; unidentified; unidentified; unidentified; unidentified; unidentified; Fungi_sp
New.ReferenceOTU1184	0	0	0	0	0	0	0	0	0	0	0	0	0	0	0	0	0	0	0	0	0	0	1	0	0	0	0	0	0	0	Fungi; unidentified; unidentified; unidentified; unidentified; unidentified; Fungi_sp
New.ReferenceOTU1252	0	0	0	0	0	0	0	0	0	0	2	0	0	0	0	0	0	0	0	0	0	0	0	0	0	0	0	0	0	0	Fungi; unidentified; unidentified; unidentified; unidentified; unidentified; Fungi_sp
New.ReferenceOTU1301	0	0	0	0	0	0	0	0	0	0	0	0	0	0	0	0	0	0	0	0	0	0	0	0	0	0	0	0	2	0	Fungi; unidentified; unidentified; unidentified; unidentified; unidentified; Fungi_sp
New.ReferenceOTU1327	0	0	0	0	0	0	0	0	1	0	0	0	0	0	0	0	0	0	0	0	0	0	0	0	0	0	0	0	0	0	Fungi; unidentified; unidentified; unidentified; unidentified; unidentified; Fungi_sp
New.ReferenceOTU1355	0	0	0	0	5	0	0	0	0	0	0	0	0	2	0	0	0	0	0	0	0	0	0	1	0	0	0	0	0	0	Fungi; unidentified; unidentified; unidentified; unidentified; unidentified; Fungi_sp
New.ReferenceOTU1368	0	0	0	0	0	0	0	2	5	0	0	0	0	0	0	0	0	0	0	0	0	0	0	0	0	0	0	0	2	0	Fungi; unidentified; unidentified; unidentified; unidentified; unidentified; Fungi_sp
New.ReferenceOTU1400	0	0	1	0	0	0	0	0	0	1	0	0	0	0	0	0	0	0	0	1	0	0	0	0	0	0	0	0	0	0	Fungi; unidentified; unidentified; unidentified; unidentified; unidentified; Fungi_sp
New.ReferenceOTU1498	0	0	0	0	0	0	0	0	0	0	0	0	0	0	0	1	1	0	0	0	0	0	0	0	0	0	0	0	0	0	Fungi; unidentified; unidentified; unidentified; unidentified; unidentified; Fungi_sp
New.ReferenceOTU1613	0	0	0	0	0	0	0	0	0	0	0	0	0	0	0	0	1	0	0	1	0	0	0	0	0	0	0	0	0	0	Fungi; unidentified; unidentified; unidentified; unidentified; unidentified; Fungi_sp
New.ReferenceOTU1650	0	0	0	0	0	0	0	0	1	0	2	0	0	0	0	0	0	0	0	0	0	0	0	0	0	0	0	0	0	0	Fungi; unidentified; unidentified; unidentified; unidentified; unidentified; Fungi_sp
New.ReferenceOTU1762	0	0	0	0	2	0	1	0	0	1	0	0	5	0	0	1	0	0	0	0	0	0	0	2	0	0	0	0	0	0	Fungi; unidentified; unidentified; unidentified; unidentified; unidentified; Fungi_sp
New.ReferenceOTU178	0	0	0	0	0	0	0	0	0	0	0	0	0	0	0	0	0	0	0	2	0	0	0	0	0	0	0	0	0	0	Fungi; unidentified; unidentified; unidentified; unidentified; unidentified; Fungi_sp
New.ReferenceOTU1781	0	0	0	0	0	0	0	0	0	0	0	0	0	0	0	0	1	0	0	0	0	0	0	0	0	0	0	0	0	0	Fungi; unidentified; unidentified; unidentified; unidentified; unidentified; Fungi_sp
New.ReferenceOTU1786	0	0	0	0	0	0	0	0	0	2	0	0	0	1	1	0	0	0	0	0	0	0	0	0	0	0	0	1	0	0	Fungi; unidentified; unidentified; unidentified; unidentified; unidentified; Fungi_sp
New.ReferenceOTU1832	0	0	0	0	0	0	0	0	0	0	0	0	0	0	0	0	0	0	1	0	0	0	0	0	0	0	0	0	0	0	Fungi; unidentified; unidentified; unidentified; unidentified; unidentified; Fungi_sp
New.ReferenceOTU1848	0	0	0	4	4	0	0	0	0	0	0	1	5	0	3	0	0	0	0	0	0	0	0	0	0	0	0	0	0	0	Fungi; unidentified; unidentified; unidentified; unidentified; unidentified; Fungi_sp
New.ReferenceOTU1849	0	0	0	0	0	0	0	0	1	0	0	0	0	0	0	0	0	0	0	0	0	0	0	0	0	0	0	0	0	0	Fungi; unidentified; unidentified; unidentified; unidentified; unidentified; Fungi_sp
New.ReferenceOTU1971	0	0	0	4	0	0	0	0	0	0	0	0	0	0	0	0	0	0	0	0	0	0	0	0	0	0	0	0	0	0	Fungi; unidentified; unidentified; unidentified; unidentified; unidentified; Fungi_sp
New.ReferenceOTU1973	0	0	0	0	0	0	0	0	2	0	0	0	0	0	0	0	0	0	0	0	0	0	0	0	0	0	0	0	0	0	Fungi; unidentified; unidentified; unidentified; unidentified; unidentified; Fungi_sp
New.ReferenceOTU206	0	1	0	0	0	0	0	0	4	1	0	0	0	0	0	0	0	0	0	0	0	0	0	0	0	5	0	1	7	0	Fungi; unidentified; unidentified; unidentified; unidentified; unidentified; Fungi_sp
New.ReferenceOTU2111	0	0	0	0	0	0	0	0	0	0	0	0	0	0	0	0	0	0	4	0	0	0	0	0	0	0	0	0	0	0	Fungi; unidentified; unidentified; unidentified; unidentified; unidentified; Fungi_sp
New.ReferenceOTU212	0	5	0	1	0	0	0	0	0	0	0	0	0	0	0	0	0	0	0	0	0	0	0	0	0	0	0	0	0	0	Fungi; unidentified; unidentified; unidentified; unidentified; unidentified; Fungi_sp
New.ReferenceOTU2191	0	0	0	0	0	0	0	0	0	0	0	0	0	0	0	2	0	0	0	0	0	0	0	0	0	0	0	0	0	0	Fungi; unidentified; unidentified; unidentified; unidentified; unidentified; Fungi_sp
New.ReferenceOTU2233	2	0	0	0	0	0	0	0	0	0	0	0	0	0	0	0	0	0	0	7	0	0	0	0	0	0	0	0	0	0	Fungi; unidentified; unidentified; unidentified; unidentified; unidentified; Fungi_sp
New.ReferenceOTU2363	0	0	0	0	0	0	0	0	0	0	0	0	0	0	0	0	0	0	5	1	0	0	0	0	0	0	0	0	0	0	Fungi; unidentified; unidentified; unidentified; unidentified; unidentified; Fungi_sp
New.ReferenceOTU2369	0	0	0	0	0	0	0	17	0	0	0	0	0	0	0	0	0	0	0	0	0	0	0	0	0	0	0	0	0	0	Fungi; unidentified; unidentified; unidentified; unidentified; unidentified; Fungi_sp
New.ReferenceOTU2370	0	0	0	0	0	0	0	0	0	0	0	0	0	0	0	0	2	0	0	0	0	0	1	0	0	0	0	0	0	0	Fungi; unidentified; unidentified; unidentified; unidentified; unidentified; Fungi_sp
New.ReferenceOTU2424	1	0	0	0	0	0	0	0	0	0	0	0	0	0	0	0	0	0	0	0	0	0	0	0	0	0	0	0	0	0	Fungi; unidentified; unidentified; unidentified; unidentified; unidentified; Fungi_sp
New.ReferenceOTU2444	0	0	0	0	0	0	0	0	0	0	0	0	0	0	0	0	0	0	1	0	0	0	0	0	0	0	0	0	0	0	Fungi; unidentified; unidentified; unidentified; unidentified; unidentified; Fungi_sp
New.ReferenceOTU2569	0	0	0	0	0	0	0	0	0	0	1	0	0	0	0	0	0	0	0	0	0	0	0	0	0	0	0	0	0	0	Fungi; unidentified; unidentified; unidentified; unidentified; unidentified; Fungi_sp
New.ReferenceOTU2582	0	0	0	0	0	0	0	0	0	0	0	0	0	0	0	0	0	0	1	0	0	0	0	0	0	0	0	0	0	0	Fungi; unidentified; unidentified; unidentified; unidentified; unidentified; Fungi_sp
New.ReferenceOTU2596	0	0	1	0	0	0	0	0	0	0	0	0	0	0	0	0	0	0	0	0	0	0	0	0	0	0	0	0	0	0	Fungi; unidentified; unidentified; unidentified; unidentified; unidentified; Fungi_sp
New.ReferenceOTU2684	0	2	0	0	0	0	0	0	0	0	0	0	0	0	0	0	0	0	1	0	0	0	0	0	0	0	0	0	0	0	Fungi; unidentified; unidentified; unidentified; unidentified; unidentified; Fungi_sp
New.ReferenceOTU27	0	0	0	0	0	0	0	4	0	0	0	0	0	0	0	1	0	0	0	0	0	0	0	0	0	0	0	0	0	0	Fungi; unidentified; unidentified; unidentified; unidentified; unidentified; Fungi_sp
New.ReferenceOTU2707	0	1	0	1	0	0	0	0	0	0	0	0	1	0	0	0	0	2	0	1	0	0	0	0	0	0	0	0	0	0	Fungi; unidentified; unidentified; unidentified; unidentified; unidentified; Fungi_sp
New.ReferenceOTU2724	0	0	0	0	0	0	0	0	0	0	0	0	0	0	0	0	0	2	13	0	0	0	0	0	0	0	0	0	0	0	Fungi; unidentified; unidentified; unidentified; unidentified; unidentified; Fungi_sp
New.ReferenceOTU2830	0	0	0	0	0	0	0	0	1	0	0	0	0	0	0	0	0	0	2	0	0	0	0	0	0	0	0	0	0	0	Fungi; unidentified; unidentified; unidentified; unidentified; unidentified; Fungi_sp
New.ReferenceOTU2887	1	0	0	0	0	0	0	0	0	0	0	0	0	0	0	0	0	0	0	0	0	0	0	0	0	0	0	0	0	0	Fungi; unidentified; unidentified; unidentified; unidentified; unidentified; Fungi_sp
New.ReferenceOTU2906	2	0	0	1	5	0	0	0	0	2	2	0	0	0	0	0	1	0	0	0	0	0	0	0	0	0	0	0	0	0	Fungi; unidentified; unidentified; unidentified; unidentified; unidentified; Fungi_sp
New.ReferenceOTU2939	0	0	0	0	0	0	0	0	0	0	0	0	0	0	1	0	0	0	0	0	0	0	0	0	0	0	0	0	0	0	Fungi; unidentified; unidentified; unidentified; unidentified; unidentified; Fungi_sp
New.ReferenceOTU2957	0	0	0	1	0	0	2	0	0	0	0	0	1	0	1	0	0	0	0	0	0	0	0	0	0	0	0	0	0	0	Fungi; unidentified; unidentified; unidentified; unidentified; unidentified; Fungi_sp
New.ReferenceOTU2967	0	0	0	0	0	0	0	0	0	0	1	0	0	0	0	0	0	0	0	0	0	0	0	5	0	1	0	0	0	0	Fungi; unidentified; unidentified; unidentified; unidentified; unidentified; Fungi_sp
New.ReferenceOTU3015	0	0	0	0	0	0	0	0	0	0	0	0	0	0	0	0	0	0	0	0	0	0	0	1	0	0	0	0	0	0	Fungi; unidentified; unidentified; unidentified; unidentified; unidentified; Fungi_sp
New.ReferenceOTU3044	0	0	0	0	0	0	0	0	0	0	0	0	0	0	0	0	0	0	0	0	0	1	0	0	0	0	0	0	0	0	Fungi; unidentified; unidentified; unidentified; unidentified; unidentified; Fungi_sp
New.ReferenceOTU3137	0	0	0	0	0	0	0	0	0	0	0	0	0	0	0	0	0	0	0	0	0	0	0	0	0	0	0	0	1	0	Fungi; unidentified; unidentified; unidentified; unidentified; unidentified; Fungi_sp
New.ReferenceOTU325	0	0	0	0	0	0	0	0	0	0	0	0	0	0	0	0	1	0	0	0	0	0	0	0	0	0	0	0	0	0	Fungi; unidentified; unidentified; unidentified; unidentified; unidentified; Fungi_sp
New.ReferenceOTU3266	0	0	0	0	0	0	0	0	0	0	0	0	0	0	0	0	0	0	11	0	0	0	0	0	0	0	0	0	0	0	Fungi; unidentified; unidentified; unidentified; unidentified; unidentified; Fungi_sp
New.ReferenceOTU3319	0	0	0	0	0	0	0	0	6	0	7	0	0	1	0	0	1	1	4	0	0	0	2	0	0	0	0	0	0	0	Fungi; unidentified; unidentified; unidentified; unidentified; unidentified; Fungi_sp
New.ReferenceOTU3363	0	0	0	0	1	0	0	0	0	0	0	0	0	0	0	0	0	0	0	0	0	0	0	0	0	0	0	0	0	0	Fungi; unidentified; unidentified; unidentified; unidentified; unidentified; Fungi_sp
New.ReferenceOTU3381	1	0	0	0	0	0	0	0	0	0	0	0	0	0	0	0	0	0	0	0	0	0	0	0	0	0	0	0	0	0	Fungi; unidentified; unidentified; unidentified; unidentified; unidentified; Fungi_sp
New.ReferenceOTU3418	0	0	0	0	4	0	0	0	0	1	0	0	0	0	0	0	0	0	0	0	0	0	0	0	0	0	0	0	0	0	Fungi; unidentified; unidentified; unidentified; unidentified; unidentified; Fungi_sp
New.ReferenceOTU3434	0	0	0	0	0	0	0	0	4	0	0	0	1	2	0	1	0	0	0	0	0	0	0	9	0	0	0	0	0	0	Fungi; unidentified; unidentified; unidentified; unidentified; unidentified; Fungi_sp
New.ReferenceOTU3459	0	0	0	1	0	0	0	0	0	0	0	0	0	0	0	0	0	0	0	0	0	0	0	0	0	0	0	0	0	0	Fungi; unidentified; unidentified; unidentified; unidentified; unidentified; Fungi_sp
New.ReferenceOTU349	0	0	0	0	0	0	0	2	0	0	0	0	0	0	0	0	0	0	0	0	0	0	0	0	0	0	0	0	0	0	Fungi; unidentified; unidentified; unidentified; unidentified; unidentified; Fungi_sp
New.ReferenceOTU3536	1	0	0	0	0	0	0	0	0	0	0	0	0	0	0	0	0	0	0	0	0	0	0	0	0	0	0	0	0	0	Fungi; unidentified; unidentified; unidentified; unidentified; unidentified; Fungi_sp
New.ReferenceOTU3567	0	0	0	0	0	0	0	0	1	1	1	0	0	0	0	0	0	2	8	1	0	0	0	0	0	0	0	1	0	0	Fungi; unidentified; unidentified; unidentified; unidentified; unidentified; Fungi_sp
New.ReferenceOTU3611	0	0	0	0	0	0	0	0	0	0	0	0	0	0	0	0	0	0	0	0	0	0	0	0	0	0	0	0	5	0	Fungi; unidentified; unidentified; unidentified; unidentified; unidentified; Fungi_sp
New.ReferenceOTU3634	0	0	0	0	0	0	0	0	0	0	0	2	0	0	0	0	0	0	0	0	0	0	0	0	0	0	0	0	0	0	Fungi; unidentified; unidentified; unidentified; unidentified; unidentified; Fungi_sp
New.ReferenceOTU3700	0	0	0	0	0	0	0	0	0	0	0	0	0	0	0	0	0	3	0	0	0	0	0	0	0	0	0	0	0	0	Fungi; unidentified; unidentified; unidentified; unidentified; unidentified; Fungi_sp
New.ReferenceOTU3707	0	0	0	0	0	0	0	0	0	0	1	0	0	0	0	0	0	0	0	0	0	0	0	0	0	0	0	0	0	0	Fungi; unidentified; unidentified; unidentified; unidentified; unidentified; Fungi_sp
New.ReferenceOTU3800	0	0	0	0	0	0	0	0	1	0	0	0	0	0	0	0	0	0	0	0	0	0	0	0	0	0	0	0	0	0	Fungi; unidentified; unidentified; unidentified; unidentified; unidentified; Fungi_sp
New.ReferenceOTU3812	0	0	0	0	0	0	0	0	0	0	0	0	0	0	0	0	0	0	0	12	0	0	0	0	0	0	0	0	0	0	Fungi; unidentified; unidentified; unidentified; unidentified; unidentified; Fungi_sp
New.ReferenceOTU3846	0	0	0	0	0	0	0	0	0	0	0	0	0	0	0	0	0	0	0	0	0	0	0	0	0	32	0	0	0	0	Fungi; unidentified; unidentified; unidentified; unidentified; unidentified; Fungi_sp
New.ReferenceOTU3863	0	0	0	0	0	0	0	0	1	0	0	0	0	0	0	0	0	0	0	0	0	0	0	0	0	0	0	0	0	0	Fungi; unidentified; unidentified; unidentified; unidentified; unidentified; Fungi_sp
New.ReferenceOTU3885	0	0	0	0	0	0	0	0	0	0	0	0	0	0	0	0	0	0	1	0	0	0	0	0	0	0	0	0	0	0	Fungi; unidentified; unidentified; unidentified; unidentified; unidentified; Fungi_sp
New.ReferenceOTU3900	0	0	0	0	0	0	0	0	0	0	0	0	0	0	0	0	0	0	0	1	0	0	0	0	0	0	0	0	0	0	Fungi; unidentified; unidentified; unidentified; unidentified; unidentified; Fungi_sp
New.ReferenceOTU396	0	0	0	0	0	0	0	0	0	0	0	0	0	0	0	0	0	0	0	2	0	0	0	0	0	0	0	0	0	0	Fungi; unidentified; unidentified; unidentified; unidentified; unidentified; Fungi_sp
New.ReferenceOTU4081	0	0	0	0	0	0	0	0	3	0	0	0	0	0	0	0	0	0	0	0	0	0	0	0	0	0	0	0	0	0	Fungi; unidentified; unidentified; unidentified; unidentified; unidentified; Fungi_sp
New.ReferenceOTU4159	1	0	1	0	0	0	0	0	0	0	0	0	0	0	1	0	0	0	6	1	0	0	1	0	0	0	0	0	0	0	Fungi; unidentified; unidentified; unidentified; unidentified; unidentified; Fungi_sp
New.ReferenceOTU4193	0	0	0	0	0	0	0	0	0	0	0	1	0	0	0	0	0	0	0	0	0	0	0	0	0	0	0	0	0	0	Fungi; unidentified; unidentified; unidentified; unidentified; unidentified; Fungi_sp
New.ReferenceOTU4402	0	0	0	0	0	0	0	0	0	0	0	0	0	1	0	0	0	0	0	0	0	0	1	0	0	0	0	0	0	0	Fungi; unidentified; unidentified; unidentified; unidentified; unidentified; Fungi_sp
New.ReferenceOTU4441	0	0	0	0	0	0	0	1	0	0	0	0	1	1	0	0	0	0	0	0	0	0	0	0	0	0	0	0	0	0	Fungi; unidentified; unidentified; unidentified; unidentified; unidentified; Fungi_sp
New.ReferenceOTU4453	0	0	0	0	0	0	0	0	0	0	1	0	0	0	0	0	1	0	0	0	0	0	0	0	0	0	0	0	0	0	Fungi; unidentified; unidentified; unidentified; unidentified; unidentified; Fungi_sp
New.ReferenceOTU4470	0	0	0	2	0	0	0	0	0	0	0	0	0	0	0	0	0	0	0	0	0	0	0	0	0	0	0	0	0	0	Fungi; unidentified; unidentified; unidentified; unidentified; unidentified; Fungi_sp
New.ReferenceOTU4567	0	0	0	0	0	0	0	0	0	0	0	0	0	0	0	0	0	7	0	0	0	0	0	0	0	0	0	0	0	0	Fungi; unidentified; unidentified; unidentified; unidentified; unidentified; Fungi_sp

New.ReferenceOTU4568	0	0	0	0	0	0	0	0	0	0	0	0	0	0	0	0	0	0	1	0	0	0	0	0	0	0	0	0	0	0	Fungi; unidentified; unidentified; unidentified; unidentified; unidentified; Fungi_sp
New.ReferenceOTU4573	0	0	0	0	0	0	0	0	0	0	0	0	0	0	0	0	0	0	0	0	0	0	0	0	0	0	0	0	4	0	Fungi; unidentified; unidentified; unidentified; unidentified; unidentified; Fungi_sp
New.ReferenceOTU4606	0	0	0	1	0	0	0	0	0	0	0	0	0	0	0	0	0	0	0	0	0	0	0	0	0	0	0	0	0	0	Fungi; unidentified; unidentified; unidentified; unidentified; unidentified; Fungi_sp
New.ReferenceOTU4607	0	0	0	0	0	0	0	1	0	0	0	0	0	0	0	0	0	0	0	0	0	0	0	0	0	0	0	0	0	0	Fungi; unidentified; unidentified; unidentified; unidentified; unidentified; Fungi_sp
New.ReferenceOTU4646	1	0	0	0	0	0	0	0	0	0	0	0	0	0	0	0	0	0	0	0	0	0	0	0	0	6	0	0	5	0	Fungi; unidentified; unidentified; unidentified; unidentified; unidentified; Fungi_sp
New.ReferenceOTU4651	0	0	0	0	0	0	0	0	0	0	0	0	0	0	0	0	0	3	0	0	0	0	0	0	0	0	0	0	0	0	Fungi; unidentified; unidentified; unidentified; unidentified; unidentified; Fungi_sp
New.ReferenceOTU4799	0	0	0	3	0	0	0	0	0	5	0	0	2	7	2	1	4	0	0	0	0	0	0	5	0	1	0	0	0	0	Fungi; unidentified; unidentified; unidentified; unidentified; unidentified; Fungi_sp
New.ReferenceOTU485	0	0	1	0	0	0	0	0	0	0	0	0	0	0	0	0	0	0	0	0	0	0	0	0	0	0	0	0	0	0	Fungi; unidentified; unidentified; unidentified; unidentified; unidentified; Fungi_sp
New.ReferenceOTU4895	0	0	0	0	0	0	0	0	0	1	0	0	0	4	0	1	0	1	0	0	0	0	0	0	0	1	0	0	0	0	Fungi; unidentified; unidentified; unidentified; unidentified; unidentified; Fungi_sp
New.ReferenceOTU4957	0	0	0	0	0	0	0	0	0	0	0	0	0	0	0	0	0	0	0	7	0	0	0	0	0	0	0	0	0	0	Fungi; unidentified; unidentified; unidentified; unidentified; unidentified; Fungi_sp
New.ReferenceOTU4993	0	0	0	0	0	0	0	0	0	0	0	0	0	0	0	0	0	0	2	0	0	0	0	0	0	0	0	0	0	0	Fungi; unidentified; unidentified; unidentified; unidentified; unidentified; Fungi_sp
New.ReferenceOTU5036	0	0	0	0	0	0	0	0	0	2	4	0	0	0	0	0	3	0	0	0	0	0	0	0	0	0	0	0	0	0	Fungi; unidentified; unidentified; unidentified; unidentified; unidentified; Fungi_sp
New.ReferenceOTU5046	0	0	0	0	0	0	0	0	0	0	0	0	0	0	0	0	0	0	0	0	0	0	0	0	1	0	2	0	0	1	Fungi; unidentified; unidentified; unidentified; unidentified; unidentified; Fungi_sp
New.ReferenceOTU5068	0	1	0	1	0	0	0	0	0	2	0	0	0	3	0	0	2	0	0	0	0	0	0	0	0	0	0	0	0	0	Fungi; unidentified; unidentified; unidentified; unidentified; unidentified; Fungi_sp
New.ReferenceOTU5113	0	0	1	0	0	0	0	0	0	0	0	0	0	0	0	0	0	0	0	0	0	0	0	0	0	0	0	0	0	0	Fungi; unidentified; unidentified; unidentified; unidentified; unidentified; Fungi_sp
New.ReferenceOTU5137	0	0	0	0	0	0	0	0	0	0	0	0	0	0	0	0	1	0	0	0	0	0	0	0	0	0	0	0	0	0	Fungi; unidentified; unidentified; unidentified; unidentified; unidentified; Fungi_sp
New.ReferenceOTU5227	0	0	0	0	0	0	0	0	0	0	0	0	0	0	0	0	0	0	0	6	0	0	0	0	0	0	0	0	0	0	Fungi; unidentified; unidentified; unidentified; unidentified; unidentified; Fungi_sp
New.ReferenceOTU5228	0	0	0	0	0	0	0	0	0	0	0	0	0	0	1	0	0	0	0	0	0	0	0	0	0	0	0	0	0	0	Fungi; unidentified; unidentified; unidentified; unidentified; unidentified; Fungi_sp
New.ReferenceOTU5245	0	0	0	0	1	0	0	0	0	0	0	0	0	0	0	0	0	0	0	0	0	0	0	0	0	0	0	0	0	0	Fungi; unidentified; unidentified; unidentified; unidentified; unidentified; Fungi_sp
New.ReferenceOTU5273	0	0	0	0	0	0	0	0	0	0	0	0	0	0	0	0	0	0	0	0	0	0	1	0	0	0	0	0	0	0	Fungi; unidentified; unidentified; unidentified; unidentified; unidentified; Fungi_sp
New.ReferenceOTU5304	0	0	2	0	7	0	0	0	1	1	2	1	0	0	1	0	0	1	1	3	0	0	0	0	0	0	3	2	0	0	Fungi; unidentified; unidentified; unidentified; unidentified; unidentified; Fungi_sp
New.ReferenceOTU5315	0	0	0	0	0	0	0	0	0	0	0	0	0	0	0	0	0	0	0	0	0	0	0	1	0	0	0	0	0	0	Fungi; unidentified; unidentified; unidentified; unidentified; unidentified; Fungi_sp
New.ReferenceOTU5323	0	0	0	0	1	0	0	0	2	1	0	0	0	0	0	0	0	0	0	0	0	0	0	0	0	0	0	0	0	0	Fungi; unidentified; unidentified; unidentified; unidentified; unidentified; Fungi_sp
New.ReferenceOTU5334	1	0	1	2	0	0	0	2	12	0	1	0	0	0	0	0	0	0	0	0	0	0	0	0	0	0	0	0	2	0	Fungi; unidentified; unidentified; unidentified; unidentified; unidentified; Fungi_sp
New.ReferenceOTU5337	0	0	0	0	0	0	0	0	0	0	0	0	0	0	0	0	0	0	1	0	0	0	0	0	0	0	0	0	0	0	Fungi; unidentified; unidentified; unidentified; unidentified; unidentified; Fungi_sp
New.ReferenceOTU5339	0	0	0	0	0	0	0	0	0	0	0	0	0	0	0	0	0	0	0	0	0	0	0	2	0	0	0	0	6	0	Fungi; unidentified; unidentified; unidentified; unidentified; unidentified; Fungi_sp
New.ReferenceOTU5347	0	0	0	0	0	0	0	1	0	0	0	0	0	0	0	0	0	0	0	0	0	0	0	0	0	0	0	0	0	0	Fungi; unidentified; unidentified; unidentified; unidentified; unidentified; Fungi_sp
New.ReferenceOTU536	0	0	0	0	0	0	0	0	0	0	0	0	0	0	0	0	0	0	1	0	0	0	0	0	0	0	0	0	0	0	Fungi; unidentified; unidentified; unidentified; unidentified; unidentified; Fungi_sp
New.ReferenceOTU5389	0	0	0	2	0	0	0	0	0	0	0	0	0	0	0	0	0	0	0	0	0	0	0	0	0	0	0	0	0	0	Fungi; unidentified; unidentified; unidentified; unidentified; unidentified; Fungi_sp
New.ReferenceOTU5392	0	0	0	0	0	0	0	0	0	0	0	0	0	0	0	0	1	0	0	0	0	0	0	1	0	0	0	0	0	0	Fungi; unidentified; unidentified; unidentified; unidentified; unidentified; Fungi_sp
New.ReferenceOTU5434	0	0	0	0	0	0	0	0	0	0	0	0	0	0	0	0	0	0	0	1	0	0	0	0	0	0	0	0	0	0	Fungi; unidentified; unidentified; unidentified; unidentified; unidentified; Fungi_sp
New.ReferenceOTU5463	0	0	0	2	0	0	0	0	2	0	0	0	1	0	0	4	5	0	0	0	0	0	0	8	0	1	0	0	0	0	Fungi; unidentified; unidentified; unidentified; unidentified; unidentified; Fungi_sp
New.ReferenceOTU5500	0	0	0	0	0	0	0	0	0	0	0	0	0	0	0	0	0	0	0	1	0	0	0	0	0	0	0	0	0	0	Fungi; unidentified; unidentified; unidentified; unidentified; unidentified; Fungi_sp
New.ReferenceOTU5508	0	0	0	0	0	0	0	0	1	0	0	0	0	0	0	0	0	0	0	0	0	0	0	0	0	0	0	0	0	0	Fungi; unidentified; unidentified; unidentified; unidentified; unidentified; Fungi_sp
New.ReferenceOTU5541	0	0	0	0	0	0	0	0	0	0	0	0	0	0	0	0	0	0	0	0	0	0	0	1	0	0	0	0	0	0	Fungi; unidentified; unidentified; unidentified; unidentified; unidentified; Fungi_sp
New.ReferenceOTU5602	0	0	0	0	0	0	0	0	1	0	0	0	0	0	0	0	0	0	0	0	0	0	0	0	0	0	1	0	0	0	Fungi; unidentified; unidentified; unidentified; unidentified; unidentified; Fungi_sp
New.ReferenceOTU5614	0	0	0	0	0	0	0	0	1	0	0	0	0	0	0	0	0	0	0	0	0	0	0	0	0	0	0	0	0	0	Fungi; unidentified; unidentified; unidentified; unidentified; unidentified; Fungi_sp
New.ReferenceOTU5618	0	0	0	3	0	0	0	0	0	0	0	0	0	0	0	0	0	0	0	0	0	0	0	0	0	0	0	0	0	0	Fungi; unidentified; unidentified; unidentified; unidentified; unidentified; Fungi_sp
New.ReferenceOTU5639	0	0	0	0	0	0	0	2	0	0	0	0	0	0	0	0	0	0	0	0	0	0	0	0	0	0	0	0	0	0	Fungi; unidentified; unidentified; unidentified; unidentified; unidentified; Fungi_sp
New.ReferenceOTU5668	1	0	0	1	1	0	0	0	0	0	3	0	2	0	0	0	0	0	0	0	0	0	0	0	0	0	0	0	0	0	Fungi; unidentified; unidentified; unidentified; unidentified; unidentified; Fungi_sp
New.ReferenceOTU5733	0	0	0	0	0	0	0	0	0	0	0	0	0	0	1	0	0	0	0	0	0	0	0	0	0	0	0	0	0	0	Fungi; unidentified; unidentified; unidentified; unidentified; unidentified; Fungi_sp
New.ReferenceOTU5748	0	0	0	0	0	0	0	0	0	0	0	0	0	0	0	0	0	1	0	0	0	0	0	0	4	0	0	0	0	0	Fungi; unidentified; unidentified; unidentified; unidentified; unidentified; Fungi_sp
New.ReferenceOTU576	0	0	0	0	0	0	0	0	3	0	0	0	0	0	0	0	0	0	0	0	0	0	0	0	0	0	0	0	0	0	Fungi; unidentified; unidentified; unidentified; unidentified; unidentified; Fungi_sp
New.ReferenceOTU5785	3	0	0	4	0	0	0	0	0	0	1	0	1	0	0	1	0	0	0	0	0	0	0	0	0	0	0	0	0	0	Fungi; unidentified; unidentified; unidentified; unidentified; unidentified; Fungi_sp
New.ReferenceOTU5802	0	0	0	0	0	0	0	0	0	0	0	0	0	0	0	1	0	0	0	0	0	0	0	0	0	0	0	0	0	0	Fungi; unidentified; unidentified; unidentified; unidentified; unidentified; Fungi_sp
New.ReferenceOTU5833	0	0	0	0	0	0	0	0	0	0	0	0	0	0	0	0	0	0	0	0	0	3	0	0	0	0	0	1	0	0	Fungi; unidentified; unidentified; unidentified; unidentified; unidentified; Fungi_sp
New.ReferenceOTU596	0	0	0	0	0	0	0	0	1	0	0	0	0	0	0	0	0	0	0	0	0	0	0	0	0	0	0	0	0	0	Fungi; unidentified; unidentified; unidentified; unidentified; unidentified; Fungi_sp
New.ReferenceOTU5975	0	0	0	0	0	0	0	0	3	0	0	0	0	0	0	0	0	0	0	0	0	0	0	0	0	0	0	0	0	0	Fungi; unidentified; unidentified; unidentified; unidentified; unidentified; Fungi_sp
New.ReferenceOTU5998	0	0	0	0	2	0	0	0	0	0	0	0	0	0	0	0	0	0	0	0	0	0	0	0	0	0	0	0	0	0	Fungi; unidentified; unidentified; unidentified; unidentified; unidentified; Fungi_sp
New.ReferenceOTU6004	0	0	0	0	0	0	0	0	0	0	0	0	0	0	0	0	0	1	0	0	0	2	0	0	0	0	0	0	0	0	Fungi; unidentified; unidentified; unidentified; unidentified; unidentified; Fungi_sp
New.ReferenceOTU6021	0	0	0	0	0	0	0	0	0	0	0	0	0	0	0	0	0	0	1	0	0	0	0	0	0	0	0	0	0	0	Fungi; unidentified; unidentified; unidentified; unidentified; unidentified; Fungi_sp
New.ReferenceOTU6046	4	0	0	0	0	0	0	0	0	0	0	0	0	0	0	0	0	0	0	0	0	0	0	0	0	0	0	0	0	0	Fungi; unidentified; unidentified; unidentified; unidentified; unidentified; Fungi_sp
New.ReferenceOTU6079	0	0	0	0	0	0	0	0	0	0	0	0	0	0	0	0	0	0	0	0	0	0	0	0	0	0	0	0	1	0	Fungi; unidentified; unidentified; unidentified; unidentified; unidentified; Fungi_sp
New.ReferenceOTU608	0	0	0	0	0	0	0	0	0	0	0	0	0	0	0	6	0	0	0	0	0	0	0	0	0	0	0	0	0	0	Fungi; unidentified; unidentified; unidentified; unidentified; unidentified; Fungi_sp
New.ReferenceOTU6080	0	0	0	0	0	0	0	0	0	0	14	0	0	0	0	0	5	0	0	0	0	0	4	0	0	0	0	0	0	0	Fungi; unidentified; unidentified; unidentified; unidentified; unidentified; Fungi_sp
New.ReferenceOTU6093	2	1	0	0	0	0	0	0	0	0	0	0	0	0	0	0	0	0	0	0	0	0	0	0	0	0	0	0	0	0	Fungi; unidentified; unidentified; unidentified; unidentified; unidentified; Fungi_sp
New.ReferenceOTU6133	0	0	0	0	0	0	0	0	0	0	2	0	0	0	0	0	0	0	0	0	0	0	0	0	0	0	0	0	0	0	Fungi; unidentified; unidentified; unidentified; unidentified; unidentified; Fungi_sp
New.ReferenceOTU6145	8	1	0	37	77	0	18	13	0	10	17	0	3	15	1	6	7	0	1	0	0	0	0	2	0	0	0	0	0	0	Fungi; unidentified; unidentified; unidentified; unidentified; unidentified; Fungi_sp
New.ReferenceOTU6150	0	0	0	0	0	0	0	0	0	0	0	0	0	0	0	0	0	0	0	0	0	0	0	0	0	0	0	0	1	0	Fungi; unidentified; unidentified; unidentified; unidentified; unidentified; Fungi_sp
New.ReferenceOTU618	0	0	0	0	0	0	0	0	0	0	0	0	0	0	0	0	0	0	1	0	0	0	0	0	0	0	0	0	0	0	Fungi; unidentified; unidentified; unidentified; unidentified; unidentified; Fungi_sp
New.ReferenceOTU6244	0	0	0	0	0	0	0	0	0	0	0	0	0	0	0	0	0	0	0	0	0	1	0	0	0	0	0	0	0	0	Fungi; unidentified; unidentified; unidentified; unidentified; unidentified; Fungi_sp
New.ReferenceOTU6268	0	0	0	0	0	0	0	0	0	0	0	0	0	0	0	0	0	0	3	0	1	0	0	0	0	0	0	0	0	0	Fungi; unidentified; unidentified; unidentified; unidentified; unidentified; Fungi_sp
New.ReferenceOTU6342	0	0	0	0	0	0	0	2	0	0	0	0	0	0	0	0	0	0	0	0	0	0	0	0	0	0	0	0	0	0	Fungi; unidentified; unidentified; unidentified; unidentified; unidentified; Fungi_sp
New.ReferenceOTU6469	0	0	0	0	0	0	0	0	0	0	0	0	0	0	0	0	0	0	1	0	0	0	0	0	0	0	0	0	0	0	Fungi; unidentified; unidentified; unidentified; unidentified; unidentified; Fungi_sp
New.ReferenceOTU6511	0	0	0	0	0	0	0	0	0	0	0	0	1	0	0	0	0	0	0	0	0	0	0	0	0	0	0	0	0	0	Fungi; unidentified; unidentified; unidentified; unidentified; unidentified; Fungi_sp
New.ReferenceOTU6523	6	0	0	39	1	0	0	0	0	0	9	0	1	0	0	0	0	0	0	0	0	0	0	0	0	0	0	0	0	0	Fungi; unidentified; unidentified; unidentified; unidentified; unidentified; Fungi_sp
New.ReferenceOTU6529	0	0	0	0	0	0	0	0	0	0	0	0	0	0	0	0	0	0	1	0	0	0	0	0	0	0	0	0	0	0	Fungi; unidentified; unidentified; unidentified; unidentified; unidentified; Fungi_sp
New.ReferenceOTU6607	0	0	0	0	0	0	0	0	2	0	0	0	0	0	0	0	0	0	0	0	0	0	0	0	0	0	0	0	0	0	Fungi; unidentified; unidentified; unidentified; unidentified; unidentified; Fungi_sp
New.ReferenceOTU6613	0	0	0	0	0	0	0	0	2	0	0	0	0	0	0	0	0	0	0	0	0	0	0	0	0	0	0	0	0	0	Fungi; unidentified; unidentified; unidentified; unidentified; unidentified; Fungi_sp
New.ReferenceOTU6654	0	0	0	0	0	0	0	0	0	0	0	0	0	1	0	0	0	0	0	0	0	0	0	0	0	0	0	0	0	0	Fungi; unidentified; unidentified; unidentified; unidentified; unidentified; Fungi_sp
New.ReferenceOTU6811	0	0	0	0	0	0	0	0	0	0	0	0	0	0	0	0	0	0	1	0	0	0	0	0	0	0	0	0	0	0	Fungi; unidentified; unidentified; unidentified; unidentified; unidentified; Fungi_sp
New.ReferenceOTU6818	0	0	0	0	0	0	0	19	0	0	0	0	0	0	0	0	0	0	0	0	0	0	0	0	0	0	0	0	0	0	Fungi; unidentified; unidentified; unidentified; unidentified; unidentified; Fungi_sp
New.ReferenceOTU6832	0	0	0	0	0	0	0	0	0	0	0	0	0	0	0	0	0	0	4	0	0	0	0	0	0	0	0	11	0	0	Fungi; unidentified; unidentified; unidentified; unidentified; unidentified; Fungi_sp
New.ReferenceOTU6847	0	0	0	0	4	0	0	0	0	0	1	0	0	0	0	0	0	0	0	0	0	0	0	0	0	0	0	0	0	0	Fungi; unidentified; unidentified; unidentified; unidentified; unidentified; Fungi_sp
New.ReferenceOTU6857	0	0	0	5	13	0	0	0	0	0	0	9	4	1	9	0	0	0	0	0	0	0	0	0	0	0	0	0	0	0	Fungi; unidentified; unidentified; unidentified; unidentified; unidentified; Fungi_sp
New.ReferenceOTU6889	0	0	0	0	1	0	0	0	0	0	0	0	0	0	0	0	0	0	0	0	0	0	0	0	0	0	0	0	0	0	Fungi; unidentified; unidentified; unidentified; unidentified; unidentified; Fungi_sp
New.ReferenceOTU6918	0	0	0	0	0	0	0	0	0	0	0	0	0	0	0	0	0	0	0	0	0	0	0	0	3	0	0	0	0	0	Fungi; unidentified; unidentified; unidentified; unidentified; unidentified; Fungi_sp
New.ReferenceOTU6966	0	1	0	0	0	0	0	0	0	0	0	0	0	0	0	0	0	0	0	0	0	0	0	0	0	0	0	0	0	0	Fungi; unidentified; unidentified; unidentified; unidentified; unidentified; Fungi_sp
New.ReferenceOTU699	0	0	0	0	0	0	0	40	0	0	0	0	0	0	0	0	0	0	0	0	0	0	0	0	0	0	0	0	0	0	Fungi; unidentified; unidentified; unidentified; unidentified; unidentified; Fungi_sp
New.ReferenceOTU7014	0	0	0	0	0	0	0	0	0	0	0	0	0	0	0	0	1	0	0	0	0	0	2	0	0	0	0	0	0	0	Fungi; unidentified; unidentified; unidentified; unidentified; unidentified; Fungi_sp
New.ReferenceOTU7098	0	0	0	0	0	0	0	0	0	0	0	0	0	0	0	0	0	0	0	0	0	0	0	0	0	0	0	0	6	0	Fungi; unidentified; unidentified; unidentified; unidentified; unidentified; Fungi_sp
New.ReferenceOTU7114	0	0	0	0	0	0	0	0	0	0	0	0	1	0	0	0	1	0	0	0	0	0	0	0	0	0	0	0	0	0	Fungi; unidentified; unidentified; unidentified; unidentified; unidentified; Fungi_sp
New.ReferenceOTU7148	1	0	0	0	0	0	0	0	0	0	0	0	0	0	0	0	0	0	0	0	0	0	0	0	0	0	0	0	0	0	Fungi; unidentified; unidentified; unidentified; unidentified; unidentified; Fungi_sp
New.ReferenceOTU7178	0	0	0	0	0	0	0	1	0	0	0	0	0	0	0	0	0	0	0	0	0	0	0	0	0	0	0	0	0	0	Fungi; unidentified; unidentified; unidentified; unidentified; unidentified; Fungi_sp
New.ReferenceOTU7222	0	0	0	0	0	0	0	0	0	0	0	0	0	0	0	4	0	0	0	0	0	0	0	0	0	0	0	0	0	0	Fungi; unidentified; unidentified; unidentified; unidentified; unidentified; Fungi_sp
New.ReferenceOTU7301	0	0	0	0	0	0	2	0	0	0	0	0	0	0	0	0	0	0	0	0	0	0	0	0	0	0	0	0	0	0	Fungi; unidentified; unidentified; unidentified; unidentified; unidentified; Fungi_sp
New.ReferenceOTU7315	3	0	0	13	0	0	0	0	0	0	5	0	0	0	0	0	0	0	0	0	0	0	0	0	0	0	0	0	0	0	Fungi; unidentified; unidentified; unidentified; unidentified; unidentified; Fungi_sp
New.ReferenceOTU734	0	0	0	0	0	0	0	0	0	0	0	0	0	0	0	0	0	0	3	0	0	0	0	0	0	0	0	0	0	0	Fungi; unidentified; unidentified; unidentified; unidentified; unidentified; Fungi_sp
New.ReferenceOTU7354	0	0	0	0	0	0	0	0	0	1	0	1	0	3	0	2	1	0	0	0	0	0	0	0	0	0	0	0	0	0	Fungi; unidentified; unidentified; unidentified; unidentified; unidentified; Fungi_sp
New.ReferenceOTU7386	0	2	2	0	0	0	0	0	0	0	0	0	0	0	0	0	0	0	0	1	0	0	0	0	0	0	0	0	0	0	Fungi; unidentified; unidentified; unidentified; unidentified; unidentified; Fungi_sp
New.ReferenceOTU7391	0	0	0	0	0	0	0	0	0	0	0	0	0	0	0	0	0	0	0	0	0	0	0	0	0	2	0	0	0	0	Fungi; unidentified; unidentified; unidentified; unidentified; unidentified; Fungi_sp
New.ReferenceOTU7411	0	0	1	0	0	0	0	0	0	0	0	0	0	0	0	0	0	0	0	0	0	0	0	0	0	0	0	0	0	0	Fungi; unidentified; unidentified; unidentified; unidentified; unidentified; Fungi_sp
New.ReferenceOTU7469	0	0	0	0	0	0	0	0	0	0	0	0	0	0	0	1	0	0	0	0	0	0	0	1	0	0	0	3	0	0	Fungi; unidentified; unidentified; unidentified; unidentified; unidentified; Fungi_sp
New.ReferenceOTU7485	0	0	0	0	0	0	0	0	0	0	0	0	0	0	0	0	0	4	0	0	0	0	0	2	0	0	0	0	0	0	Fungi; unidentified; unidentified; unidentified; unidentified; unidentified; Fungi_sp
New.ReferenceOTU7496	1	0	0	0	0	0	0	1	1	0	0	0	0	0	0	0	0	0	0	0	0	0	0	0	0	0	0	0	0	0	Fungi; unidentified; unidentified; unidentified; unidentified; unidentified; Fungi_sp
New.ReferenceOTU7500	0	0	0	0	0	0	0	1	0	0	0	0	0	0	0	0	0	0	1	0	0	0	0	0	0	0	0	0	0	0	Fungi; unidentified; unidentified; unidentified; unidentified; unidentified; Fungi_sp
New.ReferenceOTU751	0	0	0	0	0	0	0	0	0	0	0	0	0	0	0	0	0	0	0	2	0	0	0	0	0	0	0	0	0	0	Fungi; unidentified; unidentified; unidentified; unidentified; unidentified; Fungi_sp
New.ReferenceOTU7545	0	0	0	0	0	0	0	0	1	0	0	0	1	0	0	0	0	0	0	0	0	0	0	0	0	0	0	0	0	0	Fungi; unidentified; unidentified; unidentified; unidentified; unidentified; Fungi_sp
New.ReferenceOTU7552	0	0	0	0	0	0	0	0	0	1	0	0	0	0	1	1	0	0	0	0	0	0	0	0	0	0	0	0	0	0	Fungi; unidentified; unidentified; unidentified; unidentified; unidentified; Fungi_sp
New.ReferenceOTU7568	0	0	0	0	0	0	0	18	0	0	0	0	0	0	0	0	0	0	0	0	0	0	0	4	0	0	0	0	1	0	Fungi; unidentified; unidentified; unidentified; unidentified; unidentified; Fungi_sp

New.ReferenceOTU7713	1	0	0	0	0	0	0	0	0	0	0	0	0	1	0	0	0	0	0	0	0	0	0	0	0	0	0	0	0	0	Fungi; unidentified; unidentified; unidentified; unidentified; unidentified; Fungi_sp
New.ReferenceOTU7763	0	0	0	0	0	0	0	0	0	0	0	0	0	0	0	0	0	0	0	0	0	0	1	0	0	0	0	0	0	0	Fungi; unidentified; unidentified; unidentified; unidentified; unidentified; Fungi_sp
New.ReferenceOTU7774	0	0	0	0	7	0	0	0	0	0	0	0	0	0	0	0	0	0	0	0	0	0	0	0	0	0	0	0	0	0	Fungi; unidentified; unidentified; unidentified; unidentified; unidentified; Fungi_sp
New.ReferenceOTU7803	0	0	0	0	0	0	0	0	0	0	0	0	0	0	0	0	0	0	0	0	0	1	0	0	0	0	0	0	0	0	Fungi; unidentified; unidentified; unidentified; unidentified; unidentified; Fungi_sp
New.ReferenceOTU784	0	0	0	0	0	0	3	0	0	0	0	0	0	0	0	0	2	0	0	0	0	0	0	1	0	0	0	0	0	0	Fungi; unidentified; unidentified; unidentified; unidentified; unidentified; Fungi_sp
New.ReferenceOTU7938	0	0	0	0	0	0	0	0	0	0	0	0	0	0	1	0	0	0	0	0	0	0	1	0	0	0	0	0	0	0	Fungi; unidentified; unidentified; unidentified; unidentified; unidentified; Fungi_sp
New.ReferenceOTU7958	0	0	0	0	0	0	1	0	0	0	0	0	0	0	0	0	0	0	0	0	0	0	0	0	0	0	0	0	0	0	Fungi; unidentified; unidentified; unidentified; unidentified; unidentified; Fungi_sp
New.ReferenceOTU798	0	0	0	3	0	0	0	0	0	0	0	0	0	0	0	0	0	0	0	0	0	0	0	0	0	0	0	0	0	0	Fungi; unidentified; unidentified; unidentified; unidentified; unidentified; Fungi_sp
New.ReferenceOTU803	0	0	0	0	0	0	0	0	0	0	0	0	0	1	0	0	0	0	0	0	0	0	0	0	0	0	0	0	0	0	Fungi; unidentified; unidentified; unidentified; unidentified; unidentified; Fungi_sp
New.ReferenceOTU8040	0	0	0	0	0	0	0	0	0	0	0	0	3	0	0	0	0	0	0	0	0	0	0	0	0	0	0	0	0	0	Fungi; unidentified; unidentified; unidentified; unidentified; unidentified; Fungi_sp
New.ReferenceOTU8065	0	0	0	0	0	0	0	0	1	0	0	0	0	0	0	0	0	0	0	0	0	0	0	0	0	0	0	0	0	0	Fungi; unidentified; unidentified; unidentified; unidentified; unidentified; Fungi_sp
New.ReferenceOTU8091	0	0	0	0	0	0	0	0	1	0	0	0	0	0	0	0	0	0	0	0	0	0	0	0	0	0	0	0	0	0	Fungi; unidentified; unidentified; unidentified; unidentified; unidentified; Fungi_sp
New.ReferenceOTU8092	0	0	0	0	0	0	0	0	0	0	0	0	0	0	0	0	0	0	1	0	0	0	0	0	0	0	0	0	0	0	Fungi; unidentified; unidentified; unidentified; unidentified; unidentified; Fungi_sp
New.ReferenceOTU816	0	0	0	0	0	0	0	0	0	0	0	0	0	0	0	3	0	2	0	0	0	0	0	0	0	0	0	0	0	0	Fungi; unidentified; unidentified; unidentified; unidentified; unidentified; Fungi_sp
New.ReferenceOTU834	0	0	0	0	0	0	0	0	0	0	0	0	0	0	0	0	0	0	1	0	0	0	0	0	0	0	0	0	0	0	Fungi; unidentified; unidentified; unidentified; unidentified; unidentified; Fungi_sp
New.ReferenceOTU859	0	0	0	0	0	0	0	0	0	0	0	0	0	0	0	0	0	0	0	0	0	0	0	2	0	0	0	0	0	0	Fungi; unidentified; unidentified; unidentified; unidentified; unidentified; Fungi_sp
New.ReferenceOTU868	0	0	0	0	3	0	0	0	0	0	0	0	0	0	0	0	0	0	0	0	0	0	0	0	0	0	0	0	0	0	Fungi; unidentified; unidentified; unidentified; unidentified; unidentified; Fungi_sp
New.ReferenceOTU908	0	0	0	0	0	0	0	0	0	0	0	0	0	0	0	0	0	0	0	0	0	0	1	0	0	0	0	0	0	0	Fungi; unidentified; unidentified; unidentified; unidentified; unidentified; Fungi_sp
New.ReferenceOTU945	0	0	0	0	0	0	0	0	0	0	0	0	0	1	0	0	0	0	0	0	0	0	0	0	0	0	0	0	0	0	Fungi; unidentified; unidentified; unidentified; unidentified; unidentified; Fungi_sp
New.ReferenceOTU973	0	0	0	0	0	0	0	1	0	0	0	0	0	0	0	0	0	0	0	0	0	0	0	0	0	0	0	0	0	0	Fungi; unidentified; unidentified; unidentified; unidentified; unidentified; Fungi_sp
New.ReferenceOTU990	0	0	0	0	0	0	1	0	0	0	0	0	0	0	0	0	0	0	0	0	0	0	0	0	0	0	0	0	0	0	Fungi; unidentified; unidentified; unidentified; unidentified; unidentified; Fungi_sp
SH038654.06FU_UDB013022_reps_singleton	0	3	1	1	0	0	0	0	1	0	0	0	0	0	0	0	0	9	0	0	0	0	0	0	0	0	0	0	0	0	Fungi; unidentified; unidentified; unidentified; unidentified; unidentified; Fungi_sp
SH094129.06FU_EF619875_reps	0	0	0	0	0	0	0	0	0	0	0	0	3	0	0	0	0	0	0	0	0	0	0	0	0	0	0	0	0	0	Fungi; unidentified; unidentified; unidentified; unidentified; unidentified; Fungi_sp
SH121337.06FU_JQ410056_reps	0	0	0	0	0	0	0	0	1	0	0	0	0	0	0	0	0	0	0	0	0	0	0	0	0	0	0	0	0	0	Fungi; unidentified; unidentified; unidentified; unidentified; unidentified; Fungi_sp
SH122635.06FU_DQ421188_reps	0	1	0	0	0	0	0	0	0	0	0	0	0	0	0	0	0	0	0	0	0	0	0	0	0	6	0	0	14	0	Fungi; unidentified; unidentified; unidentified; unidentified; unidentified; Fungi_sp
SH140463.06FU_GQ851775_reps	0	0	0	0	0	0	0	0	0	0	1	0	0	0	0	0	0	0	0	0	0	0	0	0	0	0	0	0	0	0	Fungi; unidentified; unidentified; unidentified; unidentified; unidentified; Fungi_sp
SH147349.06FU_JQ747723_reps	0	0	0	0	0	0	0	0	0	0	0	2	0	0	0	0	0	0	0	0	0	0	0	0	0	0	0	0	0	0	Fungi; unidentified; unidentified; unidentified; unidentified; unidentified; Fungi_sp
New.ReferenceOTU4676	10	0	0	0	0	0	0	0	0	0	0	0	0	0	0	0	0	0	0	0	0	0	0	0	0	0	0	0	0	0	Fungi; unidentified; unidentified; unidentified; unidentified; unidentified; root_associated_fungal_sp_EP2_1
New.ReferenceOTU1281	0	0	0	0	0	0	0	0	0	0	0	0	0	0	0	0	0	0	0	0	0	0	0	0	0	0	0	0	1	0	Fungi; unidentified; unidentified; unidentified; unidentified; unidentified; uncultured_basal_lineage_fungus
New.ReferenceOTU2309	0	0	0	0	0	0	0	0	0	0	0	0	0	2	0	0	0	0	0	0	0	0	0	0	0	0	0	0	0	0	Fungi; unidentified; unidentified; unidentified; unidentified; unidentified; uncultured_basal_lineage_fungus
New.ReferenceOTU4175	0	0	0	0	0	0	0	2	0	1	0	0	0	0	0	0	0	0	0	0	1	0	0	0	0	0	0	0	0	1	Fungi; unidentified; unidentified; unidentified; unidentified; unidentified; uncultured_basal_lineage_fungus
New.ReferenceOTU4552	0	2	2	2	8	0	0	0	0	0	0	10	0	1	0	0	0	0	0	0	0	0	0	2	0	1	0	0	1	0	Fungi; unidentified; unidentified; unidentified; unidentified; unidentified; uncultured_basal_lineage_fungus
New.ReferenceOTU4900	0	0	0	1	0	0	0	0	0	0	0	0	0	0	0	0	0	0	0	0	0	0	0	0	0	0	0	0	0	0	Fungi; unidentified; unidentified; unidentified; unidentified; unidentified; uncultured_basal_lineage_fungus
New.ReferenceOTU6380	0	4	0	0	0	0	0	0	0	0	0	0	0	0	0	0	0	0	0	0	0	0	0	0	0	0	0	0	0	0	Fungi; unidentified; unidentified; unidentified; unidentified; unidentified; uncultured_basal_lineage_fungus
New.ReferenceOTU7171	0	0	0	0	0	0	0	1	0	0	0	0	0	0	0	0	0	0	0	0	0	0	0	0	0	0	0	0	0	4	Fungi; unidentified; unidentified; unidentified; unidentified; unidentified; uncultured_basal_lineage_fungus
New.ReferenceOTU966	0	2	0	0	2	0	0	0	0	0	0	0	0	1	0	0	0	0	0	0	0	0	0	0	0	0	0	0	0	0	Fungi; unidentified; unidentified; unidentified; unidentified; unidentified; uncultured_basal_lineage_fungus
New.ReferenceOTU644	0	0	0	0	0	0	0	0	0	0	0	0	0	0	0	7	0	0	0	0	0	0	0	0	0	0	0	0	0	0	Fungi; unidentified; unidentified; unidentified; unidentified; unidentified; uncultured_compost_fungus
New.ReferenceOTU1573	3	0	0	3	0	0	0	0	0	0	1	1	5	0	1	0	0	0	0	0	0	0	0	0	0	0	0	0	0	0	Fungi; unidentified; unidentified; unidentified; unidentified; unidentified; uncultured_ectomycorrhizal_fungus
New.ReferenceOTU1708	1	0	0	0	0	0	0	0	0	0	0	0	1	0	0	0	0	0	0	0	0	0	0	0	0	0	0	1	0	0	Fungi; unidentified; unidentified; unidentified; unidentified; unidentified; uncultured_ectomycorrhizal_fungus
New.ReferenceOTU2406	2	0	0	0	0	0	2	0	0	0	0	6	1	0	0	0	0	0	0	0	0	0	0	0	0	0	0	0	0	0	Fungi; unidentified; unidentified; unidentified; unidentified; unidentified; uncultured_ectomycorrhizal_fungus
New.ReferenceOTU4176	0	0	1	0	0	0	3	0	0	0	0	0	1	0	0	0	0	0	0	0	0	0	0	0	0	0	0	0	0	0	Fungi; unidentified; unidentified; unidentified; unidentified; unidentified; uncultured_ectomycorrhizal_fungus
New.ReferenceOTU5292	0	0	0	0	0	0	0	0	0	0	0	0	3	0	0	0	0	0	0	0	0	0	0	0	0	0	0	0	0	0	Fungi; unidentified; unidentified; unidentified; unidentified; unidentified; uncultured_ectomycorrhizal_fungus
New.ReferenceOTU5527	0	0	0	0	1	0	1	0	0	0	0	0	0	0	0	0	0	0	0	0	0	0	0	0	0	0	0	0	0	0	Fungi; unidentified; unidentified; unidentified; unidentified; unidentified; uncultured_ectomycorrhizal_fungus
New.ReferenceOTU5893	0	0	0	0	0	1	0	0	0	0	1	0	0	0	0	0	0	0	0	0	0	0	0	0	0	0	0	0	0	0	Fungi; unidentified; unidentified; unidentified; unidentified; unidentified; uncultured_ectomycorrhizal_fungus
New.ReferenceOTU6011	0	0	0	0	0	0	0	0	0	0	0	1	0	0	0	0	0	0	0	0	0	0	0	0	0	0	0	0	0	0	Fungi; unidentified; unidentified; unidentified; unidentified; unidentified; uncultured_ectomycorrhizal_fungus
New.ReferenceOTU6638	0	0	0	0	0	0	0	0	0	0	0	0	0	0	1	0	0	0	0	0	0	0	0	0	0	0	0	0	0	0	Fungi; unidentified; unidentified; unidentified; unidentified; unidentified; uncultured_ectomycorrhizal_fungus
New.ReferenceOTU6707	0	0	0	0	0	0	0	0	0	0	0	0	0	0	0	0	0	0	0	1	0	0	0	0	0	0	0	0	0	0	Fungi; unidentified; unidentified; unidentified; unidentified; unidentified; uncultured_ectomycorrhizal_fungus
New.ReferenceOTU7132	0	0	0	1	0	0	0	0	0	0	0	0	0	0	0	0	0	0	0	0	0	0	0	0	0	0	0	0	0	0	Fungi; unidentified; unidentified; unidentified; unidentified; unidentified; uncultured_ectomycorrhizal_fungus
New.ReferenceOTU887	1	0	0	0	0	0	0	0	0	0	0	0	5	0	0	0	0	0	0	0	0	0	0	0	0	0	0	0	0	0	Fungi; unidentified; unidentified; unidentified; unidentified; unidentified; uncultured_ectomycorrhizal_fungus
SH018945.06FU_AB839378_reps_singleton	0	0	0	0	0	1	0	0	0	0	0	1	5	0	0	0	0	0	0	0	0	0	0	0	0	0	0	1	0	0	Fungi; unidentified; unidentified; unidentified; unidentified; unidentified; uncultured_ectomycorrhizal_fungus
New.ReferenceOTU2743	0	0	0	0	0	0	0	0	0	0	0	0	0	0	0	0	0	0	0	0	0	0	0	1	0	0	0	0	0	0	Fungi; unidentified; unidentified; unidentified; unidentified; unidentified; uncultured_endophytic_fungus
New.ReferenceOTU3169	0	0	0	0	2	0	0	0	0	0	0	2	0	0	0	0	0	0	0	0	0	0	0	0	0	0	1	0	0	0	Fungi; unidentified; unidentified; unidentified; unidentified; unidentified; uncultured_endophytic_fungus
New.ReferenceOTU5103	1	0	0	1	0	0	0	0	0	0	0	0	0	0	0	0	0	0	0	0	0	0	0	0	0	0	0	0	0	0	Fungi; unidentified; unidentified; unidentified; unidentified; unidentified; uncultured_endophytic_fungus
New.CleanUp.ReferenceOTU1491	0	0	0	0	0	0	0	0	0	0	0	0	0	0	0	0	0	1	1	0	0	0	0	0	0	0	0	0	0	0	Fungi; unidentified; unidentified; unidentified; unidentified; unidentified; uncultured_fungus
New.CleanUp.ReferenceOTU1518	0	0	0	0	0	0	0	0	0	0	0	0	0	0	0	0	0	0	0	0	0	0	0	0	0	0	1	0	0	0	Fungi; unidentified; unidentified; unidentified; unidentified; unidentified; uncultured_fungus
New.CleanUp.ReferenceOTU2006	0	0	0	0	0	0	0	0	0	0	0	0	0	0	0	0	0	3	0	0	0	0	0	0	0	0	0	0	0	0	Fungi; unidentified; unidentified; unidentified; unidentified; unidentified; uncultured_fungus
New.CleanUp.ReferenceOTU2186	2	0	0	0	0	0	0	0	0	0	0	0	0	0	0	0	0	0	0	0	0	0	0	0	0	0	0	0	0	0	Fungi; unidentified; unidentified; unidentified; unidentified; unidentified; uncultured_fungus
New.CleanUp.ReferenceOTU2215	0	0	0	0	0	0	0	0	0	0	0	0	0	0	1	0	0	0	0	0	0	0	0	0	0	0	0	0	0	0	Fungi; unidentified; unidentified; unidentified; unidentified; unidentified; uncultured_fungus
New.ReferenceOTU1006	0	0	0	0	0	0	1	0	0	0	0	0	0	0	0	0	0	0	0	0	0	0	0	0	0	0	0	0	0	0	Fungi; unidentified; unidentified; unidentified; unidentified; unidentified; uncultured_fungus
New.ReferenceOTU1025	0	0	0	0	0	0	0	0	0	0	0	0	0	0	0	0	0	0	0	0	0	2	1	0	0	0	0	0	0	0	Fungi; unidentified; unidentified; unidentified; unidentified; unidentified; uncultured_fungus
New.ReferenceOTU1083	0	0	0	0	0	0	0	0	0	0	0	0	0	0	0	0	0	4	0	0	0	0	0	0	0	0	0	0	0	0	Fungi; unidentified; unidentified; unidentified; unidentified; unidentified; uncultured_fungus
New.ReferenceOTU1088	0	0	0	0	0	0	0	0	0	0	0	0	0	0	0	0	1	0	0	0	0	0	0	3	0	0	1	0	0	0	Fungi; unidentified; unidentified; unidentified; unidentified; unidentified; uncultured_fungus
New.ReferenceOTU1095	0	0	0	0	0	0	0	0	0	0	0	0	0	0	0	0	0	1	0	0	0	0	0	0	0	0	0	0	0	0	Fungi; unidentified; unidentified; unidentified; unidentified; unidentified; uncultured_fungus
New.ReferenceOTU1103	0	0	0	0	0	0	0	0	0	0	0	0	0	0	0	0	1	0	0	0	0	0	0	0	0	0	0	0	0	0	Fungi; unidentified; unidentified; unidentified; unidentified; unidentified; uncultured_fungus
New.ReferenceOTU1152	0	0	0	0	0	0	0	0	0	0	0	0	0	0	0	0	0	0	0	0	0	0	0	0	0	6	0	0	0	0	Fungi; unidentified; unidentified; unidentified; unidentified; unidentified; uncultured_fungus
New.ReferenceOTU1198	0	0	0	0	0	0	0	0	0	0	0	0	0	0	0	0	0	0	3	0	0	0	0	0	0	0	0	0	0	0	Fungi; unidentified; unidentified; unidentified; unidentified; unidentified; uncultured_fungus
New.ReferenceOTU1216	0	0	0	0	0	0	0	0	0	0	0	0	0	1	0	0	0	0	0	0	0	0	0	0	0	0	0	0	0	0	Fungi; unidentified; unidentified; unidentified; unidentified; unidentified; uncultured_fungus
New.ReferenceOTU1230	0	0	0	0	0	0	0	0	0	0	0	0	0	0	0	0	0	0	0	0	0	1	0	0	0	0	0	0	0	0	Fungi; unidentified; unidentified; unidentified; unidentified; unidentified; uncultured_fungus
New.ReferenceOTU1243	0	0	0	0	0	0	0	0	0	0	0	1	0	0	1	0	0	0	0	0	0	0	0	0	0	0	0	0	0	2	Fungi; unidentified; unidentified; unidentified; unidentified; unidentified; uncultured_fungus
New.ReferenceOTU1268	1	0	0	0	0	0	0	0	0	0	1	0	0	0	0	0	0	0	0	0	0	0	0	0	0	0	0	0	0	0	Fungi; unidentified; unidentified; unidentified; unidentified; unidentified; uncultured_fungus
New.ReferenceOTU129	0	0	0	0	0	0	0	0	0	0	0	0	0	0	0	0	0	4	0	0	0	0	0	0	0	1	0	0	0	0	Fungi; unidentified; unidentified; unidentified; unidentified; unidentified; uncultured_fungus
New.ReferenceOTU1313	0	1	2	1	0	0	0	0	0	0	0	0	0	0	0	0	0	0	0	0	0	0	0	0	0	0	0	0	0	0	Fungi; unidentified; unidentified; unidentified; unidentified; unidentified; uncultured_fungus
New.ReferenceOTU1348	0	0	0	0	0	0	2	0	0	0	0	0	0	0	0	0	0	0	0	0	0	0	0	0	0	0	0	0	0	0	Fungi; unidentified; unidentified; unidentified; unidentified; unidentified; uncultured_fungus
New.ReferenceOTU1383	0	0	0	0	0	0	0	0	0	0	0	0	0	0	0	0	0	0	0	0	0	0	0	0	0	0	0	1	0	0	Fungi; unidentified; unidentified; unidentified; unidentified; unidentified; uncultured_fungus
New.ReferenceOTU1394	1	0	0	0	0	0	0	0	0	0	0	0	0	0	0	0	0	0	0	0	0	0	0	0	0	0	0	0	0	0	Fungi; unidentified; unidentified; unidentified; unidentified; unidentified; uncultured_fungus
New.ReferenceOTU1441	2	0	0	0	0	0	0	0	0	0	0	0	0	0	0	0	0	0	0	0	0	0	0	0	0	0	0	0	0	0	Fungi; unidentified; unidentified; unidentified; unidentified; unidentified; uncultured_fungus
New.ReferenceOTU1444	0	0	0	0	0	0	0	0	0	0	0	0	1	0	0	0	0	0	0	0	0	0	0	0	0	0	0	0	0	0	Fungi; unidentified; unidentified; unidentified; unidentified; unidentified; uncultured_fungus
New.ReferenceOTU1516	0	0	0	1	0	0	0	0	0	0	0	0	0	0	0	0	0	0	0	0	0	0	0	0	0	0	0	0	1	0	Fungi; unidentified; unidentified; unidentified; unidentified; unidentified; uncultured_fungus
New.ReferenceOTU1518	0	0	0	2	0	0	0	0	0	0	0	0	0	0	0	0	0	0	0	0	0	0	0	0	0	0	0	0	0	0	Fungi; unidentified; unidentified; unidentified; unidentified; unidentified; uncultured_fungus
New.ReferenceOTU1523	1	0	0	0	0	0	0	0	0	0	0	0	0	0	0	0	0	0	0	0	0	0	0	0	0	0	0	0	0	0	Fungi; unidentified; unidentified; unidentified; unidentified; unidentified; uncultured_fungus
New.ReferenceOTU1550	0	0	0	0	0	0	0	1	0	0	0	0	0	0	0	0	0	0	0	0	0	0	0	0	0	0	0	0	0	0	Fungi; unidentified; unidentified; unidentified; unidentified; unidentified; uncultured_fungus
New.ReferenceOTU1569	0	0	0	0	0	0	0	0	0	0	0	0	0	0	0	0	1	0	0	0	0	0	0	0	0	0	0	0	0	0	Fungi; unidentified; unidentified; unidentified; unidentified; unidentified; uncultured_fungus
New.ReferenceOTU1578	0	0	0	0	0	0	0	0	0	0	0	0	0	0	0	0	0	0	0	0	0	0	0	0	0	0	0	1	0	0	Fungi; unidentified; unidentified; unidentified; unidentified; unidentified; uncultured_fungus
New.ReferenceOTU1647	0	0	0	1	0	0	0	0	0	0	0	0	0	0	0	0	0	0	0	0	0	0	0	0	0	0	0	0	0	0	Fungi; unidentified; unidentified; unidentified; unidentified; unidentified; uncultured_fungus
New.ReferenceOTU1672	0	0	0	0	0	0	0	0	0	0	0	0	0	0	0	0	0	0	1	0	0	0	0	0	0	0	0	0	0	0	Fungi; unidentified; unidentified; unidentified; unidentified; unidentified; uncultured_fungus
New.ReferenceOTU1673	0	0	0	0	0	0	0	0	1	0	0	0	0	0	0	0	0	0	0	0	0	0	0	0	0	0	0	0	0	0	Fungi; unidentified; unidentified; unidentified; unidentified; unidentified; uncultured_fungus
New.ReferenceOTU1682	0	0	0	0	0	0	0	0	0	0	0	0	0	0	0	0	0	0	0	0	0	0	0	0	0	0	0	4	0	0	Fungi; unidentified; unidentified; unidentified; unidentified; unidentified; uncultured_fungus
New.ReferenceOTU1784	0	0	0	0	0	0	0	0	0	0	0	1	0	0	0	0	0	0	0	0	0	0	0	0	0	0	0	0	0	0	Fungi; unidentified; unidentified; unidentified; unidentified; unidentified; uncultured_fungus
New.ReferenceOTU1806	1	2	0	0	0	0	0	0	0	0	2	0	1	1	0	2	0	1	1	0	0	0	0	0	0	0	0	0	1	0	Fungi; unidentified; unidentified; unidentified; unidentified; unidentified; uncultured_fungus
New.ReferenceOTU1854	0	0	0	0	0	0	0	0	0	0	1	0	0	0	0	0	0	0	0	0	0	0	0	0	0	0	0	0	0	0	Fungi; unidentified; unidentified; unidentified; unidentified; unidentified; uncultured_fungus
New.ReferenceOTU1878	0	0	0	0	0	0	0	0	0	0	0	0	0	0	0	0	0	0	1	0	0	0	0	0	0	0	0	0	0	0	Fungi; unidentified; unidentified; unidentified; unidentified; unidentified; uncultured_fungus
New.ReferenceOTU1883	0	0	0	0	0	0	0	0	0	0	0	0	0	1	0	0	0	0	0	0	0	0	0	0	0	0	0	0	0	0	Fungi; unidentified; unidentified; unidentified; unidentified; unidentified; uncultured_fungus
New.ReferenceOTU1907	0	0	1	0	0	0	0	0	0	0	0	0	0	0	0	0	0	0	0	0	0	0	0	0	0	0	0	0	0	0	Fungi; unidentified; unidentified; unidentified; unidentified; unidentified; uncultured_fungus
New.ReferenceOTU1936	0	0	0	0	1	0	0	0	0	0	0	0	0	0	0	0	0	0	0	0	0	0	0	0	0	0	0	0	0	0	Fungi; unidentified; unidentified; unidentified; unidentified; unidentified; uncultured_fungus
New.ReferenceOTU1986	0	0	0	0	0	0	0	0	0	0	0	0	0	0	0	0	0	0	0	0	0	0	1	0	0	0	0	0	0	0	Fungi; unidentified; unidentified; unidentified; unidentified; unidentified; uncultured_fungus
New.ReferenceOTU2004	0	0	0	0	0	0	0	0	0	0	0	0	0	0	0	0	0	0	0	0	0	0	0	0	0	0	0	0	1	0	Fungi; unidentified; unidentified; unidentified; unidentified; unidentified; uncultured_fungus
New.ReferenceOTU2007	0	0	0	0	2	0	0	0	0	0	0	0	0	0	0	0	0	0	0	0	0	0	0	1	0	0	0	0	0	0	Fungi; unidentified; unidentified; unidentified; unidentified; unidentified; uncultured_fungus
New.ReferenceOTU2058	0	0	0	1	0	0	0	0	0	0	0	0	0	0	0	0	0	0	0	0	0	0	0	0	0	0	0	0	0	0	Fungi; unidentified; unidentified; unidentified; unidentified; unidentified; uncultured_fungus
New.ReferenceOTU207	0	0	0	0	0	0	0	0	0	0	0	0	0	1	1	0	0	0	0	0	0	0	0	0	0	0	0	0	0	0	Fungi; unidentified; unidentified; unidentified; unidentified; unidentified; uncultured_fungus
New.ReferenceOTU2080	0	0	0	0	0	0	0	0	0	0	0	0	1	0	0	0	0	0	0	0	0	0	0	0	0	0	0	0	0	0	Fungi; unidentified; unidentified; unidentified; unidentified; unidentified; uncultured_fungus

New.ReferenceOTU2221	0	0	0	0	0	0	0	0	0	0	0	0	0	0	0	0	0	0	0	0	0	0	1	0	0	0	0	0	0	0	Fungi; unidentified; unidentified; unidentified; unidentified; unidentified; uncultured_fungus
New.ReferenceOTU2238	5	0	0	0	0	0	0	0	0	0	0	0	0	0	0	0	0	0	0	0	0	0	0	0	0	0	0	0	0	0	Fungi; unidentified; unidentified; unidentified; unidentified; unidentified; uncultured_fungus
New.ReferenceOTU2254	0	0	1	0	0	0	0	0	0	0	0	0	0	0	0	0	0	0	0	0	0	0	0	0	0	0	0	0	0	0	Fungi; unidentified; unidentified; unidentified; unidentified; unidentified; uncultured_fungus
New.ReferenceOTU2301	0	0	0	0	0	0	0	0	0	0	0	0	0	0	0	0	0	0	0	0	1	0	0	0	0	0	0	0	0	0	Fungi; unidentified; unidentified; unidentified; unidentified; unidentified; uncultured_fungus
New.ReferenceOTU2335	0	0	0	1	0	0	0	0	0	0	0	0	0	0	0	0	0	0	0	0	0	0	0	0	0	0	0	0	0	0	Fungi; unidentified; unidentified; unidentified; unidentified; unidentified; uncultured_fungus
New.ReferenceOTU2371	1	0	0	0	0	0	0	0	0	0	0	0	0	0	0	1	0	0	0	0	0	0	0	0	0	0	0	0	0	0	Fungi; unidentified; unidentified; unidentified; unidentified; unidentified; uncultured_fungus
New.ReferenceOTU2385	0	0	0	0	0	0	0	0	0	0	0	0	0	0	1	0	0	0	0	0	1	0	0	0	0	0	0	0	0	0	Fungi; unidentified; unidentified; unidentified; unidentified; unidentified; uncultured_fungus
New.ReferenceOTU24	0	0	0	0	0	0	0	0	0	0	0	0	0	0	0	0	1	0	0	0	0	0	0	0	0	0	0	0	0	0	Fungi; unidentified; unidentified; unidentified; unidentified; unidentified; uncultured_fungus
New.ReferenceOTU2440	1	0	0	0	0	0	0	0	1	0	0	1	0	2	0	0	0	0	0	0	0	0	0	0	0	0	0	0	0	0	Fungi; unidentified; unidentified; unidentified; unidentified; unidentified; uncultured_fungus
New.ReferenceOTU2443	0	0	0	0	1	0	0	0	0	0	0	0	0	0	0	0	0	0	0	0	0	0	0	0	0	0	0	0	0	0	Fungi; unidentified; unidentified; unidentified; unidentified; unidentified; uncultured_fungus
New.ReferenceOTU2449	2	0	0	0	0	0	1	0	0	0	0	0	0	0	0	0	0	0	0	0	0	0	0	0	0	0	0	0	0	0	Fungi; unidentified; unidentified; unidentified; unidentified; unidentified; uncultured_fungus
New.ReferenceOTU2454	0	0	0	0	0	0	0	0	0	0	0	0	0	0	0	0	0	0	0	0	0	0	0	2	0	0	0	0	0	0	Fungi; unidentified; unidentified; unidentified; unidentified; unidentified; uncultured_fungus
New.ReferenceOTU246	0	0	0	0	0	0	0	0	10	0	0	0	0	0	0	0	0	0	0	0	0	0	0	0	0	0	0	0	0	0	Fungi; unidentified; unidentified; unidentified; unidentified; unidentified; uncultured_fungus
New.ReferenceOTU2465	0	0	1	0	0	0	0	0	0	0	0	0	0	0	0	0	0	0	0	0	0	0	0	0	0	0	0	0	0	0	Fungi; unidentified; unidentified; unidentified; unidentified; unidentified; uncultured_fungus
New.ReferenceOTU2486	0	0	0	0	0	0	0	0	0	1	0	0	0	0	0	0	0	0	0	0	0	0	0	0	0	0	0	0	0	0	Fungi; unidentified; unidentified; unidentified; unidentified; unidentified; uncultured_fungus
New.ReferenceOTU2501	0	0	0	0	0	0	0	0	0	0	0	0	0	0	0	0	0	0	0	0	0	0	0	0	0	0	0	0	2	0	Fungi; unidentified; unidentified; unidentified; unidentified; unidentified; uncultured_fungus
New.ReferenceOTU2527	0	0	0	0	0	0	0	0	0	0	0	0	0	0	0	0	0	0	0	0	0	0	0	0	5	0	0	0	0	0	Fungi; unidentified; unidentified; unidentified; unidentified; unidentified; uncultured_fungus
New.ReferenceOTU2578	0	0	0	0	3	0	0	0	0	0	0	0	0	0	0	0	0	0	0	0	0	0	0	0	0	0	0	0	0	0	Fungi; unidentified; unidentified; unidentified; unidentified; unidentified; uncultured_fungus
New.ReferenceOTU2585	0	0	0	0	0	0	0	1	0	0	0	0	0	0	0	0	0	0	0	0	0	0	0	0	0	0	0	0	0	0	Fungi; unidentified; unidentified; unidentified; unidentified; unidentified; uncultured_fungus
New.ReferenceOTU259	0	0	0	0	0	0	1	0	0	0	0	0	0	0	0	0	0	0	0	0	0	0	0	0	0	0	0	0	0	0	Fungi; unidentified; unidentified; unidentified; unidentified; unidentified; uncultured_fungus
New.ReferenceOTU2597	0	0	1	0	0	0	0	0	0	0	0	0	0	0	0	0	0	0	0	0	0	0	0	0	0	0	0	0	0	0	Fungi; unidentified; unidentified; unidentified; unidentified; unidentified; uncultured_fungus
New.ReferenceOTU2680	0	0	0	1	0	0	0	1	0	2	0	0	0	0	0	0	1	0	0	0	0	0	0	0	0	0	0	0	0	0	Fungi; unidentified; unidentified; unidentified; unidentified; unidentified; uncultured_fungus
New.ReferenceOTU2786	0	0	0	0	0	0	0	0	0	0	0	0	0	0	0	1	0	0	0	0	0	0	0	0	0	0	0	0	0	0	Fungi; unidentified; unidentified; unidentified; unidentified; unidentified; uncultured_fungus
New.ReferenceOTU2797	9	2	0	0	0	4	13	4	0	0	0	1	0	1	1	0	0	0	1	0	0	0	0	0	0	1	0	0	0	0	Fungi; unidentified; unidentified; unidentified; unidentified; unidentified; uncultured_fungus
New.ReferenceOTU2815	0	0	0	0	0	0	0	0	0	0	0	1	0	0	0	0	0	0	0	0	0	0	0	0	0	0	0	0	0	0	Fungi; unidentified; unidentified; unidentified; unidentified; unidentified; uncultured_fungus
New.ReferenceOTU2862	0	0	0	0	1	0	0	0	0	0	0	0	0	0	0	0	0	0	0	0	0	0	0	0	0	0	0	0	0	0	Fungi; unidentified; unidentified; unidentified; unidentified; unidentified; uncultured_fungus
New.ReferenceOTU2930	3	0	0	0	0	0	0	0	0	0	0	0	0	0	0	0	0	0	0	0	0	0	0	0	0	0	0	0	0	0	Fungi; unidentified; unidentified; unidentified; unidentified; unidentified; uncultured_fungus
New.ReferenceOTU297	0	0	0	0	0	0	0	0	0	0	0	0	0	0	1	0	0	0	0	0	0	0	0	0	0	0	0	0	0	0	Fungi; unidentified; unidentified; unidentified; unidentified; unidentified; uncultured_fungus
New.ReferenceOTU3012	0	0	0	0	0	0	0	0	0	0	0	0	0	0	0	0	0	0	0	1	0	0	0	0	0	0	0	0	0	0	Fungi; unidentified; unidentified; unidentified; unidentified; unidentified; uncultured_fungus
New.ReferenceOTU3026	0	0	0	0	0	0	0	8	0	0	0	0	0	0	0	0	0	0	0	0	0	0	0	0	0	0	0	0	0	0	Fungi; unidentified; unidentified; unidentified; unidentified; unidentified; uncultured_fungus
New.ReferenceOTU3058	0	0	0	0	0	0	0	0	0	0	0	0	0	1	0	0	0	0	0	0	0	0	0	0	0	0	0	0	0	0	Fungi; unidentified; unidentified; unidentified; unidentified; unidentified; uncultured_fungus
New.ReferenceOTU3065	0	0	0	1	0	0	0	0	0	0	0	0	0	0	0	0	0	0	0	0	0	0	0	0	0	0	0	0	0	0	Fungi; unidentified; unidentified; unidentified; unidentified; unidentified; uncultured_fungus
New.ReferenceOTU311	0	0	4	0	0	0	0	2	0	0	0	0	0	0	0	0	0	0	0	0	0	0	0	0	0	0	0	0	0	0	Fungi; unidentified; unidentified; unidentified; unidentified; unidentified; uncultured_fungus
New.ReferenceOTU313	0	0	0	0	0	0	0	0	0	0	0	0	0	0	0	0	0	0	0	1	0	0	0	0	0	0	0	0	0	0	Fungi; unidentified; unidentified; unidentified; unidentified; unidentified; uncultured_fungus
New.ReferenceOTU314	0	0	0	0	0	0	0	0	0	0	0	0	0	0	0	0	0	0	0	0	0	0	0	0	0	0	0	1	0	0	Fungi; unidentified; unidentified; unidentified; unidentified; unidentified; uncultured_fungus
New.ReferenceOTU3173	0	0	0	0	0	0	0	0	0	1	0	0	0	0	0	0	0	0	0	0	0	0	0	0	0	0	0	0	0	0	Fungi; unidentified; unidentified; unidentified; unidentified; unidentified; uncultured_fungus
New.ReferenceOTU319	0	0	0	0	0	0	0	0	0	0	0	0	0	0	0	0	0	0	0	0	0	0	0	0	0	0	0	0	0	2	Fungi; unidentified; unidentified; unidentified; unidentified; unidentified; uncultured_fungus
New.ReferenceOTU3191	4	0	1	0	0	0	0	0	0	0	0	0	1	0	0	0	0	9	0	1	0	5	0	0	0	0	0	0	0	0	Fungi; unidentified; unidentified; unidentified; unidentified; unidentified; uncultured_fungus
New.ReferenceOTU3270	0	0	3	0	0	0	0	0	0	0	0	0	0	0	0	0	0	0	0	0	0	0	0	0	0	0	0	0	0	0	Fungi; unidentified; unidentified; unidentified; unidentified; unidentified; uncultured_fungus
New.ReferenceOTU3298	1	0	0	0	0	0	0	0	0	0	0	0	0	0	0	0	0	0	0	0	0	0	0	0	0	0	0	0	0	0	Fungi; unidentified; unidentified; unidentified; unidentified; unidentified; uncultured_fungus
New.ReferenceOTU33	0	0	0	0	0	0	0	0	0	0	0	0	0	0	0	0	0	0	0	0	0	0	0	0	0	0	0	0	0	2	Fungi; unidentified; unidentified; unidentified; unidentified; unidentified; uncultured_fungus
New.ReferenceOTU3308	0	0	0	0	0	0	0	0	0	0	0	0	0	0	1	0	0	0	0	0	0	0	0	0	0	0	0	0	0	0	Fungi; unidentified; unidentified; unidentified; unidentified; unidentified; uncultured_fungus
New.ReferenceOTU3391	8	1	9	3	0	0	0	0	0	0	0	0	0	0	3	0	1	4	3	1	0	2	2	0	0	0	0	0	2	1	Fungi; unidentified; unidentified; unidentified; unidentified; unidentified; uncultured_fungus
New.ReferenceOTU3400	0	0	0	0	2	0	0	0	0	0	0	0	0	0	0	0	0	0	0	0	0	0	0	0	0	0	0	0	0	0	Fungi; unidentified; unidentified; unidentified; unidentified; unidentified; uncultured_fungus
New.ReferenceOTU3524	0	2	0	0	0	0	0	0	0	0	0	0	0	0	0	0	0	0	0	0	0	0	0	0	0	0	0	0	0	0	Fungi; unidentified; unidentified; unidentified; unidentified; unidentified; uncultured_fungus
New.ReferenceOTU3537	0	0	0	0	0	0	0	0	0	0	0	0	0	0	0	0	0	0	1	0	0	0	0	0	0	0	0	0	0	0	Fungi; unidentified; unidentified; unidentified; unidentified; unidentified; uncultured_fungus
New.ReferenceOTU3570	1	0	0	0	0	0	0	0	0	0	0	0	0	0	0	0	0	0	0	0	0	0	0	0	0	0	0	0	0	0	Fungi; unidentified; unidentified; unidentified; unidentified; unidentified; uncultured_fungus
New.ReferenceOTU3613	0	0	0	0	0	0	0	0	0	0	0	0	0	0	0	1	0	0	0	0	0	0	0	0	0	0	0	0	0	0	Fungi; unidentified; unidentified; unidentified; unidentified; unidentified; uncultured_fungus
New.ReferenceOTU3646	0	0	0	0	0	0	0	0	0	0	0	0	0	1	0	0	0	0	0	0	0	0	0	0	0	0	0	0	0	0	Fungi; unidentified; unidentified; unidentified; unidentified; unidentified; uncultured_fungus
New.ReferenceOTU3683	0	0	0	0	0	0	0	0	0	0	0	0	0	0	0	2	0	0	0	0	0	0	0	0	0	0	0	0	0	0	Fungi; unidentified; unidentified; unidentified; unidentified; unidentified; uncultured_fungus
New.ReferenceOTU3695	0	0	0	0	0	0	0	0	0	0	0	0	0	0	0	0	0	1	0	0	0	0	0	0	0	0	0	0	0	0	Fungi; unidentified; unidentified; unidentified; unidentified; unidentified; uncultured_fungus
New.ReferenceOTU3710	0	0	0	0	1	0	0	0	0	0	0	0	0	0	0	0	0	0	0	0	0	0	0	0	0	0	0	0	0	0	Fungi; unidentified; unidentified; unidentified; unidentified; unidentified; uncultured_fungus
New.ReferenceOTU3714	0	0	0	0	0	0	4	0	0	0	0	0	0	0	0	0	0	0	0	0	0	0	0	0	0	0	0	0	0	0	Fungi; unidentified; unidentified; unidentified; unidentified; unidentified; uncultured_fungus
New.ReferenceOTU3782	0	0	0	0	0	0	0	0	0	0	0	0	0	0	0	0	1	0	0	0	0	0	0	0	0	0	0	0	0	0	Fungi; unidentified; unidentified; unidentified; unidentified; unidentified; uncultured_fungus
New.ReferenceOTU3789	0	0	0	0	0	0	0	1	0	0	0	0	0	0	0	0	0	0	0	0	0	0	0	0	0	0	0	0	0	0	Fungi; unidentified; unidentified; unidentified; unidentified; unidentified; uncultured_fungus
New.ReferenceOTU3794	0	1	0	0	0	0	0	0	0	2	0	0	0	5	0	0	2	0	0	0	0	0	0	1	0	0	0	0	0	0	Fungi; unidentified; unidentified; unidentified; unidentified; unidentified; uncultured_fungus
New.ReferenceOTU3795	0	0	0	0	0	0	0	0	0	0	0	0	0	0	0	0	0	0	0	0	0	0	1	0	0	0	1	0	0	0	Fungi; unidentified; unidentified; unidentified; unidentified; unidentified; uncultured_fungus
New.ReferenceOTU3806	0	0	0	0	0	0	0	0	0	1	1	0	0	0	0	0	0	0	0	0	0	0	0	0	0	0	0	0	0	0	Fungi; unidentified; unidentified; unidentified; unidentified; unidentified; uncultured_fungus
New.ReferenceOTU3824	3	0	0	0	0	0	0	0	0	0	0	0	0	0	0	0	0	0	0	0	0	0	0	0	0	0	0	0	0	0	Fungi; unidentified; unidentified; unidentified; unidentified; unidentified; uncultured_fungus
New.ReferenceOTU383	0	0	0	0	0	0	0	0	0	0	0	0	0	3	0	0	0	0	0	0	0	0	0	0	0	0	0	0	0	0	Fungi; unidentified; unidentified; unidentified; unidentified; unidentified; uncultured_fungus
New.ReferenceOTU3877	0	0	0	0	0	0	0	0	0	0	0	0	0	0	0	0	0	0	0	0	0	0	0	0	0	0	0	1	0	0	Fungi; unidentified; unidentified; unidentified; unidentified; unidentified; uncultured_fungus
New.ReferenceOTU3958	0	0	0	0	0	0	0	0	0	0	0	0	0	0	0	0	1	0	0	0	0	0	0	0	0	0	0	0	0	0	Fungi; unidentified; unidentified; unidentified; unidentified; unidentified; uncultured_fungus
New.ReferenceOTU3981	0	0	0	0	0	0	0	0	0	0	0	0	0	0	0	0	0	0	0	0	0	0	0	0	1	0	0	2	0	4	Fungi; unidentified; unidentified; unidentified; unidentified; unidentified; uncultured_fungus
New.ReferenceOTU4002	0	0	0	0	0	0	0	0	0	0	0	1	0	0	0	0	0	0	0	0	0	0	0	0	0	0	0	0	0	0	Fungi; unidentified; unidentified; unidentified; unidentified; unidentified; uncultured_fungus
New.ReferenceOTU4006	0	0	0	0	1	0	0	0	0	0	0	0	0	0	0	0	0	0	0	0	0	0	0	0	0	0	0	0	0	0	Fungi; unidentified; unidentified; unidentified; unidentified; unidentified; uncultured_fungus
New.ReferenceOTU4091	0	0	0	1	0	0	0	0	0	0	0	0	0	0	0	0	0	0	0	0	0	0	0	0	0	0	0	0	0	0	Fungi; unidentified; unidentified; unidentified; unidentified; unidentified; uncultured_fungus
New.ReferenceOTU4095	0	0	0	0	0	0	0	0	0	0	0	0	0	0	0	0	0	0	0	0	1	0	0	0	0	0	0	1	0	0	Fungi; unidentified; unidentified; unidentified; unidentified; unidentified; uncultured_fungus
New.ReferenceOTU4099	0	0	0	0	0	0	0	0	0	0	3	0	0	0	0	0	0	0	0	0	0	0	0	0	0	0	0	0	0	0	Fungi; unidentified; unidentified; unidentified; unidentified; unidentified; uncultured_fungus
New.ReferenceOTU4111	0	0	0	0	0	2	0	0	0	0	0	0	4	1	7	1	0	1	7	0	4	27	30	0	8	0	0	0	0	5	Fungi; unidentified; unidentified; unidentified; unidentified; unidentified; uncultured_fungus
New.ReferenceOTU4204	0	0	0	0	0	0	0	0	0	0	0	0	0	0	1	0	0	0	0	0	0	0	0	0	0	0	0	0	0	0	Fungi; unidentified; unidentified; unidentified; unidentified; unidentified; uncultured_fungus
New.ReferenceOTU4222	0	0	0	0	0	0	0	0	0	0	0	0	0	0	0	0	0	0	2	0	0	0	0	0	1	0	0	0	0	0	Fungi; unidentified; unidentified; unidentified; unidentified; unidentified; uncultured_fungus
New.ReferenceOTU4257	0	0	0	0	0	0	0	0	0	0	0	0	0	0	0	0	0	3	0	0	0	0	0	0	0	0	0	0	0	0	Fungi; unidentified; unidentified; unidentified; unidentified; unidentified; uncultured_fungus
New.ReferenceOTU4282	0	0	0	0	0	0	0	0	0	0	0	0	0	0	0	0	0	0	0	0	0	0	0	0	0	0	0	1	0	0	Fungi; unidentified; unidentified; unidentified; unidentified; unidentified; uncultured_fungus
New.ReferenceOTU4348	0	0	0	0	0	0	0	0	0	0	0	0	0	0	0	0	0	0	0	0	0	0	0	0	1	0	0	0	0	0	Fungi; unidentified; unidentified; unidentified; unidentified; unidentified; uncultured_fungus
New.ReferenceOTU4353	0	0	0	0	0	0	0	0	0	0	0	0	0	0	0	0	0	0	0	0	0	0	0	2	0	0	0	0	0	0	Fungi; unidentified; unidentified; unidentified; unidentified; unidentified; uncultured_fungus
New.ReferenceOTU4359	0	0	0	0	0	0	0	0	0	0	0	0	0	0	0	2	0	0	0	0	0	0	1	0	0	0	0	0	0	0	Fungi; unidentified; unidentified; unidentified; unidentified; unidentified; uncultured_fungus
New.ReferenceOTU4388	0	0	0	0	0	0	0	0	0	0	0	0	0	0	0	0	0	0	0	0	1	0	0	0	0	0	0	0	0	0	Fungi; unidentified; unidentified; unidentified; unidentified; unidentified; uncultured_fungus
New.ReferenceOTU4440	0	0	0	0	0	0	0	0	0	0	0	0	0	0	0	0	0	0	1	0	0	0	0	0	0	0	0	0	0	0	Fungi; unidentified; unidentified; unidentified; unidentified; unidentified; uncultured_fungus
New.ReferenceOTU4473	0	0	0	0	0	0	1	1	0	0	0	0	0	0	0	0	0	0	0	0	0	0	1	0	0	0	0	0	0	0	Fungi; unidentified; unidentified; unidentified; unidentified; unidentified; uncultured_fungus
New.ReferenceOTU4480	0	0	0	3	0	0	0	0	0	0	0	0	0	0	0	0	0	0	0	0	0	0	0	0	0	0	0	0	0	0	Fungi; unidentified; unidentified; unidentified; unidentified; unidentified; uncultured_fungus
New.ReferenceOTU4599	0	0	0	0	0	0	0	1	0	0	0	0	0	0	0	0	0	0	0	0	0	0	0	0	0	0	0	0	0	0	Fungi; unidentified; unidentified; unidentified; unidentified; unidentified; uncultured_fungus
New.ReferenceOTU4616	0	0	0	1	0	0	0	0	0	0	0	0	0	0	0	0	0	0	0	0	0	0	0	0	0	0	0	0	0	0	Fungi; unidentified; unidentified; unidentified; unidentified; unidentified; uncultured_fungus
New.ReferenceOTU4665	0	0	0	0	0	0	0	0	0	0	0	0	0	0	0	0	0	0	0	0	0	0	0	0	0	0	0	0	2	0	Fungi; unidentified; unidentified; unidentified; unidentified; unidentified; uncultured_fungus
New.ReferenceOTU4667	0	0	0	0	0	2	0	0	0	0	0	0	0	0	0	0	0	0	0	0	0	0	0	0	0	0	0	0	0	0	Fungi; unidentified; unidentified; unidentified; unidentified; unidentified; uncultured_fungus
New.ReferenceOTU4708	0	0	0	0	0	0	0	0	0	0	0	0	0	0	0	0	0	0	0	9	0	0	0	0	0	0	0	0	0	0	Fungi; unidentified; unidentified; unidentified; unidentified; unidentified; uncultured_fungus
New.ReferenceOTU4712	0	0	0	0	0	0	0	0	0	0	0	0	0	0	1	0	0	0	0	0	0	0	0	0	0	0	0	0	0	0	Fungi; unidentified; unidentified; unidentified; unidentified; unidentified; uncultured_fungus
New.ReferenceOTU4779	0	0	0	0	0	0	0	0	0	0	0	0	0	0	0	0	0	0	0	0	0	0	0	0	0	0	0	0	2	0	Fungi; unidentified; unidentified; unidentified; unidentified; unidentified; uncultured_fungus
New.ReferenceOTU4783	0	0	0	0	0	0	0	0	0	0	0	0	0	0	0	0	0	0	0	0	0	0	0	0	0	0	0	0	1	0	Fungi; unidentified; unidentified; unidentified; unidentified; unidentified; uncultured_fungus
New.ReferenceOTU4805	0	0	0	0	0	0	0	0	0	0	0	0	0	0	0	0	0	0	2	0	0	0	0	0	0	0	0	0	0	0	Fungi; unidentified; unidentified; unidentified; unidentified; unidentified; uncultured_fungus
New.ReferenceOTU4967	0	0	0	0	0	1	0	0	0	0	0	0	0	0	0	0	0	0	0	0	0	0	0	0	0	0	0	0	0	0	Fungi; unidentified; unidentified; unidentified; unidentified; unidentified; uncultured_fungus
New.ReferenceOTU4979	0	0	0	0	0	0	0	0	0	0	1	0	0	0	0	0	0	0	0	0	0	0	0	0	0	0	0	0	0	0	Fungi; unidentified; unidentified; unidentified; unidentified; unidentified; uncultured_fungus
New.ReferenceOTU506	0	0	0	0	0	0	0	0	0	0	0	0	0	0	0	0	0	5	0	0	0	0	0	0	0	0	0	0	0	0	Fungi; unidentified; unidentified; unidentified; unidentified; unidentified; uncultured_fungus
New.ReferenceOTU5130	0	0	0	0	0	0	0	0	1	3	0	0	0	0	0	0	0	0	0	0	0	0	0	0	0	0	0	0	0	0	Fungi; unidentified; unidentified; unidentified; unidentified; unidentified; uncultured_fungus
New.ReferenceOTU5161	1	0	0	0	0	0	0	0	0	0	0	0	0	0	0	0	0	0	0	0	0	0	0	0	0	0	0	0	0	1	Fungi; unidentified; unidentified; unidentified; unidentified; unidentified; uncultured_fungus
New.ReferenceOTU5218	0	0	0	0	0	0	0	0	0	0	0	1	0	0	0	0	0	0	0	0	0	0	0	0	0	0	0	0	0	0	Fungi; unidentified; unidentified; unidentified; unidentified; unidentified; uncultured_fungus
New.ReferenceOTU5249	0	0	0	0	0	0	0	0	0	0	0	0	0	0	0	0	0	0	0	1	0	0	0	0	0	0	0	0	0	0	Fungi; unidentified; unidentified; unidentified; unidentified; unidentified; uncultured_fungus
New.ReferenceOTU5283	0	0	0	0	3	0	0	0	1	0	0	0	2	0	0	0	0	0	0	0	0	0	0	0	0	0	0	0	0	0	Fungi; unidentified; unidentified; unidentified; unidentified; unidentified; uncultured_fungus
New.ReferenceOTU5319	0	0	0	0	0	0	0	0	0	0	0	0	0	0	0	0	2	0	0	0	0	0	0	0	0	0	0	0	0	0	Fungi; unidentified; unidentified; unidentified; unidentified; unidentified; uncultured_fungus
New.ReferenceOTU5342	0	0	4	0	0	0	0	0	0	0	0	0	0	0	1	3	2	0	0	0	0	1	0	0	0	0	0	0	0	0	Fungi; unidentified; unidentified; unidentified; unidentified; unidentified; uncultured_fungus
New.ReferenceOTU5349	0	0	0	0	0	0	0	0	0	1	1	0	0	3	0	1	0	0	0	0	0	0	0	1	0	0	0	0	0	0	Fungi; unidentified; unidentified; unidentified; unidentified; unidentified; uncultured_fungus

New.ReferenceOTU5350	0	11	0	0	0	0	0	0	0	0	0	0	0	0	0	0	0	0	0	0	0	0	0	0	0	0	0	0	0	0	Fungi; unidentified; unidentified; unidentified; unidentified; unidentified; uncultured_fungus
New.ReferenceOTU540	0	0	0	0	0	0	0	0	0	0	0	0	0	0	1	0	0	0	0	0	0	0	0	0	0	0	0	0	0	0	Fungi; unidentified; unidentified; unidentified; unidentified; unidentified; uncultured_fungus
New.ReferenceOTU558	0	0	0	0	0	0	0	0	0	1	0	0	0	2	0	1	1	0	0	0	0	0	0	0	0	0	0	0	0	0	Fungi; unidentified; unidentified; unidentified; unidentified; unidentified; uncultured_fungus
New.ReferenceOTU5600	0	0	0	0	0	1	0	0	0	0	0	0	0	0	0	0	0	0	0	0	0	0	0	0	0	0	0	0	0	0	Fungi; unidentified; unidentified; unidentified; unidentified; unidentified; uncultured_fungus
New.ReferenceOTU5646	0	0	0	0	0	0	0	0	0	0	0	0	0	0	0	0	0	3	0	0	0	0	0	0	0	0	0	0	0	0	Fungi; unidentified; unidentified; unidentified; unidentified; unidentified; uncultured_fungus
New.ReferenceOTU5674	0	1	0	2	0	0	0	0	0	0	0	0	0	0	0	0	0	0	0	0	0	0	0	0	0	0	0	0	0	0	Fungi; unidentified; unidentified; unidentified; unidentified; unidentified; uncultured_fungus
New.ReferenceOTU5706	0	0	0	0	0	1	1	0	0	0	0	0	1	0	0	0	0	0	0	0	0	0	0	0	0	0	0	0	0	0	Fungi; unidentified; unidentified; unidentified; unidentified; unidentified; uncultured_fungus
New.ReferenceOTU5711	0	0	1	1	0	0	0	0	0	1	0	0	0	0	0	0	1	0	0	0	0	0	0	0	1	0	0	0	2	0	Fungi; unidentified; unidentified; unidentified; unidentified; unidentified; uncultured_fungus
New.ReferenceOTU5751	0	0	0	0	0	0	0	0	0	0	3	0	0	0	0	0	0	0	0	0	0	0	0	0	0	0	0	0	0	0	Fungi; unidentified; unidentified; unidentified; unidentified; unidentified; uncultured_fungus
New.ReferenceOTU5755	0	0	0	0	0	0	0	0	0	0	0	0	0	0	0	0	0	0	0	1	0	0	0	0	0	0	0	0	0	0	Fungi; unidentified; unidentified; unidentified; unidentified; unidentified; uncultured_fungus
New.ReferenceOTU5790	0	0	0	0	0	0	0	0	0	0	0	0	0	0	0	0	0	1	11	1	0	0	0	0	0	0	0	0	0	0	Fungi; unidentified; unidentified; unidentified; unidentified; unidentified; uncultured_fungus
New.ReferenceOTU5800	0	3	0	0	0	0	0	0	0	0	0	0	0	0	0	0	0	0	0	0	0	0	0	0	0	0	0	0	0	0	Fungi; unidentified; unidentified; unidentified; unidentified; unidentified; uncultured_fungus
New.ReferenceOTU5812	0	0	0	0	0	0	0	0	0	0	0	0	0	0	0	0	0	0	0	0	0	2	0	0	0	0	0	0	0	0	Fungi; unidentified; unidentified; unidentified; unidentified; unidentified; uncultured_fungus
New.ReferenceOTU5887	0	0	0	0	0	0	0	0	0	0	0	0	0	0	2	0	0	0	0	0	0	0	0	0	0	0	0	0	0	0	Fungi; unidentified; unidentified; unidentified; unidentified; unidentified; uncultured_fungus
New.ReferenceOTU5901	0	0	0	0	0	0	0	0	0	0	0	0	0	0	0	1	0	0	0	0	0	0	0	0	0	0	0	0	0	0	Fungi; unidentified; unidentified; unidentified; unidentified; unidentified; uncultured_fungus
New.ReferenceOTU5922	0	0	0	0	0	0	0	0	0	0	0	0	0	0	0	0	0	0	0	0	0	0	0	0	0	0	0	0	0	6	Fungi; unidentified; unidentified; unidentified; unidentified; unidentified; uncultured_fungus
New.ReferenceOTU5941	0	0	0	0	0	0	0	0	0	0	0	0	0	0	0	0	0	0	0	0	0	1	0	0	0	0	0	0	0	0	Fungi; unidentified; unidentified; unidentified; unidentified; unidentified; uncultured_fungus
New.ReferenceOTU6067	0	0	0	1	0	0	0	0	0	0	0	0	0	0	0	0	0	0	0	0	0	0	0	0	0	0	0	0	0	0	Fungi; unidentified; unidentified; unidentified; unidentified; unidentified; uncultured_fungus
New.ReferenceOTU6090	14	0	0	0	0	0	0	0	0	0	0	0	0	0	0	0	0	0	0	0	0	0	0	2	0	0	0	0	0	0	Fungi; unidentified; unidentified; unidentified; unidentified; unidentified; uncultured_fungus
New.ReferenceOTU6163	0	0	0	0	0	0	0	0	0	1	0	0	0	0	0	0	0	0	0	0	0	0	0	1	0	0	0	0	0	0	Fungi; unidentified; unidentified; unidentified; unidentified; unidentified; uncultured_fungus
New.ReferenceOTU6171	0	0	0	0	3	0	0	0	0	0	0	0	0	0	0	0	0	0	0	0	0	0	0	0	0	0	0	0	0	0	Fungi; unidentified; unidentified; unidentified; unidentified; unidentified; uncultured_fungus
New.ReferenceOTU6179	0	0	0	0	1	0	0	0	0	0	0	0	0	0	0	0	0	0	0	0	0	0	0	0	0	0	0	0	0	0	Fungi; unidentified; unidentified; unidentified; unidentified; unidentified; uncultured_fungus
New.ReferenceOTU6260	0	0	0	0	2	0	0	0	0	0	0	0	0	0	0	0	0	0	0	0	0	0	0	0	0	0	0	0	0	0	Fungi; unidentified; unidentified; unidentified; unidentified; unidentified; uncultured_fungus
New.ReferenceOTU6284	0	0	0	0	0	0	0	0	0	0	1	0	0	0	0	0	0	0	0	1	0	0	0	0	0	0	1	0	0	1	Fungi; unidentified; unidentified; unidentified; unidentified; unidentified; uncultured_fungus
New.ReferenceOTU6341	0	0	0	0	2	0	0	0	0	0	0	0	0	0	0	0	0	0	0	0	0	0	0	0	0	0	0	0	0	0	Fungi; unidentified; unidentified; unidentified; unidentified; unidentified; uncultured_fungus
New.ReferenceOTU6348	0	0	0	1	0	0	0	0	0	0	0	0	2	2	0	0	0	0	0	0	0	0	0	0	0	0	0	0	1	0	Fungi; unidentified; unidentified; unidentified; unidentified; unidentified; uncultured_fungus
New.ReferenceOTU6357	0	0	0	0	0	0	0	6	0	0	0	0	0	2	0	0	2	0	0	0	0	0	0	0	0	0	0	0	0	0	Fungi; unidentified; unidentified; unidentified; unidentified; unidentified; uncultured_fungus
New.ReferenceOTU6369	0	0	0	0	0	0	0	0	0	0	0	0	11	0	0	0	0	1	0	0	0	0	0	1	0	0	0	0	0	0	Fungi; unidentified; unidentified; unidentified; unidentified; unidentified; uncultured_fungus
New.ReferenceOTU6370	0	0	0	0	1	0	1	0	0	0	0	0	0	0	0	0	0	0	0	0	0	0	0	0	0	0	0	0	0	0	Fungi; unidentified; unidentified; unidentified; unidentified; unidentified; uncultured_fungus
New.ReferenceOTU6456	0	2	0	0	0	0	0	0	0	0	0	0	0	0	0	0	0	0	0	0	0	0	0	0	0	0	0	0	0	0	Fungi; unidentified; unidentified; unidentified; unidentified; unidentified; uncultured_fungus
New.ReferenceOTU647	0	0	0	0	0	0	0	0	0	0	0	0	0	0	0	0	0	0	0	0	0	0	0	0	0	0	1	0	0	0	Fungi; unidentified; unidentified; unidentified; unidentified; unidentified; uncultured_fungus
New.ReferenceOTU6524	0	0	0	0	0	0	0	0	0	0	0	0	0	0	0	0	0	0	0	0	0	1	0	0	0	0	0	0	0	0	Fungi; unidentified; unidentified; unidentified; unidentified; unidentified; uncultured_fungus
New.ReferenceOTU6526	1	0	0	0	0	0	0	0	0	0	0	0	0	0	0	0	0	0	0	0	0	0	0	0	0	0	0	0	0	0	Fungi; unidentified; unidentified; unidentified; unidentified; unidentified; uncultured_fungus
New.ReferenceOTU654	0	0	0	0	0	0	0	0	0	0	0	0	0	0	0	0	0	0	0	1	0	0	0	0	0	0	0	0	0	0	Fungi; unidentified; unidentified; unidentified; unidentified; unidentified; uncultured_fungus
New.ReferenceOTU6554	0	0	0	0	0	0	0	0	0	0	0	0	0	0	0	0	0	0	0	0	0	0	0	0	1	0	0	0	0	0	Fungi; unidentified; unidentified; unidentified; unidentified; unidentified; uncultured_fungus
New.ReferenceOTU6558	0	0	0	0	0	0	0	0	1	0	0	0	0	0	0	0	0	0	0	0	0	2	13	0	4	0	0	1	0	0	Fungi; unidentified; unidentified; unidentified; unidentified; unidentified; uncultured_fungus
New.ReferenceOTU6685	0	0	0	0	0	0	0	0	0	0	0	0	0	0	0	0	0	0	0	0	0	0	0	0	0	0	0	0	0	1	Fungi; unidentified; unidentified; unidentified; unidentified; unidentified; uncultured_fungus
New.ReferenceOTU6706	0	0	0	0	0	0	0	0	0	0	0	0	0	0	1	0	0	0	0	0	0	0	0	0	0	0	0	0	0	0	Fungi; unidentified; unidentified; unidentified; unidentified; unidentified; uncultured_fungus
New.ReferenceOTU6712	0	0	0	0	2	0	0	0	0	0	0	0	0	0	0	0	0	0	0	0	0	0	0	0	0	0	0	0	0	0	Fungi; unidentified; unidentified; unidentified; unidentified; unidentified; uncultured_fungus
New.ReferenceOTU6725	0	0	0	0	0	0	0	0	0	0	0	0	0	0	0	0	2	0	0	0	0	0	0	0	0	0	0	0	0	0	Fungi; unidentified; unidentified; unidentified; unidentified; unidentified; uncultured_fungus
New.ReferenceOTU6779	0	0	0	0	0	0	0	0	0	0	0	0	0	0	0	0	0	0	0	0	0	1	1	0	0	0	0	0	0	2	Fungi; unidentified; unidentified; unidentified; unidentified; unidentified; uncultured_fungus
New.ReferenceOTU6822	0	4	0	0	0	0	2	0	0	4	1	0	0	4	0	1	1	3	9	13	0	0	1	0	0	0	11	0	0	0	Fungi; unidentified; unidentified; unidentified; unidentified; unidentified; uncultured_fungus
New.ReferenceOTU6831	0	0	0	0	0	0	0	0	0	0	0	0	0	0	0	0	0	0	0	0	0	0	0	0	0	1	0	0	2	0	Fungi; unidentified; unidentified; unidentified; unidentified; unidentified; uncultured_fungus
New.ReferenceOTU6833	0	0	0	0	0	0	0	9	0	0	0	0	0	0	12	0	1	0	0	0	4	6	0	0	0	0	1	2	0	3	Fungi; unidentified; unidentified; unidentified; unidentified; unidentified; uncultured_fungus
New.ReferenceOTU6848	0	0	0	0	0	0	0	0	0	0	0	0	0	3	0	0	0	0	0	0	0	0	0	0	0	0	0	0	0	0	Fungi; unidentified; unidentified; unidentified; unidentified; unidentified; uncultured_fungus
New.ReferenceOTU6854	0	0	0	0	0	0	0	0	0	0	0	0	0	0	0	0	0	0	1	0	0	0	0	0	0	0	0	0	0	0	Fungi; unidentified; unidentified; unidentified; unidentified; unidentified; uncultured_fungus
New.ReferenceOTU6859	0	0	0	0	0	0	0	0	0	0	0	0	0	0	0	0	1	0	0	0	0	1	0	0	2	0	1	0	0	0	Fungi; unidentified; unidentified; unidentified; unidentified; unidentified; uncultured_fungus
New.ReferenceOTU6860	0	0	0	0	1	0	0	0	0	0	0	0	0	0	0	0	0	0	0	0	0	0	0	0	0	0	0	0	0	0	Fungi; unidentified; unidentified; unidentified; unidentified; unidentified; uncultured_fungus
New.ReferenceOTU6879	0	0	0	0	0	0	0	0	0	0	0	0	0	0	0	0	0	0	0	0	0	0	0	1	0	0	0	0	0	0	Fungi; unidentified; unidentified; unidentified; unidentified; unidentified; uncultured_fungus
New.ReferenceOTU6891	0	0	0	0	0	0	0	0	0	0	0	0	0	0	0	0	0	0	0	0	1	0	0	0	0	0	0	0	0	0	Fungi; unidentified; unidentified; unidentified; unidentified; unidentified; uncultured_fungus
New.ReferenceOTU690	0	0	0	0	0	0	0	0	0	0	0	0	0	0	0	0	0	0	0	0	1	0	0	0	0	0	0	0	0	0	Fungi; unidentified; unidentified; unidentified; unidentified; unidentified; uncultured_fungus
New.ReferenceOTU6902	1	0	0	0	0	0	0	0	0	0	0	0	0	0	0	0	0	0	0	0	0	0	0	0	0	0	0	0	0	0	Fungi; unidentified; unidentified; unidentified; unidentified; unidentified; uncultured_fungus
New.ReferenceOTU6943	0	0	0	0	0	0	0	0	0	0	0	0	0	0	0	0	0	0	0	0	0	0	0	0	0	0	0	2	0	0	Fungi; unidentified; unidentified; unidentified; unidentified; unidentified; uncultured_fungus
New.ReferenceOTU6957	0	12	0	0	0	0	0	0	0	0	0	0	0	0	0	0	0	0	0	0	0	0	0	1	0	13	0	0	0	0	Fungi; unidentified; unidentified; unidentified; unidentified; unidentified; uncultured_fungus
New.ReferenceOTU6963	0	0	0	0	0	0	0	0	0	0	0	0	0	0	0	0	0	0	0	0	0	0	0	3	0	0	0	0	0	0	Fungi; unidentified; unidentified; unidentified; unidentified; unidentified; uncultured_fungus
New.ReferenceOTU7035	0	0	0	0	0	0	0	0	0	0	0	0	0	0	0	0	0	0	0	0	1	0	0	0	0	0	0	0	0	0	Fungi; unidentified; unidentified; unidentified; unidentified; unidentified; uncultured_fungus
New.ReferenceOTU7073	0	0	0	0	0	0	0	0	0	1	0	0	0	0	0	0	0	0	0	0	0	0	0	1	0	0	0	0	0	0	Fungi; unidentified; unidentified; unidentified; unidentified; unidentified; uncultured_fungus
New.ReferenceOTU7085	0	0	0	0	0	0	0	0	0	0	0	0	0	0	0	0	0	0	0	0	0	0	0	0	0	0	0	1	0	0	Fungi; unidentified; unidentified; unidentified; unidentified; unidentified; uncultured_fungus
New.ReferenceOTU7091	0	0	0	0	0	0	0	0	0	0	0	0	0	0	0	0	1	0	0	0	1	0	0	0	0	0	0	0	0	0	Fungi; unidentified; unidentified; unidentified; unidentified; unidentified; uncultured_fungus
New.ReferenceOTU7142	0	0	0	0	0	0	0	0	0	0	0	0	0	0	0	0	0	0	1	0	0	0	0	0	0	0	0	0	0	0	Fungi; unidentified; unidentified; unidentified; unidentified; unidentified; uncultured_fungus
New.ReferenceOTU7161	1	0	0	0	0	0	0	0	0	0	0	0	0	0	0	0	0	0	0	0	0	0	0	0	0	0	0	0	0	0	Fungi; unidentified; unidentified; unidentified; unidentified; unidentified; uncultured_fungus
New.ReferenceOTU7259	0	0	0	0	0	0	0	0	0	0	0	0	0	0	0	0	0	0	0	0	0	0	1	0	0	0	0	0	0	0	Fungi; unidentified; unidentified; unidentified; unidentified; unidentified; uncultured_fungus
New.ReferenceOTU7273	0	0	0	1	0	0	0	1	0	0	0	0	0	0	0	0	0	0	0	0	0	0	0	0	0	0	0	0	0	0	Fungi; unidentified; unidentified; unidentified; unidentified; unidentified; uncultured_fungus
New.ReferenceOTU7280	0	0	0	0	0	0	0	0	0	1	0	0	0	0	0	0	0	0	0	0	0	0	1	0	0	0	1	0	0	0	Fungi; unidentified; unidentified; unidentified; unidentified; unidentified; uncultured_fungus
New.ReferenceOTU7313	0	0	0	0	0	0	0	0	0	0	0	0	0	0	0	0	0	0	0	0	0	0	0	0	0	0	0	2	0	0	Fungi; unidentified; unidentified; unidentified; unidentified; unidentified; uncultured_fungus
New.ReferenceOTU7384	0	0	0	0	0	0	0	0	0	0	0	0	0	0	0	0	1	0	0	0	0	0	0	0	0	0	0	0	0	0	Fungi; unidentified; unidentified; unidentified; unidentified; unidentified; uncultured_fungus
New.ReferenceOTU7417	0	0	0	0	0	0	0	0	0	0	0	0	0	0	0	0	0	0	0	0	0	0	0	0	0	0	0	0	0	1	Fungi; unidentified; unidentified; unidentified; unidentified; unidentified; uncultured_fungus
New.ReferenceOTU7431	0	0	0	0	0	0	0	0	0	0	0	0	0	0	0	0	0	0	0	1	0	0	0	0	0	0	0	0	0	0	Fungi; unidentified; unidentified; unidentified; unidentified; unidentified; uncultured_fungus
New.ReferenceOTU7435	0	0	0	0	2	0	0	0	0	0	0	0	0	0	0	0	0	0	0	0	0	0	0	0	0	0	0	0	0	0	Fungi; unidentified; unidentified; unidentified; unidentified; unidentified; uncultured_fungus
New.ReferenceOTU7446	0	0	0	0	0	0	0	0	0	0	0	0	0	0	0	0	0	0	0	0	0	0	0	0	3	0	0	0	0	0	Fungi; unidentified; unidentified; unidentified; unidentified; unidentified; uncultured_fungus
New.ReferenceOTU7448	0	0	0	0	0	0	0	0	0	0	0	0	0	0	0	0	0	0	0	0	0	0	0	1	0	0	0	0	0	0	Fungi; unidentified; unidentified; unidentified; unidentified; unidentified; uncultured_fungus
New.ReferenceOTU7462	3	0	0	0	0	0	0	0	0	0	0	0	0	0	0	0	0	0	0	0	0	0	0	0	0	0	0	0	0	0	Fungi; unidentified; unidentified; unidentified; unidentified; unidentified; uncultured_fungus
New.ReferenceOTU7524	0	4	1	0	0	0	0	0	0	0	0	0	0	0	0	1	0	1	0	0	0	1	0	0	0	0	0	0	0	0	Fungi; unidentified; unidentified; unidentified; unidentified; unidentified; uncultured_fungus
New.ReferenceOTU7619	0	0	0	0	0	7	0	0	0	0	0	0	0	0	0	0	0	0	0	0	0	0	0	0	0	0	0	0	0	0	Fungi; unidentified; unidentified; unidentified; unidentified; unidentified; uncultured_fungus
New.ReferenceOTU7623	0	0	0	0	0	1	0	0	0	0	0	0	0	0	0	0	0	0	0	0	0	0	0	0	0	0	0	0	0	0	Fungi; unidentified; unidentified; unidentified; unidentified; unidentified; uncultured_fungus
New.ReferenceOTU7637	0	0	0	0	0	0	0	0	0	0	0	0	1	0	0	0	0	0	0	0	0	0	0	0	0	0	0	0	0	1	Fungi; unidentified; unidentified; unidentified; unidentified; unidentified; uncultured_fungus
New.ReferenceOTU7660	1	4	1	7	0	0	0	0	0	0	0	0	0	0	0	0	0	0	0	0	0	0	0	0	0	1	0	0	4	0	Fungi; unidentified; unidentified; unidentified; unidentified; unidentified; uncultured_fungus
New.ReferenceOTU7754	0	0	0	0	0	0	0	1	0	0	0	0	0	0	0	0	0	0	0	0	0	0	0	0	0	0	0	0	0	0	Fungi; unidentified; unidentified; unidentified; unidentified; unidentified; uncultured_fungus
New.ReferenceOTU7818	0	8	2	0	0	0	0	0	0	0	0	0	0	0	0	0	1	0	0	0	0	0	0	0	0	0	0	0	0	0	Fungi; unidentified; unidentified; unidentified; unidentified; unidentified; uncultured_fungus
New.ReferenceOTU7822	0	0	0	0	0	0	0	0	0	0	0	0	0	0	0	0	0	0	0	0	2	0	0	0	0	0	0	0	0	0	Fungi; unidentified; unidentified; unidentified; unidentified; unidentified; uncultured_fungus
New.ReferenceOTU790	0	0	0	0	0	0	0	0	0	0	0	0	0	0	0	0	3	0	0	0	0	0	0	0	0	0	0	0	0	0	Fungi; unidentified; unidentified; unidentified; unidentified; unidentified; uncultured_fungus
New.ReferenceOTU7900	0	0	1	1	0	0	0	0	0	0	0	1	0	0	1	0	0	0	0	0	0	0	0	0	0	0	0	0	0	0	Fungi; unidentified; unidentified; unidentified; unidentified; unidentified; uncultured_fungus
New.ReferenceOTU7927	0	0	0	0	0	0	0	0	0	0	0	0	0	0	0	0	0	0	0	0	0	0	0	0	1	0	0	0	0	0	Fungi; unidentified; unidentified; unidentified; unidentified; unidentified; uncultured_fungus
New.ReferenceOTU7929	0	0	0	0	0	0	0	0	0	0	0	0	0	0	0	0	0	0	2	0	0	0	0	0	0	0	0	0	0	0	Fungi; unidentified; unidentified; unidentified; unidentified; unidentified; uncultured_fungus
New.ReferenceOTU7956	0	0	0	0	0	0	0	0	0	0	0	0	0	0	0	0	0	0	0	0	3	0	0	0	0	0	0	0	0	0	Fungi; unidentified; unidentified; unidentified; unidentified; unidentified; uncultured_fungus
New.ReferenceOTU804	0	0	1	0	0	0	0	0	0	0	0	0	0	0	0	0	0	0	0	0	0	0	0	0	0	0	0	0	0	0	Fungi; unidentified; unidentified; unidentified; unidentified; unidentified; uncultured_fungus
New.ReferenceOTU8089	0	0	0	0	2	0	0	0	0	0	1	0	0	0	0	0	0	0	0	0	0	0	0	0	0	0	0	0	0	0	Fungi; unidentified; unidentified; unidentified; unidentified; unidentified; uncultured_fungus
New.ReferenceOTU812	0	0	0	0	0	0	0	0	0	0	0	1	1	0	0	0	0	0	1	0	0	0	0	0	0	0	1	0	0	0	Fungi; unidentified; unidentified; unidentified; unidentified; unidentified; uncultured_fungus
New.ReferenceOTU890	0	0	1	0	0	0	0	0	0	0	0	0	0	0	0	0	0	0	5	0	0	0	0	0	0	0	0	0	0	0	Fungi; unidentified; unidentified; unidentified; unidentified; unidentified; uncultured_fungus
New.ReferenceOTU897	0	0	0	1	0	0	0	0	0	0	0	0	0	0	0	0	0	0	0	0	0	0	0	0	0	0	0	0	0	0	Fungi; unidentified; unidentified; unidentified; unidentified; unidentified; uncultured_fungus
SH015774.06FU_GU053897_reps_singleton	0	0	0	0	0	0	0	0	0	0	0	0	0	0	0	2	0	0	0	0	0	0	0	0	0	0	0	0	0	0	Fungi; unidentified; unidentified; unidentified; unidentified; unidentified; uncultured_fungus
SH016166.06FU_FJ528725_reps_singleton	0	0	0	0	0	0	0	0	0	0	1	0	0	0	0	0	0	0	0	0	0	0	0	0	1	0	0	0	0	0	Fungi; unidentified; unidentified; unidentified; unidentified; unidentified; uncultured_fungus
SH016184.06FU_EU687001_reps_singleton	0	0	0	0	0	0	0	0	0	0	0	0	0	0	0	0	0	1	0	0	0	0	0	0	2	0	0	0	0	0	Fungi; unidentified; unidentified; unidentified; unidentified; unidentified; uncultured_fungus
SH017947.06FU_HQ873372_reps_singleton	0	0	0	0	0	0	0	0	0	0	0	0	0	0	0	0	0	0	0	0	0	0	0	0	3	0	1	0	0	0	Fungi; unidentified; unidentified; unidentified; unidentified; unidentified; uncultured_fungus
SH024626.06FU_EU179869_reps_singleton	0	0	0	0	0	0	0	0	0	0	0	0	1	0	0	0	0	0	0	0	0	0	0	0	0	0	0	0	0	0	Fungi; unidentified; unidentified; unidentified; unidentified; unidentified; uncultured_fungus
SH030193.06FU_FJ820822_reps_singleton	0	0	0	0	0	0	0	0	0	0	0	0	0	0	0	0	1	0	0	0	0	0	0	0	0	0	0	0	0	0	Fungi; unidentified; unidentified; unidentified; unidentified; unidentified; uncultured_fungus
SH033561.06FU_EU437432_reps_singleton	0	0	0	10	0	0	0	11	0	0	0	0	0	0	0	0	0	0	0	0	0	0	1	0	0	0	0	0	0	0	Fungi; unidentified; unidentified; unidentified; unidentified; unidentified; uncultured_fungus
SH033564.06FU_HQ905446_reps_singleton	0	0	3	1	0	0	0	0	0	0	0	0	0	0	0	0	0	0	0	0	2	0	2	0	0	0	0	1	0	0	Fungi; unidentified; unidentified; unidentified; unidentified; unidentified; uncultured_fungus
SH440464.06FU_JN890179_reps_singleton	0	0	0	0	0	0	0	0	0	0	0	0	0	0	0	0	1	0	0	0	0	0	0	0	0	0	0	0	0	0	Fungi; unidentified; unidentified; unidentified; unidentified; unidentified; uncultured_fungus
New.ReferenceOTU146	1	0	0	0	0	0	0	0	0	0	0	0	0	0	0	0	0	0	0	0	0	0	0	0	0	0	0	0	0	0	Fungi; unidentified; unidentified; unidentified; unidentified; unidentified; uncultured_mycorrhizal_fungus

New.ReferenceOTU3505	0	0	0	0	0	0	0	0	0	0	0	0	0	0	3	0	0	0	0	0	0	0	0	0	0	0	0	0	0	0	Fungi; unidentified; unidentified; unidentified; unidentified; unidentified; uncultured_root_associated_fungus
New.ReferenceOTU7649	0	0	0	0	0	0	0	0	0	0	0	0	0	0	0	0	0	0	0	0	0	0	0	2	0	0	0	0	0	0	Fungi; unidentified; unidentified; unidentified; unidentified; unidentified; uncultured_root_associated_fungus
New.ReferenceOTU1043	1	0	0	0	0	0	0	0	0	0	1	0	0	0	0	0	0	0	0	0	0	0	0	0	0	0	0	0	0	0	Fungi; unidentified; unidentified; unidentified; unidentified; unidentified; uncultured_soil_fungus
New.ReferenceOTU1128	1	0	0	0	0	0	0	0	0	0	0	0	0	0	0	0	0	0	0	0	0	0	0	0	0	0	0	0	0	0	Fungi; unidentified; unidentified; unidentified; unidentified; unidentified; uncultured_soil_fungus
New.ReferenceOTU1283	0	0	0	1	2	0	0	1	0	1	0	0	0	0	0	0	0	0	0	0	0	0	0	0	0	0	0	0	0	0	Fungi; unidentified; unidentified; unidentified; unidentified; unidentified; uncultured_soil_fungus
New.ReferenceOTU1371	0	0	0	0	0	0	0	0	0	0	0	0	0	0	0	0	0	0	0	0	0	0	0	7	0	0	0	0	0	0	Fungi; unidentified; unidentified; unidentified; unidentified; unidentified; uncultured_soil_fungus
New.ReferenceOTU1528	0	0	0	0	0	0	0	0	0	0	0	0	0	0	0	0	0	15	0	0	0	0	0	0	0	0	0	0	0	0	Fungi; unidentified; unidentified; unidentified; unidentified; unidentified; uncultured_soil_fungus
New.ReferenceOTU1765	0	0	0	0	0	0	0	0	0	0	0	0	0	0	0	0	0	0	5	0	0	0	0	0	0	0	0	0	0	0	Fungi; unidentified; unidentified; unidentified; unidentified; unidentified; uncultured_soil_fungus
New.ReferenceOTU1991	0	0	1	0	0	0	0	1	0	0	0	0	0	0	0	0	0	0	0	0	0	0	0	0	0	0	0	0	0	0	Fungi; unidentified; unidentified; unidentified; unidentified; unidentified; uncultured_soil_fungus
New.ReferenceOTU2730	0	0	1	1	2	0	0	9	0	3	0	1	0	0	0	0	0	0	0	0	0	1	0	0	0	0	0	0	0	0	Fungi; unidentified; unidentified; unidentified; unidentified; unidentified; uncultured_soil_fungus
New.ReferenceOTU2803	0	0	0	0	0	0	0	0	0	0	0	0	0	0	0	1	0	0	0	0	0	0	0	0	0	0	0	0	0	0	Fungi; unidentified; unidentified; unidentified; unidentified; unidentified; uncultured_soil_fungus
New.ReferenceOTU2979	0	0	0	0	0	0	0	0	0	0	0	0	0	0	0	0	0	0	0	3	0	0	0	0	0	0	0	0	0	0	Fungi; unidentified; unidentified; unidentified; unidentified; unidentified; uncultured_soil_fungus
New.ReferenceOTU323	0	0	0	0	0	0	0	0	0	0	0	0	0	0	0	0	0	0	0	0	0	1	0	0	0	0	0	0	0	0	Fungi; unidentified; unidentified; unidentified; unidentified; unidentified; uncultured_soil_fungus
New.ReferenceOTU3249	0	0	0	0	0	0	0	0	0	1	0	0	0	0	0	0	0	0	1	0	0	0	0	0	0	0	0	0	0	0	Fungi; unidentified; unidentified; unidentified; unidentified; unidentified; uncultured_soil_fungus
New.ReferenceOTU354	0	1	0	0	0	0	0	0	0	0	0	0	0	0	0	0	0	0	0	0	0	0	0	0	0	0	0	0	0	0	Fungi; unidentified; unidentified; unidentified; unidentified; unidentified; uncultured_soil_fungus
New.ReferenceOTU3927	0	3	0	0	0	0	0	0	0	0	0	0	0	0	0	0	0	0	0	0	0	0	0	0	0	0	0	0	0	0	Fungi; unidentified; unidentified; unidentified; unidentified; unidentified; uncultured_soil_fungus
New.ReferenceOTU4039	0	1	0	0	0	0	0	0	0	0	0	0	0	0	0	0	0	0	0	0	0	0	0	0	0	10	0	0	0	0	Fungi; unidentified; unidentified; unidentified; unidentified; unidentified; uncultured_soil_fungus
New.ReferenceOTU4086	3	0	0	0	0	0	0	0	0	0	0	0	0	0	0	0	0	0	0	0	0	0	0	0	0	0	0	0	0	0	Fungi; unidentified; unidentified; unidentified; unidentified; unidentified; uncultured_soil_fungus
New.ReferenceOTU4195	0	1	0	0	0	0	0	0	0	0	0	0	0	0	0	0	0	0	0	0	0	0	0	0	0	0	0	0	0	0	Fungi; unidentified; unidentified; unidentified; unidentified; unidentified; uncultured_soil_fungus
New.ReferenceOTU4499	0	0	0	0	0	0	0	0	1	0	0	0	0	0	0	0	0	0	0	0	0	0	0	0	0	0	0	0	0	0	Fungi; unidentified; unidentified; unidentified; unidentified; unidentified; uncultured_soil_fungus
New.ReferenceOTU4735	0	0	0	0	0	0	0	1	0	0	1	0	0	0	0	0	0	0	0	0	0	0	0	0	0	0	0	0	0	0	Fungi; unidentified; unidentified; unidentified; unidentified; unidentified; uncultured_soil_fungus
New.ReferenceOTU4973	0	0	0	1	0	0	0	0	0	0	0	0	0	0	0	0	0	0	0	0	0	0	0	0	0	0	0	0	0	0	Fungi; unidentified; unidentified; unidentified; unidentified; unidentified; uncultured_soil_fungus
New.ReferenceOTU5062	1	0	0	0	0	0	0	0	0	0	0	0	0	0	0	0	0	0	0	0	0	0	0	0	0	0	0	0	0	0	Fungi; unidentified; unidentified; unidentified; unidentified; unidentified; uncultured_soil_fungus
New.ReferenceOTU5138	0	0	0	0	0	0	0	0	0	0	0	0	0	0	0	0	0	0	0	0	0	0	0	0	0	0	0	0	6	0	Fungi; unidentified; unidentified; unidentified; unidentified; unidentified; uncultured_soil_fungus
New.ReferenceOTU5422	0	6	0	0	0	0	0	0	0	0	0	0	0	0	0	0	0	0	0	0	0	0	0	0	0	0	0	0	0	0	Fungi; unidentified; unidentified; unidentified; unidentified; unidentified; uncultured_soil_fungus
New.ReferenceOTU5583	0	0	1	0	0	0	1	0	0	0	0	0	0	0	0	0	0	0	0	0	0	0	0	0	0	0	0	0	0	0	Fungi; unidentified; unidentified; unidentified; unidentified; unidentified; uncultured_soil_fungus
New.ReferenceOTU5675	1	0	0	0	0	0	0	0	0	0	0	0	0	0	0	0	0	0	0	0	0	0	0	0	0	0	0	0	0	0	Fungi; unidentified; unidentified; unidentified; unidentified; unidentified; uncultured_soil_fungus
New.ReferenceOTU5791	0	0	0	0	1	0	0	0	0	0	0	0	0	0	0	0	0	0	0	0	0	0	0	0	0	0	0	0	0	0	Fungi; unidentified; unidentified; unidentified; unidentified; unidentified; uncultured_soil_fungus
New.ReferenceOTU6293	0	3	0	1	0	0	0	0	0	0	0	0	0	0	0	0	0	0	0	0	0	0	0	0	0	0	0	0	0	0	Fungi; unidentified; unidentified; unidentified; unidentified; unidentified; uncultured_soil_fungus
New.ReferenceOTU6378	0	0	0	0	0	0	0	0	0	0	0	0	0	0	0	0	0	0	0	0	0	0	0	0	0	0	0	0	0	3	Fungi; unidentified; unidentified; unidentified; unidentified; unidentified; uncultured_soil_fungus
New.ReferenceOTU6557	0	0	0	0	0	0	0	0	0	0	0	0	0	0	0	0	0	0	1	0	0	0	0	0	0	0	0	0	0	0	Fungi; unidentified; unidentified; unidentified; unidentified; unidentified; uncultured_soil_fungus
New.ReferenceOTU6571	1	0	0	0	0	0	0	0	0	0	0	0	0	0	0	0	0	0	0	0	0	0	0	0	0	0	0	0	0	0	Fungi; unidentified; unidentified; unidentified; unidentified; unidentified; uncultured_soil_fungus
New.ReferenceOTU6792	0	0	0	0	0	0	0	0	0	0	0	0	0	0	0	0	0	0	0	0	0	1	0	0	0	0	0	0	0	0	Fungi; unidentified; unidentified; unidentified; unidentified; unidentified; uncultured_soil_fungus
New.ReferenceOTU6871	1	0	0	0	0	0	0	0	0	0	0	0	0	0	0	0	0	0	0	0	0	0	0	0	0	0	0	0	0	0	Fungi; unidentified; unidentified; unidentified; unidentified; unidentified; uncultured_soil_fungus
New.ReferenceOTU6886	1	0	0	0	0	0	0	0	0	0	0	0	0	0	0	0	0	0	0	0	0	0	0	0	0	0	0	0	0	0	Fungi; unidentified; unidentified; unidentified; unidentified; unidentified; uncultured_soil_fungus
New.ReferenceOTU7182	0	1	0	0	0	0	0	0	0	0	0	0	0	0	0	1	0	0	0	0	0	0	0	0	0	0	0	0	0	0	Fungi; unidentified; unidentified; unidentified; unidentified; unidentified; uncultured_soil_fungus
New.ReferenceOTU7232	0	0	0	0	0	0	0	0	0	0	0	0	0	0	0	0	0	0	3	0	0	0	0	0	0	0	0	0	0	0	Fungi; unidentified; unidentified; unidentified; unidentified; unidentified; uncultured_soil_fungus
New.ReferenceOTU73	0	0	0	0	1	0	0	0	0	0	0	0	0	0	0	0	0	0	0	0	0	0	0	0	0	0	0	0	0	0	Fungi; unidentified; unidentified; unidentified; unidentified; unidentified; uncultured_soil_fungus
New.ReferenceOTU7458	0	0	0	0	0	0	0	0	0	0	0	0	0	0	0	0	1	0	0	0	0	0	0	0	0	0	0	0	0	0	Fungi; unidentified; unidentified; unidentified; unidentified; unidentified; uncultured_soil_fungus
New.ReferenceOTU8074	0	0	0	0	0	0	0	0	0	0	0	0	0	0	0	0	0	0	0	0	0	0	0	0	0	0	0	1	0	0	Fungi; unidentified; unidentified; unidentified; unidentified; unidentified; uncultured_soil_fungus
New.ReferenceOTU1382	0	0	0	0	1	0	0	0	0	0	0	0	0	0	0	0	0	0	0	0	0	0	0	0	0	0	0	0	0	0	Fungi; unidentified; unidentified; unidentified; unidentified; unidentified; uncultured_zygomycete
New.ReferenceOTU2099	0	0	0	0	2	0	0	0	0	0	0	0	0	0	0	0	0	0	0	0	0	0	0	0	0	0	0	0	0	0	Fungi; unidentified; unidentified; unidentified; unidentified; unidentified; uncultured_zygomycete
New.ReferenceOTU3890	0	0	0	0	0	0	0	0	0	5	0	0	0	0	0	0	0	0	0	0	0	0	0	0	0	0	0	0	0	0	Fungi; unidentified; unidentified; unidentified; unidentified; unidentified; uncultured_zygomycete
New.ReferenceOTU1431	0	0	0	0	0	0	0	0	0	0	0	0	0	0	0	0	0	0	0	0	0	0	0	0	0	2	0	0	1	0	Fungi; Zygomycota; Incertae_sedis; Endogonales; unidentified; unidentified; Endogonales_sp
New.ReferenceOTU2564	0	0	0	0	0	0	0	0	0	0	0	0	0	0	0	0	0	0	0	0	0	0	0	0	0	3	0	0	2	0	Fungi; Zygomycota; Incertae_sedis; Endogonales; unidentified; unidentified; Endogonales_sp
New.ReferenceOTU3223	0	0	0	0	0	0	0	0	0	0	0	0	0	0	0	1	0	0	0	0	0	0	0	0	0	0	0	0	0	0	Fungi; Zygomycota; Incertae_sedis; Mortierellales; Mortierellaceae; unidentified; Mortierellaceae_sp
New.ReferenceOTU2128	2	0	0	0	0	0	0	0	0	0	0	0	0	0	0	0	0	0	0	0	0	0	0	0	0	0	0	0	0	0	Fungi; Zygomycota; Incertae_sedis; Mortierellales; unidentified; unidentified; Mortierellales_sp
New.ReferenceOTU6437	0	0	0	0	0	0	0	0	0	0	0	0	0	1	0	0	0	0	0	0	0	0	0	0	0	0	0	0	0	0	Fungi; Zygomycota; Incertae_sedis; Mortierellales; unidentified; unidentified; Mortierellales_sp
New.ReferenceOTU7691	0	0	0	0	0	0	0	0	0	0	0	0	0	0	1	0	0	0	0	0	0	0	0	0	0	0	0	0	0	0	Fungi; Zygomycota; Incertae_sedis; Mortierellales; unidentified; unidentified; Mortierellales_sp
New.CleanUp.ReferenceOTU788	0	0	0	0	0	0	0	0	0	0	0	0	0	0	0	0	0	0	0	0	0	0	0	0	0	0	0	0	1	0	Fungi; Zygomycota; unidentified; unidentified; unidentified; unidentified; Mucoromycotina_sp_MIB_8354
New.ReferenceOTU4539	1	0	0	0	0	0	0	0	0	0	0	0	0	0	0	0	0	0	0	0	0	0	0	0	0	0	0	0	0	0	Fungi; Zygomycota; unidentified; unidentified; unidentified; unidentified; Mucoromycotina_sp_MIB_8354
