# Supplementary material for: Intensive tropical land use massively shifts soil fungal communities
Source: Sci Rep. 2019 Mar 4;9:3403. doi: 10.1038/s41598-019-39829-4 (PMC6399230; doi:10.1038/s41598-019-39829-4)
Supplement: Supplementary file 3 — Supplementary data S3 [file 41598_2019_39829_MOESM3_ESM.pdf]

Intensive tropical land use massively shifts soil fungal communities

Nicole Brinkmann[1,#,*], Dominik Schneider[2,#], Josephine Sahner[1], Johannes Ballauff[1], Nur Edy[1,3], Henry Barus[3], Bambang Irawan[4], Sri Wilarso Budi[5], Matin Qaim[6], Rolf Daniel[2], Andrea Polle[1]

[1]Forest Botany and Tree Physiology, University of Goettingen, Germany, [2]Genomic and Applied Microbiology and Göttingen Genomics Laboratory, University of Goettingen, Germany, [3]Department of Agrotechnology, Faculty of Agriculture, Tadulako University, Indonesia, [4]Department of Forestry, University of Jambi, Indonesia, [5]Department of Silviculture, Faculty of Forestry, Bogor Agriculture University, Bogor, Indonesia, [6]Department of Agricultural Economics and Rural Development, University of Goettingen, Germany

[#]These authors contributed equally to this work

*Correspondence: Nicole Brinkmann, Forest Botany and Tree Physiology, University of Goettingen, Büsgenweg 2, 37077 Goettingen, Germany, nbrinkm3@gwdg.de, Tel.: +49 551 39 9745, Fax: +49 551 39 22705

The authors declare no conflict of interest

Supplementary data S3. Environmental variables analyzed to explain possible dissimilarities in fungal community composition among different land use systems using the envfit function of the vegan package in R. Permutation: free. Number of permutations: 999. Significant codes: 0, ‘***’; 0.001, ‘**’; 0.01, ‘*’; 0.05, ‘.’.

Vectors	NMDS12	NMDS23	r2	P value	Significance	Data source
land use intensity (LUI)	0.77014	0.63788	0.4707	0.001	***	this study
plant biomass	-0.7654	-0.64356	0.8181	0.001	***	Drescher et al. 2015
plant species richness	-0.85236	-0.52296	0.8218	0.001	***	Drescher et al. 2015
biomass of fine roots (dw_fr)	-0.63856	-0.76957	0.4014	0.003	**	Sahner et al. 2015
litter carbon concentration (C_litter)	-0.52655	-0.85015	0.3978	0.006	**	Sahner et al. 2015
soil pH value	0.82306	0.56795	0.2968	0.012	*	Allen et al. 2015
litter nitrogen concentration (N_litter)	0.07883	-0.99689	0.2101	0.058	.	Sahner et al. 2015
soil magnesium concentration (Mg_soil)	-0.07458	-0.99721	0.2042	0.059	.	Sahner et al. 2015
operational taxonomic units (OTU)	0.99972	0.02348	0.1862	0.087		this study
distorted root tips (dead_rt)	0.14411	0.98956	0.1323	0.174		Sahner et al. 2015
soil potassium concentration (K_soil)	-0.24127	-0.97046	0.1236	0.213		Sahner et al. 2015
soil carbon concentration (C_soil)	-0.94886	-0.3157	0.0348	0.656		Sahner et al. 2015
soil nitrogen concentration (N_soil)	-0.34109	-0.94003	0.0309	0.668		Sahner et al. 2015
soil calcium concentration (Ca_soil)	0.00347	-0.99999	0.0265	0.712		Sahner et al. 2015
available phosphorous in soil (avail_P_soil)	0.48186	-0.87625	0.0007	0.996		Sahner et al. 2015
soil moisture	-0.09523	-0.99546	0.1514	0.132		Allen et al. 2015

Centroid	NMDS1	NMDS2	
LS_LUBukit_Jungle_rubber	-0.0813	-0.0675	
LS_LUBukit_Oil_palm	0.0464	0.1567	
LS_LUBukit_Rainforest	-0.1339	-0.2642	
LS_LUBukit_Rubber_plantations	0.2573	-0.0107	
LS_LUHarapan_Jungle_rubber	-0.1078	0.1206	
LS_LUHarapan_Oil_palm	0.073	0.0383	
LS_LUHarapan_Rainforest	-0.4001	0.0142	
LS_LUHarapan_Rubber_plantations	0.2134	-0.0775	
LU_Jungle_rubber	-0.0972	0.0454	
LU_Oil_palm	0.0616	0.089	
LU_Rainforest	-0.286	-0.1051	
LU_Rubber_plantations	0.2354	-0.0441	
LS_Bukit	0.0503	-0.0417	
LS_Harapan	-0.0519	0.0174	
Goodness of fit:	r2	Pr(>r)	
LS_LU	0.8252	0.001	***
LU	0.65	0.001	***
LS	0.0485	0.283	
