# Supplementary material for: Intensive tropical land use massively shifts soil fungal communities
Source: Sci Rep. 2019 Mar 4;9:3403. doi: 10.1038/s41598-019-39829-4 (PMC6399230; doi:10.1038/s41598-019-39829-4)
Supplement: Supplementary file 4 — Supplementary data S4 [file 41598_2019_39829_MOESM4_ESM.pdf]

Intensive tropical land use massively shifts soil fungal communities

Nicole Brinkmann[1,#,*], Dominik Schneider[2,#], Josephine Sahner[1], Johannes Ballauff[1], Nur Edy[1,3], Henry Barus[3], Bambang Irawan[4], Sri Wilarso Budi[5], Matin Qaim[6], Rolf Daniel[2], Andrea Polle[1]

[1]Forest Botany and Tree Physiology, University of Goettingen, Germany, [2]Genomic and Applied Microbiology and Göttingen Genomics Laboratory, University of Goettingen, Germany, [3]Department of Agrotechnology, Faculty of Agriculture, Tadulako University, Indonesia, [4]Department of Forestry, University of Jambi, Indonesia, [5]Department of Silviculture, Faculty of Forestry, Bogor Agriculture University, Bogor, Indonesia, [6]Department of Agricultural Economics and Rural Development, University of Goettingen, Germany

[#]These authors contributed equally to this work

*Correspondence: Nicole Brinkmann, Forest Botany and Tree Physiology, University of Goettingen, Büsgenweg 2, 37077 Goettingen, Germany, nbrinkm3@gwdg.de, Tel.: +49 551 39 9745, Fax: +49 551 39 22705

The authors declare no conflict of interest

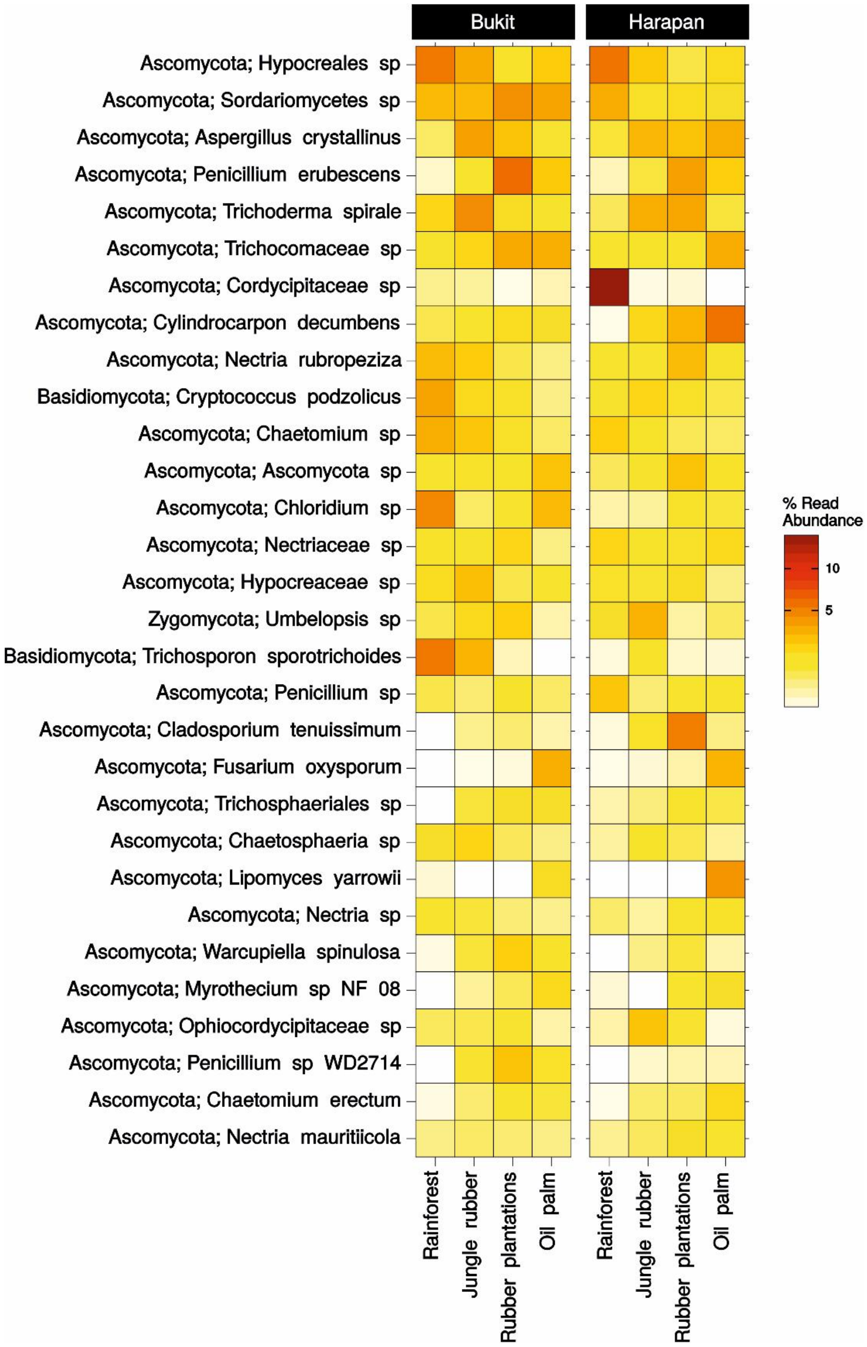

Supplementary data S4. Relative abundance of the top 30 (average abundance among all plots) fungal species in the landscapes and four land use systems. Unidentified fungal OTUs were removed beforehand (Fungi; unidentified; unidentified; unidentified; unidentified; unidentified; uncultured_fungus). Abundance color is scaled square root.
