# Supplementary material for: Intensive tropical land use massively shifts soil fungal communities
Source: Sci Rep. 2019 Mar 4;9:3403. doi: 10.1038/s41598-019-39829-4 (PMC6399230; doi:10.1038/s41598-019-39829-4)
Supplement: Supplementary file 5 — Supplementary data S5 [file 41598_2019_39829_MOESM5_ESM.pdf]

Intensive tropical land use massively shifts soil fungal communities

Nicole Brinkmann[1,#,*], Dominik Schneider[2,#], Josephine Sahner[1], Johannes Ballauff[1], Nur Edy[1,3], Henry Barus[3], Bambang Irawan[4], Sri Wilarso Budi[5], Matin Qaim[6], Rolf Daniel[2], Andrea Polle[1]

[1]Forest Botany and Tree Physiology, University of Goettingen, Germany, [2]Genomic and Applied Microbiology and Göttingen Genomics Laboratory, University of Goettingen, Germany, [3]Department of Agrotechnology, Faculty of Agriculture, Tadulako University, Indonesia, [4]Department of Forestry, University of Jambi, Indonesia, [5]Department of Silviculture, Faculty of Forestry, Bogor Agriculture University, Bogor, Indonesia, [6]Department of Agricultural Economics and Rural Development, University of Goettingen, Germany

[#]These authors contributed equally to this work

*Correspondence: Nicole Brinkmann, Forest Botany and Tree Physiology, University of Goettingen, Büsgenweg 2, 37077 Goettingen, Germany, nbrinkm3@gwdg.de, Tel.: +49 551 39 9745, Fax: +49 551 39 22705

The authors declare no conflict of interest

Supplementary data 5. Fungal genera significantly associated with distinct land use systems representing a broad taxonomic spectrum.
Phylum-, order-, family and genus level and their presence/absence in distinct land use systems and corresponding p values are given.
Classification of fungal OTUs according to their trophic modus using the FUNGuild database.

Phylum	Order	Family	Genus	F	J	R	O	p.value	Trophic mode
Ascomycota	Incertae_sedis	Myxotrichaceae	Oidiodendron	x	x	-	-	0.003	Symbiotroph
Ascomycota	Hypocreales	Bionectriaceae	Bionectria	x	x	-	-	0.016	Pathotroph
Basidiomycota	Polyporales	Ganodermataceae	Ganoderma	x	x	-	-	0.03	Pathotroph
Ascomycota	Incertae_sedis	Incertae_sedis	Amplistroma	x	-	-	-	0.002	Unknown
Ascomycota	Hypocreales	unidentified	unidentified	x	-	-	-	0.002	Saprotroph
Ascomycota	Hypocreales	Cordycipitaceae	unidentified	x	-	-	-	0.003	Saprotroph
Basidiomycota	Sporidiobolales	unidentified	unidentified	x	-	-	-	0.003	Pathotroph
Ascomycota	Saccharomycetales	unidentified	unidentified	x	-	-	-	0.005	Saprotroph
Basidiomycota	Dacrymycetales	Dacrymycetaceae	Dacryopinax	x	-	-	-	0.005	Saprotroph
Ascomycota	Ophiostomatales	Ophiostomataceae	Sporothrix	x	-	-	-	0.007	Pathotroph
Zygomycota	Endogonales	unidentified	unidentified	x	-	-	-	0.009	Unknown
Basidiomycota	Agaricales	Agaricaceae	Calvatia	x	-	-	-	0.017	Saprotroph
Ascomycota	Onygenales	Onygenaceae	Auxarthron	x	-	-	-	0.022	Saprotroph
Glomeromycota	Archaeosporales	Archaeosporaceae	Archaeospora	x	-	-	-	0.023	Symbiotroph
Basidiomycota	Agaricales	Agaricaceae	unidentified	x	-	-	-	0.027	Saprotroph
Basidiomycota	Cantharellales	Tulasnellaceae	unidentified	x	-	-	-	0.037	Unknown
Basidiomycota	Geastrales	Geastraceae	Schenella	x	-	-	-	0.037	Saprotroph
Basidiomycota	Boletales	Sclerodermataceae	Scleroderma	x	-	-	-	0.041	Symbiotroph
Ascomycota	Coniochaetales	unidentified	unidentified	x	-	-	-	0.043	Saprotroph
Basidiomycota	Agaricales	Agaricaceae	Micropsalliota	x	-	-	-	0.043	Saprotroph
Ascomycota	Onygenales	Gymnoascaceae	Gymnascella	x	-	-	-	0.048	Saprotroph
Ascomycota	unidentified	unidentified	unidentified	-	x	x	x	0.006	Unknown
Ascomycota	Incertae_sedis	Incertae_sedis	Ochroconis	-	x	x	-	0.001	Saprotroph
Ascomycota	Hypocreales	Hypocreaceae	Trichoderma	-	x	x	-	0.02	Saprotroph
Ascomycota	Botryosphaeriales	Botryosphaeriaceae	Lasiodiplodia	-	x	x	-	0.025	Pathotroph
Ascomycota	Xylariales	Xylariaceae	Hypoxylon	-	x	-	-	0.001	Saprotroph
Ascomycota	Pleosporales	Pleosporaceae	Alternaria	-	x	-	-	0.005	Saprotroph
Ascomycota	Xylariales	Xylariaceae	Biscogniauxia	-	x	-	-	0.013	Saprotroph
Ascomycota	Coniochaetales	Coniochaetaceae	Lecythophora	-	x	-	-	0.014	Pathotroph
Ascomycota	Eurotiales	Thermoascaceae	unidentified	-	x	-	-	0.035	Saprotroph
Glomeromycota	Diversisporales	Acaulosporaceae	unidentified	-	x	-	-	0.037	Symbiotroph
Ascomycota	Leotiales	Leotiaceae	unidentified	-	x	-	-	0.04	Saprotroph
Basidiomycota	Agaricales	Marasmiaceae	Rectipilus	-	x	-	-	0.042	Saprotroph
Zygomycota	Mortierellales	unidentified	unidentified	-	x	-	-	0.046	Saprotroph
Ascomycota	Pleosporales	Tubeufiaceae	Tubeufia	-	x	-	-	0.047	Saprotroph
Ascomycota	Hypocreales	Incertae_sedis	Myrothecium	-	-	x	x	0.001	Saprotroph
Ascomycota	Trichosphaeriales	Incertae_sedis	unidentified	-	-	x	x	0.005	Saprotroph
Ascomycota	Eurotiales	Trichocomaceae	Merimbla	-	-	x	x	0.039	Saprotroph
Ascomycota	Eurotiales	Trichocomaceae	Penicillium	-	-	x	-	0.003	Saprotroph
Ascomycota	Pleosporales	Incertae_sedis	Letendraea	-	-	x	-	0.005	Unknown
Ascomycota	Incertae_sedis	Incertae_sedis	Leptodiscella	-	-	x	-	0.005	Saprotroph
Ascomycota	Capnodiales	Davidiellaceae	Cladosporium	-	-	x	-	0.01	Pathotroph
Basidiomycota	Tremellales	Incertae_sedis	unidentified	-	-	x	-	0.012	Pathotroph
Ascomycota	Trichosphaeriales	Incertae_sedis	Khuskia	-	-	x	-	0.014	Saprotroph
Ascomycota	Pleosporales	Massarinaceae	Massarina	-	-	x	-	0.023	Saprotroph
Ascomycota	Pleosporales	Leptosphaeriaceae	Coniothyrium	-	-	x	-	0.029	Saprotroph
Ascomycota	Pezizales	Pezizaceae	unidentified	-	-	x	-	0.045	Unknown
Ascomycota	Hypocreales	Clavicipitaceae	unidentified	-	-	x	-	0.046	Saprotroph
Ascomycota	Boliniales	Boliniaceae	Camarops	-	-	x	-	0.048	Saprotroph
Ascomycota	Pleosporales	Incertae_sedis	Berkleasmium	-	-	-	x	0.001	Unknown
Ascomycota	Pleosporales	Montagnulaceae	Paraconiothyrium	-	-	-	x	0.001	Saprotroph
Ascomycota	Hypocreales	Nectriaceae	Fusarium	-	-	-	x	0.001	Pathotroph
Ascomycota	Incertae_sedis	Incertae_sedis	Asteromella	-	-	-	x	0.002	Pathotroph
Ascomycota	Pleosporales	Sporormiaceae	Westerdykella	-	-	-	x	0.002	Saprotroph
Ascomycota	Saccharomycetales	Lipomycetaceae	Lipomyces	-	-	-	x	0.002	Saprotroph
Ascomycota	Incertae_sedis	Apiosporaceae	Arthrinium	-	-	-	x	0.002	Saprotroph
Ascomycota	Eurotiales	unidentified	unidentified	-	-	-	x	0.005	Unknown
Ascomycota	Pleosporales	Lophiostomataceae	Lophiostoma	-	-	-	x	0.006	Saprotroph
Ascomycota	Pleosporales	Incertae_sedis	Stagonosporopsis	-	-	-	x	0.007	Pathotroph
Ascomycota	Chaetothyriales	Herpotrichiellaceae	Exophiala	-	-	-	x	0.007	Saprotroph
Ascomycota	Pleosporales	Cucurbitariaceae	Pyrenochaetopsis	-	-	-	x	0.009	Saprotroph
Ascomycota	Pleosporales	Incertae_sedis	Pyrenochaeta	-	-	-	x	0.01	Saprotroph
Ascomycota	Lecanorales	Ramalinaceae	Bacidina	-	-	-	x	0.01	Symbiotroph
Ascomycota	Microascales	Ceratocystidaceae	Ceratocystis	-	-	-	x	0.012	Pathotroph
Ascomycota	Pleosporales	Arthopyreniaceae	unidentified	-	-	-	x	0.014	Unknown
Ascomycota	Pleosporales	Corynesporascaceae	Corynespora	-	-	-	x	0.014	Saprotroph
Ascomycota	Pleosporales	Incertae_sedis	Phoma	-	-	-	x	0.015	Pathotroph
Ascomycota	Chaetothyriales	Herpotrichiellaceae	Cladophialophora	-	-	-	x	0.018	Saprotroph
Ascomycota	Eurotiales	Trichocomaceae	Phialosimplex	-	-	-	x	0.022	Saprotroph
Ascomycota	Pleosporales	Montagnulaceae	Microsphaeropsis	-	-	-	x	0.026	Saprotroph
Ascomycota	Chaetothyriales	unidentified	unidentified	-	-	-	x	0.035	Saprotroph
Ascomycota	Eurotiales	Trichocomaceae	Sagenomella	-	-	-	x	0.041	Saprotroph
Ascomycota	Pleosporales	Dacampiaceae	Munkovalsaria	-	-	-	x	0.047	Saprotroph
Ascomycota	Incertae_sedis	Incertae_sedis	Savoryella	-	-	-	x	0.047	Saprotroph
